# The Pharmacogenetic Footprint of ACE Inhibition: A Population-Based Metabolomics Study

**DOI:** 10.1371/journal.pone.0153163

**Published:** 2016-04-27

**Authors:** Elisabeth Altmaier, Cristina Menni, Margit Heier, Christa Meisinger, Barbara Thorand, Jan Quell, Michael Kobl, Werner Römisch-Margl, Ana M. Valdes, Massimo Mangino, Melanie Waldenberger, Konstantin Strauch, Thomas Illig, Jerzy Adamski, Tim Spector, Christian Gieger, Karsten Suhre, Gabi Kastenmüller

**Affiliations:** 1 Research Unit of Molecular Epidemiology, Helmholtz Zentrum München, German Research Center for Environmental Health, Ingolstädter Landstr. 1, D-85764 Neuherberg, Germany; 2 Department of Twin Research & Genetic Epidemiology, King’s College London, London SE1 7EH, United Kingdom; 3 Institute of Epidemiology II, Helmholtz Zentrum München, German Research Center for Environmental Health, Ingolstädter Landstr. 1, D-85764 Neuherberg, Germany; 4 Institute of Bioinformatics and Systems Biology, Helmholtz Zentrum München, German Research Center for Environmental Health, Ingolstädter Landstr. 1, D-85764 Neuherberg, Germany; 5 Institute of Genetic Epidemiology, Helmholtz Zentrum München, German Research Center for Environmental Health, Ingolstädter Landstr. 1, D-85764 Neuherberg, Germany; 6 Institute of Medical Informatics, Biometry and Epidemiology, Chair of Genetic Epidemiology, Ludwig-Maximilians-Universität, Marchionistr. 15, D-81377 München, Germany; 7 Hannover Unified Biobank, Hannover Medical School, Carl-Neuberg-Str. 1, D-30625 Hannover, Germany; 8 Institute of Human Genetics, Hannover Medical School, Carl-Neuberg-Str. 1, D-30625 Hanover, Germany; 9 Institute of Experimental Genetics, Genome Analysis Center, Helmholtz Zentrum München, German Research Center for Environmental Health, Ingolstädter Landstr. 1, D-85764 Neuherberg, Germany; 10 Institute of Experimental Genetics, Life and Food Science Center Weihenstephan, Technische Universität München, D-85354 Freising, Germany; 11 German Center for Diabetes Research (DZD e.V.), Ingolstädter Landstr. 1, D-85764 Neuherberg, Germany; 12 Department of Physiology and Biophysics, Weill Cornell Medical College in Qatar, Education City, Qatar Foundation, PO Box 24144, Doha, State of Qatar; Escola Paulista de Medicina, BRAZIL

## Abstract

Angiotensin-I-converting enzyme (ACE) inhibitors are an important class of antihypertensives whose action on the human organism is still not fully understood. Although it is known that ACE especially cleaves COOH-terminal dipeptides from active polypeptides, the whole range of substrates and products is still unknown. When analyzing the action of ACE inhibitors, effects of genetic variation on metabolism need to be considered since genetic variance in the ACE gene locus was found to be associated with ACE-concentration in blood as well as with changes in the metabolic profiles of a general population. To investigate the interactions between genetic variance at the ACE-locus and the influence of ACE-therapy on the metabolic status we analyzed 517 metabolites in 1,361 participants from the KORA F4 study. We replicated our results in 1,964 individuals from TwinsUK. We observed differences in the concentration of five dipeptides and three ratios of di- and oligopeptides between ACE inhibitor users and non-users that were genotype dependent. Such changes in the concentration affected major homozygotes, and to a lesser extent heterozygotes, while minor homozygotes showed no or only small changes in the metabolite status. Two of these resulting dipeptides, namely aspartylphenylalanine and phenylalanylserine, showed significant associations with blood pressure which qualifies them—and perhaps also the other dipeptides—as readouts of ACE-activity. Since so far ACE activity measurement is substrate specific due to the usage of only one oligopeptide, taking several dipeptides as potential products of ACE into account may provide a broader picture of the ACE activity.

## Introduction

Hypertension is a highly prevalent risk factor for the development of cardiovascular diseases and atherosclerosis as well as for renal failure. Antihypertensive medication ranks among the most frequently prescribed drugs in medical practice.

An important class of antihypertensive agents are angiotensin-I-converting enzyme (ACE) inhibitors. ACE catalyzes the conversion of angiotensin-I to angiotensin-II which causes the muscles surrounding blood vessels to contract, thereby restricting the blood flow and increasing blood pressure. ACE inhibitors diminish the action of this enzyme and, thus, blood vessels dilate and blood pressure is reduced [1, 2].

ACE acts not only on angiotensin I cleaving the dipeptide histidylleucine (His-Leu), but also cleaves COOH-terminal dipeptides from several other active peptides [3]. These peptides include for example the vasodilator bradykinin [4, 5], the neuropeptide cholecystokinin-8 [6], which acts on the gastrointestinal system, different forms of the opioid peptide encephalin [7, 8] as well as the neuropeptides substance P and neurotensin [9]. While the whole range of substrates and products is still unknown, it has been suggested that ACE, and thus ACE inhibitors, might have a more general impact on biologically active peptides and subsequently on metabolism than previously recognized [10, 11].

Applying a metabolomics approach, we recently investigated the effect of antihypertensives on blood metabolomes in the general population by analyzing the association of ACE inhibitor intake and metabolite levels in serum samples from the KORA (Cooperative Health Research in the Region of Augsburg) study [12]. The analysis yielded several significant associations with oligopeptides including des-Arg(9)-bradykinin and aspartylphenylalanine (Asp-Phe), a substrate and a product of peptide cleavage catalyzed by ACE.

In recent genome-wide association studies with traits from high-throughput metabolite screening (mGWAS), Asp-Phe and several other dipeptides had been found to associate with single nucleotide polymorphisms (SNPs) in the ACE locus [13–15]. Though His-Leu was not among the quantified metabolites, these findings are consistent with results from Chung et al. [16], who reported that the same genetic variants influence the activity of ACE in blood as measured through an assay that specifically targets the cleavage of p-hydroxyhippuryl-L-histidyl-L-leucine. As a consequence, genetic factors could have potential implications for the individual response to ACE inhibitor treatment and could also modulate the broader metabolic effects of ACE inhibitors.

In this study we aimed to find genotype dependent differences in the metabolic response to ACE inhibitor intake using metabolites as readout of the ACE inhibitor’s effect. To this end, we used a mass spectrometry based non-targeted metabolomics approach that includes measurements of several di- and oligopeptides. We analyzed the metabolic profiles of 1,361 participants from the KORA (Cooperative Health Research in the Region of Augsburg) study and 1,964 individuals from the TwinsUK cohort. Using such a population based approach provides the possibility to find hypotheses that reflect the drug response under everyday life conditions.

## Methods

### Study populations

The research platform KORA conducts population-based surveys and subsequent follow-up studies in the fields of health care research, health economics, epidemiology and genetics. A multitude of different parameters is provided, including medical history. Here, we analyzed a dataset that was taken from the F4 study, which was conducted in 2006−2008 as a follow-up of the fourth KORA survey (S4; 1999−2001). From this KORA F4 population, the metabolic profiles of 1,768 participants, aged between 32 and 77 years were measured. To determine the use of medication participants were asked to bring all medications along with them, which they had taken during the past 7 days preceding the interview. The medical staff registered the medication data online using the IDOM software [17]. The drug classes, including the class “ACE inhibitors”, were categorized according to the Anatomical Therapeutical Chemical (ATC) classification index recommended by the World Health Organization (WHO; http://www.whocc.no/atc/structure_and_principles/). Out of the 1,768 KORA F4 participants 282 individuals were ACE inhibitor users, while 1,079 individuals did not take any antihypertensives at all. These 1361 individuals were used for analysis. The characteristics of ACE users vs. non-users of any antihypertensive drugs are shown in *S1 Table*. A pie-chart of the agents within the class “ACE inhibitors” is given in the *S1 Fig.*

The TwinsUK cohort is an adult UK twin registry composed of mostly women aged 18 to 85. Twins were recruited from the general UK population through national media campaigns and were shown to have similar disease-related and lifestyle characteristics as population-based singletons in the same age group [18]. The 1,964 individuals analyzed in this study were 99.7% female in the age range of 23 to 84 years (mean of 58 years). In total, 166 ACE inhibitor users and 1,798 individuals not on any antihypertensive treatments were included.

Informed written consent was obtained from each participant of both populations and all study protocols were approved by the local ethics committees (Bayerische Landesärztekammer for KORA and Guy’s and St. Thomas’ Hospital Ethics Committee for TwinsUK).

### Blood samples

For both populations, we collected blood serum of the study participants. To avoid variation due to circadian rhythm, KORA blood samples were taken after overnight fasting (at least 8 hours) in the morning between 8 and 10:30 am. Medication was taken in the morning as usual. After drawing the material into serum gel tubes, it was gently inverted twice and subsequently rested 30 min at room temperature (18–25°C) to obtain complete coagulation. The material was then centrifuged for 10 min (2,750g at 15°C). Serum was aliquoted and stored at 4°C, after which it was deep frozen to -80°C on the same day until analysis of the metabolites.

For the TwinsUK study, blood was drawn after at least 6 h of fasting. The samples were immediately inverted three times, followed by 40 min of resting at 4°C to obtain complete coagulation. After centrifuging the samples for 10 min at 2,000g serum was collected. Four aliquots of 1.5 ml were placed into skirted microcentrifuge tubes and then stored at −45°C until sampling.

### Metabolite profiling and metabolite spectrum

The metabolite profiles were measured by the US-company Metabolon Inc., a commercial supplier of metabolomic analyses. Their platform integrates the chemical analysis, including identification and relative quantification, data reduction, and quality assurance components of the process. One gas chromatography/mass spectrometry (GC/MS) injection and two separate ultrahigh performance liquid chromatography/tandem mass spectrometry (UHPLC/MS/MS; positive and negative mode) injections were done on this platform.

The GC/MS platform utilized a Thermo-Finnigan Trace DSQ MS, while for UHPLC/MS/MS analysis a Waters Acquity UPLC and a ThermoFisher LTQ mass spectrometer were used.

A standard library containing retention time, molecular mass to charge ratio (m/z), preferred in-source fragments and adducts as well as their associated MS/MS spectra for all molecules in the library, subsequently allowed to identify a multitude of metabolites based on the measured spectra.

Metabolon applies an analytic approach which is semi-quantitative. This means that the injected standards are not used to calculate the metabolite concentrations but mainly to determine the retention time. Thus, a relative intensity is measured and the measurement is sensitive to instrument parameters as well as fluctuations caused by maintenances such as column change. Since these fluctuations are run day dependent, a run day normalization of the metabolic data was done: For each individual and each metabolite the data was first divided by the day median of the respective metabolite and then multiplied for the overall median of this metabolite.

Metabolon showed that their analytical platform is able to perform relative quantitative analysis of analytical data in a high-throughput mode and that it identifies a broad spectrum of molecules with a high degree of confidence [19]. The measured panel includes 517 metabolites from many relevant classes such as carbohydrates, acylcarnitines, glyceropospholipids, lipids, amino acids, small peptides, cofactors and vitamins, nucleotides and xenobiotics. A third of these metabolites, marked with an identifier starting with the letter “X” followed by five digits, are currently identified by their mass, and MS/MS fragmentation and chromatographic retention time, but their biochemical identity is unknown. A list of all metabolites is given in the–*S2 Table.*

### Genotyping and imputation

For all used samples from KORA, genotype information derived from the Affymetrix Axiom Chip was available. Genotype calling was conducted with the Affymetrix Software and variant annotation was carried out according to NCBI build 37. For quality control, we applied the criteria of at least 97% call rate per person and 98% call rate per SNP, HWE (p-value ≥5×10^−6^) and a minor allele frequency of ≥0.01. Pre-phasing was done with SHAPEIT v2. Imputation was performed with IMPUTE v2.3.0 based on the 1000G phase1 (v3) reference panel.

### Statistical analysis

For the statistical analysis the statistical analysis system R (http://www.r-project.org/) was used.

Besides the metabolite values, the ratios between the values of all possible pairs of metabolites were used in the analysis. This metabolic data was inverse normalized since the metabolite concentrations didn’t show a normal distribution. To avoid false-positive associations, data points of metabolic traits that lay more than three standard deviations off the mean were excluded from further analysis.

For the identification of relevant ACE-SNPs in KORA, we used the results of two former studies on KORA individuals. In a systems approach combining genetic and metabolic information to identify unknown metabolites, the ACE-SNP rs4343 showed several significant associations with metabolite changes [20]. Another KORA study on human metabolic individuality revealed rs4329 as an ACE-SNP influencing the metabotype [14]. Proxy SNPs for rs4343 and rs4329 based on a linkage disequilibrium threshold of at least 0.9 (genome assembly: GRCh37; variant set: 1000 Genomes, Phase 3 v5; genome annotation: Ensemble 77; http://snipa.org [21]) were also included in the analysis.

We did a two-step approach to test possible interactions between ACE-SNPs and ACE inhibitor intake.

In the first analysis step a linear regression test with cofactors age and sex was used to model the association of each metabolite and metabolite ratio with each of the 25 ACE-SNPs (assuming an additive genetic model). The linear regression tests were separately applied for ACE inhibitor users and individuals not taking any antihypertensives (non-users). To control for the effect of testing multiple hypotheses we used the Bonferroni correction. Thus, only associations with a p-value smaller than 1.4x10^-8^ (0.05/(517*(517/2) metabolite ratios + 517 metabolites)/25 SNPs) and with a p-gain greater than 517 (for ratios) were considered as significant. The p-gain is a measure to determine whether the ratio between two metabolite concentrations carries more information than the two corresponding metabolites alone [22].

Metabolites and metabolite ratios with significant associations were only found for the group of non-users, while the corresponding tests in the group of ACE inhibitor users were not significant. Based on this result we investigated if genotypes in the non-users group showed metabolite concentrations that were significantly different from the metabolite levels of all ACE inhibitor users. Thus, in a second analysis step we applied a linear regression test to each genotype group of the non-users to compare their metabolite levels with those of all ACE inhibitor users.

Metabolites with significant differences between a genotype group of non-users and all users were tested for replication in the TwinsUK population. From the three candidate ratios only aspartylphenylalanine/HWESASXX was available in the replication cohort as for the remaining ratios were not in the TwinsUK metabolomics data set. As in KORA all ACE-SNPs showed the same effect, we only attempted to replicate the SNP rs4329. For TwinsUK we used linear mixed-effects models to account for familial relationship.

## Results

### Genotypes at the ACE-SNPs only affect peptide levels in non-users

In a first step, we analyzed the KORA F4 metabolite data for associations with the ACE-SNPs separately for ACE inhibitor users and participants who did not take any antihypertensives (non-users). As an example, the results for ACE-SNP rs4329 are given in *Table 1*.

**Table 1:** Significant results from the KORA F4 analysis for rs4329.

metabolic trait	n (non-users)	beta (non-users)	p-value (non-users)	p-gain (non-users)	n (users)	beta (users)	p-value (users)	n AA	beta AA	p-value AA	n AG	beta AG	p-value AG	n GG	beta GG	p-value GG
aspartylphenylalanine	1013	-0.306	6.04 x10^-11^		265	-0.053	0.556	284	-0.762	1.61x10^-15^	521	-0.497	1.92 x10^-09^	208	-0.120	0.235
aspartylphenylalanine / HWESASXX	981	-0.356	5.33 x10^-14^	1133.15	261	-0.106	0.248	274	-1.000	1.84 x10^-25^	506	-0.702	3.29 x10^-17^	201	-0.252	0.014
aspartylphenylalanine / X11805	1009	-0.503	9.16 x10^-28^	6.60x10^16^	259	-0.216	0.019	282	-1.491	7.25 x10^-52^	520	-1.050	4.57 x10^-42^	207	-0.638	2.81 x10^-11^
aspartylphenylalanine / phenylalanylleucine (X14450)	872	-0.407	4.71 x10^-16^	128329.95	222	-0.128	0.197	245	-1.198	2.40 x10^-31^	456	-0.830	1.28 x10^-21^	171	-0.437	1.81 x10^-05^
X14086	1024	-0.347	6.39 x10^-14^		259	0.027	0.767	286	-1.074	2.26 x10^-28^	526	-0.828	2.03 x10^-24^	212	-0.494	1.16 x10^-06^
leucylalanine (X14189) pos. mode	1022	-0.434	2.65 x10^-21^		254	-0.001	0.987	287	-1.250	1.29 x10^-38^	522	-1.012	1.80 x10^-37^	213	-0.466	3.35 x10^-07^
α-glutamyltyrosine (X14205)	934	-0.336	3.02 x10^-12^		138	-0.279	0.023	266	-1.337	1.26 x10^-29^	477	-1.105	2.51 x10^-27^	191	-0.771	1.43 x10^-10^
phenylalanylserine (X14208)	1025	-0.373	5.95 x10^-16^		179	-0.008	0.945	286	-1.507	2.03 x10^-41^	528	-1.269	7.91 x10^-44^	211	-0.901	1.18 x10^-15^
leucylalanine (X14304) neg. mode	916	-0.396	2.26 x10^-15^		169	0.040	0.723	269	-1.253	1.88 x10^-30^	484	-0.899	1.89 x10^-22^	163	-0.532	2.14 x10^-06^

Columns 2–8 give the results of the linear regression tests between metabolites (metabolite ratios, respectively) and the ACE-SNP rs4329. The linear regression tests were separately applied for ACE inhibitor users (users) and individuals who take no antihypertensives (non-users). Results with a p-value smaller than 1.4x10^-8^ (0.05/(517*(517/2) metabolite ratios + 517 metabolites)/25 SNPs) and with a p-gain greater than 517 (for ratios) were considered as significant.

Columns 9–17 show the results of the linear regression tests comparing the metabolite levels of each genotype group of the non-users (AA: major homozygotes, AG: heterozygotes, GG minor homozygotes) with those of all ACE inhibitor users. n gives the number of non-users in each genotype group.

Boxplots and results for all other analyzed ACE-SNPs can be found in *S1 Text* and *S3 Table*.

**Fig 1:**
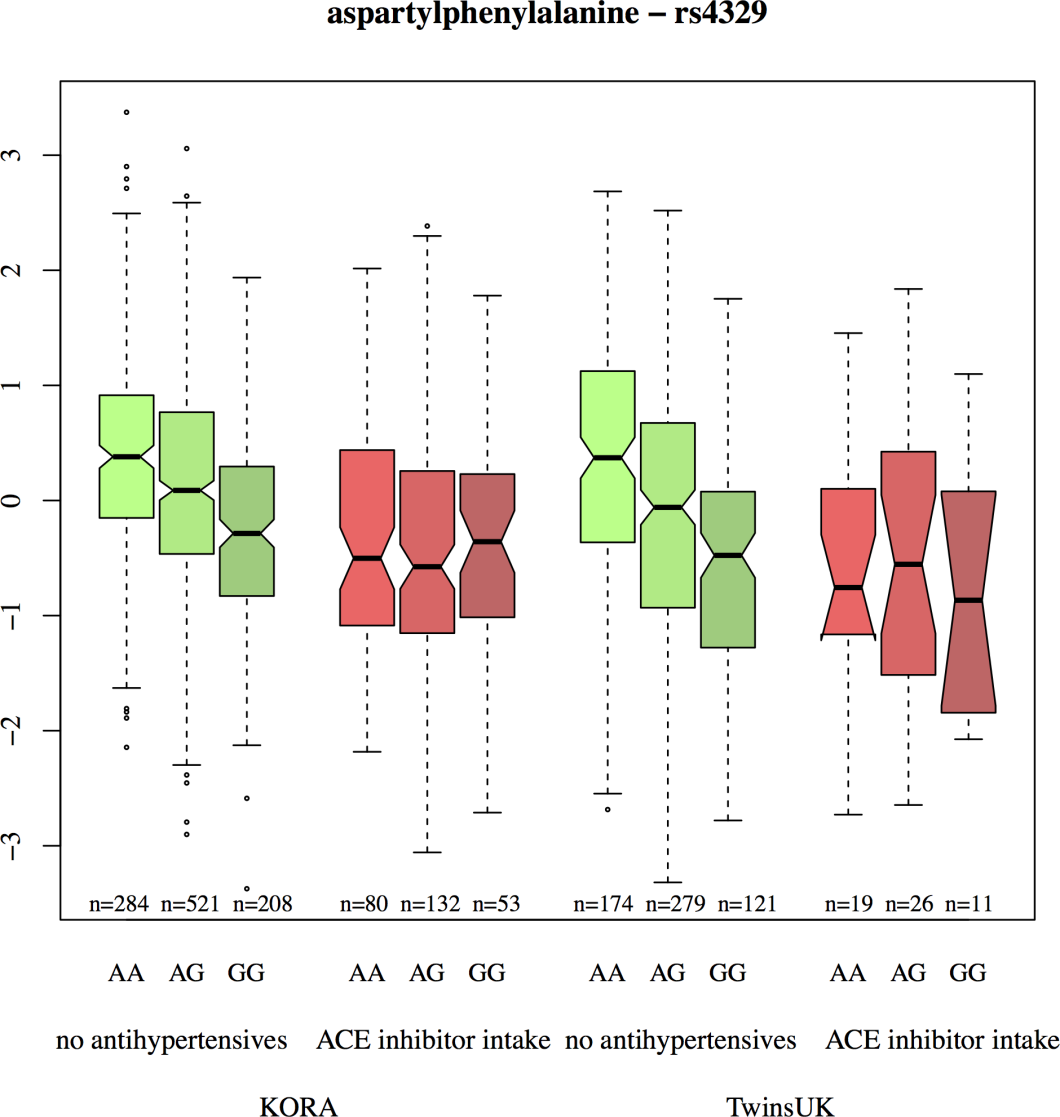
Inverse normalized values of aspartylphenylalanine for KORA and TwinsUK (AA: major homozygotes, AG: heterozygotes, GG minor homozygotes).

Significant associations with the ACE-SNPs could only be found in the analysis of the non-users. The dipeptides aspartylphenylalanine (Asp-Phe) (*Fig 1*), X14086 (possibly threonylglutamate), α-glutamyltyrosine (α-Glu-Tyr, formerly X14205), phenylalanylserine (Phe-Ser, formerly X14208) and leucylalanine (Leu-Ala, formerly X14304, negative mode (and X14189 positive mode)) were all negatively associated with the ACE-SNPs. Likewise, the metabolite ratios Asp-Phe/HWESASXX, Asp-Phe/X11805 and Asp-Phe/phenylalanylleucine (Phe-Leu, formerly X14450) showed negative associations with these SNPs. The oligopeptide HWESASXX is a part of the factor C3f which is degraded from C3 complement. The p-values of all significant results for the example rs4329 ranged from 6.04x10^-11^ to 9.16x10^-28^. P-gains from 1133.158 to 6.60x10^16^ indicate that these ratios carry more information than the two corresponding metabolites alone. Using additional cofactors, such as BMI, HDL cholesterol, LDL cholesterol, total cholesterol, triglycerides, diabetes and hypertension in the linear regression, or using the inversed ratios yielded the same significant associations.

### Differences in peptide levels between non-users and users depend on genotype

These metabolites and metabolite ratios with significant genetic associations in the non-users were analyzed in a second step to elucidate if genotype groups of the non-users differ significantly from all ACE inhibitor users. The results of this analysis (*Table 1*) showed that the metabolite concentrations of major homozygous (genotype AA) as well as heterozygous (genotype AG) non-users were significantly different from the metabolite concentrations of the users. Looking at the consistently negative beta estimates for every metabolite separately, a clear decreasing trend could be observed: the absolute beta value of the major homozygotes was always higher than the one of the heterozygotes which was in turn higher than the absolute beta value of the minor homozygotes. This trend was observed for all ACE-SNPs.

### Replication in TwinsUK

The metabolites and metabolite ratios showing significant associations with the ACE-SNPs in the KORA non-users were used for replication in TwinsUK (*Table 2; S2 Text*).

**Table 2:** Replication results from the TwinsUK study for rs4329.

metabolic trait	n (non-users)	beta (non-users)	p-value (non-users)	n (users)	beta (users)	p-value (users)	n AA	beta AA	p-value AA	n AG	beta AG	p-value AG	n GG	beta GG	p-value GG
aspartylphenylalanine	574	-0.436	3.80x10^-10^	56	-0.017	0.939	174	-0.764	0.0001	279	-0.464	0.005	121	-0.059	0.731
aspartylphenylalanine / HWESASXX	574	-0.233	2.15x10^-06^	56	-0.158	0.308	174	-0.441	0.001	279	-0.269	0.018	121	-0.090	0.439
X14086	486	-0.130	0.046	46	-0.321	0.209	143	-0.181	0.298	240	-0.076	0.648	103	0.075	0.680
leucylalanine (X14189) pos. mode	510	-0.308	8.58 x10^-07^	47	0.036	0.879	152	-0.792	2.19 x10^-05^	248	-0.756	6.03 x10^-06^	110	-0.218	0.184
α-glutamyltyrosine (X14205)	169	-0.180	0.113	4	0.242	0.877	61	-0.498	0.379	84	-0.341	0.491	24	0.205	0.653
phenylalanylserine (X14208)	408	-0.408	9.75 x10^-08^	15	0.059	0.892	131	-0.697	0.014	194	-0.415	0.121	83	0.232	0.439
leucylalanine (X14304) neg. mode	488	-0.302	3.58 x10^-06^	48	0.094	0.628	147	-1.017	1.76 x10^-07^	237	-0.961	4.62 x10^-08^	104	-0.408	0.015

For column description see Table 1; due to the family structure linear mixed-effects models were used instead of linear regressions; data for the ratios aspartylphenylalanine/X11805 and aspartylphenylalanine/phenylalanylleucin (X14450) was not reported in TwinsUK.

For three metabolites–Asp-Phe, Leu-Ala (X14304 and X14189) and Phe-Ser–and the ratio Asp-Phe/HWESASXX, the effect observed in KORA could be replicated. All five metabolite traits are significantly associated with the ACE-SNP rs4329 in non-users with p-values ranging from 2.15 x10^-06^ to 3.80x10^-10^, while in ACE inhibitor users no such association could be found.

Consistent with the KORA findings, also their concentrations in major homozygous (genotype AA) as well as in heterozygous (genotype AG) non-users were significantly different from the concentrations in the users–except for Phe-Ser, which showed only a significant association in the major homozygotes. The decreasing trend indicated by the beta estimates could not only be observed for the three metabolites with significant associations, but for all analyzed candidate metabolites.

### Significant association with blood pressure

To investigate whether the resulting metabolites are connected to blood pressure we applied a linear regression model with cofactors age and sex.

Asp-Phe shows a significant positive association with diastolic (p-value: 0.002) as well as systolic blood pressure (p-value: 0.009) in individuals not on antihypertensive treatment (non-users). Thus, with higher circulating levels of Asp-Phe a higher blood pressure is observed. Likewise, Phe-Ser is positively associated with diastolic (p-value: 0.001) as well as systolic (p-value: 0.005) blood pressure.

## Discussion

In this study we investigated genotype dependent differences in the metabolic response to ACE inhibitor intake. To this end, 517 serum metabolites of 1,361 participants from the population-based KORA F4 cohort were analyzed. For several di- and oligopeptides, we found significant influences of genetic variants in the ACE locus on the metabolites’ blood concentrations for individuals not treated with antihypertensives (non-users), whereas this effect was not observable in subjects medicated with ACE inhibitors (users). Moreover, significant effects on the levels of these metabolites through intake of ACE inhibitors can only be reported for individuals with major homozygous or heterozygous genotypes for ACE variants tagged by rs4329. Please note that this is a cross-sectional study which—in contrast to a longitudinal approach—observes no intra-individual changes. The results for the dipeptides aspartylphenylalanine (Asp-Phe), leucylalanine (Leu-Ala) and phenylalanylserine (Phe-Ser) as well as for the ratio Asp-Phe/HWESASXX could be replicated in the independent TwinsUK cohort proving the robustness of our results. In the following, we discuss specific hypotheses that can be generated from our findings and that might give useful hints for personalized treatment of hypertension. Analyzing several dipeptides as potential products of ACE at once provides a broader picture of the ACE activity than focusing only on the cleavage of angiotensin. Hence, we found hints for genotype dependent shifts in the activity of ACE regarding different substrates.

### Dipeptides as potential functional markers of ACE activity

Besides angiotensin I and II, ACE metabolizes a multitude of different substrates. Cholecystokinin-8 (CCK-8) is one substrate of ACE from which the dipeptide Asp-Phe-NH2 is cleaved [6]. Consistent with an inhibition of this process, in a former KORA study we observed lower levels of Asp-Phe in ACE inhibitior users [12]. This means, that the dipeptide Asp-Phe as a product of this cleavage process can be considered as a functional marker for ACE activity.

Our observation of a significant positive association between Asp-Phe and the diastolic blood pressure strengthens this suggested role of Asp-Phe as a functional marker for ACE activity.

When the data was stratified by ACE genotype (second analysis step), it turned out that only homozygotes of the rs4329 ACE major allele, and to a lesser extent also heterozygotes, displayed lower Asp-Phe concentrations in treated individuals when compared to non-treated ones. For the homozygotes of the minor allele, the Asp-Phe concentration did not show significant differences between ACE inhibitor users and non-users. Thus, Asp-Phe concentration, which was found to be connected to blood pressure, was not affected by the intake of ACE inhibitors in homozygotes of the minor allele. This suggests, that these patients might not fully profit from the effect of the ACE inhibitor.

Similar to Asp-Phe, four other dipeptides, namely Leu-Ala, α-Glu-Tyr, Phe-Ser and the unknown metabolite X14086 (probably threonylglutamate), showed genotype dependent relative differences in the metabolic concentration between individuals using ACE inhibitors and non-users. Though only for Phe-Ser a significant association with blood pressure could be observed in our KORA analysis, these four dipeptides might work as functional markers for genotype dependent ACE activity or shifts in its substrate specificity. For example, Phe-Ser is included in the amino acid sequence of bradykinin, a known substrate of ACE which plays a role in the decrease of blood pressure [4, 5].

### Ratios indicate possible shifts in ACE substrate specificity

The opioid Leu-enkephalin (Tyr-Gly-Gly-Phe-Leu) is known to be degraded by ACE by release of Phe-Leu [7, 8]. As many opioid peptides Leu-encephalin is suggested to be involved in the cardiac metabolism [23]. Since we observed a negative association with ACE inhibitor intake for this ratio (beta: -0.150, p-value: 2.75x10^-29^, p-gain: 2.68x10^13^), we hypothesize that in individuals using ACE inhibitors the Leu-enkephalin degradation releasing Phe-Leu is relatively increased compared to the cleavage of CCK-8 releasing Asp-Phe. We might further speculate whether ACE inhibitors rather interfere with the cleavage of CCK-8 than of Leu-enkephalin. A possible beneficial effect of CCK-8 was reported by *Ahren et al*. [24] who showed that CCK-8 exerts an antidiabetogenic action by stimulating insulin secretion.

When stratified by ACE genotype the ratio Asp-Phe/Phe-Leu also shows the effect that only homozygotes of the major allele, and to a lesser extent heterozygotes, display lower concentrations in ACE inhibitor users when compared to non-user. With this result, the question arises if these major homozygotes and heterozygotes might be more affected by possible side-effects (whether favorable or not) caused by a shift towards a possibly preferred cleavage of Leu-enkephalin.

Besides Asp-Phe/Phe-Leu, also the ratios Asp-Phe/HWESASXX and Asp-Phe/X11805 show the genotype dependent decreasing trend in non-users.

HWESASXX is a part of the factor C3f (NH2-Ser-Ser-Lys-Ile-Thr-His-Arg-Ile-**His-Trp-Glu-Ser-Ala-Ser-Leu-Leu**-Arg- COOH) [25], a degradation product of C3b which is itself liberated during the cleavage of the C3 complement [26, 27]. C3 is one of several proteins in the complement system that plays an important role in the human immune response. However, an over-activation of its proteins was found to be involved in the development of many different diseases [28]. Based on the analysis of C3-deficient mice *Zhou et al*. suggested that “C3 may be a primary factor to activate the renal RA [renin-angiotensin] systems to induce hypertension” [29], *Muscari et al*. identified in a population study C3 as a predictor of myocardial infarction in men [30], and *Engström et al*. found that C3 is associated with the risk of developing hypertension [31]. The fragment C3f was observed to induce spasms and to increase vascular permeability in the skin of guinea pigs [26]. A suggested connection between angiotensin and the complement system/inflammation is not fully understood so far [32].

In our study the genotype dependent differences in the ratio Asp-Phe/HWESASXX between ACE inhibitor users and non-users, implicate that for major homozygotes and (to a lesser extent) heterozygotes the relation of Asp-Phe and HWESASXX in the blood changes with the intake of ACE inhibitors. Thus, these groups might be more affected by advantages or disadvantages that may come with a shift towards a decreased cleavage of CCK-8 and a hypothesized increase in C3f degradation.

From the unknown X11805 in the ratio Asp-Phe/X11805 it is only known that it is an oligopeptide. One may speculate if it might be a product of ACE, but more insight in possible effects of a genotype dependent shift of this ratio has to be postponed until X11805 is identified.

### Limitations

Both study populations were composed of Caucasians of European ancestry, which means that our results cannot be generalized to other ethnicities. Since ACE activity was not directly measured and because of the cross-sectional design of this study, we can only generate hypotheses based on associations.

For our analysis we created the drug class “ACE inhibitor” to increase the statistical power. Thus, it is not possible to trace the observed effects on the metabolism back to single pharmaceutical agents. However, significant associations could be clearly observed using this drug class.

The dipeptide His-Leu cleaved from the most prominent ACE substrate angiotensin I was not among the significant associations. We may speculate if it is one of the unknown metabolites (starting with “X”) and does not show any significant changes due to further regulation mechanisms. On the other hand it might just not been detected by the used analytical method.

## Conclusion

**Fig 2:**
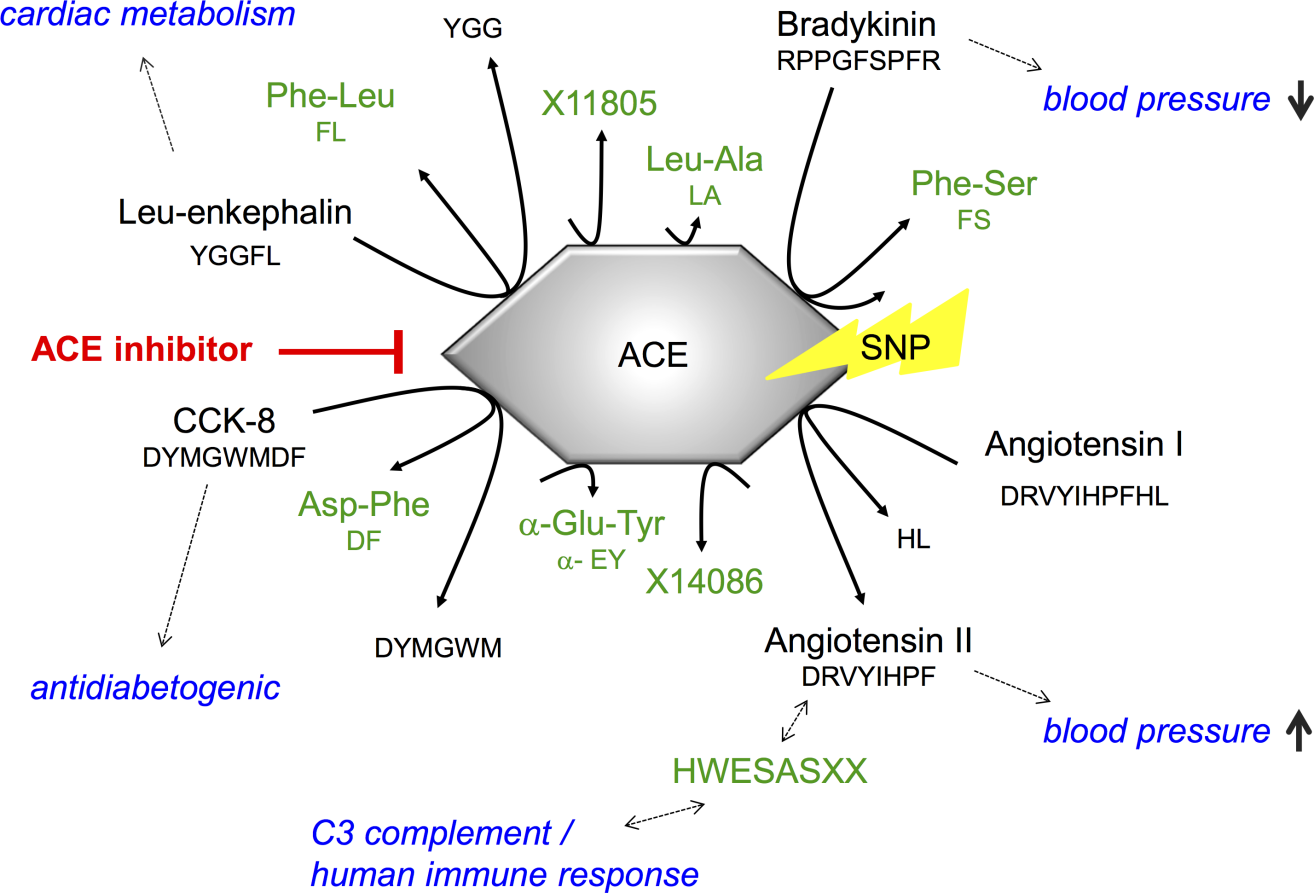
Summary of here discussed genotype dependent polypeptide cleavage catalyzed by ACE. Blue coloring: health effect of the respective polypeptide; green: measured metabolites.

In summary, we could identify several di- and oligopeptides that show genotype dependent differences in their concentration between ACE inhibitor users and non-users (*Fig 2*). Such changes in the concentration affected major homozygotes, and to a lesser extent heterozygotes, while minor homozygotes showed no or only small changes in the metabolite status. The significant associations of aspartylphenylalanine and phenylalanylserine with blood pressure showed that our results are connected to blood pressure. This means that these two dipeptides and perhaps also the other resulting metabolites might be markers for ACE activity. Thus, depending on the respective genotype, it might be worth considering an alternative drug for hypertension medication with respect to efficacy as well as side effects possibly induced by a shift in ACE substrate specificity.

## Supporting Information

S1 Fig Usage of the agents within the class “ACE inhibitors”. (PDF)

S1 Table Characteristics of the population related to the intake of ACE inhibitors. (PDF)

S2 Table Metabolites measured in KORA F4. (PDF)

S3 Table Results for all analyzed ACE SNPs in KORA. (XLS)

S1 Text Boxplots for all analyzed ACE-SNPs in KORA. (PDF)

S2 Text Boxplots for ACE-SNP rs4329 in TwinsUK. (PDF)
