# Supplementary material for: The Pharmacogenetic Footprint of ACE Inhibition: A Population-Based Metabolomics Study
Source: PLoS One. 2016 Apr 27;11(4):e0153163. doi: 10.1371/journal.pone.0153163 (PMC4847917; doi:10.1371/journal.pone.0153163)

The Pharmacogenetic Footprint of ACE Inhibition: a Population-Based Metabolomics Study

Authors:

Elisabeth Altmaier, Cristina Menni, Margit Heier, Christa Meisinger, Barbara Thorand, Jan Quell, Michael Kobl, Werner Römisch-Margl, Ana M Valdes, Massimo Mangino, Melanie Waldenberger, Konstantin Strauch, Thomas Illig, Jerzy Adamski, Tim Spector, Christian Gieger, Karsten Suhre, Gabi Kastenmüller

Journal: PLOS One

Corresponding author:

Gabi Kastenmüller
Helmholtz Zentrum München, German Research Center for Environmental Health,
Ingolstädter Landstr. 1,
D-85764 Neuherberg, Germany
e-mail: g.kastenmueller@helmholtz-muenchen.de

S1 Fig: Usage of the agents within the class "ACE inhibitors".

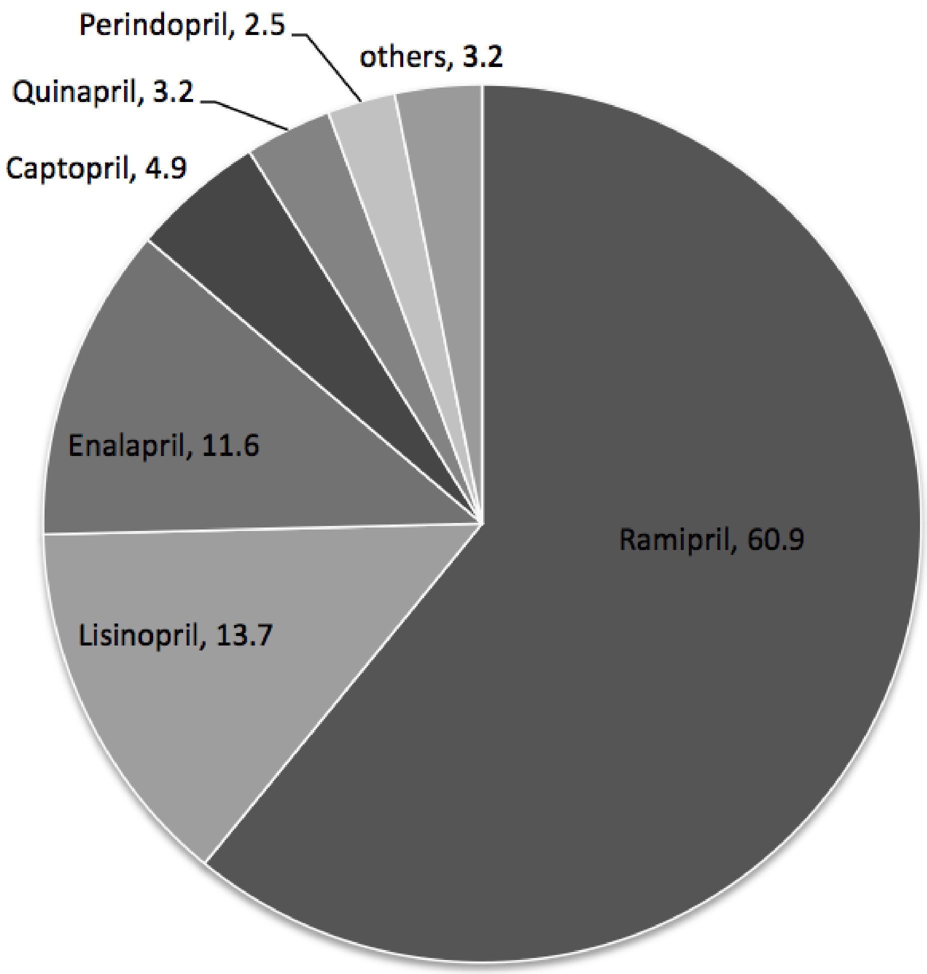

Supplement: S1 Fig — (PDF) [file pone.0153163.s001.pdf]
