# Supplementary material for: The Pharmacogenetic Footprint of ACE Inhibition: A Population-Based Metabolomics Study
Source: PLoS One. 2016 Apr 27;11(4):e0153163. doi: 10.1371/journal.pone.0153163 (PMC4847917; doi:10.1371/journal.pone.0153163)
Supplement: S1 Table — (PDF) [file pone.0153163.s002.pdf]

The Pharmacogenetic Footprint of ACE Inhibition: a Population-Based Metabolomics Study

Authors:

Elisabeth Altmaier, Cristina Menni, Margit Heier, Christa Meisinger, Barbara Thorand, Jan Quell, Michael Kobl, Werner Römisch-Margl, Ana M Valdes, Massimo Mangino, Melanie Waldenberger, Konstantin Strauch, Thomas Illig, Jerzy Adamski, Tim Spector, Christian Gieger, Karsten Suhre, Gabi Kastenmüller

Journal: PLOS One

Corresponding author:

Gabi Kastenmüller
Helmholtz Zentrum München, German Research Center for Environmental Health,
Ingolstädter Landstr. 1,
D-85764 Neuherberg, Germany
e-mail: g.kastenmueller@helmholtz-muenchen.de

S1 Table: Characteristics of the population related to the intake of ACE inhibitors

	Intake of ACE inhibitors Mean (SD) or n (%)	
	Yes (n=282)	No antihypertensives (n=1079)
**Age**	65.97 (7.7)	58.04 (8.2)
**Gender**		
Male	165 (12.1)	493 (36.2)
Female	117 (8.6)	586 (43.1)
**HDL-cholesterol (mmol/l)**	1.34 (0.3)	1.51 (0.3)
**LDL-cholesterol (mmol/l)**	3.42 (0.8)	3.71 (0.9)
**Total cholesterol (mmol/l)**	5.47 (0.9)	5.85 (1.0)
**Triglycerides (mmol/l)**	1.69 (1.0)	1.44 (1.1)
**BMI kg/m²**	30.55 (5.0)	26.94 (4.2)
**Diabetes mellitus**		
Yes	70 (5.1)	39 (2.9)
No	212 (15.6)	1039 (76.3)
**Hypertension**		
Yes	261 (19.2)	51 (3.7)
No	21 (1.5)	1026 (75.4)
**Intake of beta-blockers**		
Yes	139 (10.2)	0 (0)
No	143 (10.5)	1079 (79.3)
**Intake of diuretics**		
Yes	178 (13.1)	0 (0)
No	104 (7.6)	1079 (79.3)
