# Supplementary material for: The Pharmacogenetic Footprint of ACE Inhibition: A Population-Based Metabolomics Study
Source: PLoS One. 2016 Apr 27;11(4):e0153163. doi: 10.1371/journal.pone.0153163 (PMC4847917; doi:10.1371/journal.pone.0153163)
Supplement: S2 Table — (PDF) [file pone.0153163.s003.pdf]

The Pharmacogenetic Footprint of ACE Inhibition: a Population-Based Metabolomics Study

Authors:

Elisabeth Altmaier, Cristina Menni, Margit Heier, Christa Meisinger, Barbara Thorand, Jan Quell, Michael Kobl, Werner Römisch-Margl, Ana M Valdes, Massimo Mangino, Melanie Waldenberger, Konstantin Strauch, Thomas Illig, Jerzy Adamski, Tim Spector, Christian Gieger, Karsten Suhre, Gabi Kastenmüller

Journal: PLOS One

Corresponding author:

Gabi Kastenmüller
Helmholtz Zentrum München, German Research Center for Environmental Health,
Ingolstädter Landstr. 1,
D-85764 Neuherberg, Germany
e-mail: g.kastenmueller@helmholtz-muenchen.de

S2 Table: Metabolites measured in KORA F4.
For each metabolite we report the super-pathway, pathway and measurement platform. The biochemical identity of the metabolites is in general determined using adequate pure substances; in cases where metabolite identities were inferred based on their fragmentation spectrum and other biochemical evidence, these are indicated by a '*'.

metabolite	super-pathway	pathway	platform
alanine	Amino acid	Alanine and aspartate metabolism	GC/MS
asparagine	Amino acid	Alanine and aspartate metabolism	GC/MS
aspartate	Amino acid	Alanine and aspartate metabolism	GC/MS
N-acetylalanine	Amino acid	Alanine and aspartate metabolism	LC/MS neg
2-aminobutyrate	Amino acid	Butanoate metabolism	LC/MS pos
creatine	Amino acid	Creatine metabolism	LC/MS pos
creatinine	Amino acid	Creatine metabolism	LC/MS pos
2-hydroxybutyrate (AHB)	Amino acid	Cysteine, methionine, SAM, taurine metabolism	GC/MS
cysteine	Amino acid	Cysteine, methionine, SAM, taurine metabolism	GC/MS
cystine	Amino acid	Cysteine, methionine, SAM, taurine metabolism	GC/MS
methionine	Amino acid	Cysteine, methionine, SAM, taurine metabolism	LC/MS neg
glutamate	Amino acid	Glutamate metabolism	GC/MS
glutamine	Amino acid	Glutamate metabolism	LC/MS pos
pyroglutamine*	Amino acid	Glutamate metabolism	LC/MS pos
5-oxoproline	Amino acid	Glutathione metabolism	LC/MS pos
cysteine-glutathione disulfide	Amino acid	Glutathione metabolism	LC/MS pos
betaine	Amino acid	Glycine, serine and threonine metabolism	LC/MS pos
glycine	Amino acid	Glycine, serine and threonine metabolism	GC/MS
N-(2-furoyl)glycine	Amino acid	Glycine, serine and threonine metabolism	LC/MS pos
N-acetylglycine	Amino acid	Glycine, serine and threonine metabolism	GC/MS
N-acetylthreonine	Amino acid	Glycine, serine and threonine metabolism	LC/MS neg
serine	Amino acid	Glycine, serine and threonine metabolism	GC/MS
threonine	Amino acid	Glycine, serine and threonine metabolism	LC/MS pos

4-acetamidobutanoate	Amino acid	Guanidino and acetamido metabolism	LC/MS pos
3-methylhistidine	Amino acid	Histidine metabolism	LC/MS neg
histidine	Amino acid	Histidine metabolism	LC/MS neg
glutaroyl carnitine	Amino acid	Lysine metabolism	LC/MS pos
lysine	Amino acid	Lysine metabolism	LC/MS pos
pipecolate	Amino acid	Lysine metabolism	LC/MS pos
3-(3-hydroxyphenyl)propionate	Amino acid	Phenylalanine & tyrosine metabolism	LC/MS neg
3-(4-hydroxyphenyl)lactate	Amino acid	Phenylalanine & tyrosine metabolism	LC/MS neg
3-methoxytyrosine	Amino acid	Phenylalanine & tyrosine metabolism	LC/MS pos
3-phenylpropionate (hydrocinnamate)	Amino acid	Phenylalanine & tyrosine metabolism	LC/MS neg
4-hydroxyphenylacetate	Amino acid	Phenylalanine & tyrosine metabolism	GC/MS
p-cresol sulfate	Amino acid	Phenylalanine & tyrosine metabolism	LC/MS neg
phenol sulfate	Amino acid	Phenylalanine & tyrosine metabolism	LC/MS neg
phenylacetate	Amino acid	Phenylalanine & tyrosine metabolism	LC/MS neg
phenylacetylglutamine	Amino acid	Phenylalanine & tyrosine metabolism	LC/MS pos
phenylalanine	Amino acid	Phenylalanine & tyrosine metabolism	LC/MS pos
phenyllactate (PLA)	Amino acid	Phenylalanine & tyrosine metabolism	LC/MS neg
tyrosine	Amino acid	Phenylalanine & tyrosine metabolism	LC/MS pos
3-indoxyl sulfate	Amino acid	Tryptophan metabolism	LC/MS neg
C-glycosyltryptophan*	Amino acid	Tryptophan metabolism	LC/MS pos
indoleacetate	Amino acid	Tryptophan metabolism	LC/MS pos
indolelactate	Amino acid	Tryptophan metabolism	LC/MS pos
indolepropionate	Amino acid	Tryptophan metabolism	LC/MS pos
kynurenine	Amino acid	Tryptophan metabolism	LC/MS pos
serotonin (5HT)	Amino acid	Tryptophan metabolism	LC/MS pos

tryptophan	Amino acid	Tryptophan metabolism	LC/MS pos
arginine	Amino acid	Urea cycle; arginine-, proline-, metabolism	LC/MS neg
citrulline	Amino acid	Urea cycle; arginine-, proline-, metabolism	LC/MS pos
dimethylarginine (SDMA + ADMA)	Amino acid	Urea cycle; arginine-, proline-, metabolism	LC/MS pos
homocitrulline	Amino acid	Urea cycle; arginine-, proline-, metabolism	LC/MS pos
homostachydrine*	Amino acid	Urea cycle; arginine-, proline-, metabolism	LC/MS pos
N-acetylornithine	Amino acid	Urea cycle; arginine-, proline-, metabolism	LC/MS pos
ornithine	Amino acid	Urea cycle; arginine-, proline-, metabolism	LC/MS pos
proline	Amino acid	Urea cycle; arginine-, proline-, metabolism	LC/MS pos
stachydrine	Amino acid	Urea cycle; arginine-, proline-, metabolism	LC/MS pos
trans-4-hydroxyproline	Amino acid	Urea cycle; arginine-, proline-, metabolism	GC/MS
urea	Amino acid	Urea cycle; arginine-, proline-, metabolism	GC/MS
2-hydroxyisobutyrate	Amino acid	Valine, leucine and isoleucine metabolism	GC/MS
2-methylbutyroylcarnitine	Amino acid	Valine, leucine and isoleucine metabolism	LC/MS pos
3-hydroxy-2-ethylpropionate	Amino acid	Valine, leucine and isoleucine metabolism	GC/MS
3-methyl-2-oxobutyrate	Amino acid	Valine, leucine and isoleucine metabolism	LC/MS neg
3-methyl-2-oxovalerate	Amino acid	Valine, leucine and isoleucine metabolism	LC/MS neg
4-methyl-2-oxopentanoate	Amino acid	Valine, leucine and isoleucine metabolism	LC/MS neg
alpha-hydroxyisovalerate	Amino acid	Valine, leucine and isoleucine metabolism	LC/MS neg
beta-hydroxyisovalerate	Amino acid	Valine, leucine and isoleucine metabolism	LC/MS neg
hydroxyisovaleroyl carnitine	Amino acid	Valine, leucine and isoleucine metabolism	LC/MS pos
isobutyrylcarnitine	Amino acid	Valine, leucine and isoleucine metabolism	LC/MS pos
isoleucine	Amino acid	Valine, leucine and isoleucine metabolism	LC/MS pos
isovalerylcarnitine	Amino acid	Valine, leucine and isoleucine metabolism	LC/MS pos
leucine	Amino acid	Valine, leucine and isoleucine metabolism	LC/MS pos

levulinate (4-oxovalerate)	Amino acid	Valine, leucine and isoleucine metabolism	LC/MS pos or neg
tiglyl carnitine	Amino acid	Valine, leucine and isoleucine metabolism	LC/MS pos
valine	Amino acid	Valine, leucine and isoleucine metabolism	LC/MS pos
erythronate*	Carbohydrate	Aminosugars metabolism	GC/MS
erythrose	Carbohydrate	Fructose, mannose, galactose, starch, and sucrose metabolism	GC/MS
fructose	Carbohydrate	Fructose, mannose, galactose, starch, and sucrose metabolism	GC/MS
mannitol	Carbohydrate	Fructose, mannose, galactose, starch, and sucrose metabolism	GC/MS
mannose	Carbohydrate	Fructose, mannose, galactose, starch, and sucrose metabolism	GC/MS
1,5-anhydroglucitol (1,5-AG)	Carbohydrate	Glycolysis, gluconeogenesis, pyruvate metabolism	LC/MS neg
1,6-anhydroglucose	Carbohydrate	Glycolysis, gluconeogenesis, pyruvate metabolism	GC/MS
glucose	Carbohydrate	Glycolysis, gluconeogenesis, pyruvate metabolism	GC/MS
glycerate	Carbohydrate	Glycolysis, gluconeogenesis, pyruvate metabolism	GC/MS
lactate	Carbohydrate	Glycolysis, gluconeogenesis, pyruvate metabolism	GC/MS
pyruvate	Carbohydrate	Glycolysis, gluconeogenesis, pyruvate metabolism	GC/MS
arabinose	Carbohydrate	Nucleotide sugars, pentose metabolism	GC/MS
arabitol	Carbohydrate	Nucleotide sugars, pentose metabolism	GC/MS
threitol	Carbohydrate	Nucleotide sugars, pentose metabolism	GC/MS
ascorbate (Vitamin C)	Cofactors and vitamins	Ascorbate and aldarate metabolism	GC/MS
threonate	Cofactors and vitamins	Ascorbate and aldarate metabolism	GC/MS
heme*	Cofactors and vitamins	Hemoglobin and porphyrin	LC/MS pos

bilirubin (E,E)*	Cofactors and vitamins	Hemoglobin and porphyrin metabolism	LC/MS pos
bilirubin (E,Z or Z,E)*	Cofactors and vitamins	Hemoglobin and porphyrin metabolism	LC/MS pos
bilirubin (Z,Z)	Cofactors and vitamins	Hemoglobin and porphyrin metabolism	LC/MS neg
biliverdin	Cofactors and vitamins	Hemoglobin and porphyrin metabolism	LC/MS neg
trigonelline (N'-methylnicotinate)	Cofactors and vitamins	Nicotinate and nicotinamide metabolism	LC/MS pos
pantothenate	Cofactors and vitamins	Pantothenate and CoA metabolism	LC/MS pos
riboflavin (Vitamin B2)	Cofactors and vitamins	Riboflavin metabolism	LC/MS pos
alpha-tocopherol	Cofactors and vitamins	Tocopherol metabolism	GC/MS
gamma-tocopherol	Cofactors and vitamins	Tocopherol metabolism	GC/MS
pyridoxate	Cofactors and vitamins	Vitamin B6 metabolism	LC/MS neg
alpha-ketoglutarate	Energy	Krebs cycle	GC/MS
citrate	Energy	Krebs cycle	GC/MS
malate	Energy	Krebs cycle	GC/MS
succinylcarnitine	Energy	Krebs cycle	LC/MS pos
acetylphosphate	Energy	Oxidative phosphorylation	GC/MS
phosphate	Energy	Oxidative phosphorylation	LC/MS neg
cholate	Lipid	Bile acid metabolism	LC/MS neg
deoxycholate	Lipid	Bile acid metabolism	LC/MS neg
glycochenodeoxycholate	Lipid	Bile acid metabolism	LC/MS neg
glycocholate	Lipid	Bile acid metabolism	LC/MS pos
glycodeoxycholate	Lipid	Bile acid metabolism	LC/MS neg
hyodeoxycholate	Lipid	Bile acid metabolism	LC/MS neg
taurochenodeoxycholate	Lipid	Bile acid metabolism	LC/MS neg
taurocholate	Lipid	Bile acid metabolism	LC/MS neg
taurodeoxycholate	Lipid	Bile acid metabolism	LC/MS neg

taurolithocholate 3-sulfate	Lipid	Bile acid metabolism	LC/MS neg
ursodeoxycholate	Lipid	Bile acid metabolism	LC/MS neg
2-tetradecenoyl carnitine	Lipid	Carnitine metabolism	LC/MS pos
3-dehydrocarnitine*	Lipid	Carnitine metabolism	LC/MS pos
acetylcarnitine	Lipid	Carnitine metabolism	LC/MS pos
carnitine	Lipid	Carnitine metabolism	LC/MS pos
decanoylcarnitine	Lipid	Carnitine metabolism	LC/MS pos
hexanoylcarnitine	Lipid	Carnitine metabolism	LC/MS pos
laurylcarnitine	Lipid	Carnitine metabolism	LC/MS pos
octanoylcarnitine	Lipid	Carnitine metabolism	LC/MS pos
oleoylcarnitine	Lipid	Carnitine metabolism	LC/MS pos
palmitoylcarnitine	Lipid	Carnitine metabolism	LC/MS pos
stearoylcarnitine	Lipid	Carnitine metabolism	LC/MS pos
thromboxane B2	Lipid	Eicosanoid	LC/MS neg
dihomo-linolenate (20:3n3 or n6)	Lipid	Essential fatty acid	LC/MS neg
docosahexaenoate (DHA; 22:6n3)	Lipid	Essential fatty acid	LC/MS neg
docosapentaenoate (n3 DPA; 22:5n3)	Lipid	Essential fatty acid	LC/MS neg
eicosapentaenoate (EPA; 20:5n3)	Lipid	Essential fatty acid	LC/MS neg
linolenate [alpha or gamma; (18:3n3 or 6)]	Lipid	Essential fatty acid	LC/MS neg
isovalerate	Lipid	Fatty acid metabolism	LC/MS neg
butyrylcarnitine	Lipid	Fatty acid metabolism (also BCAA metabolism)	LC/MS pos
propionylcarnitine	Lipid	Fatty acid metabolism (also BCAA metabolism)	LC/MS pos
linoleamide (18:2n6)	Lipid	Fatty acid, amide	LC/MS pos
oleamide	Lipid	Fatty acid, amide	LC/MS pos
3-carboxy-4-methyl-5-propyl-2-furanpropanoate (CMPF)	Lipid	Fatty acid, dicarboxylate	LC/MS neg

dodecanedioate	Lipid	Fatty acid, dicarboxylate	LC/MS neg
hexadecanedioate	Lipid	Fatty acid, dicarboxylate	LC/MS neg
octadecanedioate	Lipid	Fatty acid, dicarboxylate	LC/MS neg
sebacate (decanedioate)	Lipid	Fatty acid, dicarboxylate	LC/MS neg
tetradecanedioate	Lipid	Fatty acid, dicarboxylate	LC/MS neg
n-Butyl Oleate	Lipid	Fatty acid, ester	GC/MS
2-hydroxypalmitate	Lipid	Fatty acid, monohydroxy	LC/MS neg
2-hydroxystearate	Lipid	Fatty acid, monohydroxy	LC/MS neg
choline	Lipid	Glycerolipid metabolism	LC/MS pos
glycerol	Lipid	Glycerolipid metabolism	GC/MS
glycerol 3-phosphate (G3P)	Lipid	Glycerolipid metabolism	GC/MS
glycerophosphorylcholine (GPC)	Lipid	Glycerolipid metabolism	LC/MS pos
inositol 1-phosphate (I1P)	Lipid	Inositol metabolism	GC/MS
myo-inositol	Lipid	Inositol metabolism	GC/MS
scyllo-inositol	Lipid	Inositol metabolism	GC/MS
3-hydroxybutyrate (BHBA)	Lipid	Ketone bodies	GC/MS
10-heptadecenoate (17:1n7)	Lipid	Long chain fatty acid	LC/MS neg
10-nonadecenoate (19:1n9)	Lipid	Long chain fatty acid	LC/MS neg
adrenate (22:4n6)	Lipid	Long chain fatty acid	LC/MS neg
arachidonate (20:4n6)	Lipid	Long chain fatty acid	LC/MS neg
dihomo-linoleate (20:2n6)	Lipid	Long chain fatty acid	LC/MS neg
eicosenoate (20:1n9 or 11)	Lipid	Long chain fatty acid	LC/MS neg
linoleate (18:2n6)	Lipid	Long chain fatty acid	LC/MS neg
margarate (17:0)	Lipid	Long chain fatty acid	LC/MS neg
myristate (14:0)	Lipid	Long chain fatty acid	LC/MS neg

myristoleate (14:1n5)	Lipid	Long chain fatty acid	LC/MS neg
nonadecanoate (19:0)	Lipid	Long chain fatty acid	LC/MS neg
oleate (18:1n9)	Lipid	Long chain fatty acid	LC/MS neg
palmitate (16:0)	Lipid	Long chain fatty acid	LC/MS neg
palmitoleate (16:1n7)	Lipid	Long chain fatty acid	LC/MS neg
pentadecanoate (15:0)	Lipid	Long chain fatty acid	GC/MS
stearate (18:0)	Lipid	Long chain fatty acid	LC/MS neg
stearidonate (18:4n3)	Lipid	Long chain fatty acid	LC/MS neg
1-arachidonoylglycerophosphocholine*	Lipid	Lysolipid	LC/MS pos
1-arachidonoylglycerophosphoethanolamine*	Lipid	Lysolipid	LC/MS neg
1-arachidonoylglycerophosphoinositol*	Lipid	Lysolipid	LC/MS neg
1-docosahexaenoylglycerophosphocholine*	Lipid	Lysolipid	LC/MS pos
1-eicosadienoylglycerophosphocholine*	Lipid	Lysolipid	LC/MS pos
1-eicosatrienoylglycerophosphocholine*	Lipid	Lysolipid	LC/MS pos
1-heptadecanoylglycerophosphocholine	Lipid	Lysolipid	LC/MS pos
1-linoleoylglycerophosphocholine	Lipid	Lysolipid	LC/MS pos
1-linoleoylglycerophosphoethanolamine*	Lipid	Lysolipid	LC/MS neg
1-myristoylglycerophosphocholine	Lipid	Lysolipid	LC/MS pos
1-oleoylglycerophosphocholine	Lipid	Lysolipid	LC/MS pos
1-oleoylglycerophosphoethanolamine	Lipid	Lysolipid	LC/MS neg
1-palmitoleoylglycerophosphocholine*	Lipid	Lysolipid	LC/MS pos
1-palmitoylglycerophosphocholine	Lipid	Lysolipid	LC/MS pos
1-palmitoylglycerophosphoethanolamine	Lipid	Lysolipid	LC/MS neg
1-palmitoylglycerophosphoinositol*	Lipid	Lysolipid	LC/MS neg
1-stearoylglycerophosphocholine	Lipid	Lysolipid	LC/MS pos

1-stearoylglycerophosphoethanolamine	Lipid	Lysolipid	LC/MS neg
1-stearoylglycerophosphoinositol	Lipid	Lysolipid	LC/MS neg
2-linoleoylglycerophosphocholine*	Lipid	Lysolipid	LC/MS pos
2-linoleoylglycerophosphoethanolamine*	Lipid	Lysolipid	LC/MS neg
2-oleoylglycerophosphocholine*	Lipid	Lysolipid	LC/MS pos
2-palmitoylglycerophosphocholine*	Lipid	Lysolipid	LC/MS pos
2-stearoylglycerophosphocholine*	Lipid	Lysolipid	LC/MS pos
10-undecenoate (11:1n1)	Lipid	Medium chain fatty acid	LC/MS neg
5-dodecenoate (12:1n7)	Lipid	Medium chain fatty acid	LC/MS neg
caprate (10:0)	Lipid	Medium chain fatty acid	LC/MS neg
caproate (6:0)	Lipid	Medium chain fatty acid	LC/MS neg
caprylate (8:0)	Lipid	Medium chain fatty acid	LC/MS neg
heptanoate (7:0)	Lipid	Medium chain fatty acid	LC/MS neg
laurate (12:0)	Lipid	Medium chain fatty acid	LC/MS neg
pelargonate (9:0)	Lipid	Medium chain fatty acid	LC/MS neg
undecanoate (11:0)	Lipid	Medium chain fatty acid	LC/MS neg
1-linoleoylglycerol (1-monolinolein)	Lipid	Monoacylglycerol	LC/MS neg
1-oleoylglycerol (1-monoolein)	Lipid	Monoacylglycerol	LC/MS pos
1-palmitoylglycerol (1-monopalmitin)	Lipid	Monoacylglycerol	GC/MS
1-stearoylglycerol (1-monostearin)	Lipid	Monoacylglycerol	GC/MS
valerate	Lipid	Short chain fatty acid	LC/MS neg
7-alpha-hydroxy-3-oxo-4-cholestenoate (7-Hoca)	Lipid	Sterol/Steroid	LC/MS neg
androsterone sulfate	Lipid	Sterol/Steroid	LC/MS neg
cholesterol	Lipid	Sterol/Steroid	GC/MS
cortisol	Lipid	Sterol/Steroid	LC/MS pos

cortisone	Lipid	Sterol/Steroid	LC/MS pos
dehydroisoandrosterone sulfate (DHEA-S)	Lipid	Sterol/Steroid	LC/MS neg
epiandrosterone sulfate	Lipid	Sterol/Steroid	LC/MS neg
estrone 3-sulfate	Lipid	Sterol/Steroid	LC/MS neg
lathosterol	Lipid	Sterol/Steroid	GC/MS
carbamazepine*			LC/MS pos
hypoxanthine	Nucleotide	Purine metabolism, (hypo)xanthine/inosine containing	LC/MS neg
inosine	Nucleotide	Purine metabolism, (hypo)xanthine/inosine containing	LC/MS neg
xanthine	Nucleotide	Purine metabolism, (hypo)xanthine/inosine containing	LC/MS pos
adenosine	Nucleotide	Purine metabolism, adenine containing	LC/MS pos
N1-methyladenosine	Nucleotide	Purine metabolism, adenine containing	LC/MS pos
7-methylguanine	Nucleotide	Purine metabolism, guanine containing	LC/MS pos
guanosine	Nucleotide	Purine metabolism, guanine containing	LC/MS pos
N2,N2-dimethylguanosine	Nucleotide	Purine metabolism, guanine containing	LC/MS pos
allantoin	Nucleotide	Purine metabolism, urate metabolism	GC/MS
urate	Nucleotide	Purine metabolism, urate metabolism	LC/MS neg
pseudouridine	Nucleotide	Pyrimidine metabolism, uracil containing	LC/MS pos
uridine	Nucleotide	Pyrimidine metabolism, uracil containing	LC/MS neg
aspartylphenylalanine	Peptide	Dipeptide	LC/MS pos
glycylvaline	Peptide	Dipeptide	LC/MS pos
leucylleucine	Peptide	Dipeptide	LC/MS pos
phenylalanylphenylalanine	Peptide	Dipeptide	LC/MS pos
pro-hydroxy-pro	Peptide	Dipeptide	LC/MS pos

pyroglutamylglycine	Peptide	Dipeptide	LC/MS neg
ADpSGEGDFXAEGGGVR*	Peptide	Fibrinogen cleavage peptide	LC/MS pos
ADSGEGDFXAEGGGVR*	Peptide	Fibrinogen cleavage peptide	LC/MS pos
DSGEGDFXAEGGGVR*	Peptide	Fibrinogen cleavage peptide	LC/MS pos
gamma-glutamylglutamate	Peptide	gamma-glutamyl	LC/MS pos
gamma-glutamylglutamine	Peptide	gamma-glutamyl	LC/MS pos
gamma-glutamylisoleucine*	Peptide	gamma-glutamyl	LC/MS pos
gamma-glutamylleucine	Peptide	gamma-glutamyl	LC/MS pos
gamma-glutamylmethionine*	Peptide	gamma-glutamyl	LC/MS pos
gamma-glutamylphenylalanine	Peptide	gamma-glutamyl	LC/MS pos
gamma-glutamylthreonine*	Peptide	gamma-glutamyl	LC/MS pos
gamma-glutamyltyrosine	Peptide	gamma-glutamyl	LC/MS pos
gamma-glutamylvaline	Peptide	gamma-glutamyl	LC/MS pos
bradykinin, des-arg(9)	Peptide	Polypeptide	LC/MS pos
HWESASXX*	Peptide	Polypeptide	LC/MS pos
2-hydroxyhippurate (salicylurate)	Xenobiotics	Benzoate metabolism	LC/MS neg
3-ethylphenylsulfate*	Xenobiotics	Benzoate metabolism	LC/MS neg
4-ethylphenylsulfate	Xenobiotics	Benzoate metabolism	LC/MS neg
4-vinylphenol sulfate	Xenobiotics	Benzoate metabolism	LC/MS neg
benzoate	Xenobiotics	Benzoate metabolism	LC/MS neg
catechol sulfate	Xenobiotics	Benzoate metabolism	LC/MS neg
hippurate	Xenobiotics	Benzoate metabolism	LC/MS pos
glycerol 2-phosphate	Xenobiotics	Chemical	GC/MS
2-hydroxyacetaminophen sulfate*	Xenobiotics	Drug	LC/MS neg
2-methoxyacetaminophen sulfate*	Xenobiotics	Drug	LC/MS neg

3-(cystein-S-yl)acetaminophen*	Xenobiotics	Drug	LC/MS pos
4-acetamidophenol	Xenobiotics	Drug	GC/MS
4-acetaminophen sulfate	Xenobiotics	Drug	LC/MS neg
hydroquinone sulfate	Xenobiotics	Drug	LC/MS neg
hydroxypioglitazone*	Xenobiotics	Drug	LC/MS pos
ibuprofen	Xenobiotics	Drug	LC/MS neg
metoprolol	Xenobiotics	Drug	LC/MS pos
metoprolol acid metabolite*	Xenobiotics	Drug	LC/MS pos
naproxen	Xenobiotics	Drug	LC/MS neg
p-acetamidophenylglucuronide	Xenobiotics	Drug	LC/MS pos
pioglitazone*	Xenobiotics	Drug	LC/MS pos
salicylate	Xenobiotics	Drug	GC/MS
salicyluric glucuronide*	Xenobiotics	Drug	LC/MS neg
piperine	Xenobiotics	Food component/Plant	LC/MS pos
quinate	Xenobiotics	Food component/Plant	GC/MS
saccharin	Xenobiotics	Food component/Plant	LC/MS neg
thymol sulfate	Xenobiotics	Food component/Plant	LC/MS neg
erythritol	Xenobiotics	Sugar, sugar substitute, starch	GC/MS
cotinine	Xenobiotics	Tobacco metabolite	LC/MS pos
1,3,7-trimethylurate	Xenobiotics	Xanthine metabolism	LC/MS neg
1,7-dimethylurate	Xenobiotics	Xanthine metabolism	LC/MS neg
1-methylurate	Xenobiotics	Xanthine metabolism	LC/MS pos
1-methylxanthine	Xenobiotics	Xanthine metabolism	LC/MS pos
3-methylxanthine	Xenobiotics	Xanthine metabolism	LC/MS pos
7-methylxanthine	Xenobiotics	Xanthine metabolism	LC/MS pos

caffeine	Xenobiotics	Xanthine metabolism	LC/MS pos
paraxanthine	Xenobiotics	Xanthine metabolism	LC/MS pos
theobromine	Xenobiotics	Xanthine metabolism	LC/MS pos
theophylline	Xenobiotics	Xanthine metabolism	LC/MS neg
X- 01911			LC/MS pos
X- 02249			LC/MS neg
X- 02269			LC/MS neg
X- 02973			GC/MS
X- 03003			GC/MS
X- 03056			LC/MS pos
X- 03088			GC/MS
X- 03090			GC/MS
X- 03094			GC/MS
X- 04357			GC/MS
X- 04494			GC/MS
X- 04495			GC/MS
X- 04498			GC/MS
X- 04499			GC/MS
X- 04500			GC/MS
X- 04515			GC/MS
X- 04621			GC/MS
X- 05426			GC/MS
X- 05491			GC/MS
X- 05907			GC/MS
X- 06126			LC/MS neg

X- 06226	GC/MS
X- 06227	GC/MS
X- 06246	GC/MS
X- 06267	GC/MS
X- 06307	GC/MS
X- 06350	GC/MS
X- 06351	GC/MS
X- 07765	LC/MS neg
X- 08402	GC/MS
X- 08766	GC/MS
X- 08988	GC/MS
X- 09026	GC/MS
X- 09108	GC/MS
X- 09706	GC/MS
X- 09789	LC/MS neg
X- 10346	LC/MS neg
X- 10395	GC/MS
X- 10419	GC/MS
X- 10429	GC/MS
X- 10500	GC/MS
X- 10506	GC/MS
X- 10510	GC/MS
X- 10675	GC/MS
X- 10810	GC/MS
X- 11204	LC/MS pos

X- 11244	1	LC/MS neg
X- 11247		LC/MS neg
X- 11255		LC/MS pos
X- 11261		LC/MS pos
X- 11299		LC/MS neg
X- 11315		LC/MS pos
X- 11317		LC/MS neg
X- 11319		LC/MS neg
X- 11327		LC/MS pos
X- 11334		LC/MS pos
X- 11374		LC/MS pos
X- 11381		LC/MS pos
X- 11412		LC/MS pos
X- 11421		LC/MS pos
X- 11422		LC/MS neg
X- 11423		LC/MS neg
X- 11437		LC/MS neg
X- 11438		LC/MS neg
X- 11440		LC/MS neg
X- 11441		LC/MS neg
X- 11442		LC/MS neg
X- 11443		LC/MS neg
X- 11444		LC/MS neg
X- 11445		LC/MS neg
X- 11450		LC/MS neg

X- 11452	LC/MS neg
X- 11469	LC/MS pos
X- 11470	LC/MS neg
X- 11478	LC/MS neg
X- 11483	LC/MS neg
X- 11485	LC/MS neg
X- 11491	LC/MS neg
X- 11497	LC/MS neg
X- 11521	LC/MS pos
X- 11529	LC/MS neg
X- 11530	LC/MS neg
X- 11537	LC/MS pos
X- 11538	LC/MS neg
X- 11540	LC/MS pos
X- 11546	LC/MS neg
X- 11550	LC/MS neg
X- 11552	LC/MS pos
X- 11568	LC/MS pos
X- 11593	LC/MS neg
X- 11786	LC/MS pos
X- 11787	LC/MS pos
X- 11792	LC/MS pos
X- 11793	LC/MS pos
X- 11795	LC/MS pos
X- 11799	LC/MS pos

X- 11805	LC/MS pos
X- 11809	LC/MS pos
X- 11818	LC/MS pos
X- 11820	LC/MS pos
X- 11826	LC/MS neg
X- 11843	LC/MS neg
X- 11845	LC/MS neg
X- 11847	LC/MS neg
X- 11849	LC/MS neg
X- 11850	LC/MS neg
X- 11852	LC/MS neg
X- 11858	LC/MS neg
X- 11859	LC/MS neg
X- 11876	LC/MS neg
X- 11880	LC/MS neg
X- 11905	LC/MS neg
X- 12007	LC/MS neg
X- 12013	LC/MS neg
X- 12029	LC/MS neg
X- 12038	LC/MS neg
X- 12039	LC/MS neg
X- 12040	LC/MS neg
X- 12056	LC/MS pos
X- 12063	LC/MS neg
X- 12092	LC/MS pos

X- 12093	LC/MS pos
X- 12094	LC/MS pos
X- 12095	LC/MS pos
X- 12100	LC/MS pos
X- 12116	LC/MS pos
X- 12188	LC/MS neg
X- 12189	LC/MS neg
X- 12206	LC/MS neg
X- 12212	LC/MS neg
X- 12216	LC/MS neg
X- 12217	LC/MS neg
X- 12230	LC/MS neg
X- 12231	LC/MS neg
X- 12236	LC/MS neg
X- 12244	LC/MS pos
X- 12253	LC/MS neg
X- 12261	LC/MS neg
X- 12263	LC/MS neg
X- 12329	LC/MS neg
X- 12405	LC/MS neg
X- 12407	LC/MS neg
X- 12428	LC/MS neg
X- 12435	LC/MS neg
X- 12441	LC/MS neg
X- 12442	LC/MS neg

X- 12443	LC/MS neg
X- 12450	LC/MS neg
X- 12456	LC/MS neg
X- 12465	LC/MS pos
X- 12510	LC/MS pos
X- 12524	LC/MS neg
X- 12544	LC/MS pos
X- 12556	GC/MS
X- 12627	LC/MS neg
X- 12644	LC/MS neg
X- 12645	LC/MS neg
X- 12680	LC/MS pos
X- 12696	LC/MS neg
X- 12704	LC/MS neg
X- 12711	LC/MS neg
X- 12712	LC/MS neg
X- 12717	LC/MS neg
X- 12719	LC/MS neg
X- 12726	LC/MS neg
X- 12728	LC/MS neg
X- 12729	LC/MS neg
X- 12734	LC/MS neg
X- 12740	LC/MS neg
X- 12749	LC/MS pos
X- 12771	GC/MS

X- 12776	LC/MS neg
X- 12786	GC/MS
X- 12798	LC/MS pos
X- 12816	LC/MS neg
X- 12830	LC/MS neg
X- 12833	LC/MS neg
X- 12844	LC/MS neg
X- 12847	LC/MS neg
X- 12850	LC/MS neg
X- 12851	LC/MS neg
X- 12855	LC/MS pos
X- 12990	LC/MS neg
X- 13069	LC/MS neg
X- 13183	LC/MS pos
X- 13215	LC/MS neg
X- 13372	LC/MS neg
X- 13429	LC/MS neg
X- 13431	LC/MS pos
X- 13435	LC/MS pos
X- 13477	LC/MS pos
X- 13496	GC/MS
X- 13548	LC/MS pos
X- 13549	LC/MS pos
X- 13553	LC/MS neg
X- 13619	GC/MS

X- 13640			LC/MS neg
X- 13658			LC/MS neg
X- 13671			LC/MS neg
X- 13699			LC/MS pos
X- 13741			LC/MS neg
X- 13859			LC/MS neg
X- 14056			LC/MS pos
X- 14057			LC/MS pos
X- 14086			LC/MS pos
X- 14189 (leucylalanine)	Protein	Dipeptide	LC/MS pos
X- 14205 (α-glutamyltyrosine)	Protein	Dipeptide	LC/MS pos
X- 14208 (phenylalanylserine)	Protein	Dipeptide	LC/MS pos
X- 14304 (leucylalanine)	Protein	Dipeptide	LC/MS neg
X- 14374			LC/MS pos
X- 14450 (phenylalanylleucin)	Protein	Dipeptide	LC/MS pos
X- 14473			LC/MS pos
X- 14478			LC/MS pos
X- 14486			LC/MS pos
X- 14541			LC/MS neg
X- 14588			LC/MS neg
X- 14625			LC/MS neg
X- 14626			LC/MS neg
X- 14632			LC/MS neg
X- 14658			LC/MS neg
X- 14662			LC/MS neg

X- 14663	LC/MS neg
X- 14745	GC/MS
X- 14977	LC/MS pos
