# Supplementary material for: The Pharmacogenetic Footprint of ACE Inhibition: A Population-Based Metabolomics Study
Source: PLoS One. 2016 Apr 27;11(4):e0153163. doi: 10.1371/journal.pone.0153163 (PMC4847917; doi:10.1371/journal.pone.0153163)
Supplement: S1 Text — (PDF) [file pone.0153163.s005.pdf]

The pharmacogenetic footprint of ACE inhibition: a population based metabolomics study

Authors:

Elisabeth Altmaier, Cristina Menni, Margit Heier, Christa Meisinger, Barbara Thorand, Jan Quell, Michael Kobl, Werner Römisch-Margl, Ana M Valdes, Massimo Mangino, Melanie Waldenberger, Konstantin Strauch, Thomas Illig, Jerzy Adamski, Tim D. Spector, Christian Gieger, Karsten Suhre, Gabi Kastenmüller

Journal: PLOS One

Corresponding author:

Gabi Kastenmüller
Helmholtz Zentrum München, German Research Center for Environmental Health,
Ingolstädter Landstr. 1,
D-85764 Neuherberg, Germany
e-mail: g.kastenmueller@helmholtz-muenchen.de

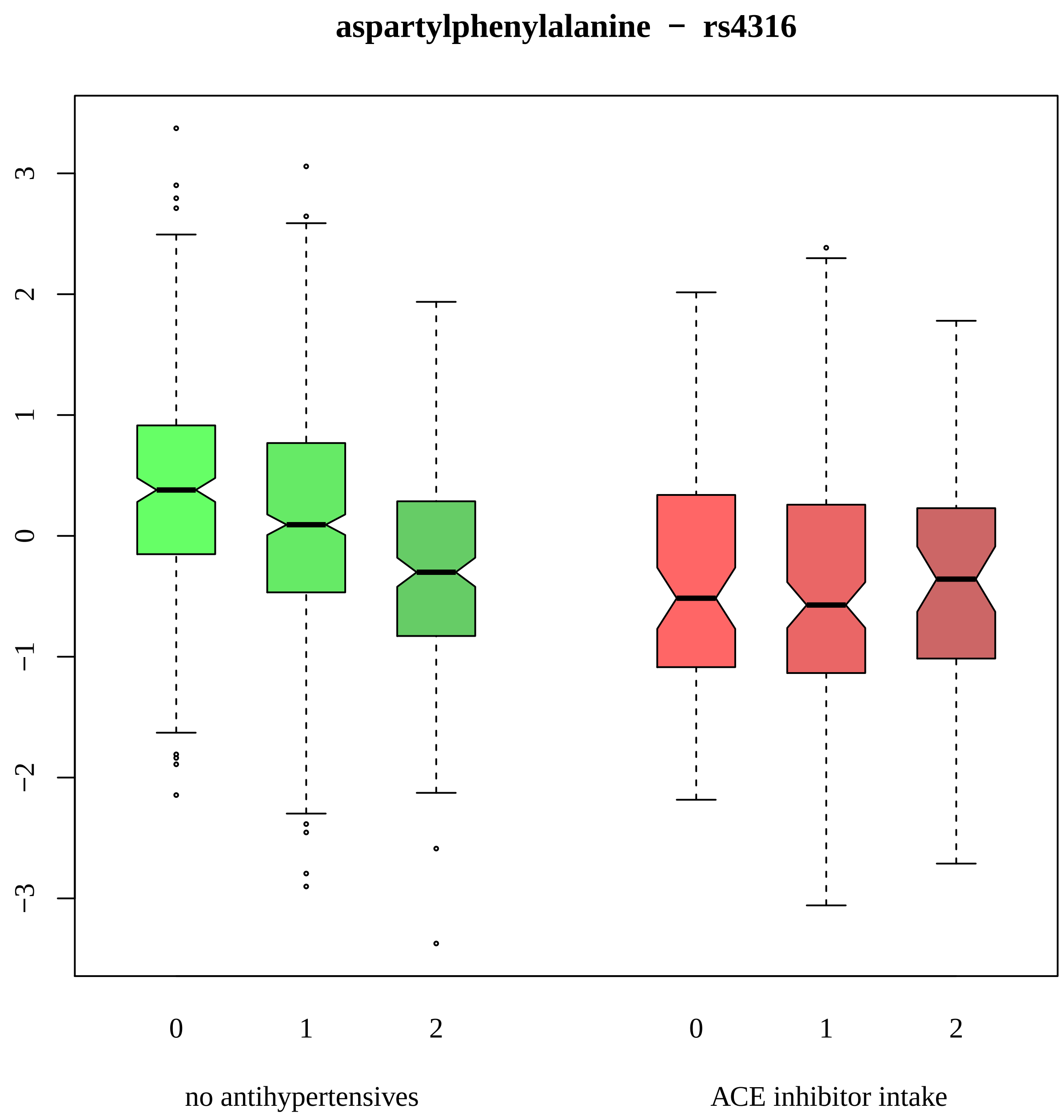
aspartylphenylalanine – rs4316
3
2
1
0
−1
−2
−3
0
1
2
0
1
2
no antihypertensives
ACE inhibitor intake

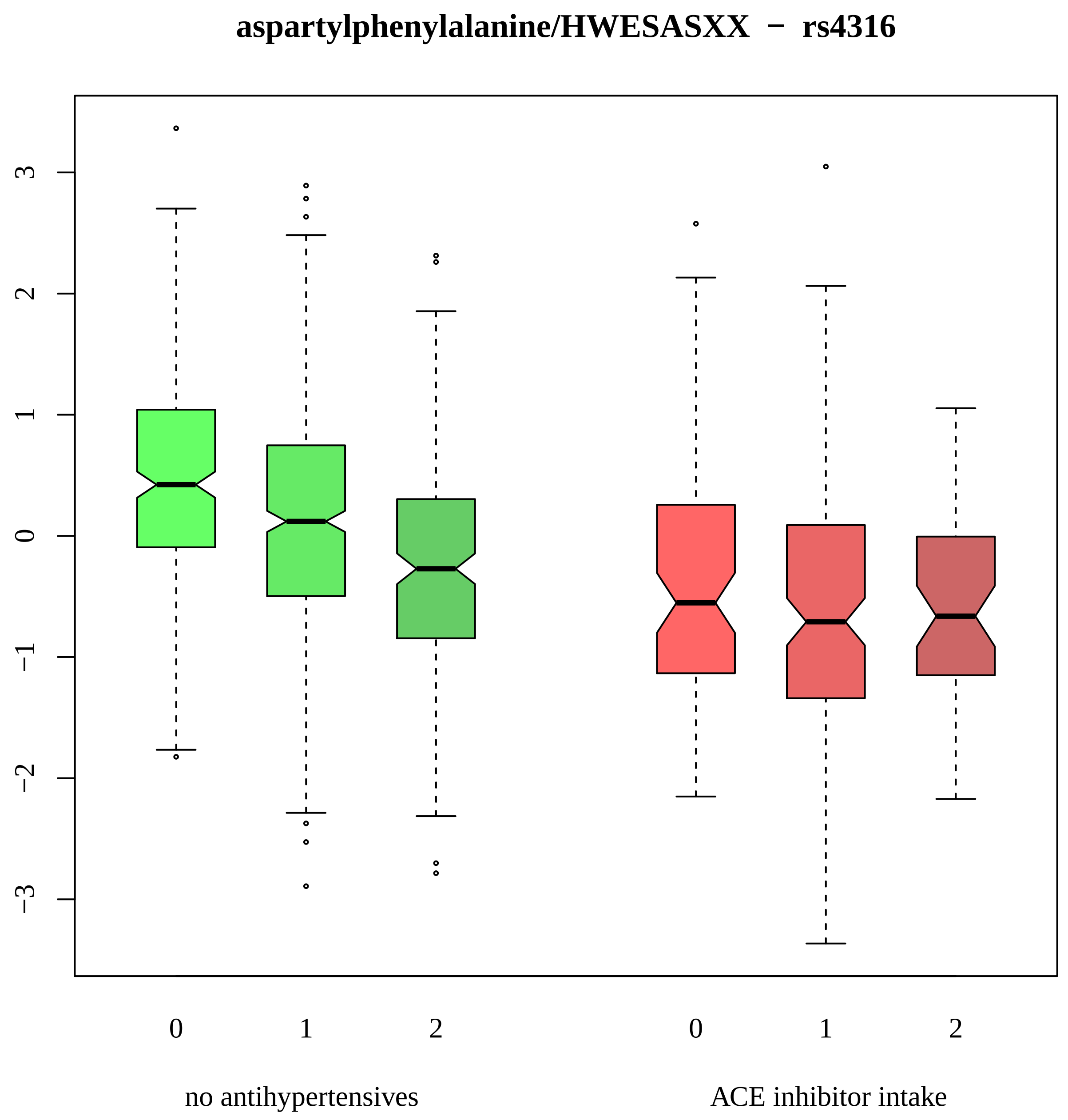

aspartylphenylalanine/HWESASXX − rs4316
3
2
1
0
−1
−2
−3
0
1
2
0
1
2
no antihypertensives
ACE inhibitor intake

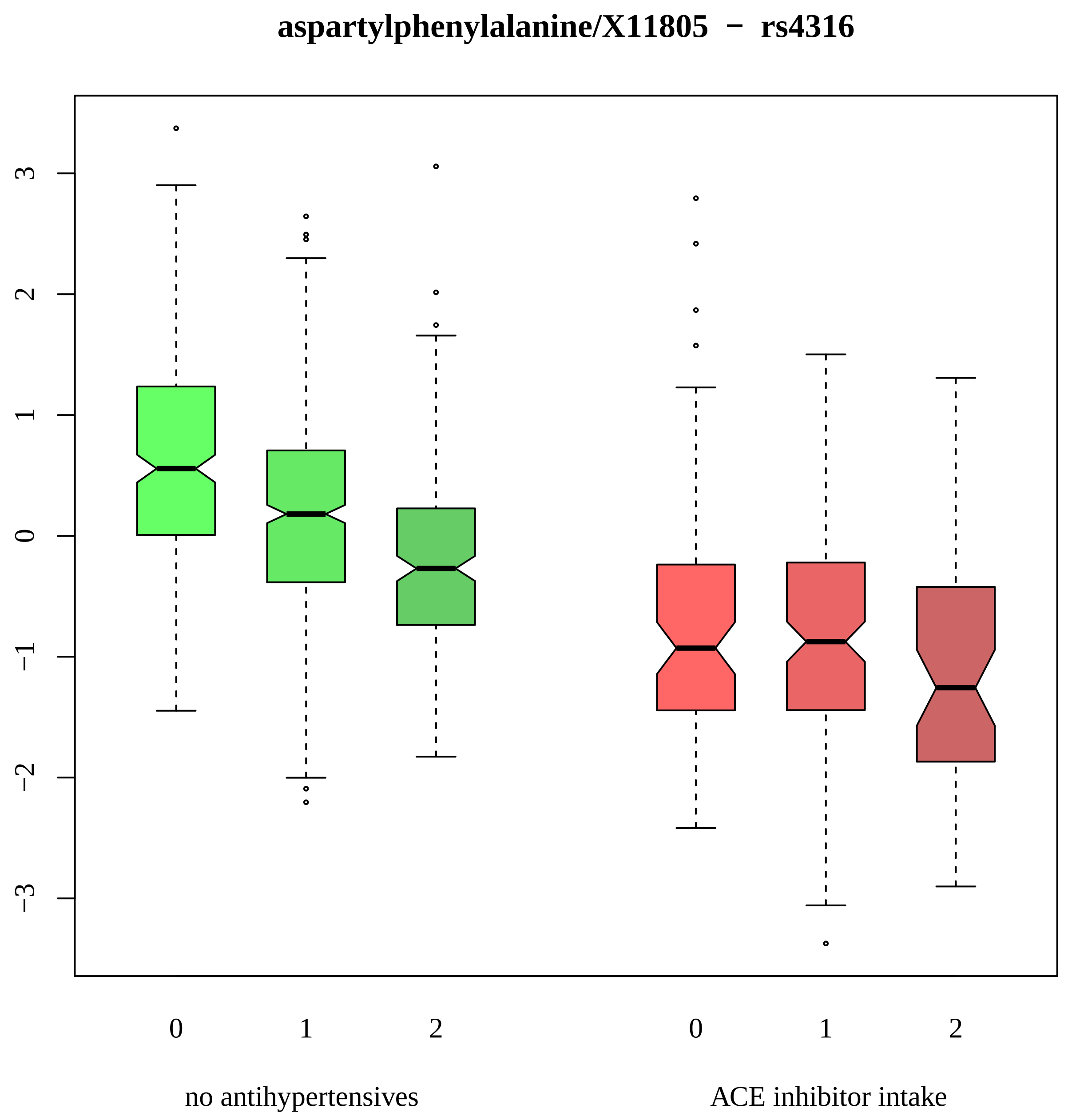

aspartylphenylalanine/X11805 – rs4316
3
2
1
0
−1
−2
−3
0
1
2
0
1
2
no antihypertensives
ACE inhibitor intake

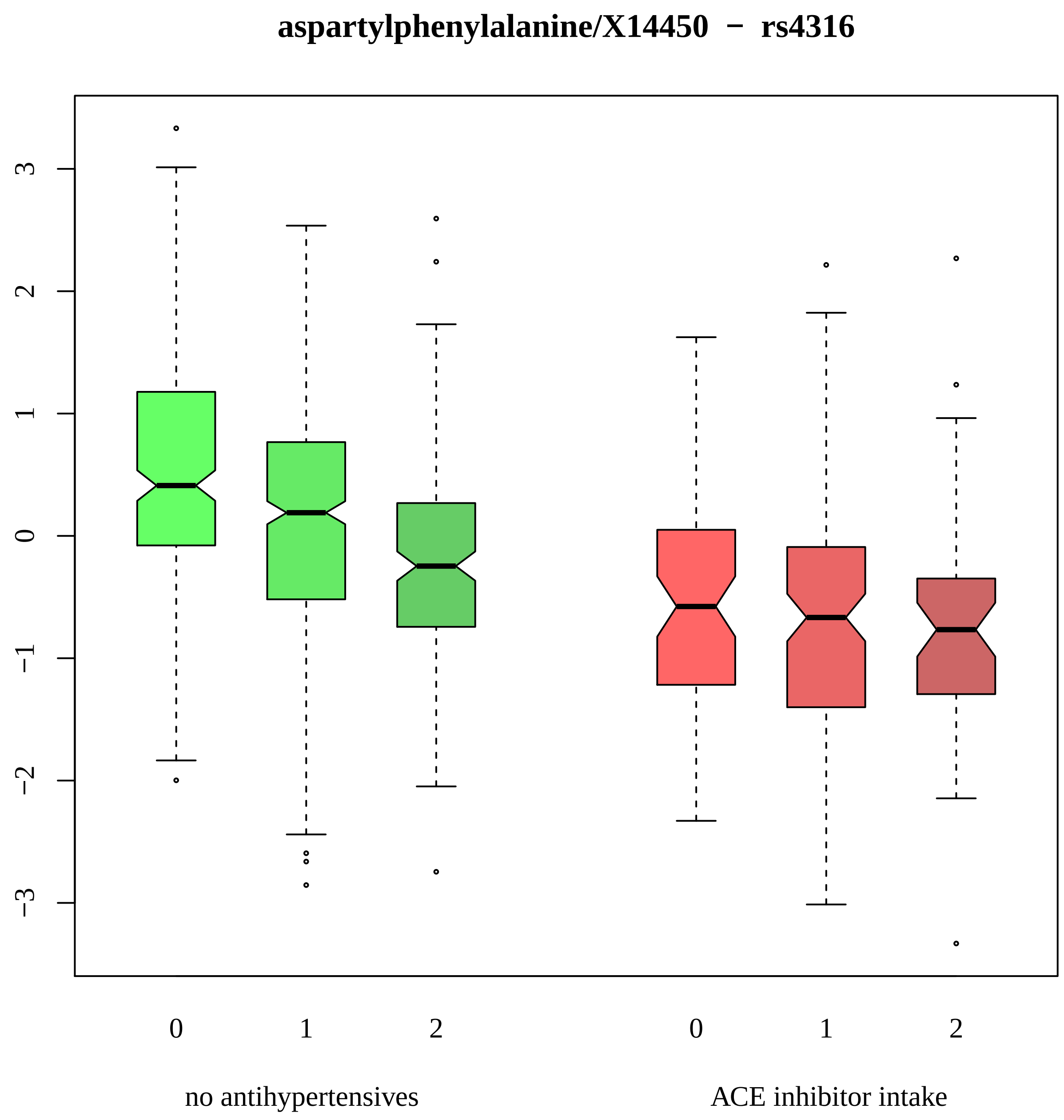
aspartylphenylalanine/X14450 – rs4316
3
2
1
0
−1
−2
−3
0
1
2
0
1
2
no antihypertensives
ACE inhibitor intake

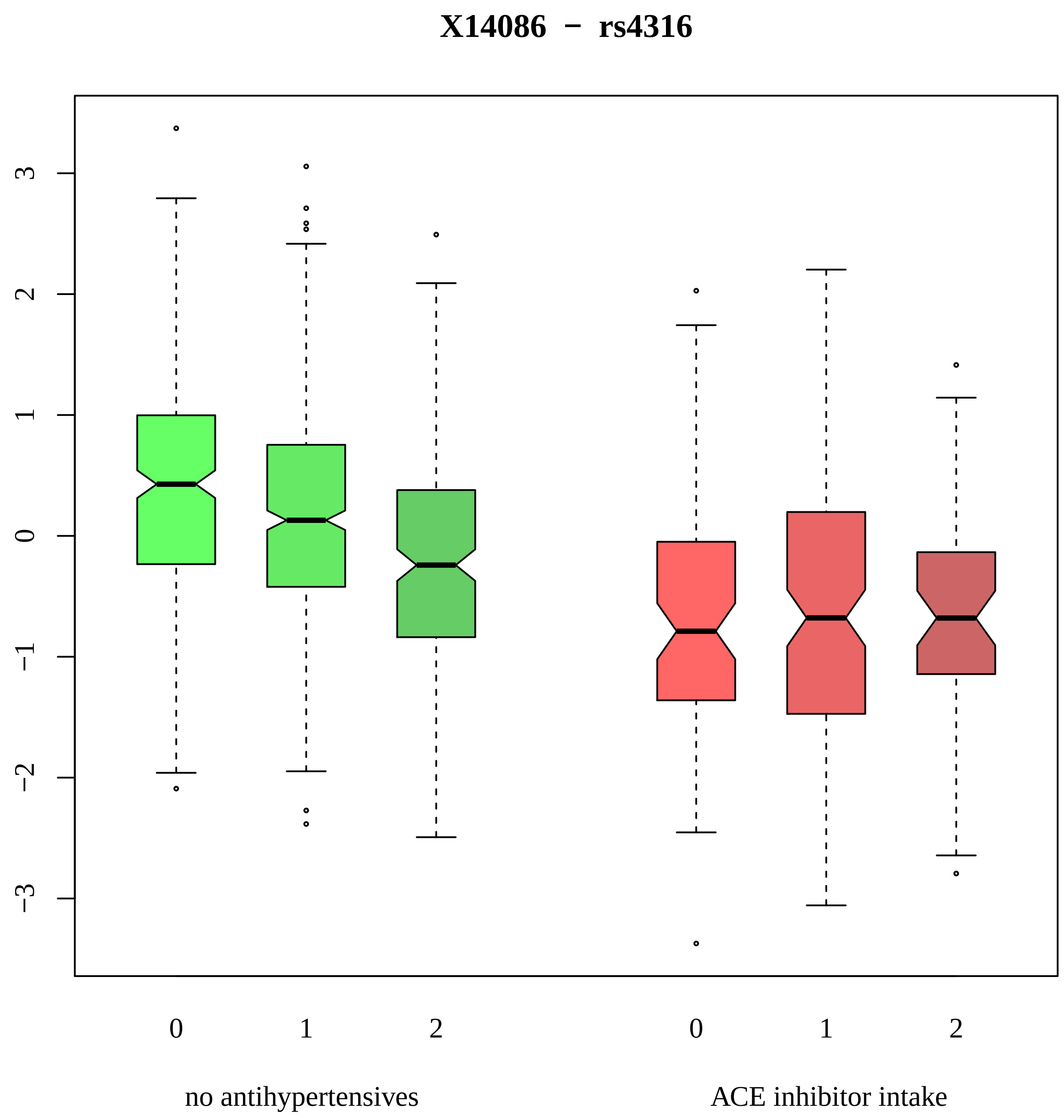

X14086 – rs4316
3
2
1
0
−1
−2
−3
0
1
2
0
1
2
no antihypertensives
ACE inhibitor intake

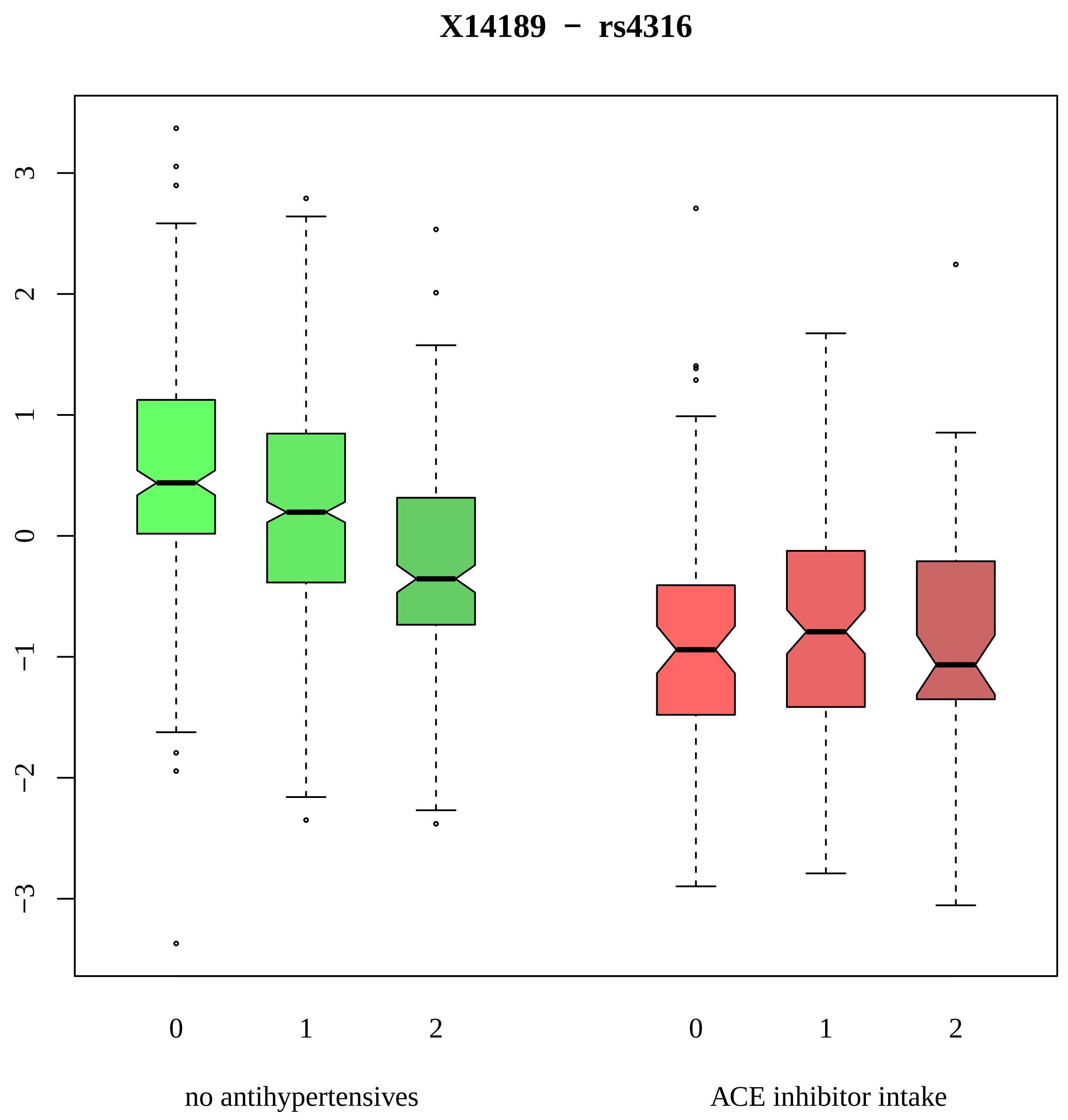

X14189 – rs4316
3
2
1
0
−1
−2
−3
0
1
2
0
1
2
no antihypertensives
ACE inhibitor intake

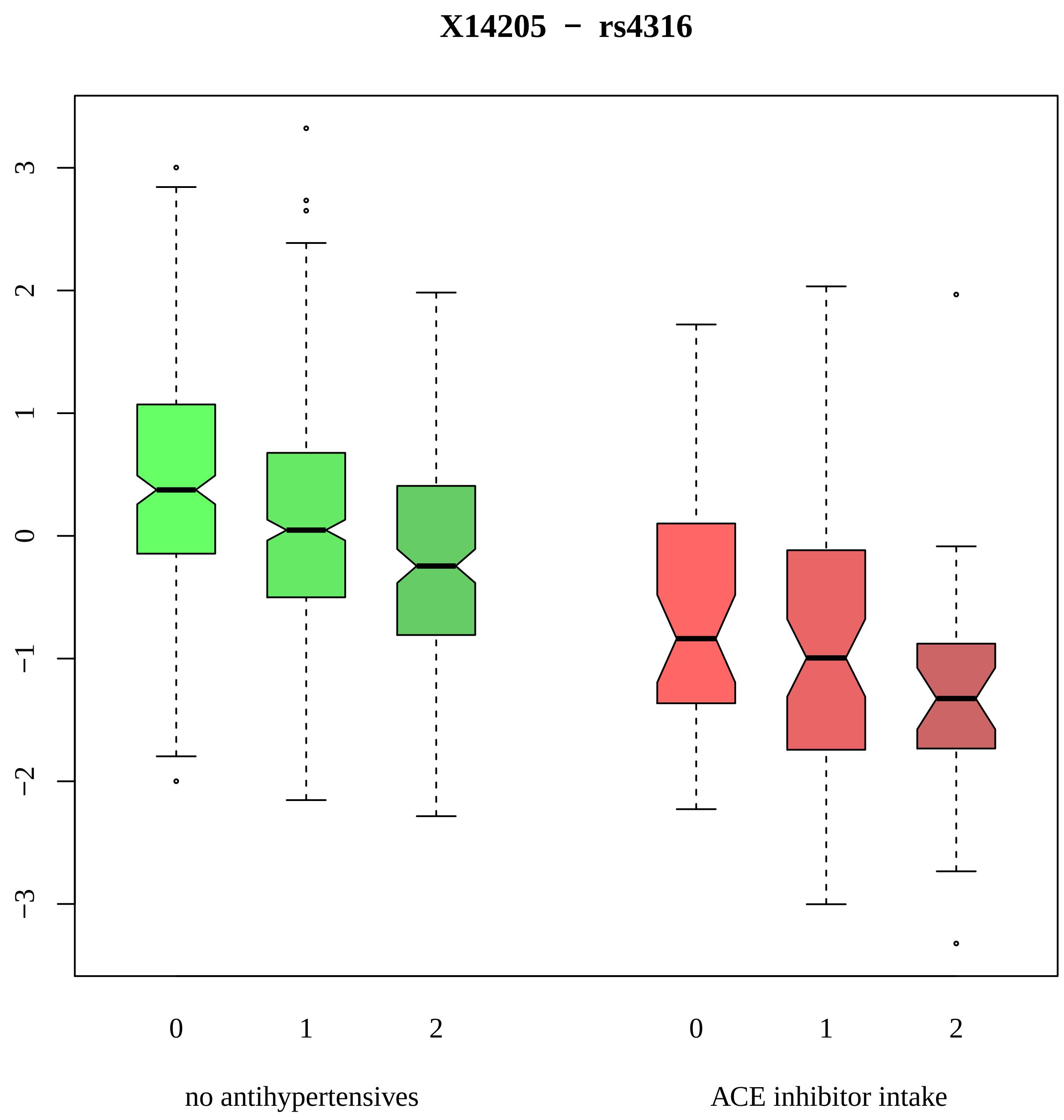

X14205 – rs4316
3
2
1
0
−1
−2
−3
0
1
2
0
1
2
no antihypertensives
ACE inhibitor intake

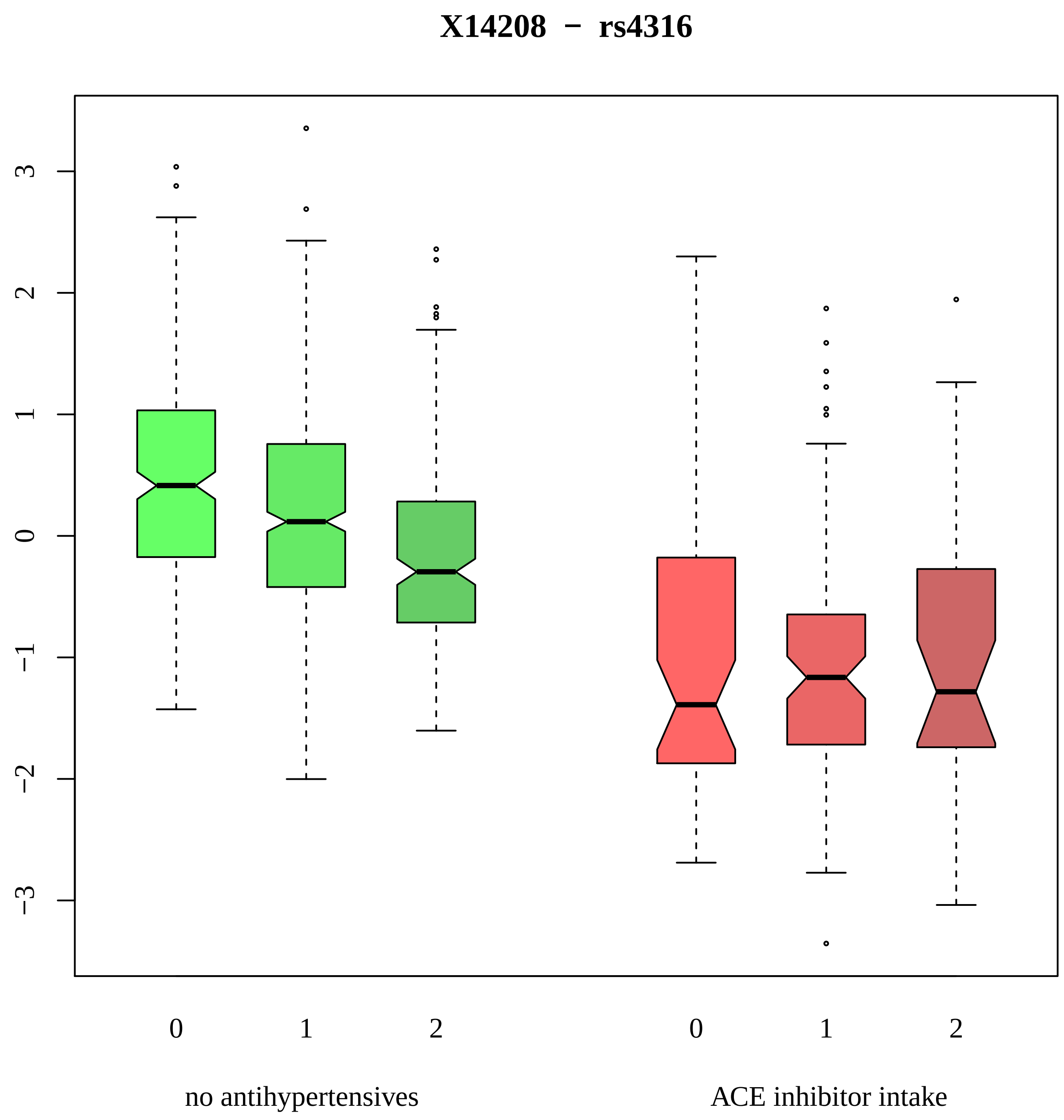

X14208 – rs4316
3
2
1
0
−1
−2
−3
0
1
2
0
1
2
no antihypertensives
ACE inhibitor intake

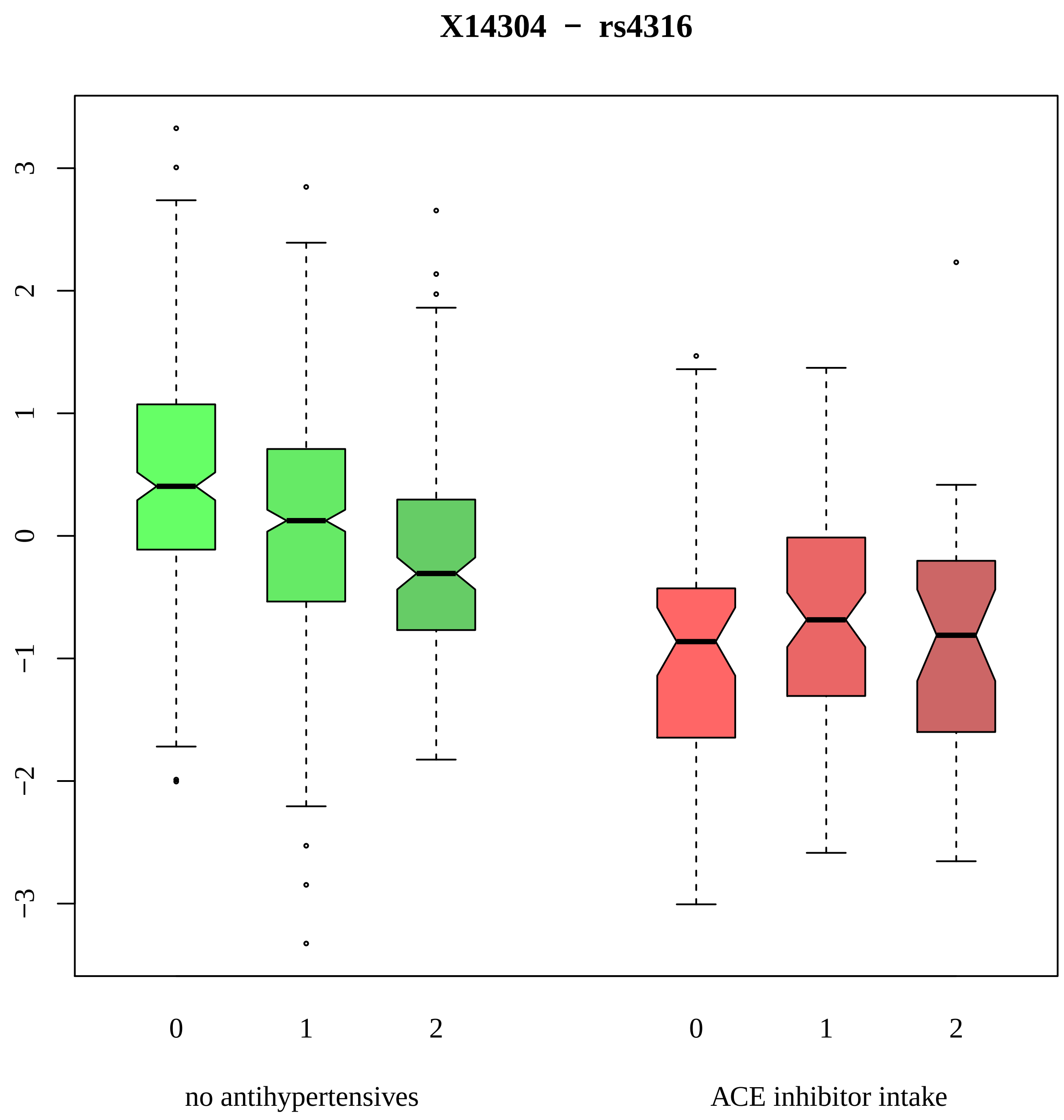
X14304 – rs4316
3
2
1
0
−1
−2
−3
0
1
2
0
1
2
no antihypertensives
ACE inhibitor intake

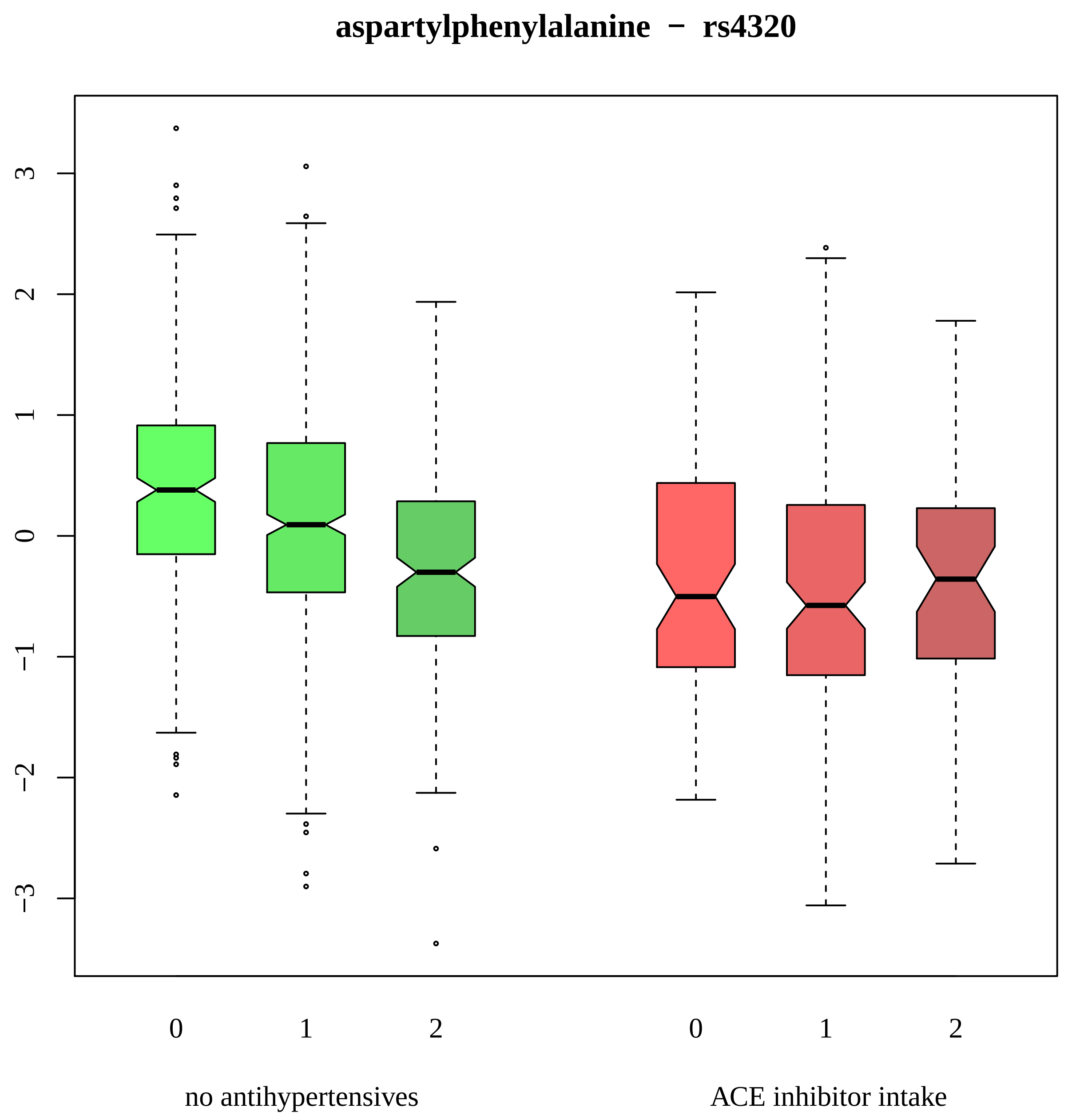

aspartylphenylalanine − rs4320
3
2
1
0
−1
−2
−3
0
1
2
0
1
2
no antihypertensives
ACE inhibitor intake

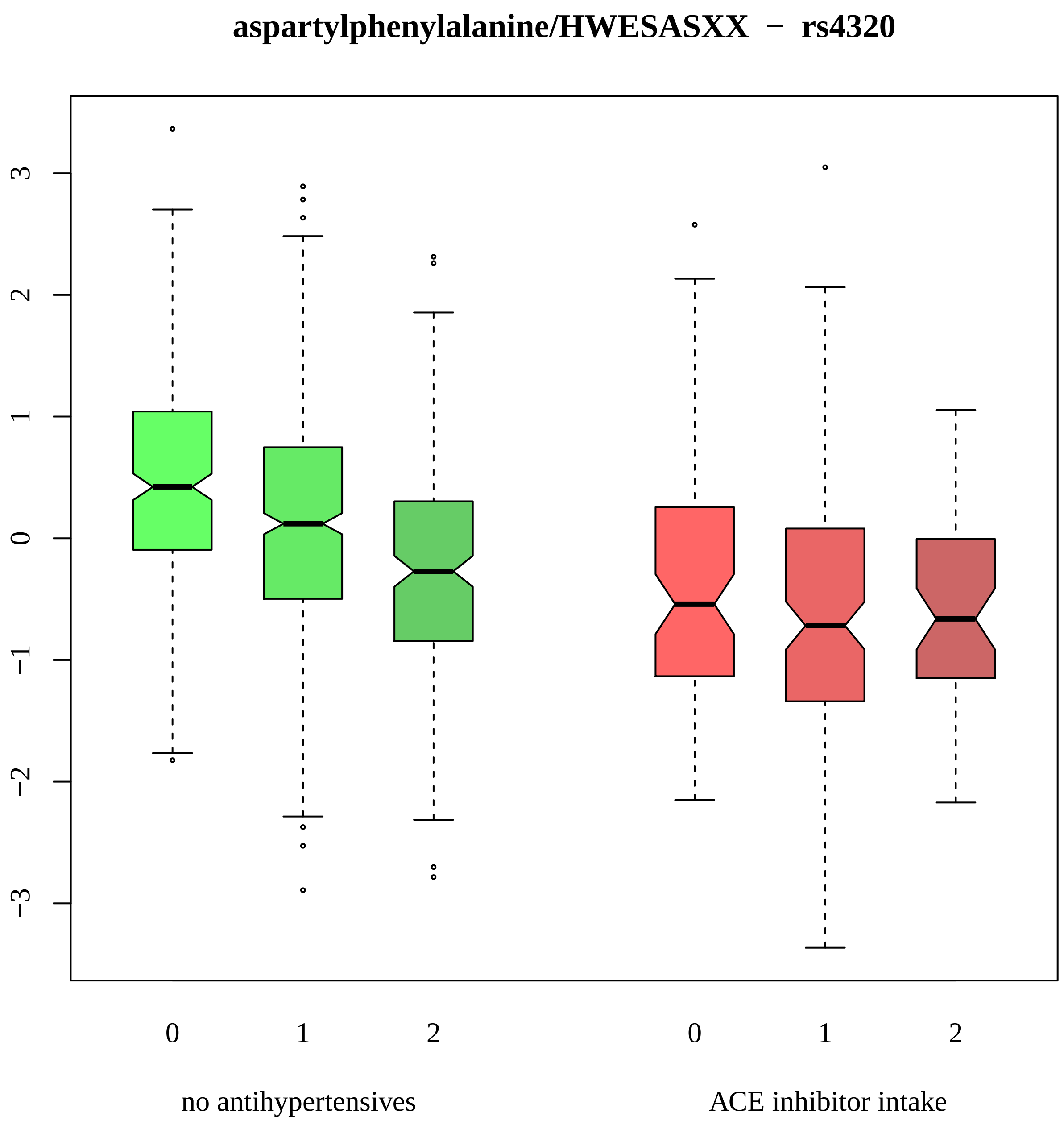
aspartylphenylalanine/HWESASXX – rs4320
3
2
1
0
−1
−2
−3
0
1
2
0
1
2
no antihypertensives
ACE inhibitor intake

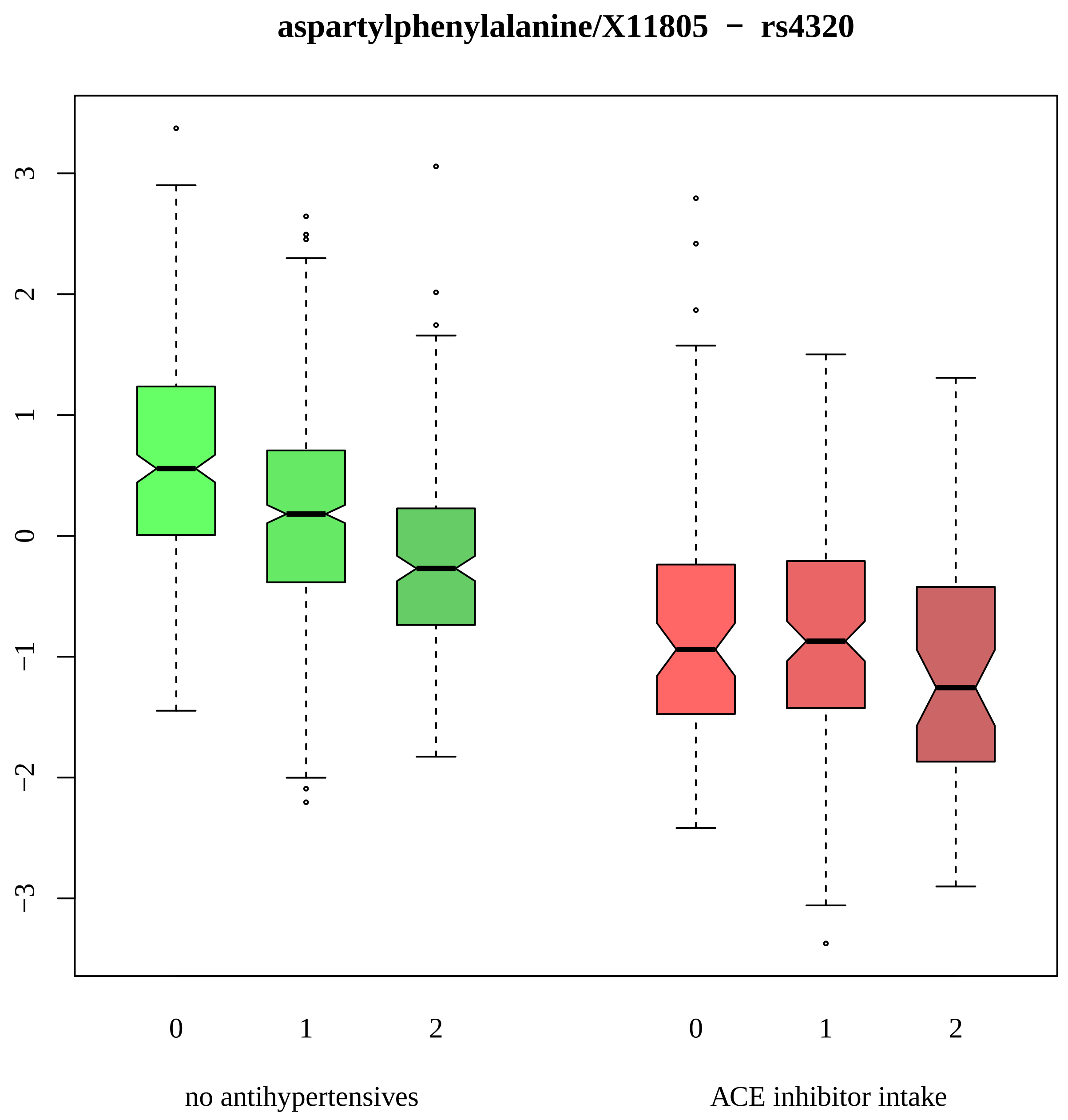

aspartylphenylalanine/X11805 – rs4320
3
2
1
0
−1
−2
−3
0
1
2
0
1
2
no antihypertensives
ACE inhibitor intake

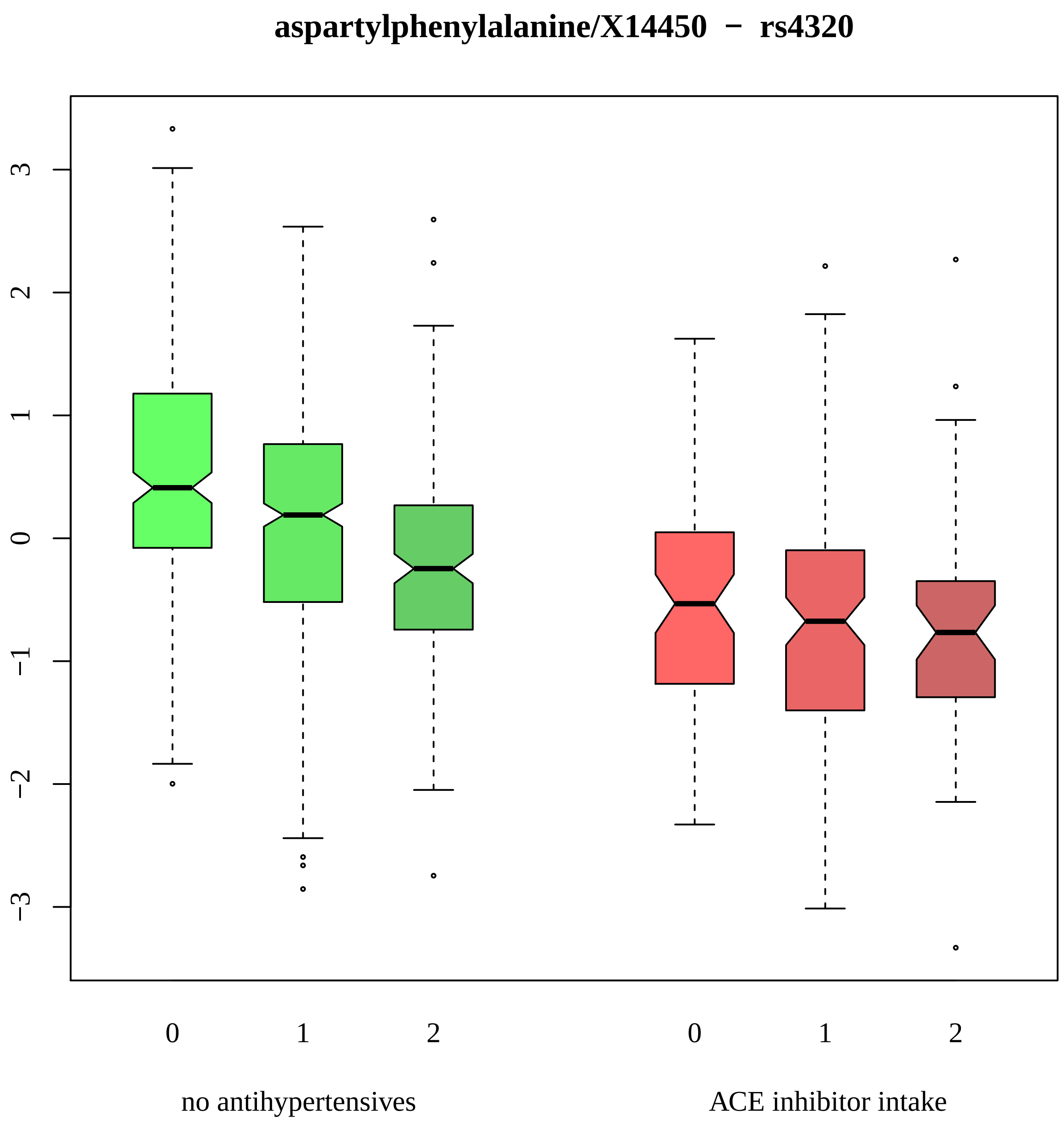

aspartylphenylalanine/X14450 − rs4320
3
2
1
0
−1
−2
−3
0
1
2
0
1
2
no antihypertensives
ACE inhibitor intake

X14086 – rs4320

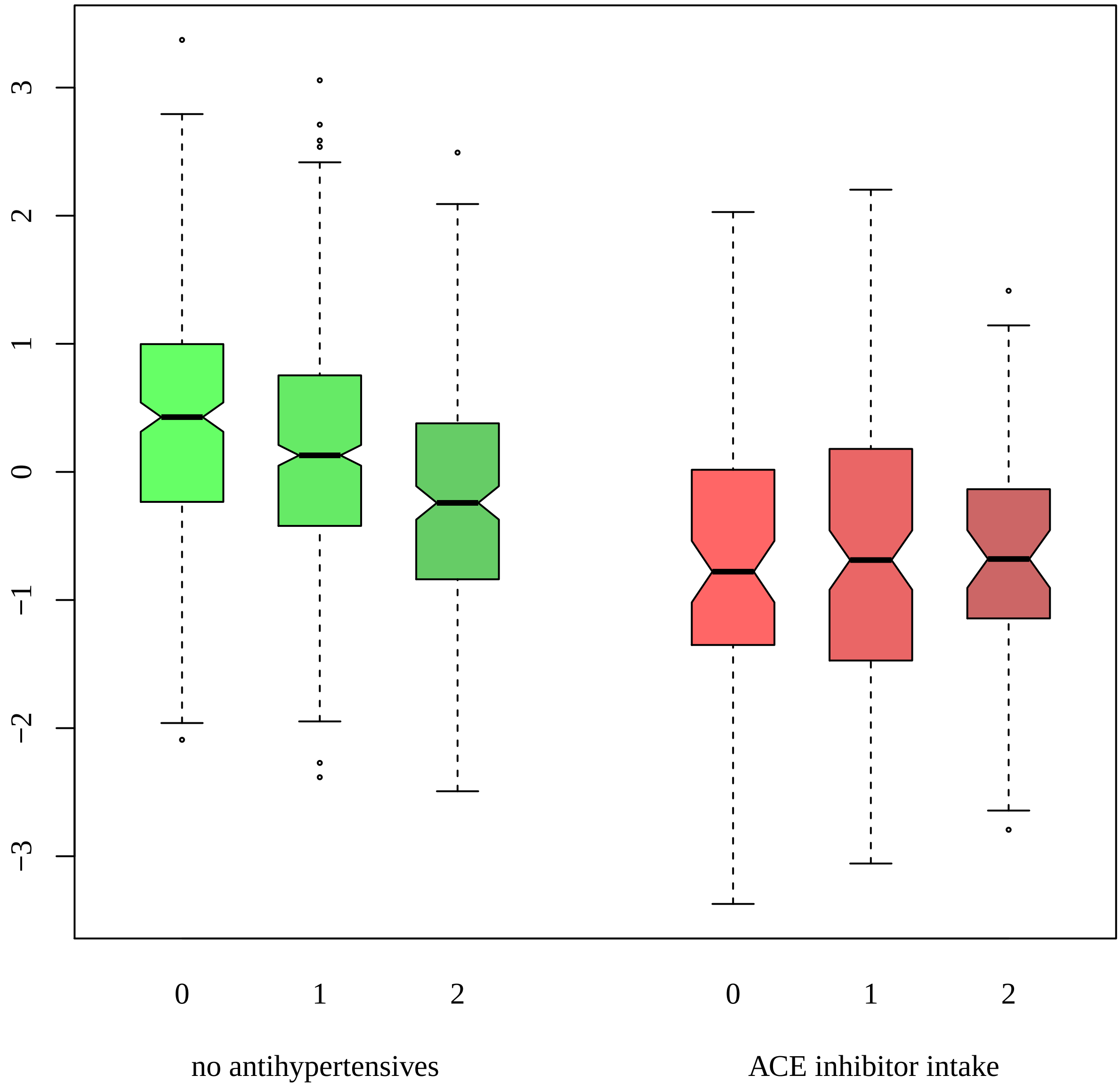

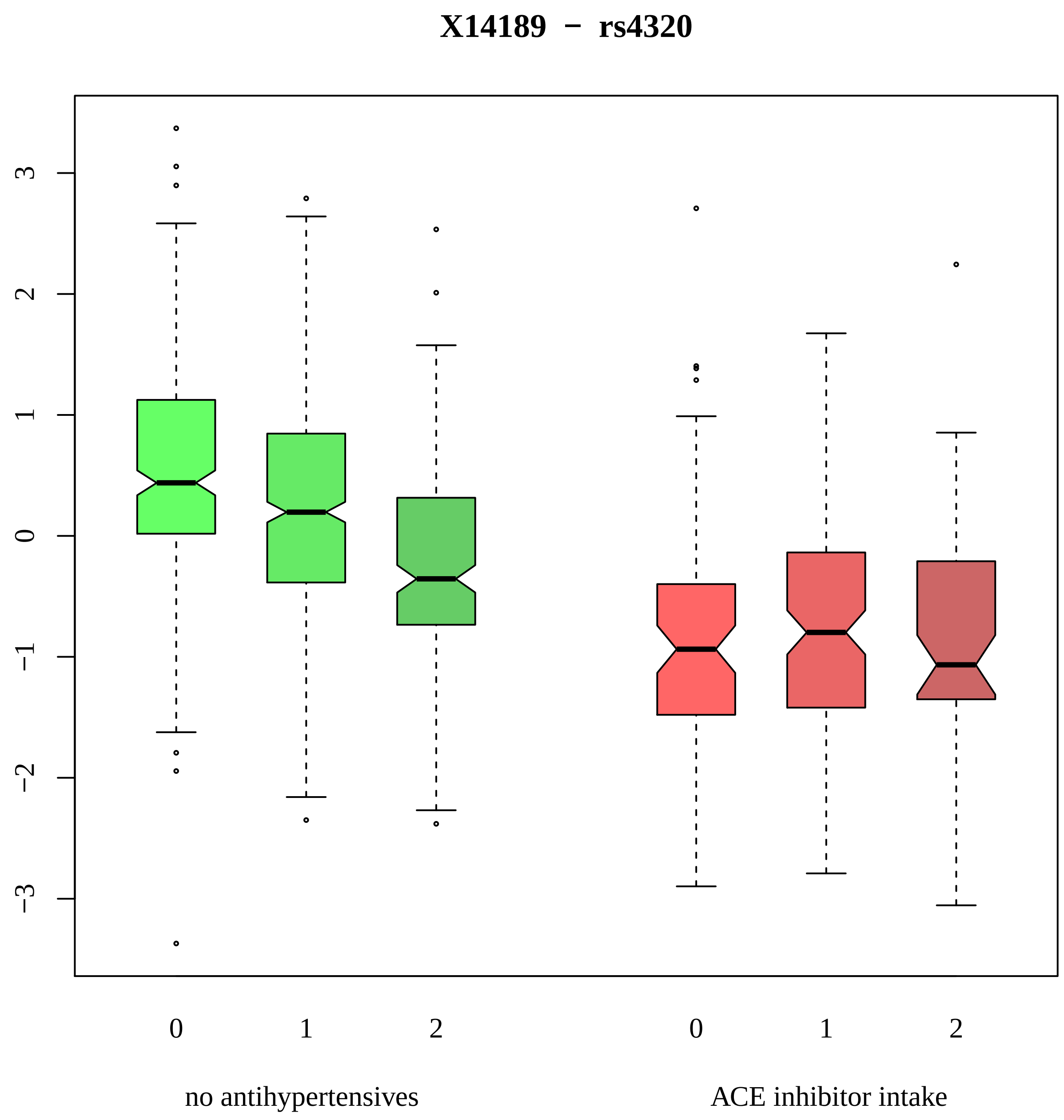
X14189 – rs4320
3
2
1
0
−1
−2
−3
0
1
2
0
1
2
no antihypertensives
ACE inhibitor intake

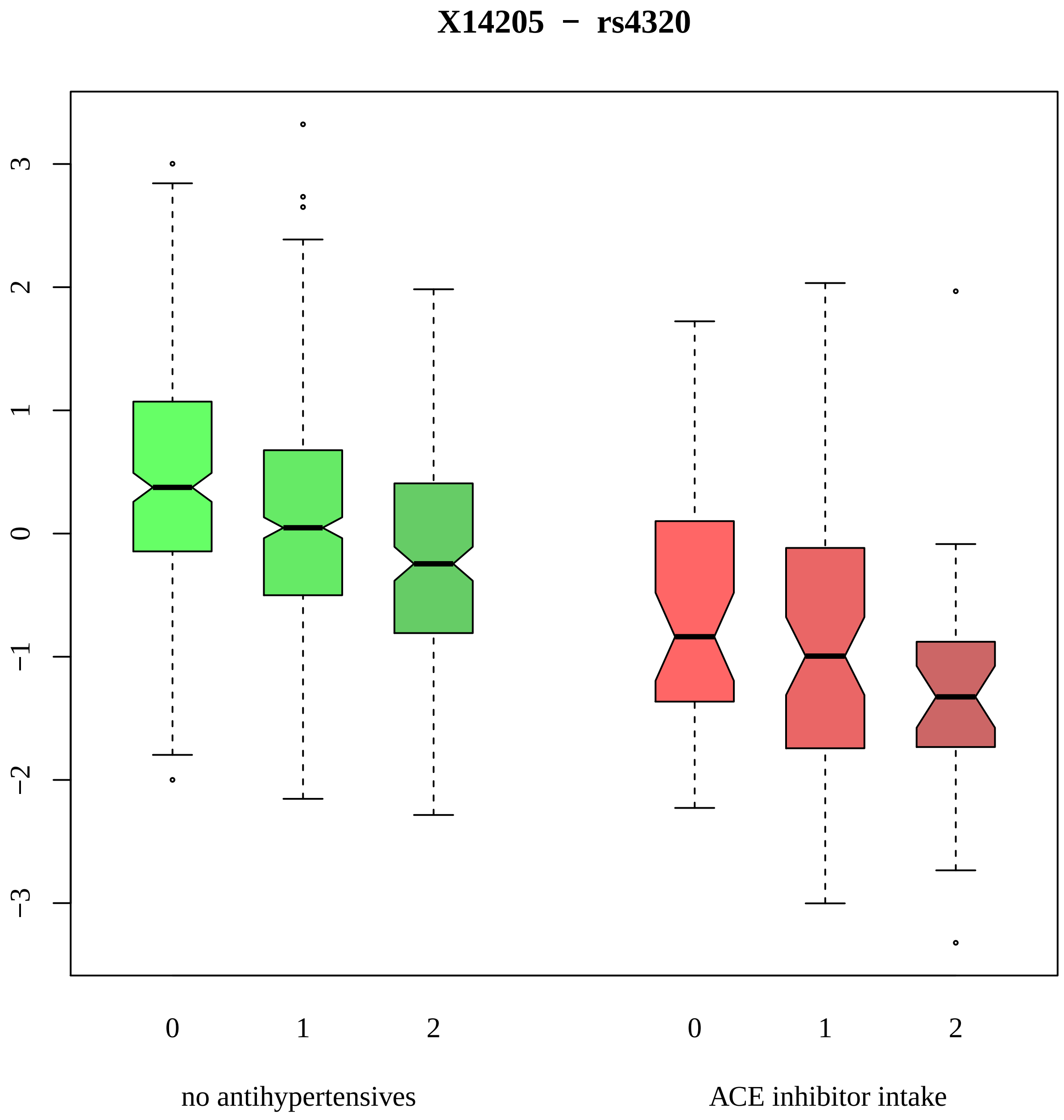
X14205 – rs4320
3
2
1
0
−1
−2
−3
0
1
2
0
1
2
no antihypertensives
ACE inhibitor intake

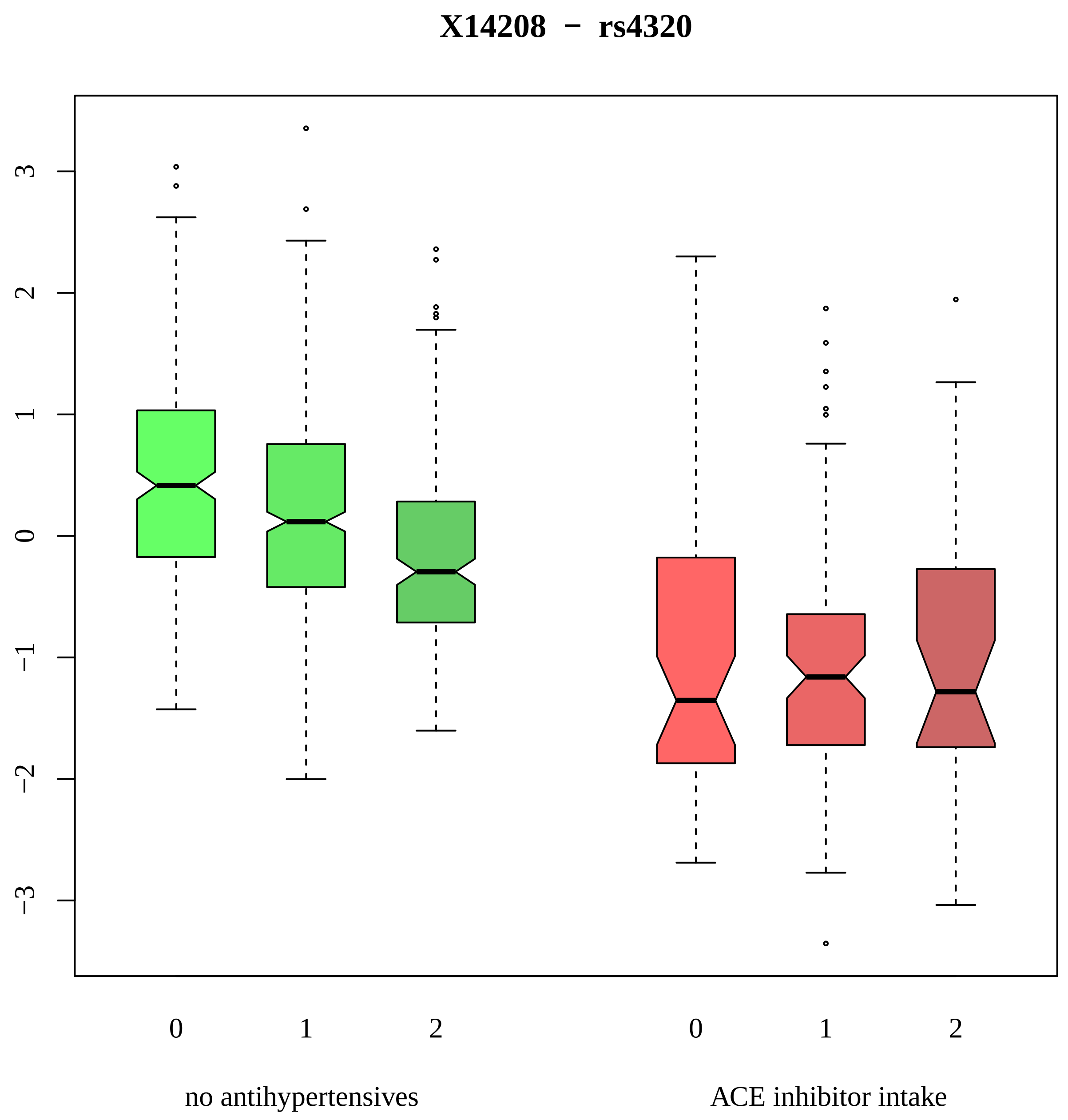

X14208 – rs4320
3
2
1
0
−1
−2
−3
0
1
2
0
1
2
no antihypertensives
ACE inhibitor intake

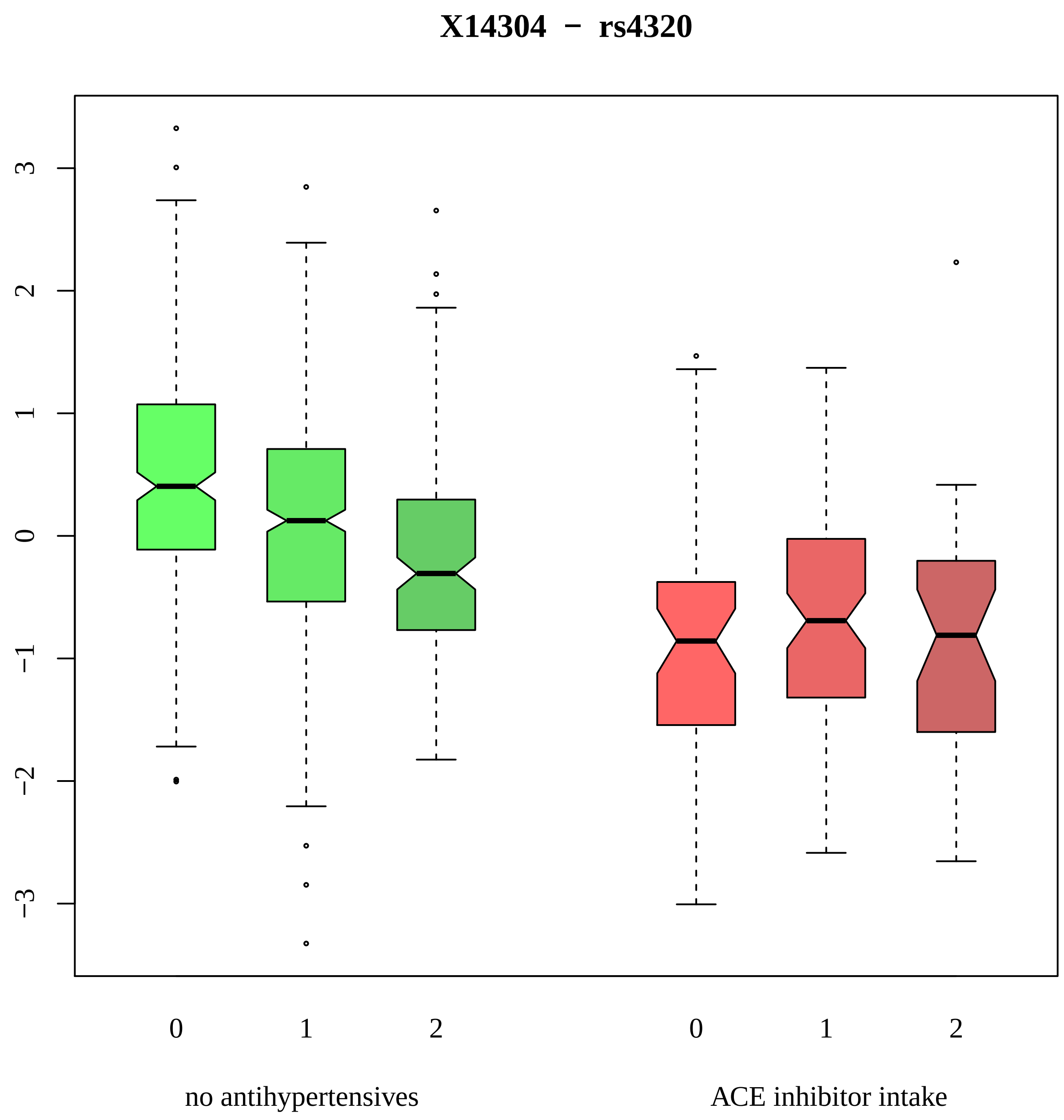
X14304 – rs4320
3
2
1
0
−1
−2
−3
0
1
2
0
1
2
no antihypertensives
ACE inhibitor intake

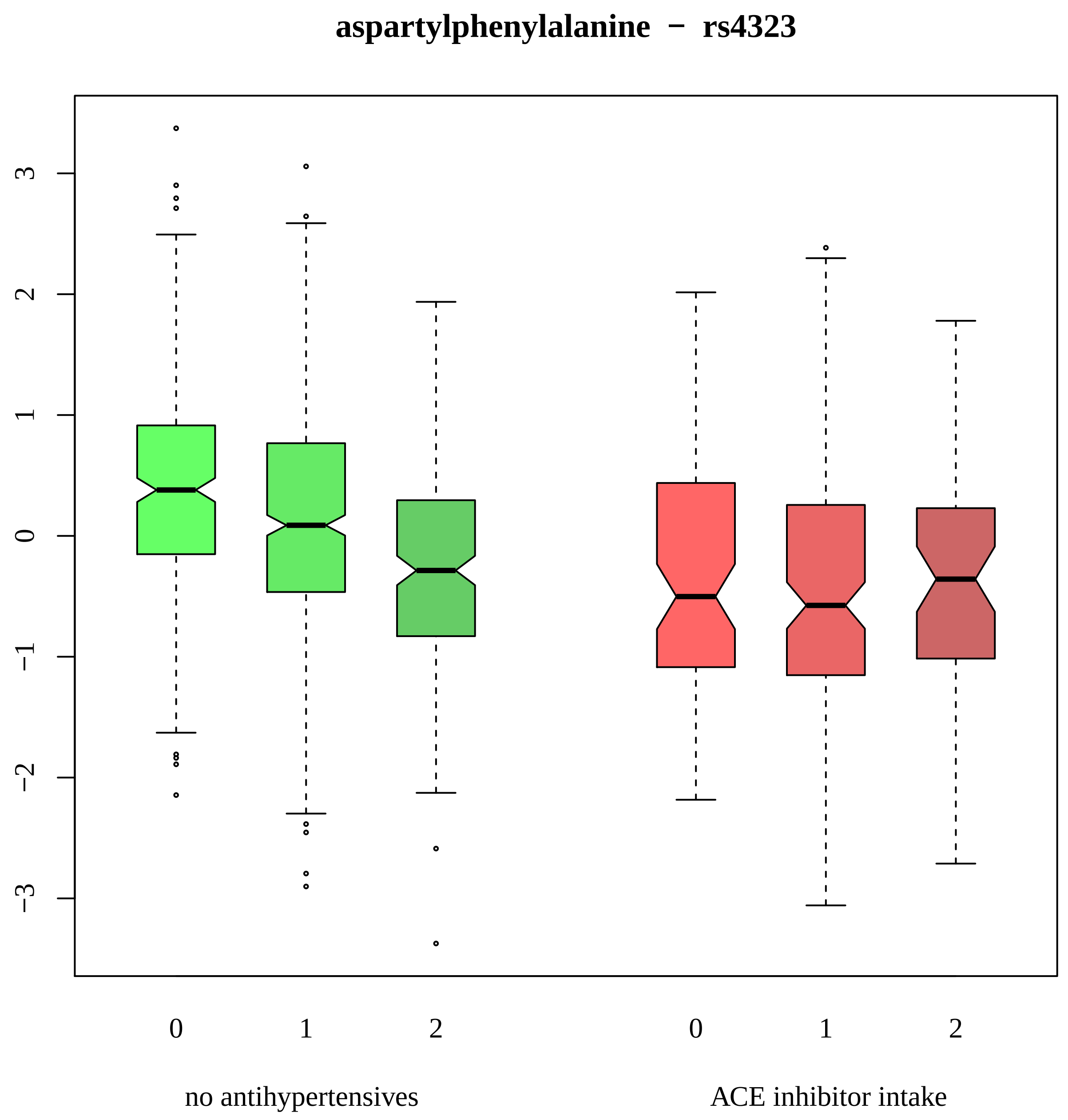
aspartylphenylalanine − rs4323
3
2
1
0
−1
−2
−3
0
1
2
0
1
2
no antihypertensives
ACE inhibitor intake

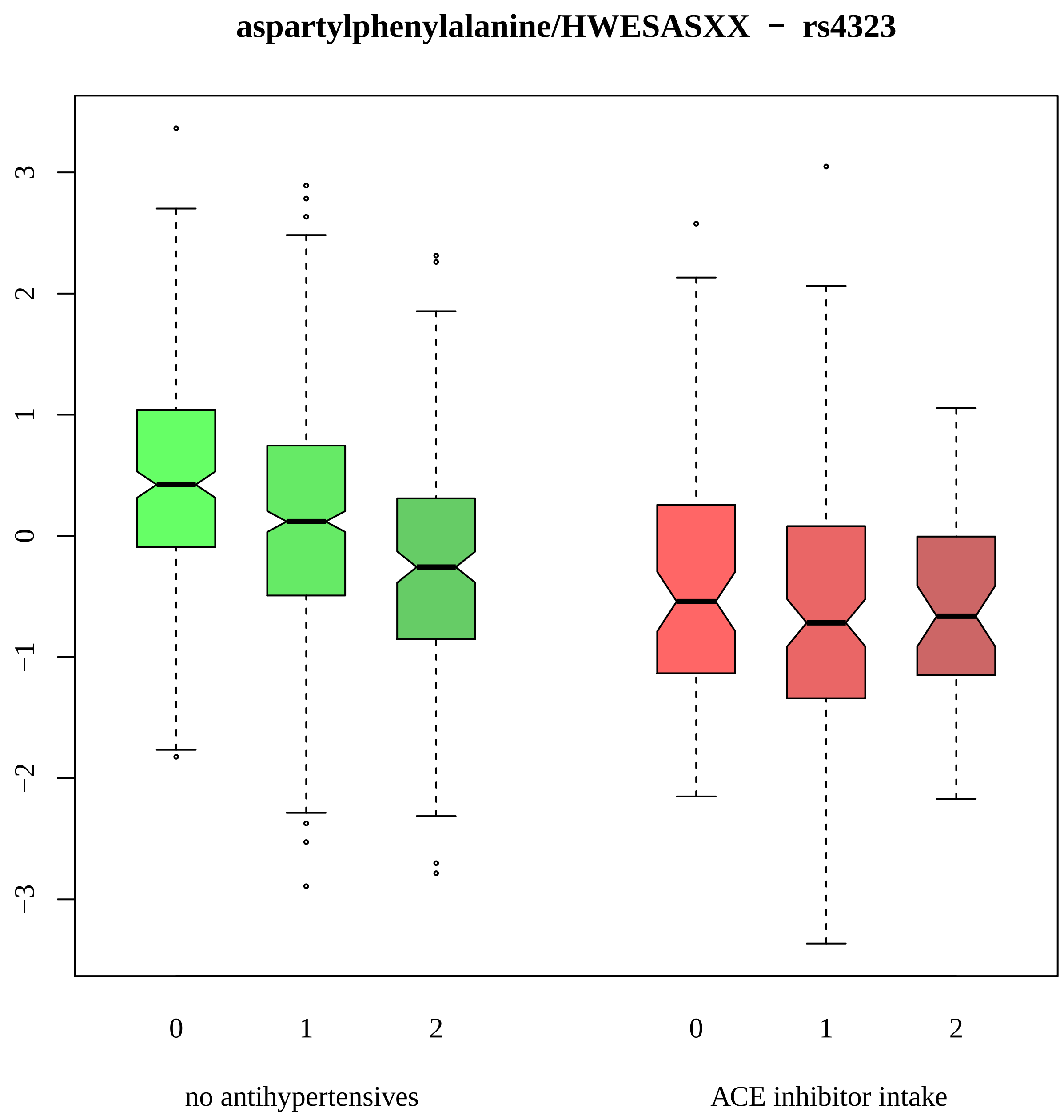

aspartylphenylalanine/HWESASXX – rs4323
3
2
1
0
−1
−2
−3
0
1
2
0
1
2
no antihypertensives
ACE inhibitor intake

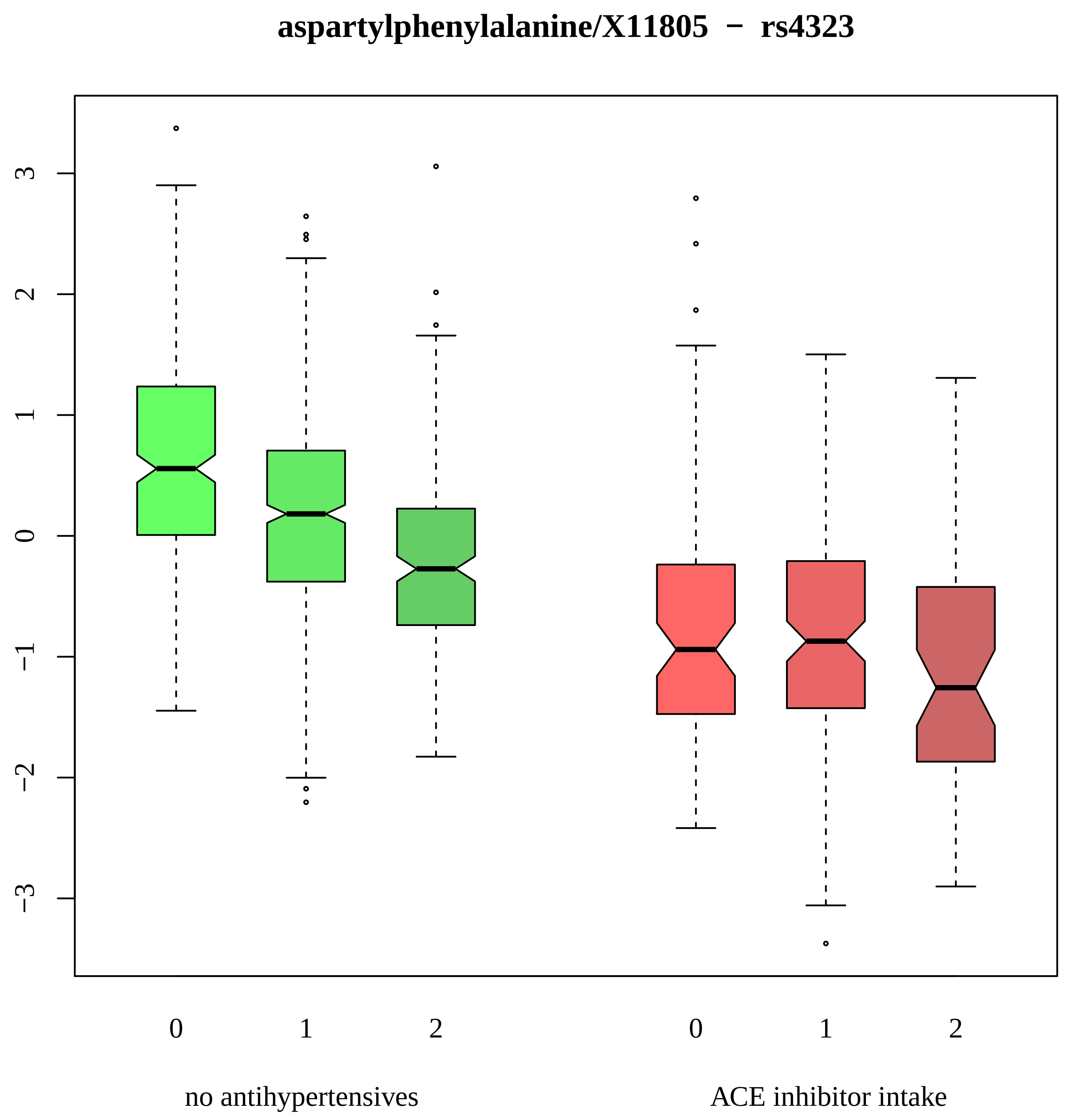
aspartylphenylalanine/X11805 – rs4323
3
2
1
0
−1
−2
−3
0
1
2
0
1
2
no antihypertensives
ACE inhibitor intake

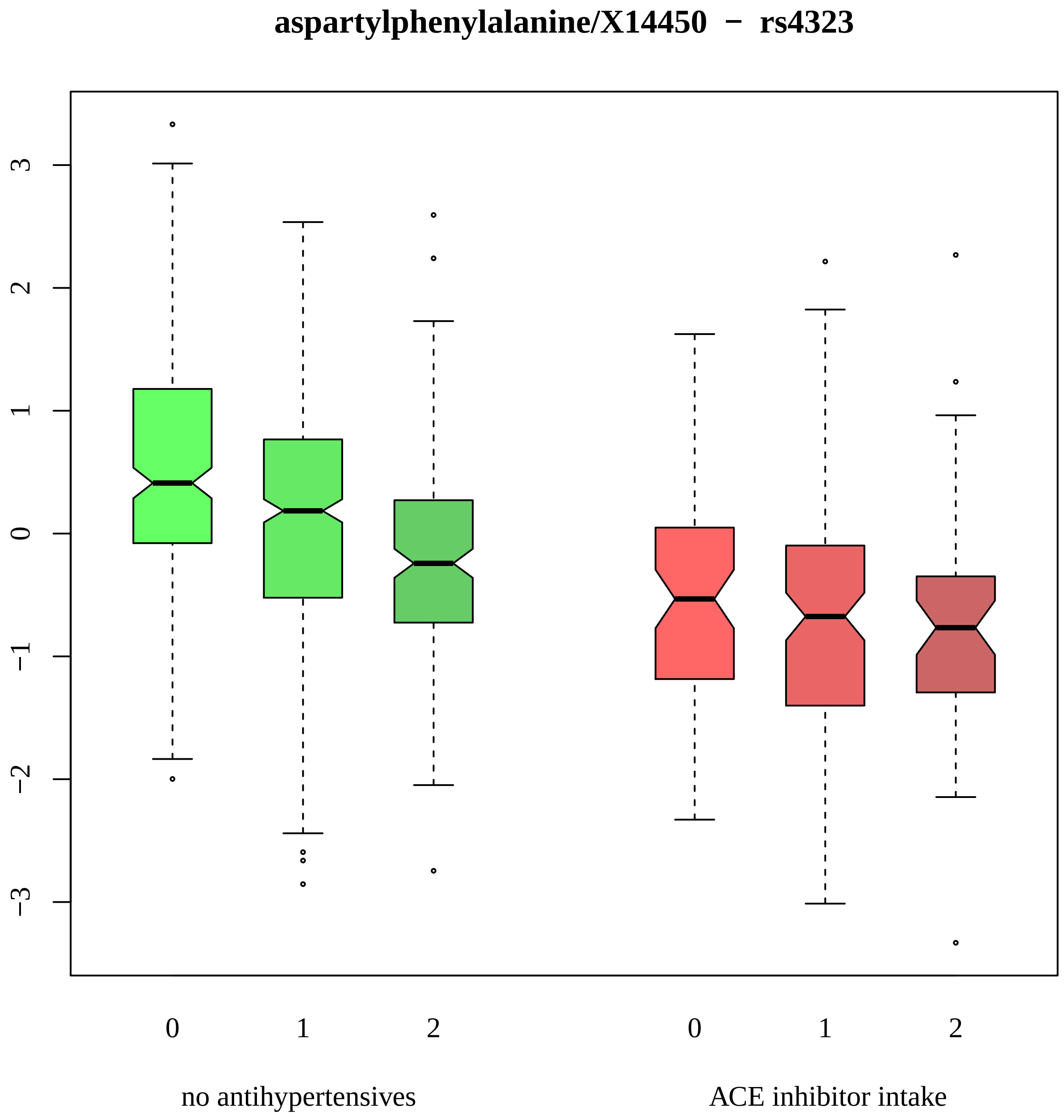
aspartylphenylalanine/X14450 – rs4323
3
2
1
0
−1
−2
−3
0
1
2
0
1
2
no antihypertensives
ACE inhibitor intake

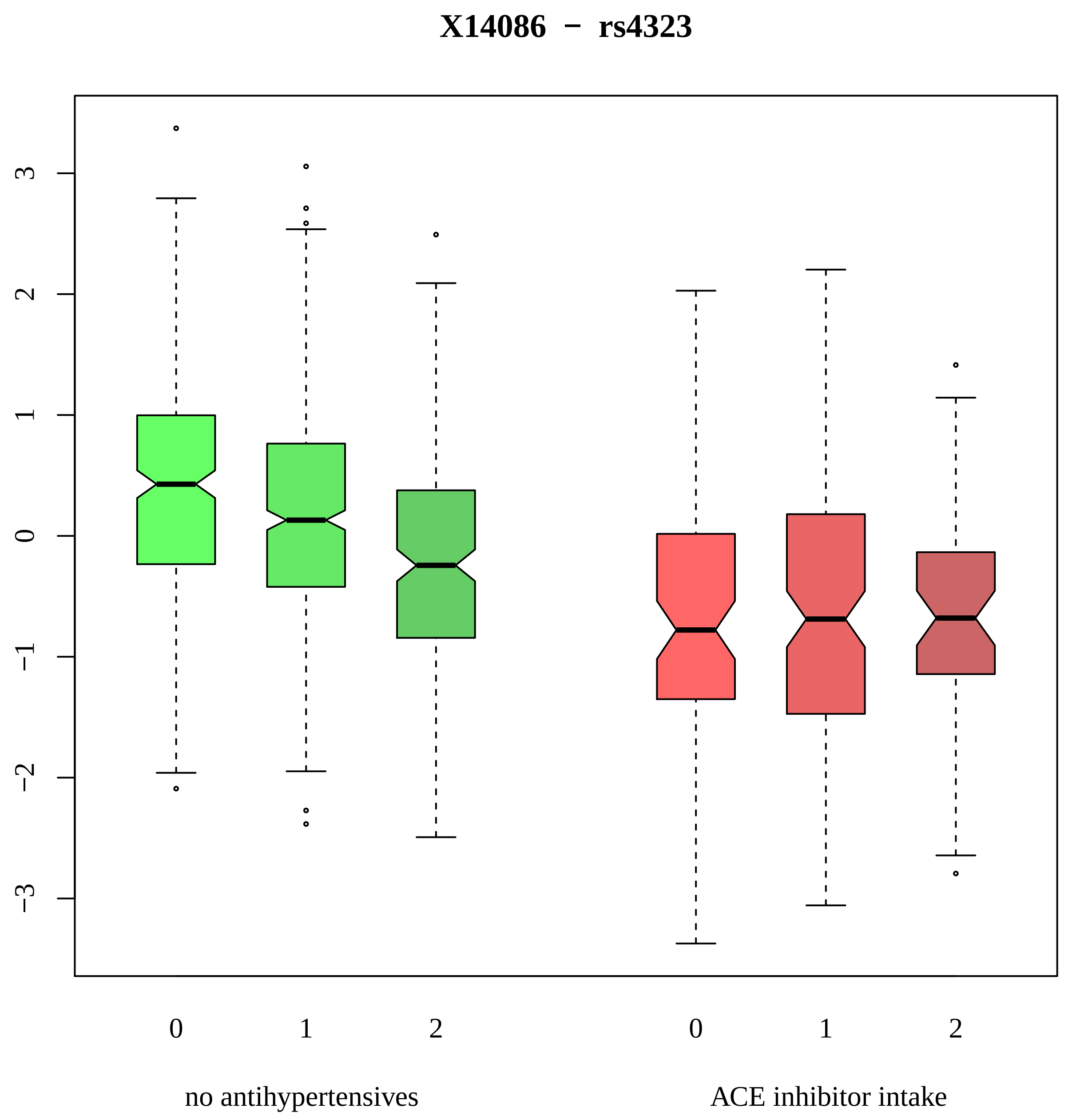

X14086 – rs4323
3
2
1
0
−1
−2
−3
0
1
2
0
1
2
no antihypertensives
ACE inhibitor intake

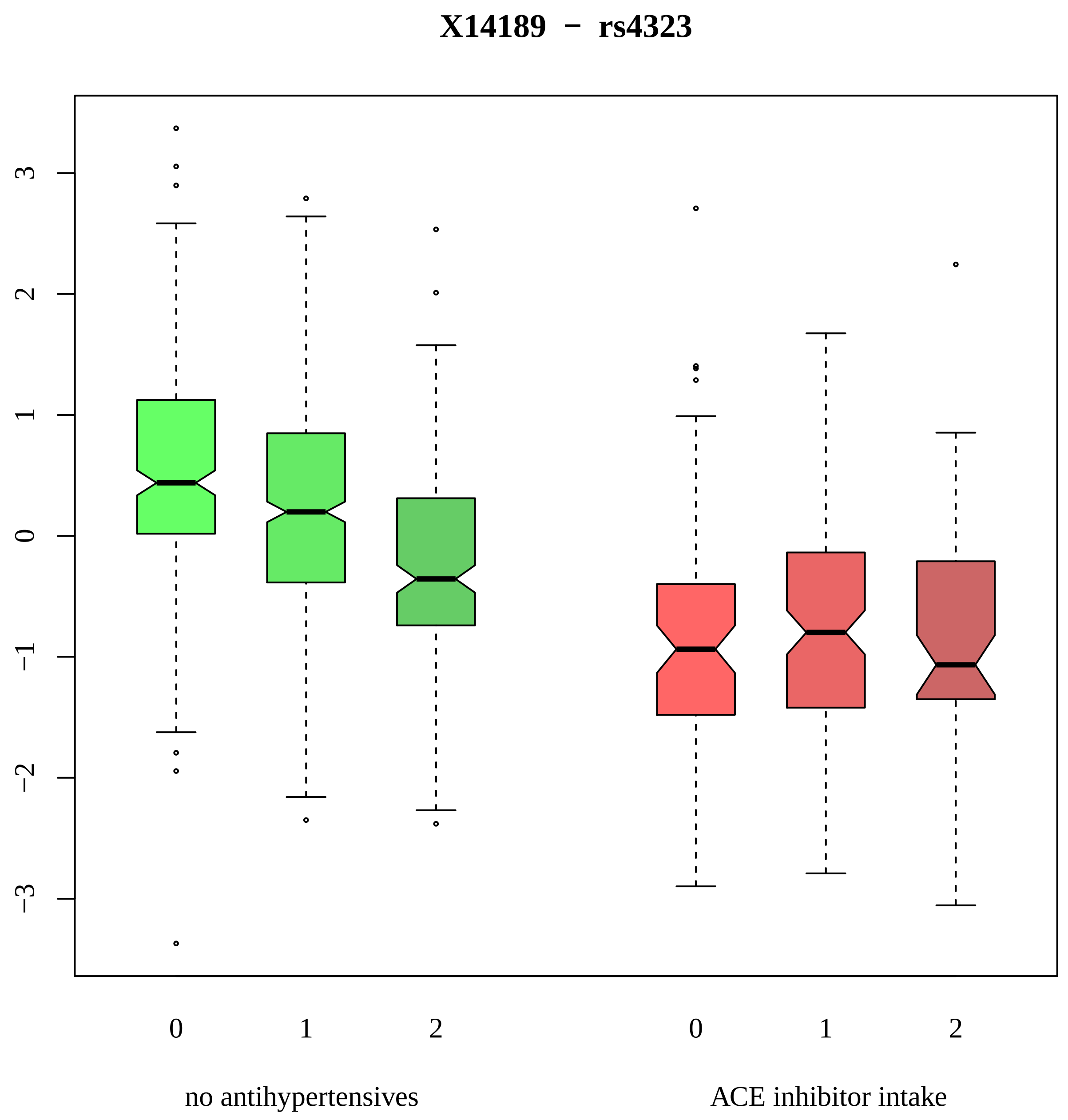

X14189 – rs4323
3
2
1
0
−1
−2
−3
0
1
2
0
1
2
no antihypertensives
ACE inhibitor intake

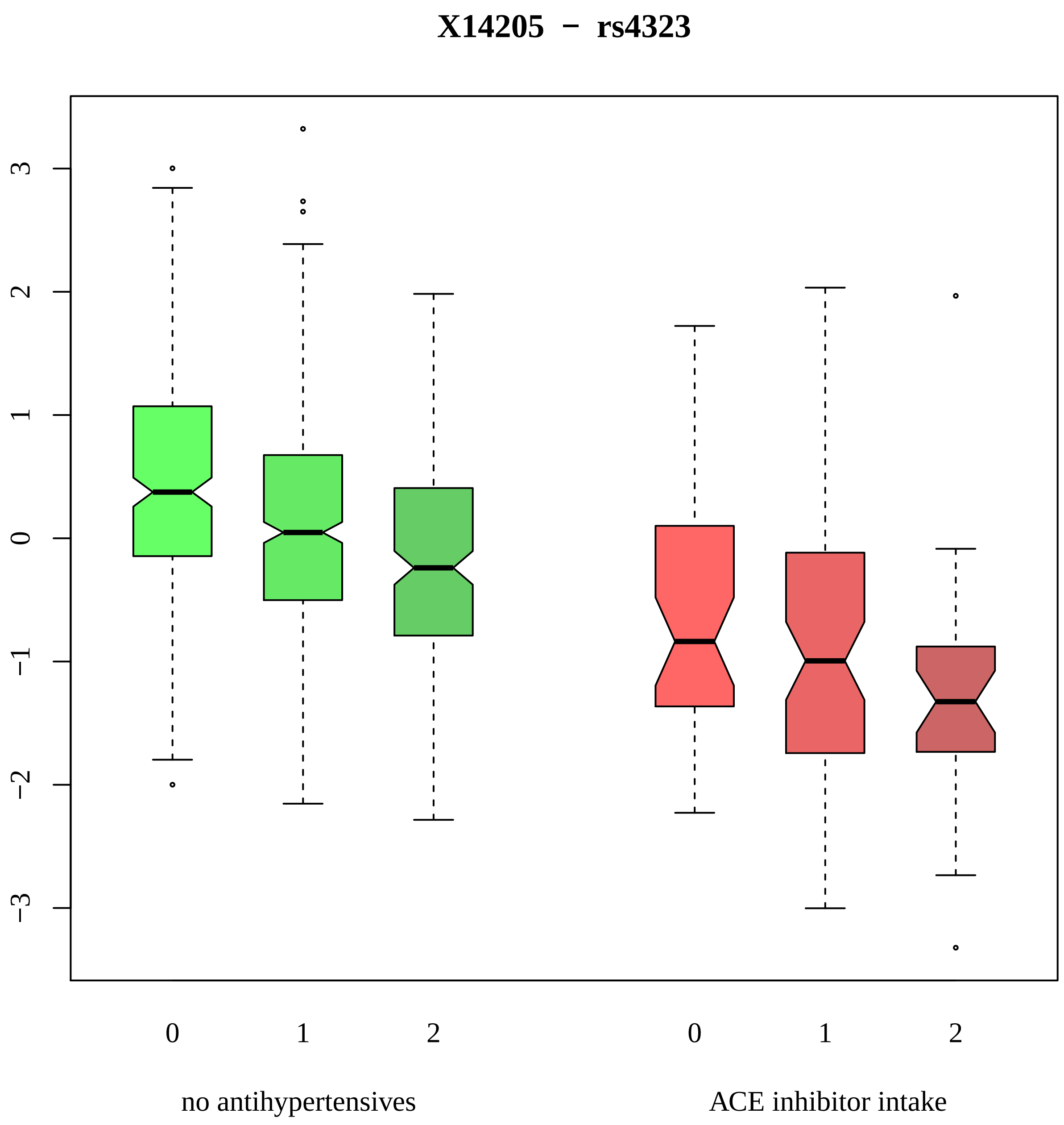

X14205 – rs4323
3
2
1
0
−1
−2
−3
0
1
2
0
1
2
no antihypertensives
ACE inhibitor intake

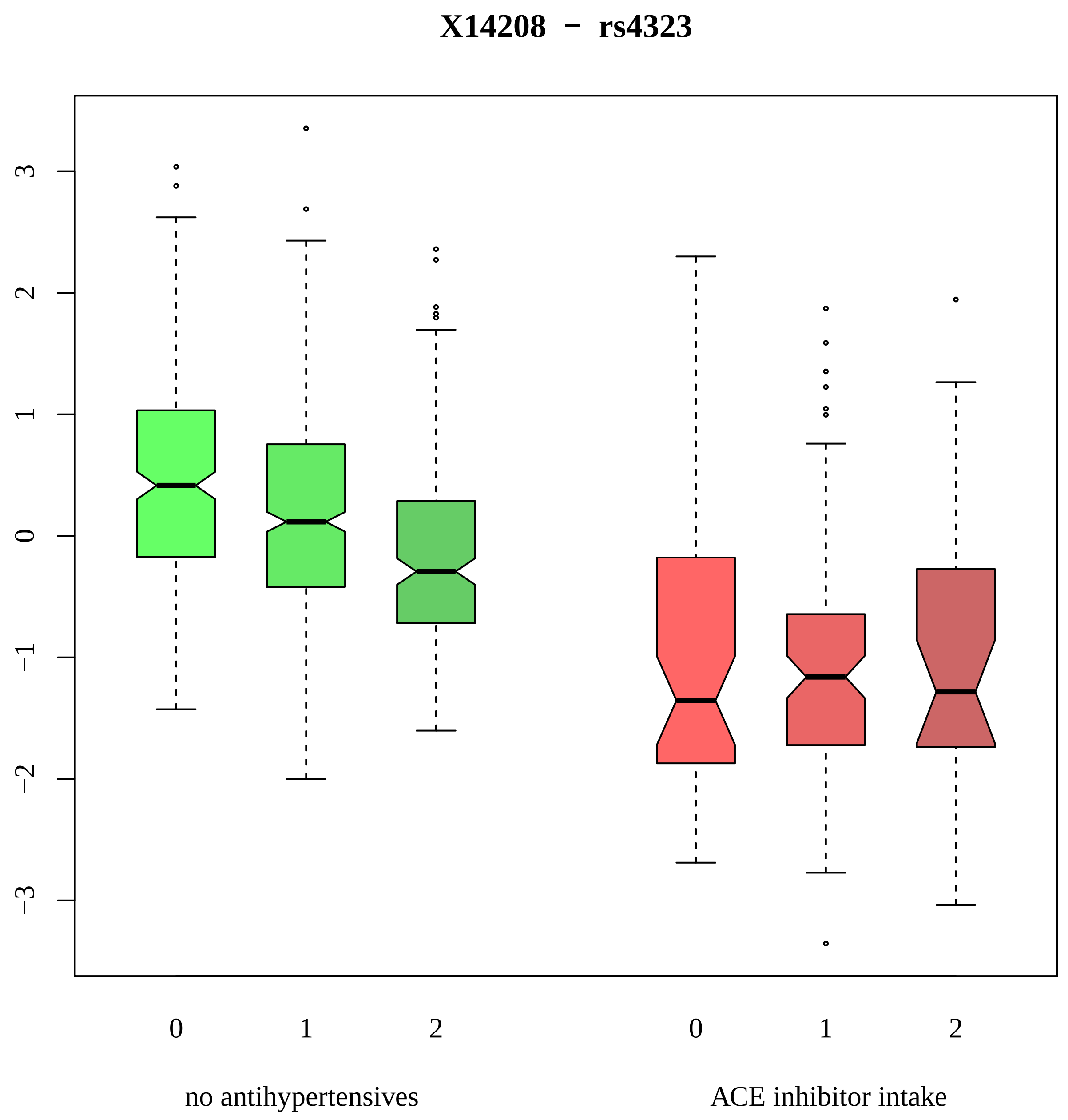

X14208 – rs4323
3
2
1
0
−1
−2
−3
0
1
2
0
1
2
no antihypertensives
ACE inhibitor intake

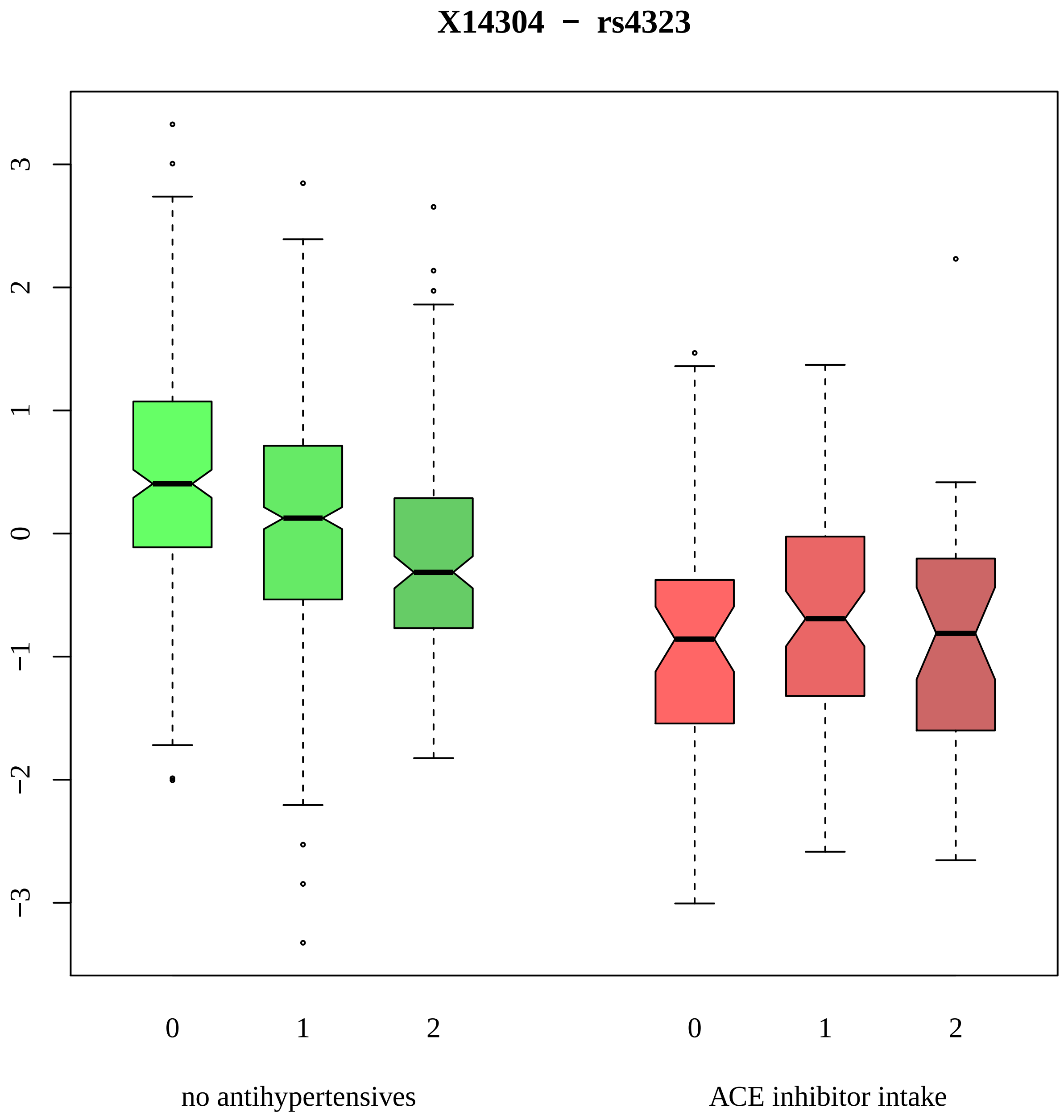

X14304 – rs4323
3
2
1
0
−1
−2
−3
0
1
2
0
1
2
no antihypertensives
ACE inhibitor intake

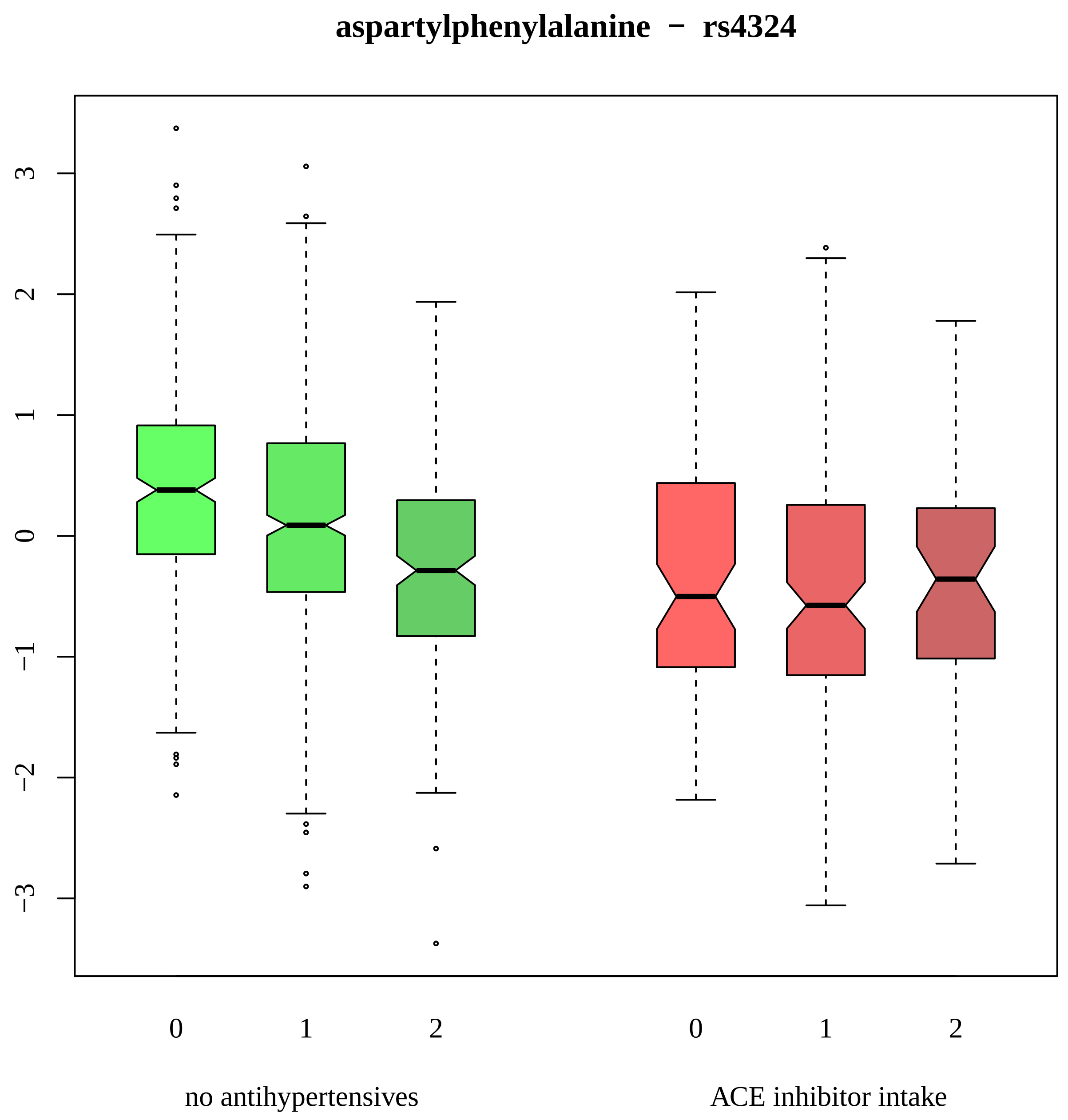

aspartylphenylalanine − rs4324
3
2
1
0
−1
−2
−3
0
1
2
0
1
2
no antihypertensives
ACE inhibitor intake

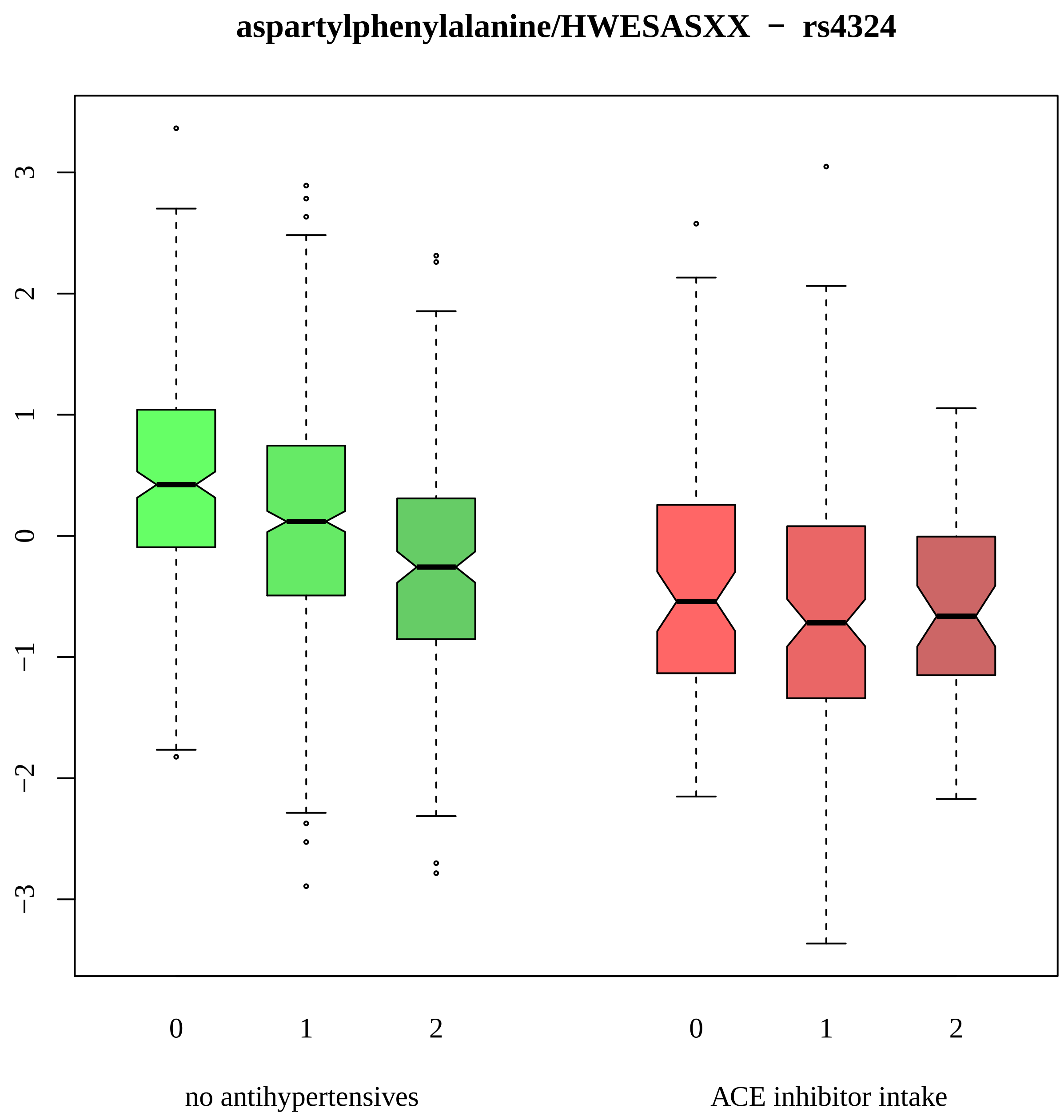
aspartylphenylalanine/HWESASXX – rs4324
3
2
1
0
−1
−2
−3
0
1
2
0
1
2
no antihypertensives
ACE inhibitor intake

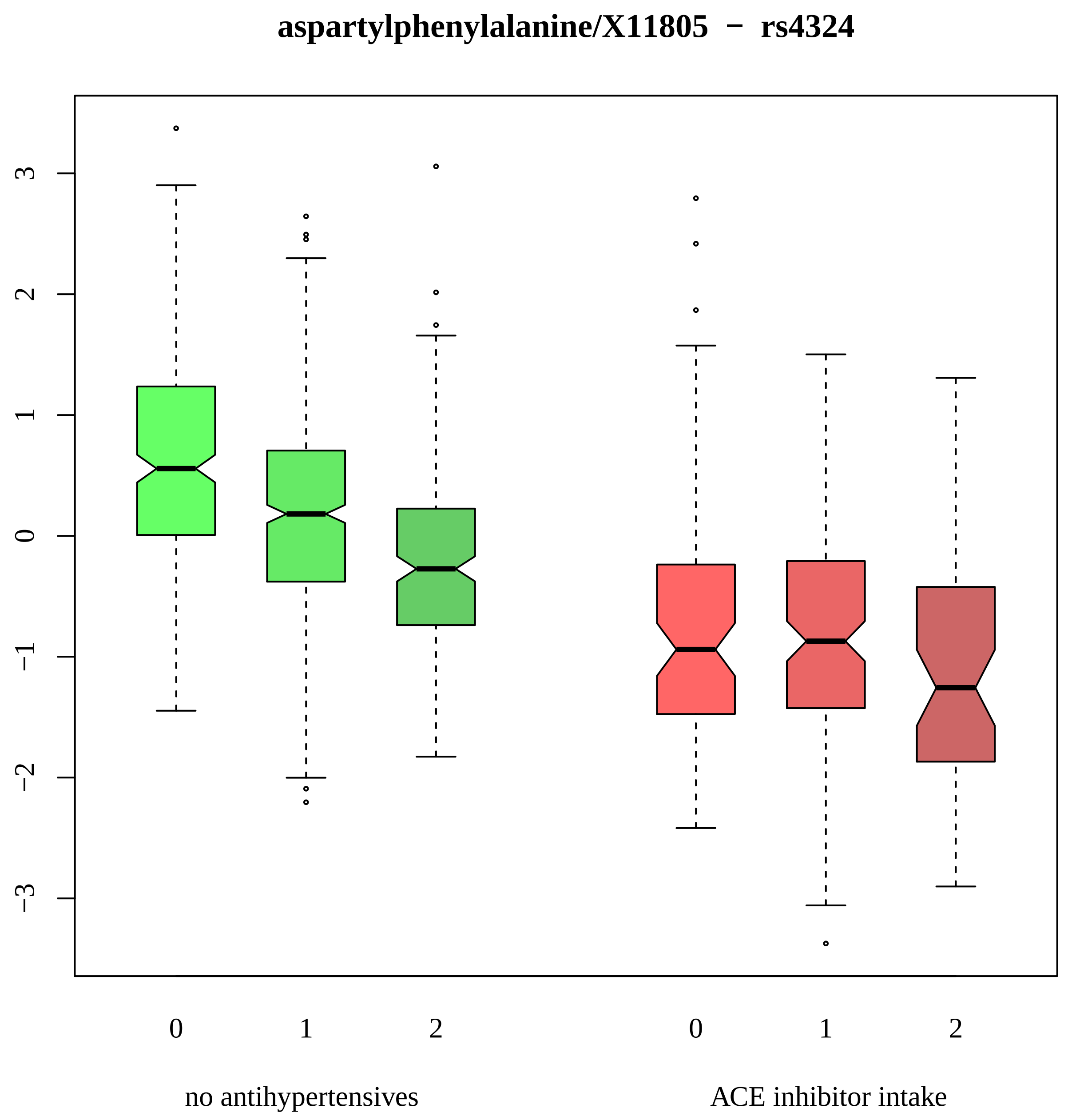
aspartylphenylalanine/X11805 – rs4324
3
2
1
0
−1
−2
−3
0
1
2
0
1
2
no antihypertensives
ACE inhibitor intake

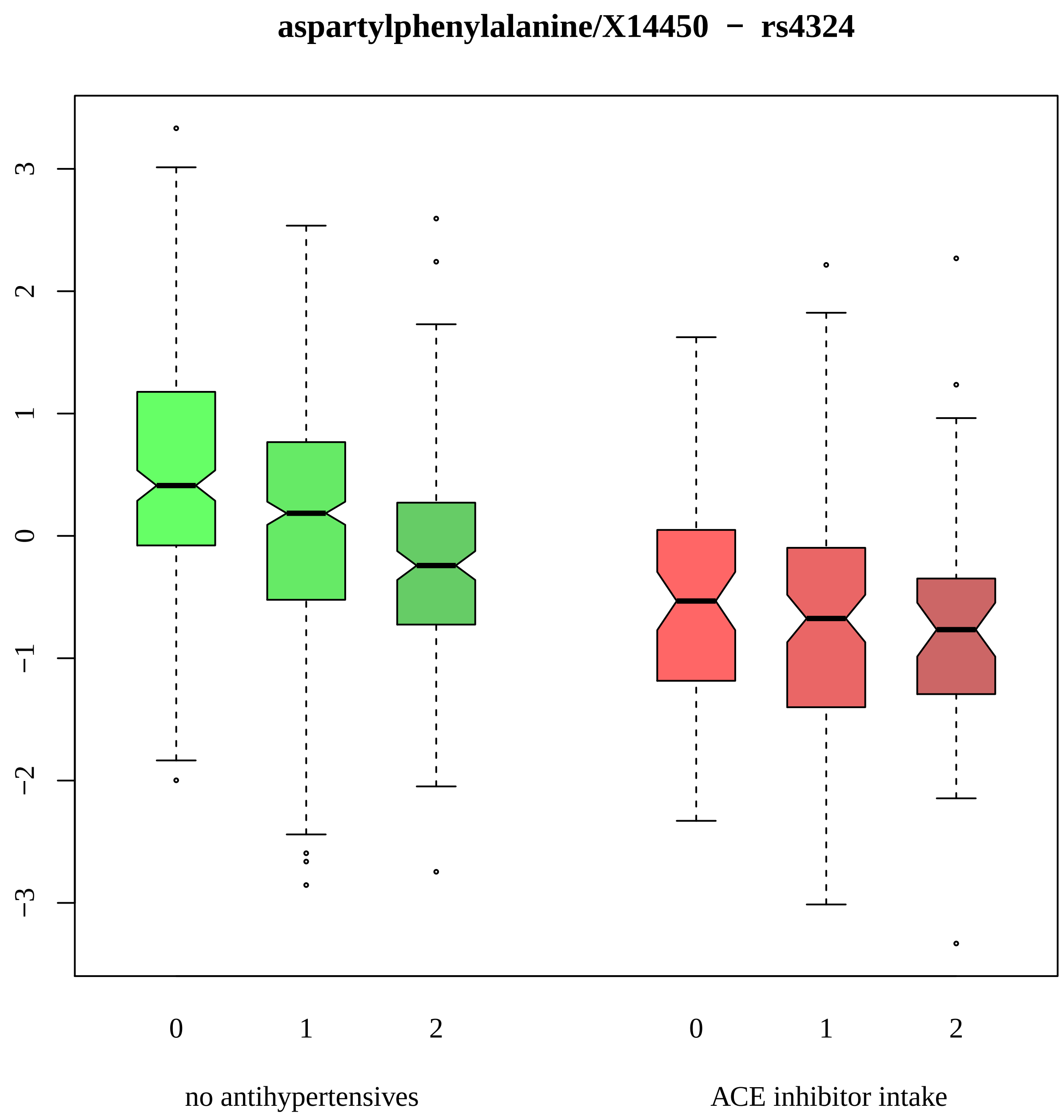

aspartylphenylalanine/X14450 – rs4324
3
2
1
0
−1
−2
−3
0
1
2
0
1
2
no antihypertensives
ACE inhibitor intake

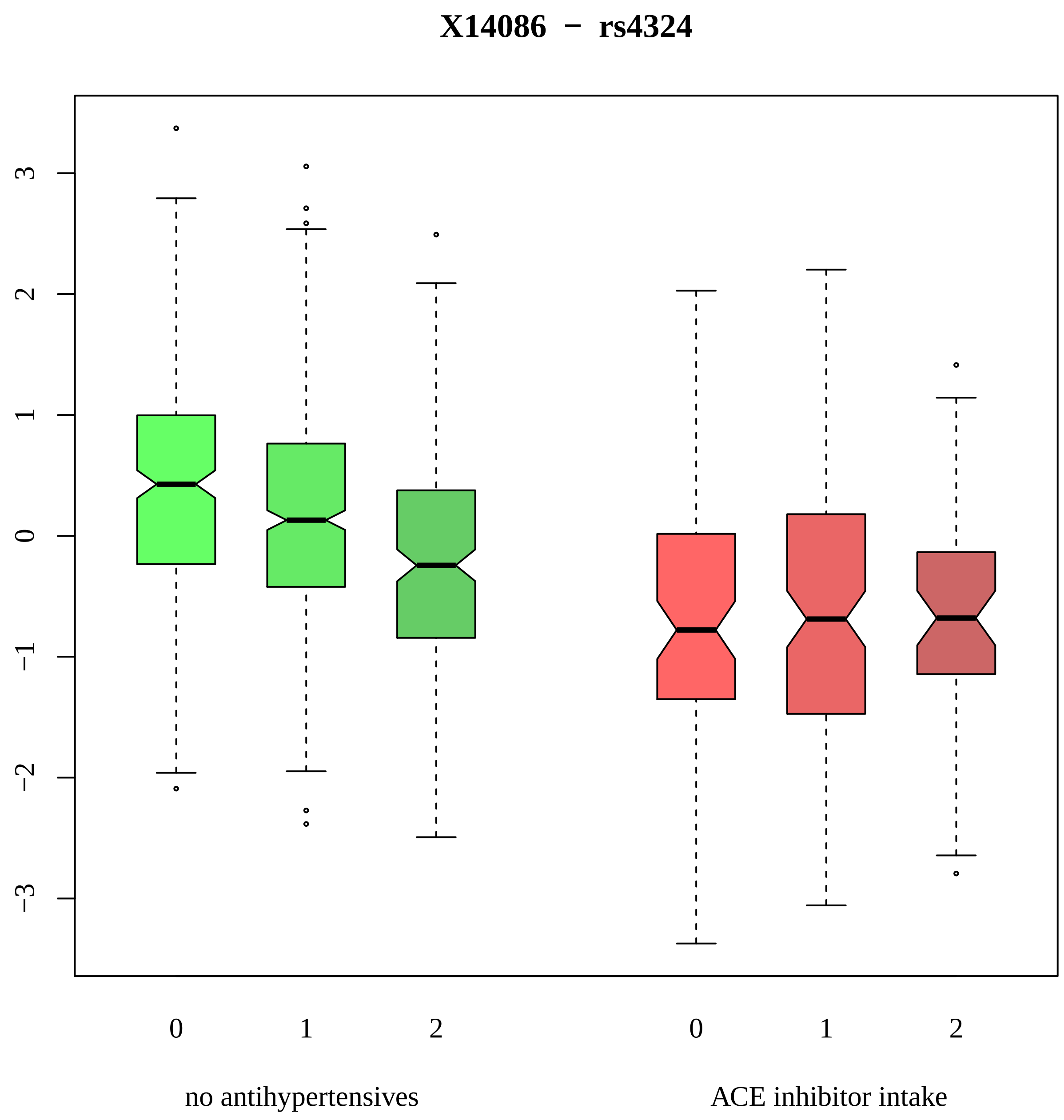
X14086 − rs4324
3
2
1
0
−1
−2
−3
0
1
2
0
1
2
no antihypertensives
ACE inhibitor intake

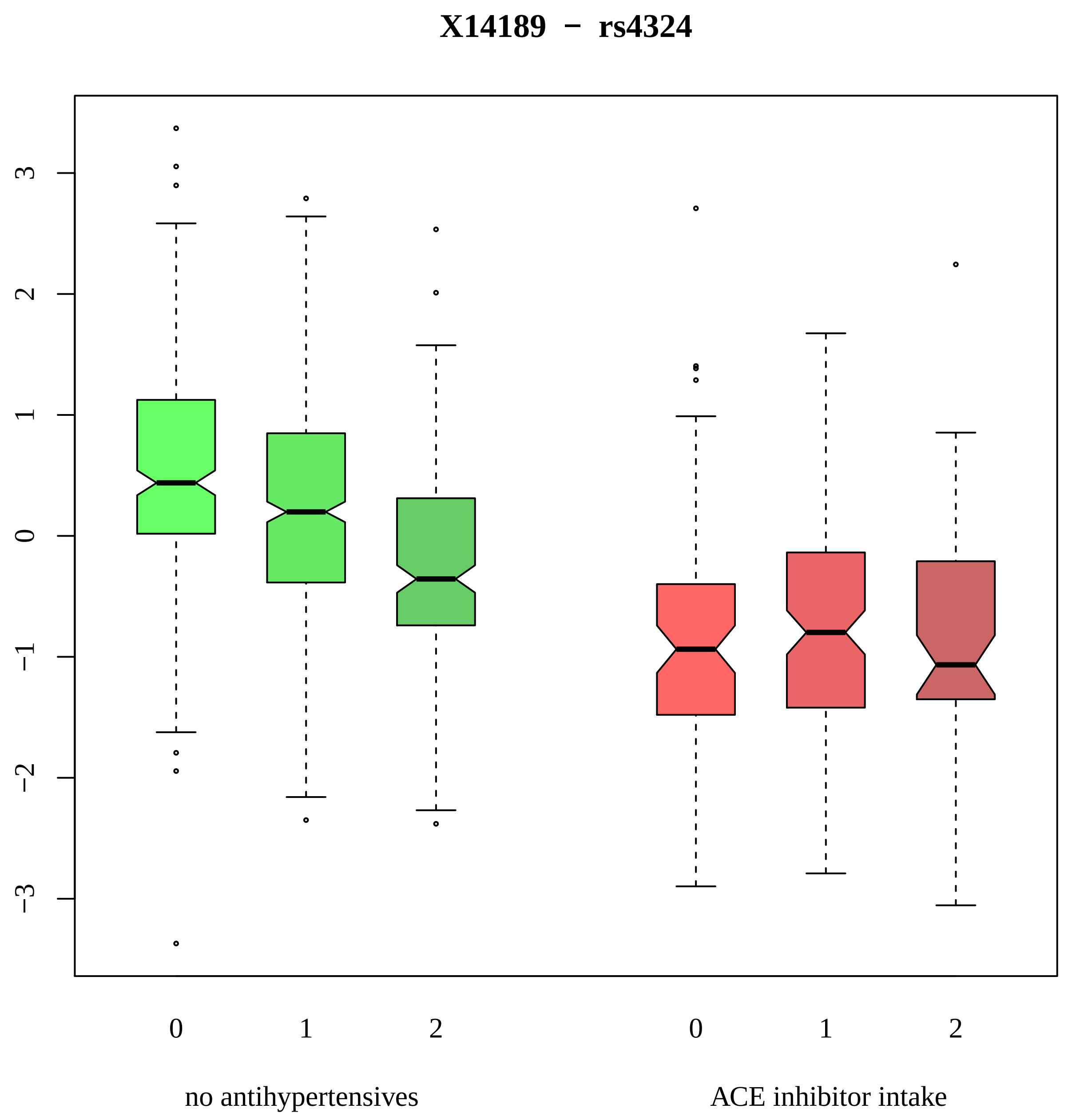
X14189 – rs4324
3
2
1
0
−1
−2
−3
0
1
2
0
1
2
no antihypertensives
ACE inhibitor intake

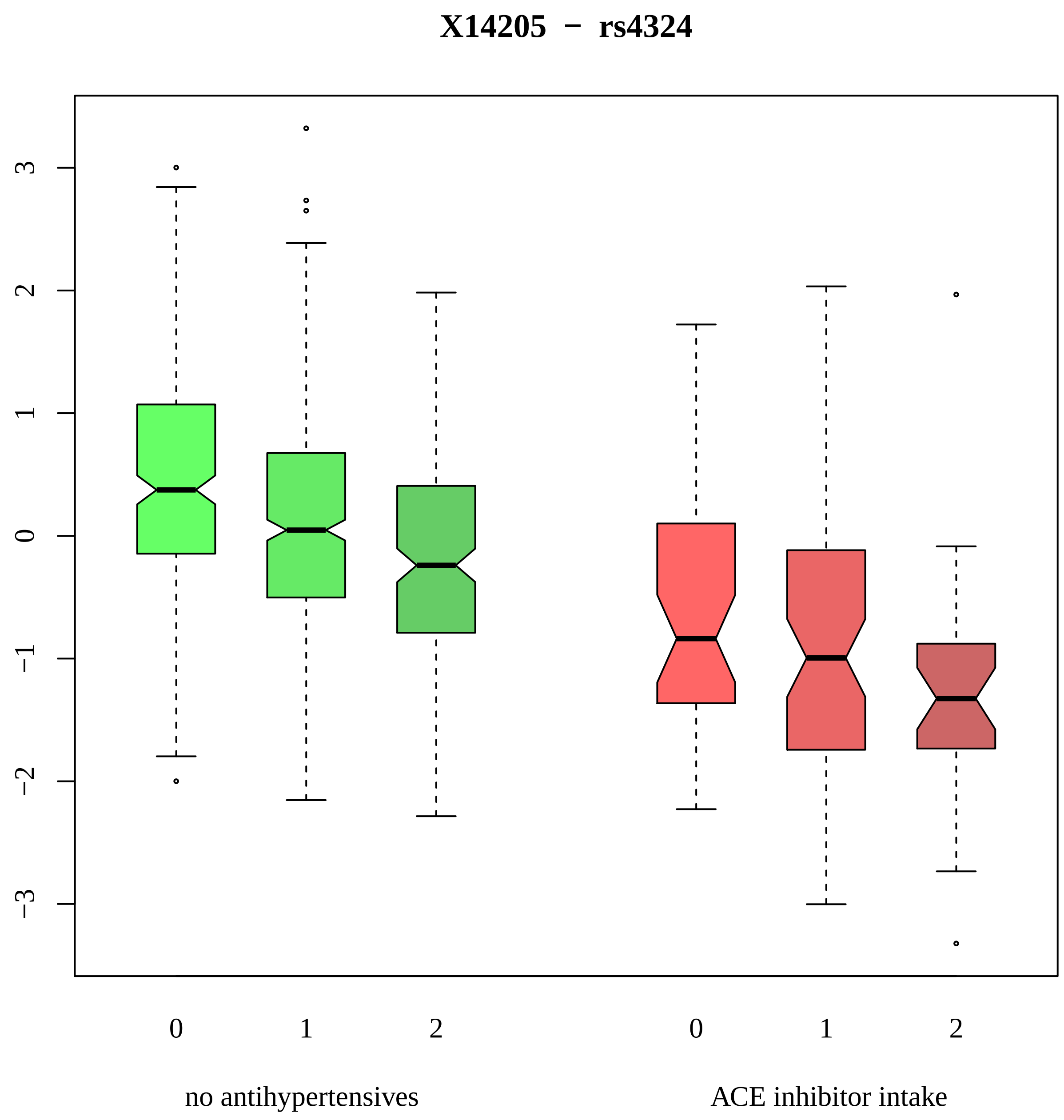

X14205 – rs4324
3
2
1
0
−1
−2
−3
0
1
2
0
1
2
no antihypertensives
ACE inhibitor intake

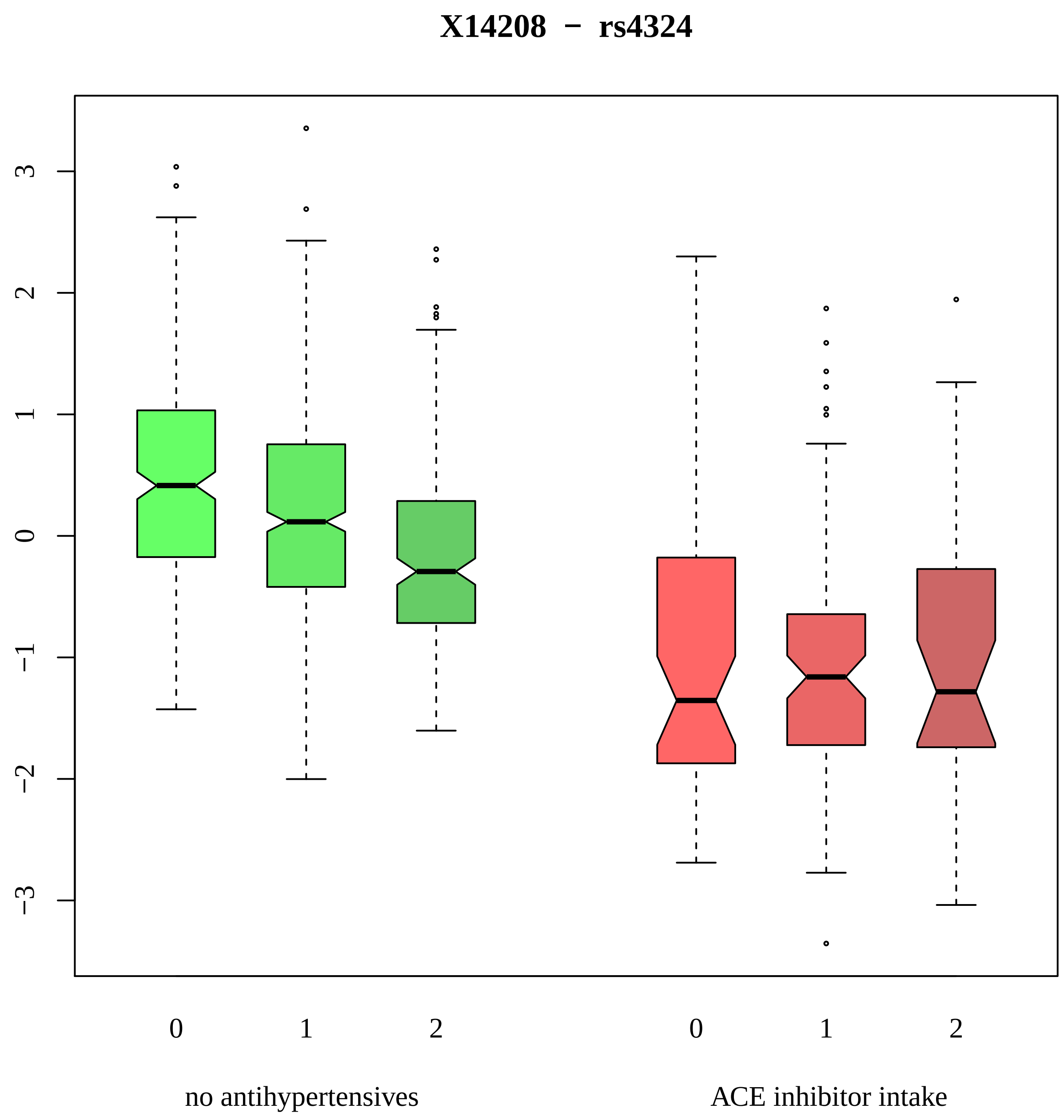
X14208 – rs4324
3
2
1
0
−1
−2
−3
0
1
2
0
1
2
no antihypertensives
ACE inhibitor intake

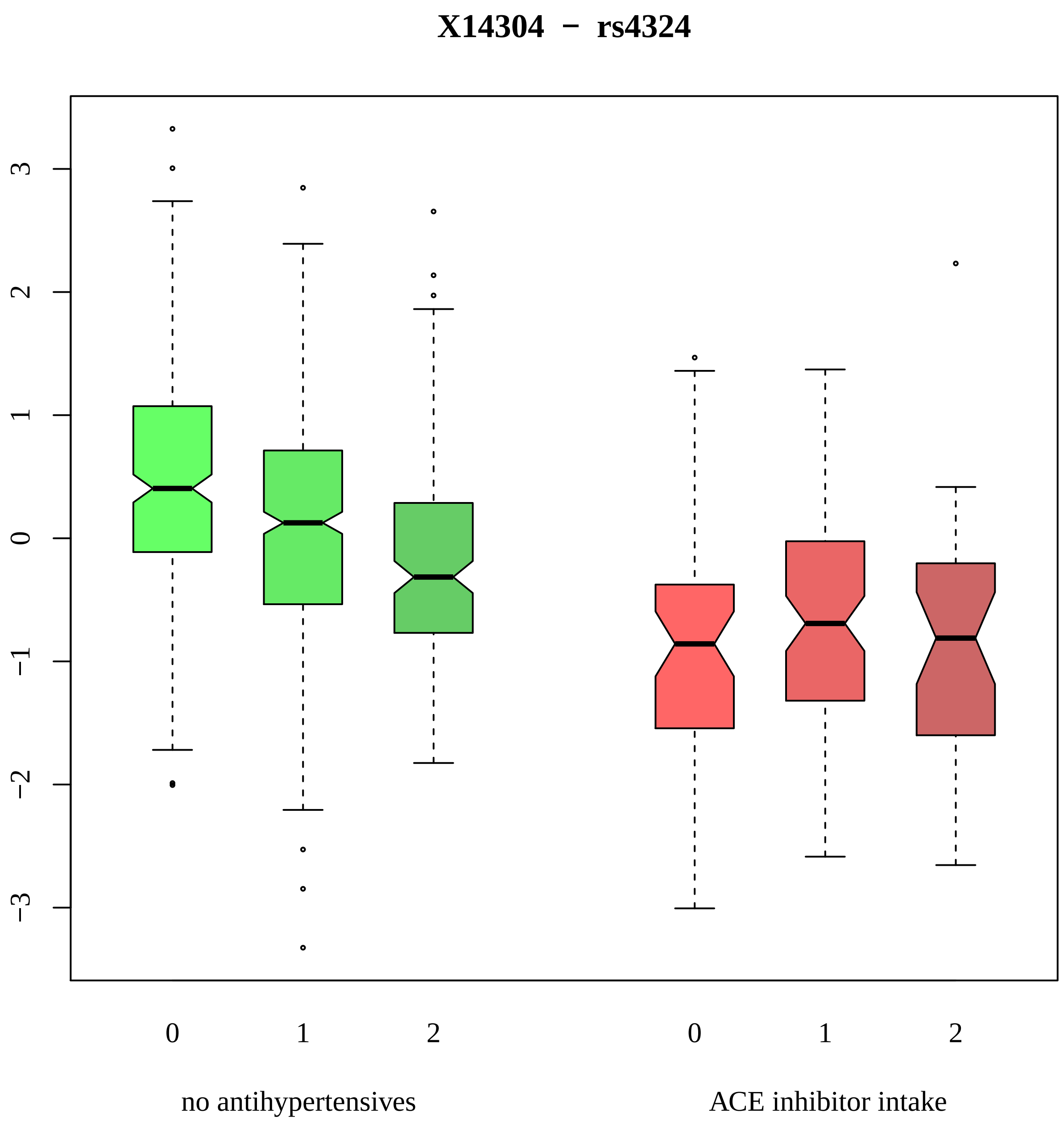

X14304 – rs4324
3
2
1
0
−1
−2
−3
0
1
2
0
1
2
no antihypertensives
ACE inhibitor intake

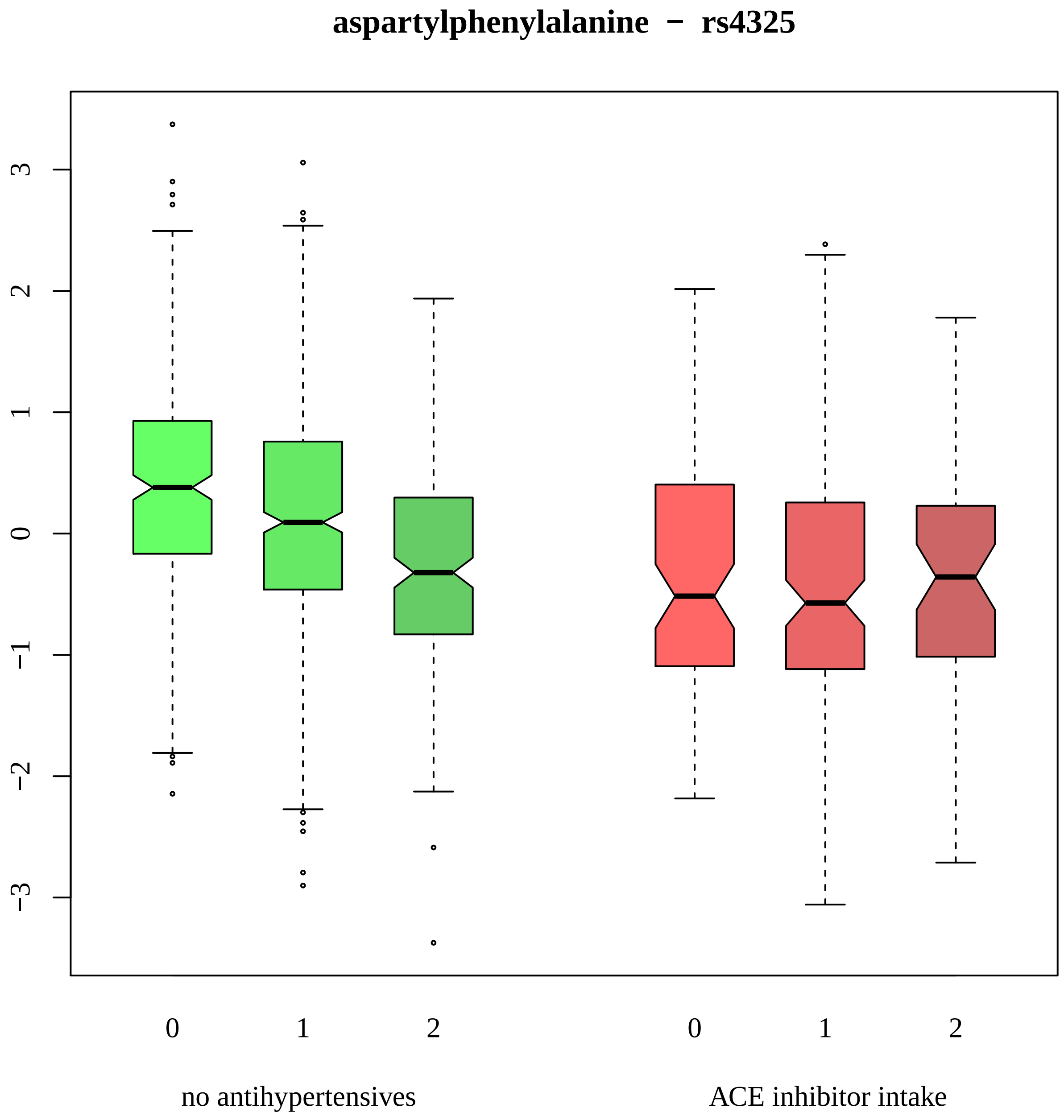

aspartylphenylalanine − rs4325
3
2
1
0
−1
−2
−3
0
1
2
0
1
2
no antihypertensives
ACE inhibitor intake

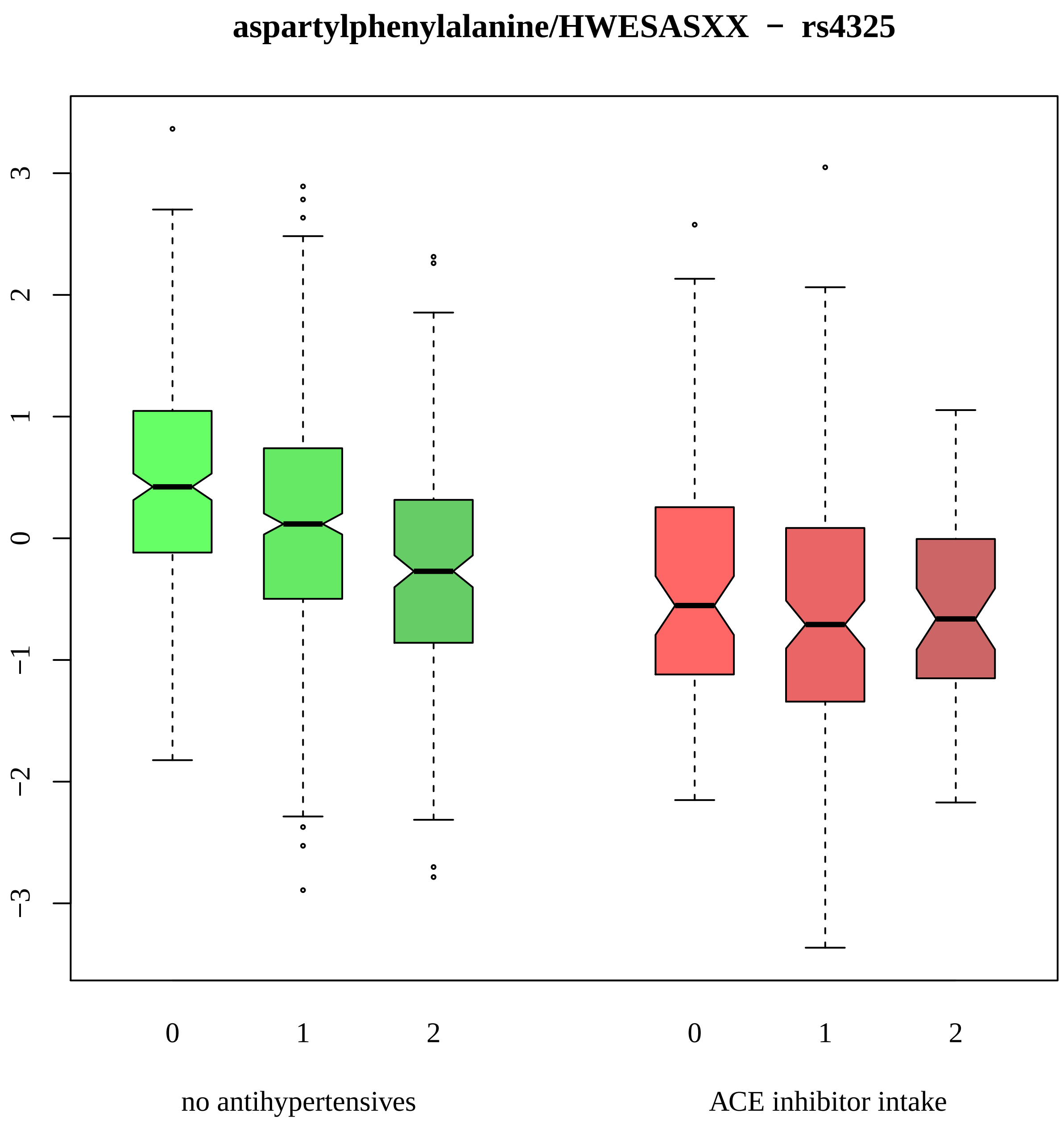
aspartylphenylalanine/HWESASXX − rs4325
3
2
1
0
−1
−2
−3
0
1
2
0
1
2
no antihypertensives
ACE inhibitor intake

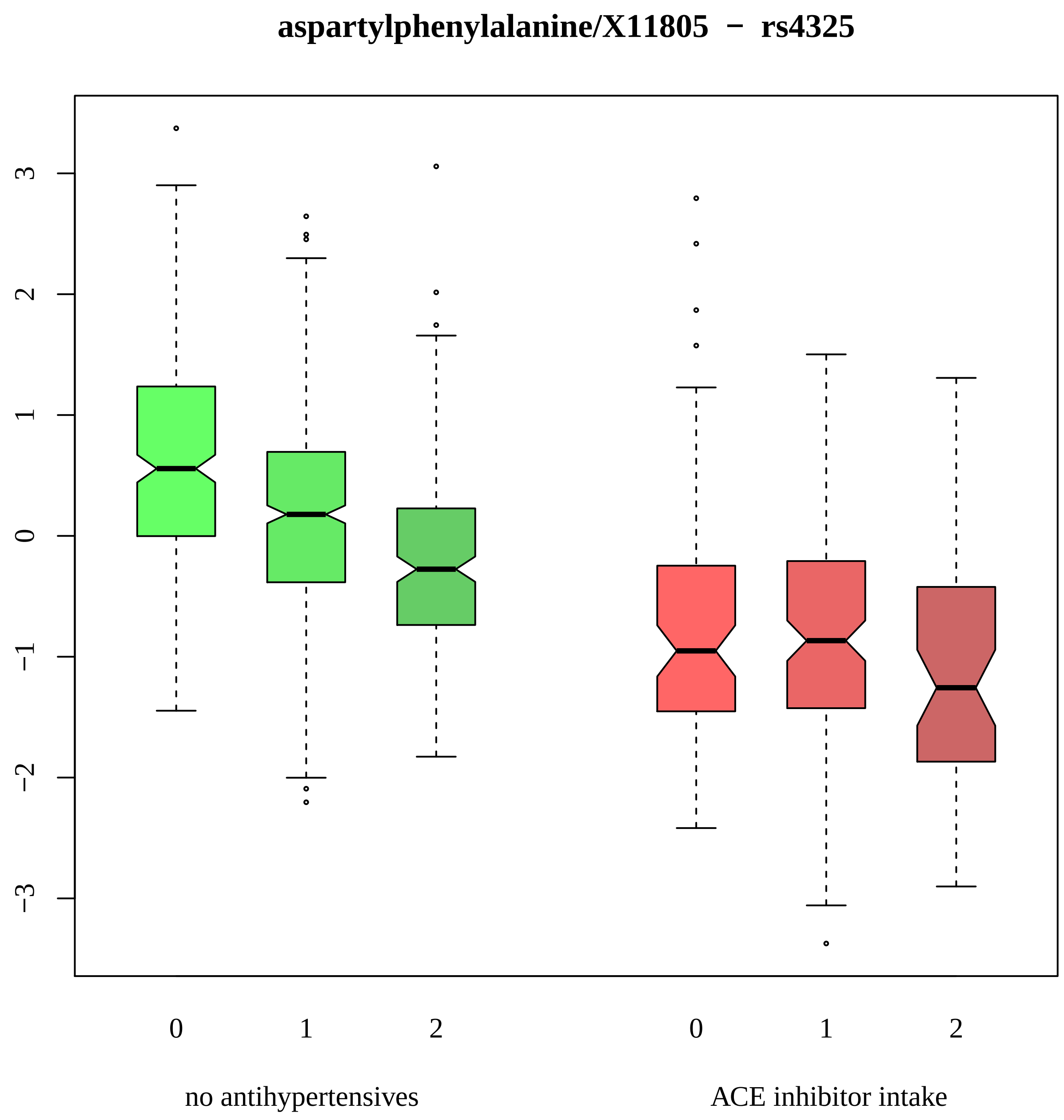
aspartylphenylalanine/X11805 − rs4325
3
2
1
0
−1
−2
−3
0
1
2
0
1
2
no antihypertensives
ACE inhibitor intake

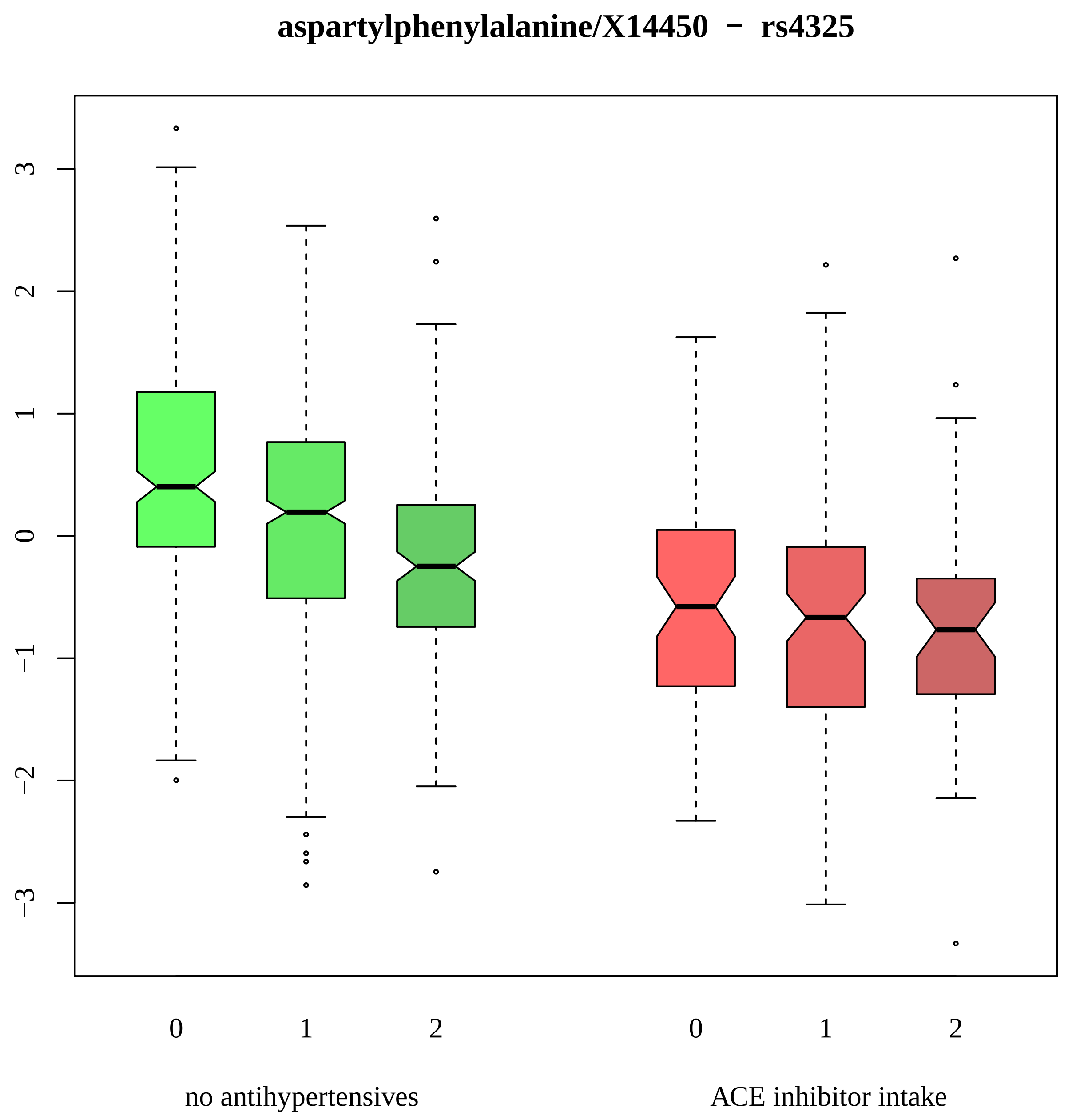

aspartylphenylalanine/X14450 – rs4325
3
2
1
0
−1
−2
−3
0
1
2
0
1
2
no antihypertensives
ACE inhibitor intake

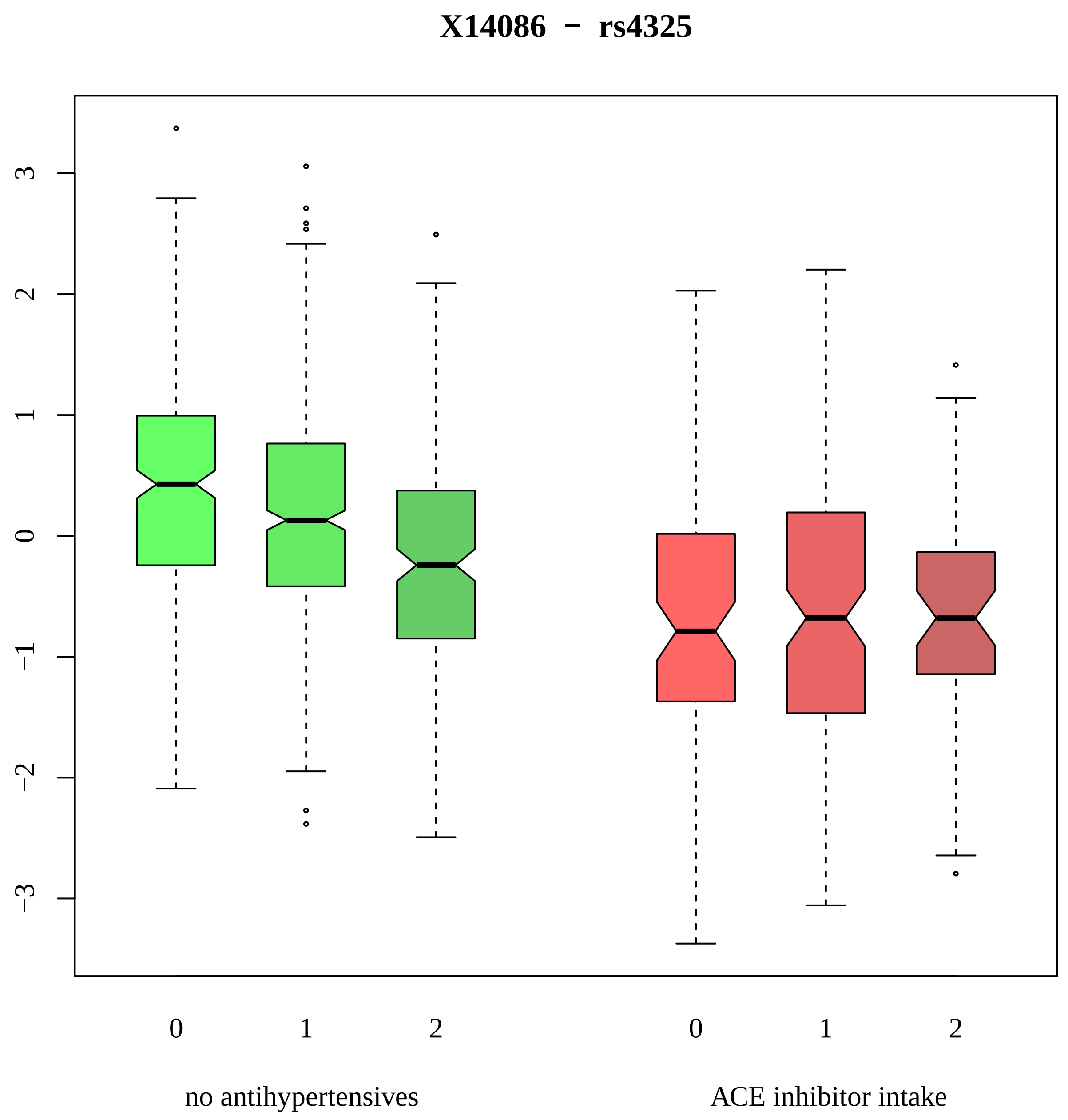

X14086 – rs4325
3
2
1
0
−1
−2
−3
0
1
2
0
1
2
no antihypertensives
ACE inhibitor intake

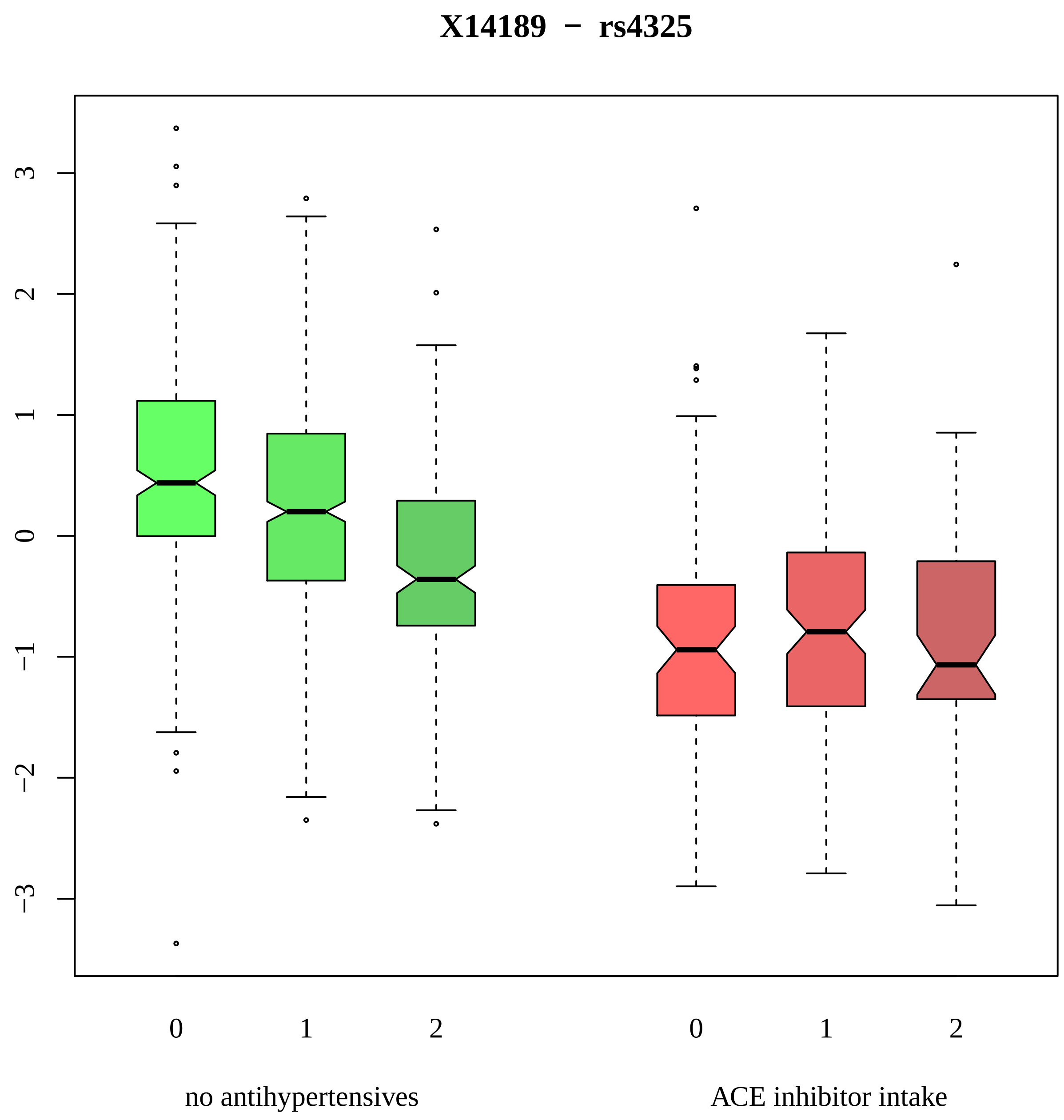

X14189 – rs4325
3
2
1
0
−1
−2
−3
0
1
2
0
1
2
no antihypertensives
ACE inhibitor intake

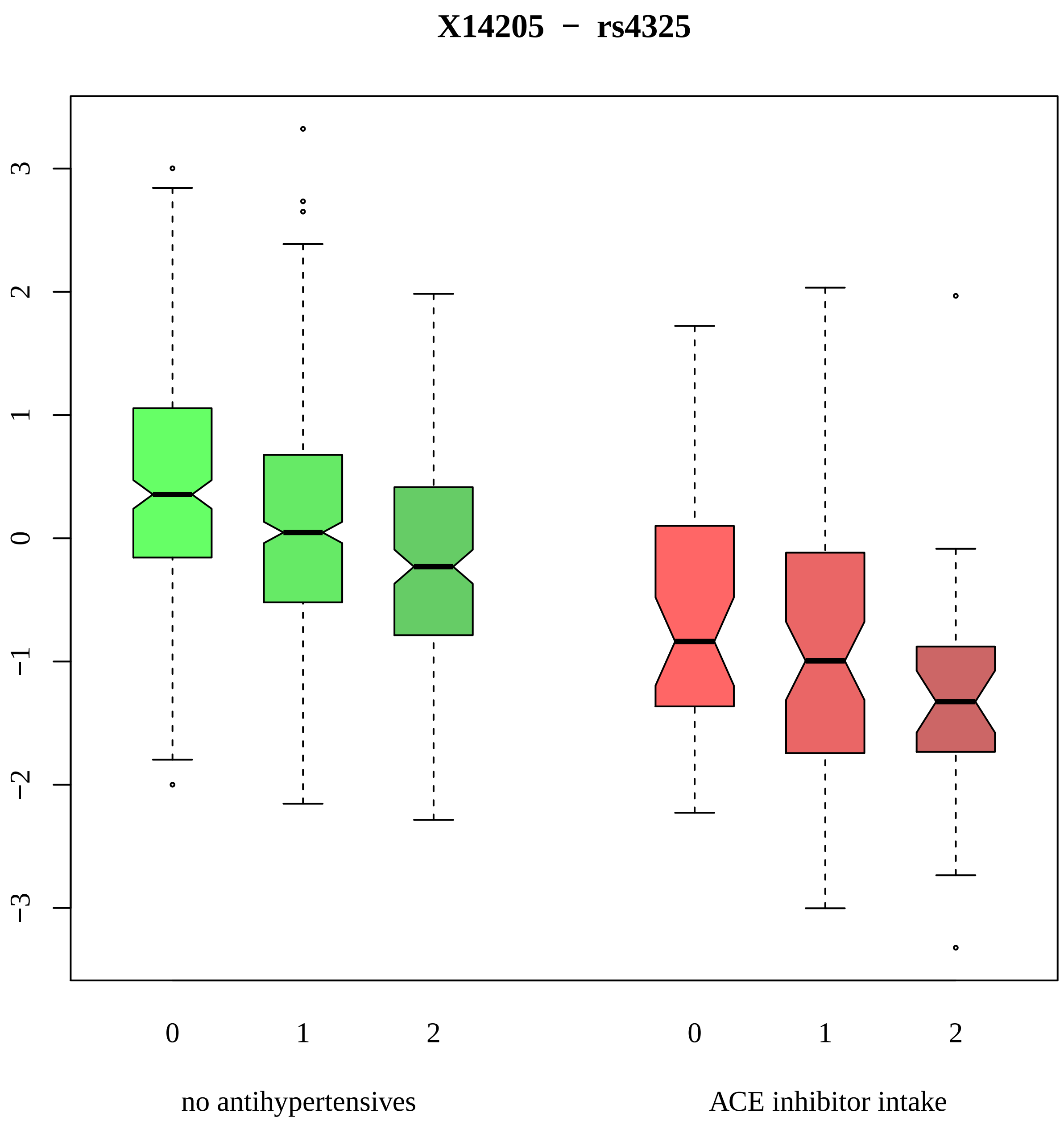

X14205 – rs4325
3
2
1
0
−1
−2
−3
0
1
2
0
1
2
no antihypertensives
ACE inhibitor intake

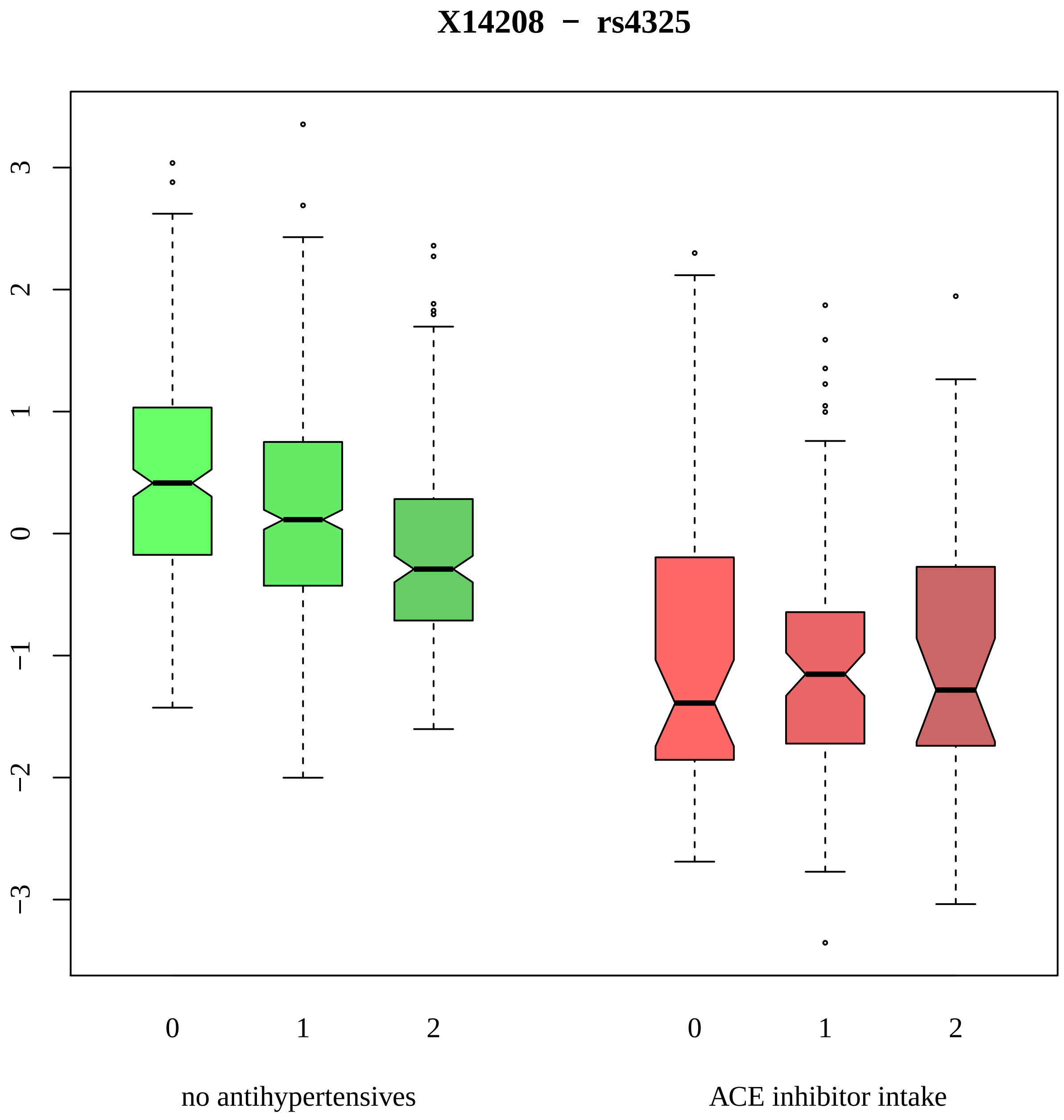

X14208 – rs4325
3
2
1
0
−1
−2
−3
0
1
2
0
1
2
no antihypertensives
ACE inhibitor intake

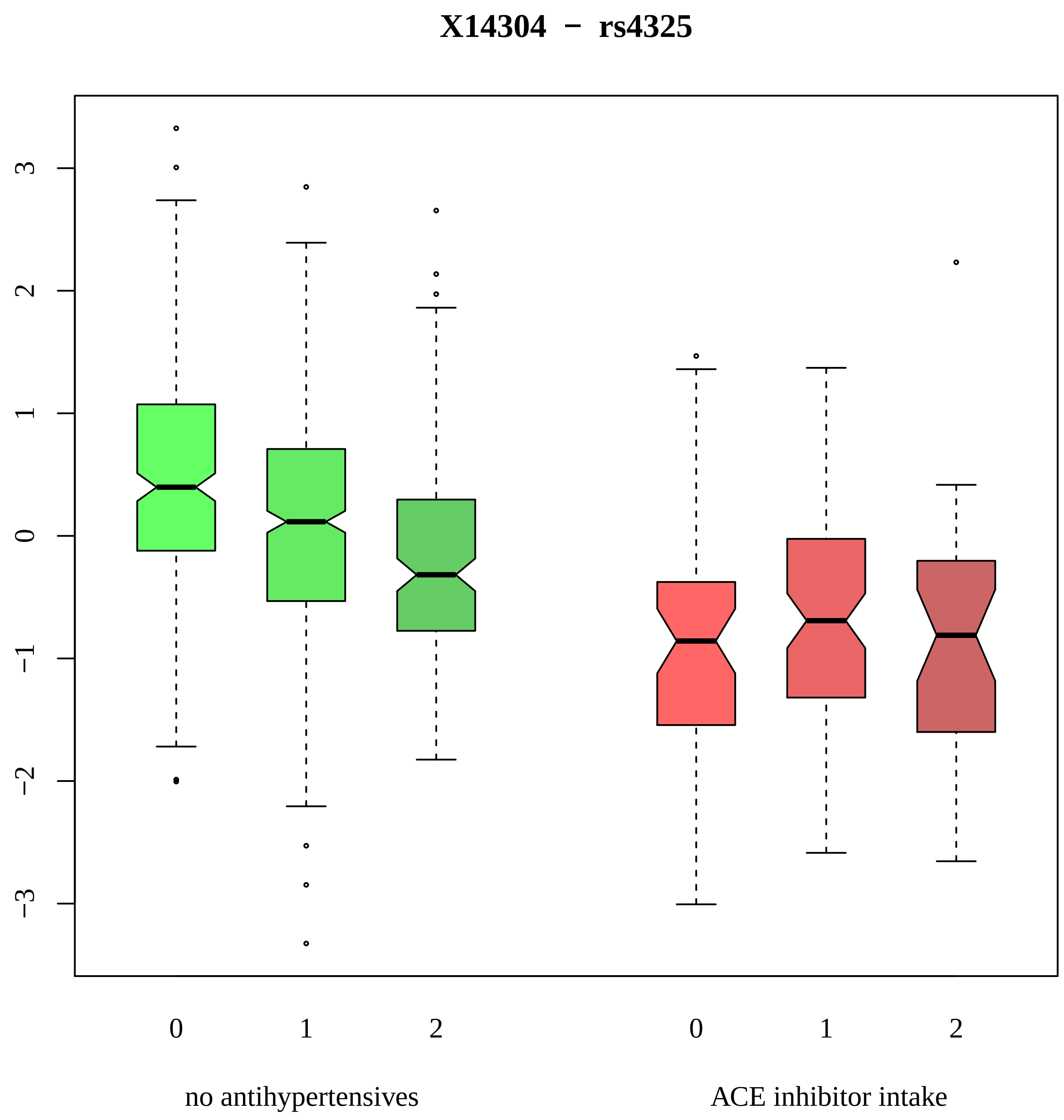

X14304 – rs4325
3
2
1
0
−1
−2
−3
0
1
2
0
1
2
no antihypertensives
ACE inhibitor intake

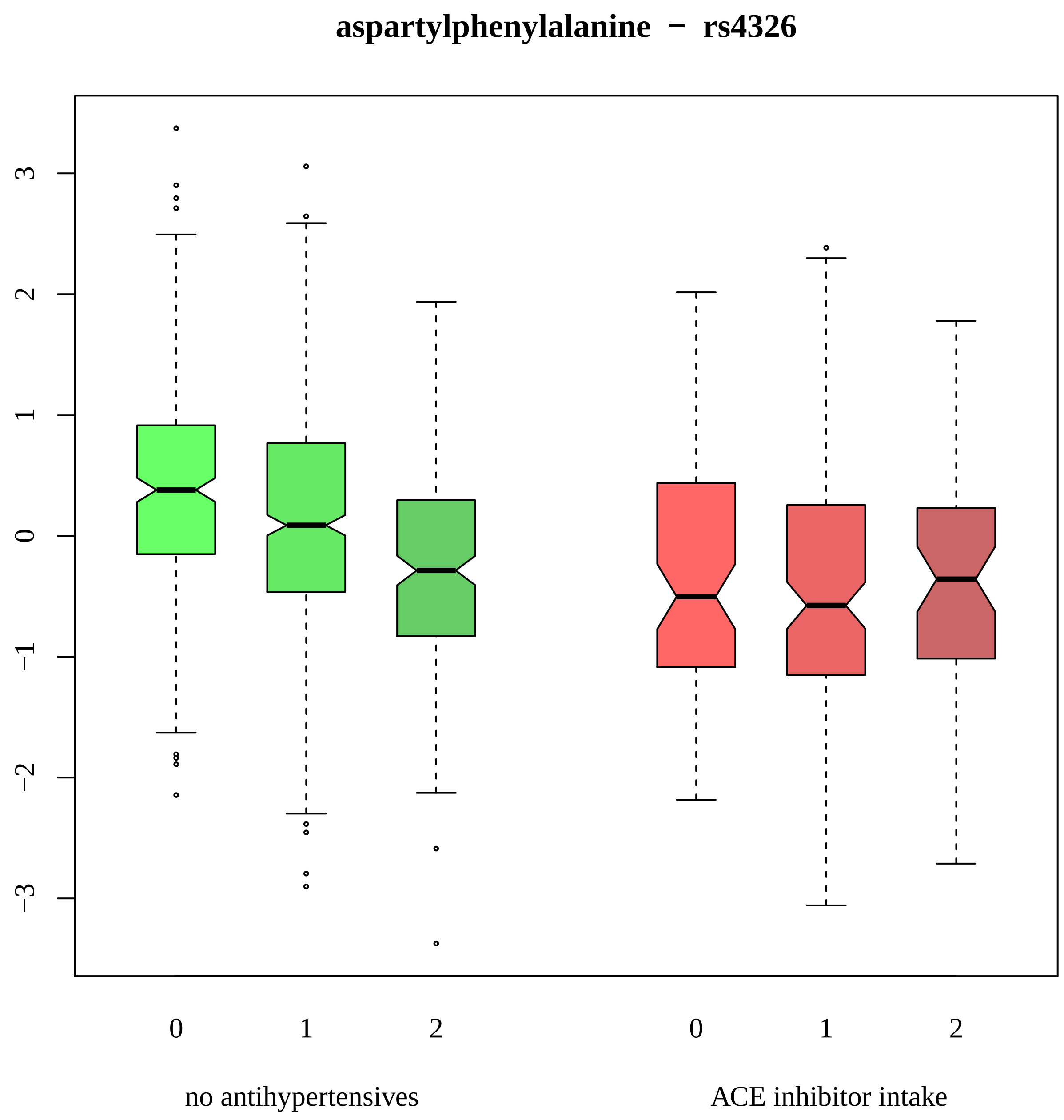

aspartylphenylalanine - rs4326
3
2
1
0
−1
−2
−3
0
1
2
0
1
2
no antihypertensives
ACE inhibitor intake

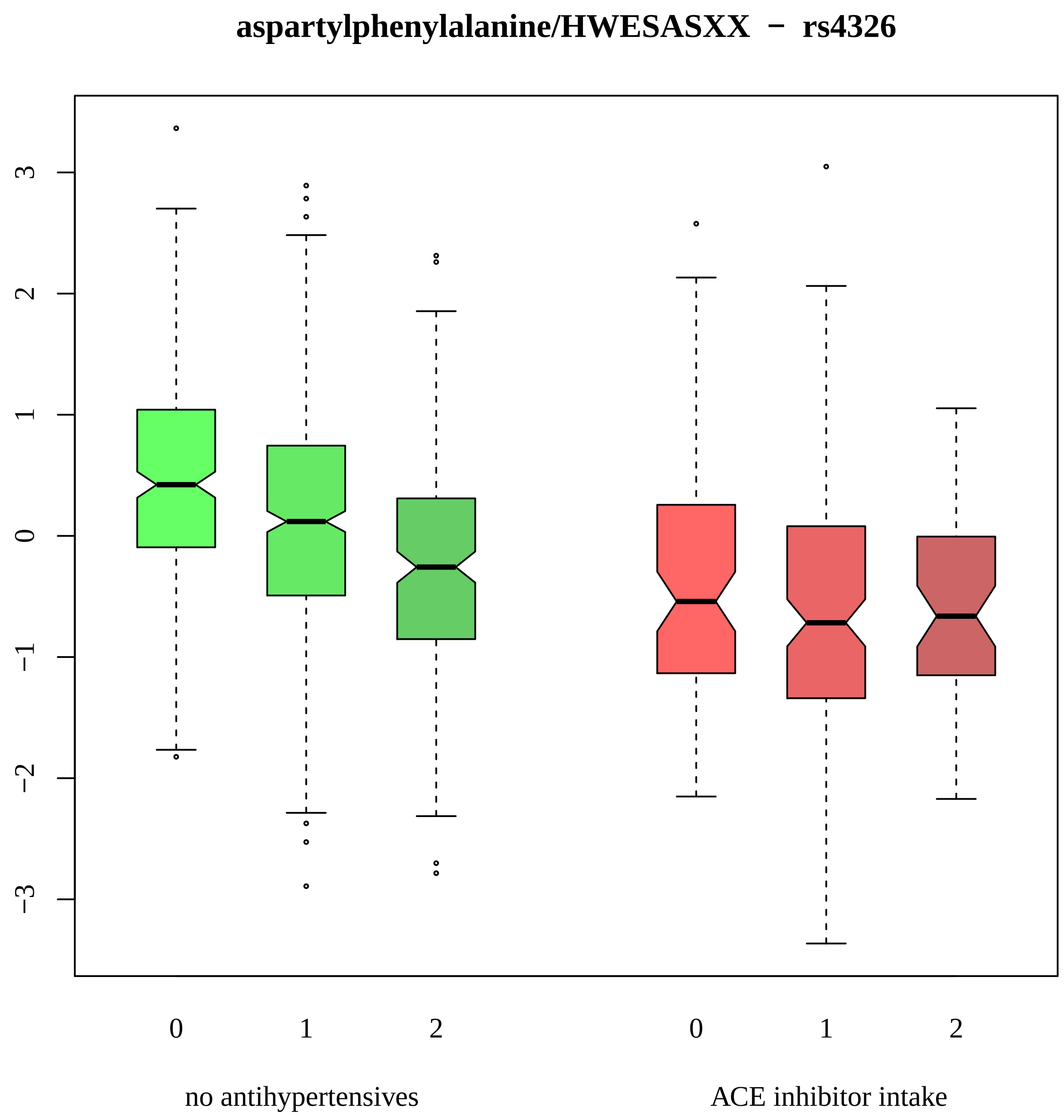
aspartylphenylalanine/HWESASXX – rs4326
3
2
1
0
−1
−2
−3
0
1
2
0
1
2
no antihypertensives
ACE inhibitor intake

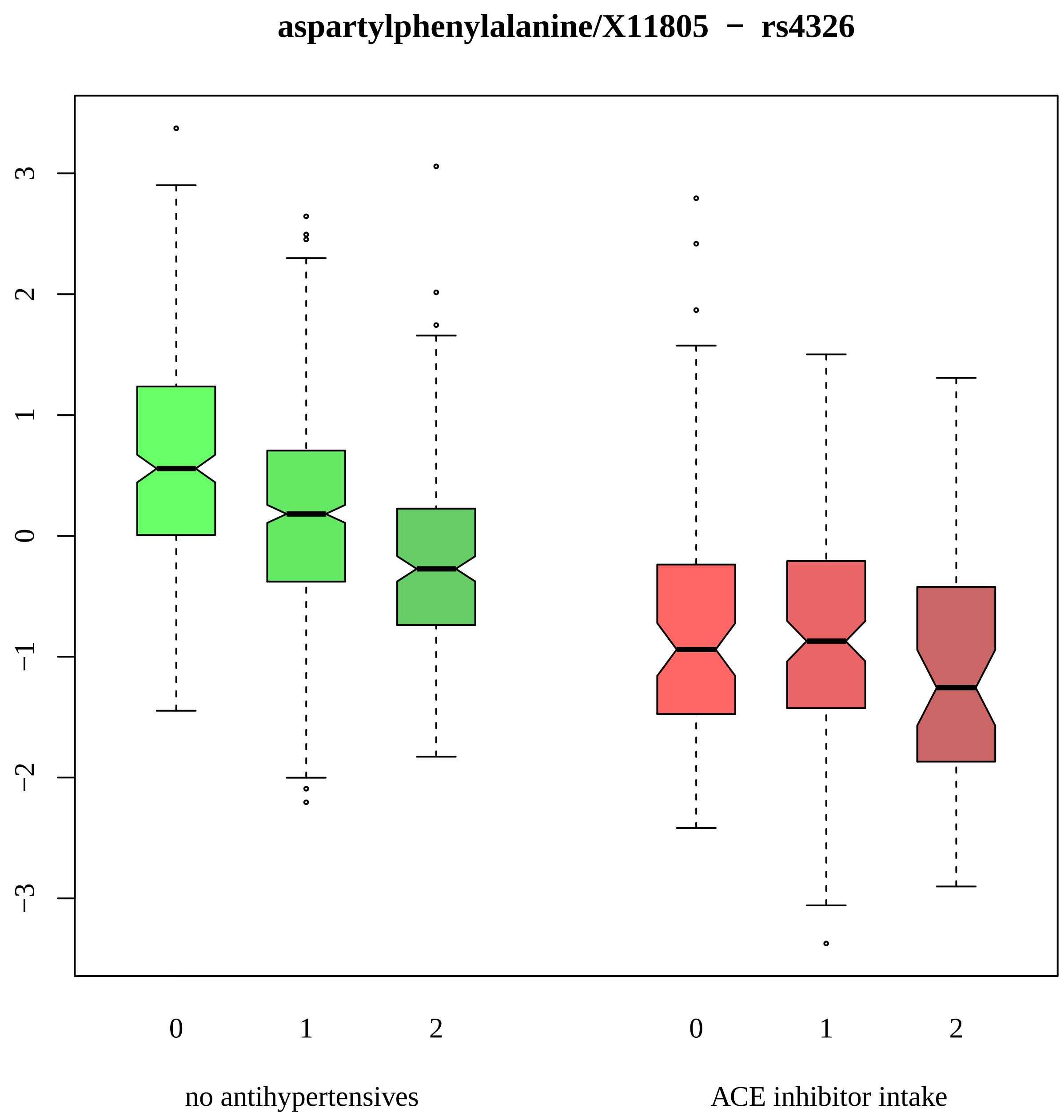
aspartylphenylalanine/X11805 – rs4326
3
2
1
0
−1
−2
−3
0
1
2
0
1
2
no antihypertensives
ACE inhibitor intake

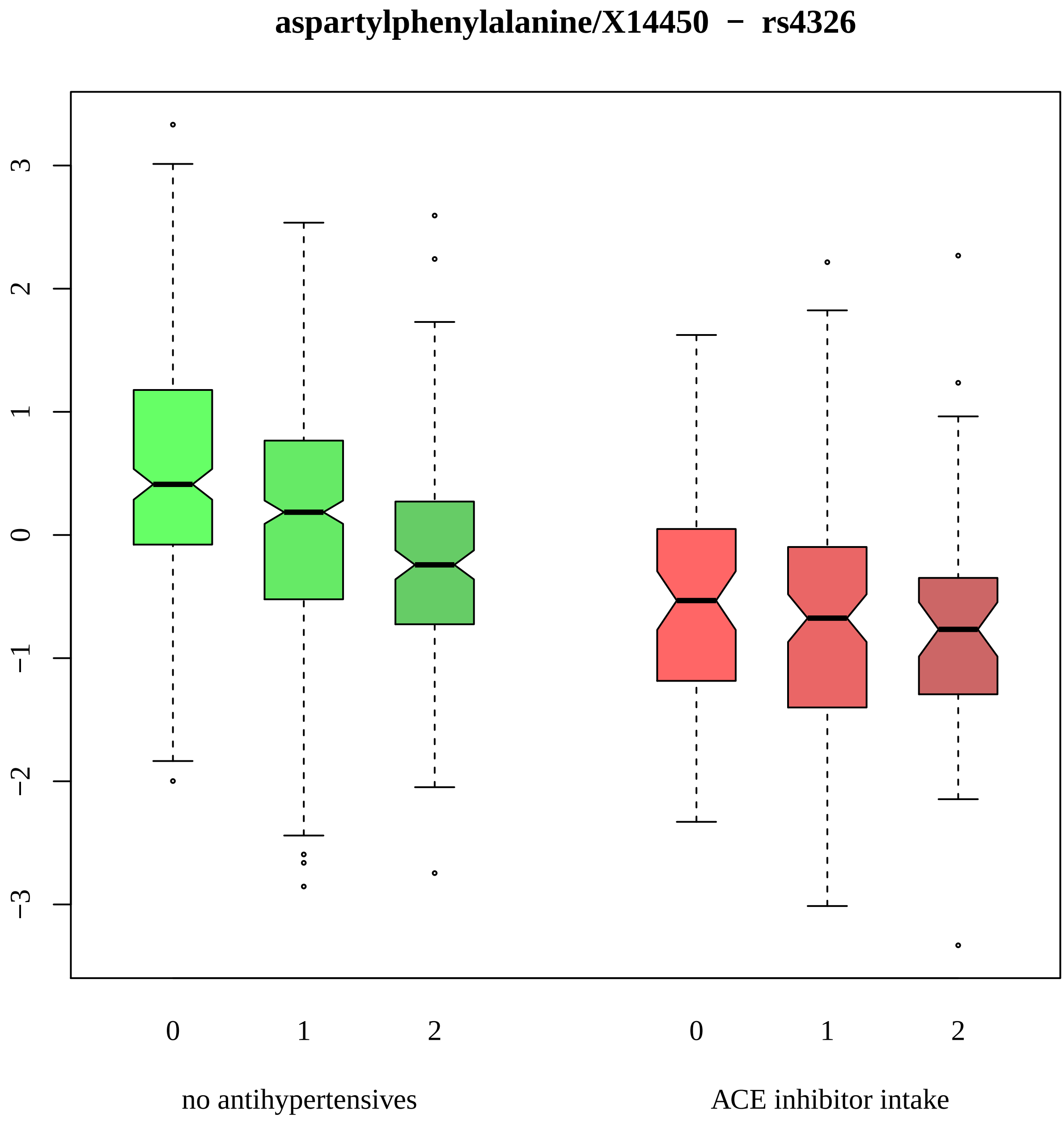

aspartylphenylalanine/X14450 – rs4326
3
2
1
0
−1
−2
−3
0
1
2
0
1
2
no antihypertensives
ACE inhibitor intake

X14086 – rs4326

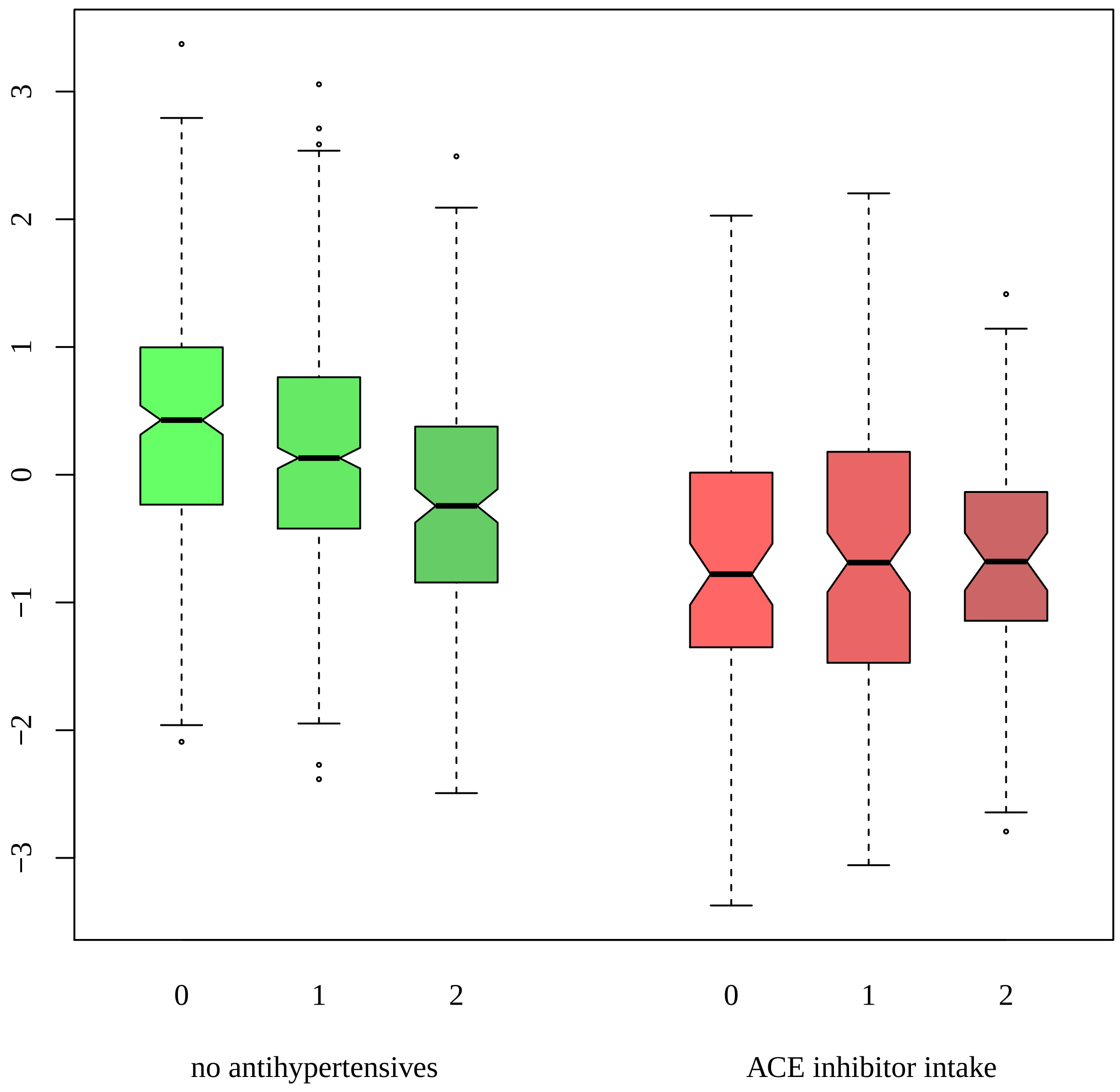

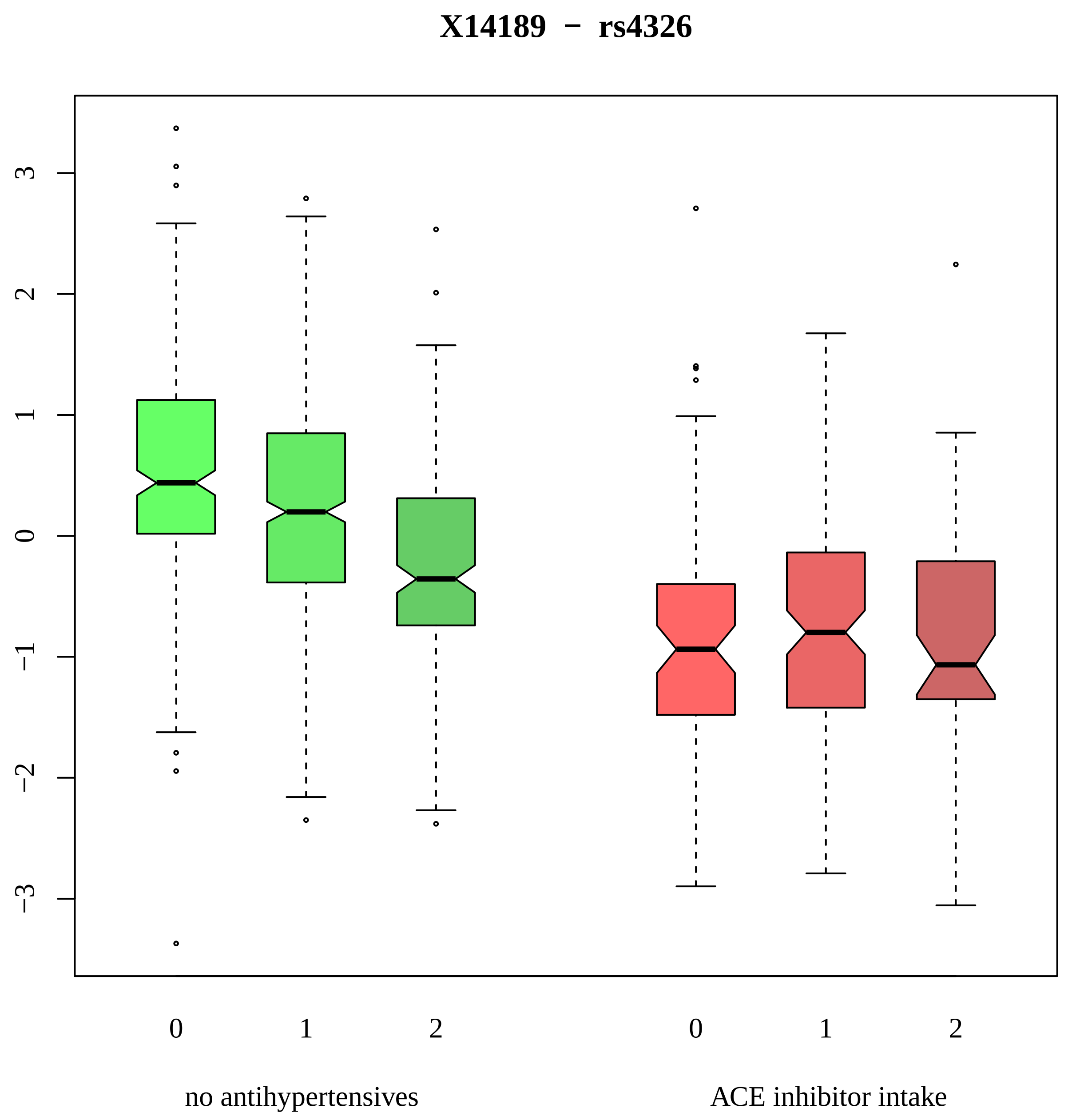
X14189 – rs4326
3
2
1
0
−1
−2
−3
0
1
2
0
1
2
no antihypertensives
ACE inhibitor intake

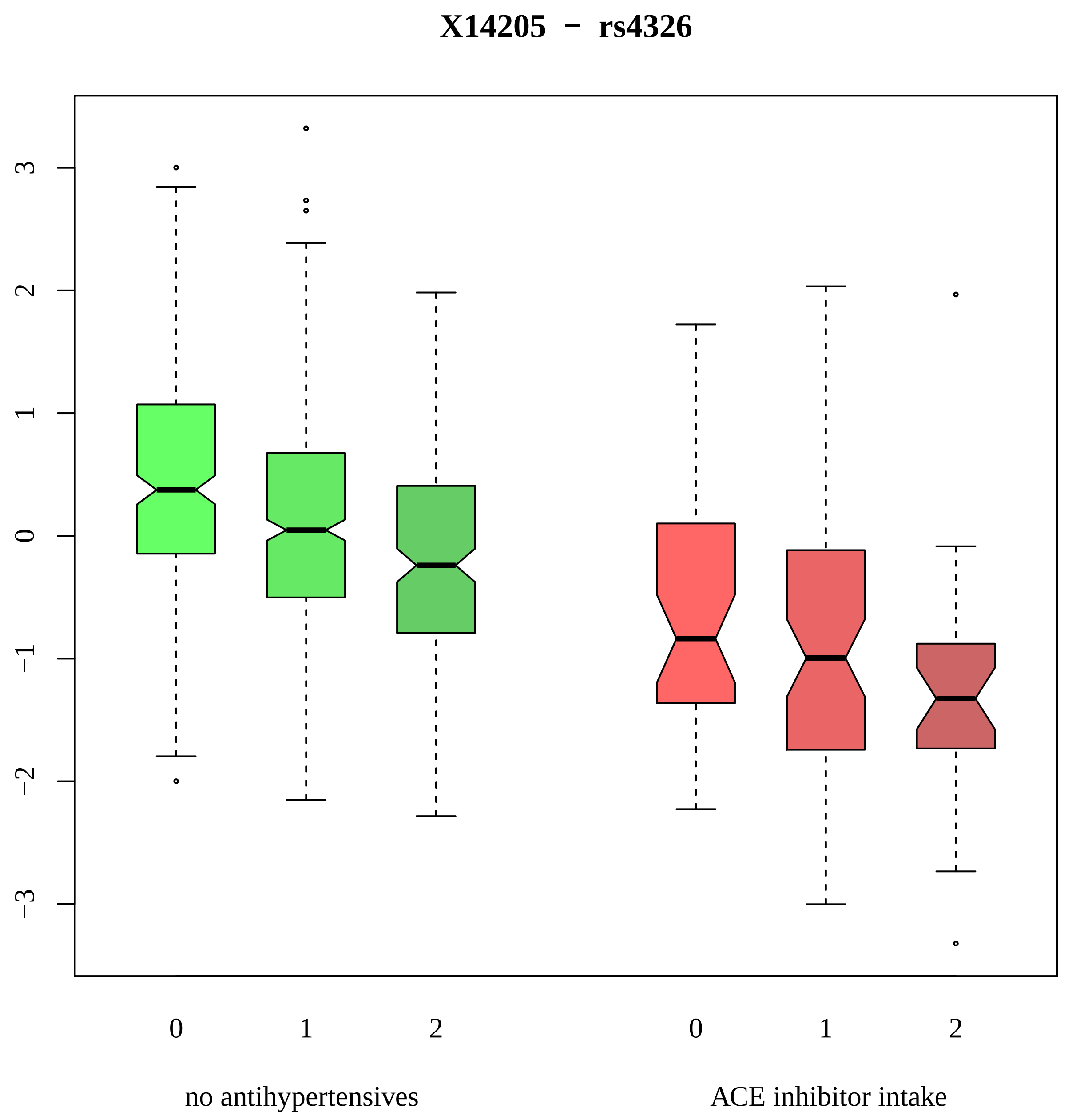

X14205 – rs4326
3
2
1
0
−1
−2
−3
0
1
2
0
1
2
no antihypertensives
ACE inhibitor intake

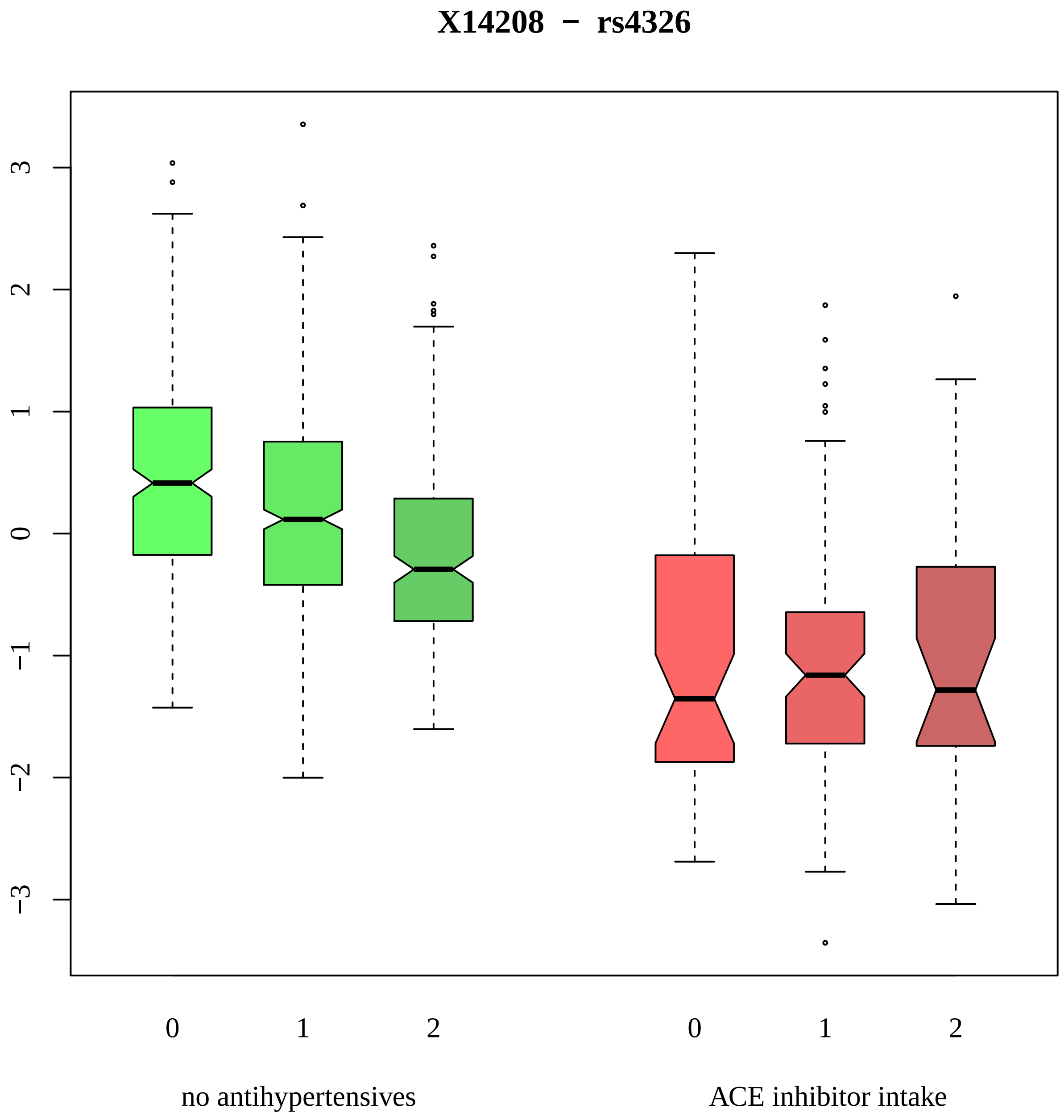

X14208 – rs4326
3
2
1
0
−1
−2
−3
0
1
2
0
1
2
no antihypertensives
ACE inhibitor intake

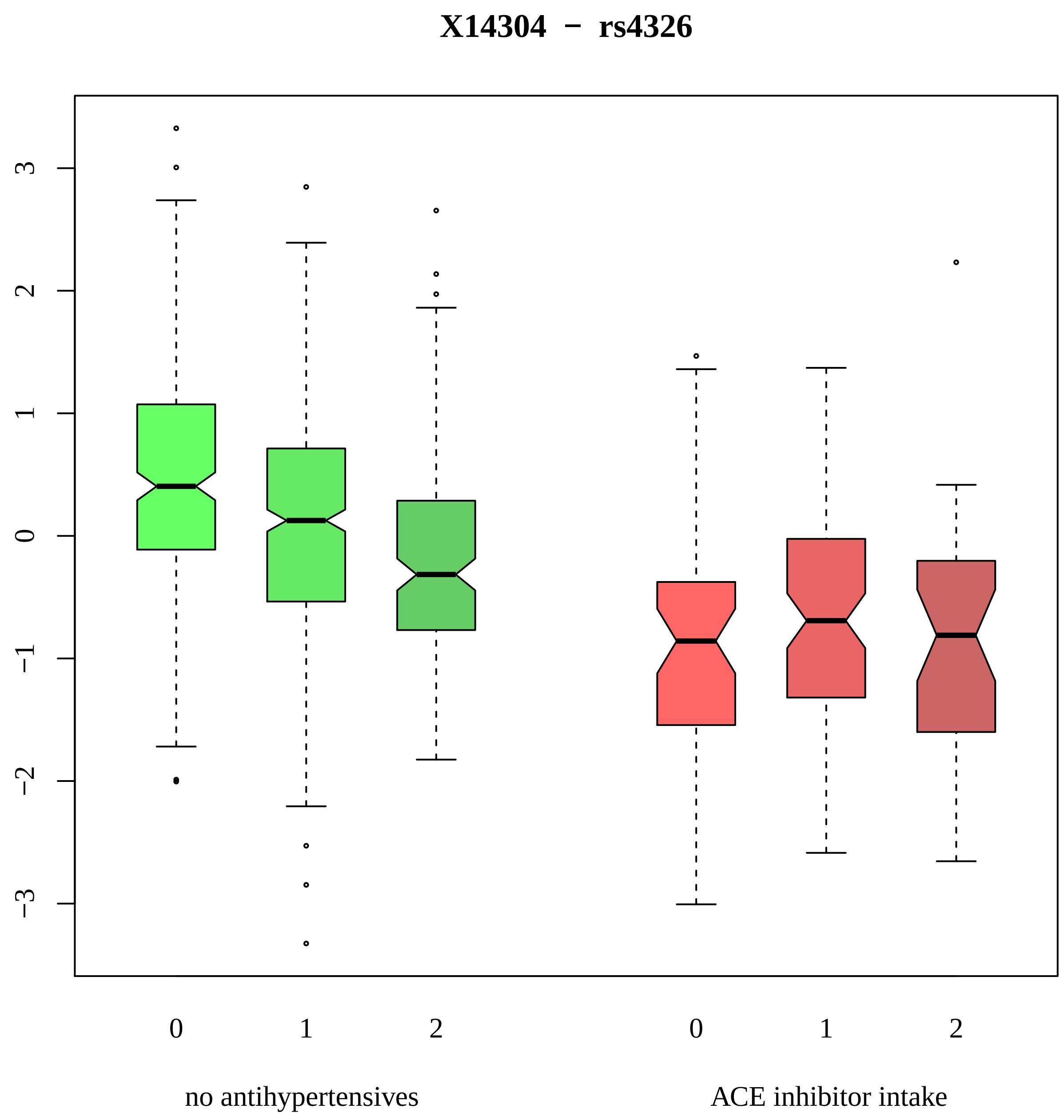
X14304 – rs4326
3
2
1
0
−1
−2
−3
0
1
2
0
1
2
no antihypertensives
ACE inhibitor intake

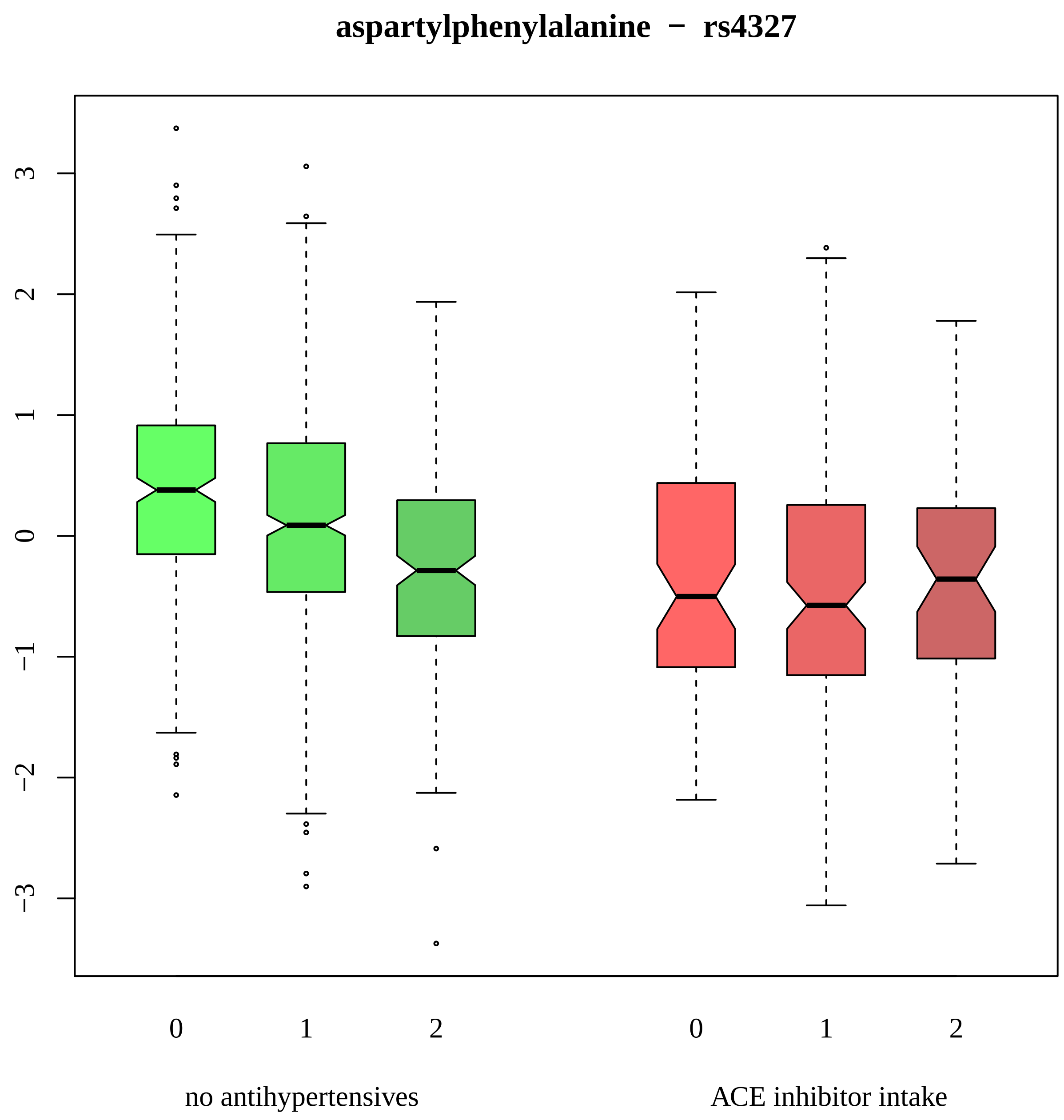
aspartylphenylalanine – rs4327
3
2
1
0
−1
−2
−3
0
1
2
0
1
2
no antihypertensives
ACE inhibitor intake

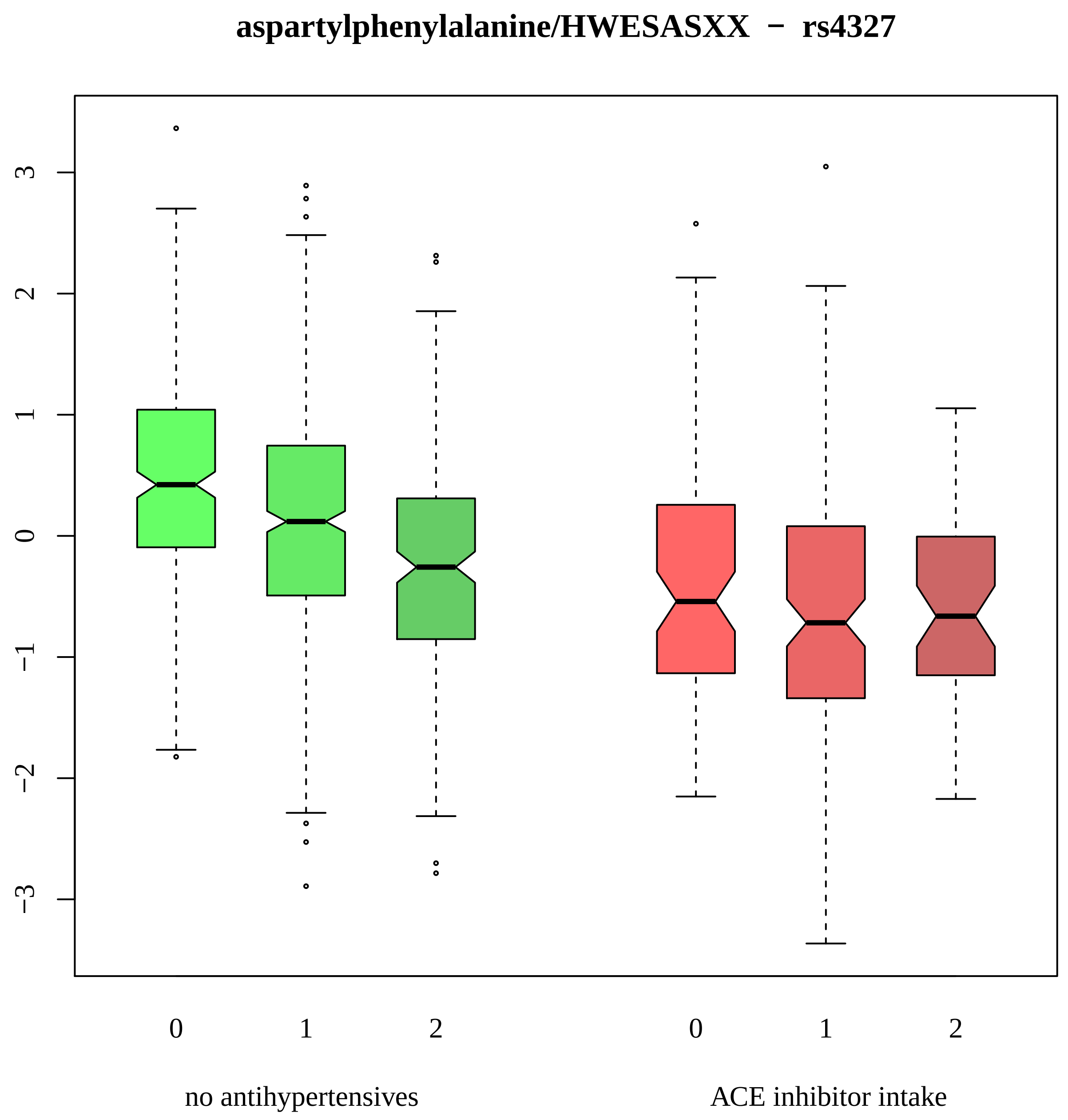

aspartylphenylalanine/HWESASXX – rs4327
3
2
1
0
−1
−2
−3
0
1
2
0
1
2
no antihypertensives
ACE inhibitor intake

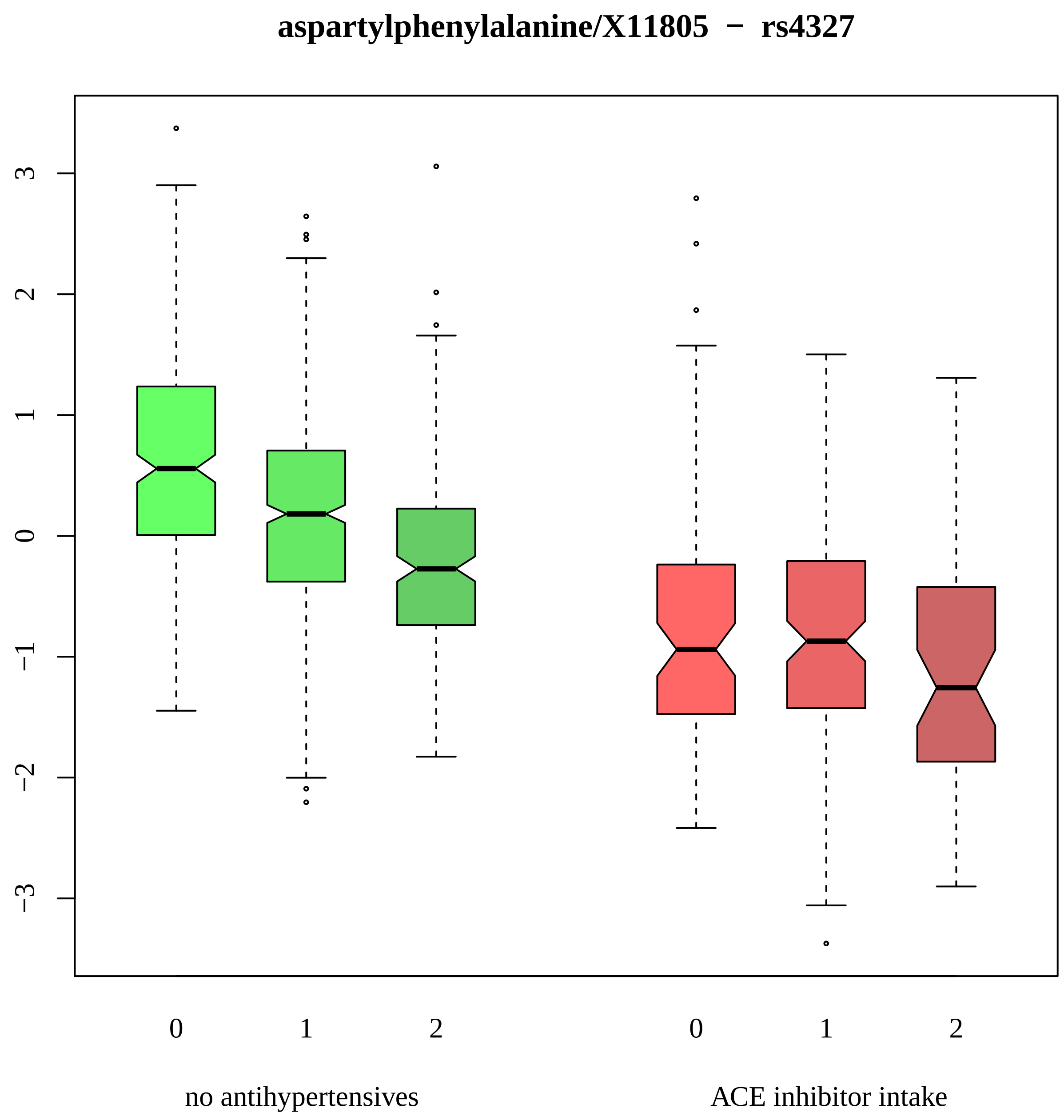

aspartylphenylalanine/X11805 – rs4327
3
2
1
0
−1
−2
−3
0
1
2
0
1
2
no antihypertensives
ACE inhibitor intake

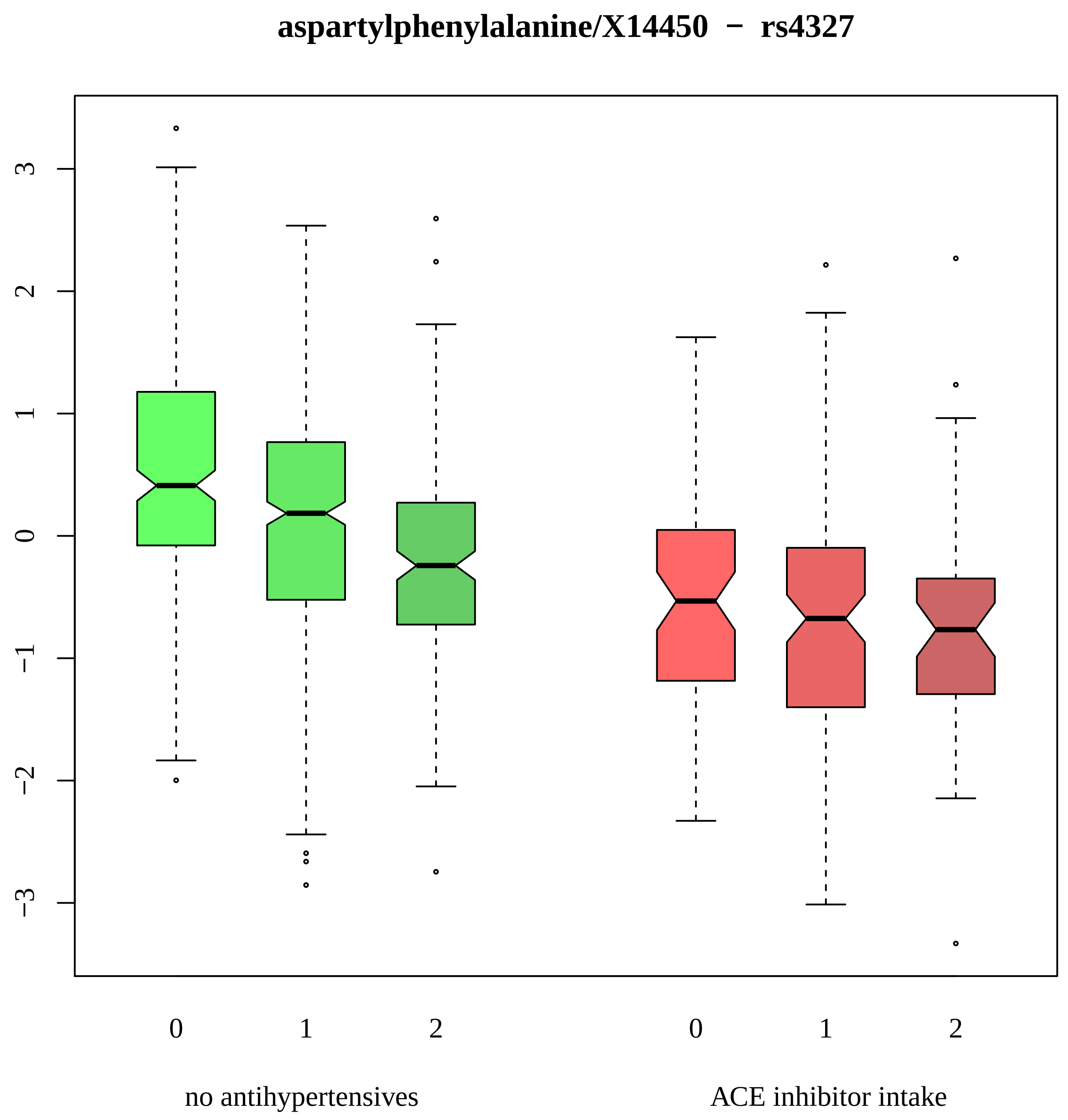
aspartylphenylalanine/X14450 – rs4327
3
2
1
0
−1
−2
−3
0
1
2
0
1
2
no antihypertensives
ACE inhibitor intake

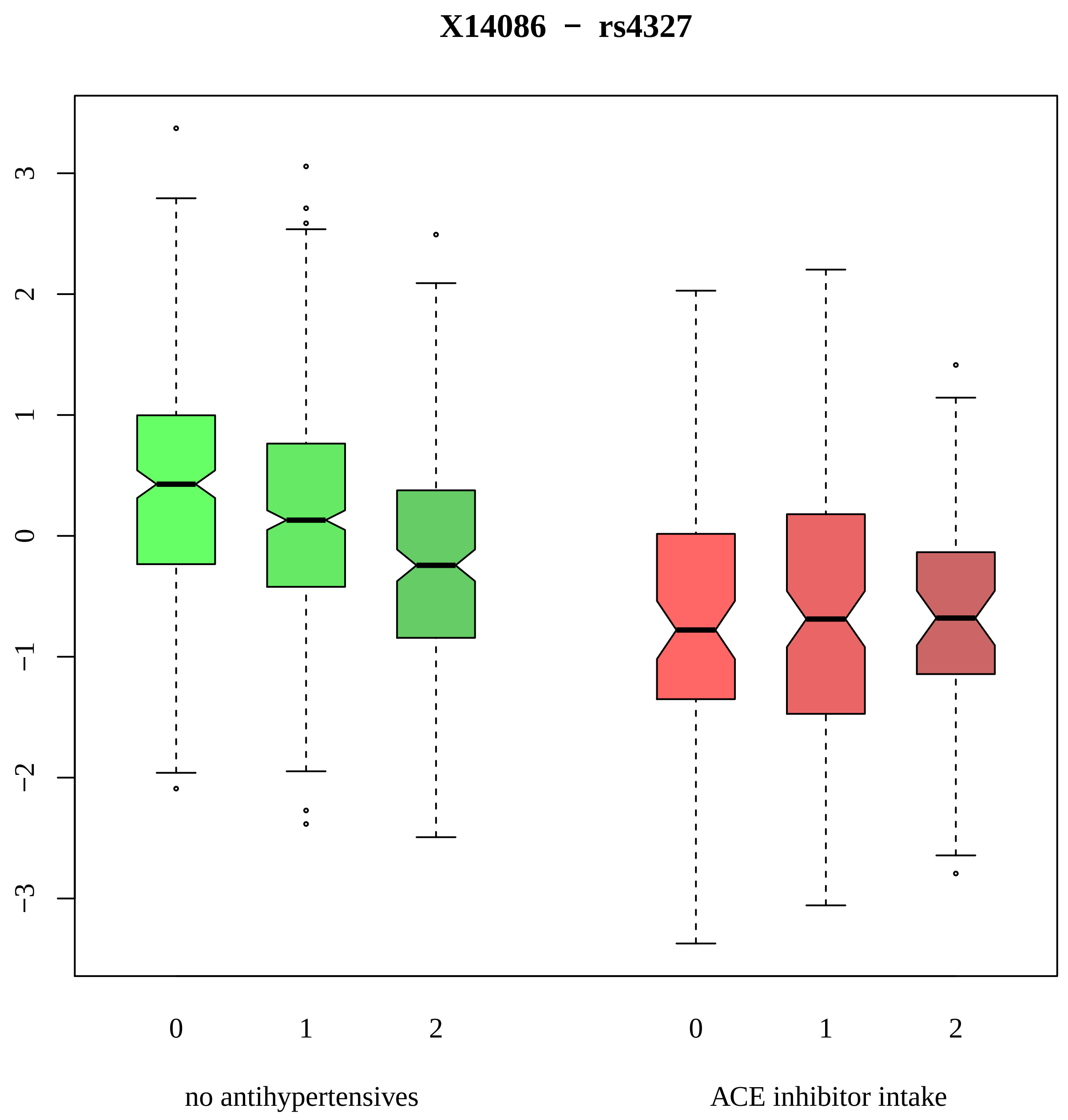

X14086 – rs4327
3
2
1
0
−1
−2
−3
0
1
2
0
1
2
no antihypertensives
ACE inhibitor intake

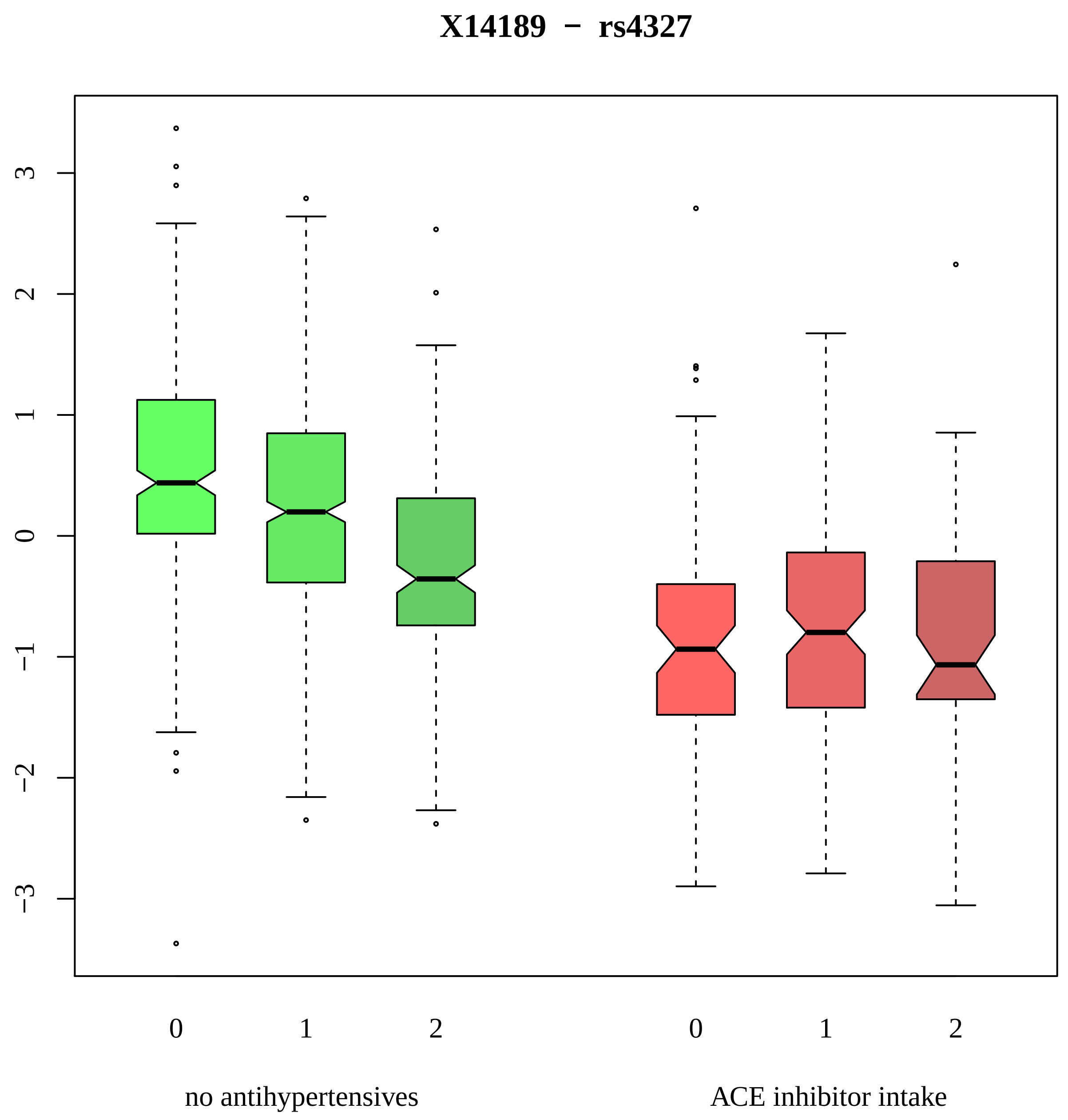

X14189 – rs4327
3
2
1
0
−1
−2
−3
0
1
2
0
1
2
no antihypertensives
ACE inhibitor intake

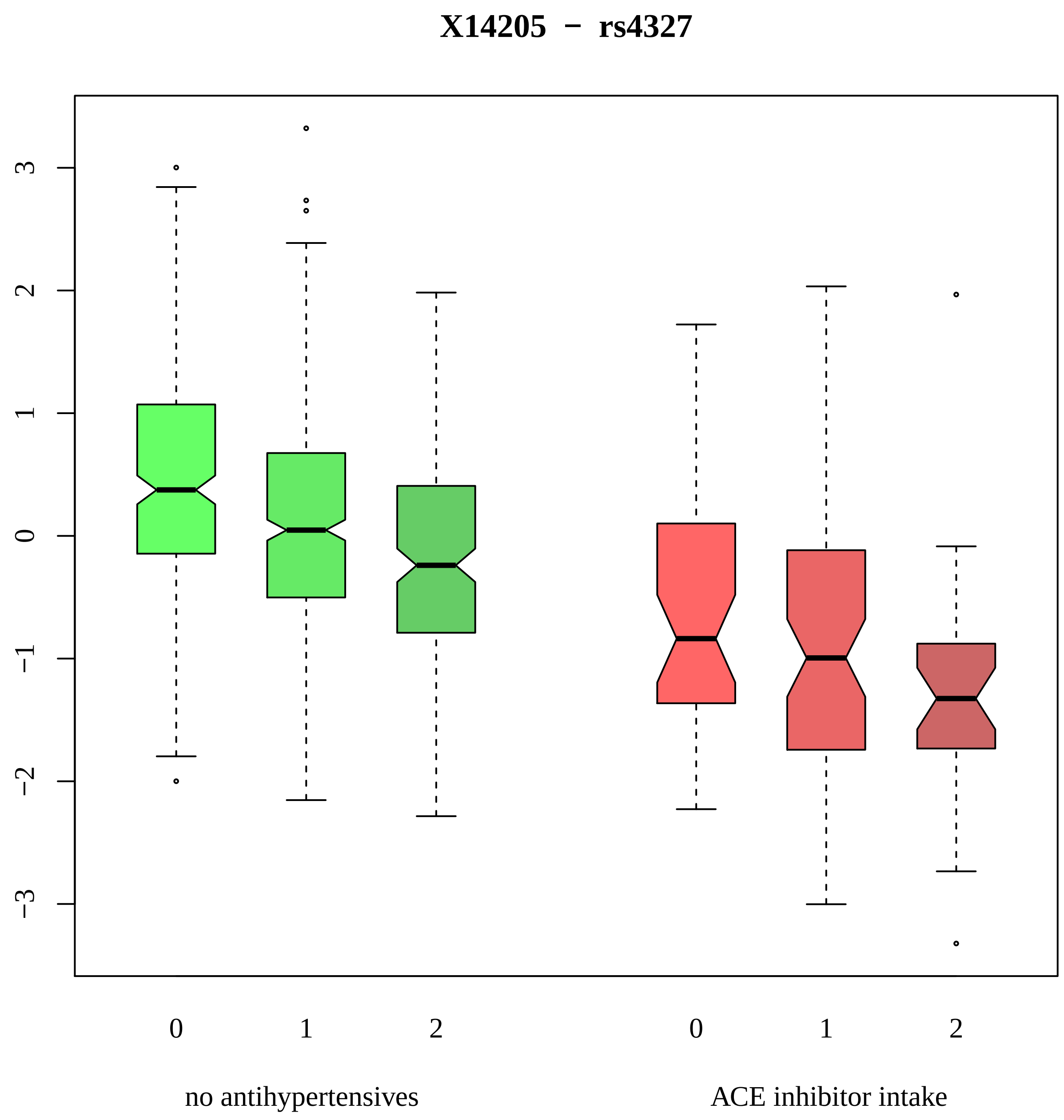
X14205 – rs4327
3
2
1
0
−1
−2
−3
0
1
2
0
1
2
no antihypertensives
ACE inhibitor intake

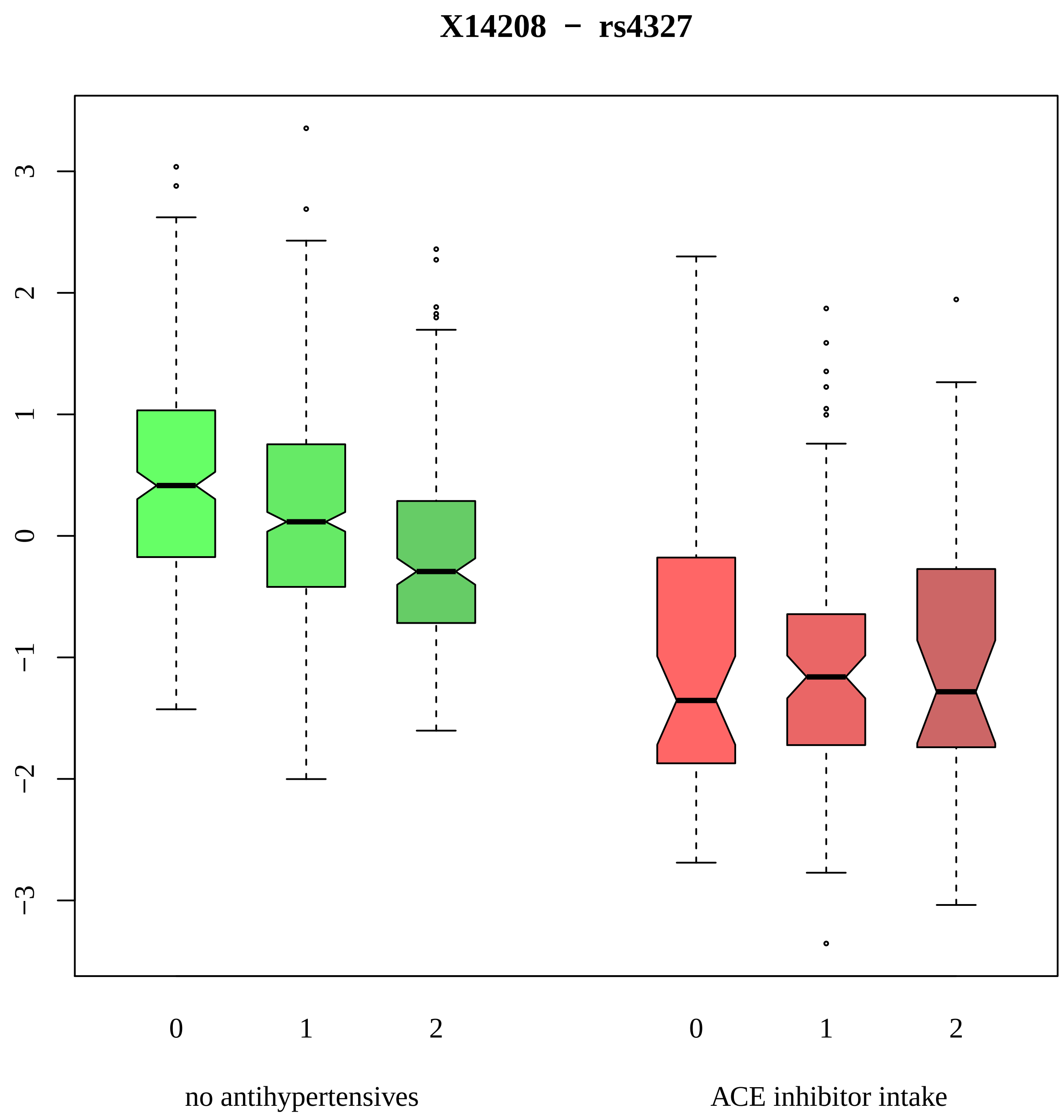
X14208 – rs4327
3
2
1
0
−1
−2
−3
0
1
2
0
1
2
no antihypertensives
ACE inhibitor intake

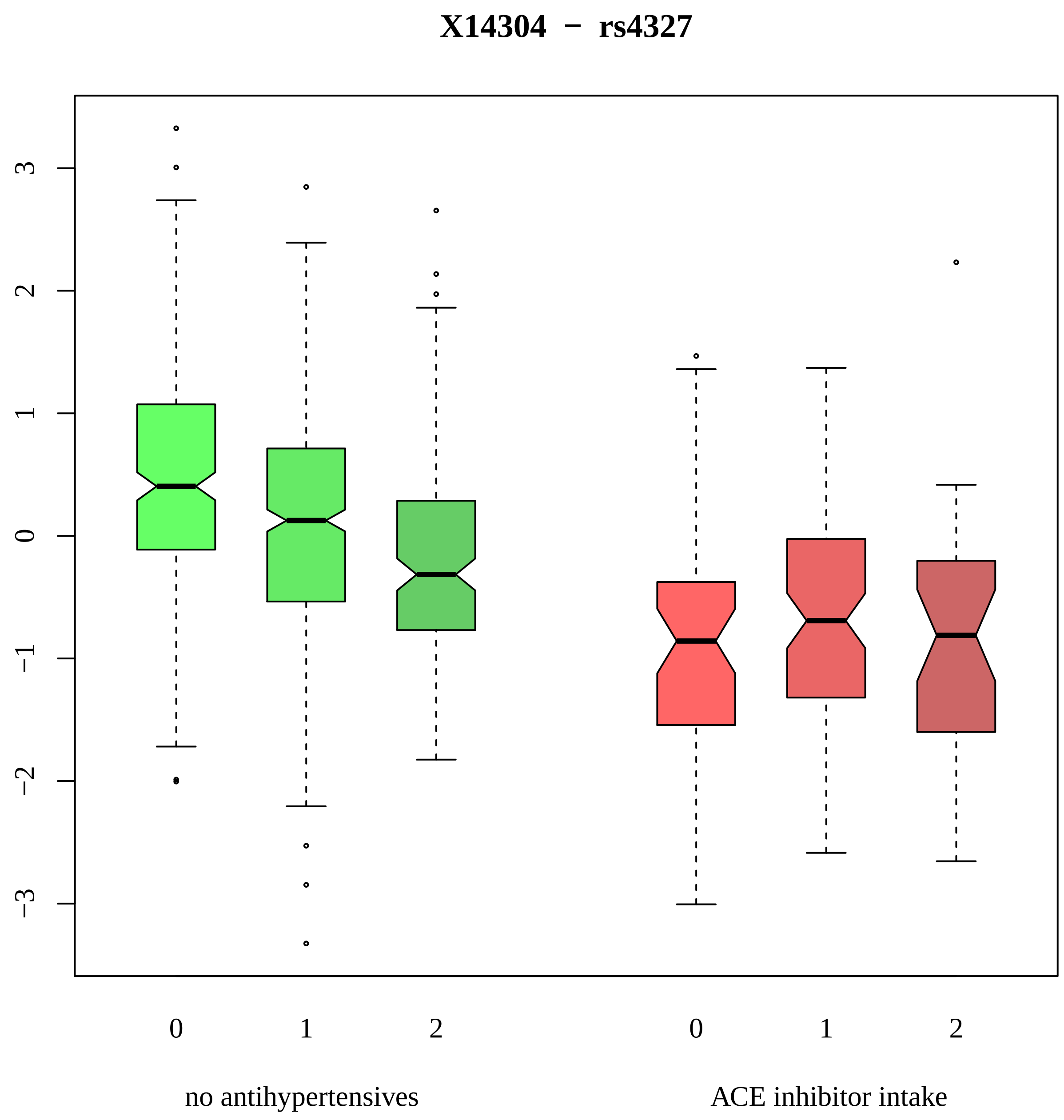
X14304 – rs4327
3
2
1
0
−1
−2
−3
0
1
2
0
1
2
no antihypertensives
ACE inhibitor intake

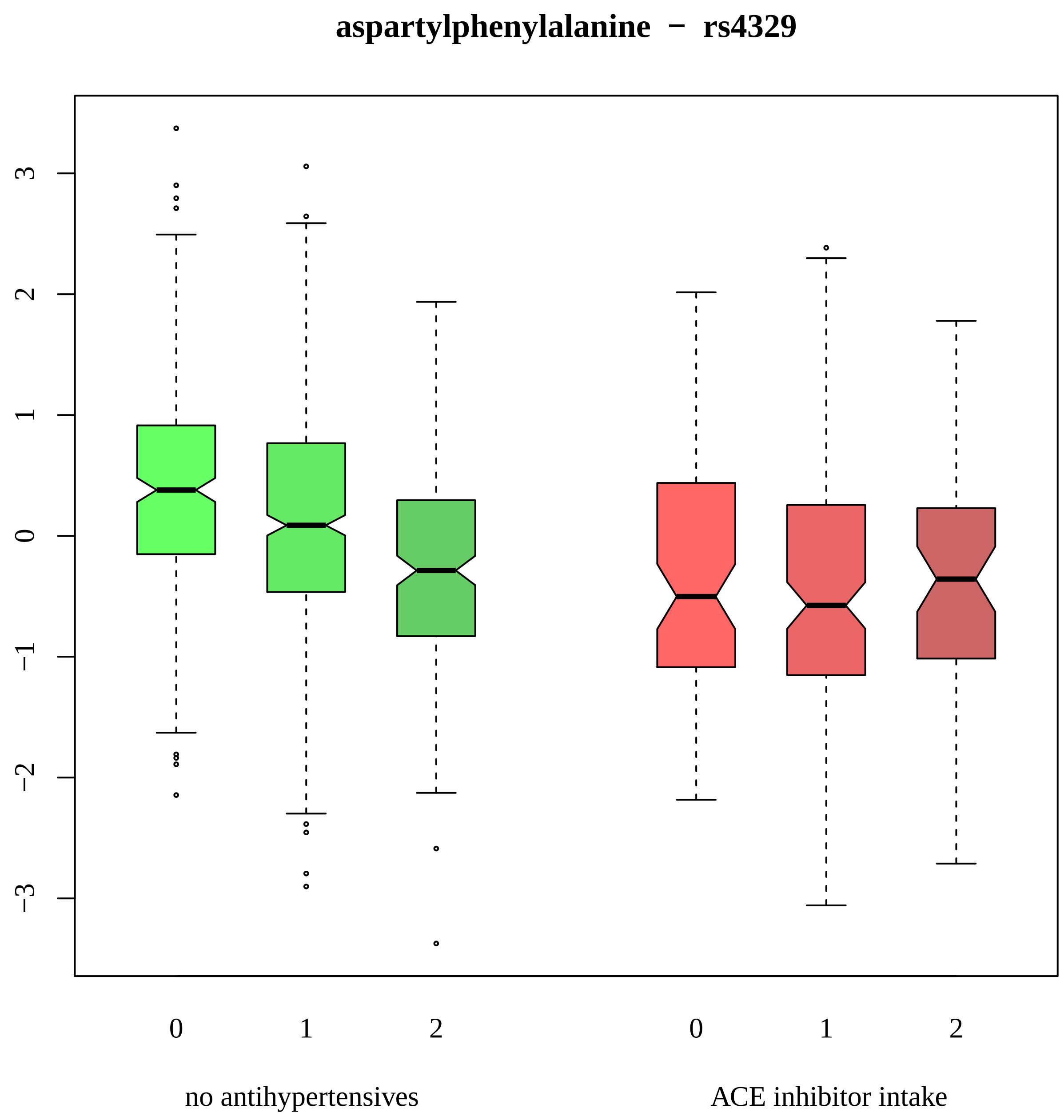

aspartylphenylalanine − rs4329
3
2
1
0
−1
−2
−3
0
1
2
0
1
2
no antihypertensives
ACE inhibitor intake

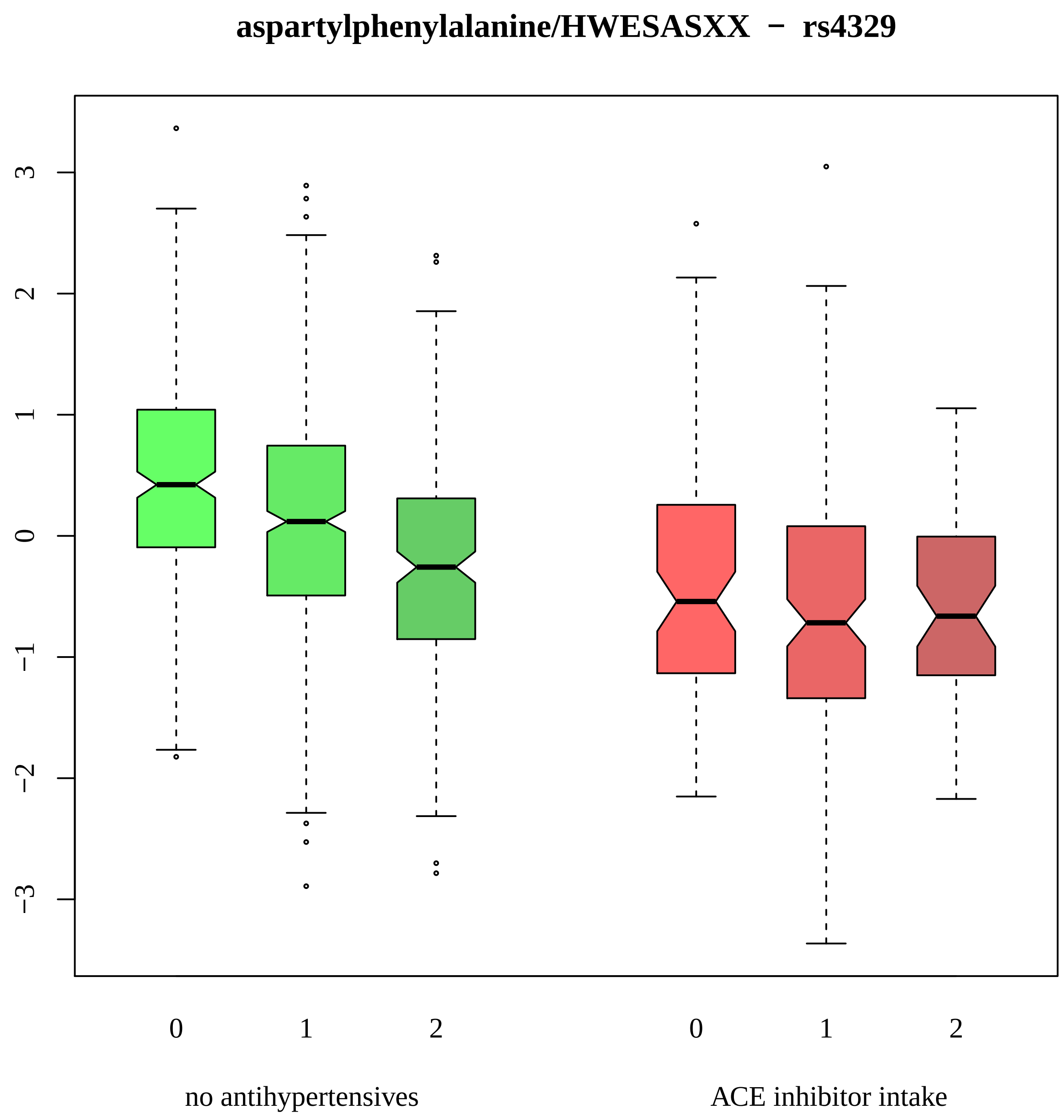
aspartylphenylalanine/HWESASXX – rs4329
3
2
1
0
−1
−2
−3
0
1
2
0
1
2
no antihypertensives
ACE inhibitor intake

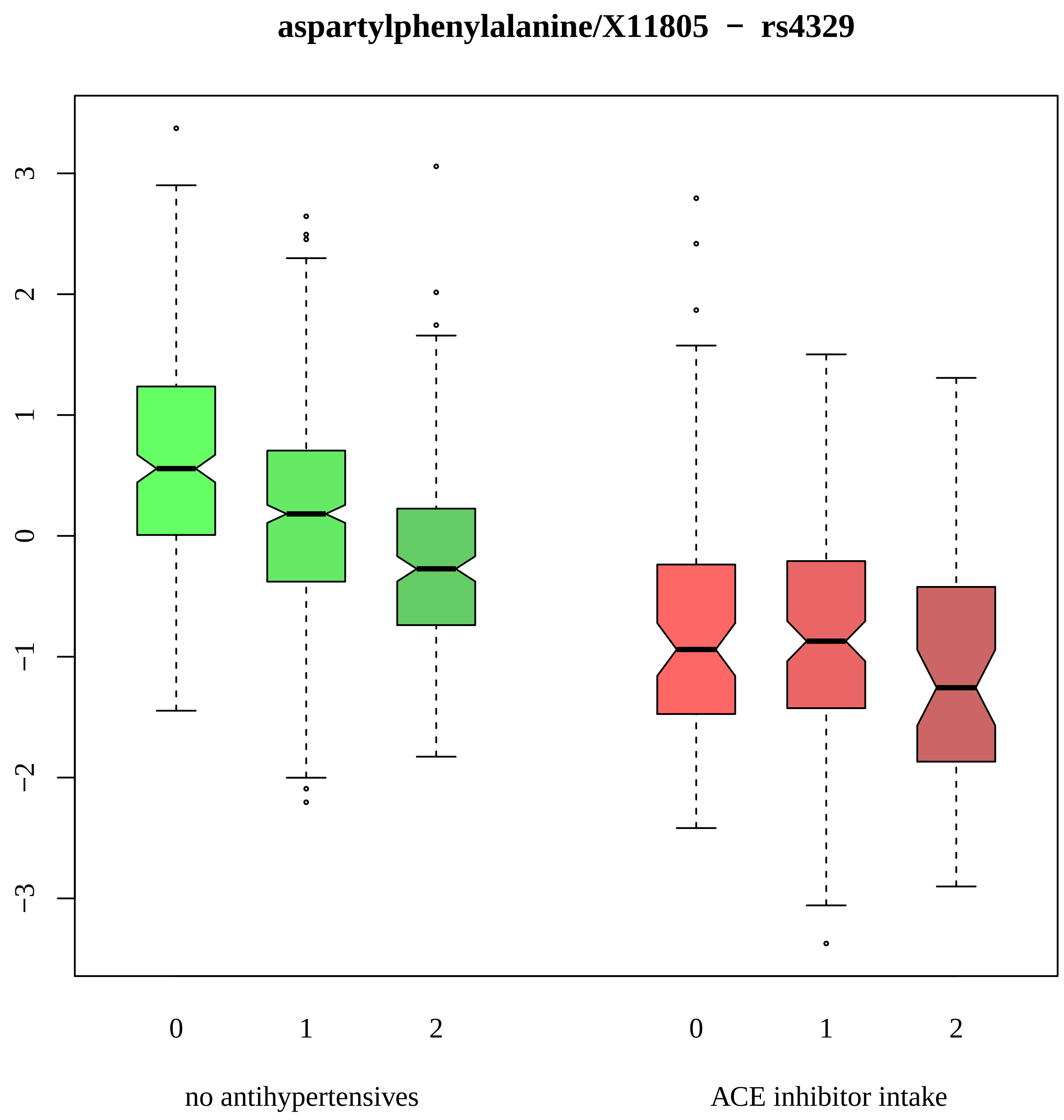
aspartylphenylalanine/X11805 – rs4329
3
2
1
0
−1
−2
−3
0
1
2
0
1
2
no antihypertensives
ACE inhibitor intake

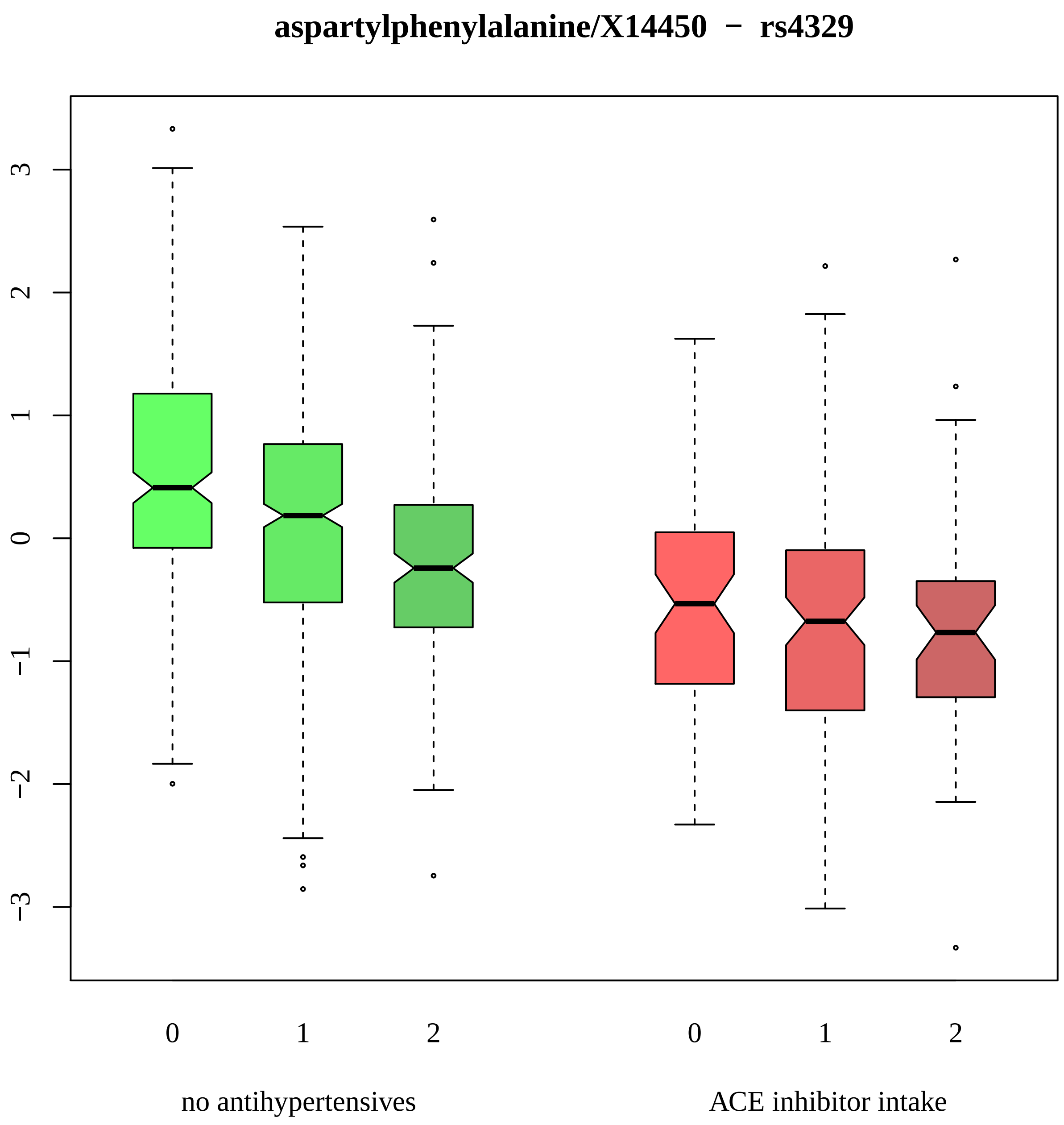
aspartylphenylalanine/X14450 – rs4329
3
2
1
0
−1
−2
−3
0
1
2
0
1
2
no antihypertensives
ACE inhibitor intake

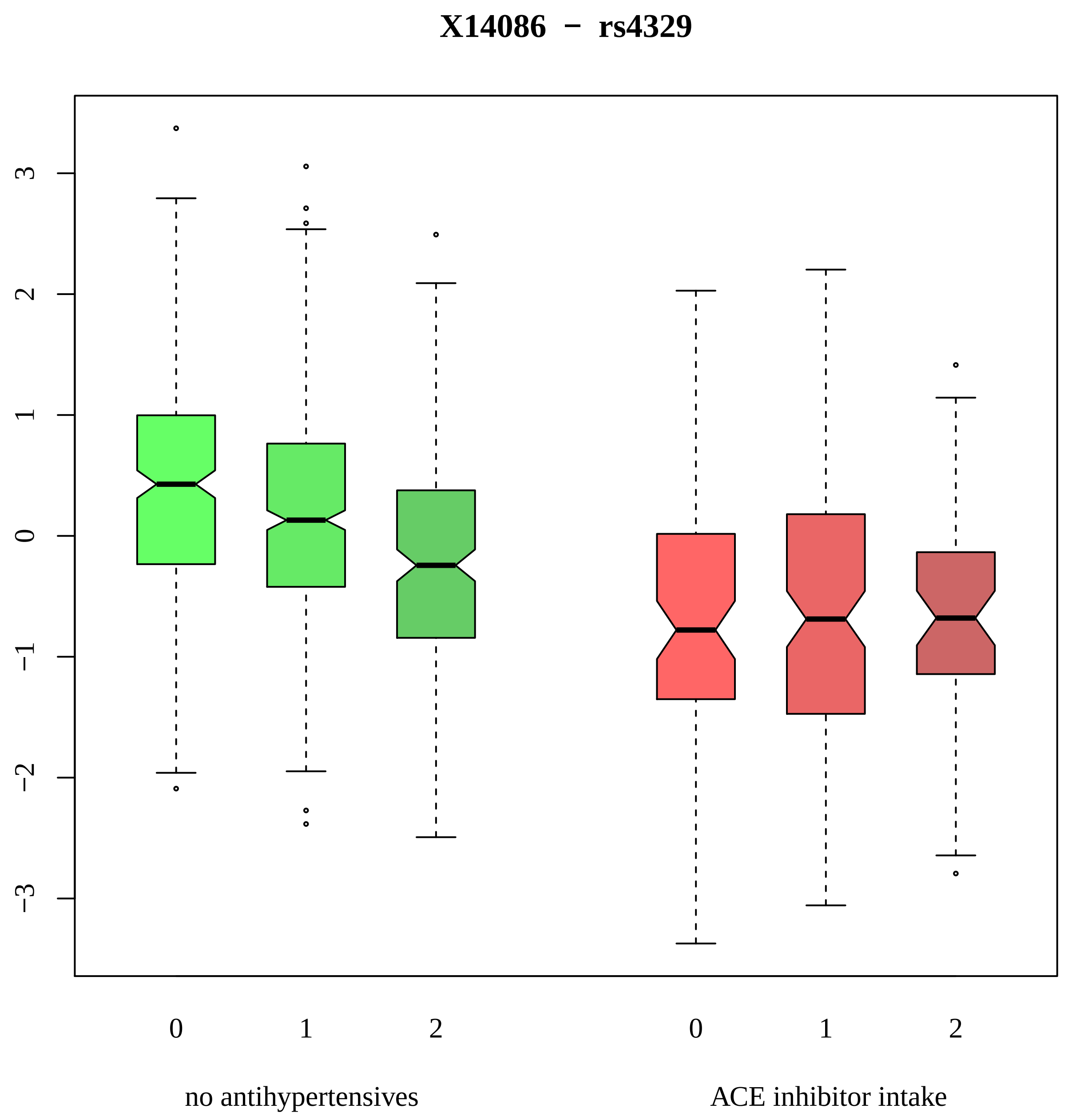
X14086 – rs4329
3
2
1
0
−1
−2
−3
0
1
2
0
1
2
no antihypertensives
ACE inhibitor intake

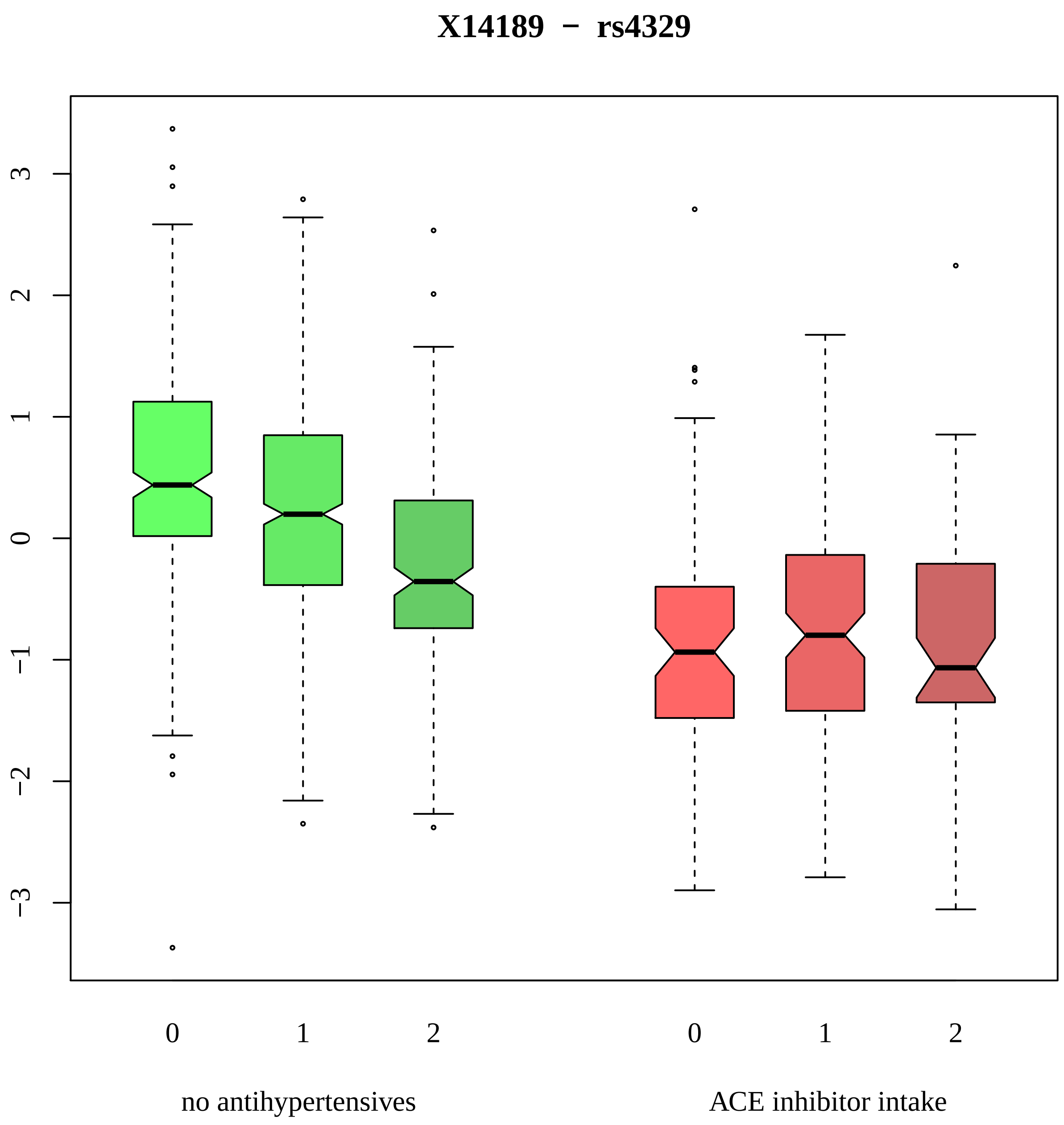

X14189 – rs4329
3
2
1
0
−1
−2
−3
0
1
2
0
1
2
no antihypertensives
ACE inhibitor intake

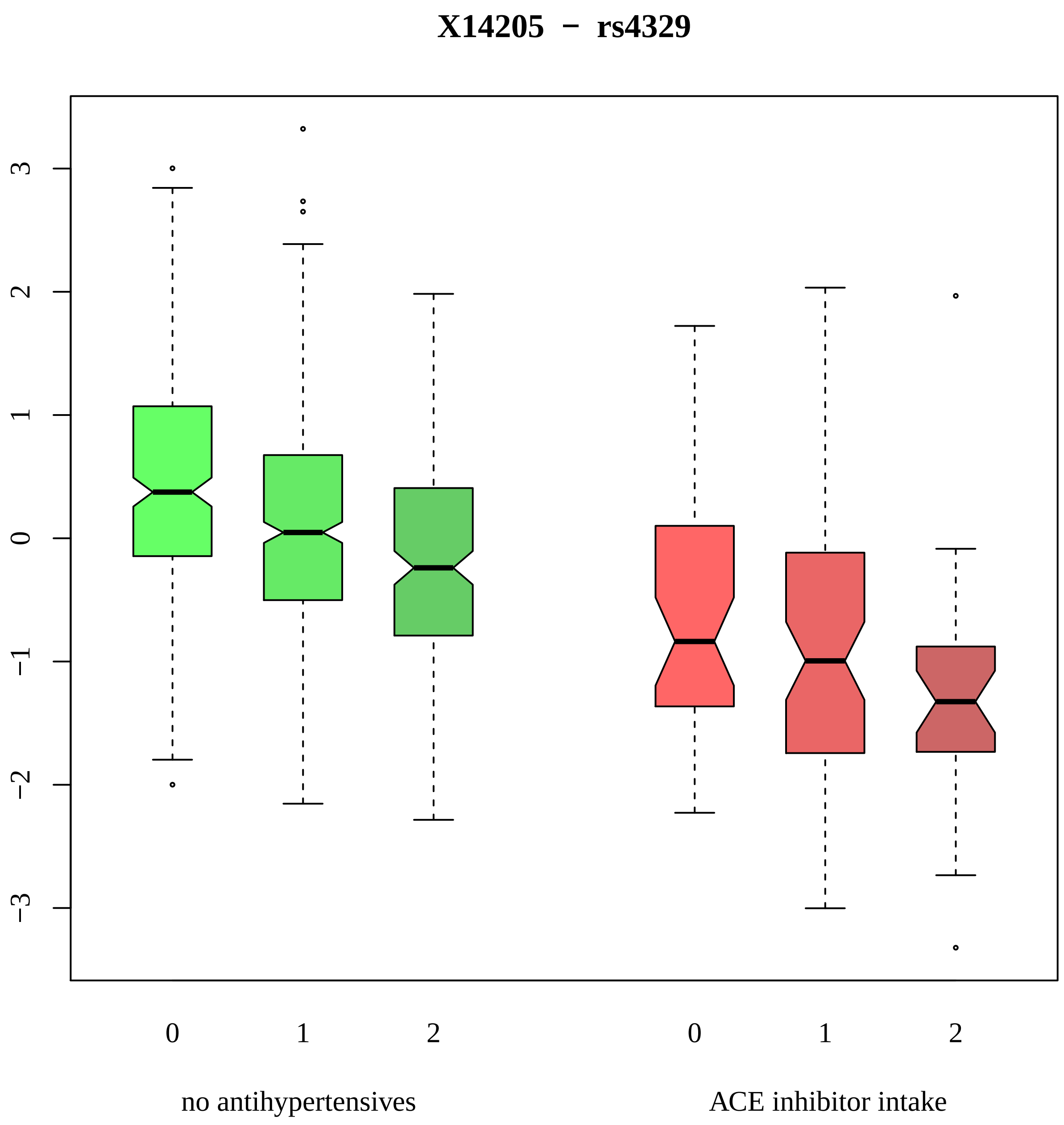

X14205 – rs4329
3
2
1
0
−1
−2
−3
0
1
2
0
1
2
no antihypertensives
ACE inhibitor intake

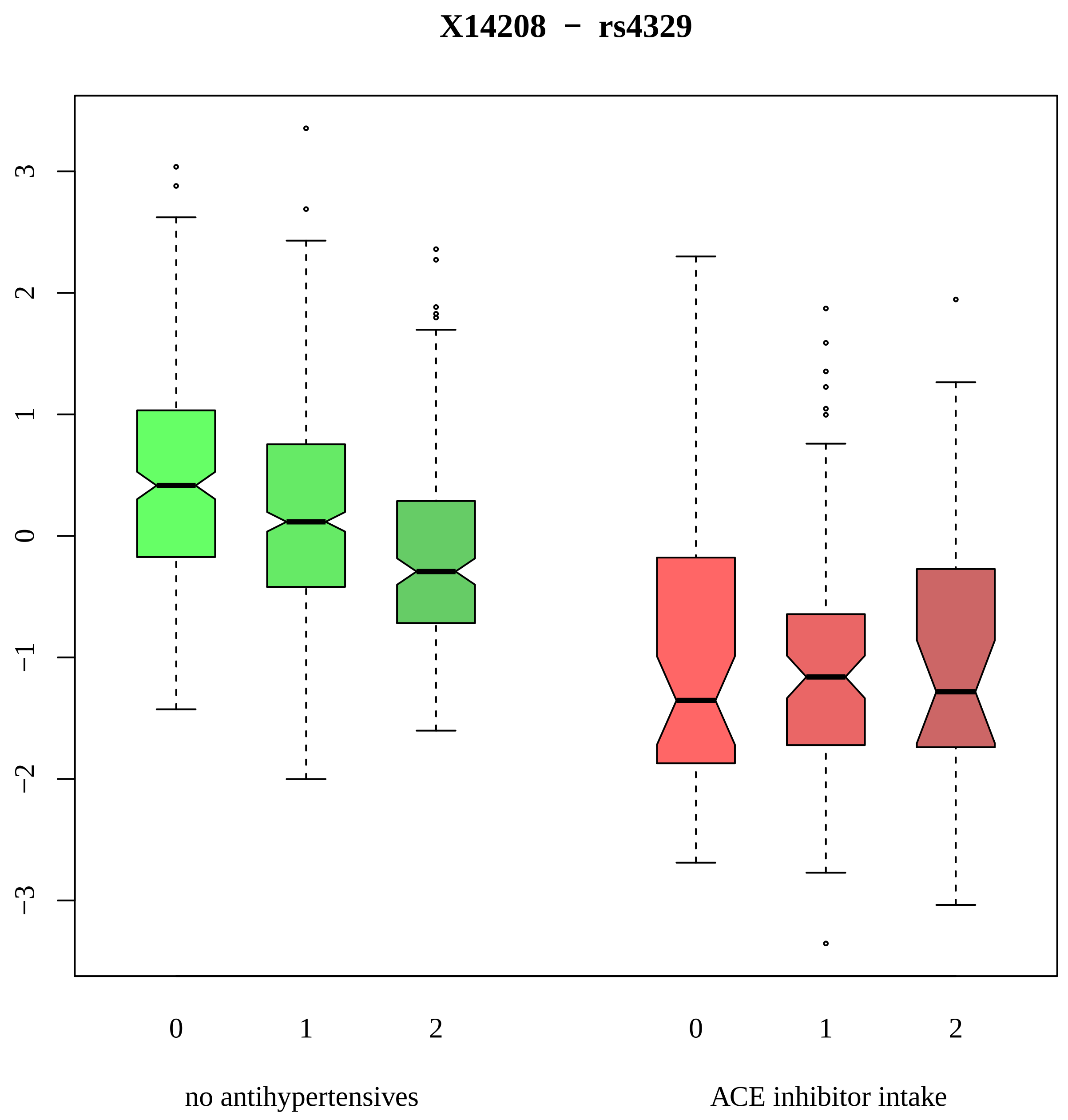
X14208 – rs4329
3
2
1
0
−1
−2
−3
0
1
2
0
1
2
no antihypertensives
ACE inhibitor intake

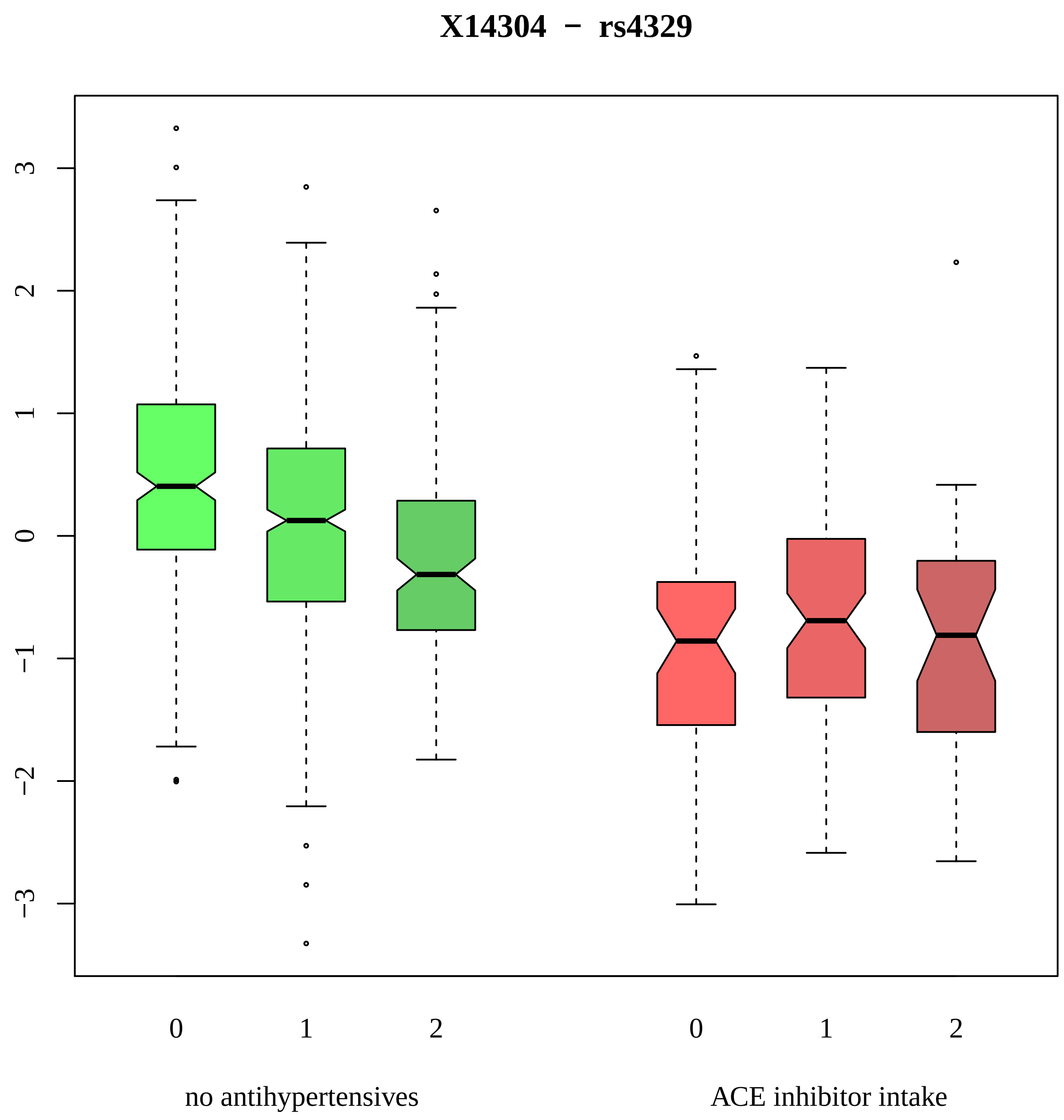
X14304 – rs4329
3
2
1
0
−1
−2
−3
0
1
2
0
1
2
no antihypertensives
ACE inhibitor intake

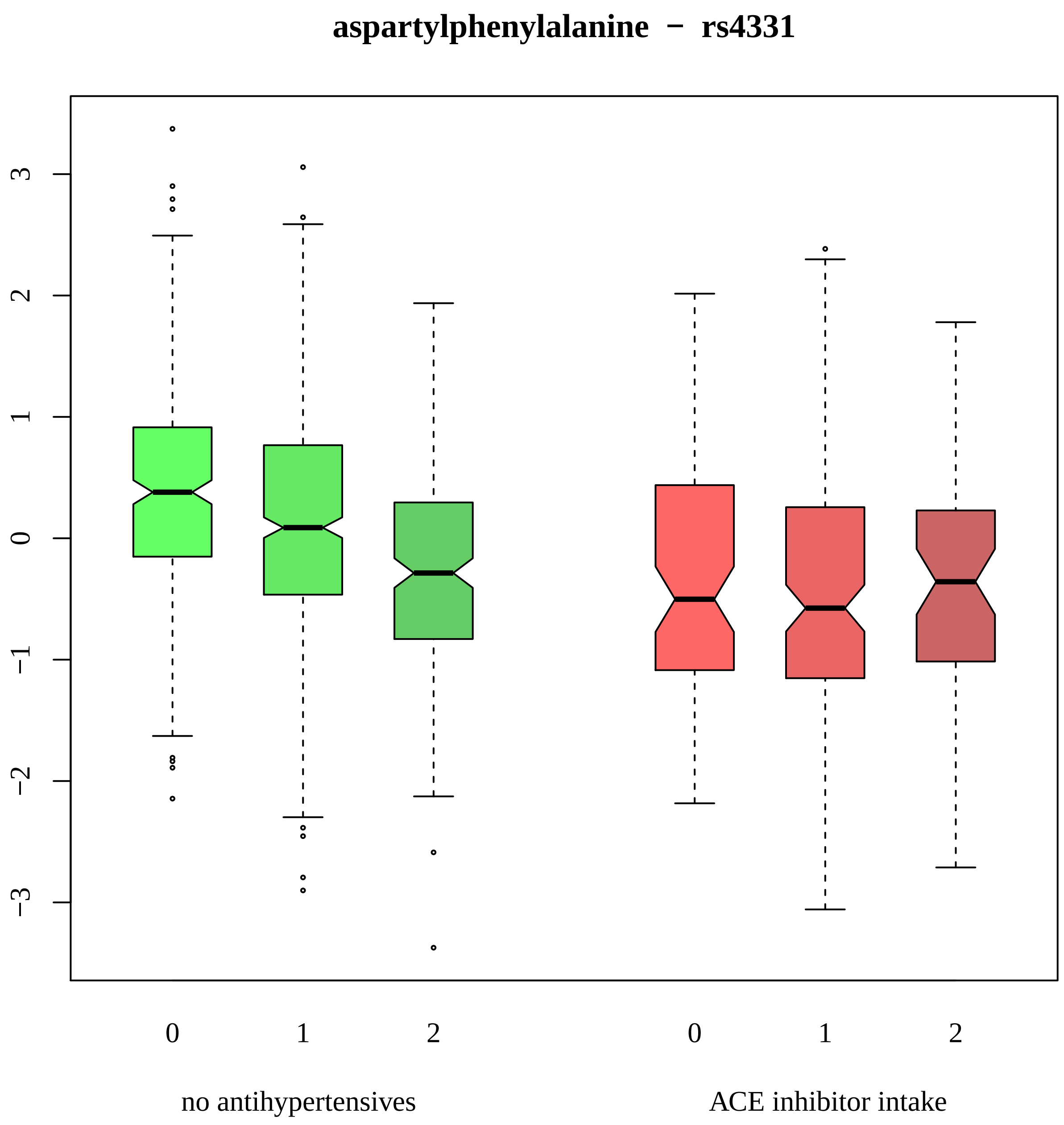

aspartylphenylalanine − rs4331
3
2
1
0
−1
−2
−3
0
1
2
0
1
2
no antihypertensives
ACE inhibitor intake

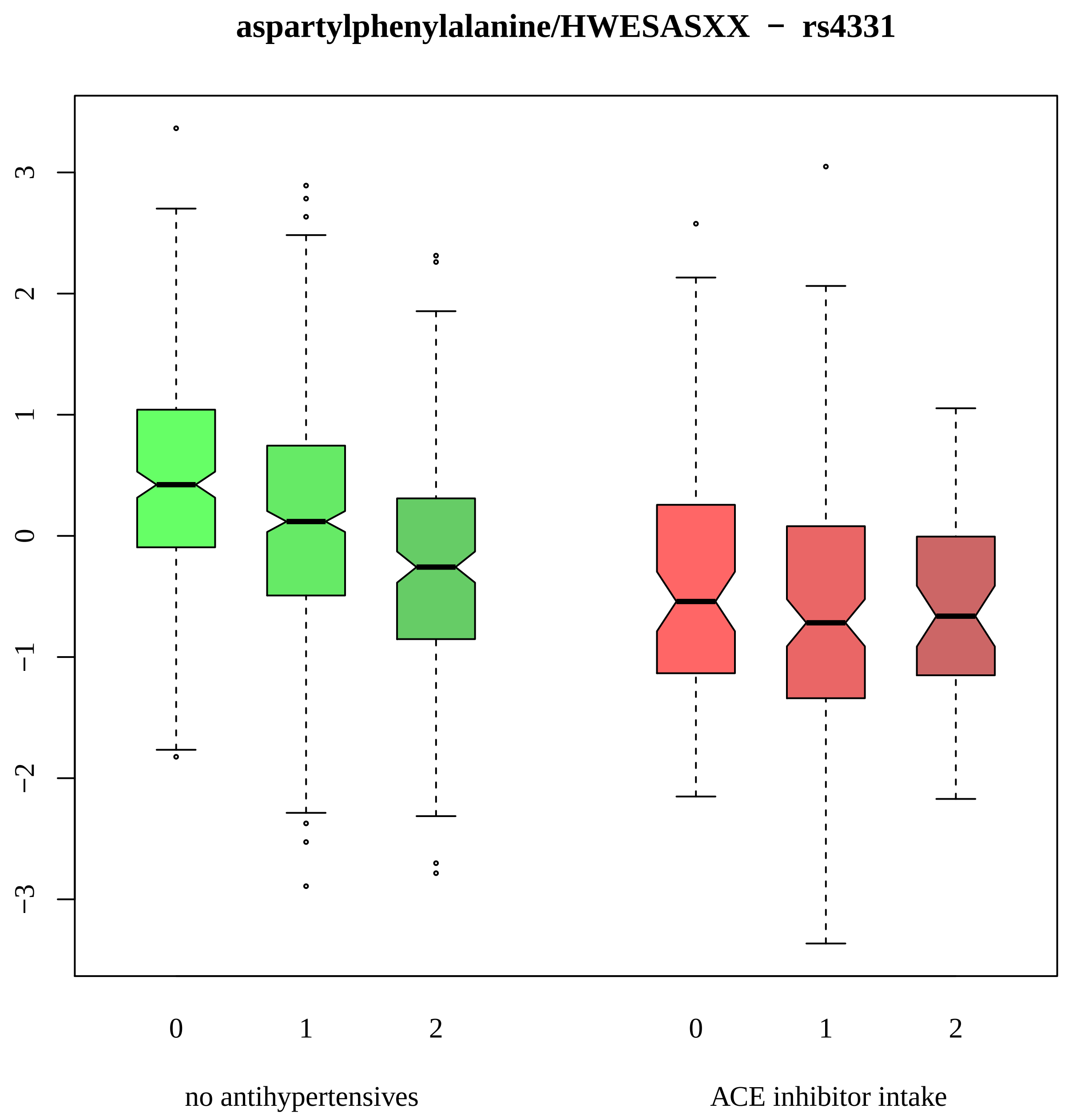
aspartylphenylalanine/HWESASXX – rs4331
3
2
1
0
−1
−2
−3
0
1
2
0
1
2
no antihypertensives
ACE inhibitor intake

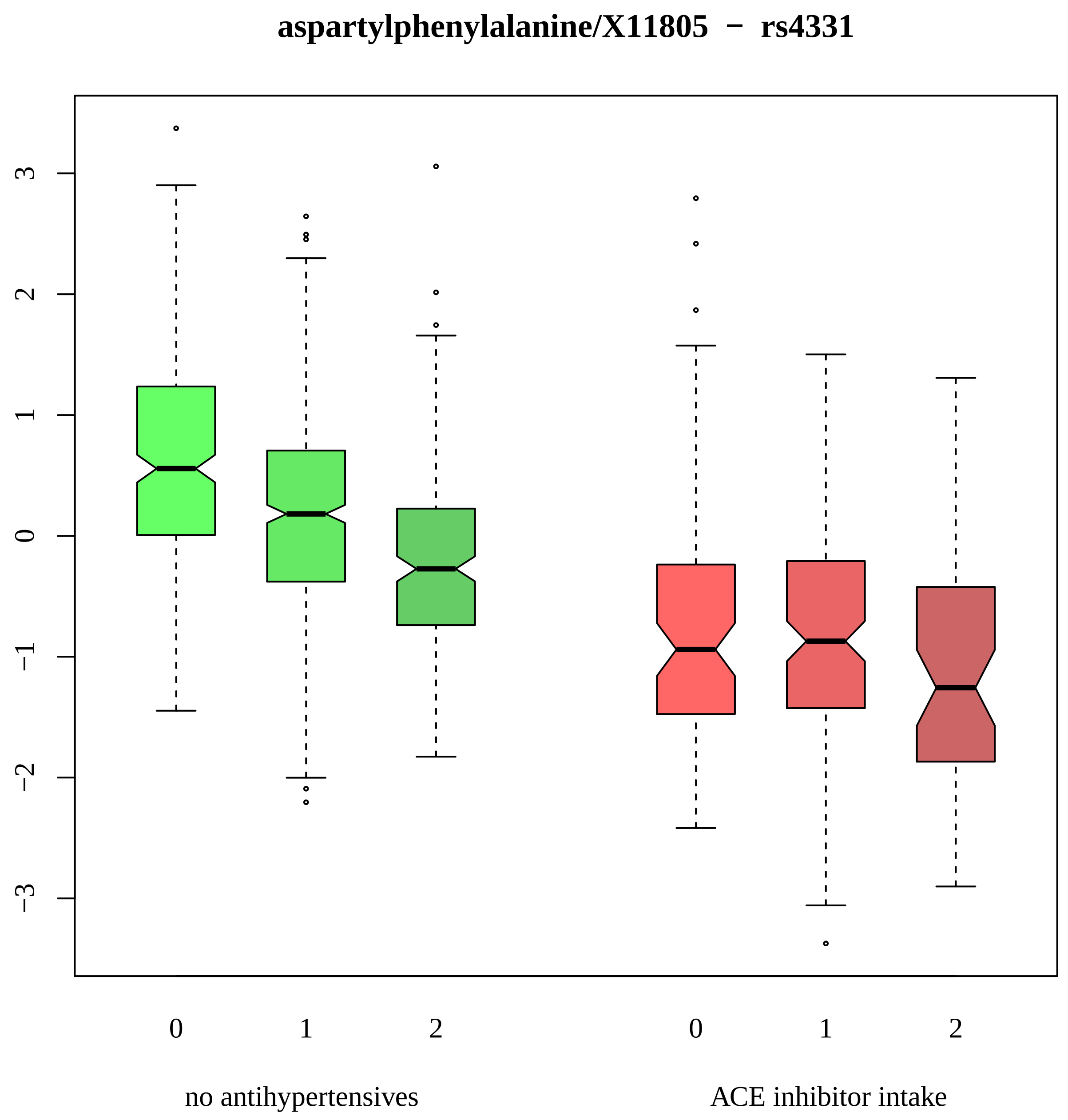

aspartylphenylalanine/X11805 – rs4331
3
2
1
0
−1
−2
−3
0
1
2
0
1
2
no antihypertensives
ACE inhibitor intake

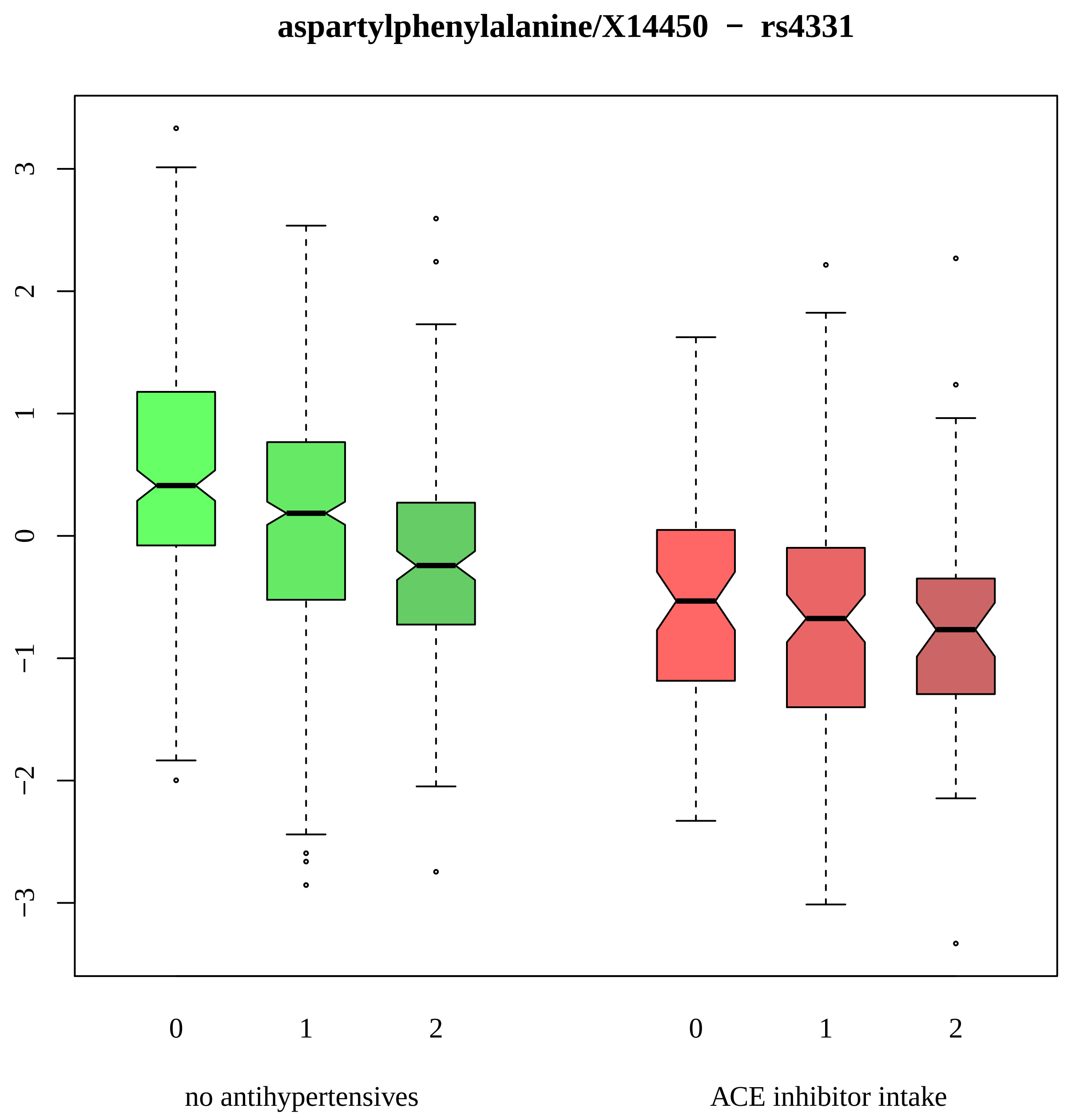

aspartylphenylalanine/X14450 – rs4331
3
2
1
0
−1
−2
−3
0
1
2
0
1
2
no antihypertensives
ACE inhibitor intake

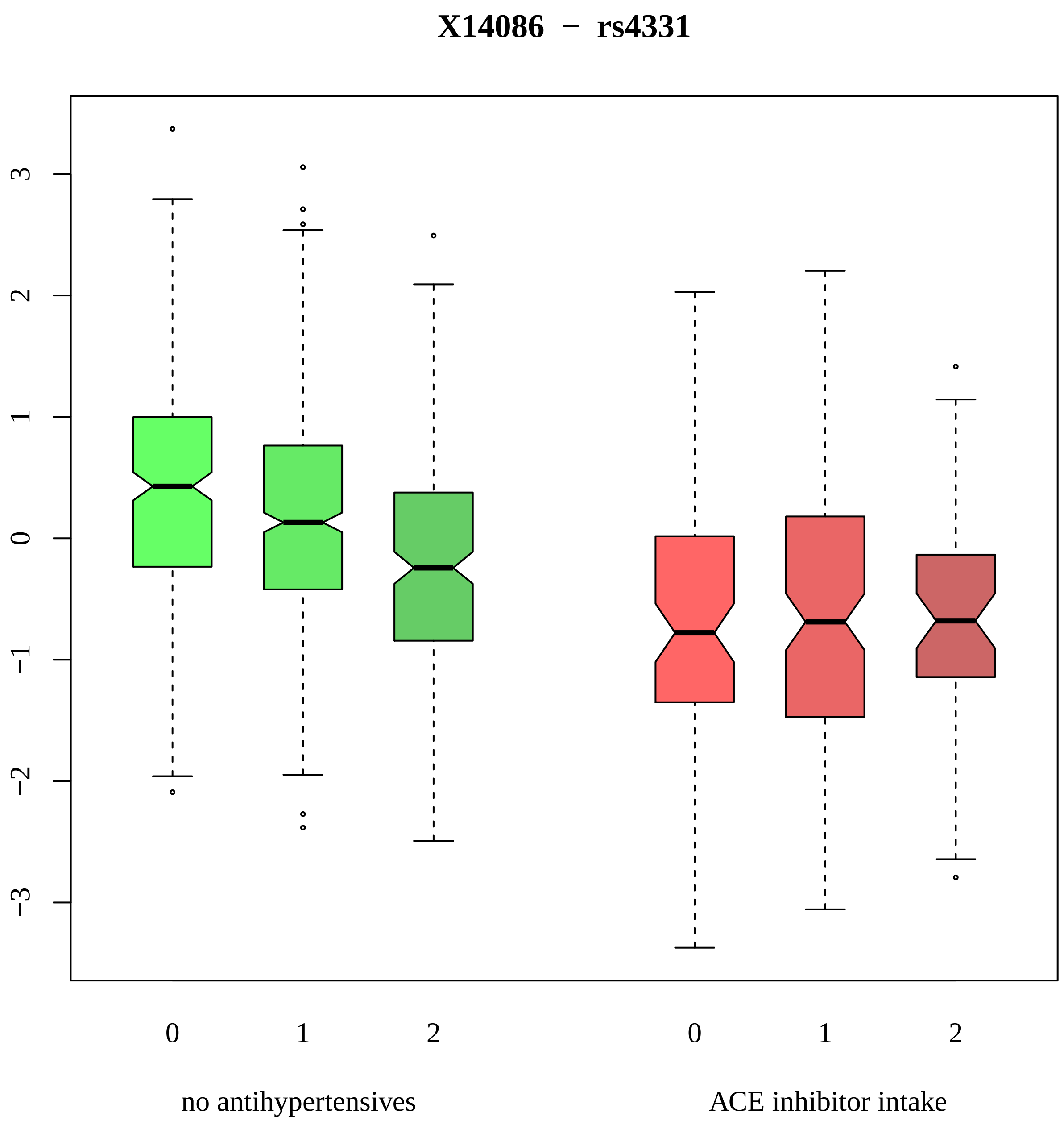

X14086 – rs4331
3
2
1
0
−1
−2
−3
0
1
2
0
1
2
no antihypertensives
ACE inhibitor intake

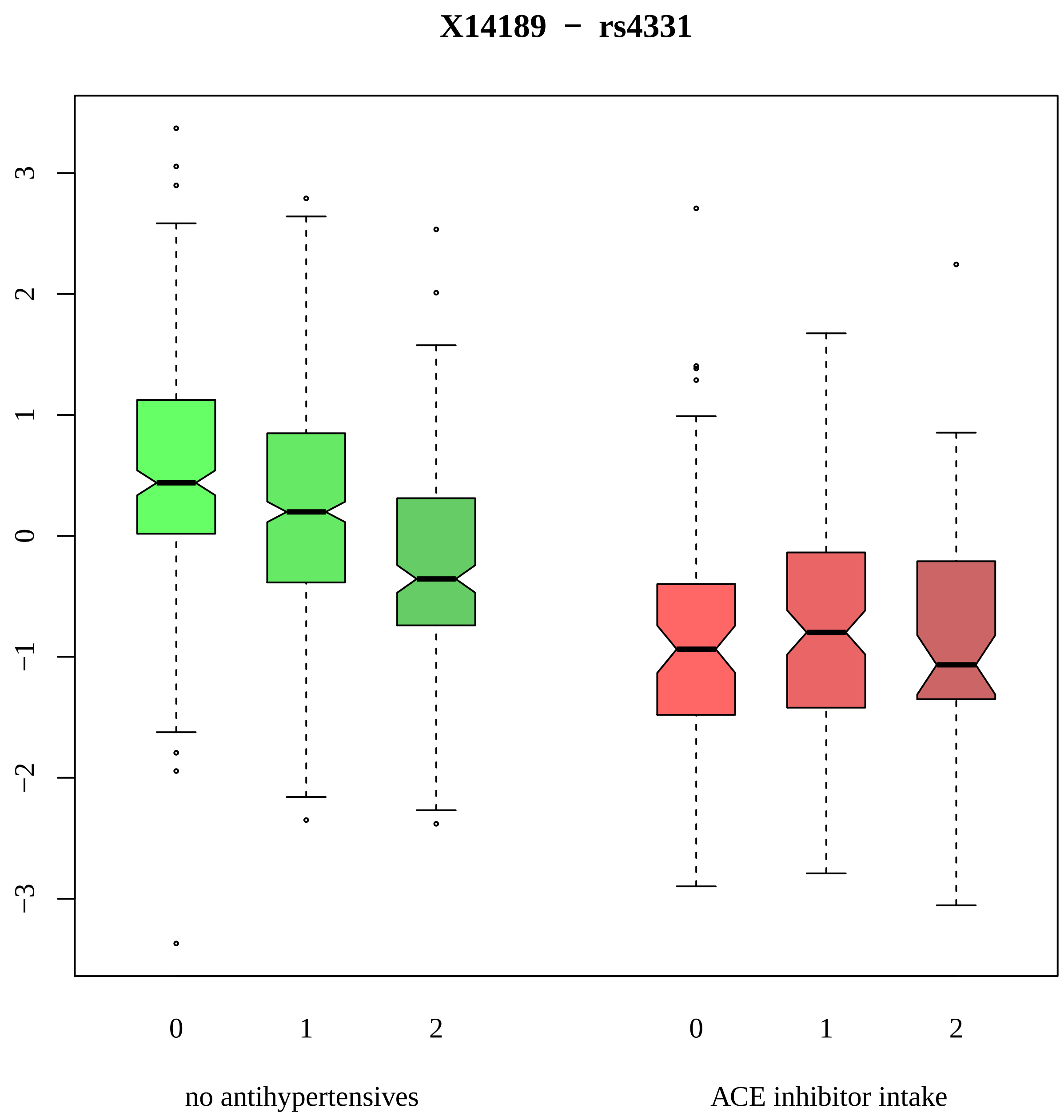
X14189 – rs4331
3
2
1
0
−1
−2
−3
0
1
2
0
1
2
no antihypertensives
ACE inhibitor intake

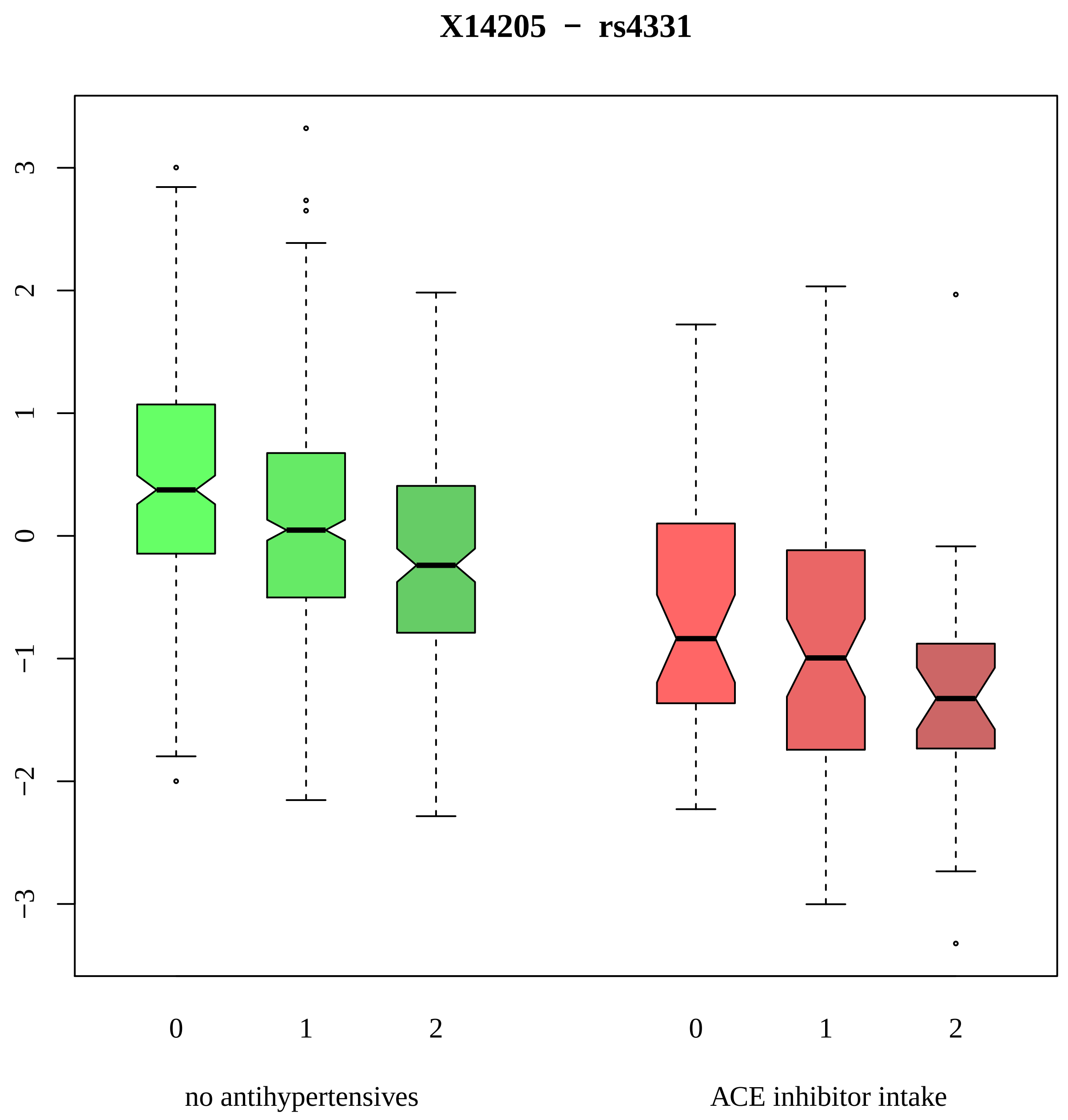
X14205 – rs4331
3
2
1
0
−1
−2
−3
0
1
2
0
1
2
no antihypertensives
ACE inhibitor intake

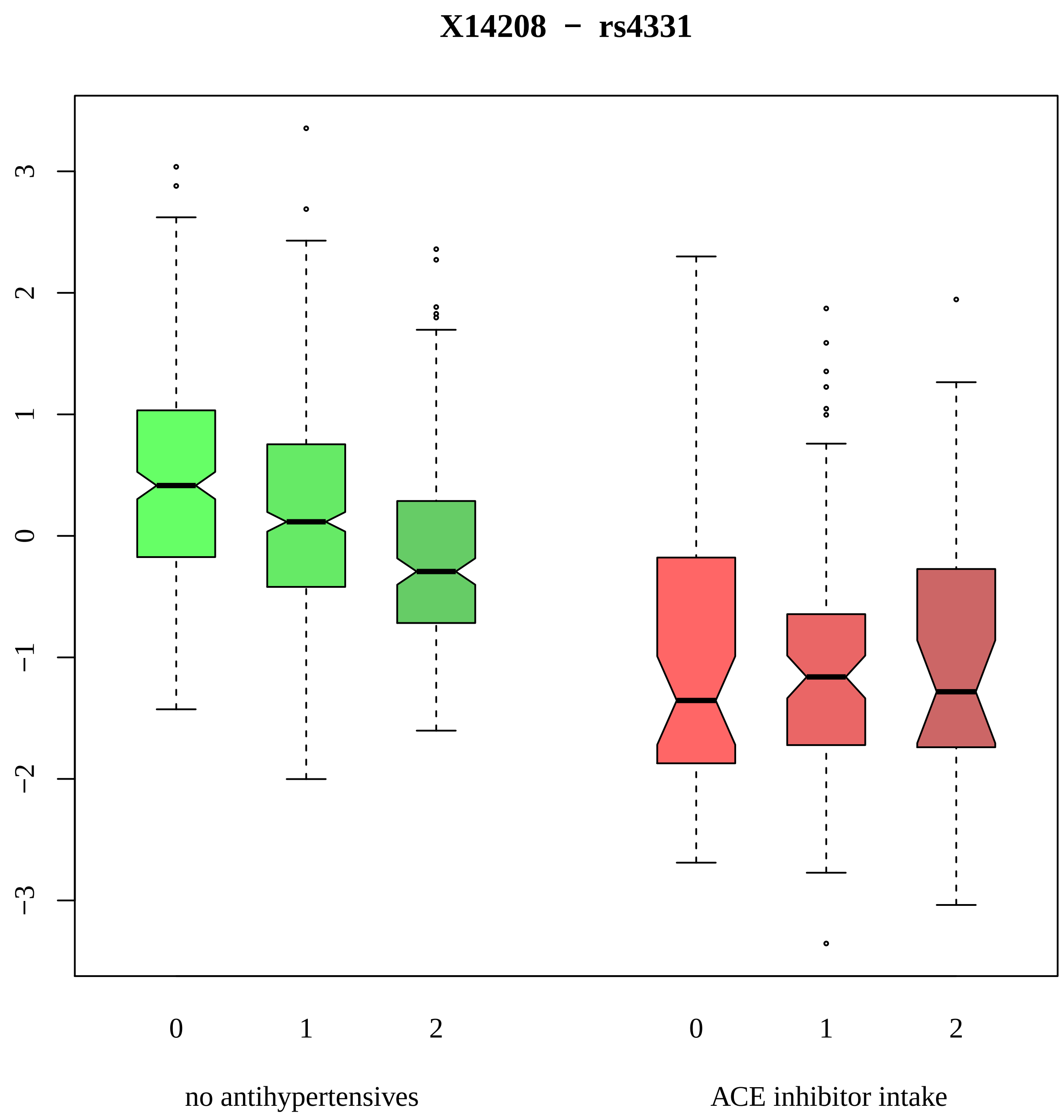
X14208 – rs4331
3
2
1
0
−1
−2
−3
0
1
2
0
1
2
no antihypertensives
ACE inhibitor intake

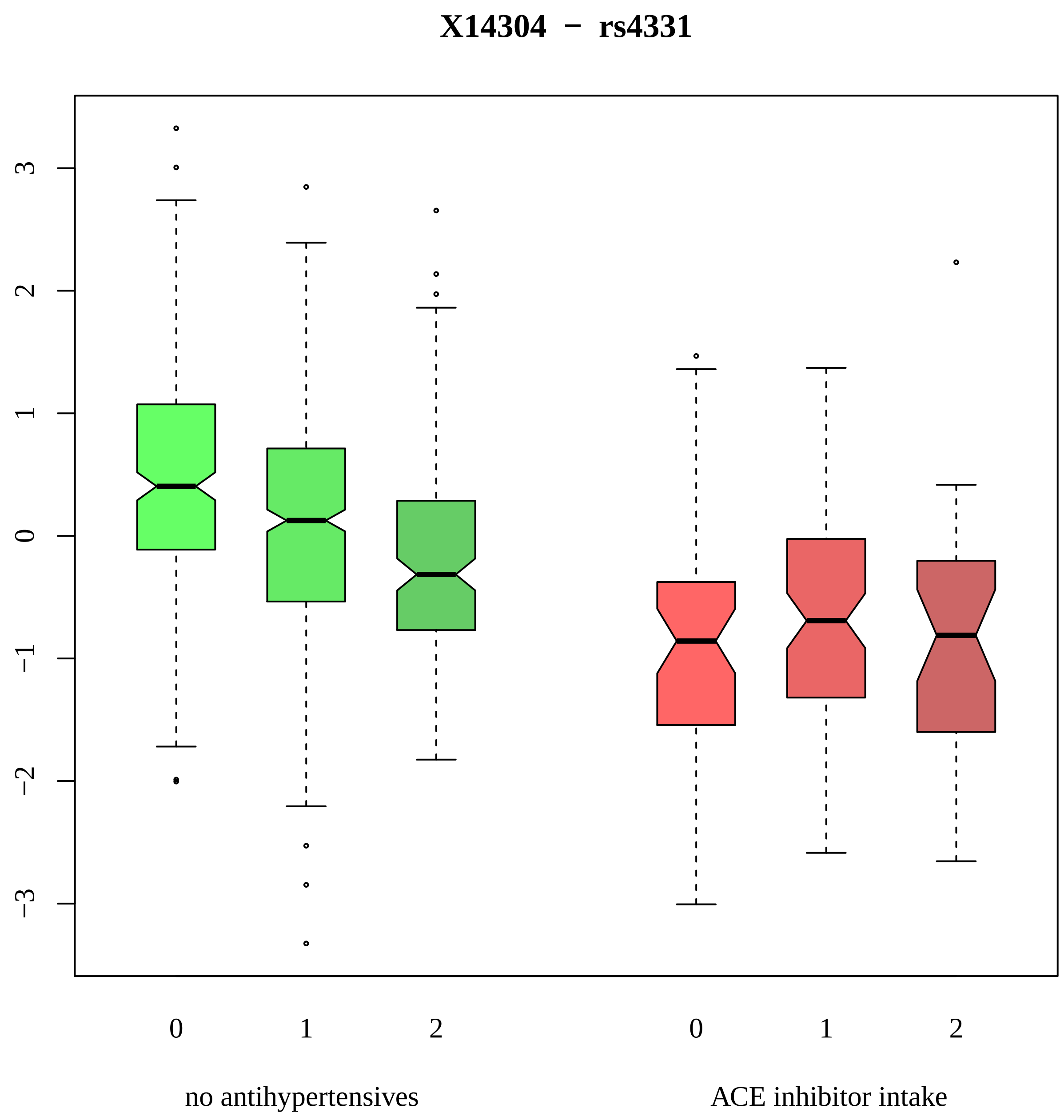

X14304 – rs4331
3
2
1
0
−1
−2
−3
0
1
2
0
1
2
no antihypertensives
ACE inhibitor intake

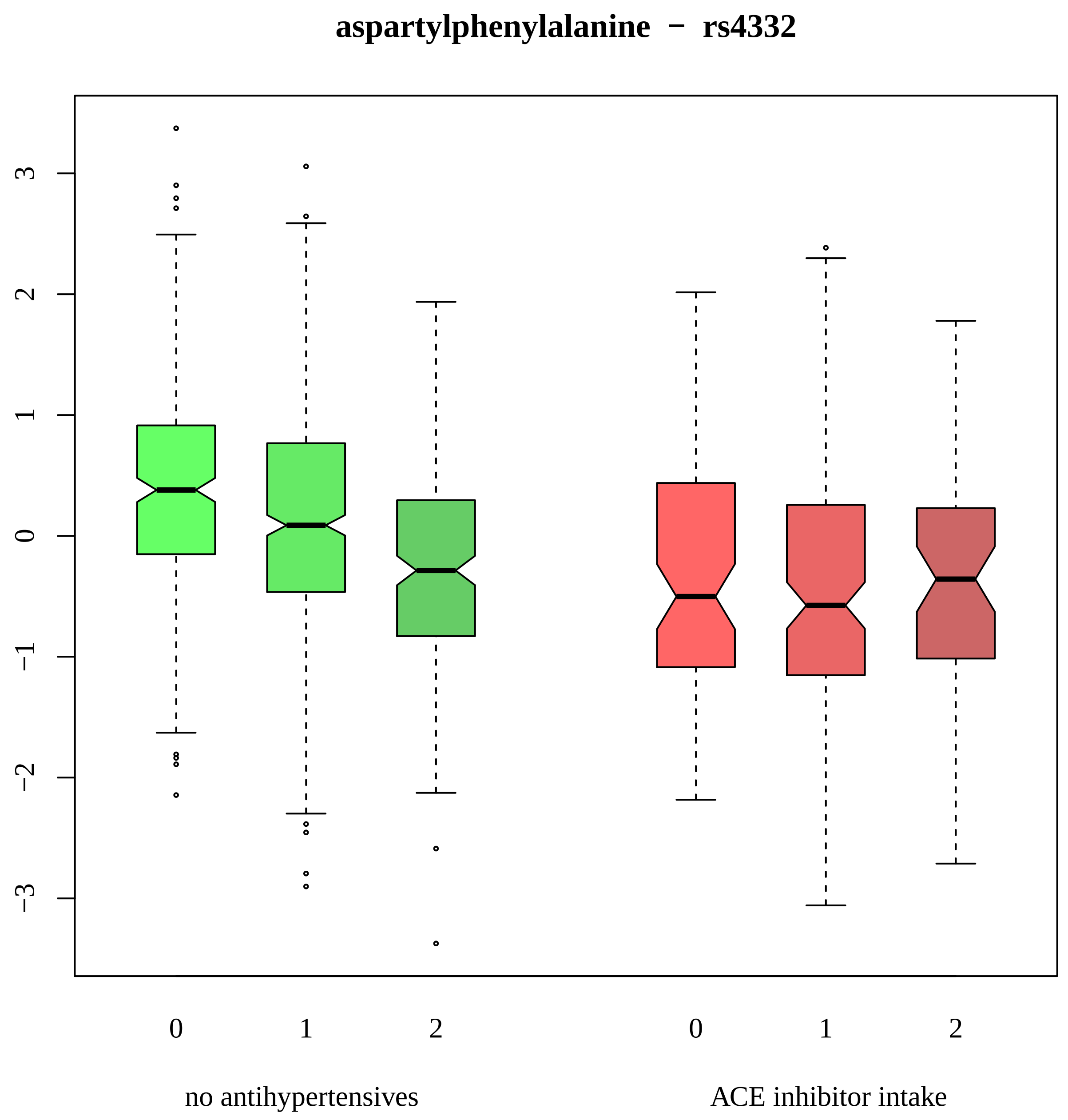
aspartylphenylalanine − rs4332
3
2
1
0
−1
−2
−3
0
1
2
0
1
2
no antihypertensives
ACE inhibitor intake

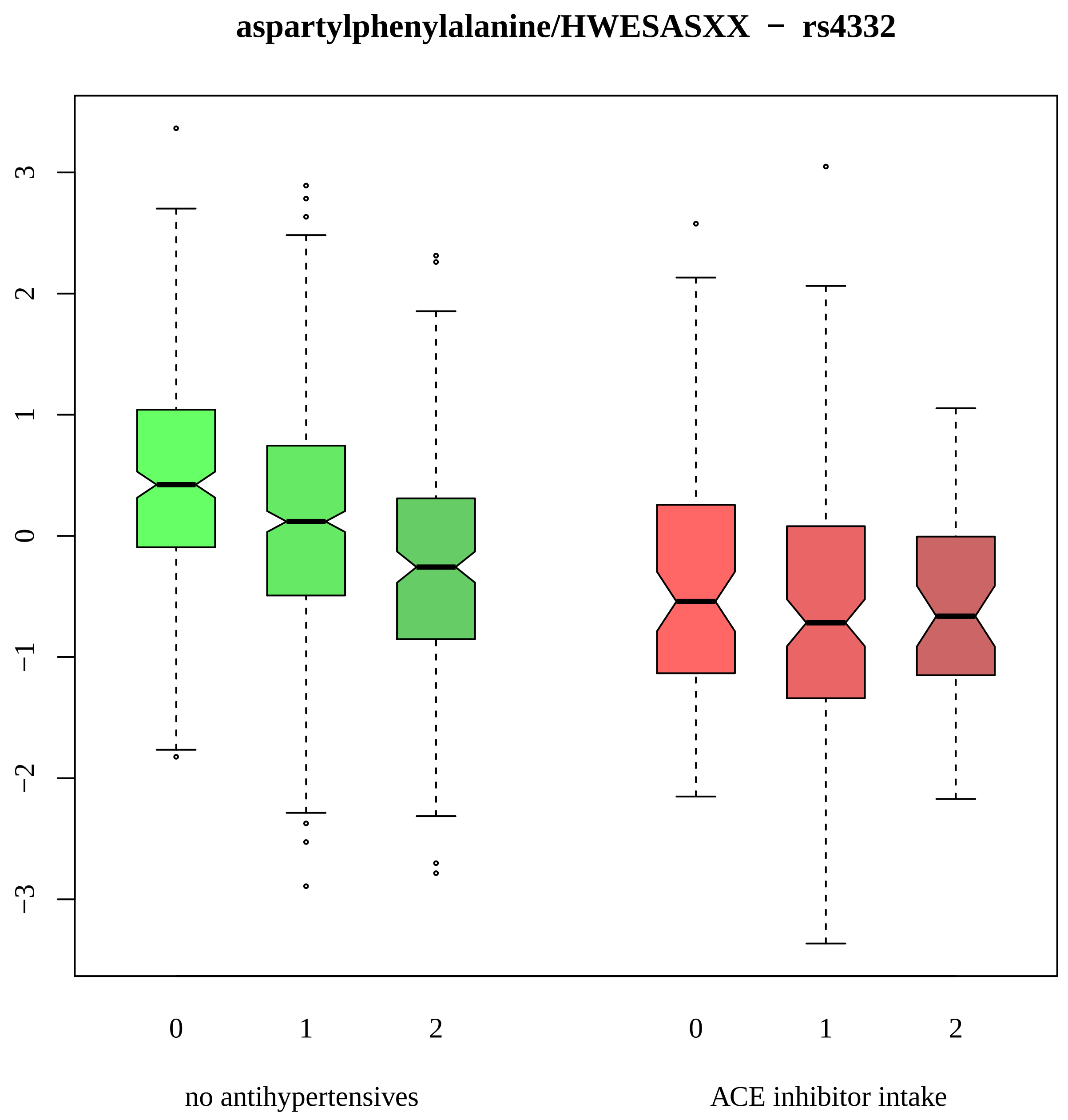

aspartylphenylalanine/HWESASXX – rs4332
3
2
1
0
−1
−2
−3
0
1
2
0
1
2
no antihypertensives
ACE inhibitor intake

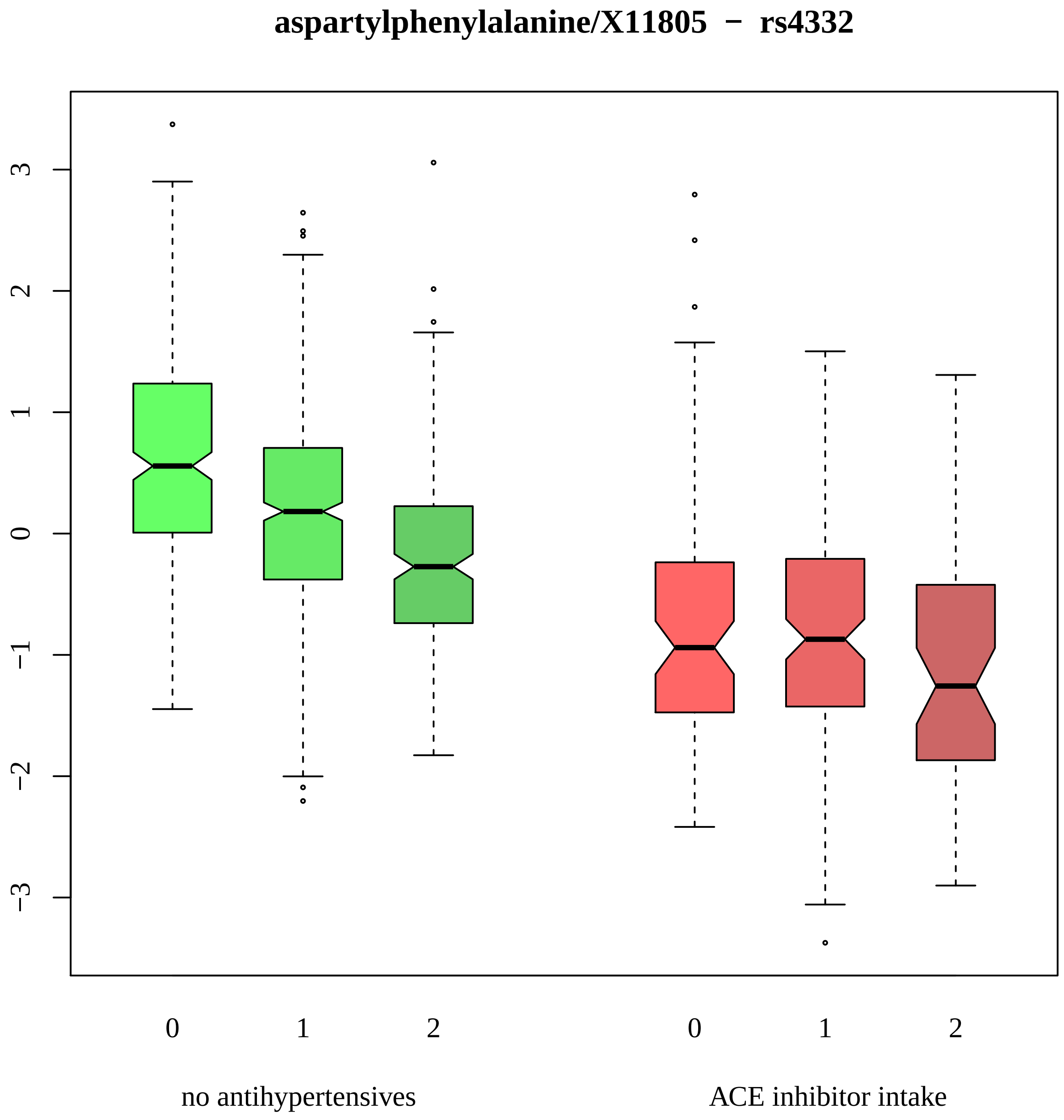

aspartylphenylalanine/X11805 – rs4332
3
2
1
0
−1
−2
−3
0
1
2
0
1
2
no antihypertensives
ACE inhibitor intake

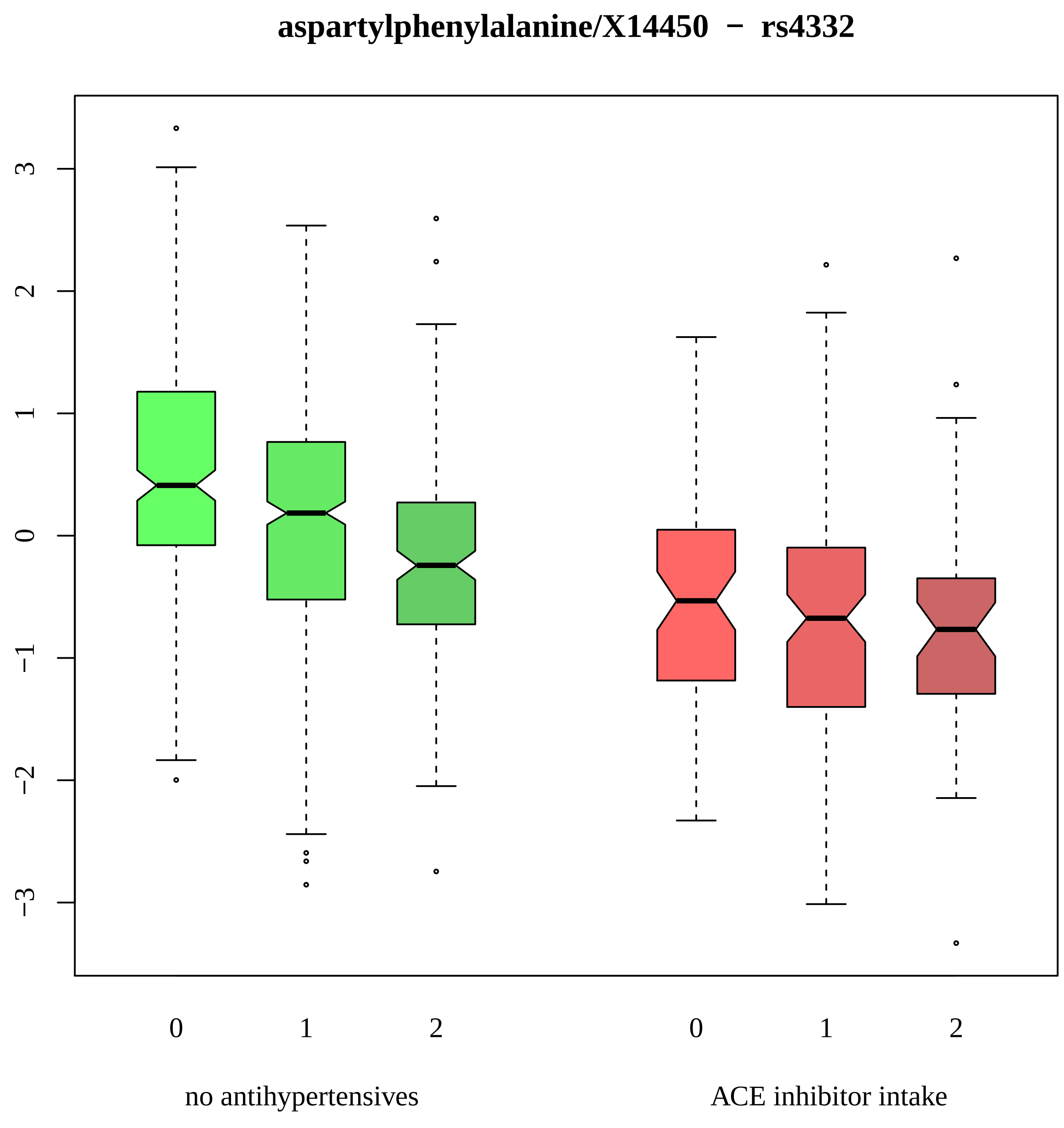

aspartylphenylalanine/X14450 − rs4332
3
2
1
0
−1
−2
−3
0
1
2
0
1
2
no antihypertensives
ACE inhibitor intake

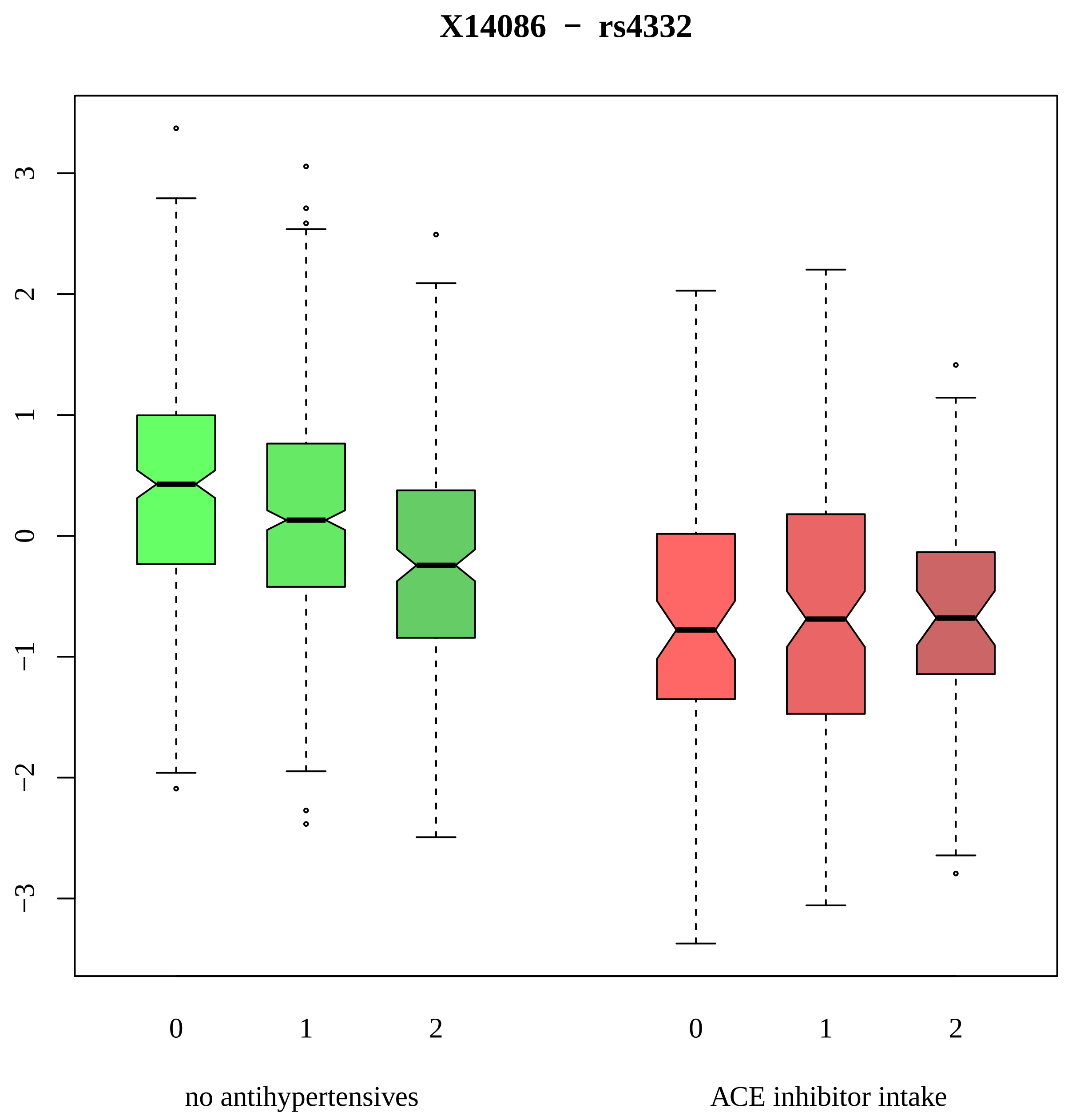
X14086 – rs4332
3
2
1
0
−1
−2
−3
0
1
2
0
1
2
no antihypertensives
ACE inhibitor intake

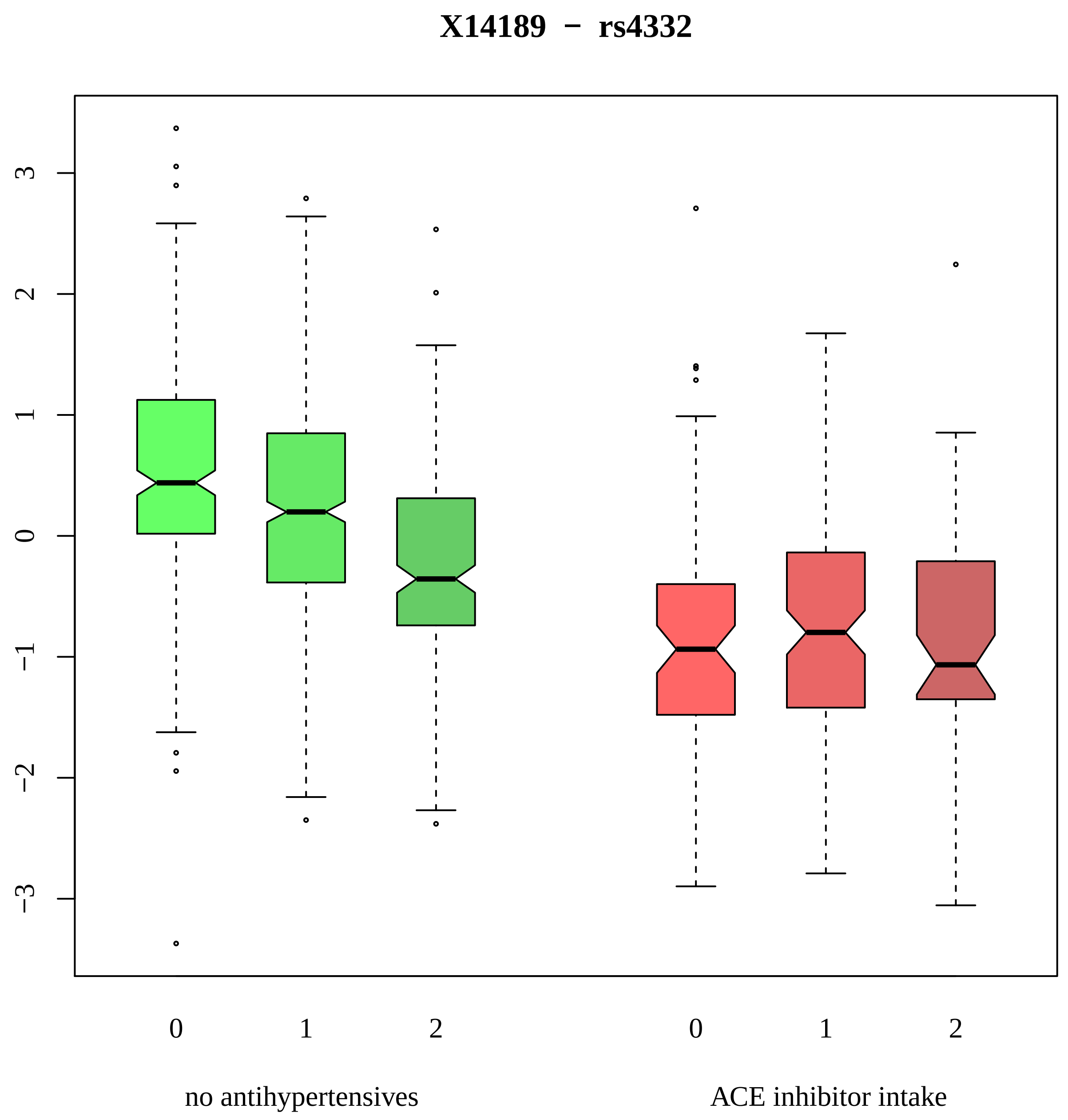

X14189 – rs4332
3
2
1
0
−1
−2
−3
0
1
2
0
1
2
no antihypertensives
ACE inhibitor intake

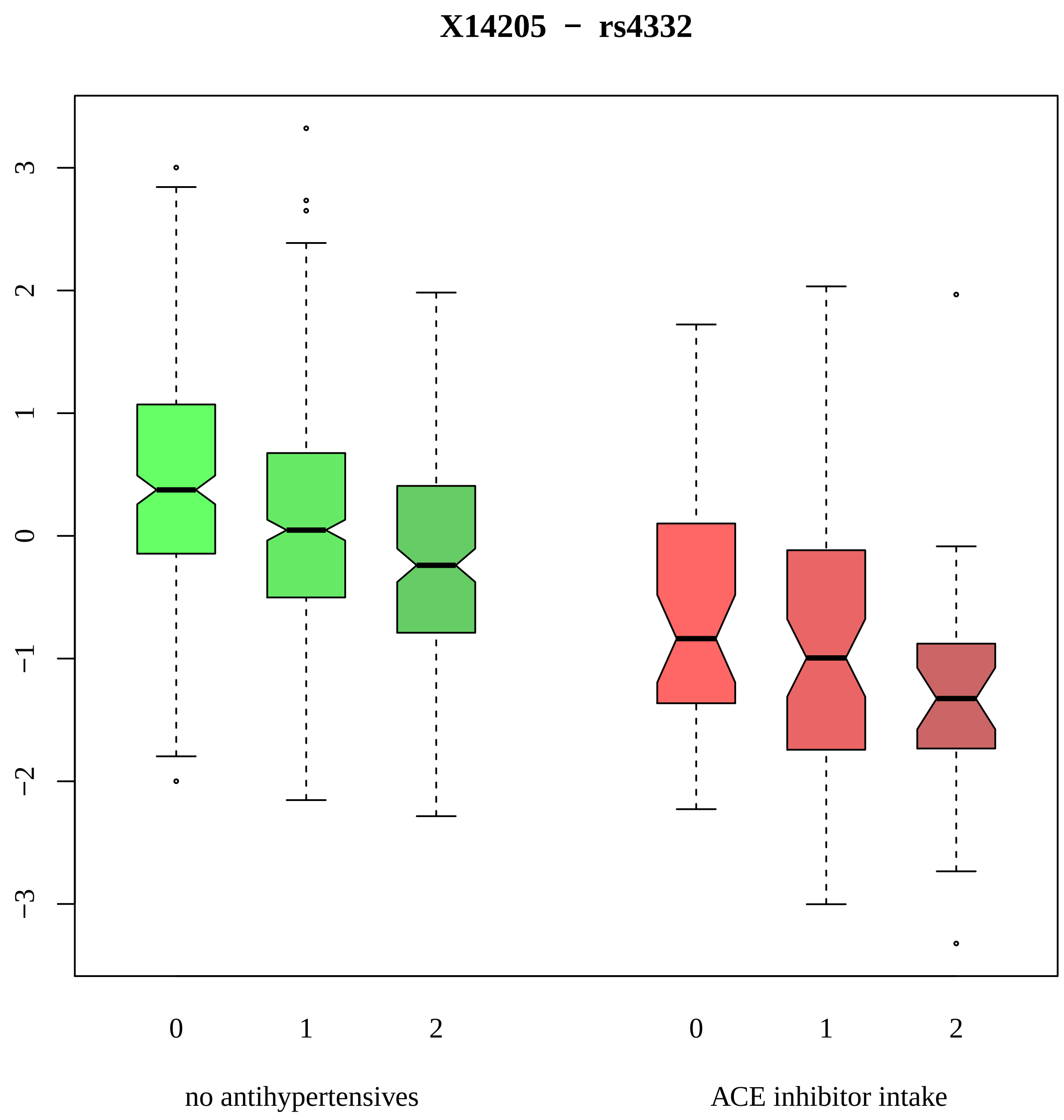

X14205 – rs4332
3
2
1
0
−1
−2
−3
0
1
2
0
1
2
no antihypertensives
ACE inhibitor intake

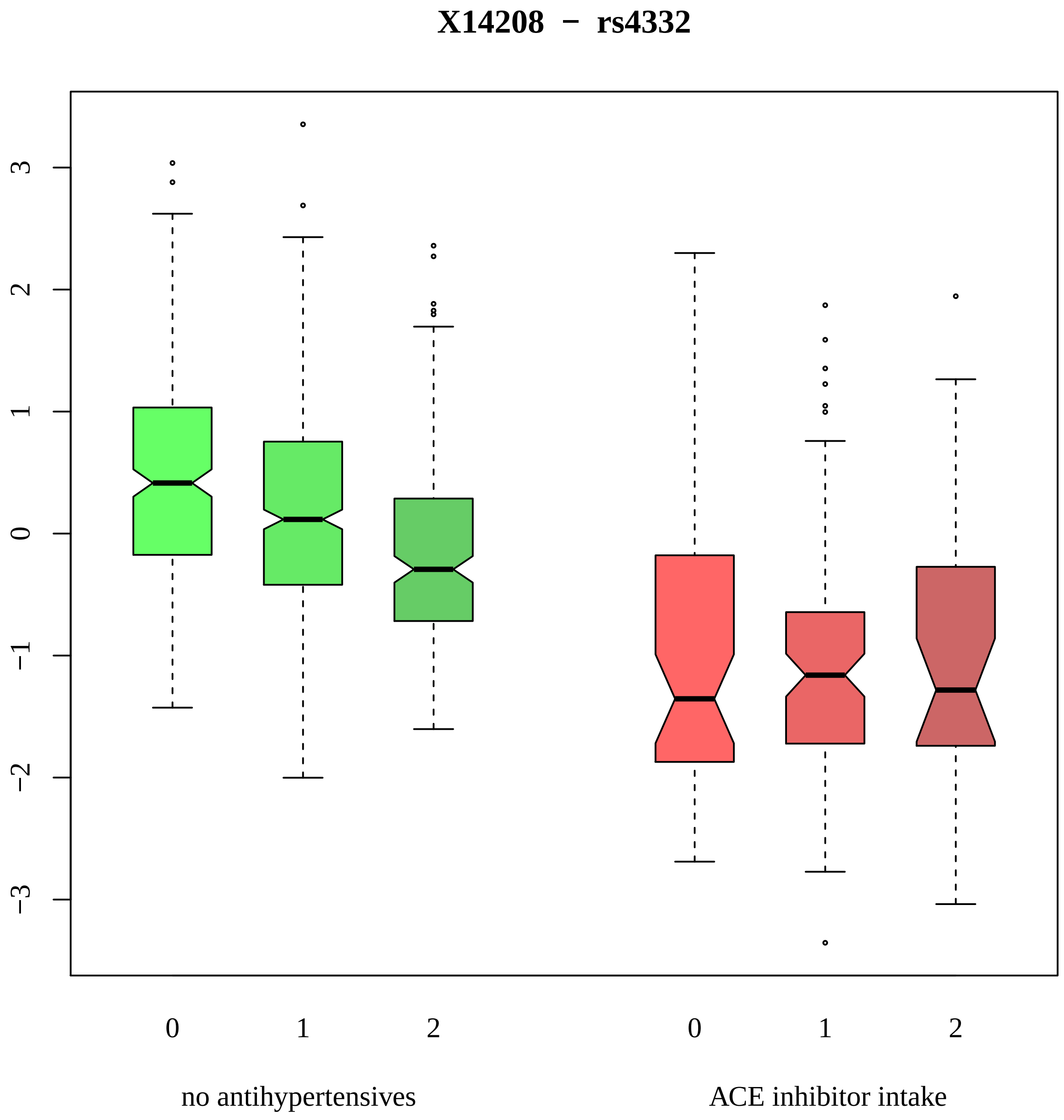

X14208 – rs4332
3
2
1
0
−1
−2
−3
0
1
2
0
1
2
no antihypertensives
ACE inhibitor intake

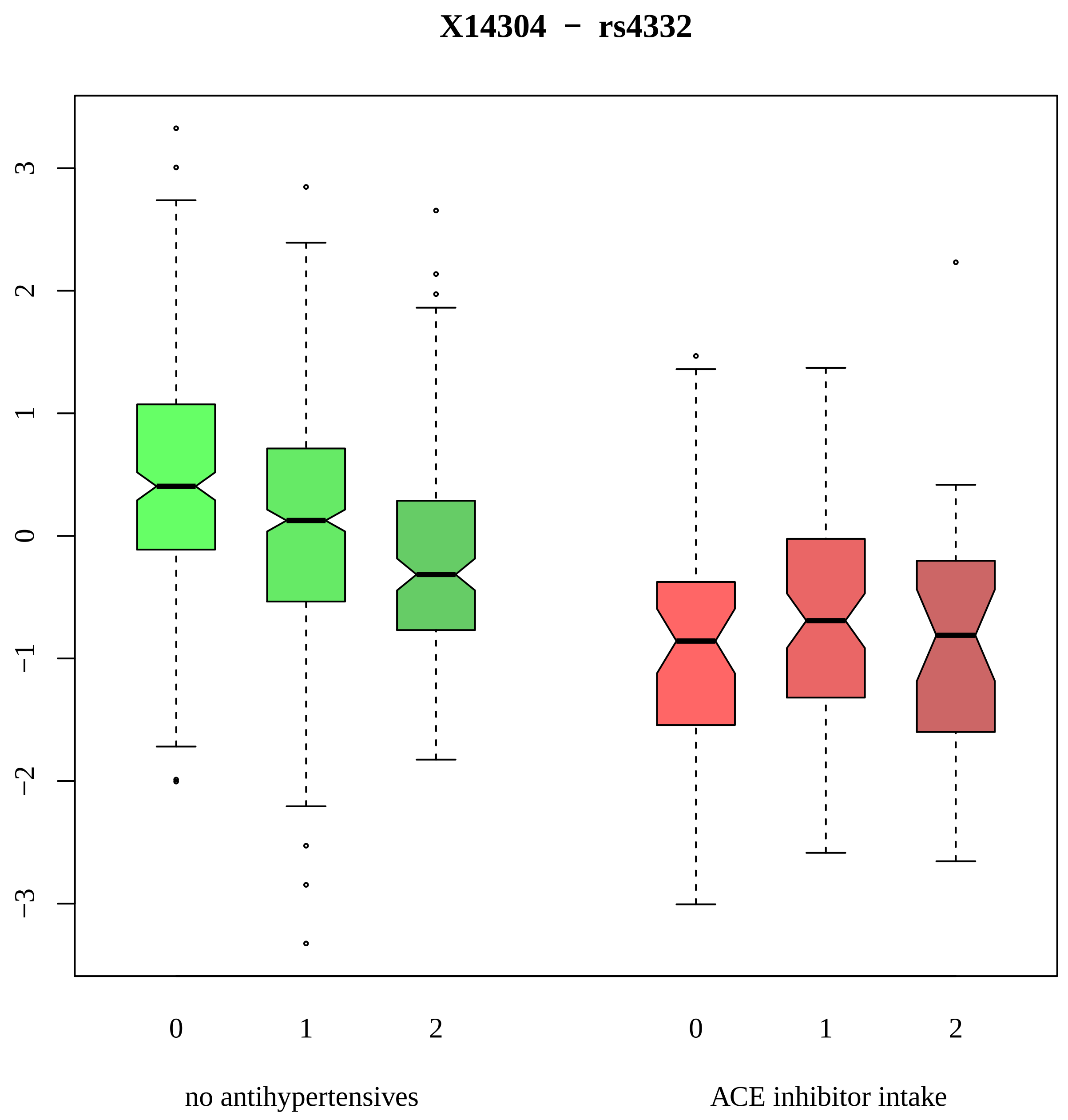
X14304 – rs4332
3
2
1
0
−1
−2
−3
0
1
2
0
1
2
no antihypertensives
ACE inhibitor intake

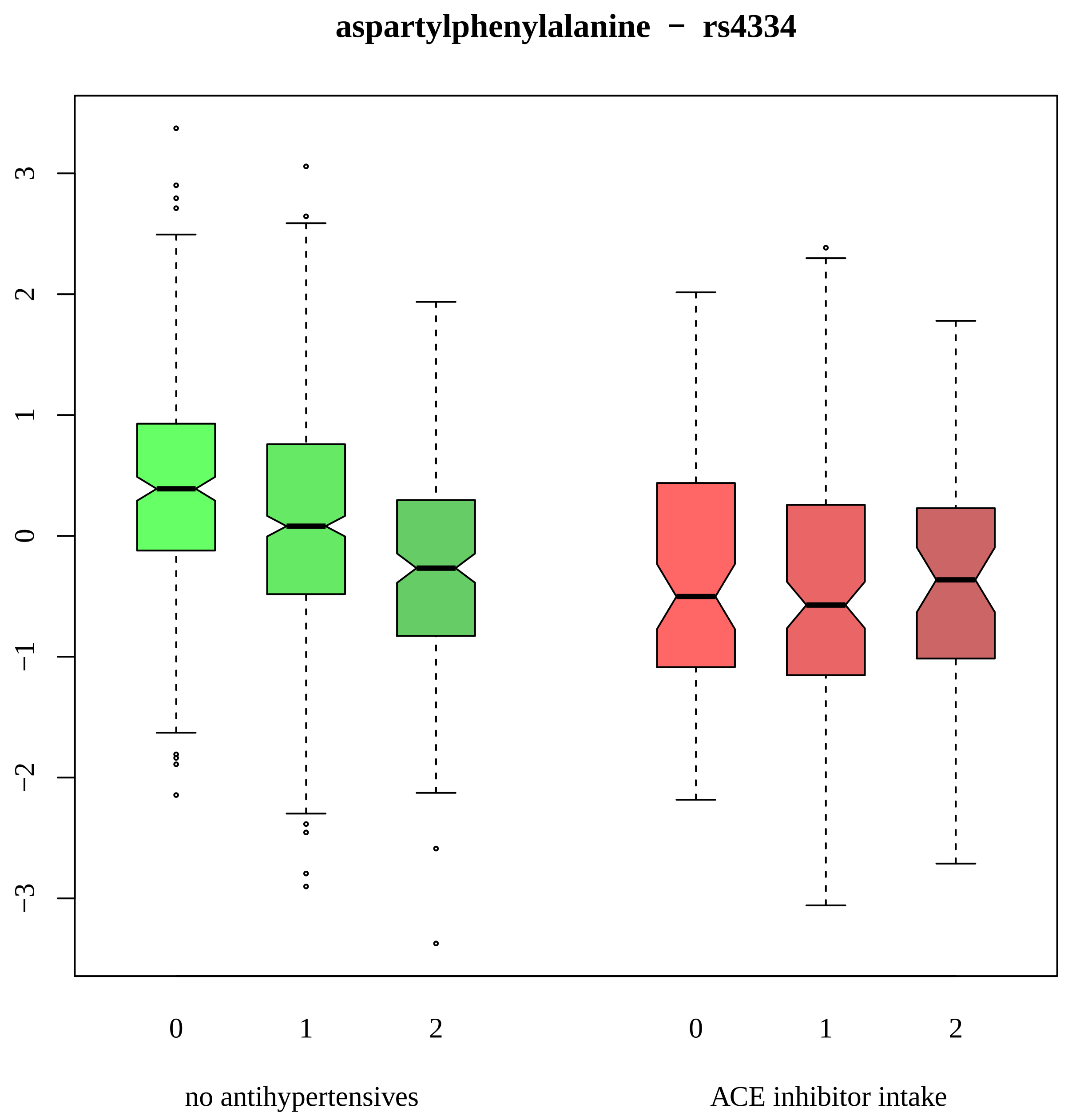

aspartylphenylalanine – rs4334
3
2
1
0
−1
−2
−3
0
1
2
0
1
2
no antihypertensives
ACE inhibitor intake

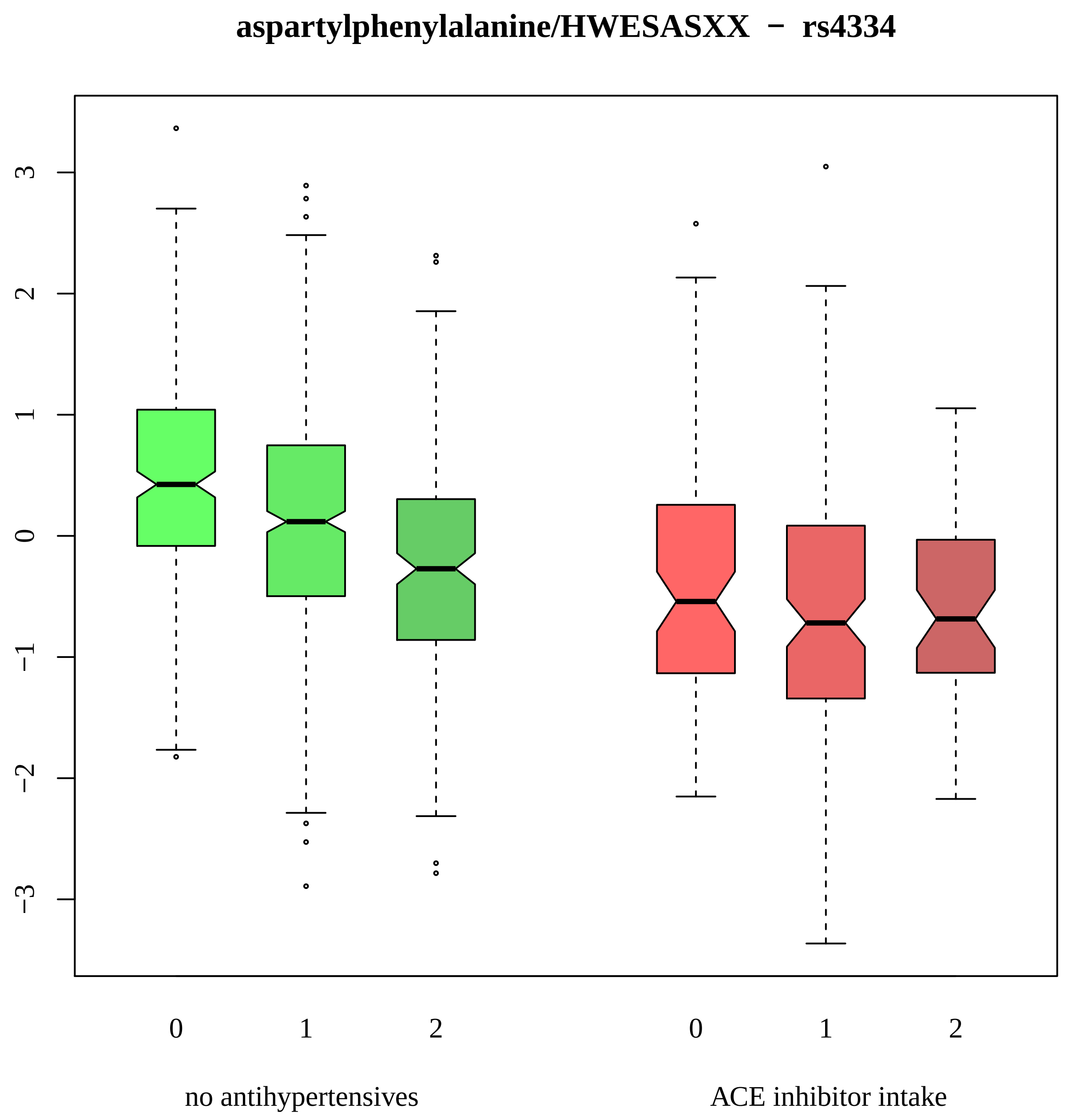

aspartylphenylalanine/HWESASXX – rs4334
3
2
1
0
−1
−2
−3
0
1
2
0
1
2
no antihypertensives
ACE inhibitor intake

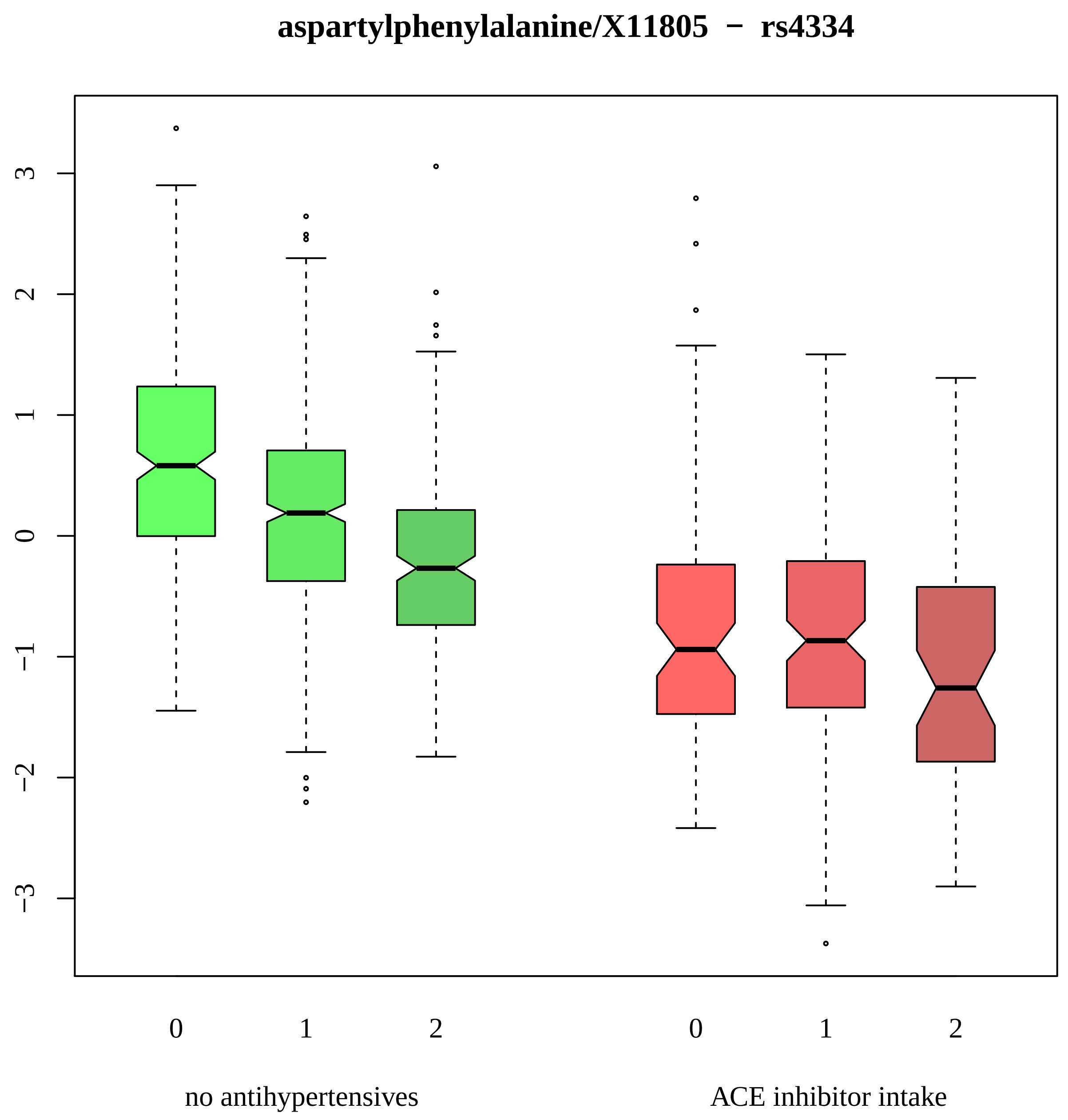

aspartylphenylalanine/X11805 – rs4334
3
2
1
0
−1
−2
−3
0
1
2
0
1
2
no antihypertensives
ACE inhibitor intake

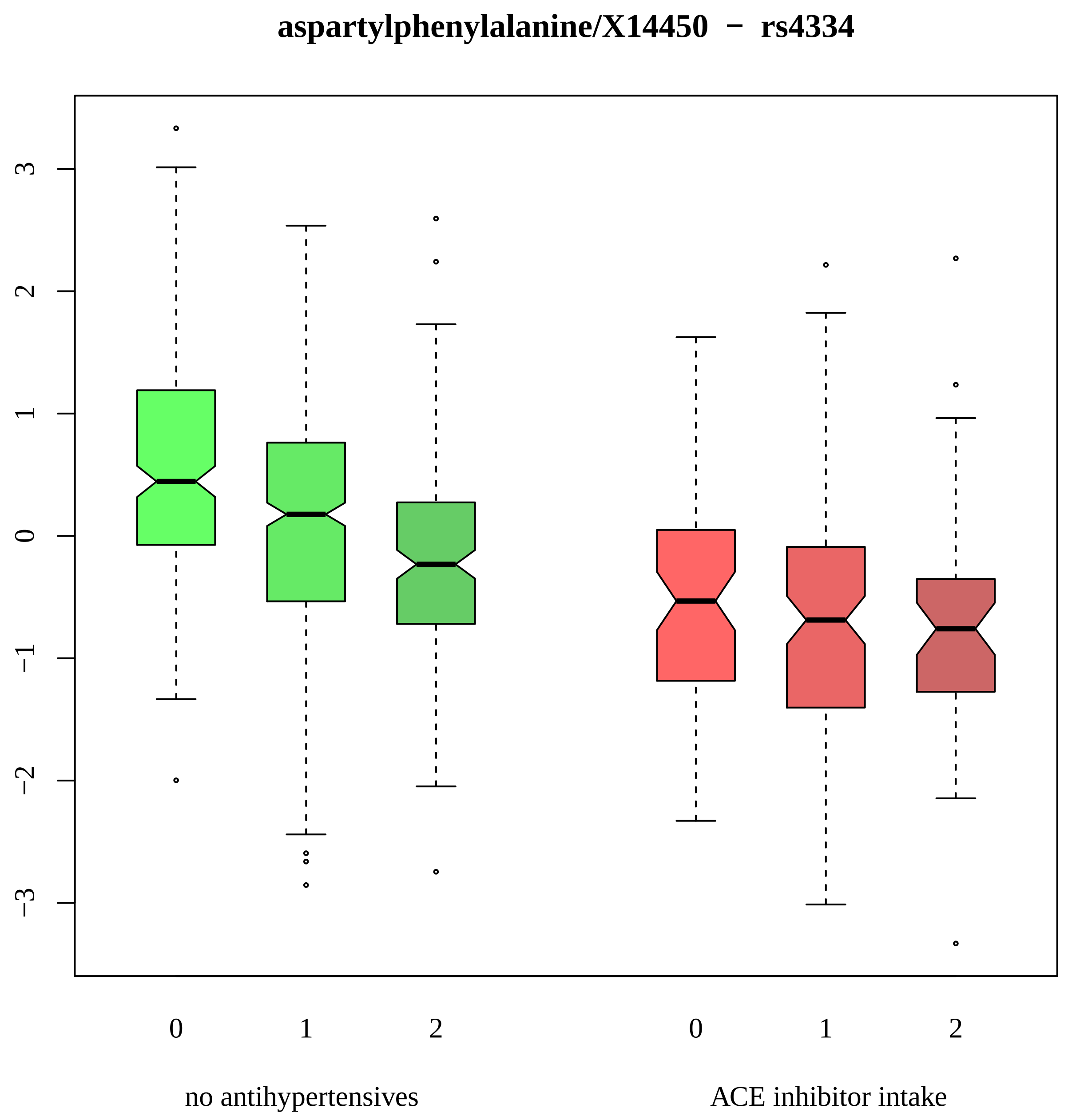
aspartylphenylalanine/X14450 – rs4334
3
2
1
0
−1
−2
−3
0
1
2
0
1
2
no antihypertensives
ACE inhibitor intake

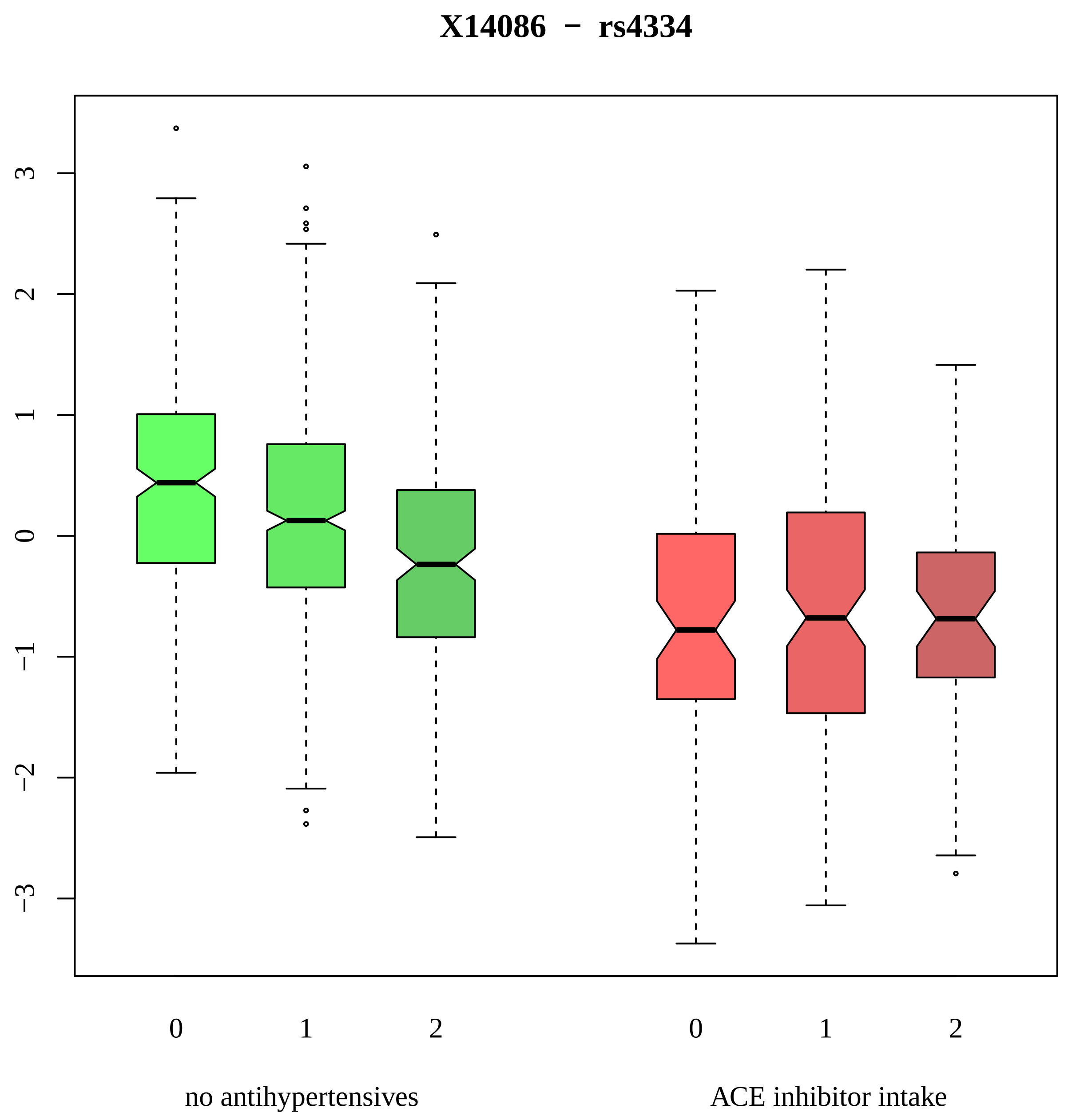

X14086 – rs4334
3
2
1
0
−1
−2
−3
0
1
2
0
1
2
no antihypertensives
ACE inhibitor intake

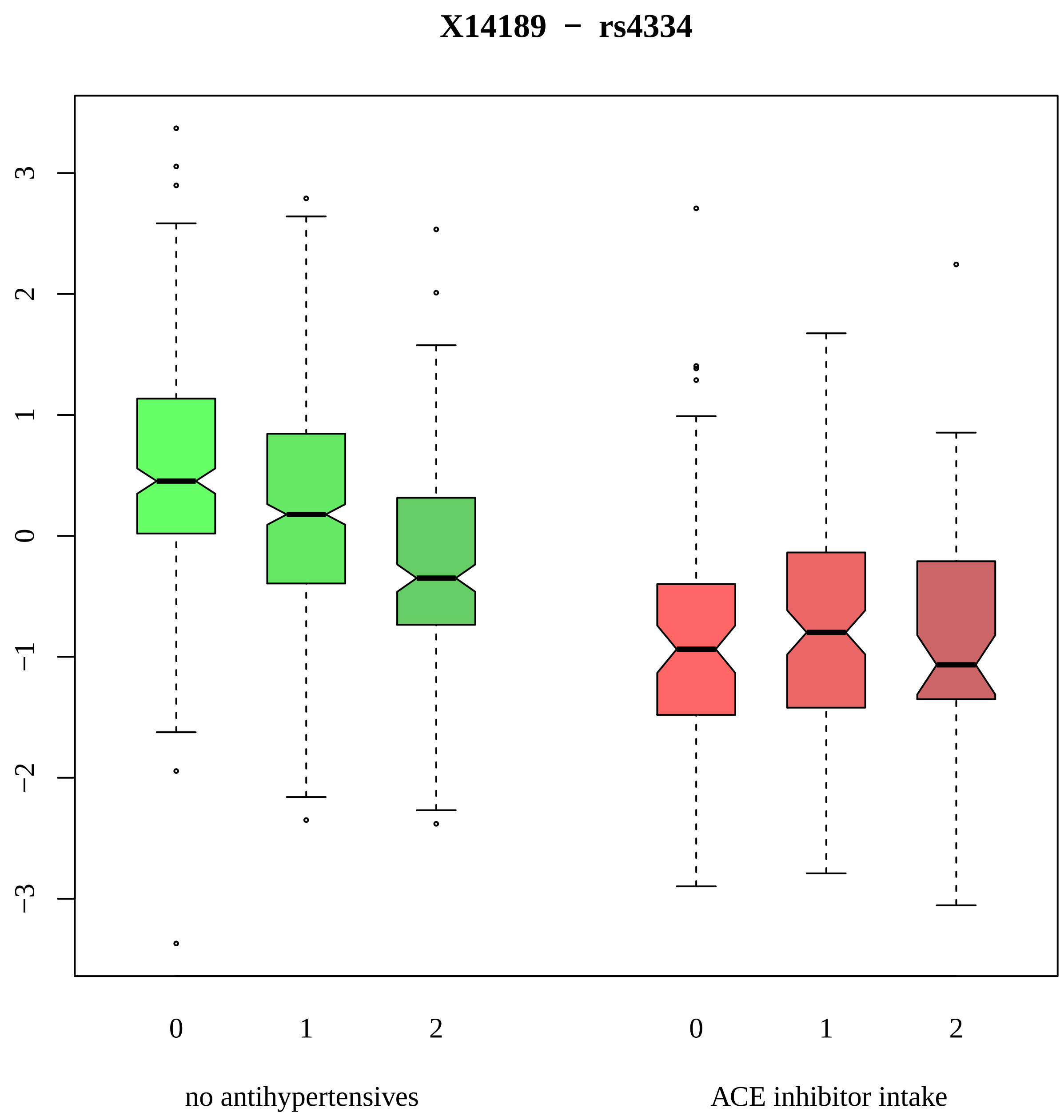

X14189 – rs4334
3
2
1
0
−1
−2
−3
0
1
2
0
1
2
no antihypertensives
ACE inhibitor intake

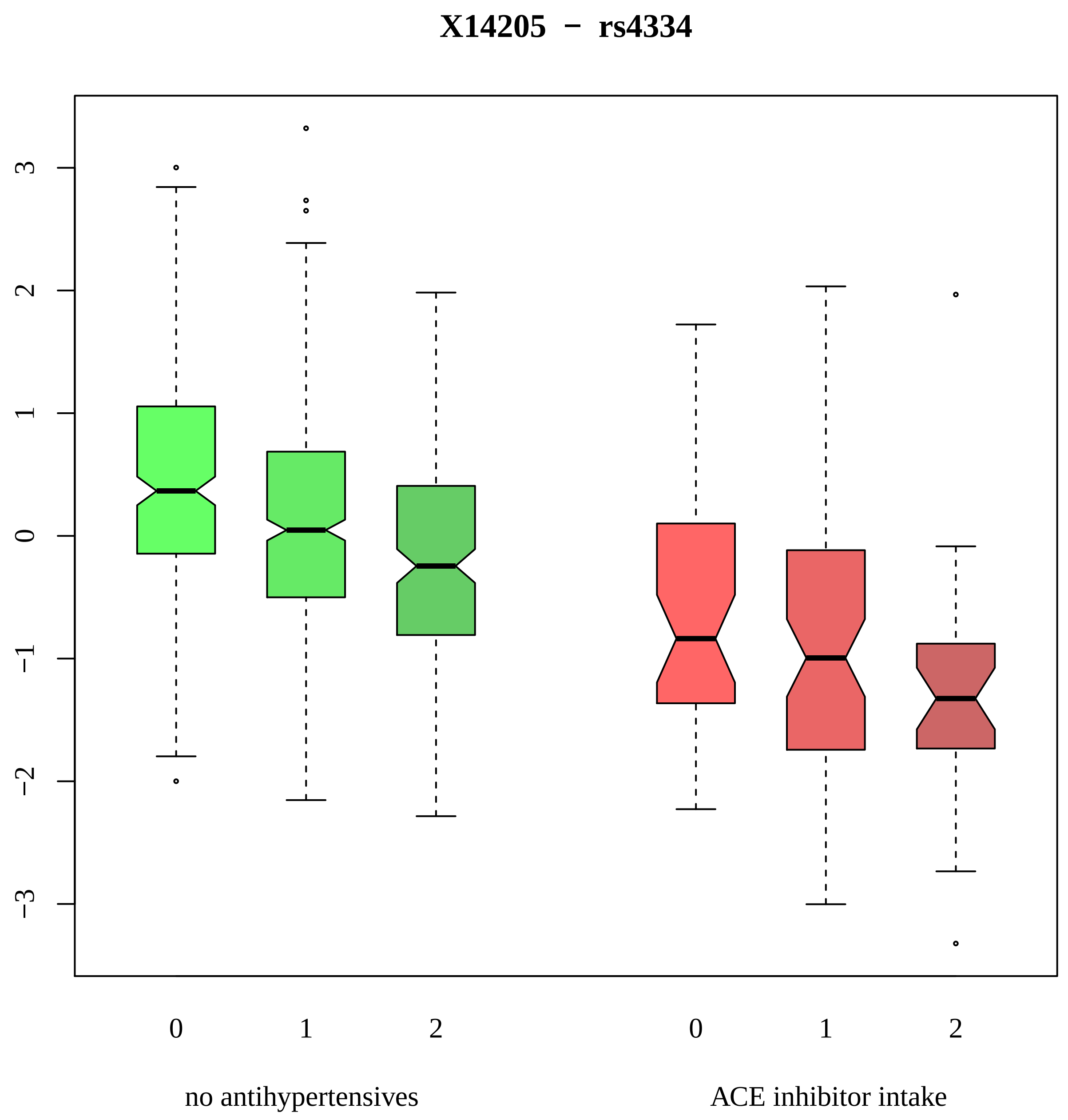

X14205 – rs4334
3
2
1
0
−1
−2
−3
0
1
2
0
1
2
no antihypertensives
ACE inhibitor intake

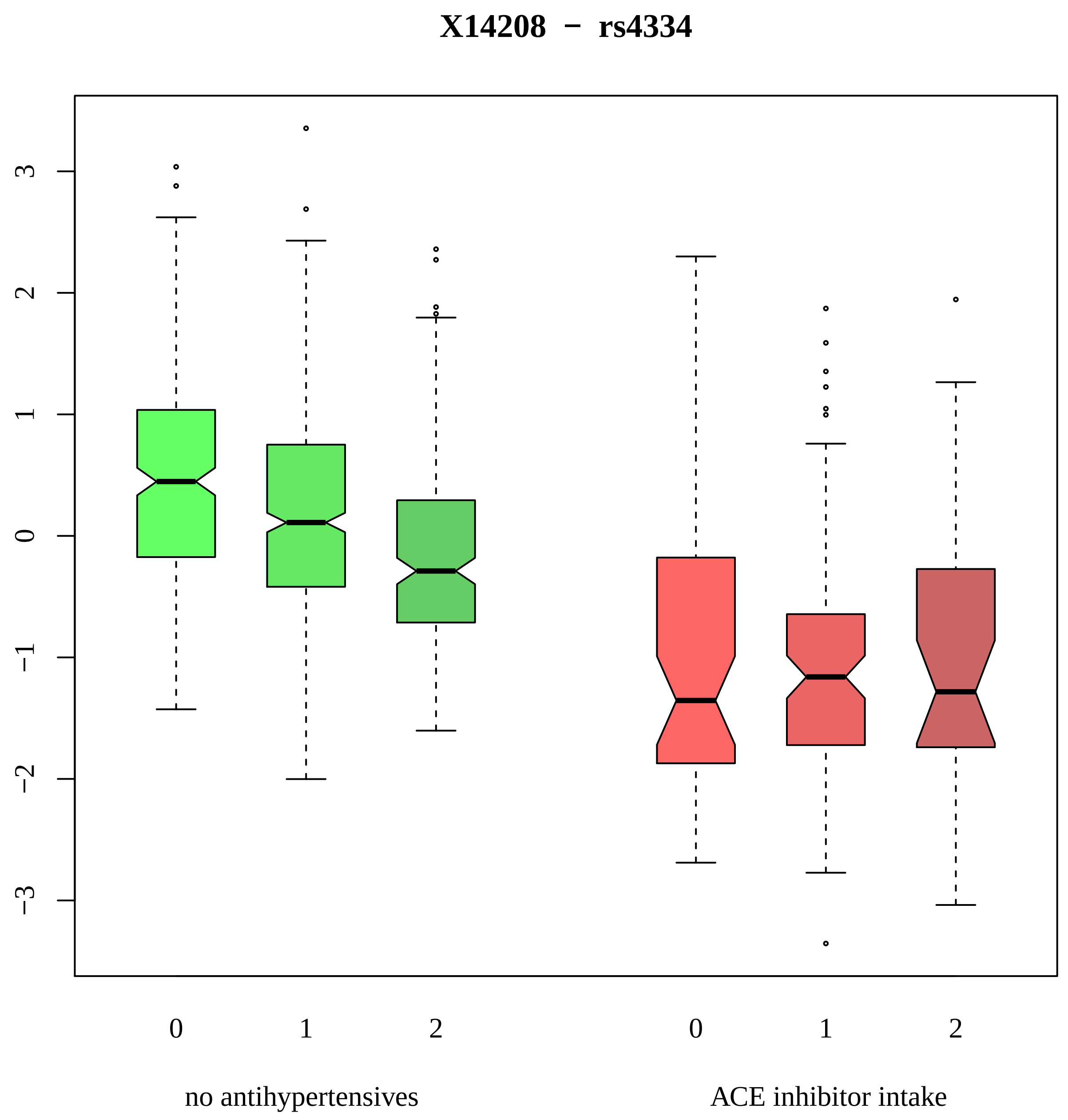
X14208 – rs4334
3
2
1
0
−1
−2
−3
0
1
2
0
1
2
no antihypertensives
ACE inhibitor intake

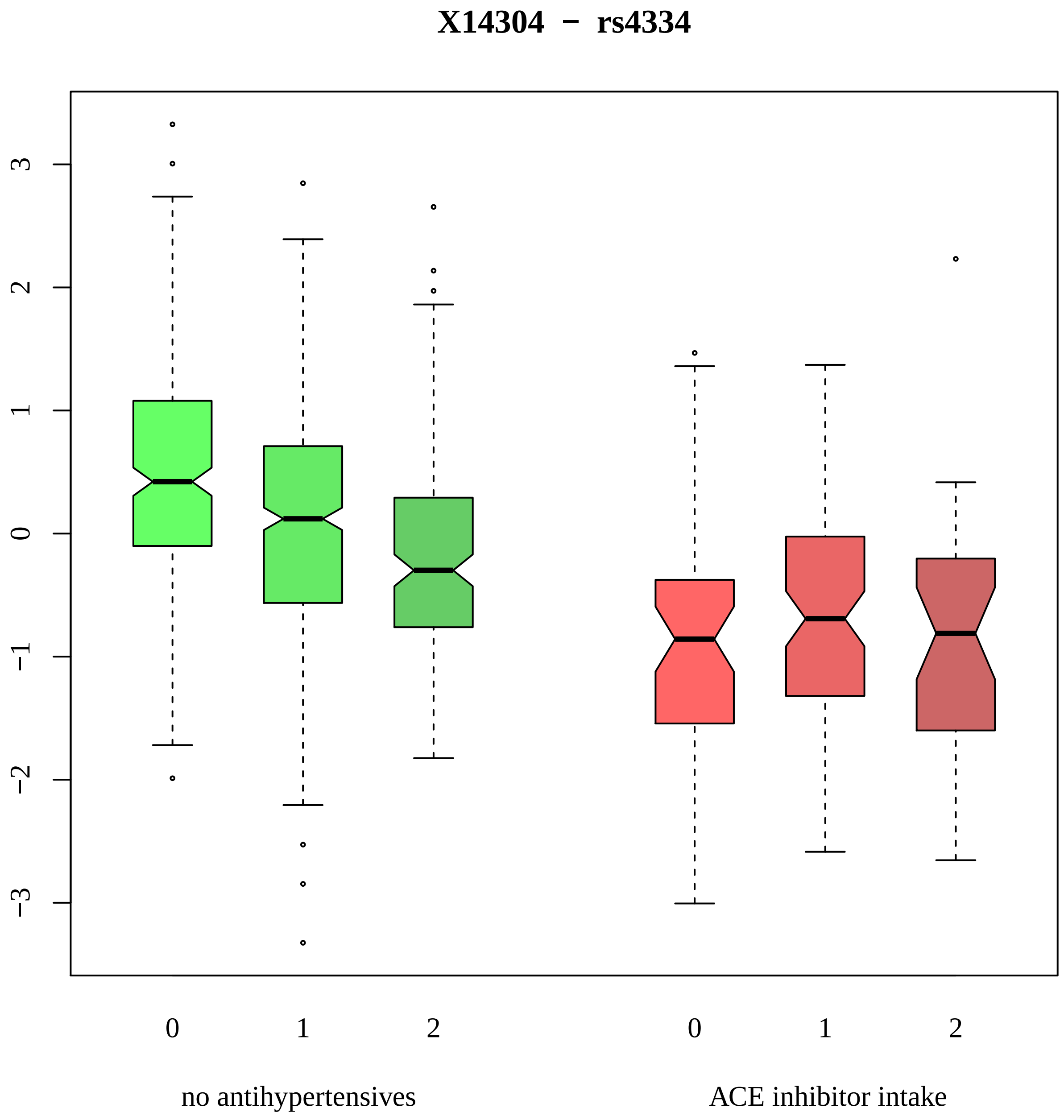

X14304 – rs4334
3
2
1
0
−1
−2
−3
0
1
2
0
1
2
no antihypertensives
ACE inhibitor intake

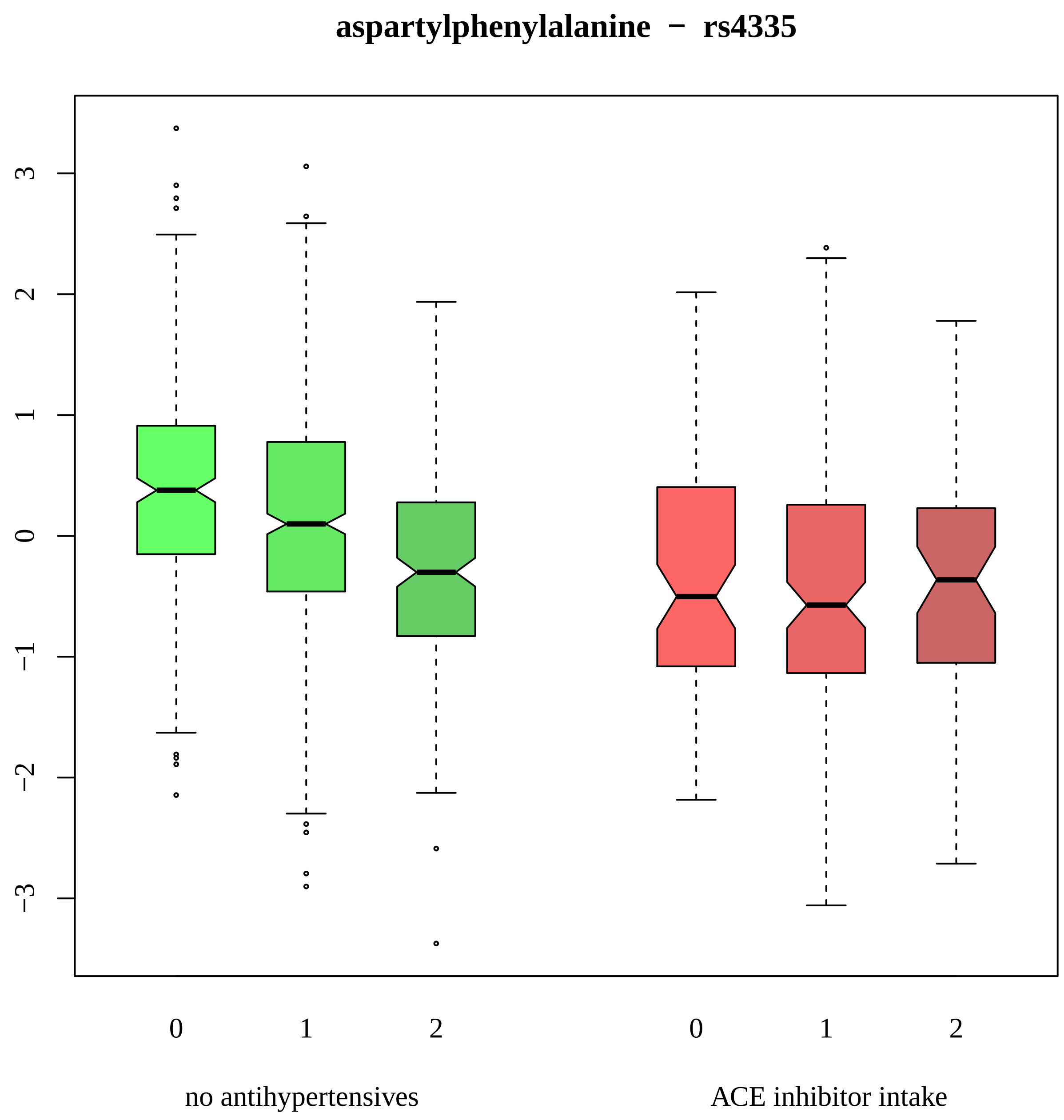
aspartylphenylalanine − rs4335
3
2
1
0
−1
−2
−3
0
1
2
0
1
2
no antihypertensives
ACE inhibitor intake

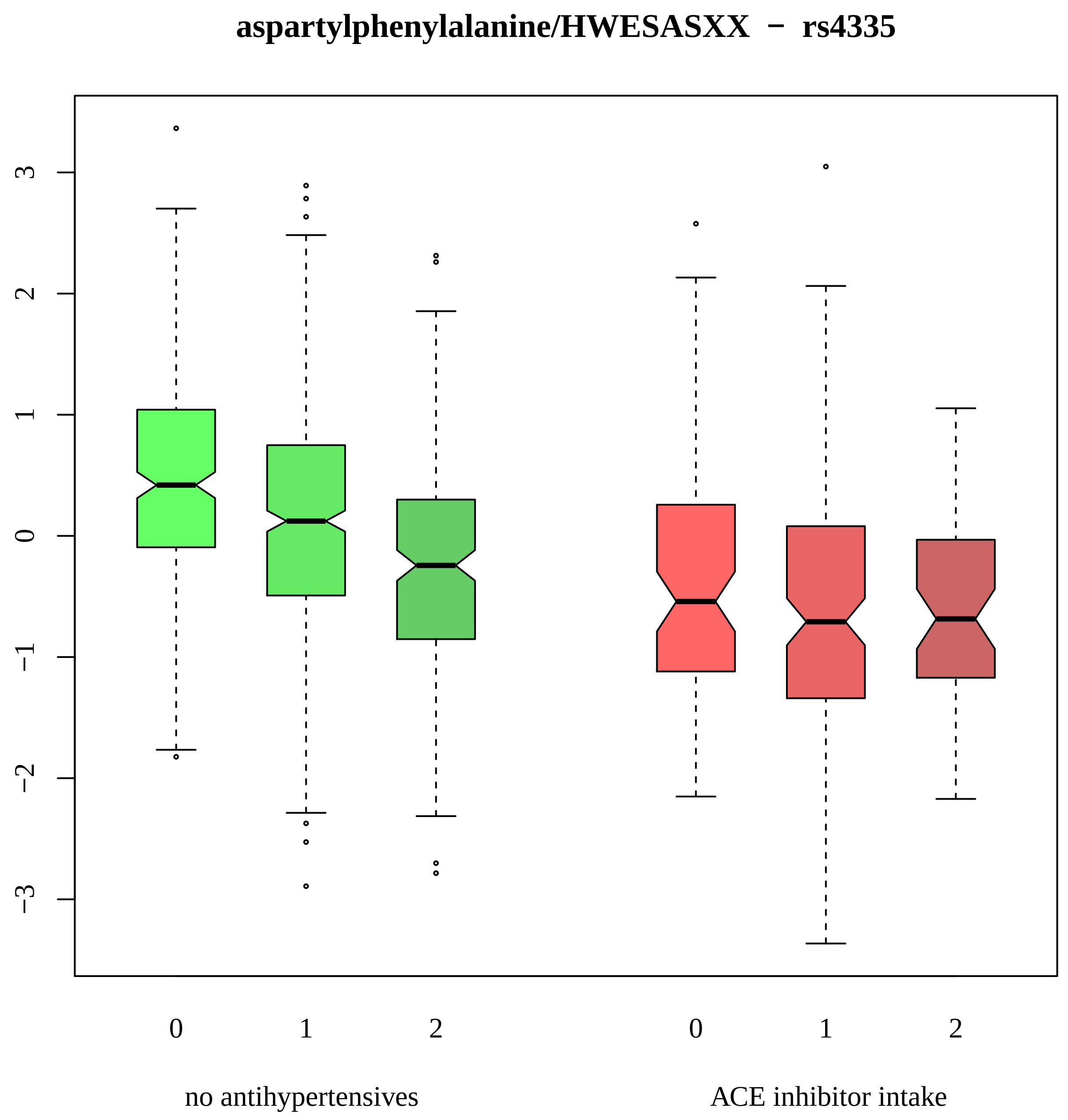

aspartylphenylalanine/HWESASXX – rs4335
3
2
1
0
−1
−2
−3
0
1
2
0
1
2
no antihypertensives
ACE inhibitor intake

aspartylphenylalanine/X11805 – rs4335

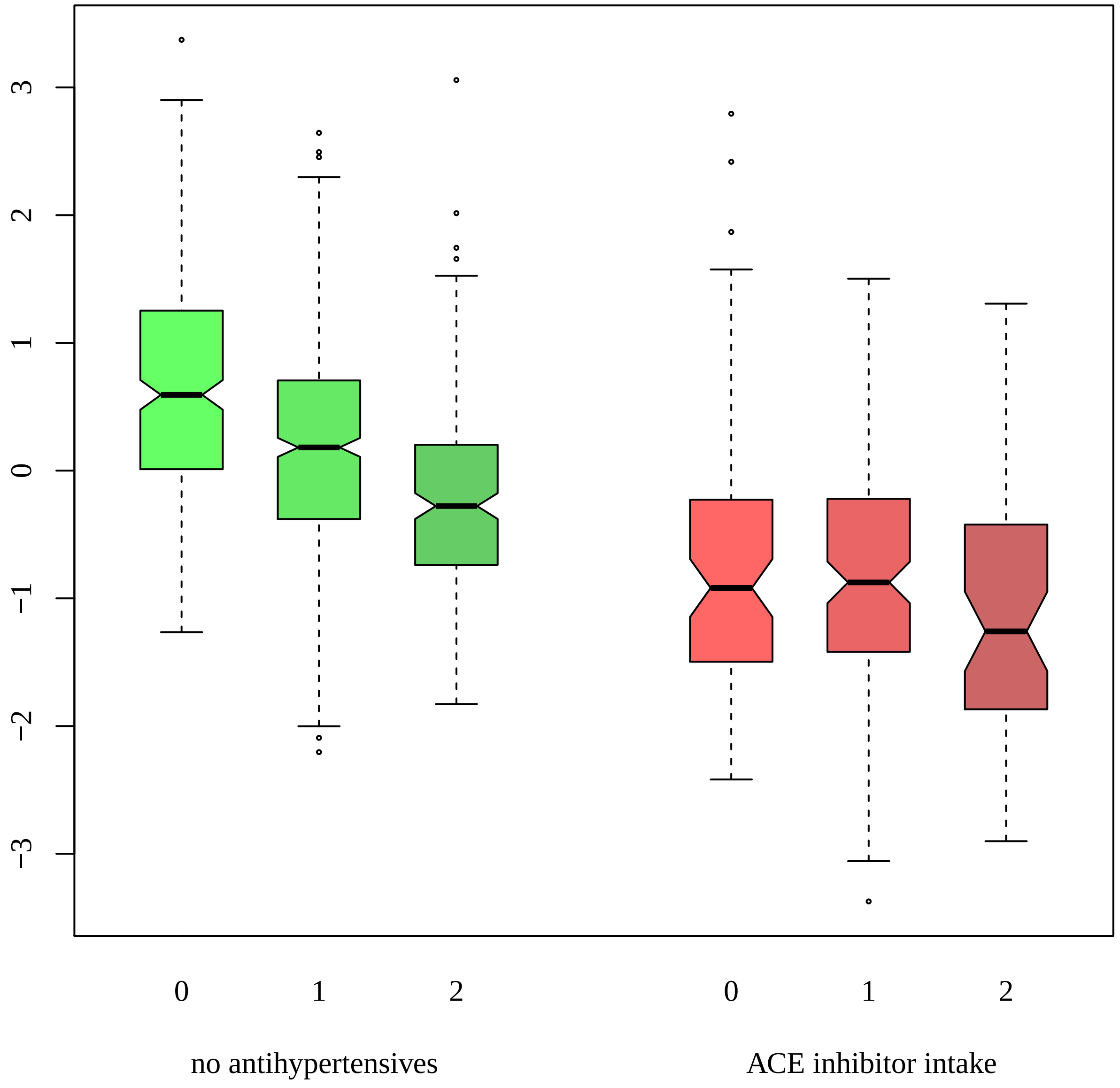

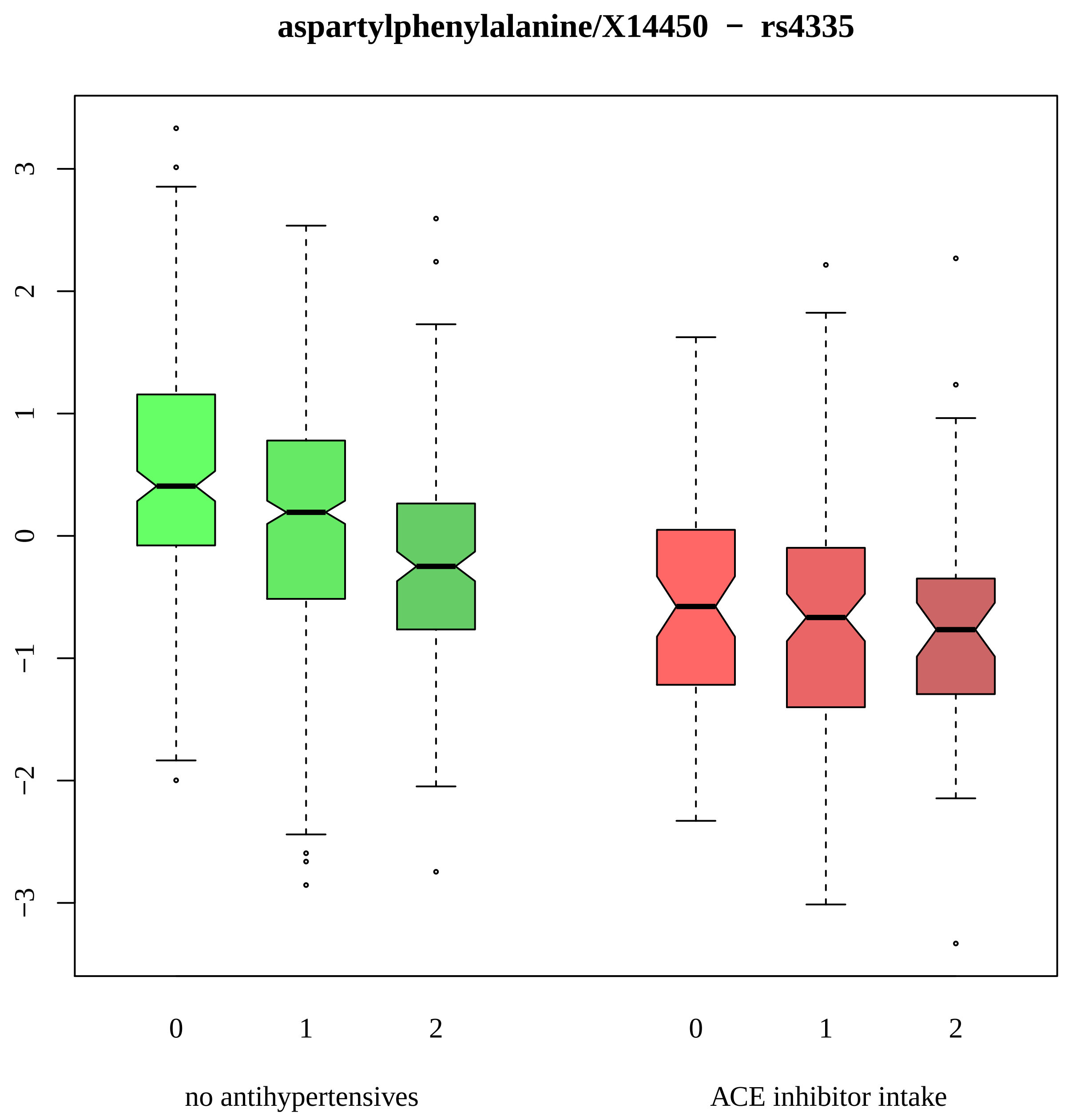

aspartylphenylalanine/X14450 – rs4335
3
2
1
0
−1
−2
−3
0
1
2
0
1
2
no antihypertensives
ACE inhibitor intake

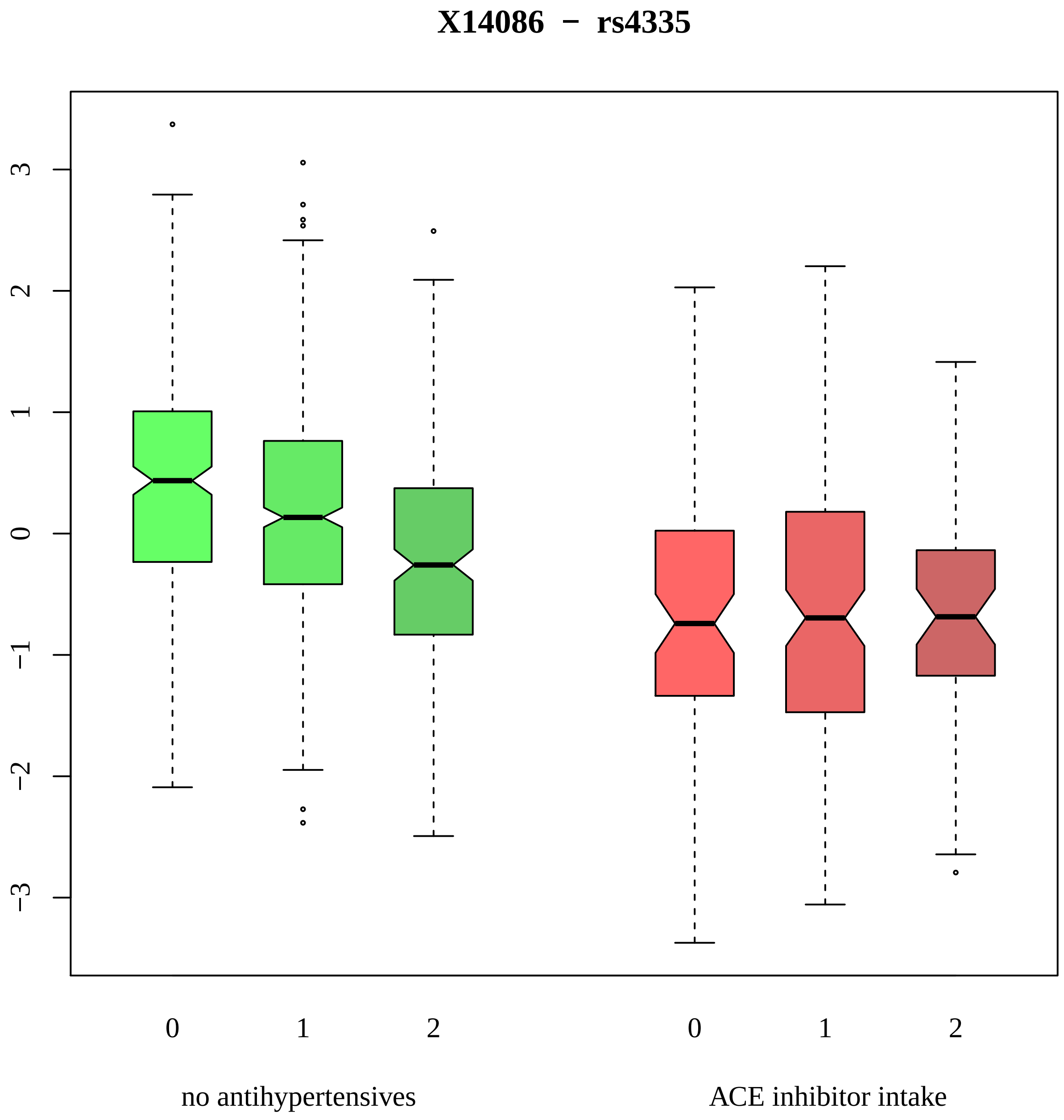

X14086 – rs4335
3
2
1
0
−1
−2
−3
0
1
2
0
1
2
no antihypertensives
ACE inhibitor intake

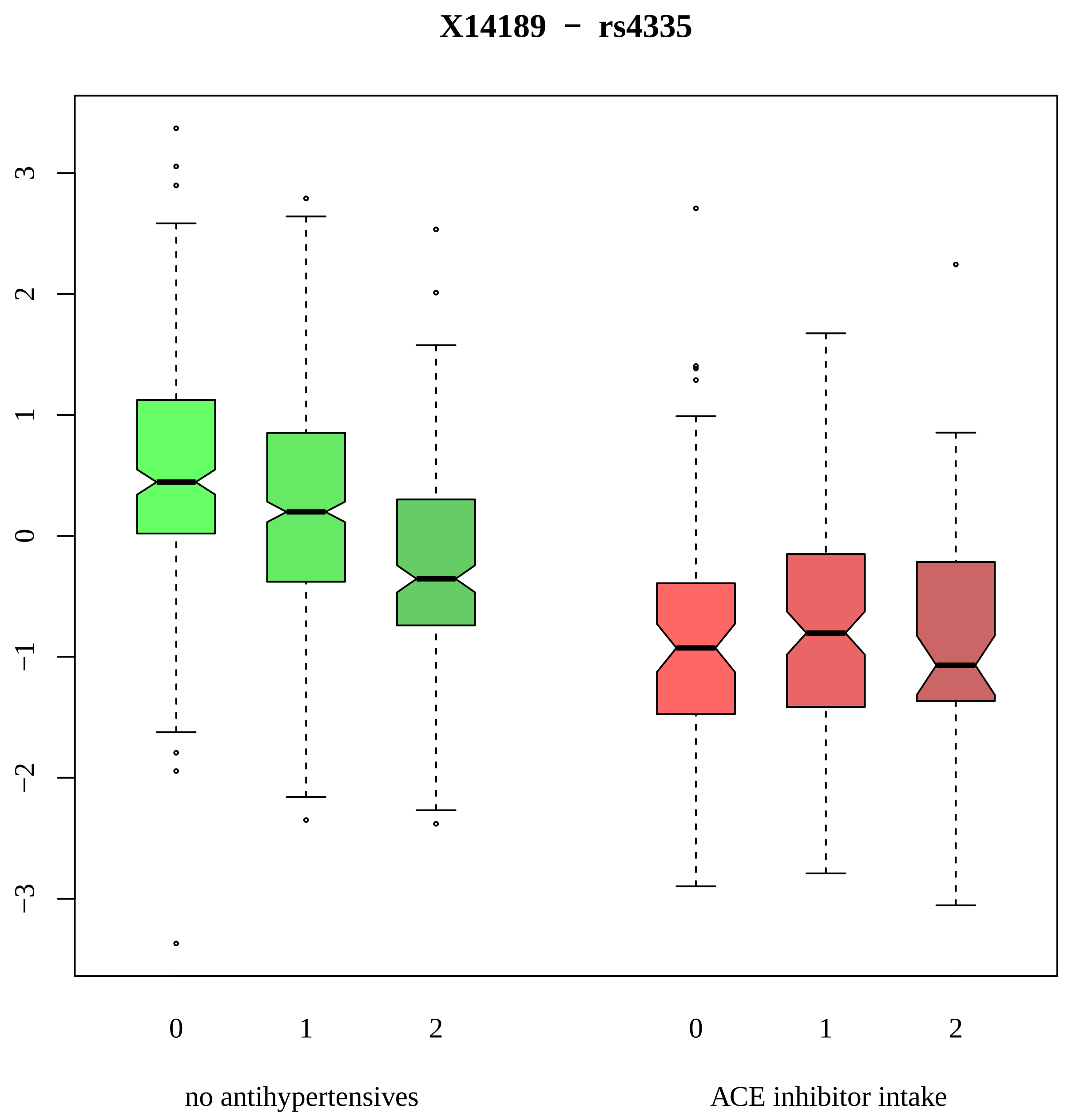

X14189 – rs4335
3
2
1
0
−1
−2
−3
0
1
2
0
1
2
no antihypertensives
ACE inhibitor intake

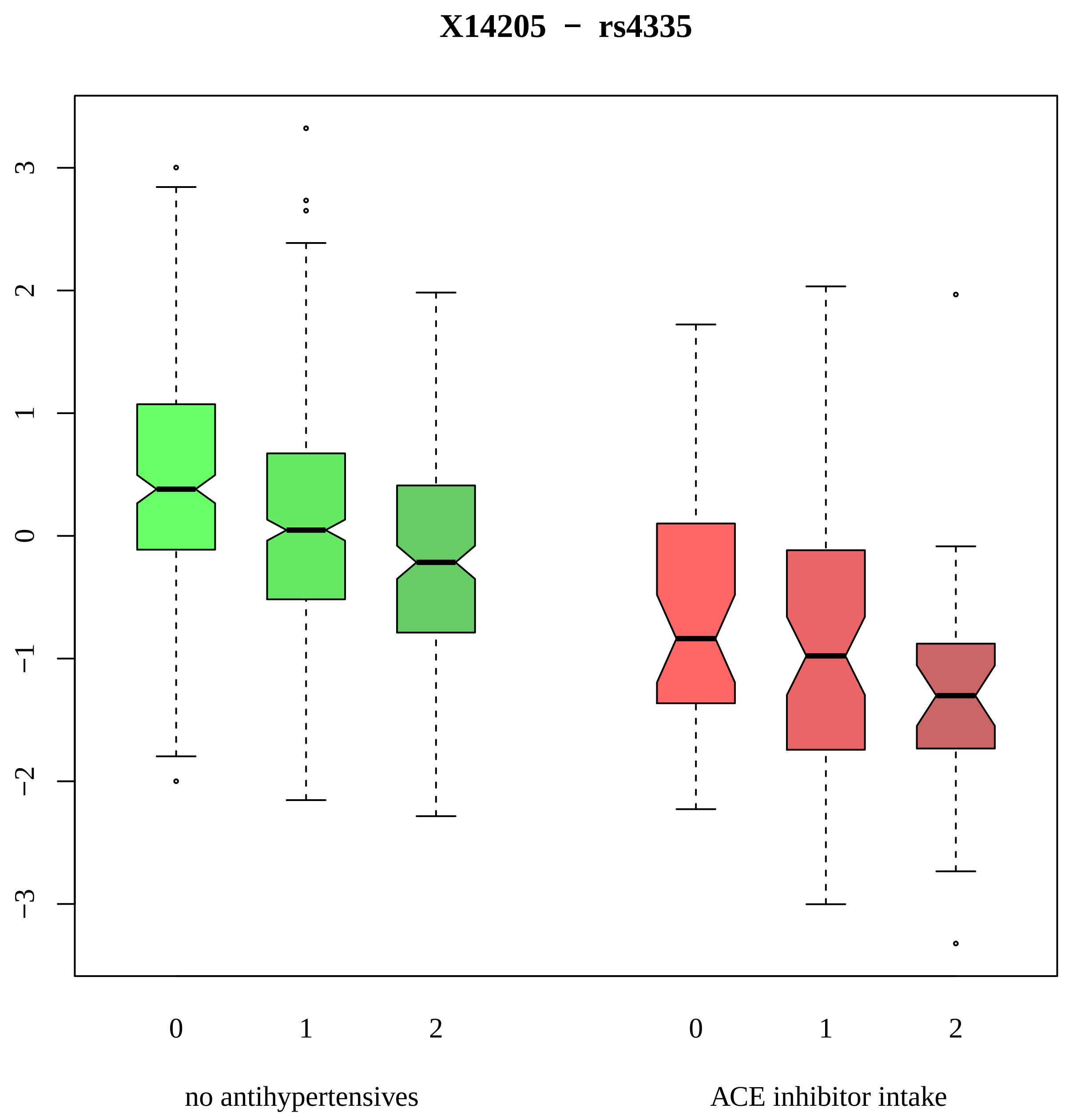

X14205 – rs4335
3
2
1
0
−1
−2
−3
0
1
2
0
1
2
no antihypertensives
ACE inhibitor intake

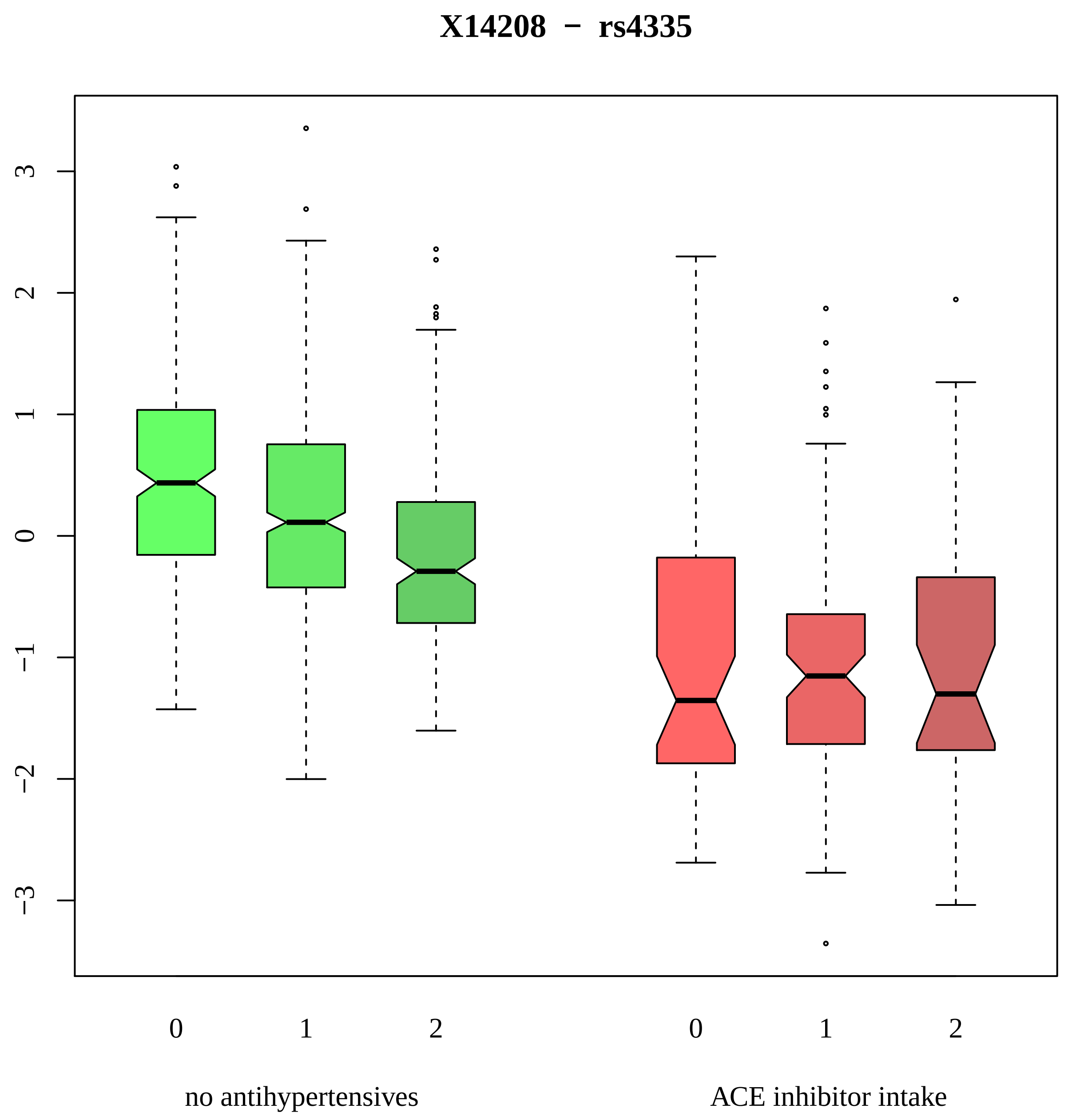
X14208 – rs4335
3
2
1
0
−1
−2
−3
0
1
2
0
1
2
no antihypertensives
ACE inhibitor intake

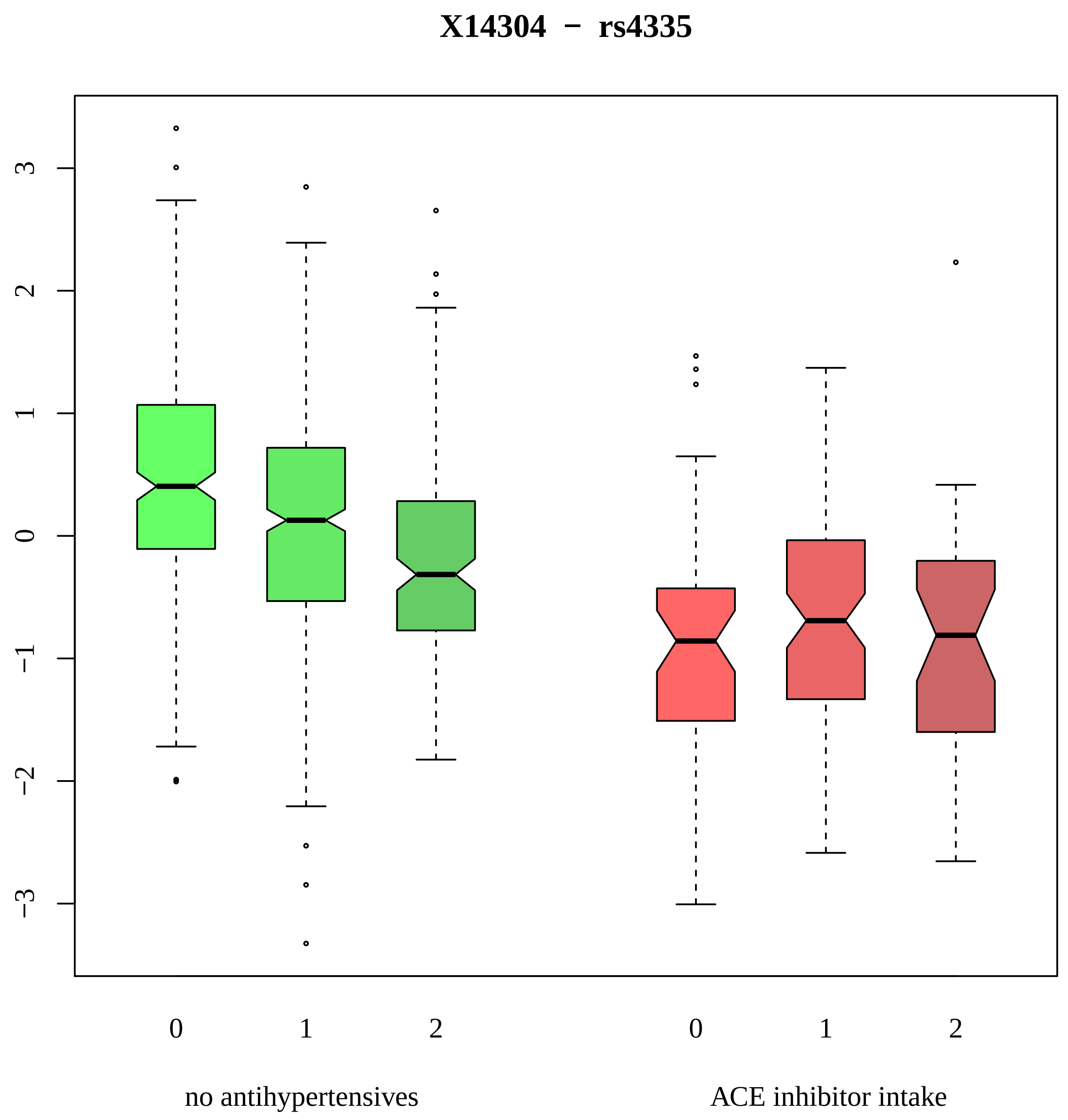
X14304 – rs4335
3
2
1
0
−1
−2
−3
0
1
2
0
1
2
no antihypertensives
ACE inhibitor intake

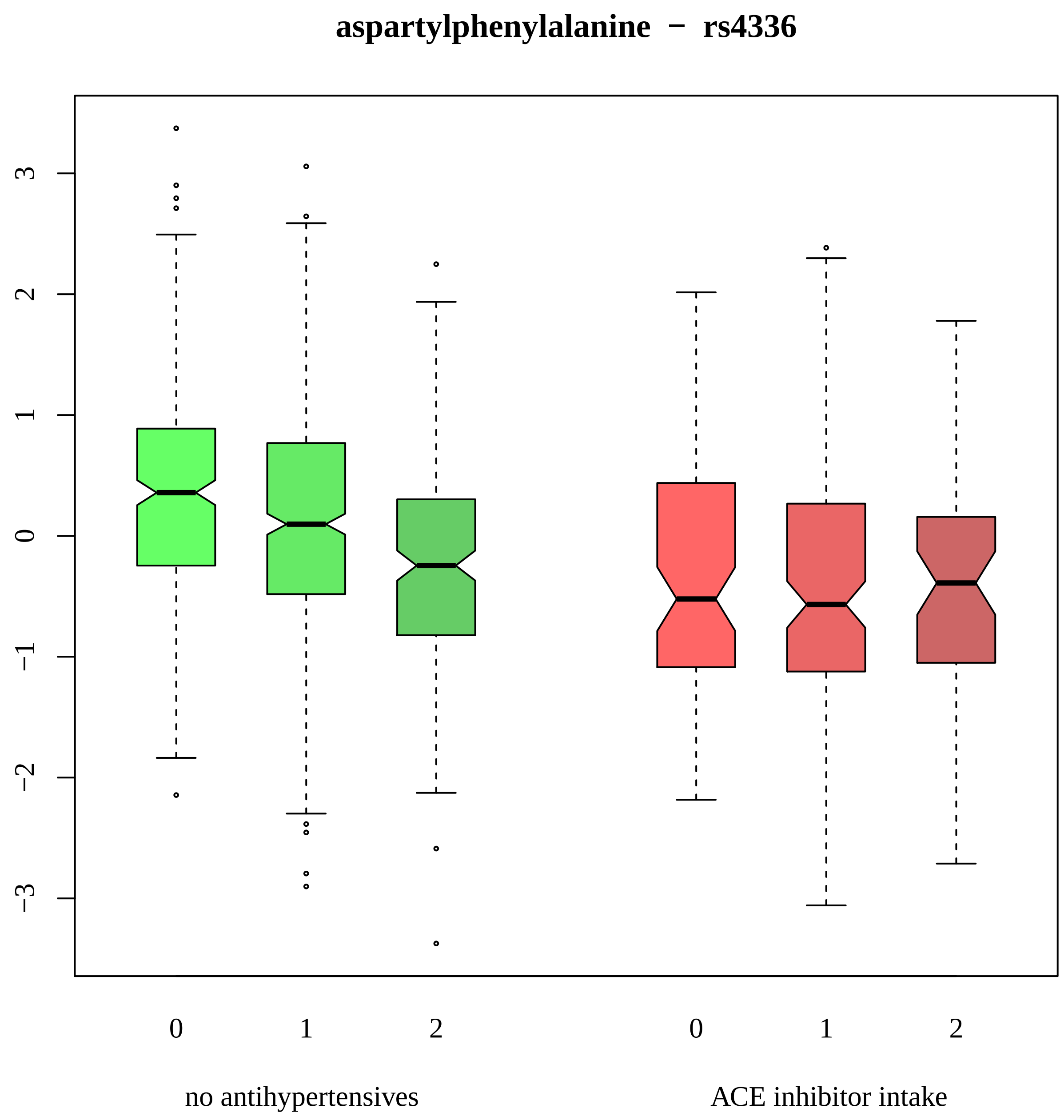

aspartylphenylalanine – rs4336
3
2
1
0
−1
−2
−3
0
1
2
0
1
2
no antihypertensives
ACE inhibitor intake

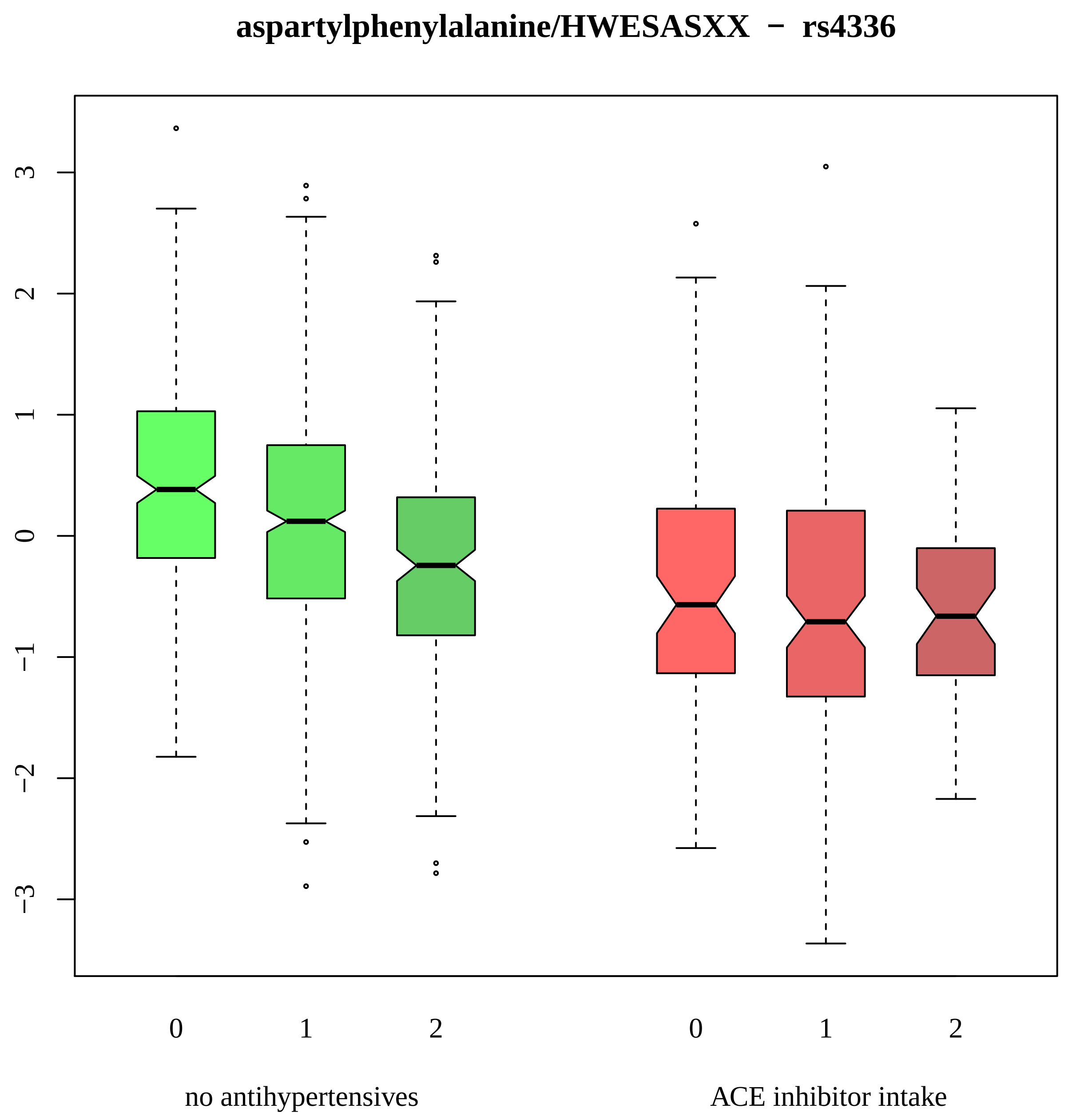
aspartylphenylalanine/HWESASXX – rs4336
3
2
1
0
−1
−2
−3
0
1
2
0
1
2
no antihypertensives
ACE inhibitor intake

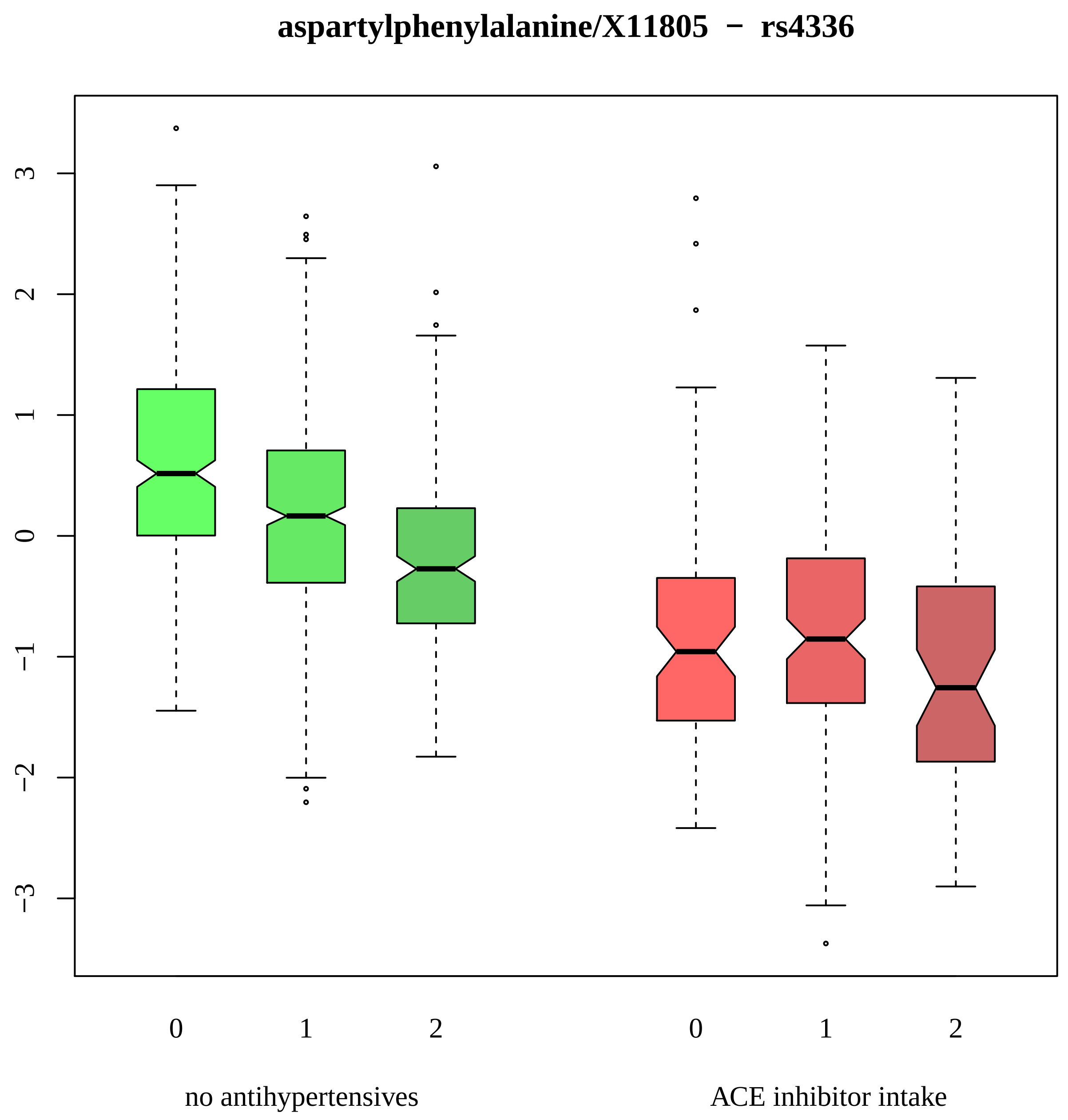

aspartylphenylalanine/X11805 – rs4336
3
2
1
0
−1
−2
−3
0
1
2
0
1
2
no antihypertensives
ACE inhibitor intake

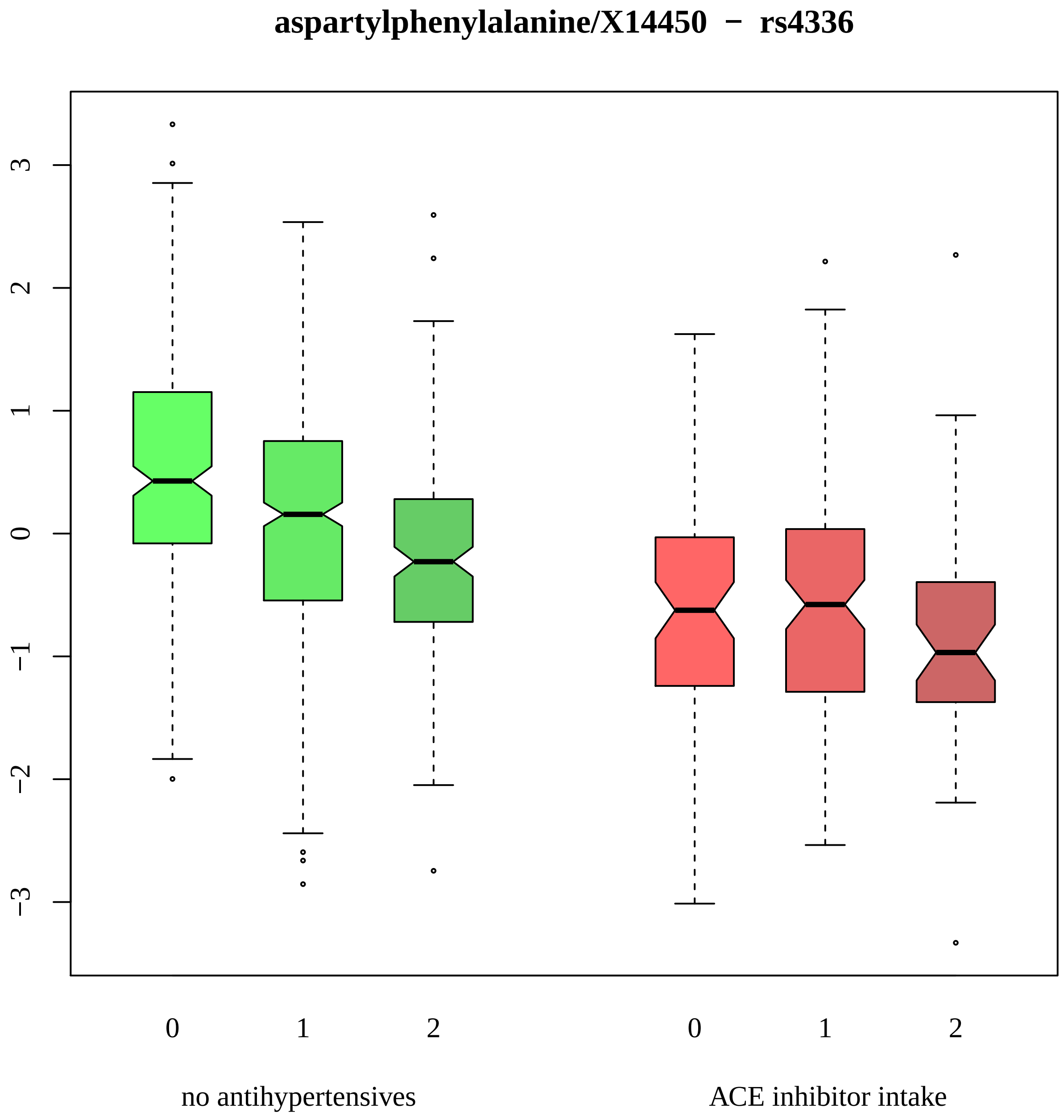

aspartylphenylalanine/X14450 – rs4336
3
2
1
0
−1
−2
−3
0
1
2
0
1
2
no antihypertensives
ACE inhibitor intake

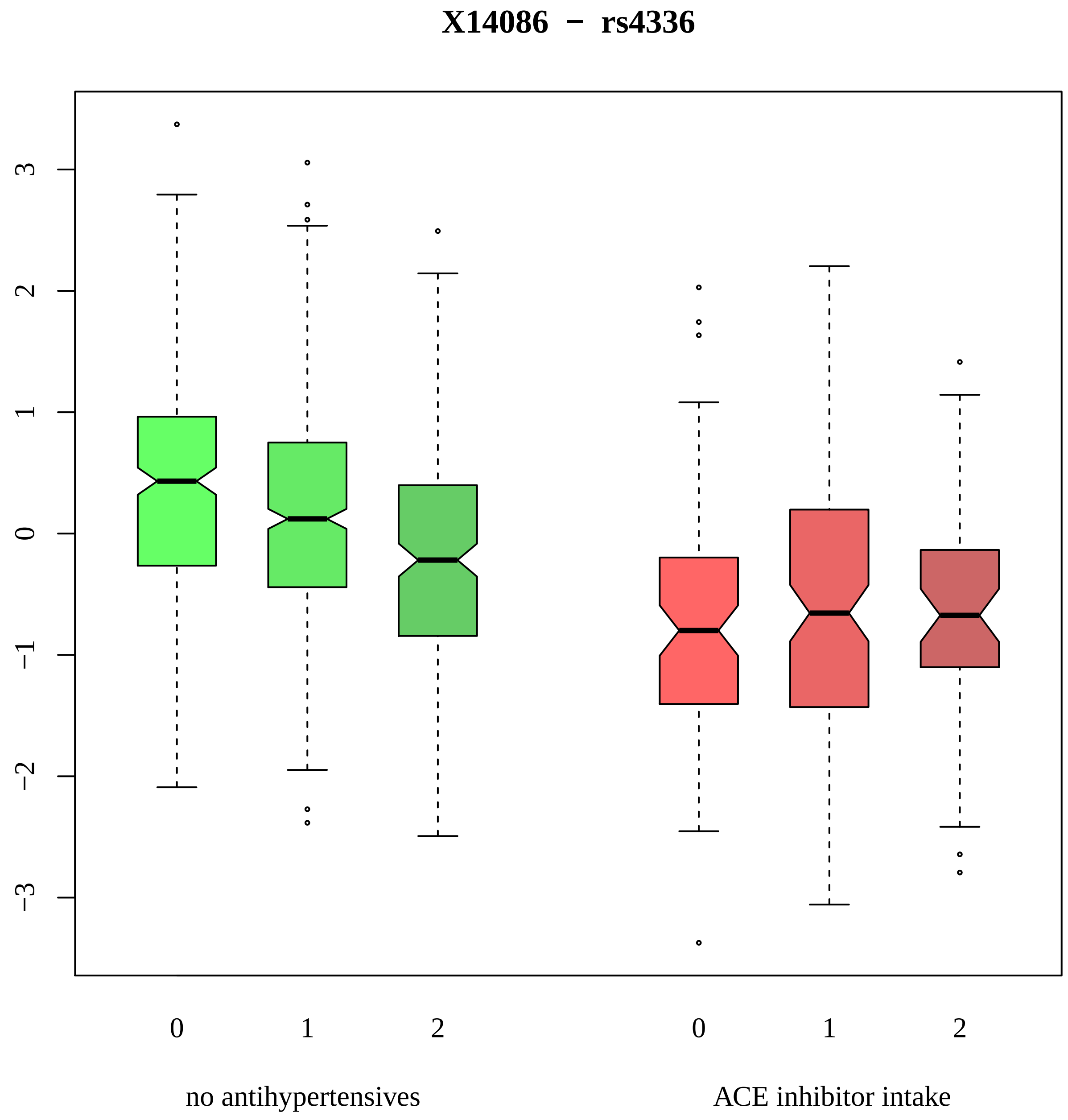

X14086 – rs4336
3
2
1
0
−1
−2
−3
0
1
2
0
1
2
no antihypertensives
ACE inhibitor intake

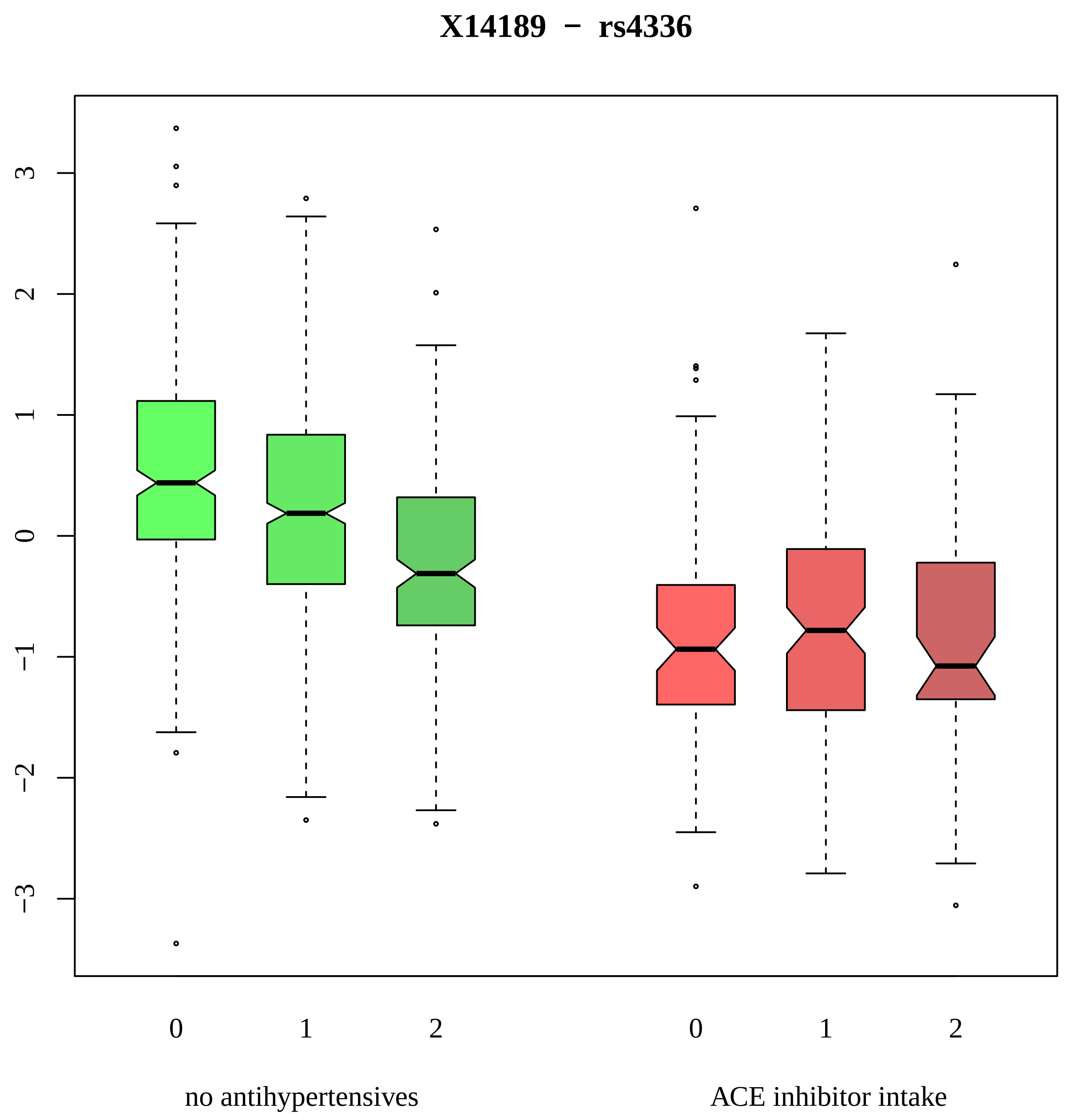
X14189 – rs4336
3
2
1
0
−1
−2
−3
0
1
2
0
1
2
no antihypertensives
ACE inhibitor intake

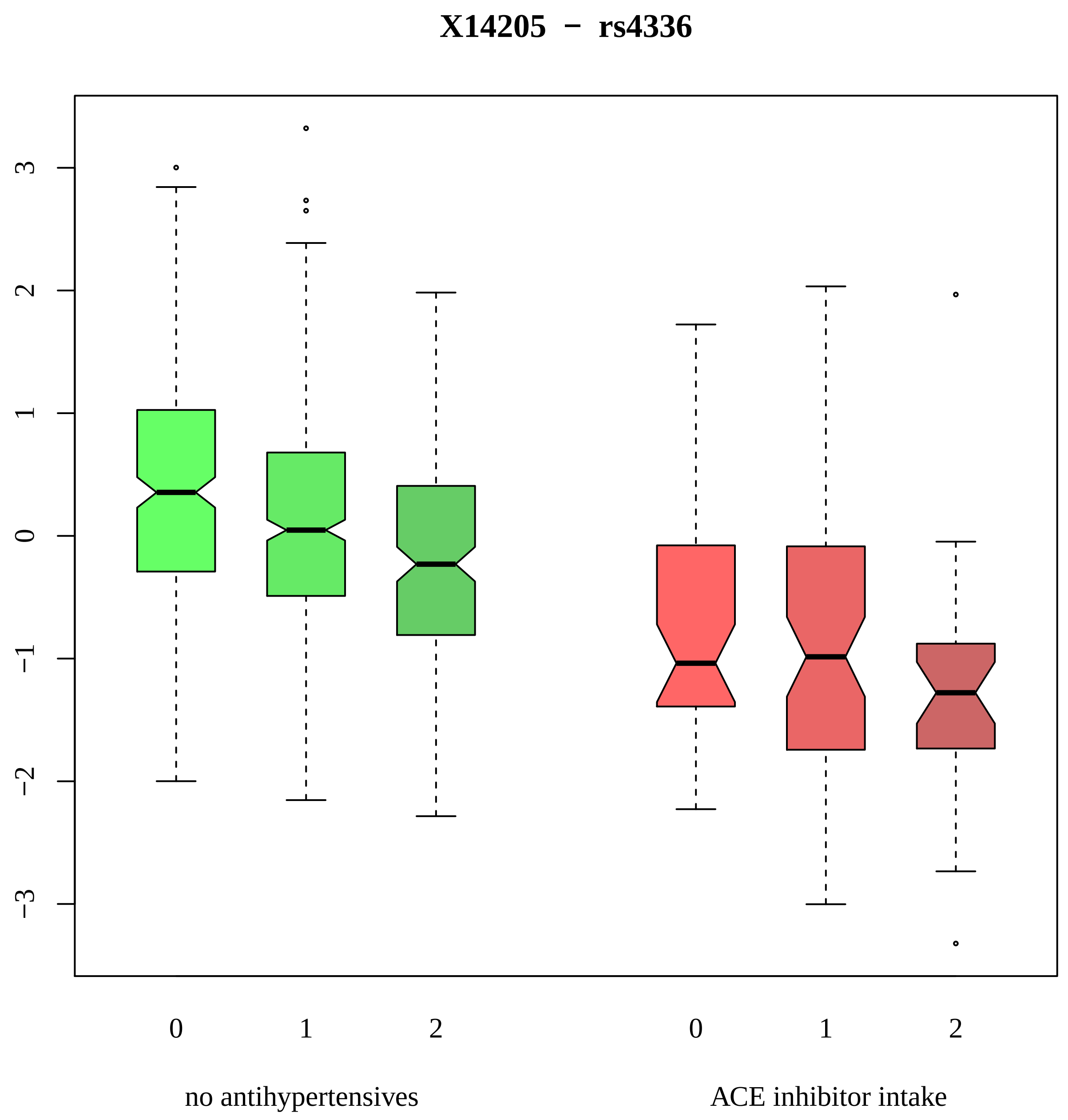

X14205 – rs4336
3
2
1
0
−1
−2
−3
0
1
2
0
1
2
no antihypertensives
ACE inhibitor intake

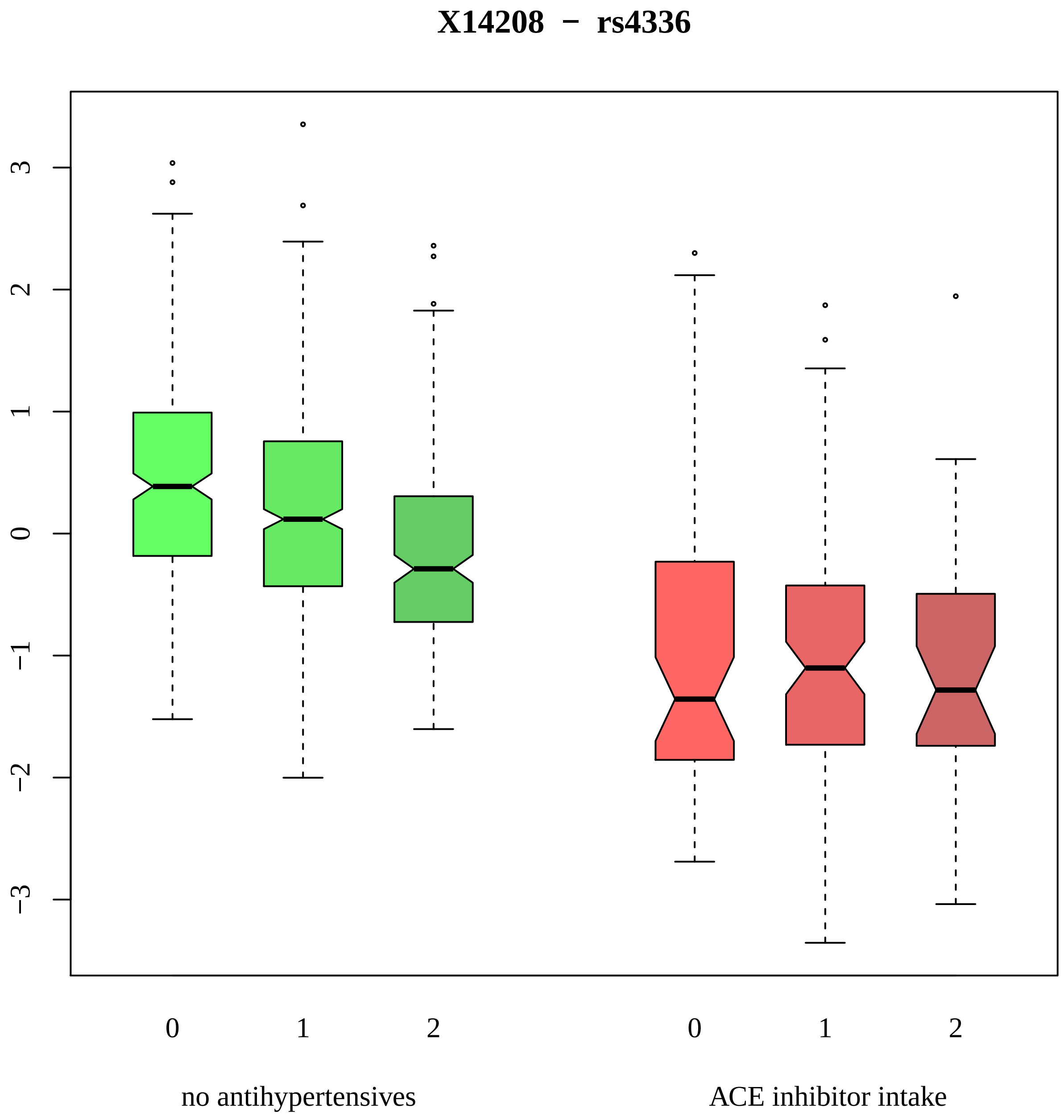
X14208 – rs4336
3
2
1
0
−1
−2
−3
0
1
2
0
1
2
no antihypertensives
ACE inhibitor intake

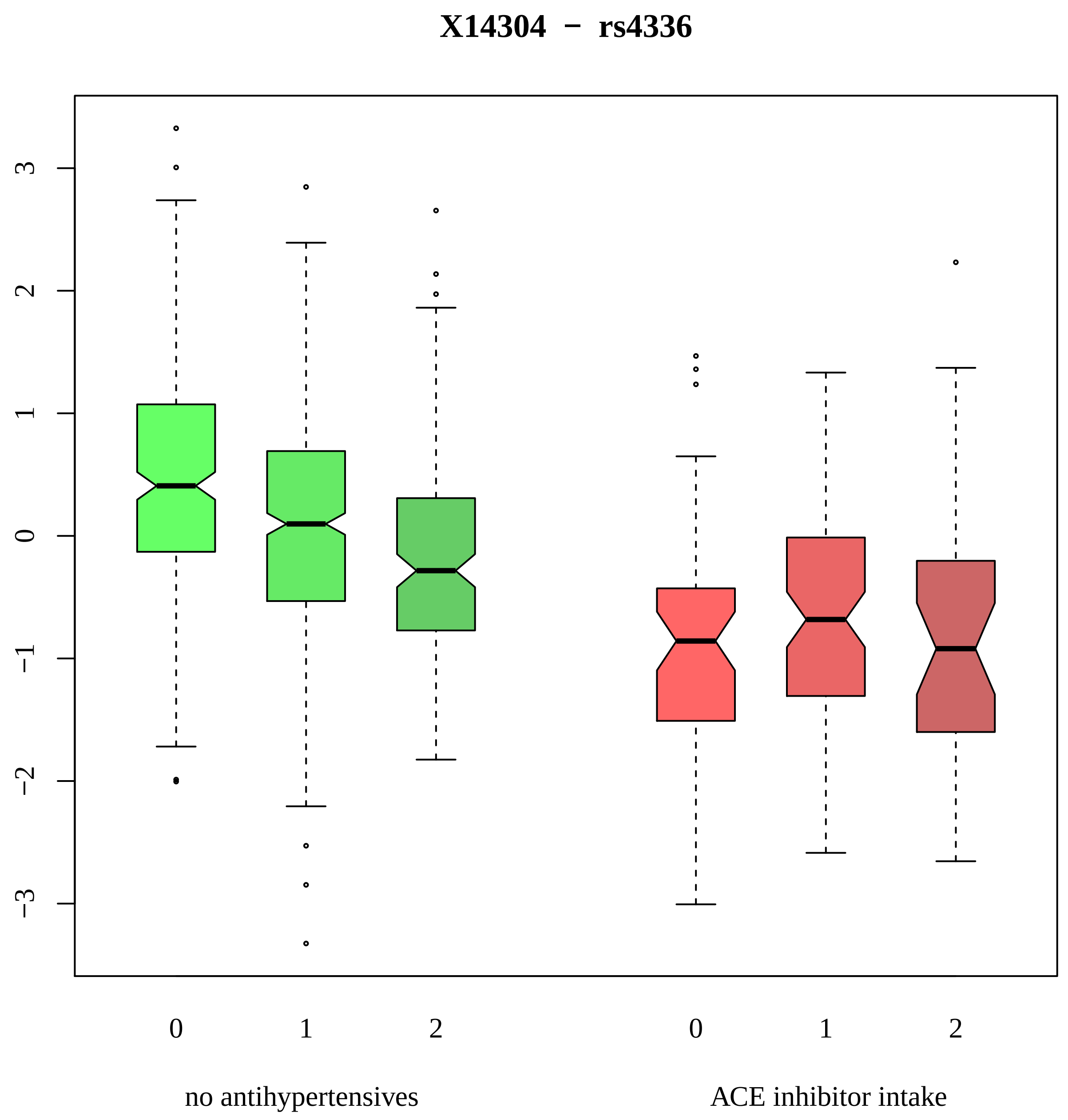

X14304 – rs4336
3
2
1
0
−1
−2
−3
0
1
2
0
1
2
no antihypertensives
ACE inhibitor intake

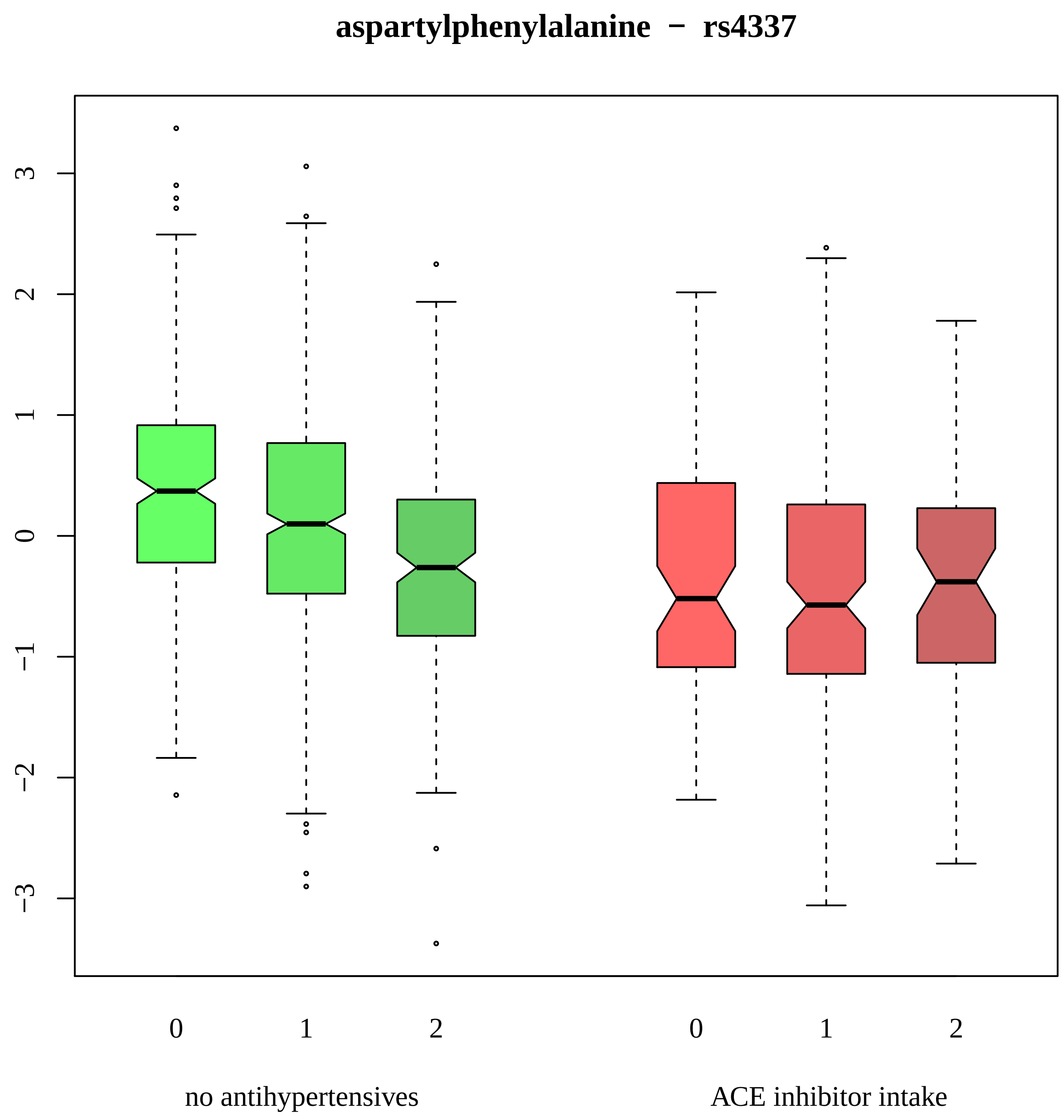

aspartylphenylalanine − rs4337
3
2
1
0
−1
−2
−3
0
1
2
0
1
2
no antihypertensives
ACE inhibitor intake

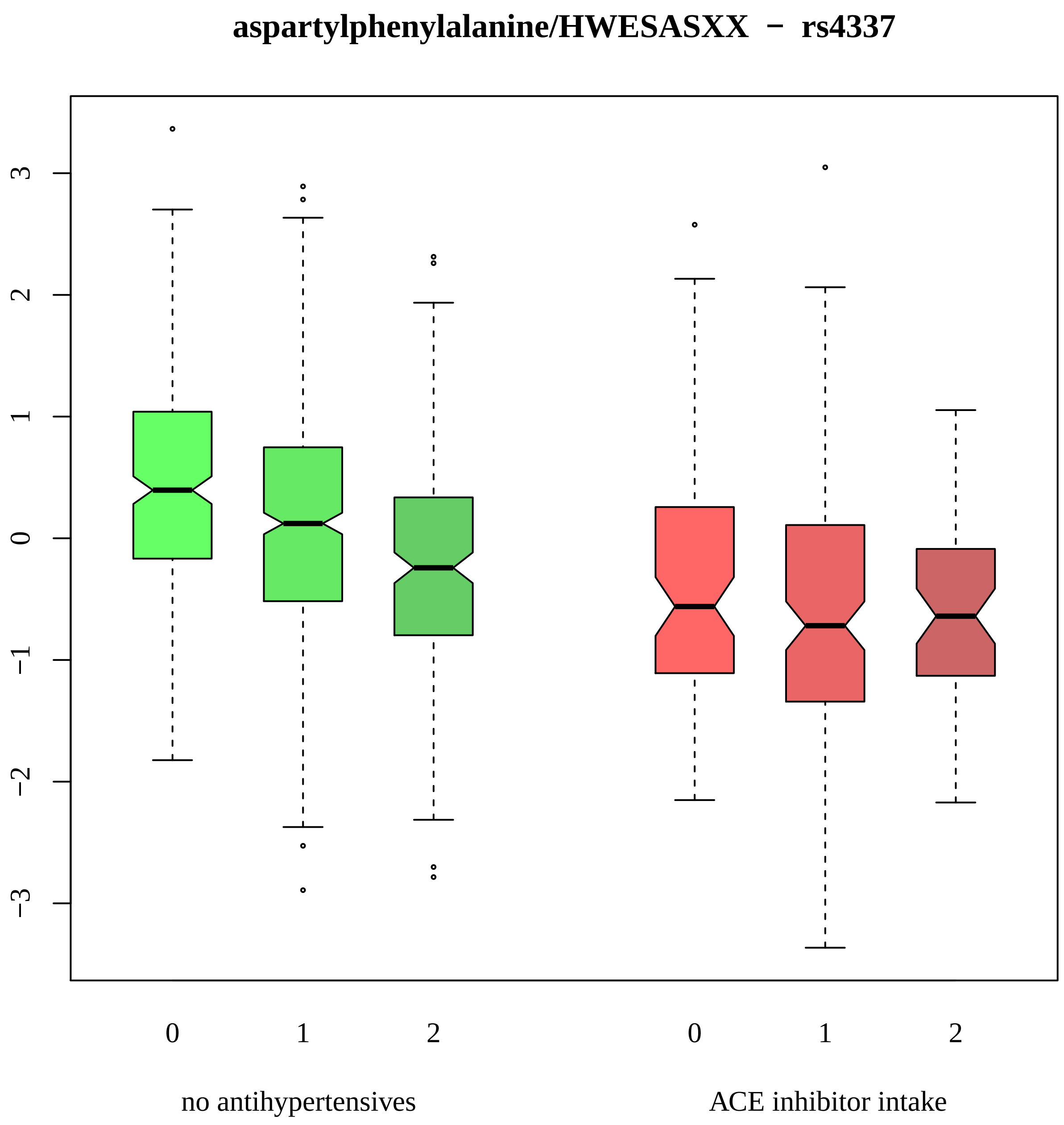

aspartylphenylalanine/HWESASXX – rs4337
3
2
1
0
−1
−2
−3
0
1
2
0
1
2
no antihypertensives
ACE inhibitor intake

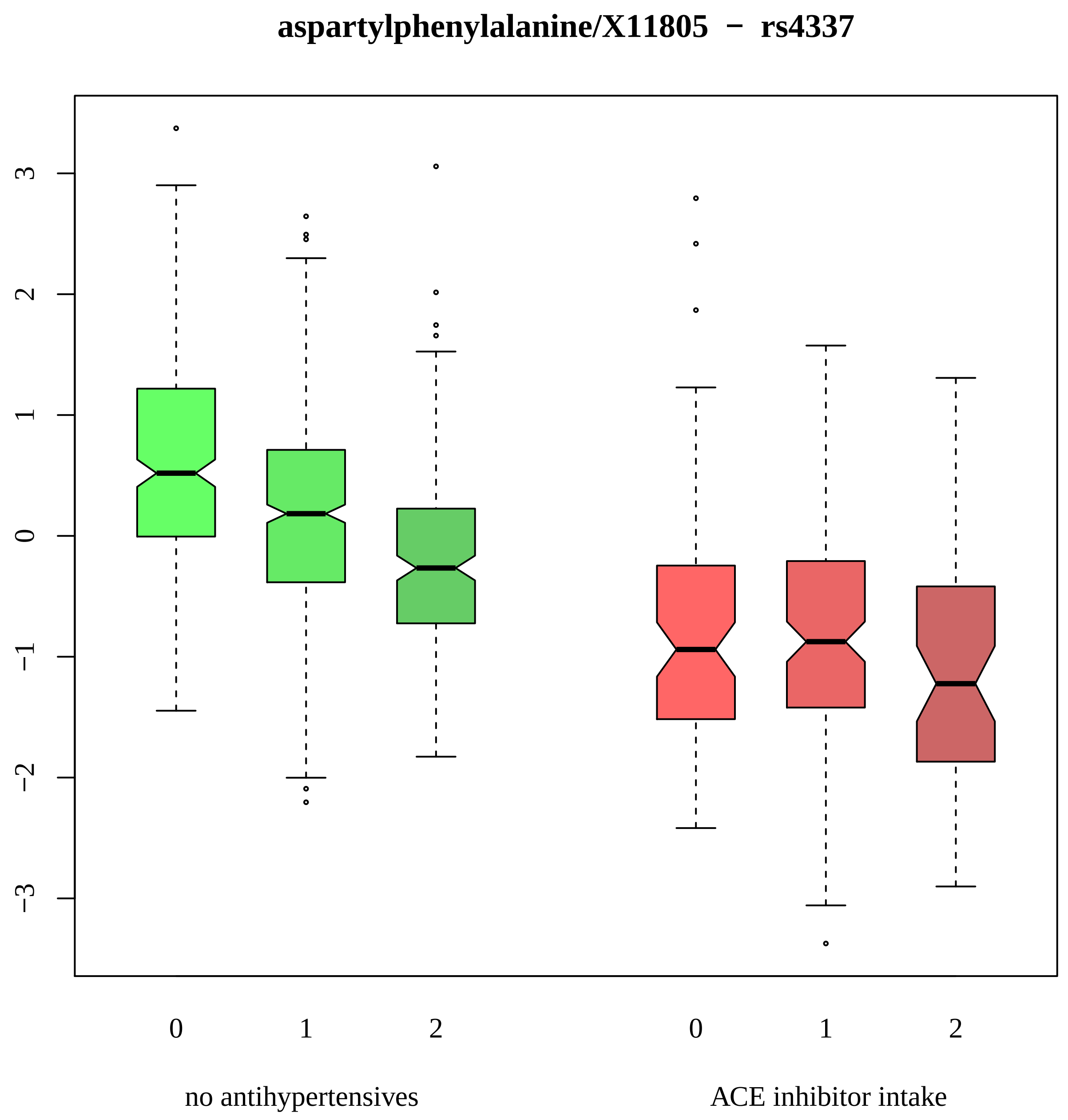
aspartylphenylalanine/X11805 – rs4337
3
2
1
0
−1
−2
−3
0
1
2
0
1
2
no antihypertensives
ACE inhibitor intake

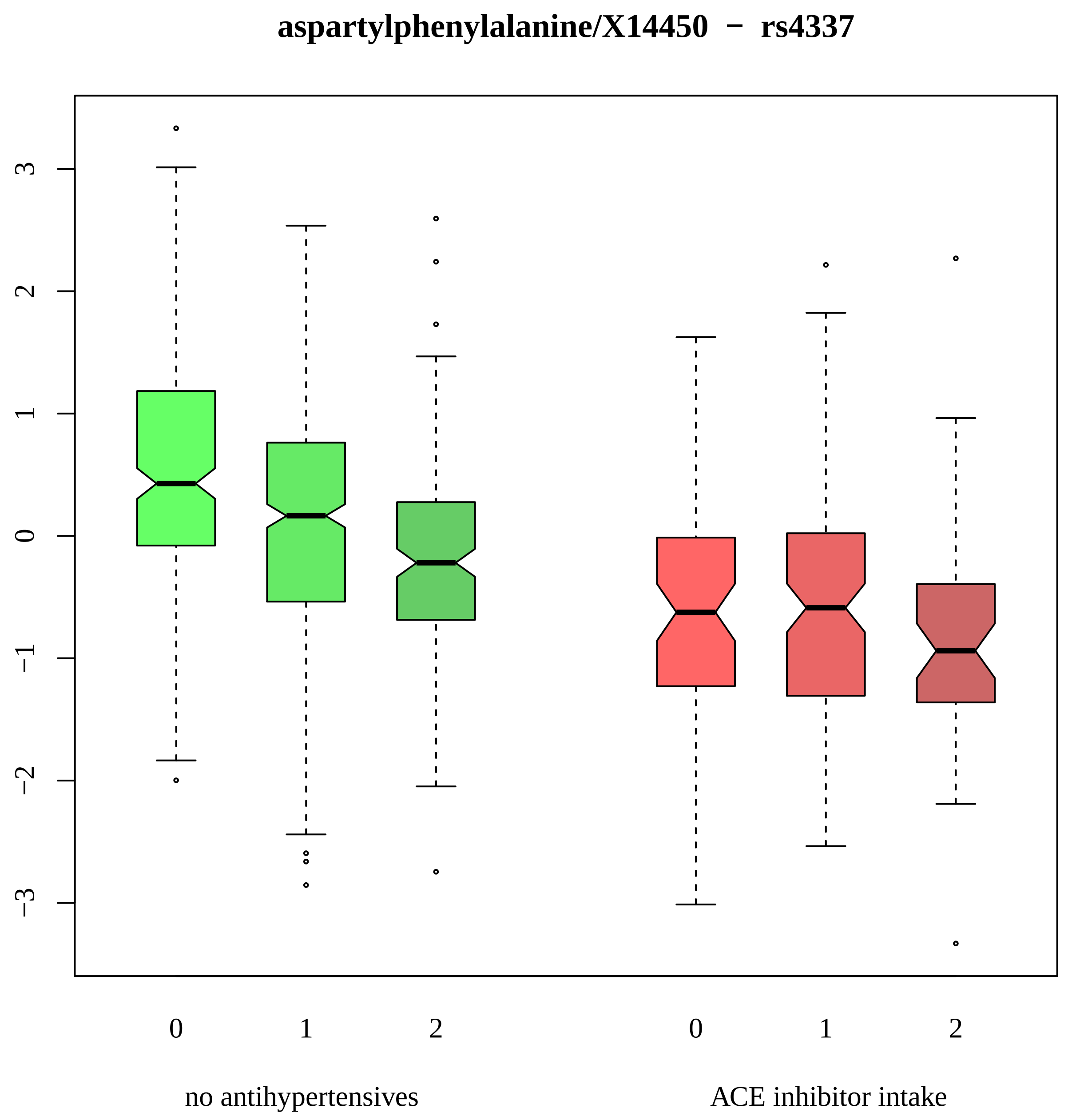
aspartylphenylalanine/X14450 – rs4337
3
2
1
0
−1
−2
−3
0
1
2
0
1
2
no antihypertensives
ACE inhibitor intake

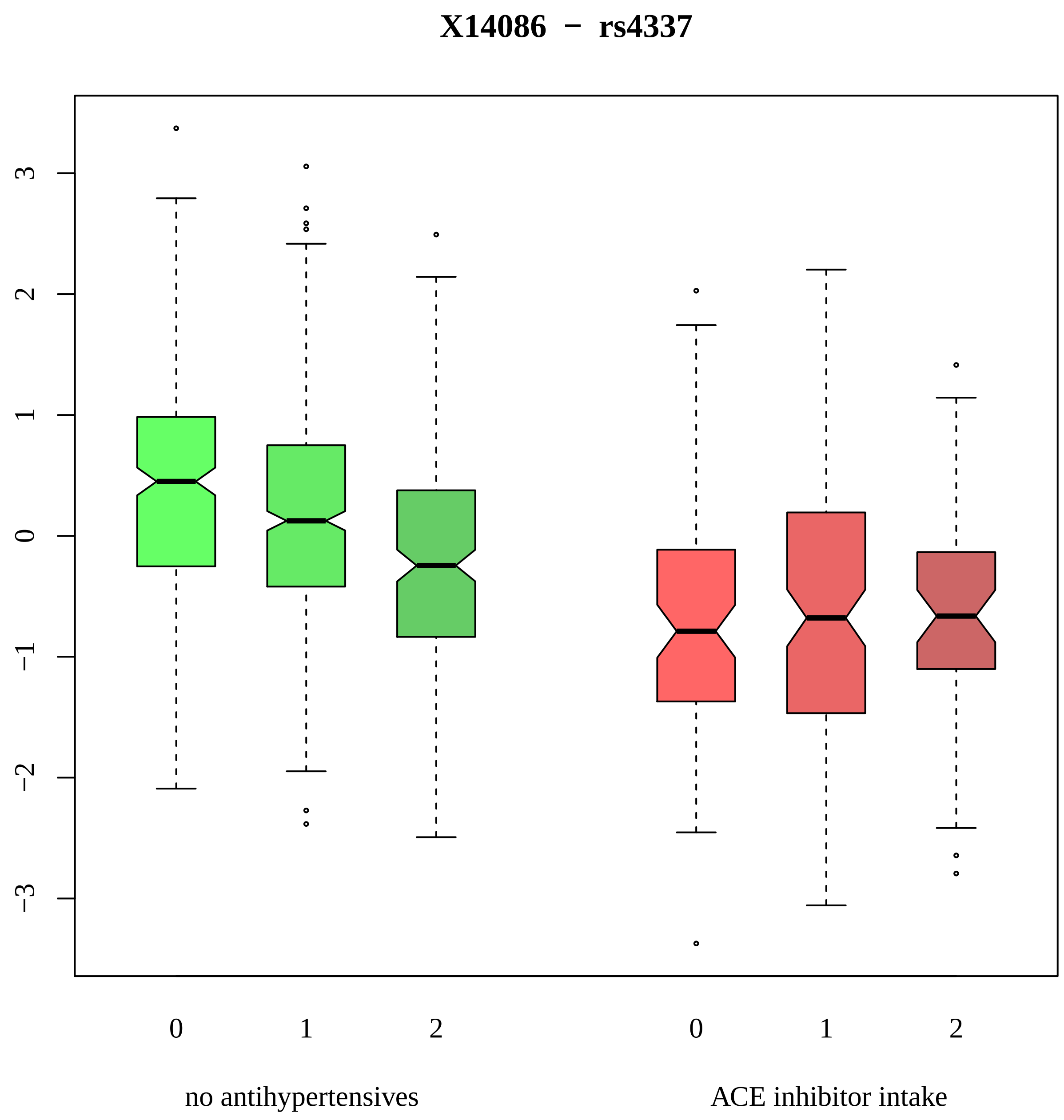

X14086 – rs4337
3
2
1
0
−1
−2
−3
0
1
2
0
1
2
no antihypertensives
ACE inhibitor intake

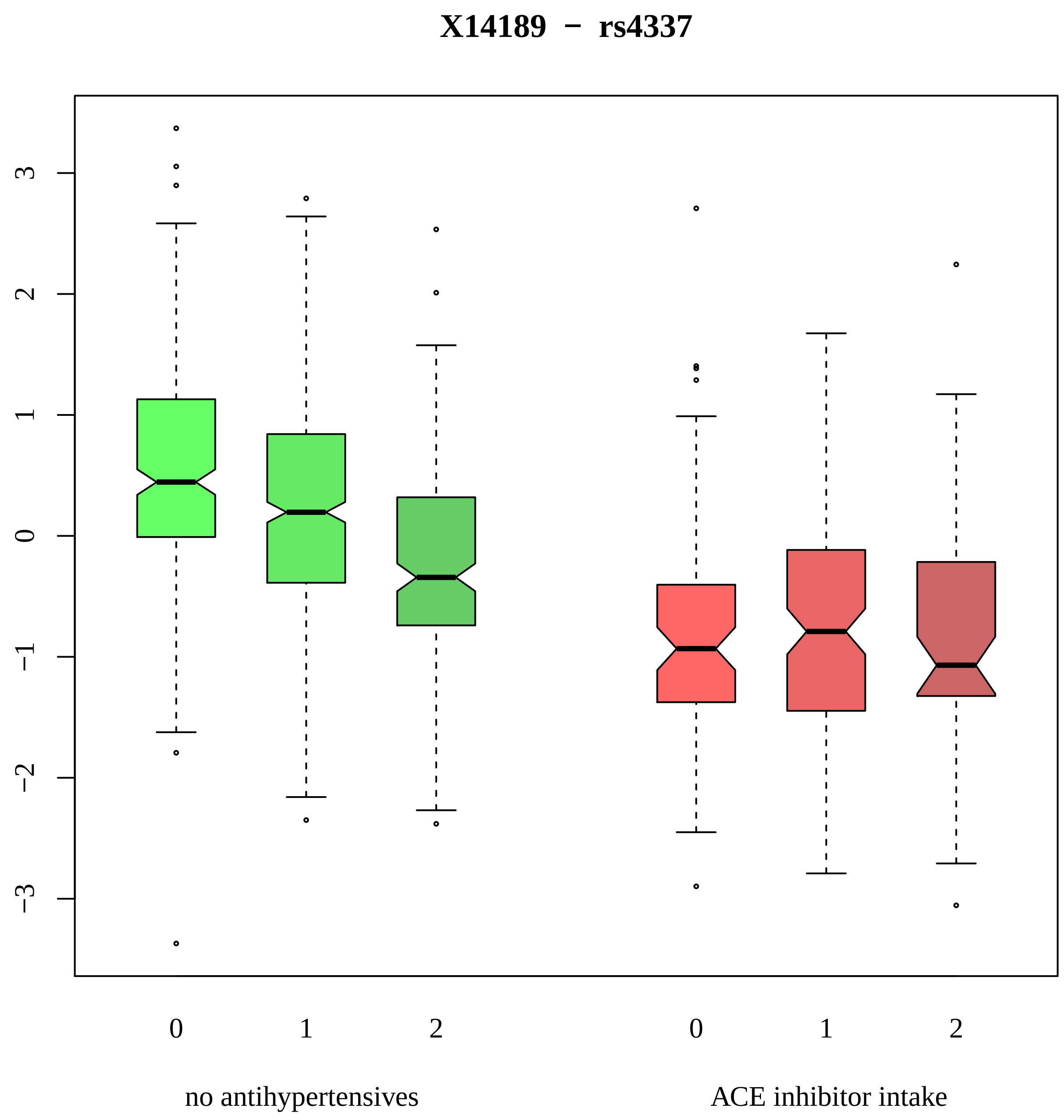

X14189 – rs4337
3
2
1
0
−1
−2
−3
0
1
2
0
1
2
no antihypertensives
ACE inhibitor intake

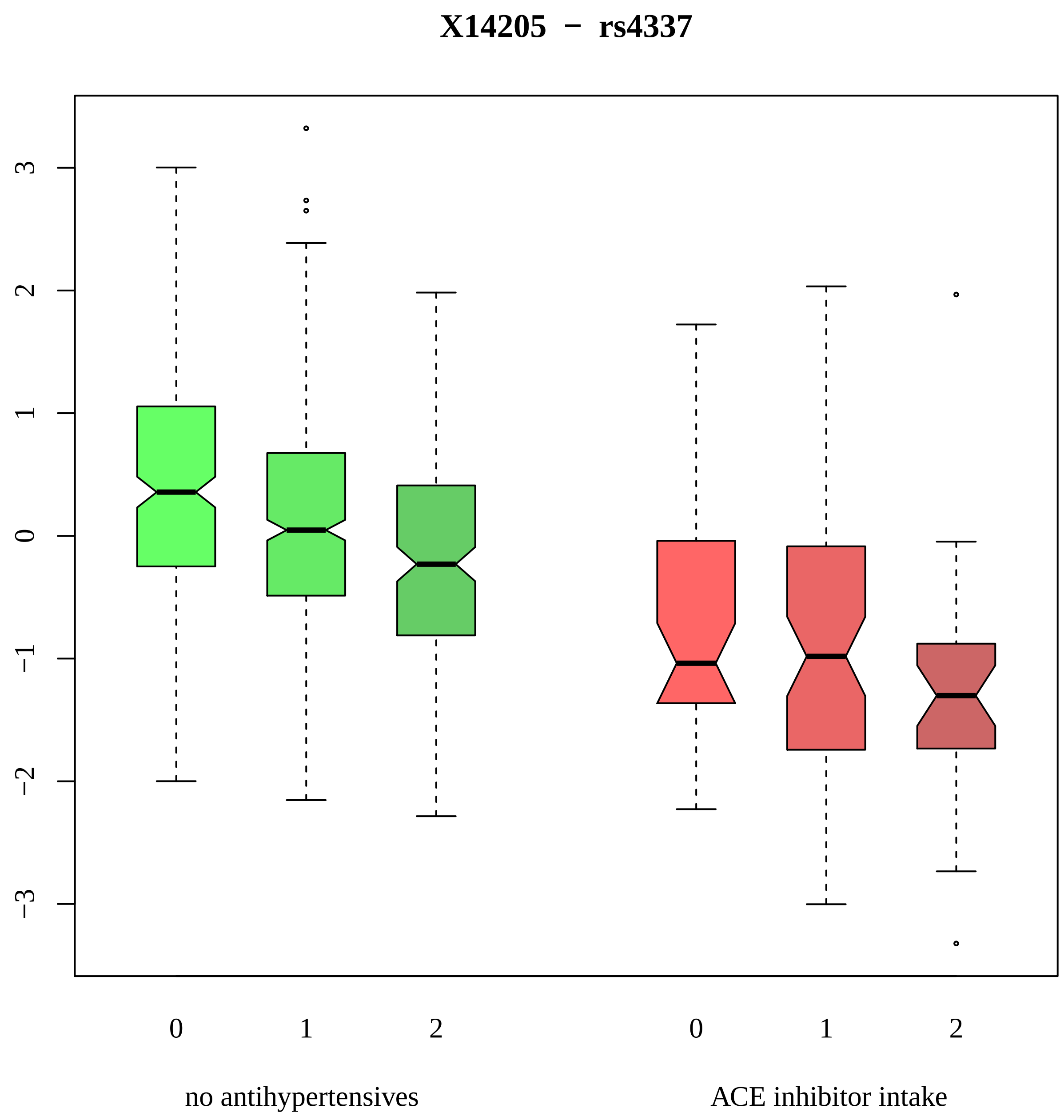
X14205 – rs4337
3
2
1
0
−1
−2
−3
0
1
2
0
1
2
no antihypertensives
ACE inhibitor intake

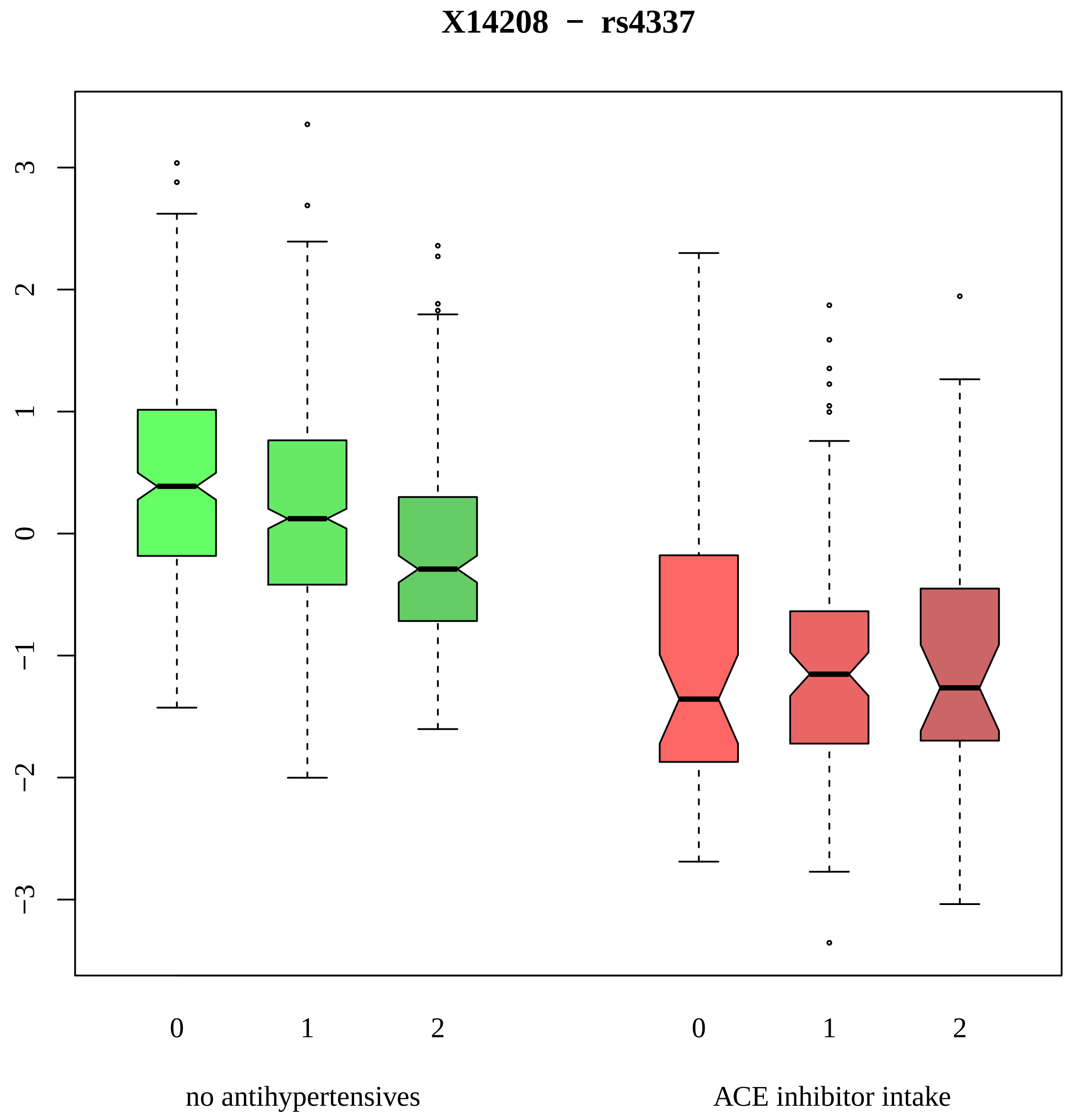

X14208 – rs4337
3
2
1
0
−1
−2
−3
0
1
2
0
1
2
no antihypertensives
ACE inhibitor intake

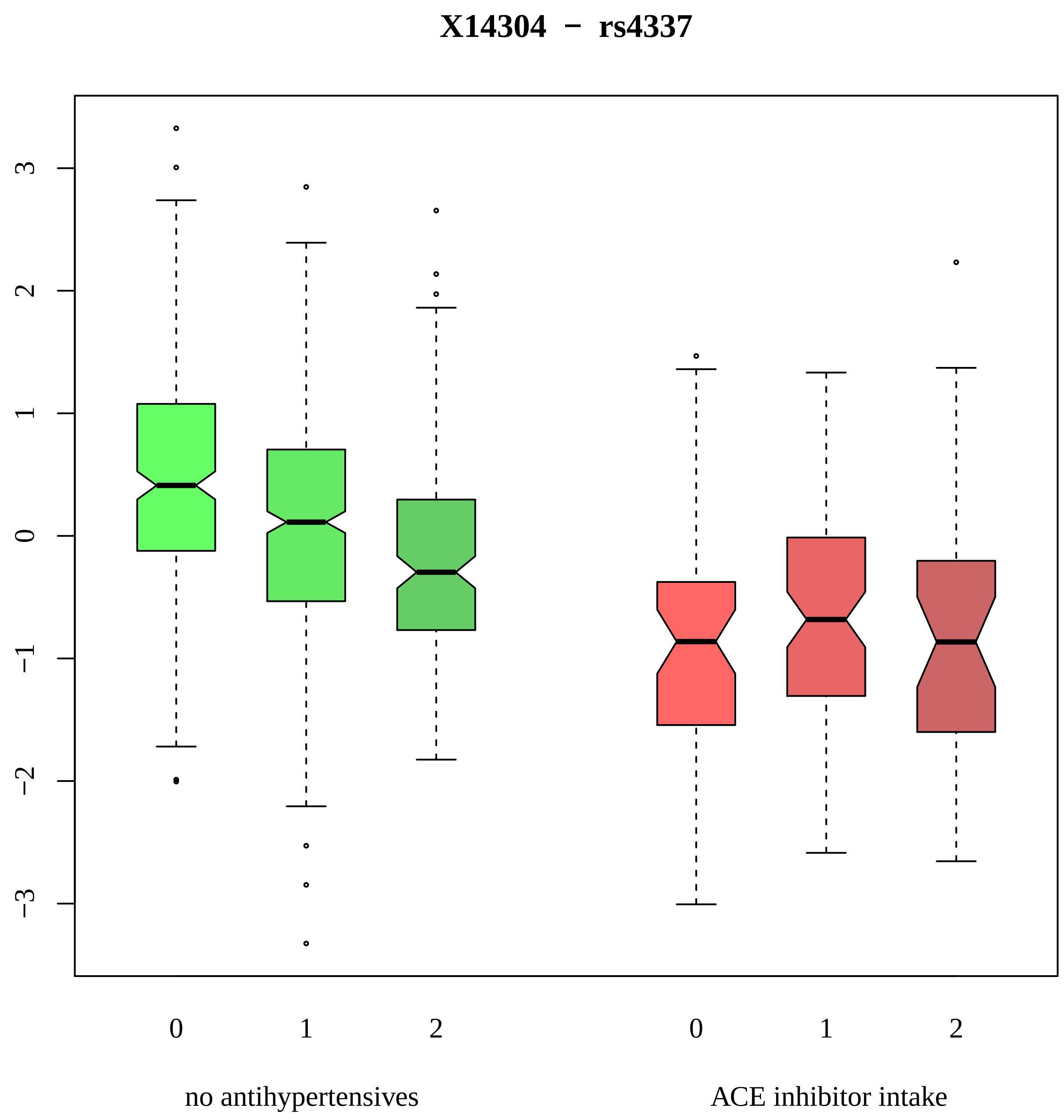

X14304 – rs4337
3
2
1
0
−1
−2
−3
0
1
2
0
1
2
no antihypertensives
ACE inhibitor intake

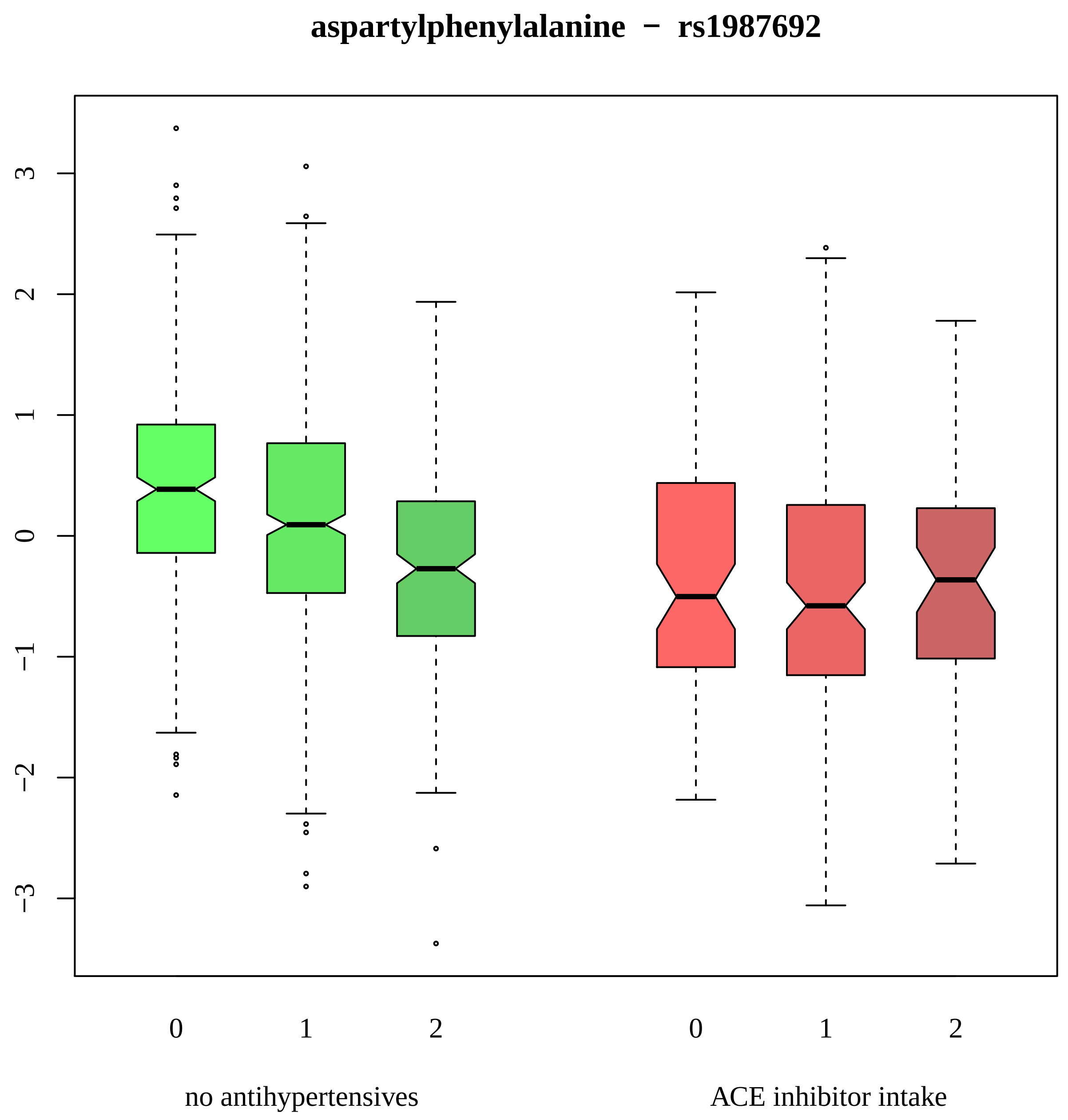
aspartylphenylalanine − rs1987692
3
2
1
0
−1
−2
−3
0
1
2
0
1
2
no antihypertensives
ACE inhibitor intake

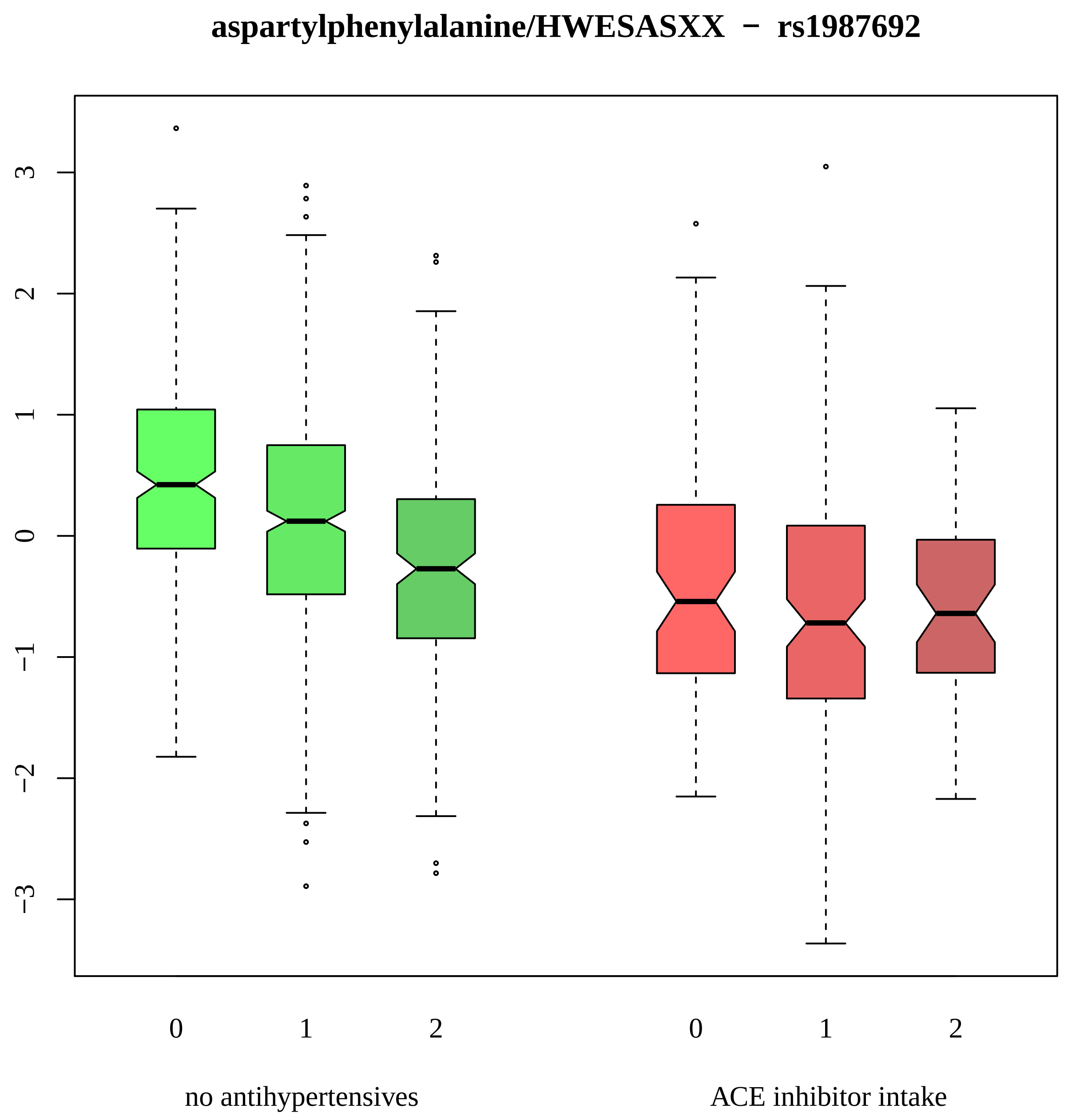

aspartylphenylalanine/HWESASXX – rs1987692
3
2
1
0
−1
−2
−3
0
1
2
0
1
2
no antihypertensives
ACE inhibitor intake

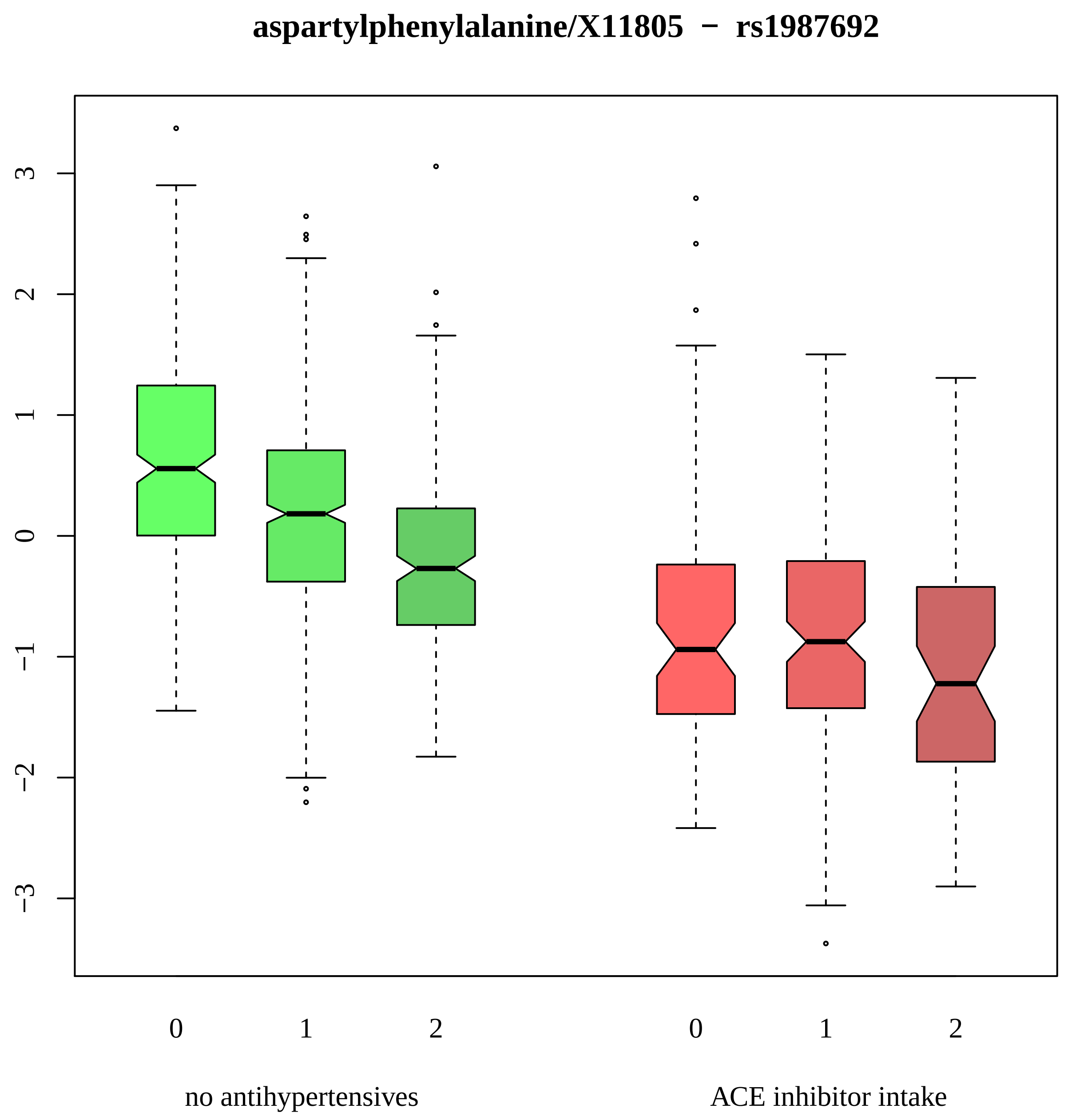

aspartylphenylalanine/X11805 − rs1987692
3
2
1
0
−1
−2
−3
0
1
2
0
1
2
no antihypertensives
ACE inhibitor intake

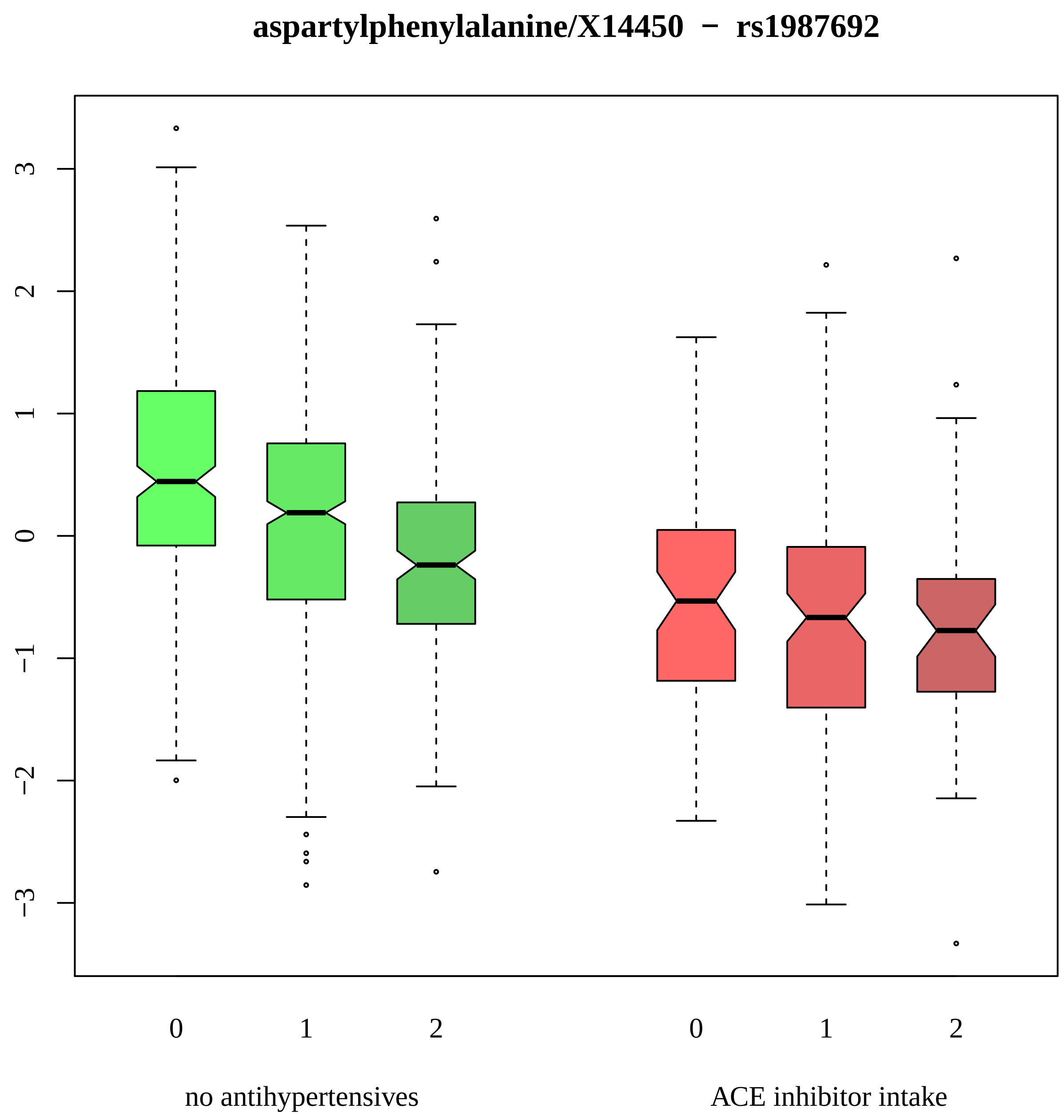
aspartylphenylalanine/X14450 − rs1987692
3
2
1
0
−1
−2
−3
0
1
2
0
1
2
no antihypertensives
ACE inhibitor intake

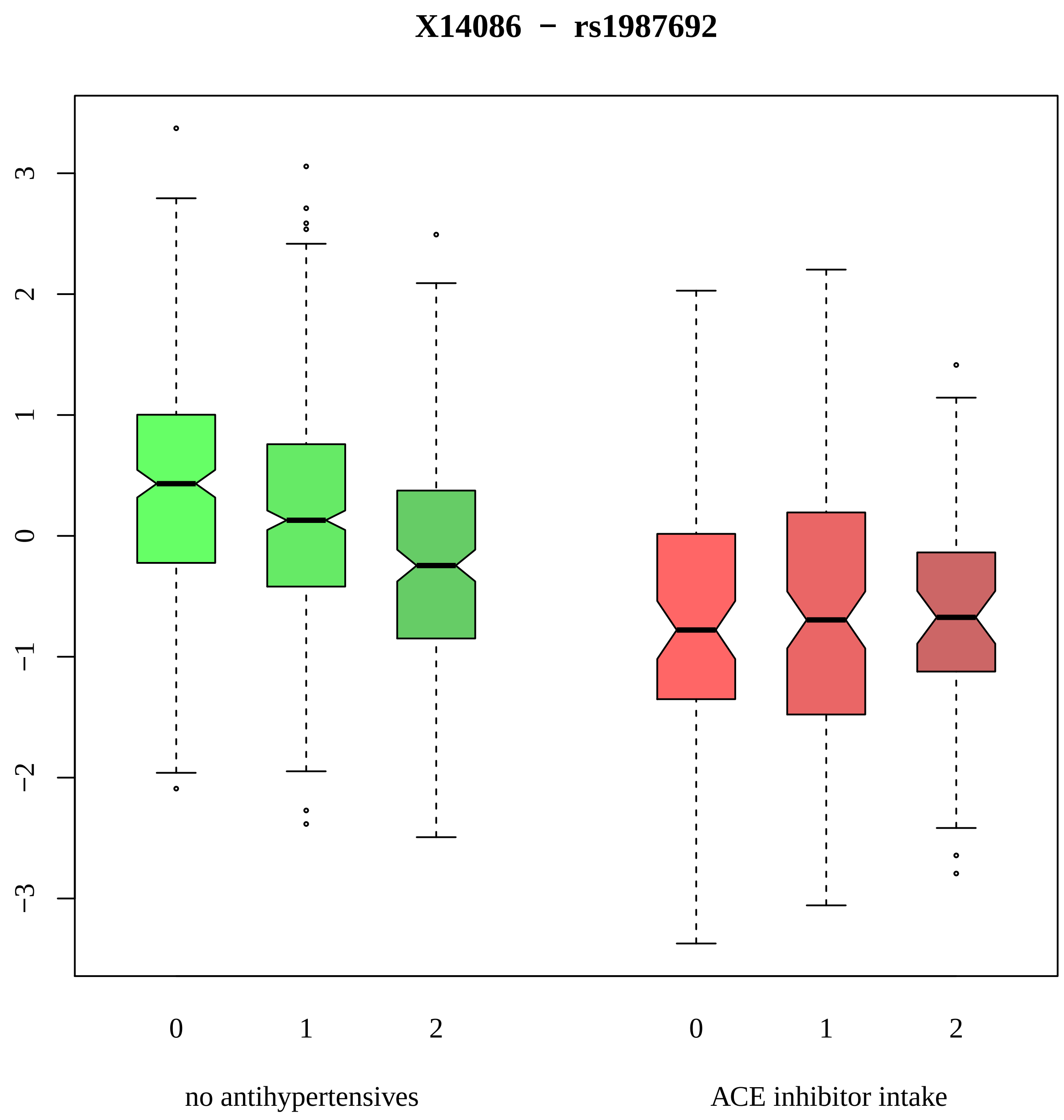

X14086 − rs1987692
3
2
1
0
−1
−2
−3
0
1
2
0
1
2
no antihypertensives
ACE inhibitor intake

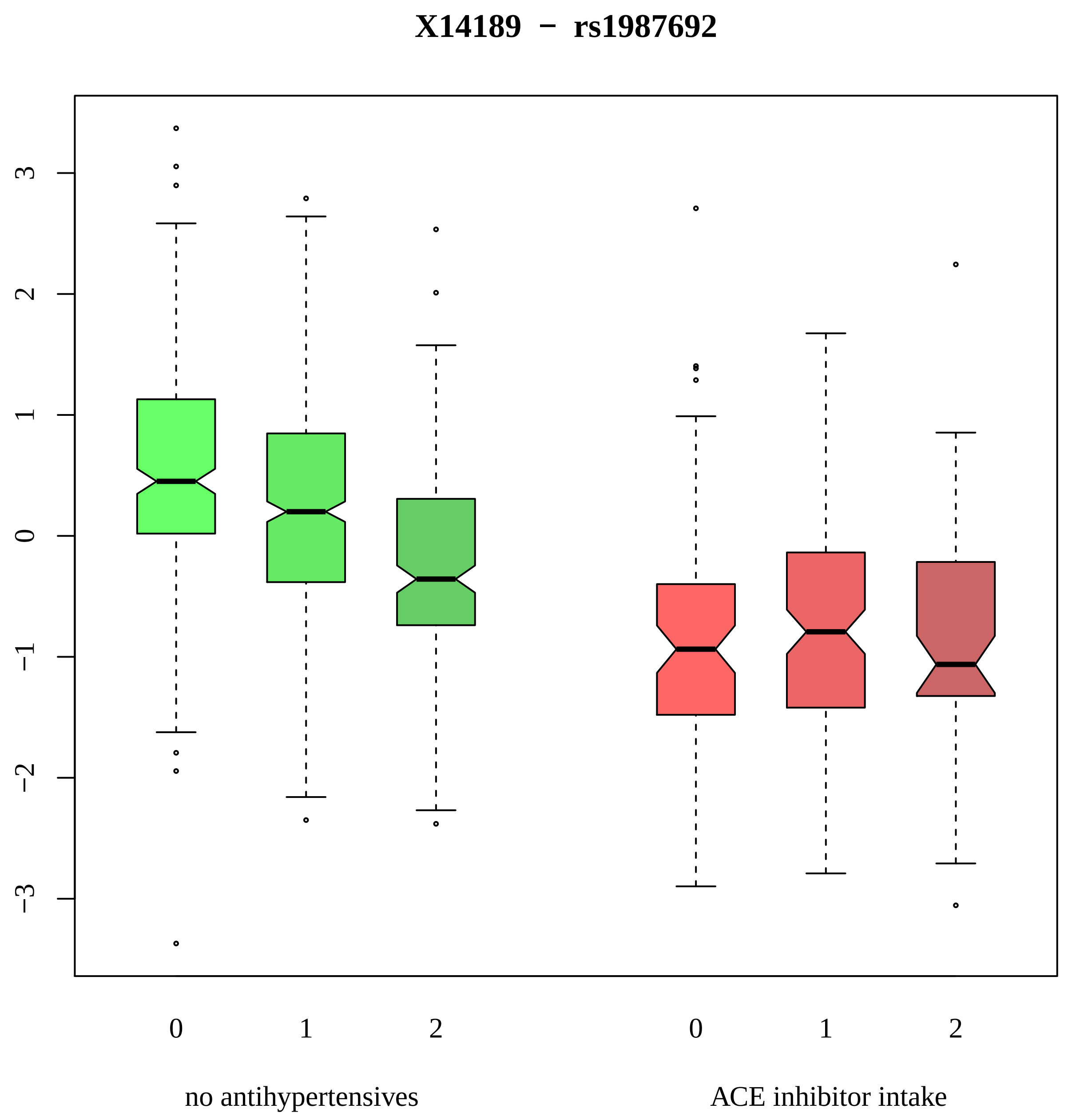

X14189 – rs1987692
3
2
1
0
−1
−2
−3
0
1
2
0
1
2
no antihypertensives
ACE inhibitor intake

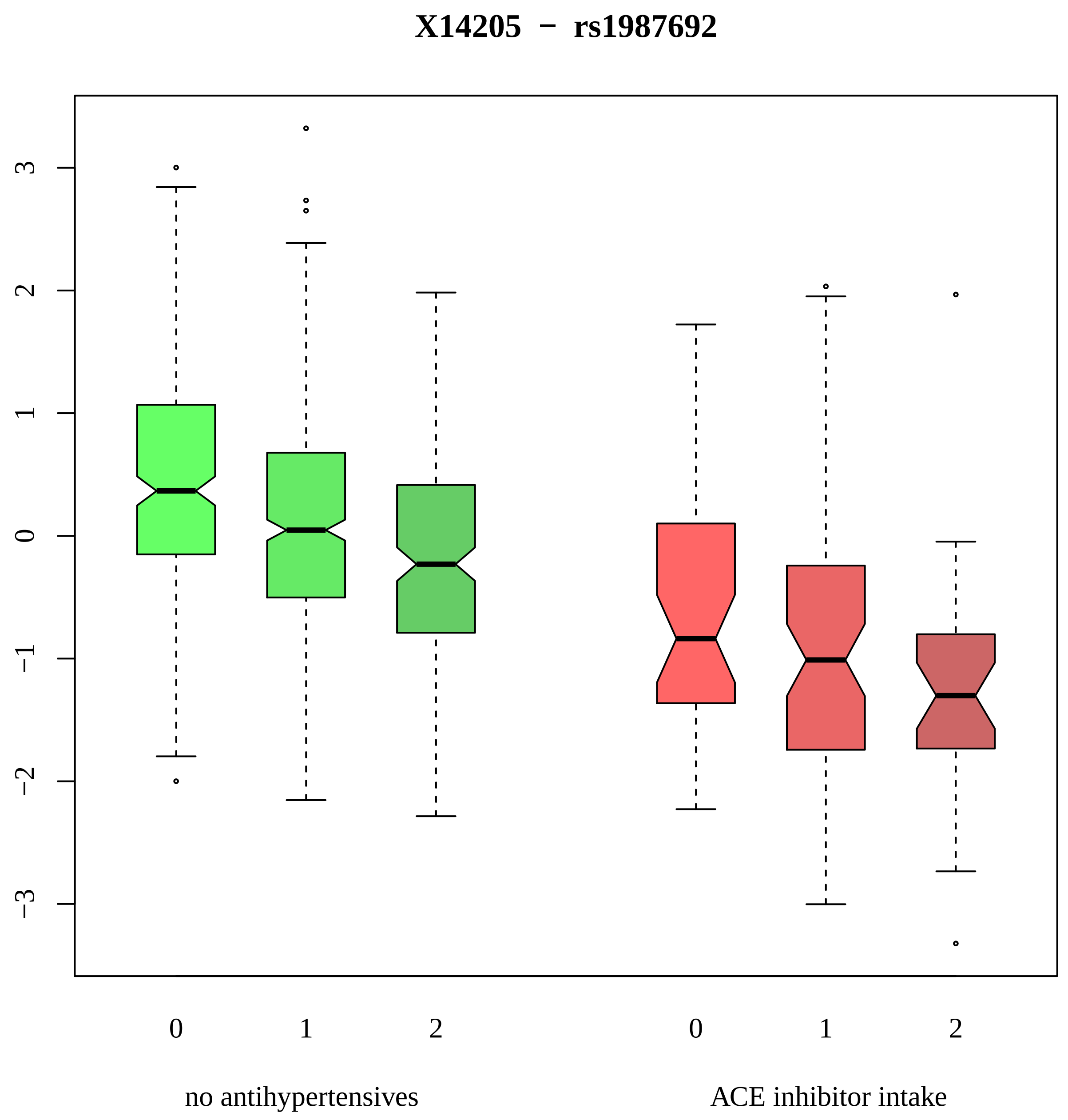

X14205 – rs1987692
3
2
1
0
−1
−2
−3
0
1
2
0
1
2
no antihypertensives
ACE inhibitor intake

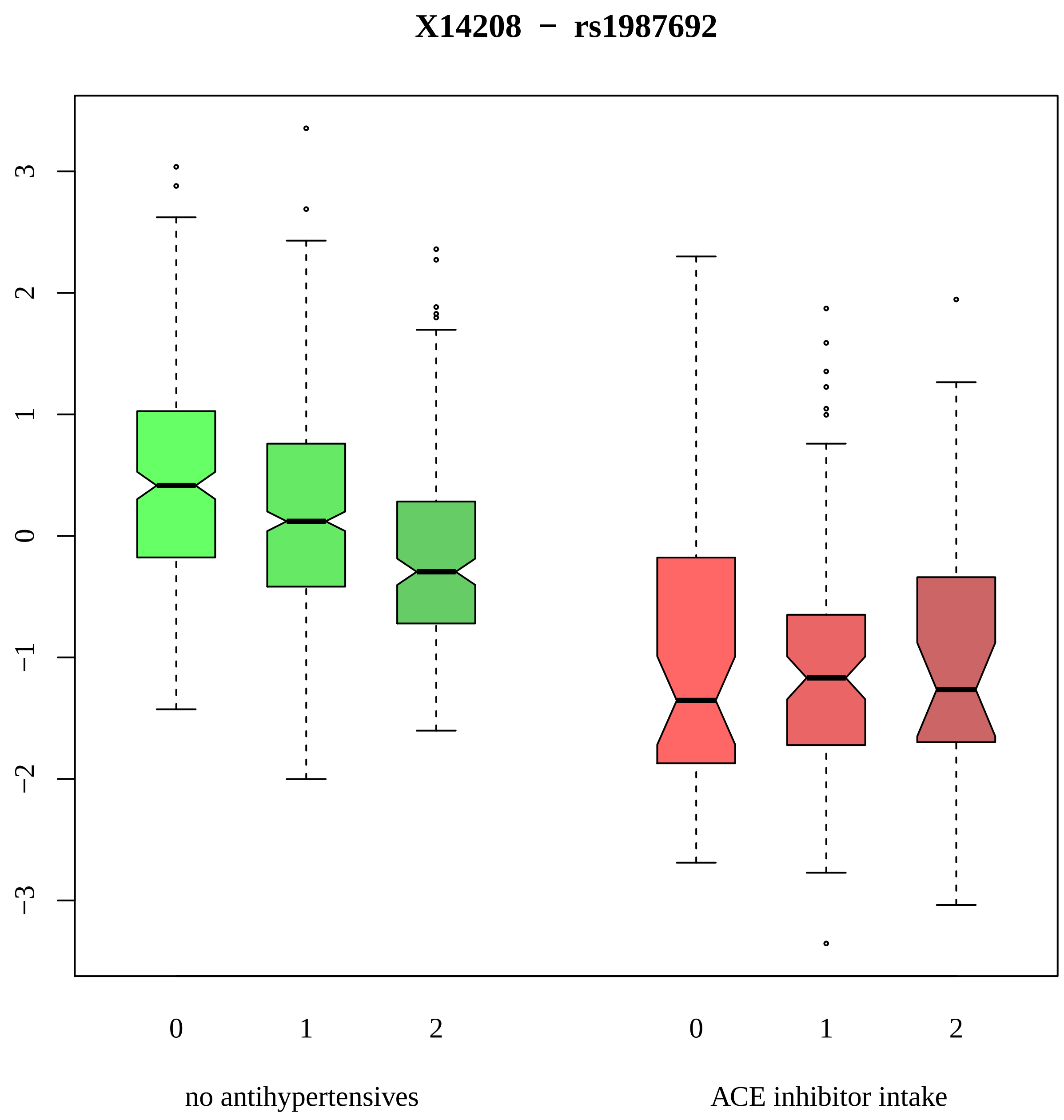

X14208 – rs1987692
3
2
1
0
−1
−2
−3
0
1
2
0
1
2
no antihypertensives
ACE inhibitor intake

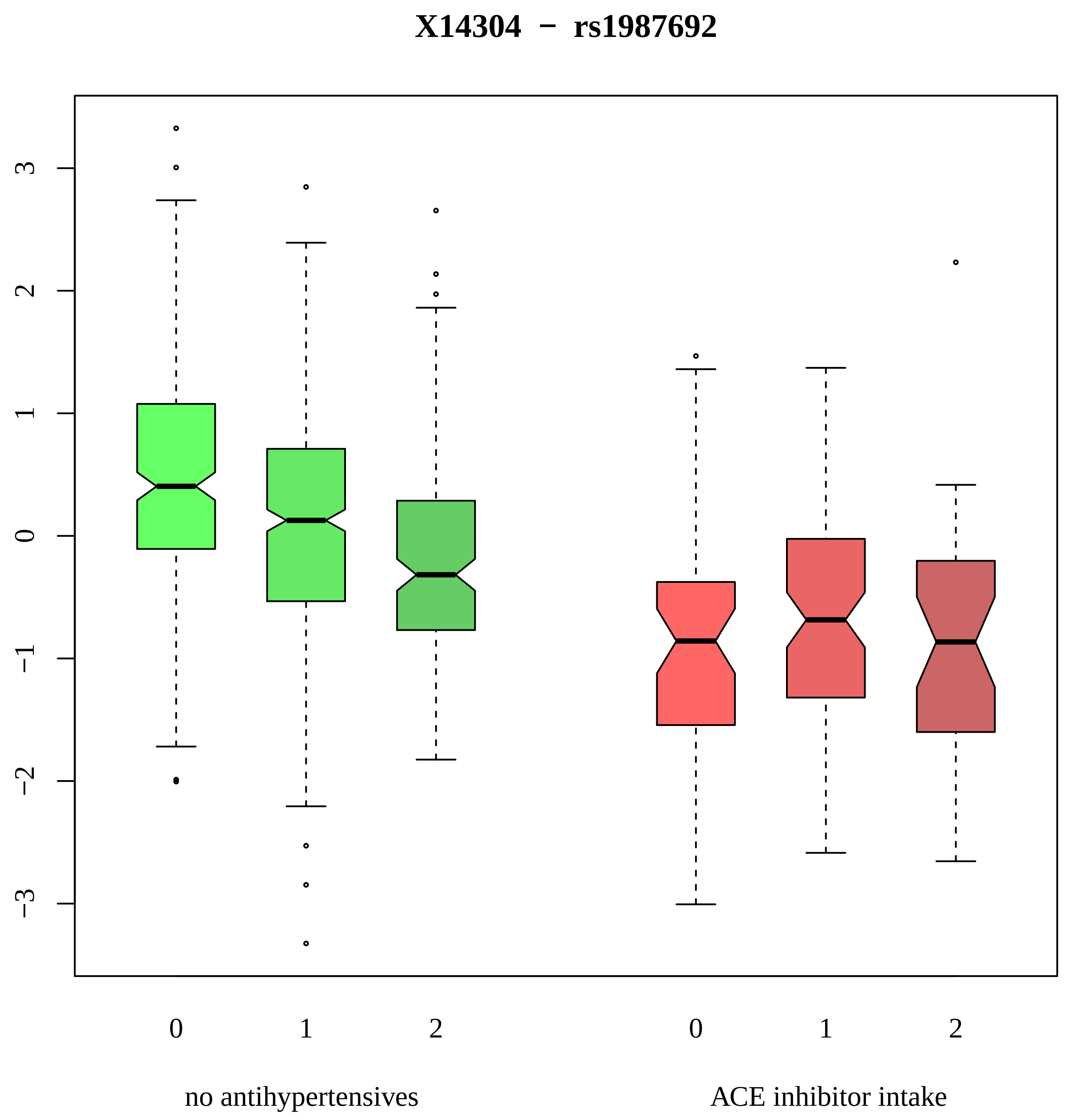

X14304 – rs1987692
3
2
1
0
−1
−2
−3
0
1
2
0
1
2
no antihypertensives
ACE inhibitor intake

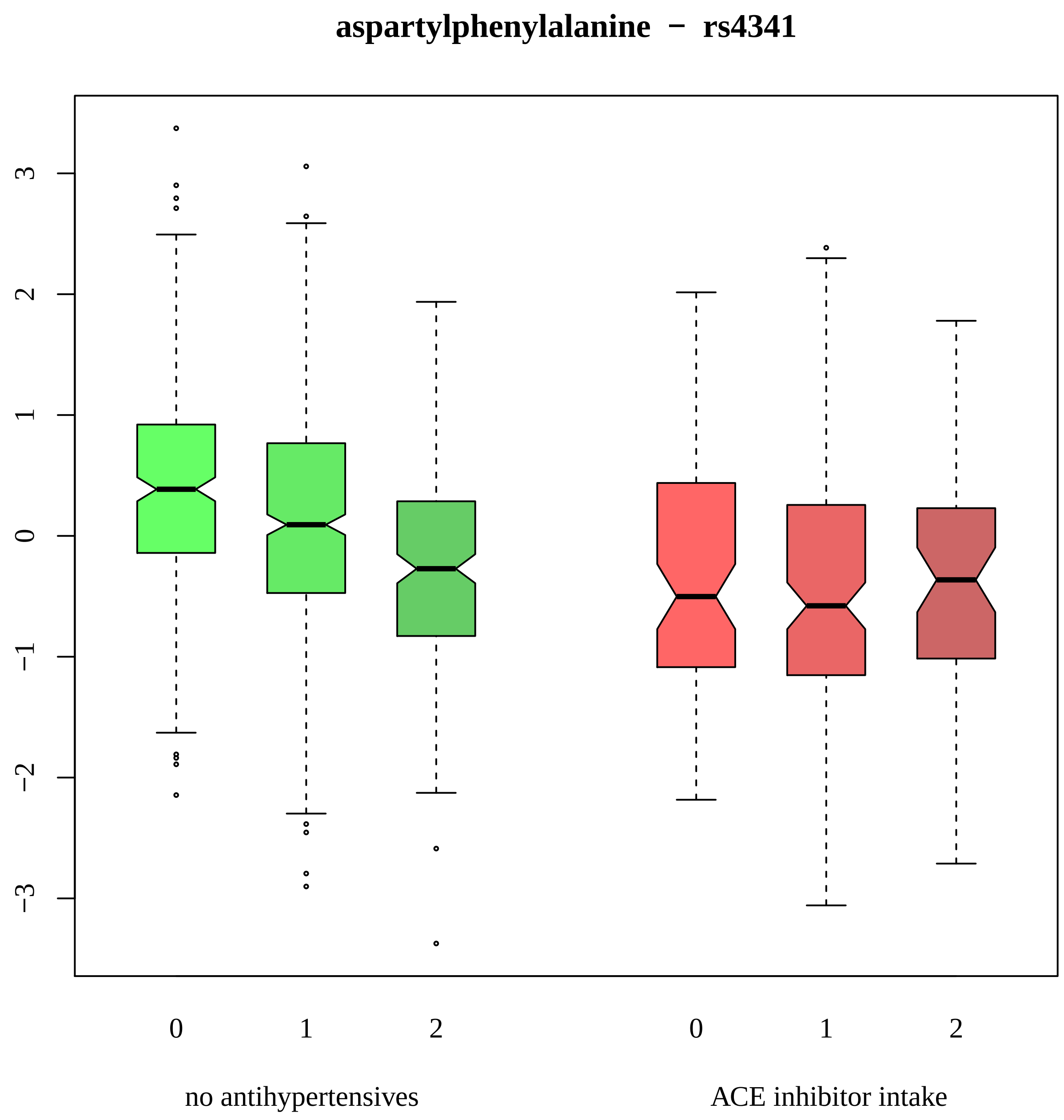
aspartylphenylalanine − rs4341
3
2
1
0
−1
−2
−3
0
1
2
0
1
2
no antihypertensives
ACE inhibitor intake

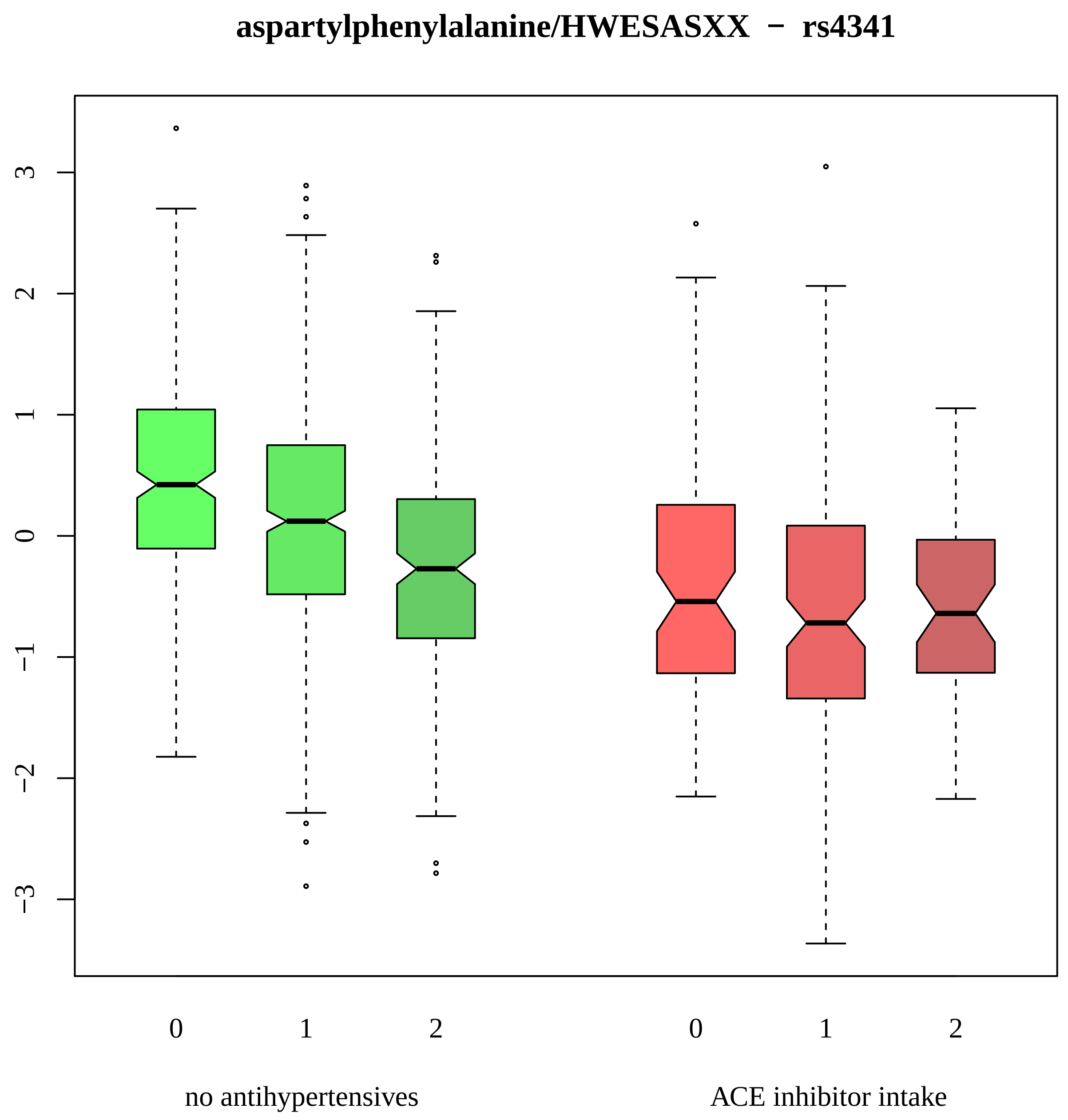

aspartylphenylalanine/HWESASXX – rs4341
3
2
1
0
−1
−2
−3
0
1
2
0
1
2
no antihypertensives
ACE inhibitor intake

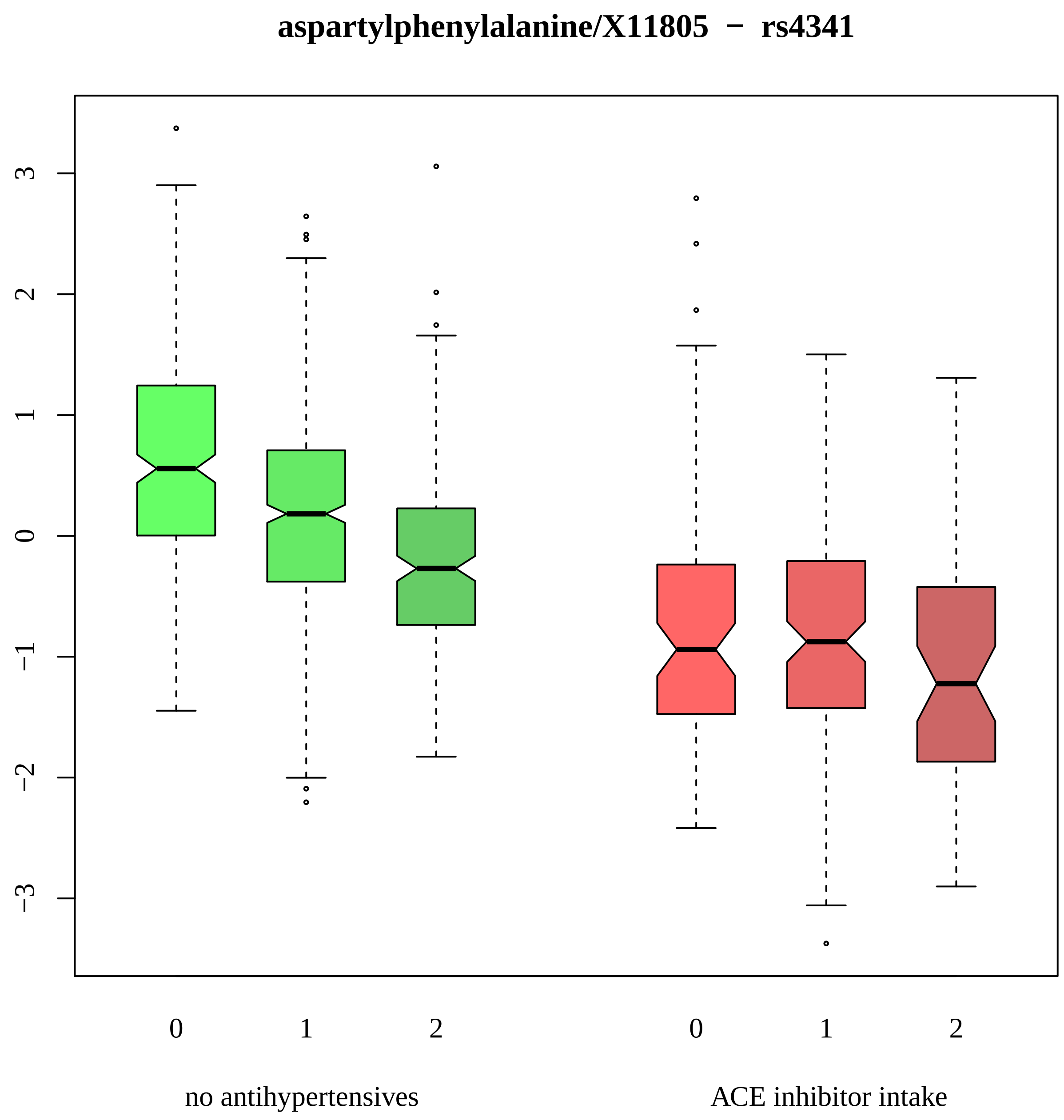

aspartylphenylalanine/X11805 – rs4341
3
2
1
0
−1
−2
−3
0
1
2
0
1
2
no antihypertensives
ACE inhibitor intake

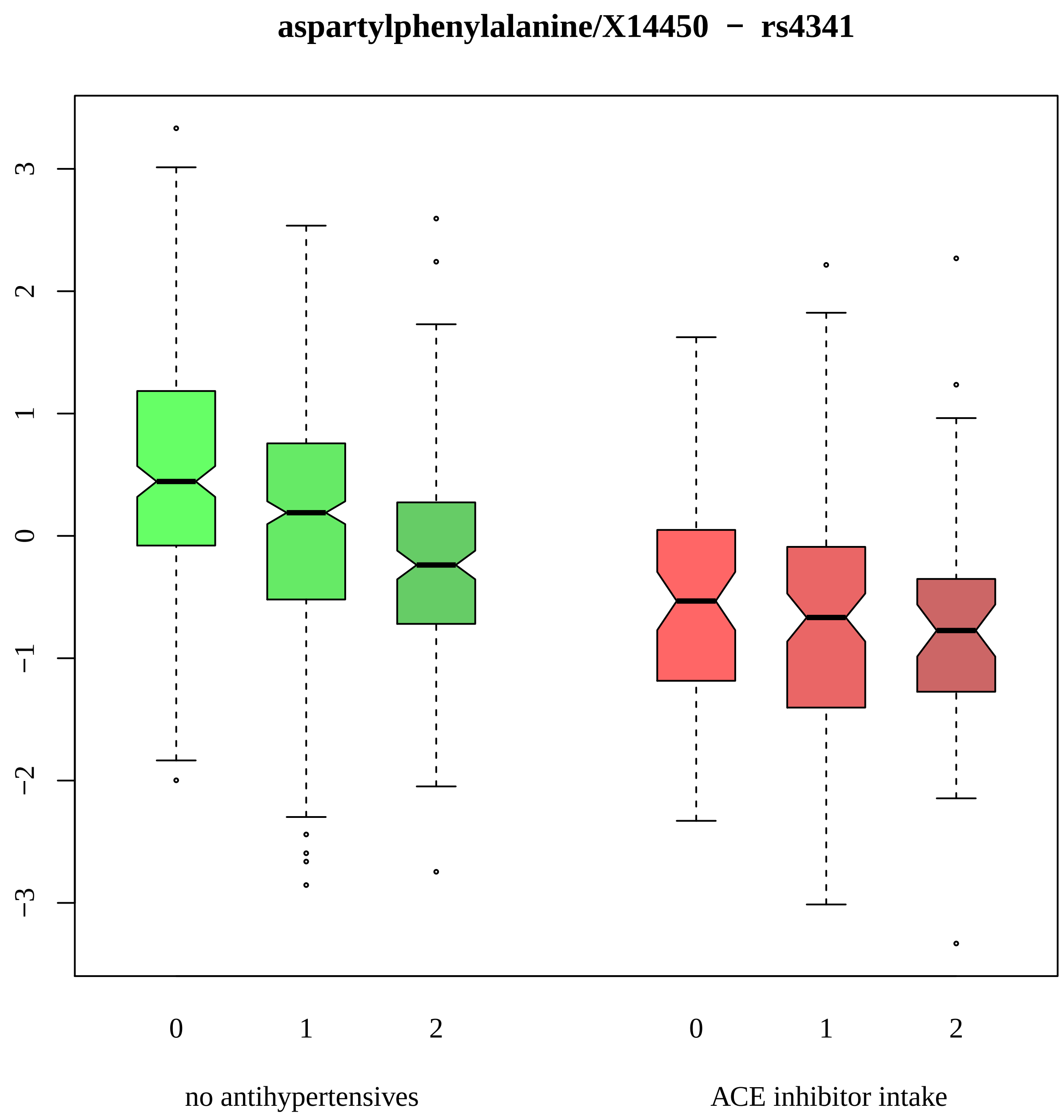
aspartylphenylalanine/X14450 – rs4341
3
2
1
0
−1
−2
−3
0
1
2
0
1
2
no antihypertensives
ACE inhibitor intake

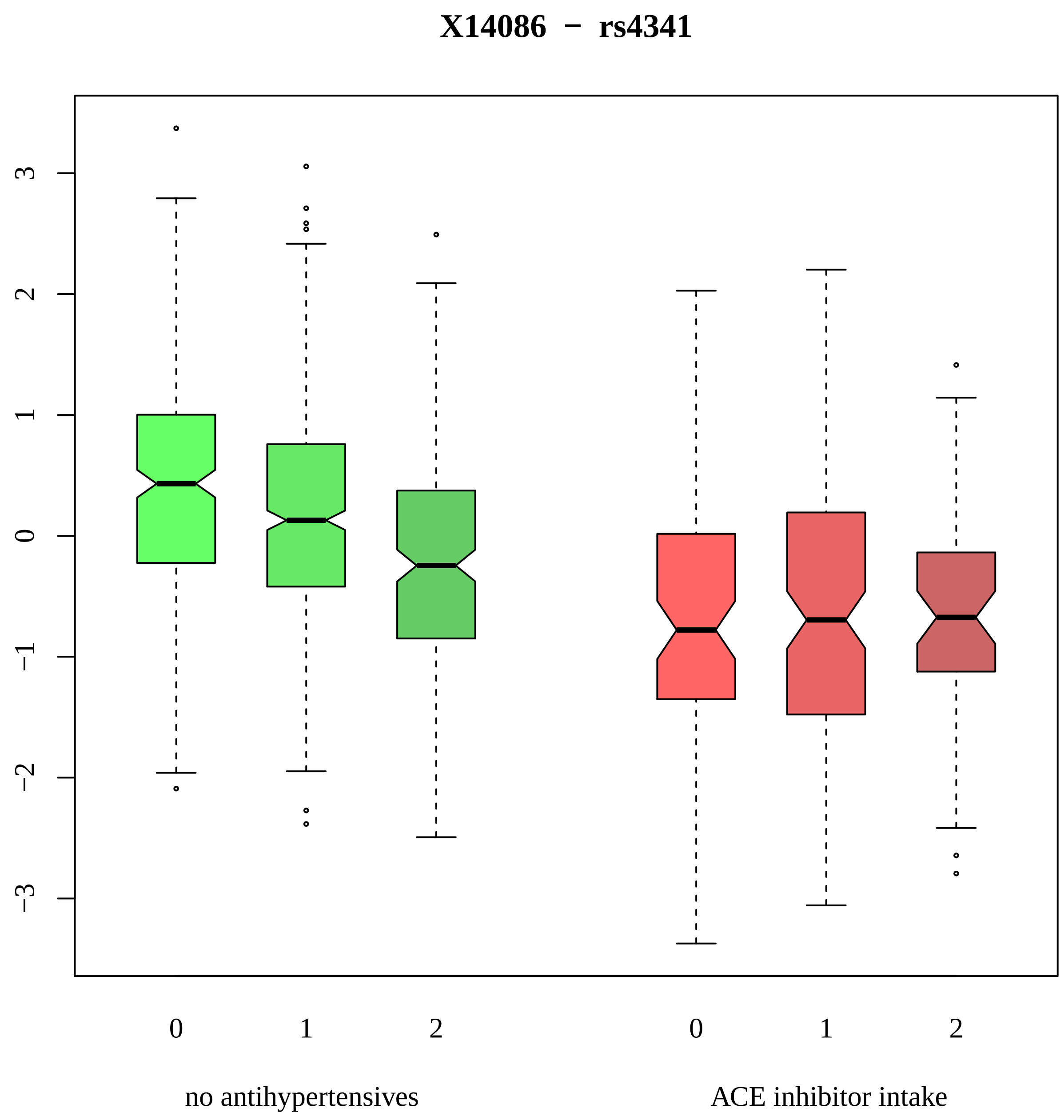
X14086 – rs4341
3
2
1
0
−1
−2
−3
0
1
2
0
1
2
no antihypertensives
ACE inhibitor intake

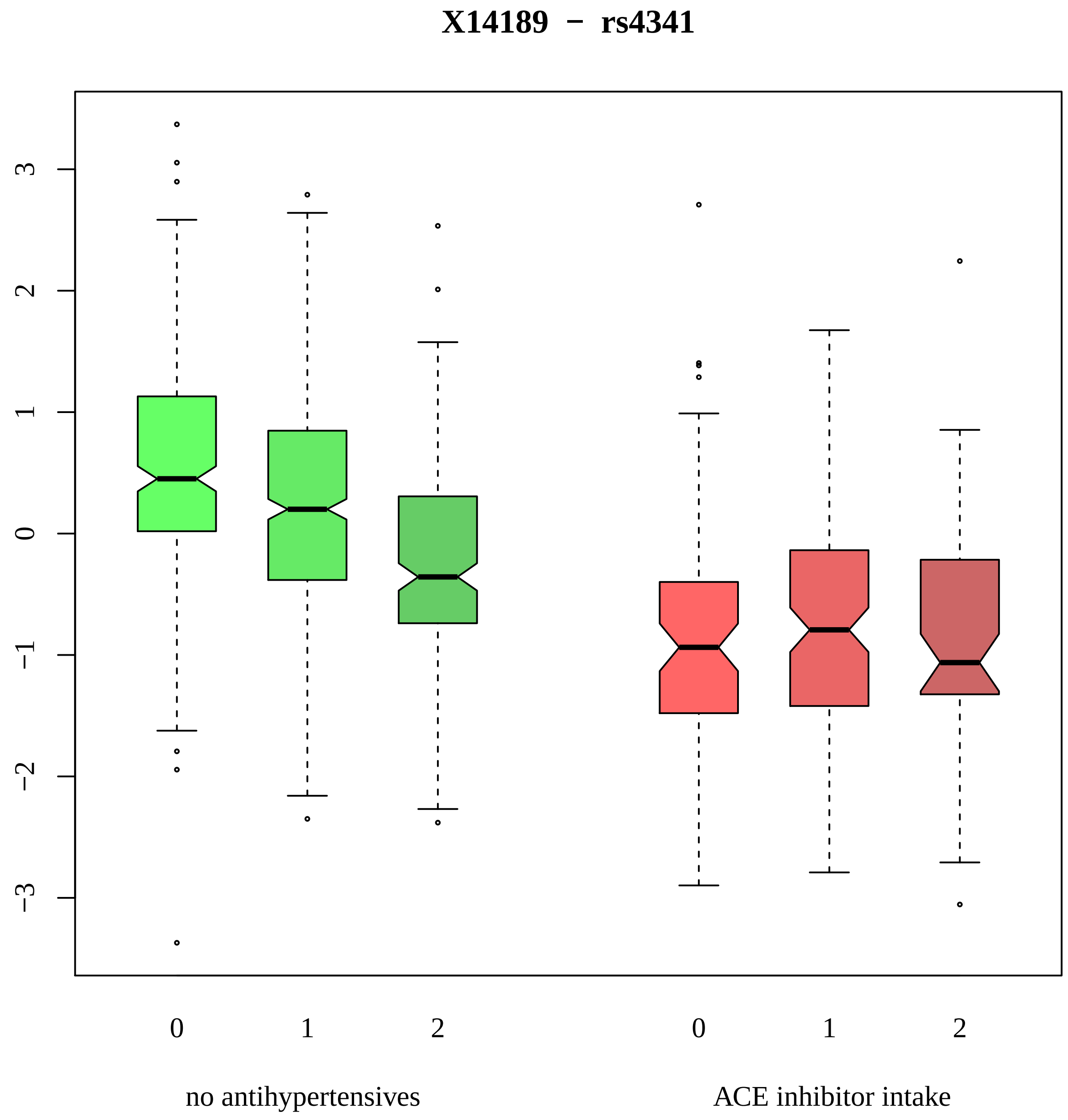

X14189 – rs4341
3
2
1
0
−1
−2
−3
0
1
2
0
1
2
no antihypertensives
ACE inhibitor intake

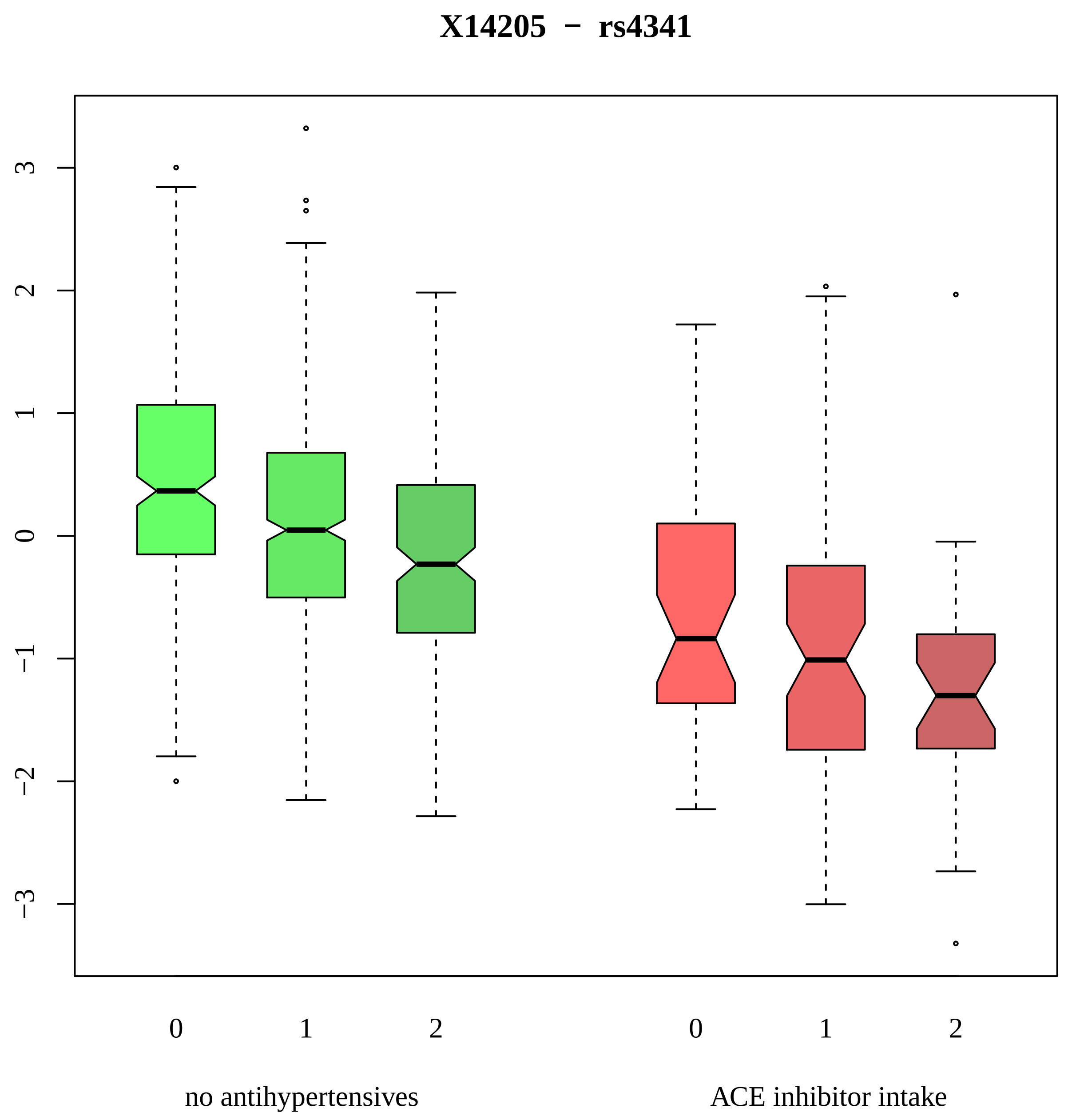
X14205 − rs4341
3
2
1
0
−1
−2
−3
0
1
2
0
1
2
no antihypertensives
ACE inhibitor intake

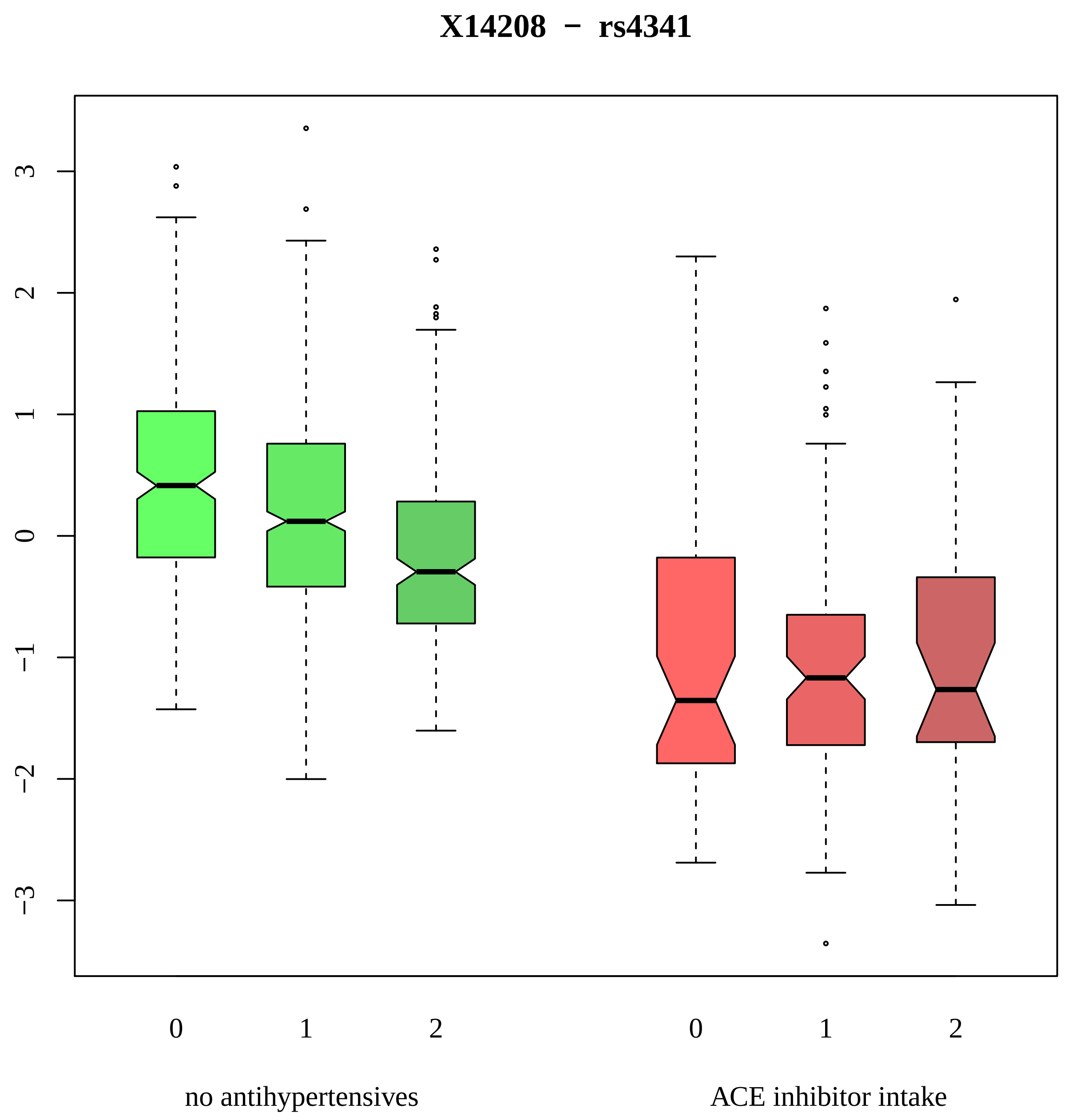

X14208 – rs4341
3
2
1
0
−1
−2
−3
0
1
2
0
1
2
no antihypertensives
ACE inhibitor intake

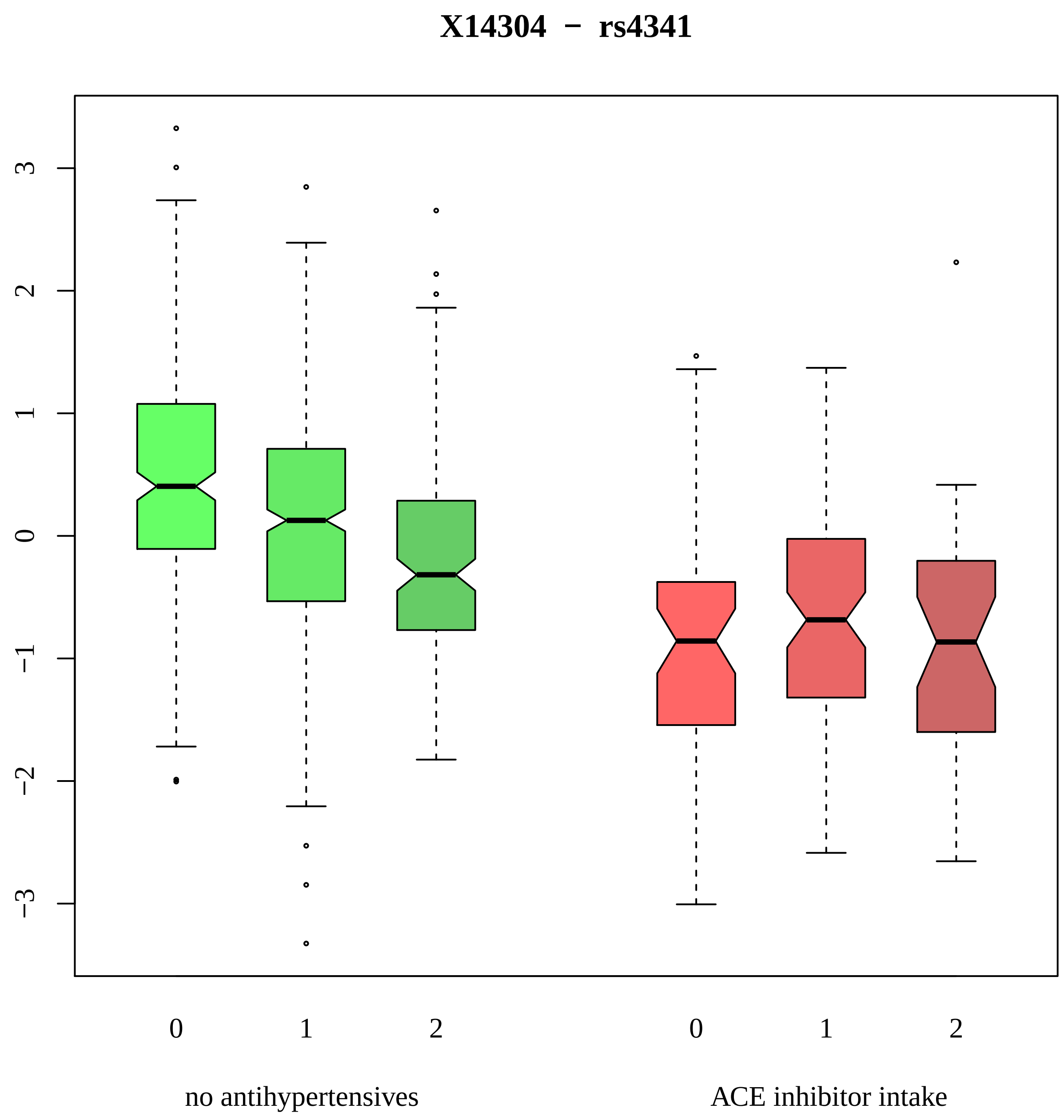

X14304 – rs4341
3
2
1
0
−1
−2
−3
0
1
2
0
1
2
no antihypertensives
ACE inhibitor intake

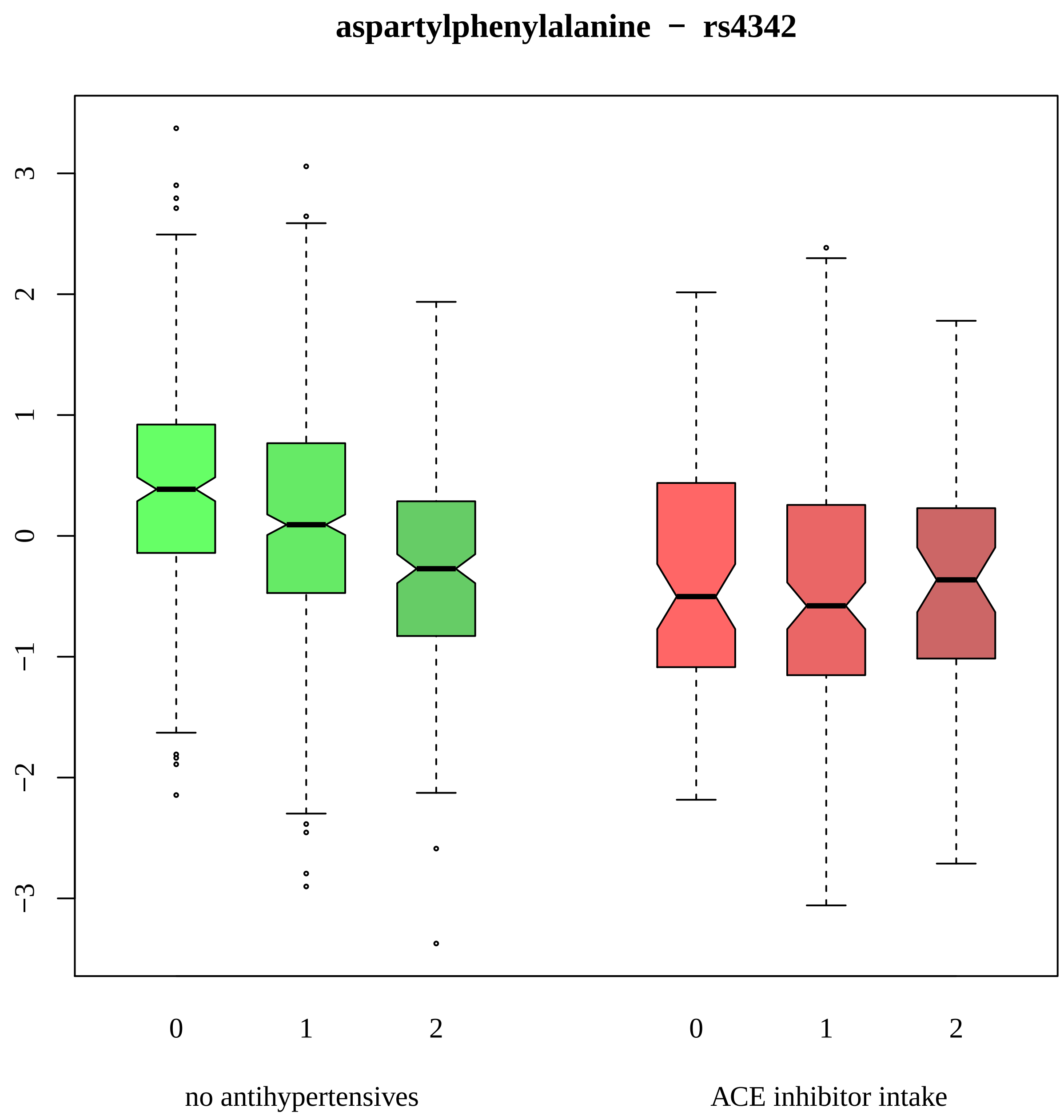
aspartylphenylalanine − rs4342
3
2
1
0
−1
−2
−3
0
1
2
0
1
2
no antihypertensives
ACE inhibitor intake

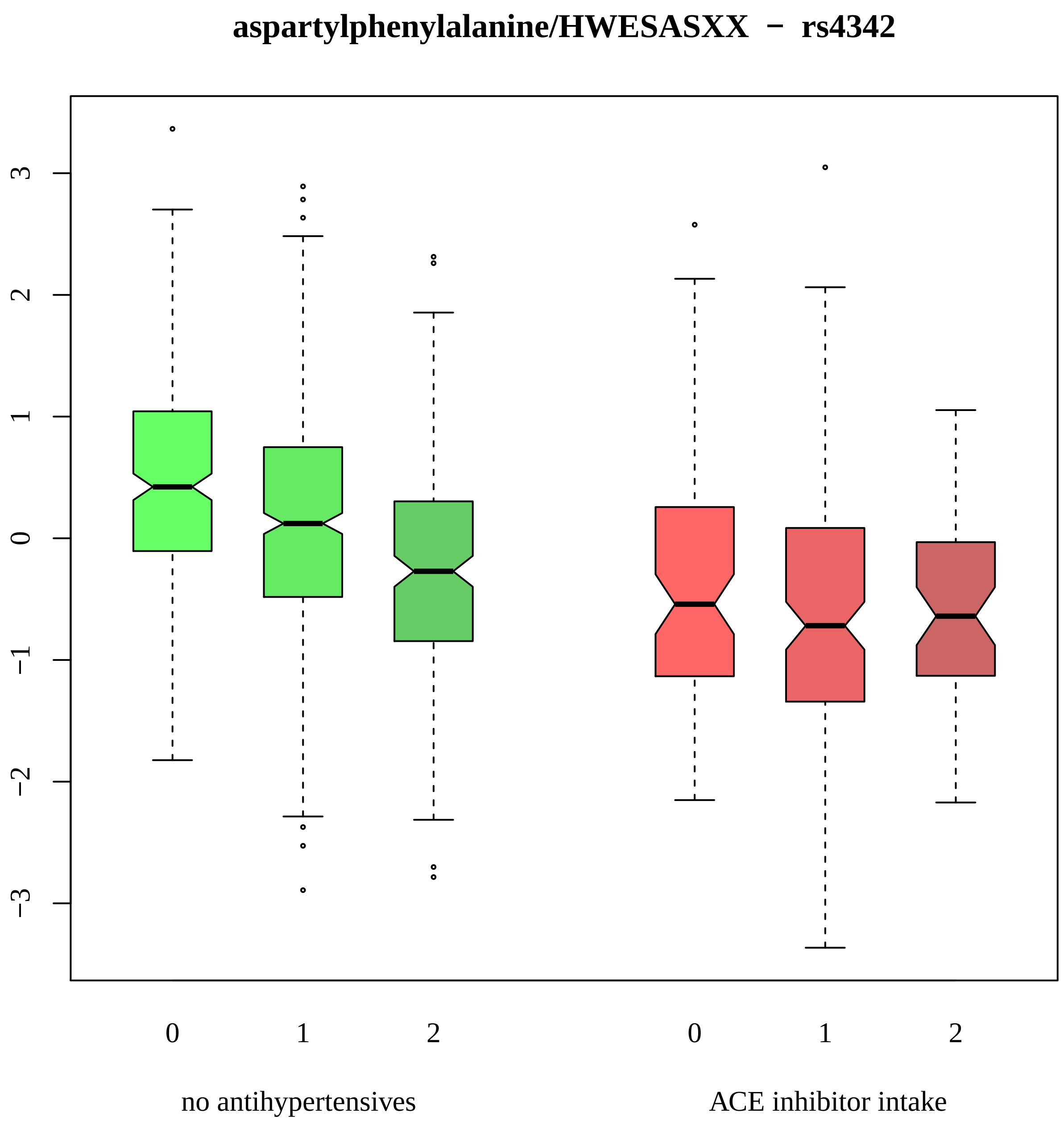

aspartylphenylalanine/HWESASXX – rs4342
3
2
1
0
−1
−2
−3
0
1
2
0
1
2
no antihypertensives
ACE inhibitor intake

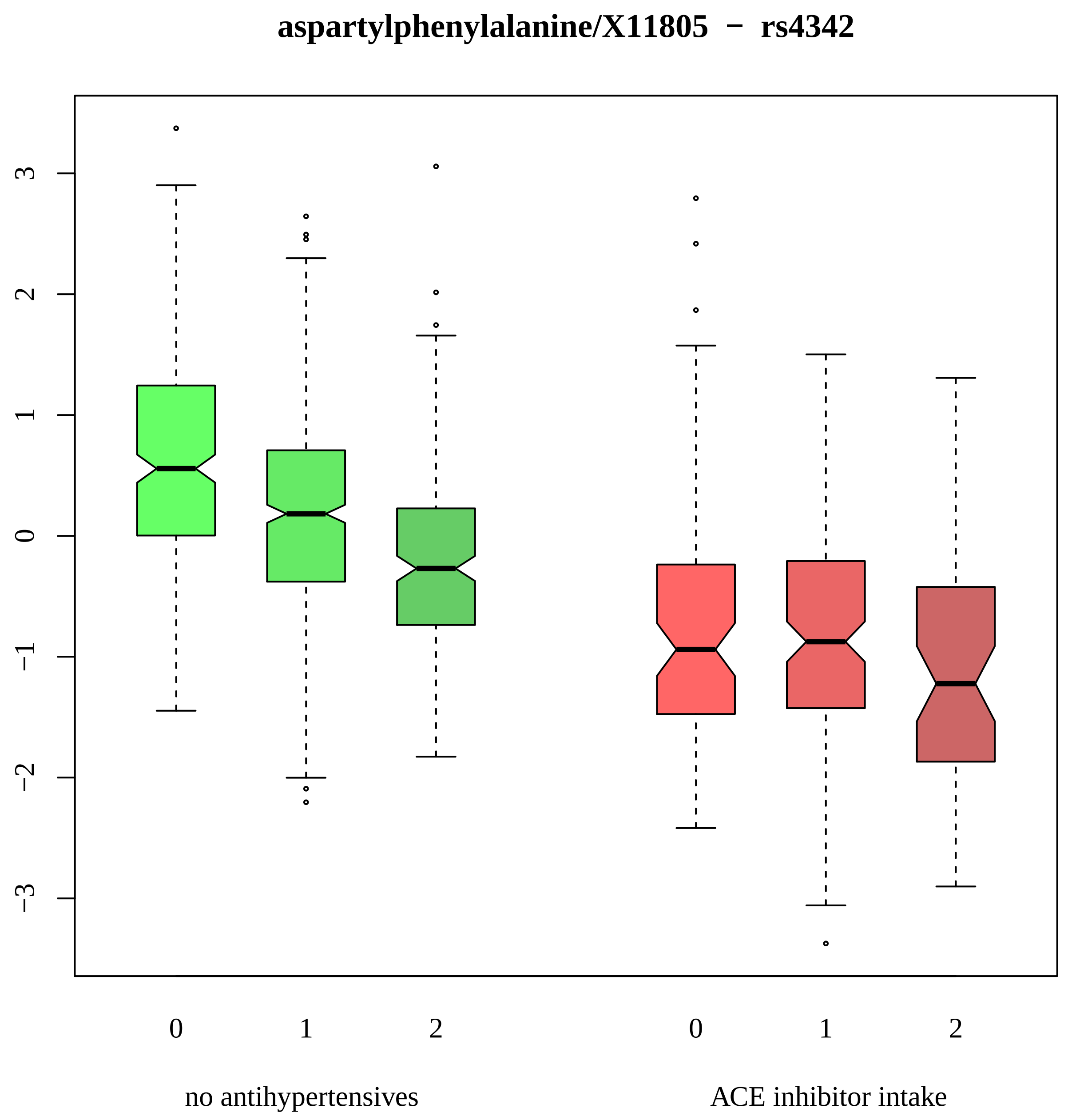
aspartylphenylalanine/X11805 – rs4342
3
2
1
0
−1
−2
−3
0
1
2
0
1
2
no antihypertensives
ACE inhibitor intake

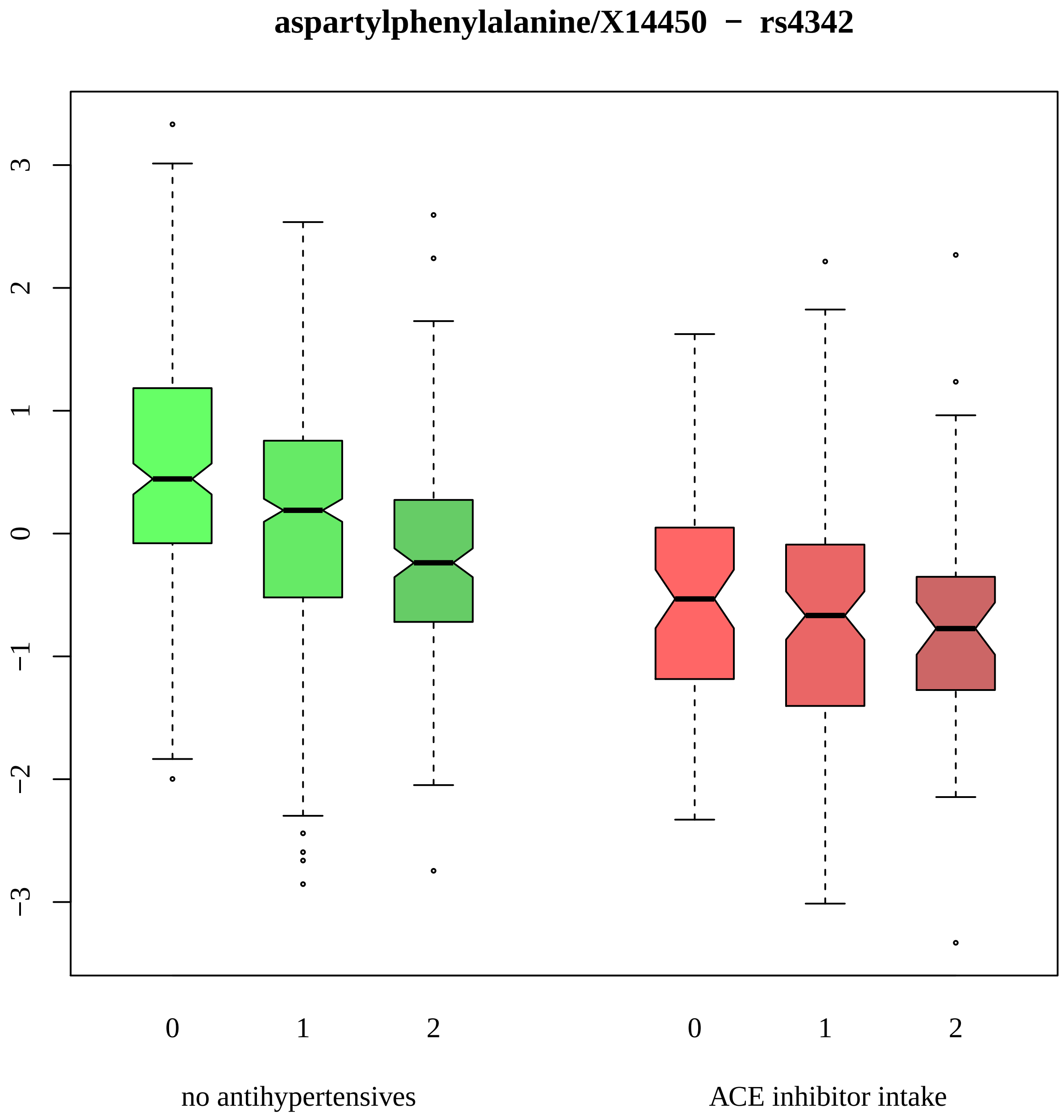

aspartylphenylalanine/X14450 – rs4342
3
2
1
0
−1
−2
−3
0
1
2
0
1
2
no antihypertensives
ACE inhibitor intake

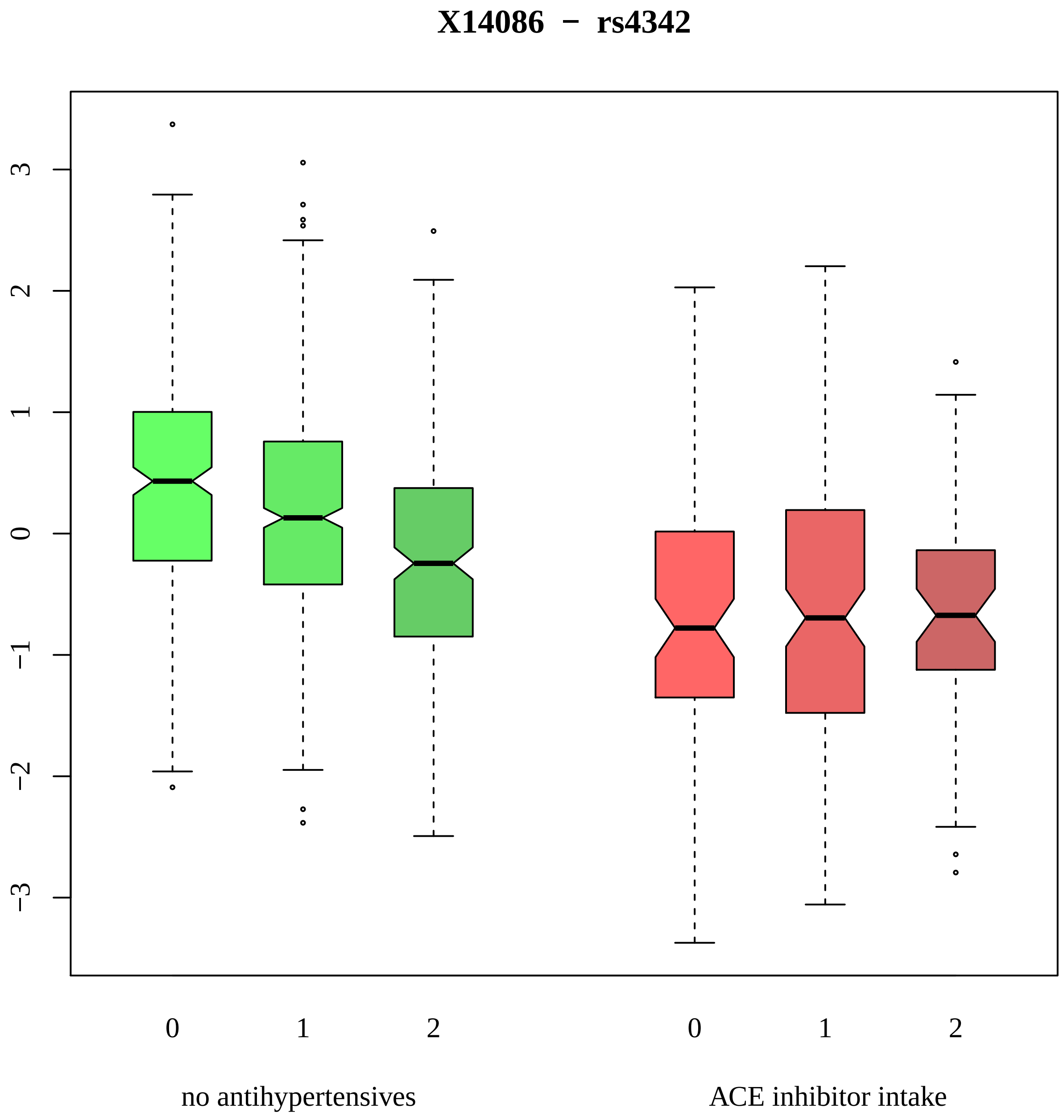

X14086 – rs4342
3
2
1
0
−1
−2
−3
0
1
2
0
1
2
no antihypertensives
ACE inhibitor intake

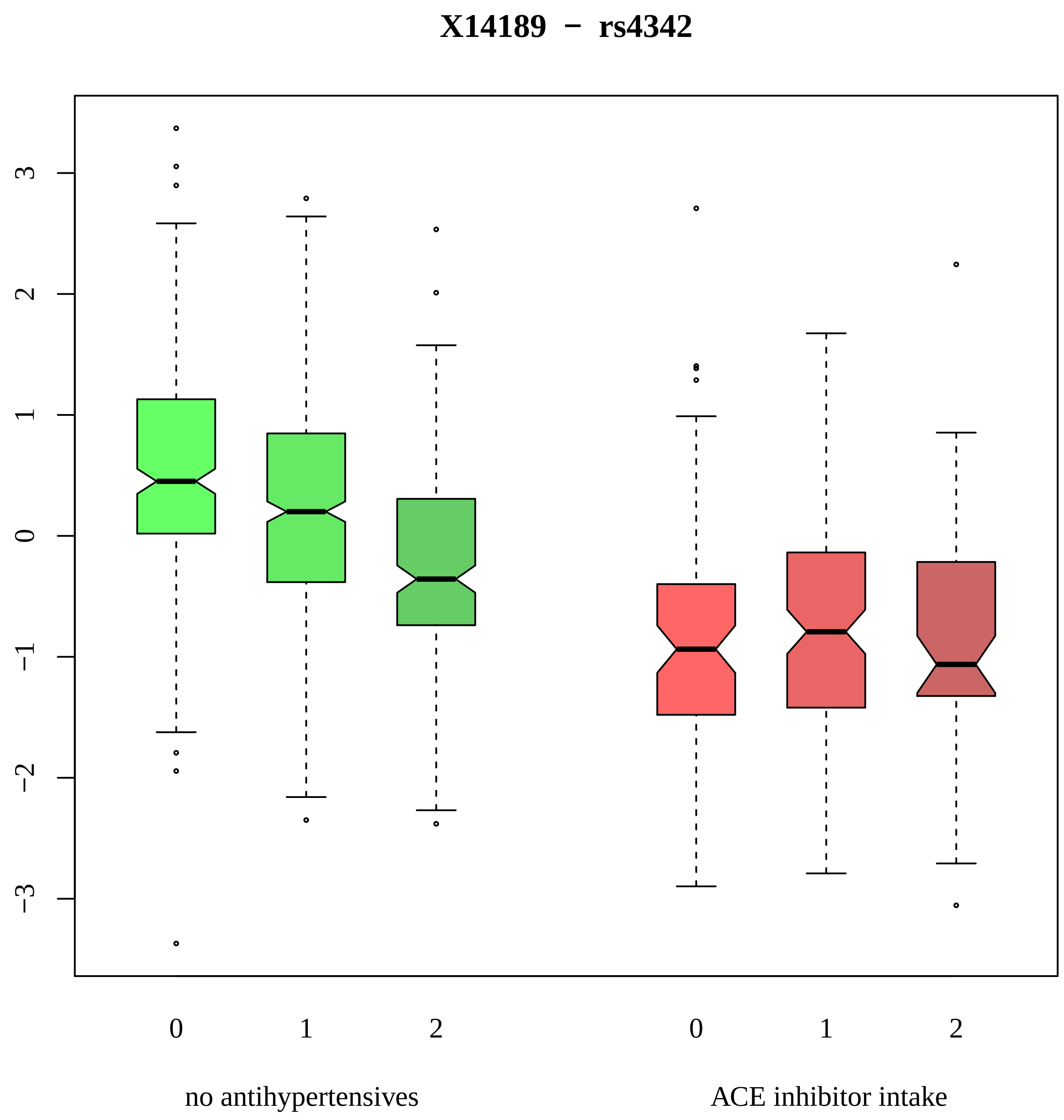

X14189 – rs4342
3
2
1
0
−1
−2
−3
0
1
2
0
1
2
no antihypertensives
ACE inhibitor intake

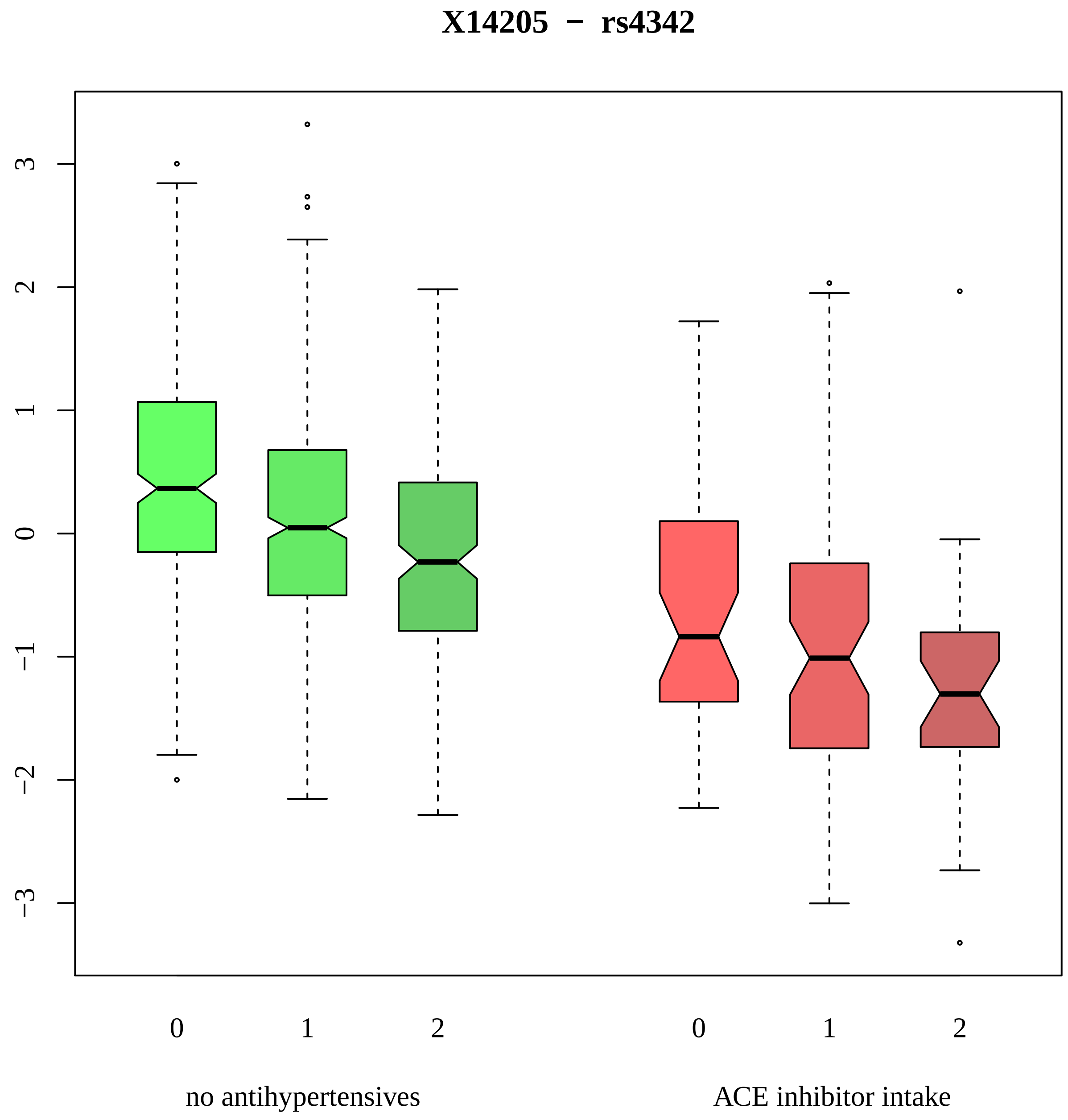

X14205 – rs4342
3
2
1
0
−1
−2
−3
0
1
2
0
1
2
no antihypertensives
ACE inhibitor intake

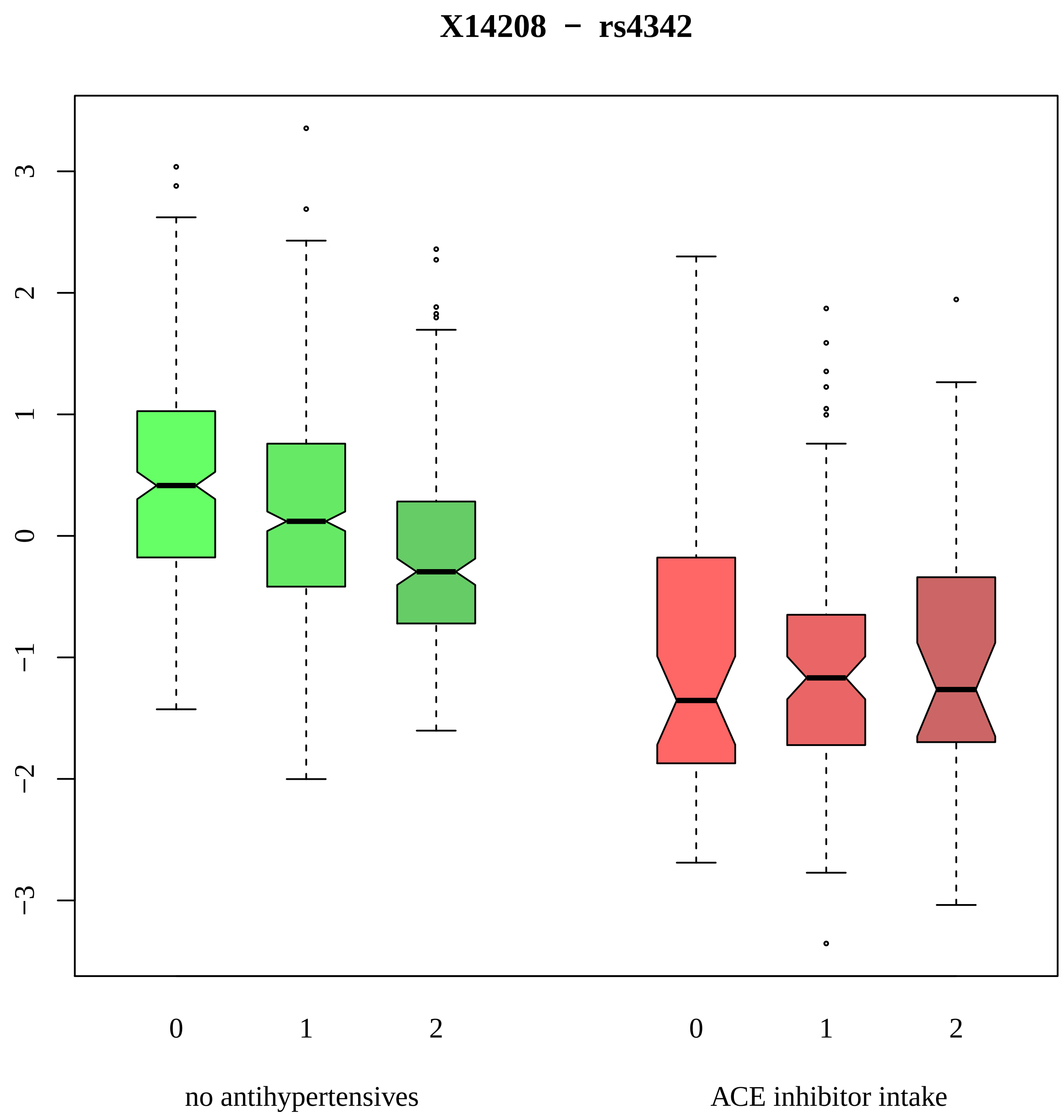

X14208 – rs4342
3
2
1
0
−1
−2
−3
0
1
2
0
1
2
no antihypertensives
ACE inhibitor intake

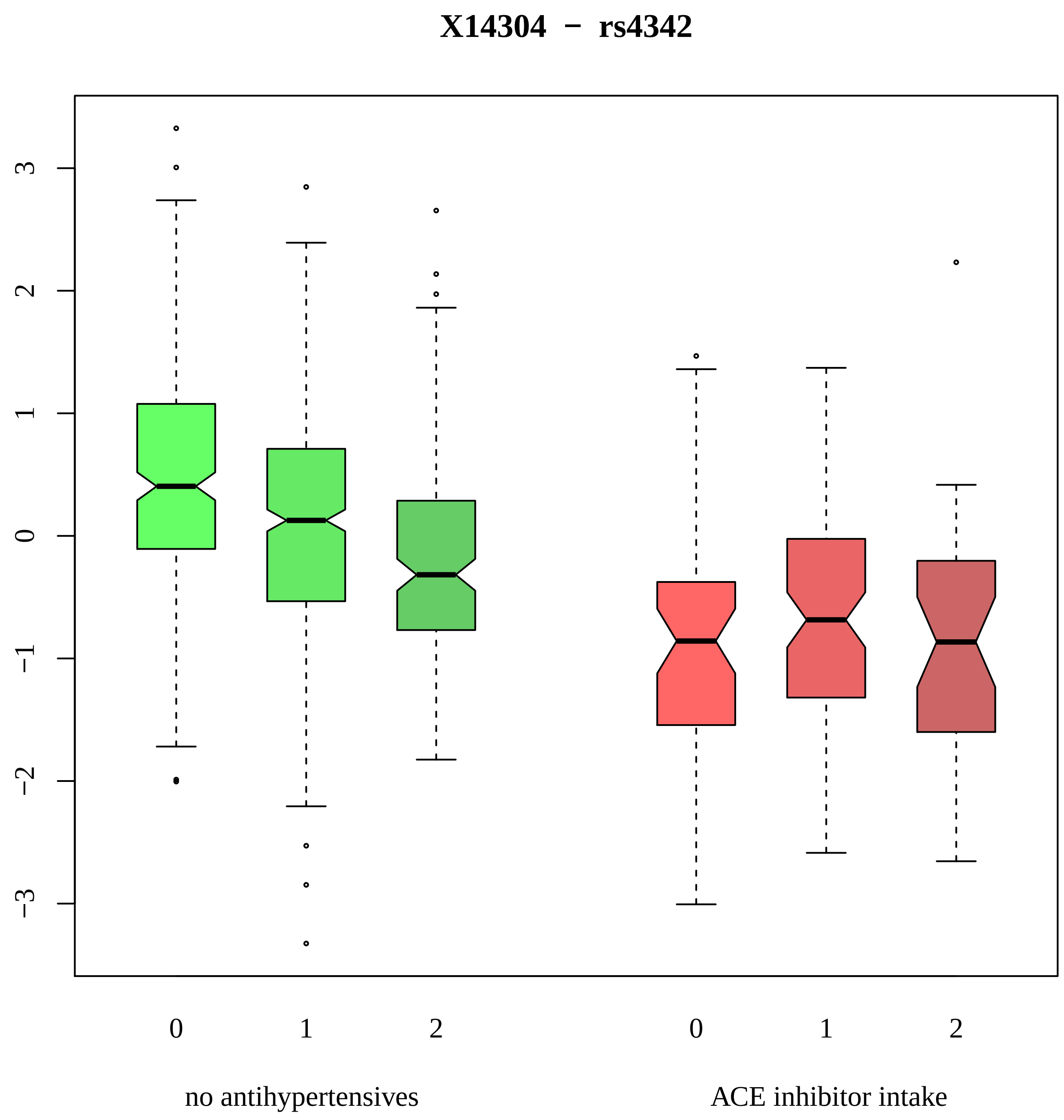
X14304 – rs4342
3
2
1
0
−1
−2
−3
0
1
2
0
1
2
no antihypertensives
ACE inhibitor intake

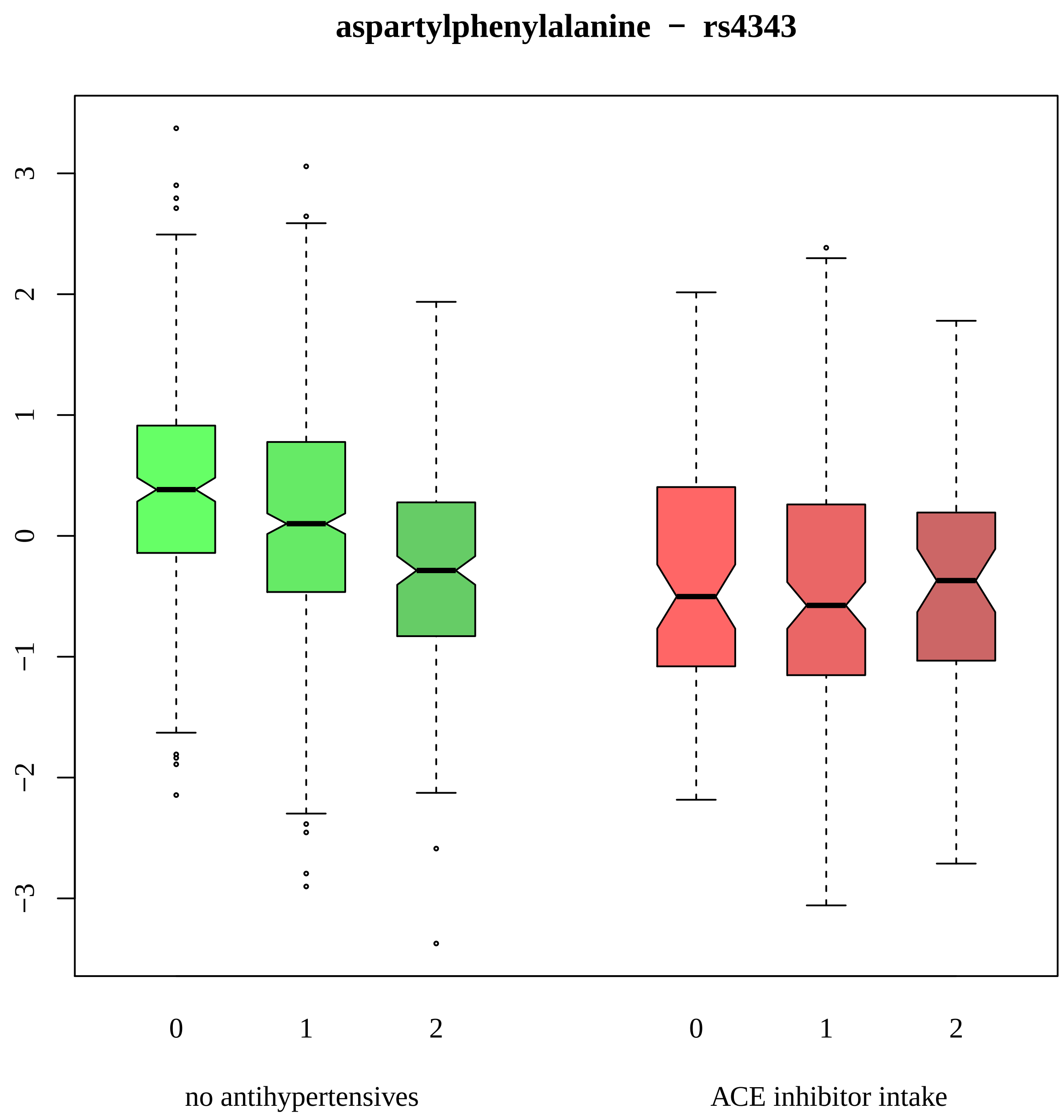

aspartylphenylalanine − rs4343
3
2
1
0
−1
−2
−3
0
1
2
0
1
2
no antihypertensives
ACE inhibitor intake

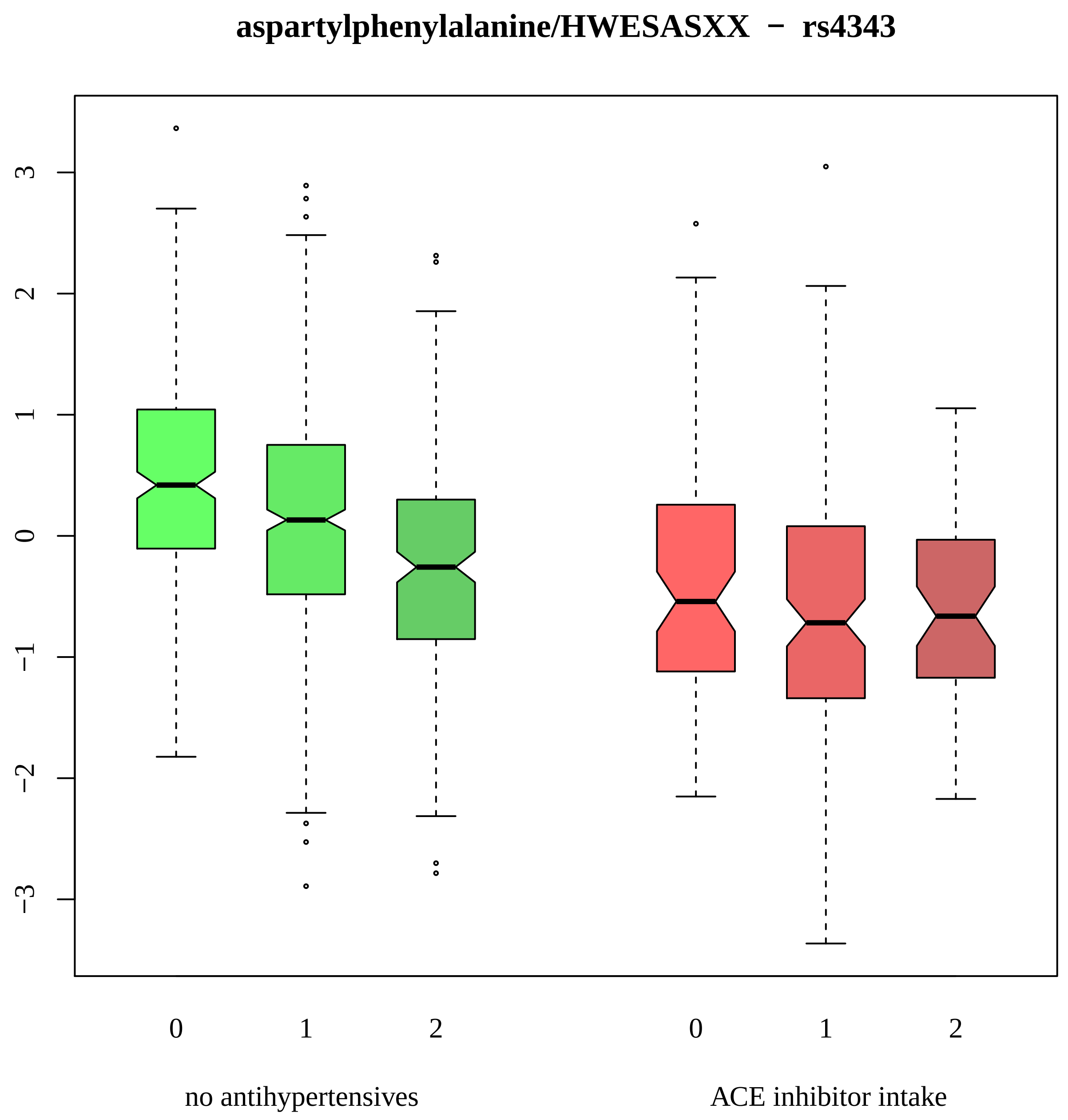
aspartylphenylalanine/HWESASXX – rs4343
3
2
1
0
−1
−2
−3
0
1
2
0
1
2
no antihypertensives
ACE inhibitor intake

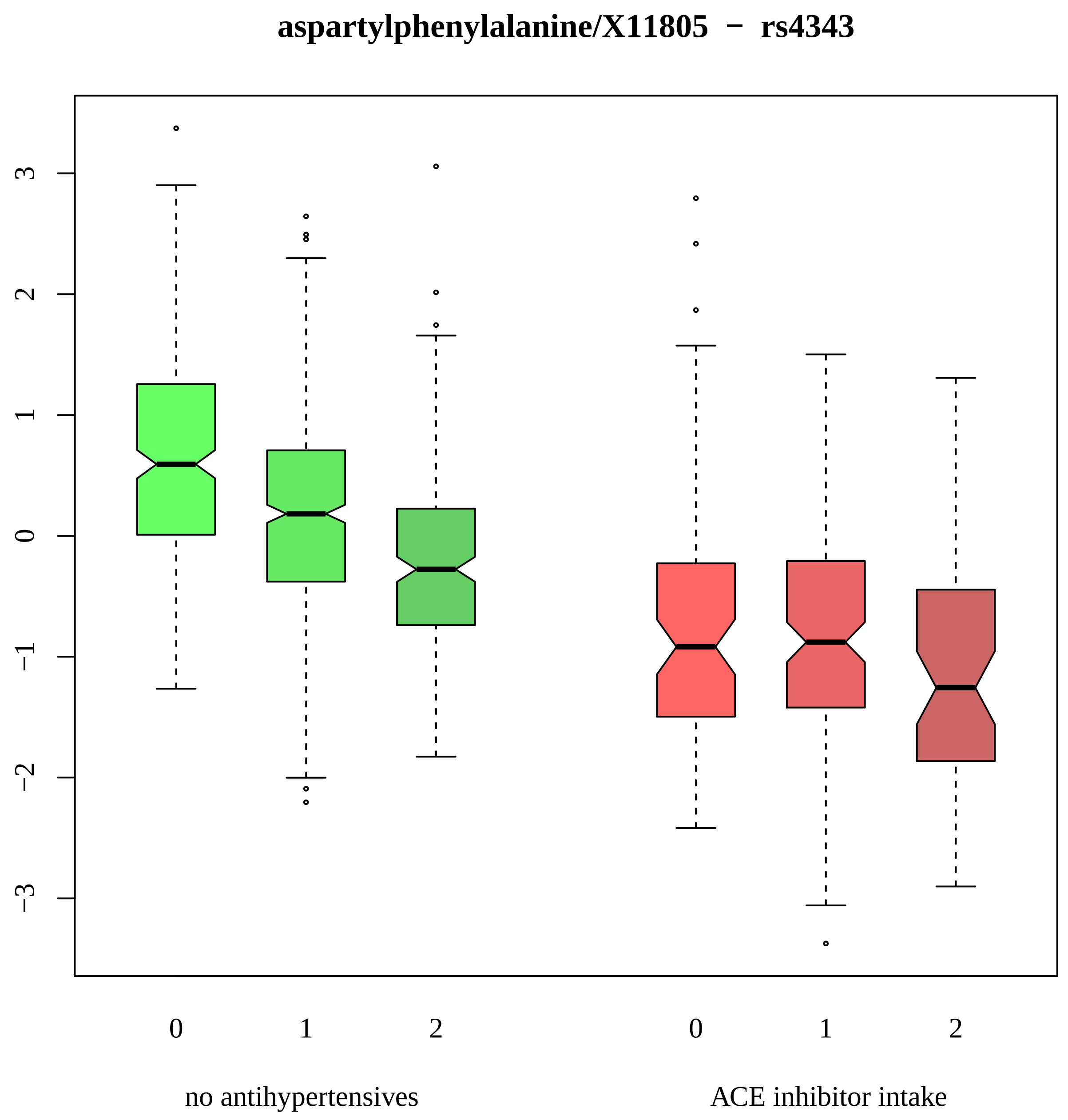
aspartylphenylalanine/X11805 – rs4343
3
2
1
0
−1
−2
−3
0
1
2
0
1
2
no antihypertensives
ACE inhibitor intake

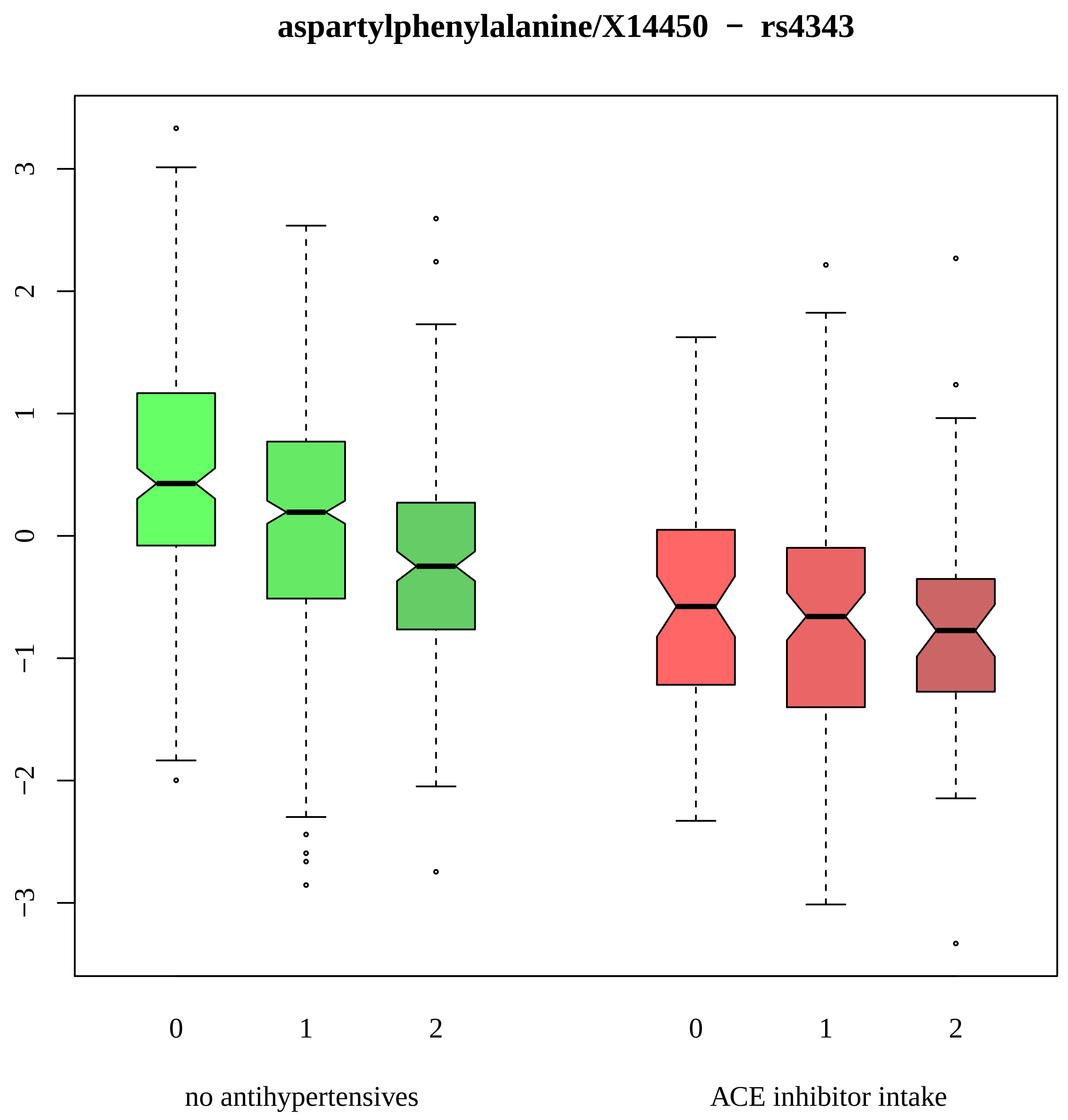
aspartylphenylalanine/X14450 – rs4343
3
2
1
0
−1
−2
−3
0
1
2
0
1
2
no antihypertensives
ACE inhibitor intake

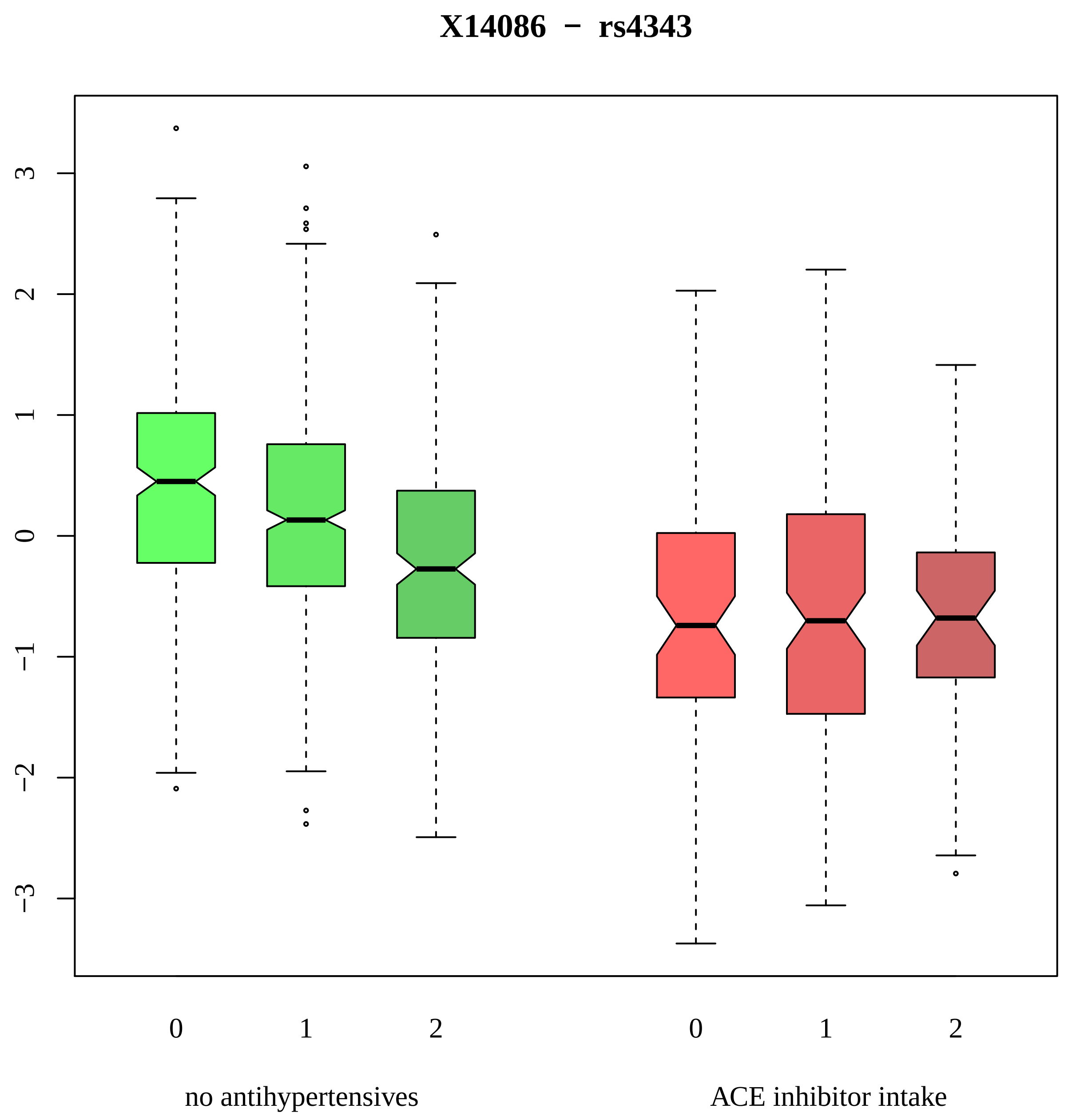
X14086 – rs4343
3
2
1
0
−1
−2
−3
0
1
2
0
1
2
no antihypertensives
ACE inhibitor intake

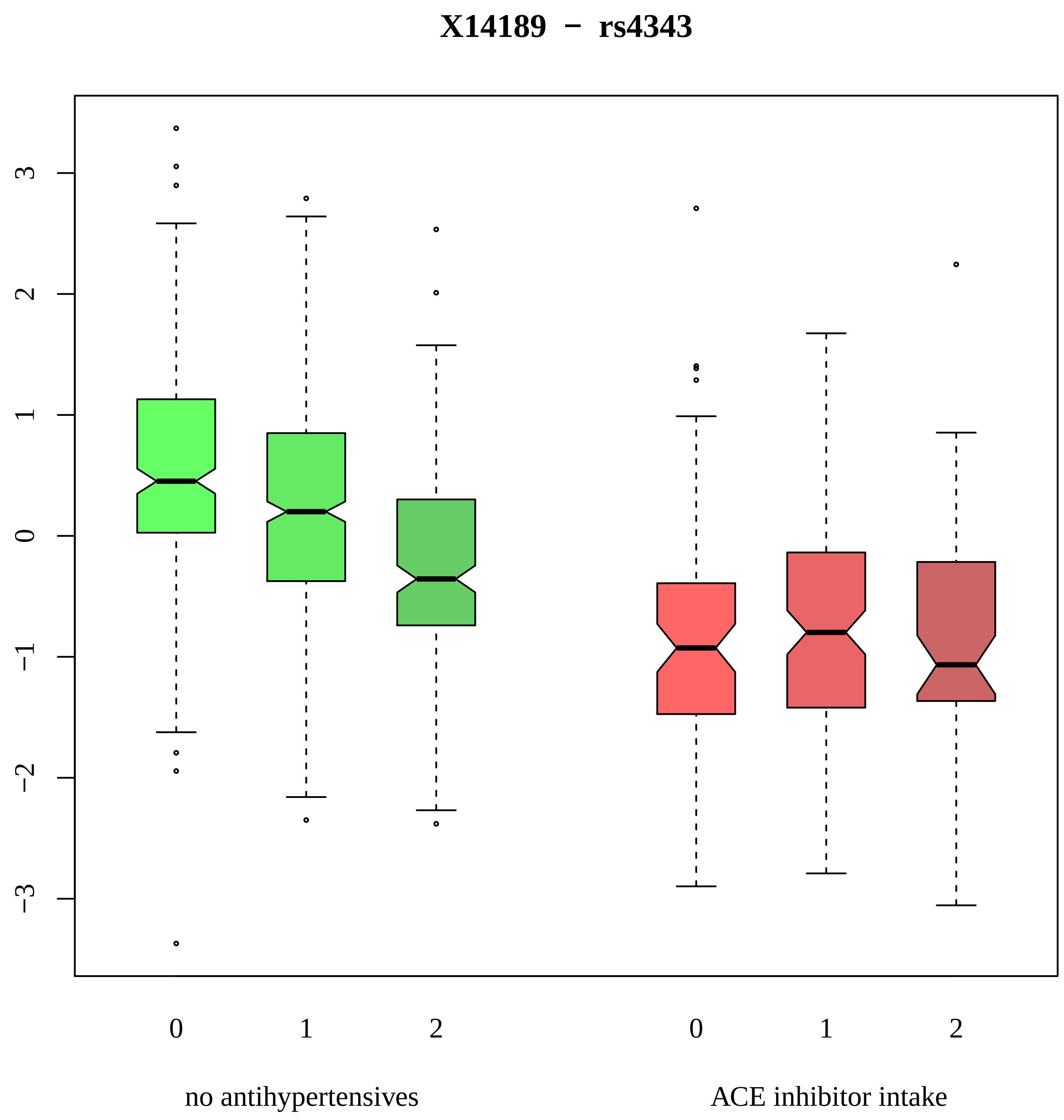
X14189 – rs4343
3
2
1
0
−1
−2
−3
0
1
2
0
1
2
no antihypertensives
ACE inhibitor intake

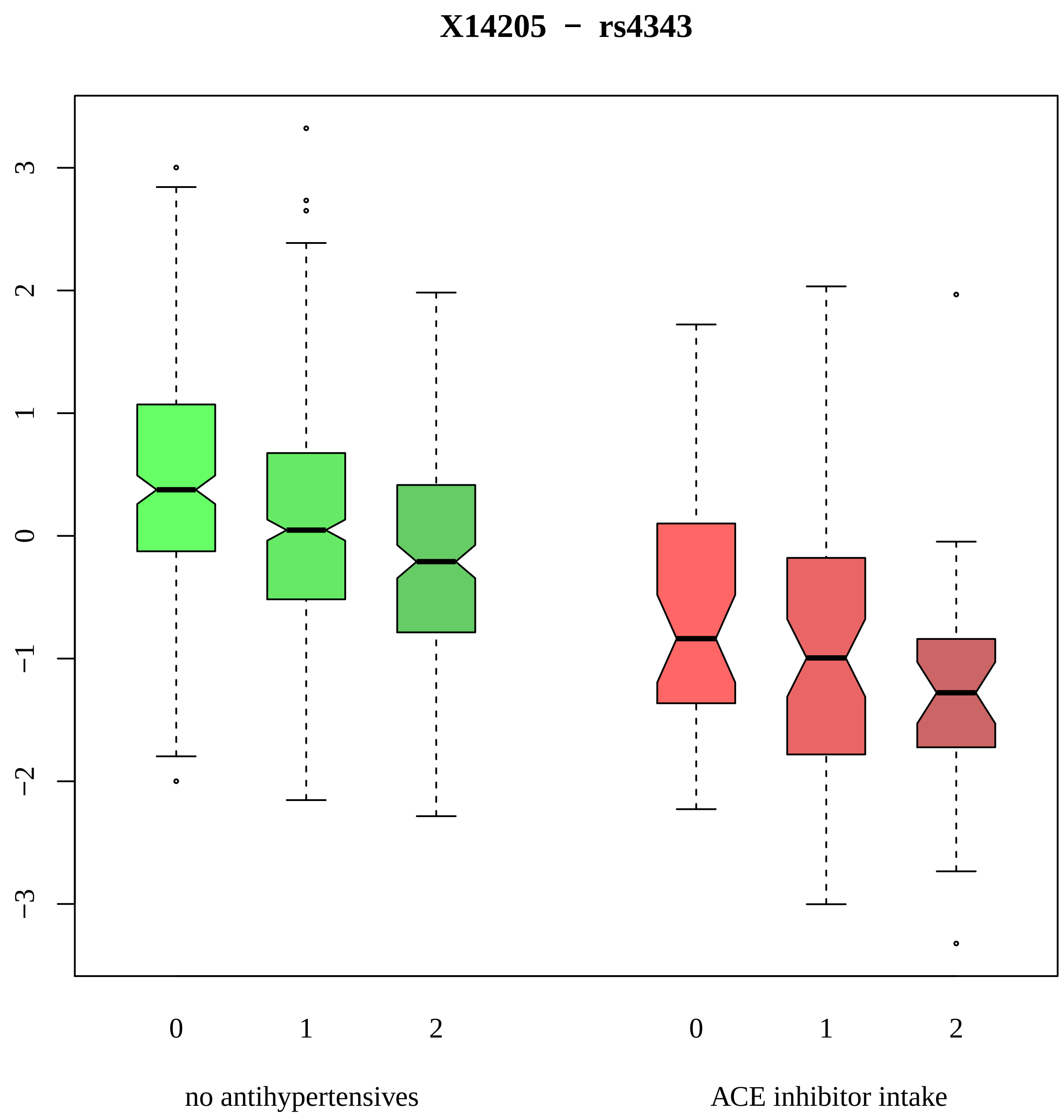
X14205 – rs4343
3
2
1
0
−1
−2
−3
0
1
2
0
1
2
no antihypertensives
ACE inhibitor intake

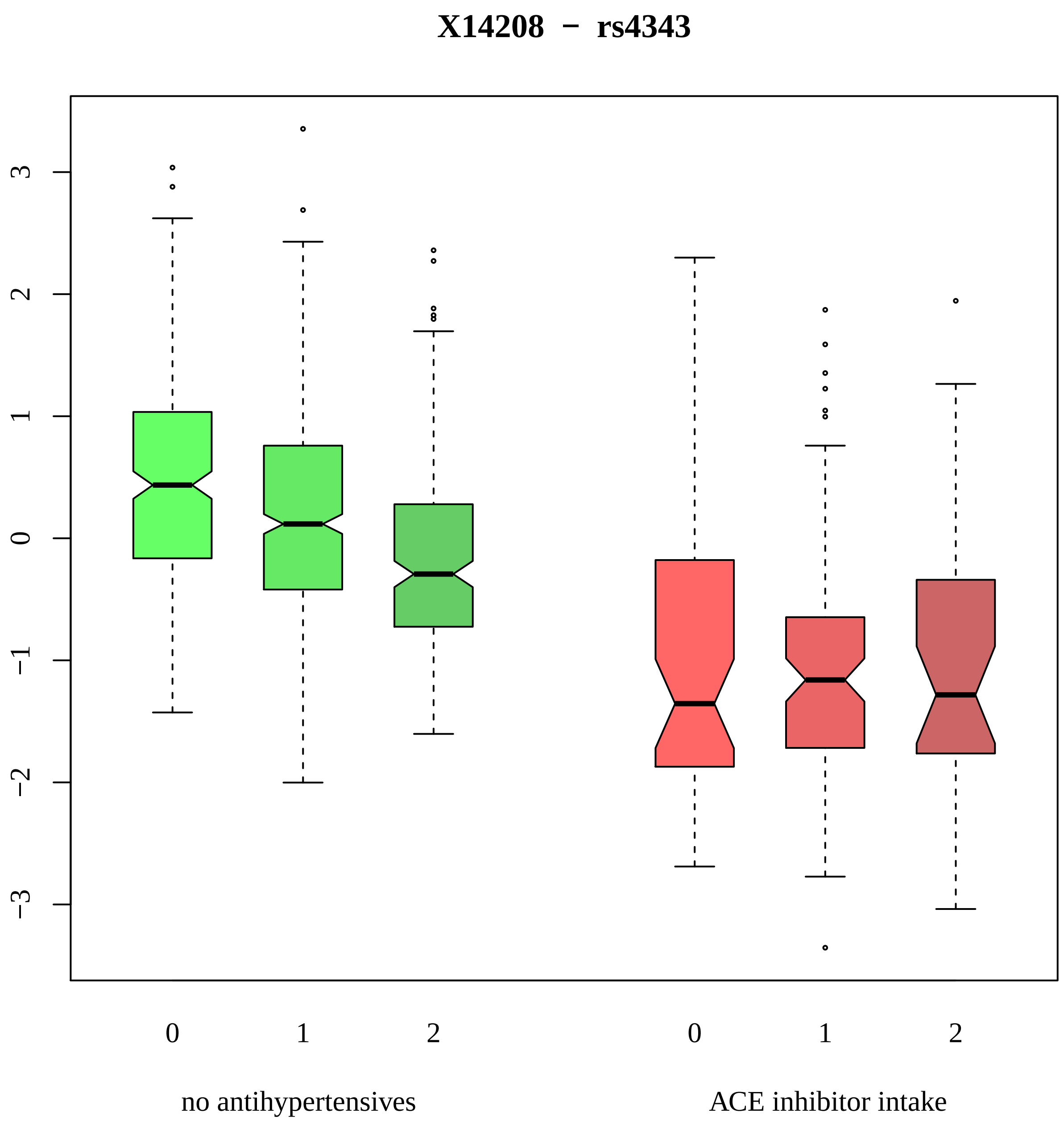

X14208 – rs4343
3
2
1
0
−1
−2
−3
0
1
2
0
1
2
no antihypertensives
ACE inhibitor intake

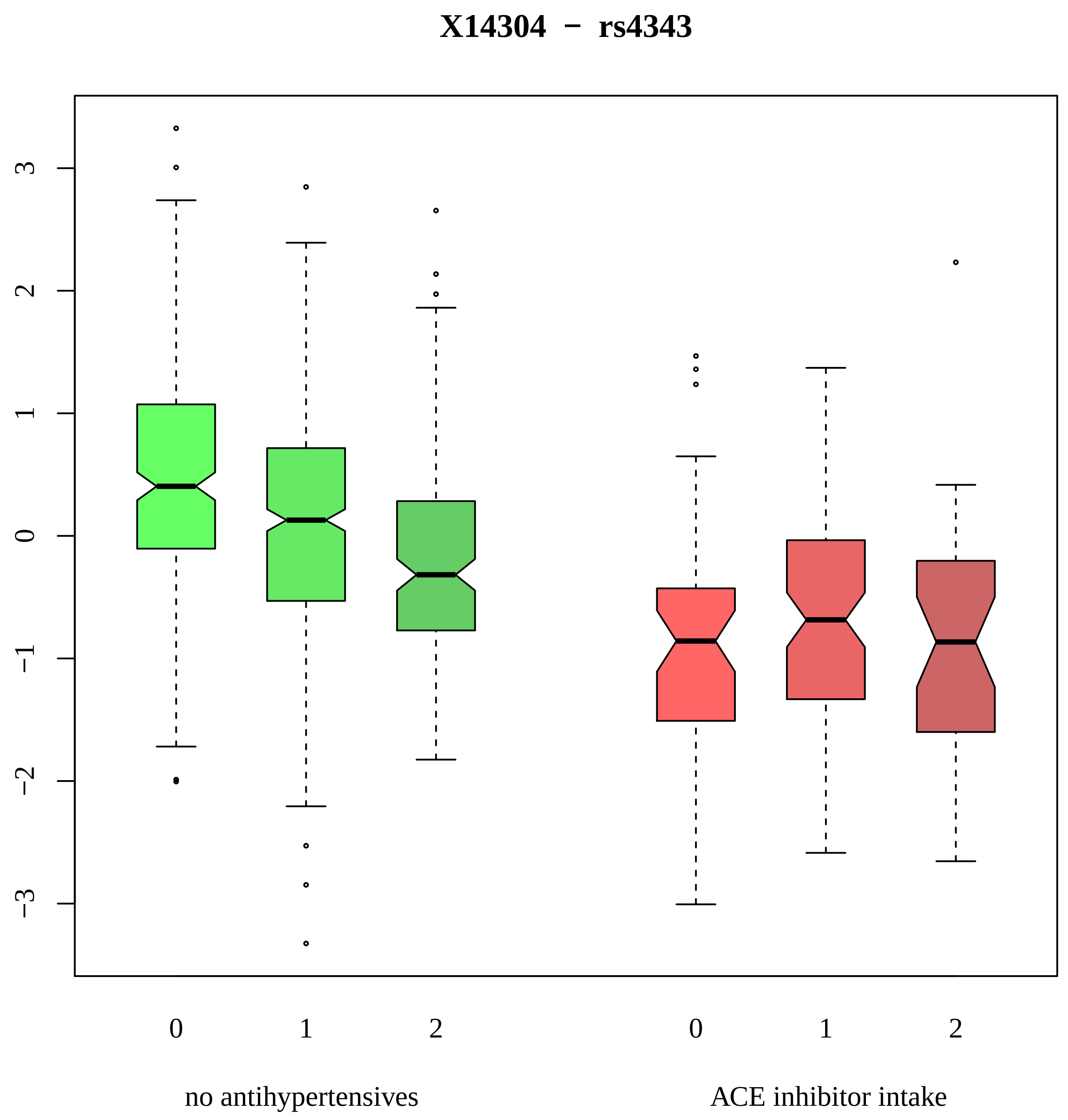
X14304 – rs4343
3
2
1
0
−1
−2
−3
0
1
2
0
1
2
no antihypertensives
ACE inhibitor intake

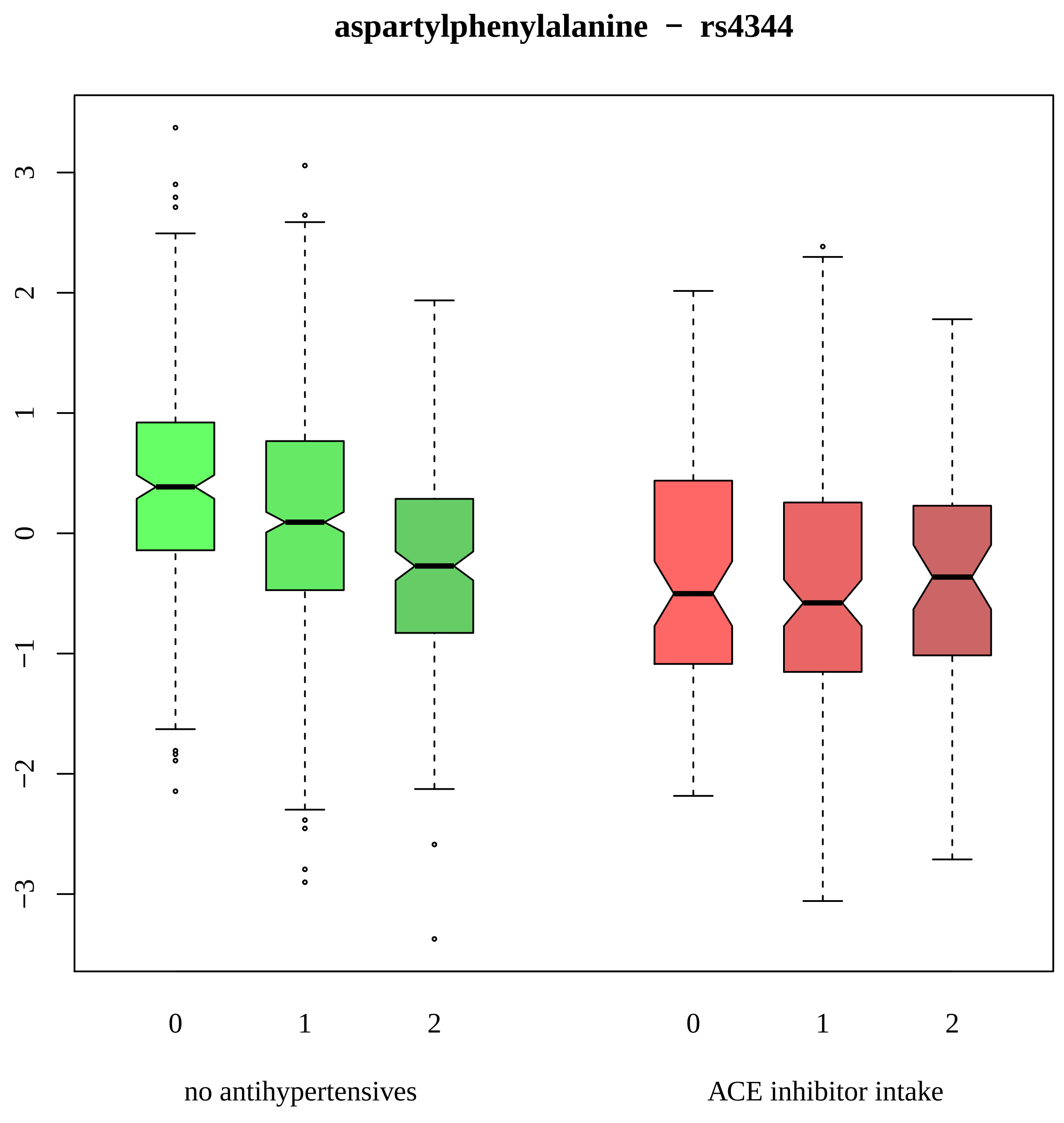

aspartylphenylalanine − rs4344
3
2
1
0
−1
−2
−3
0
1
2
0
1
2
no antihypertensives
ACE inhibitor intake

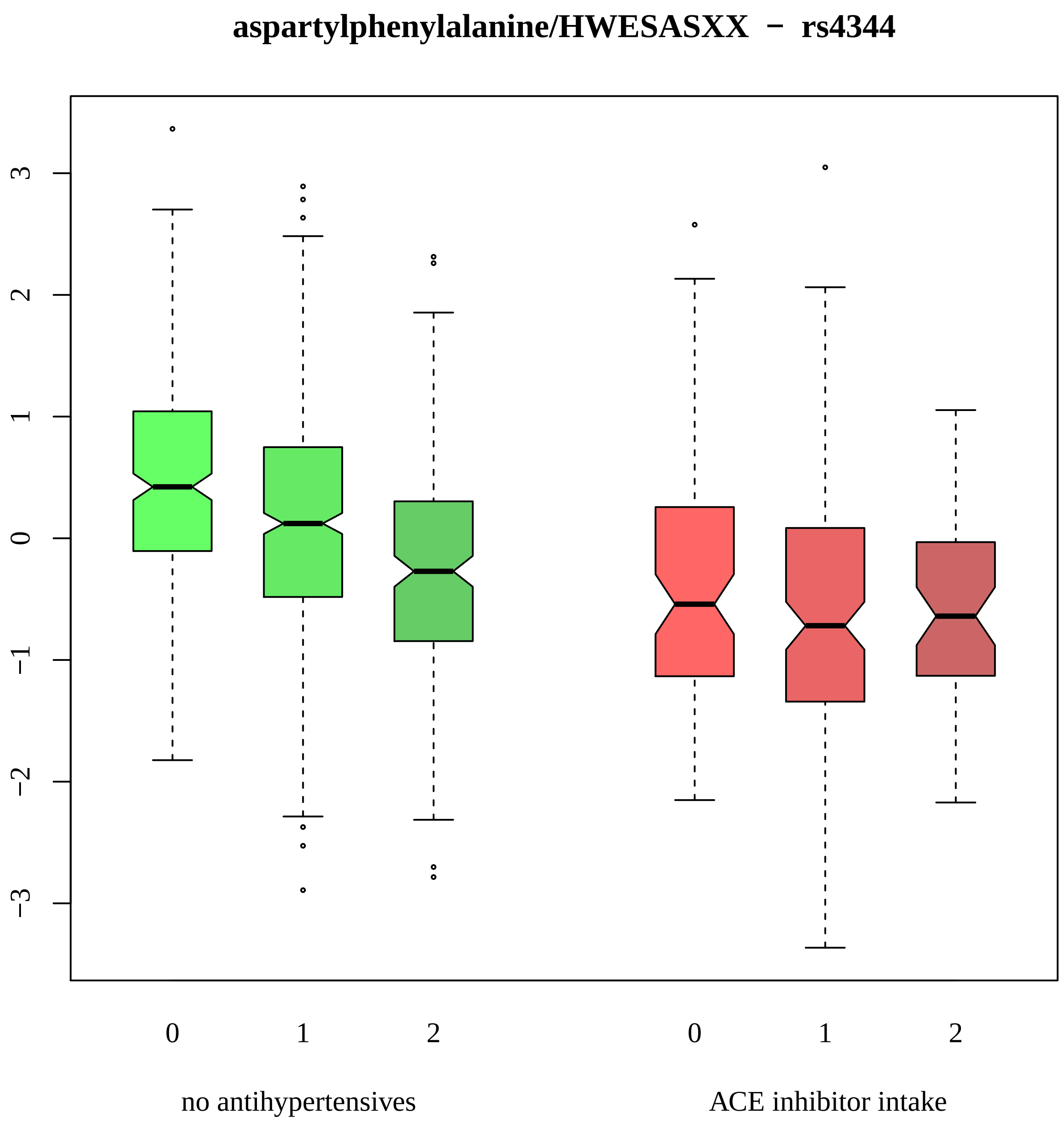

aspartylphenylalanine/HWESASXX – rs4344
3
2
1
0
−1
−2
−3
0
1
2
0
1
2
no antihypertensives
ACE inhibitor intake

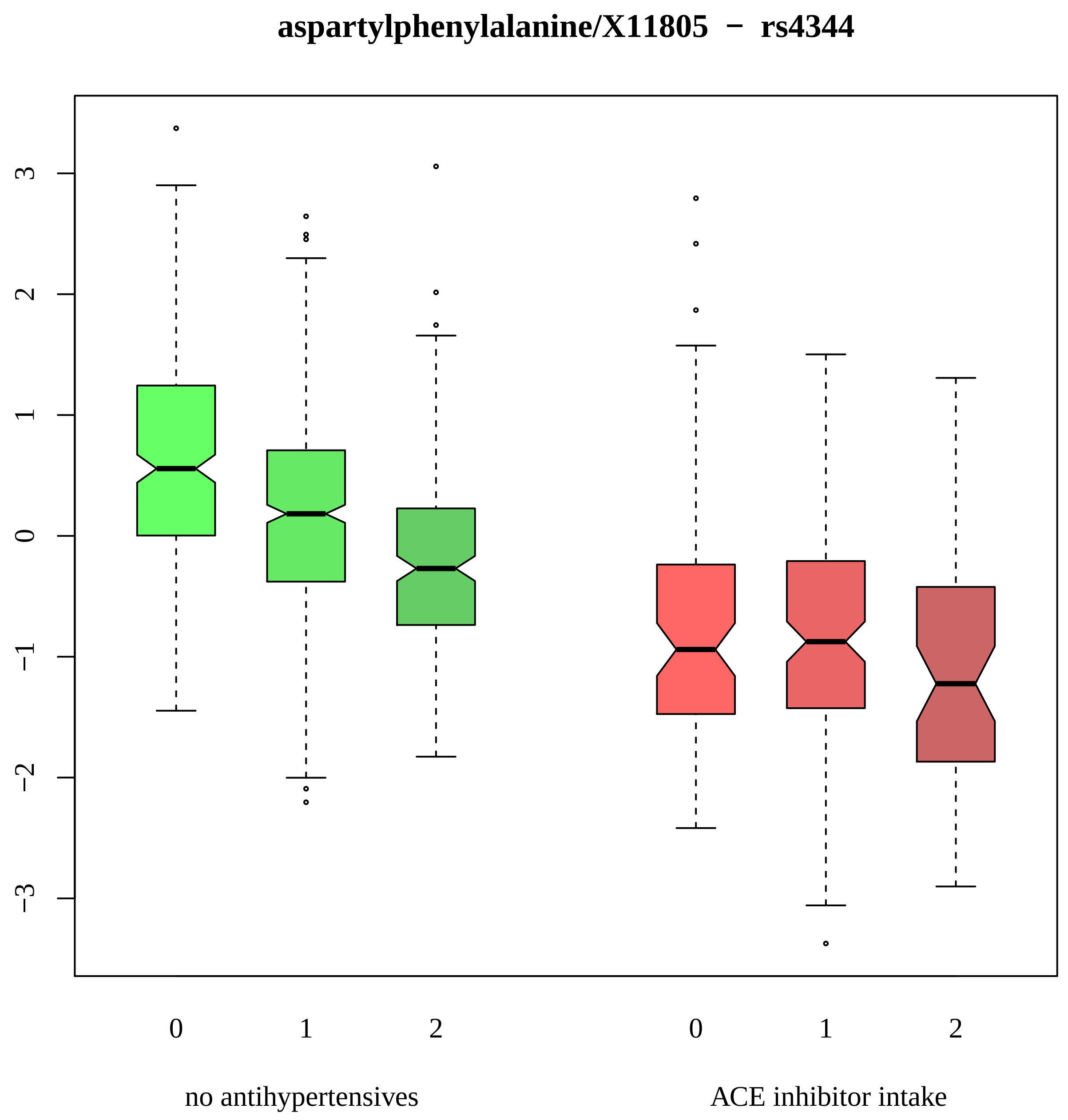

aspartylphenylalanine/X11805 − rs4344
3
2
1
0
−1
−2
−3
0
1
2
0
1
2
no antihypertensives
ACE inhibitor intake

aspartylphenylalanine/X14450 – rs4344

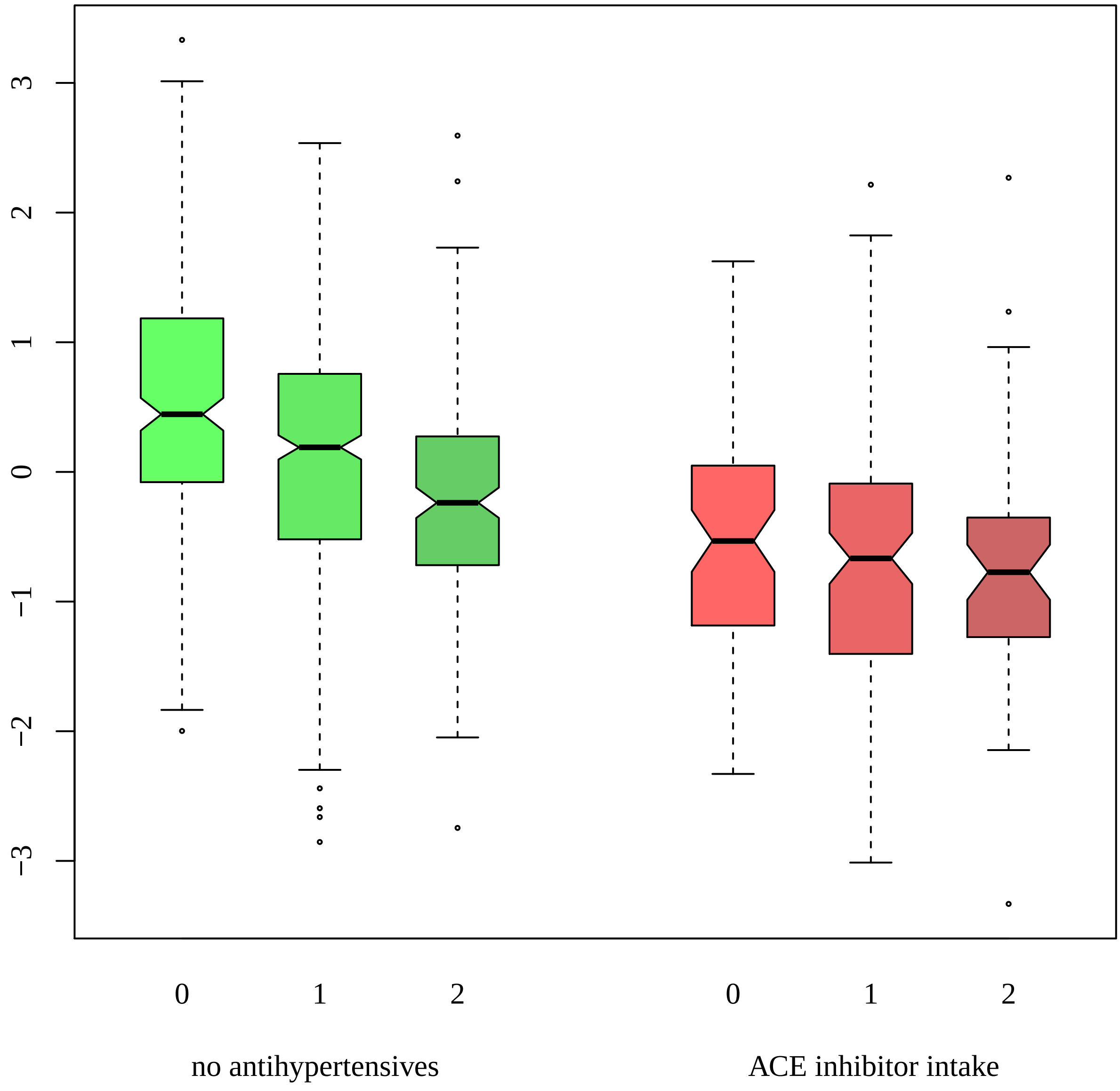

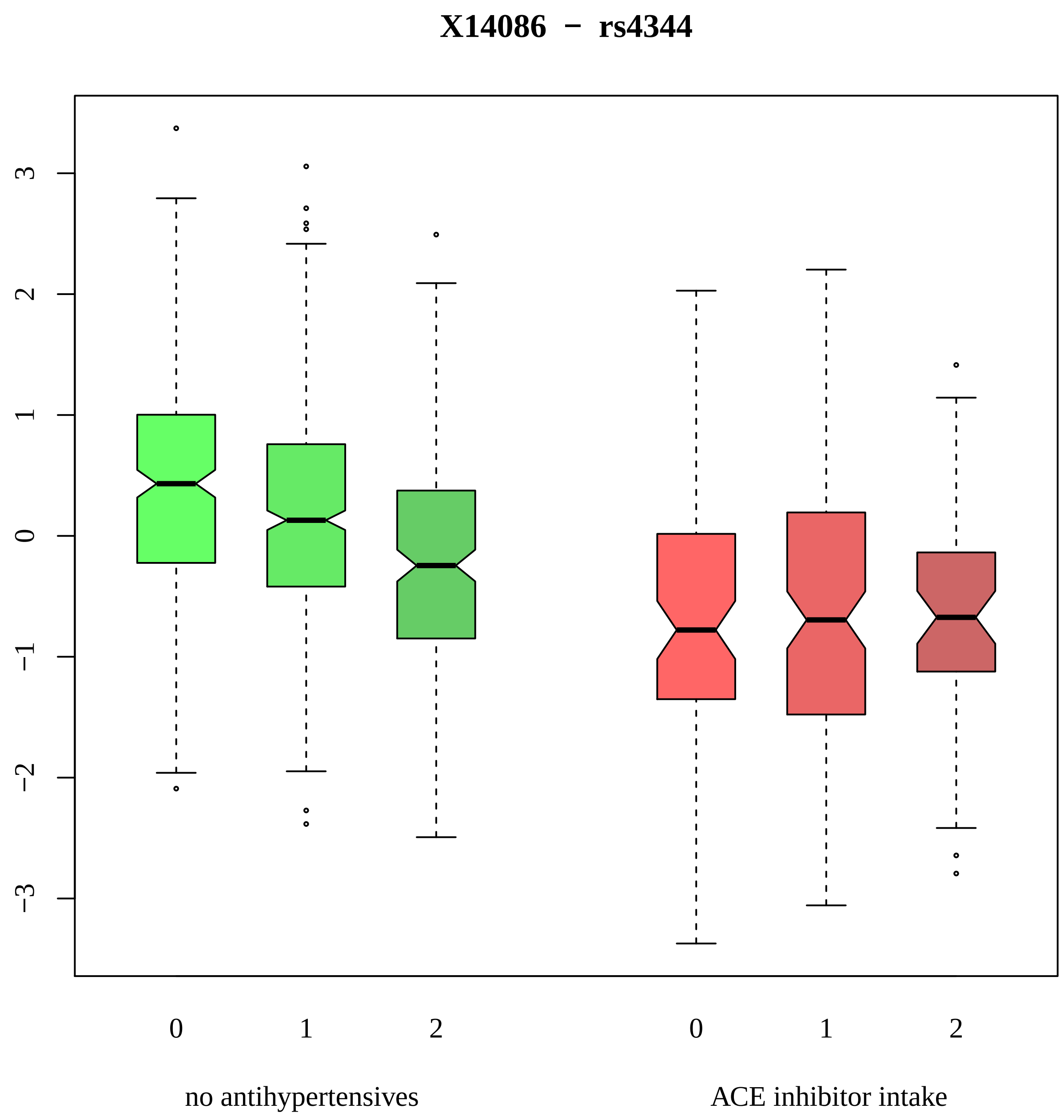
X14086 – rs4344
3
2
1
0
−1
−2
−3
0
1
2
0
1
2
no antihypertensives
ACE inhibitor intake

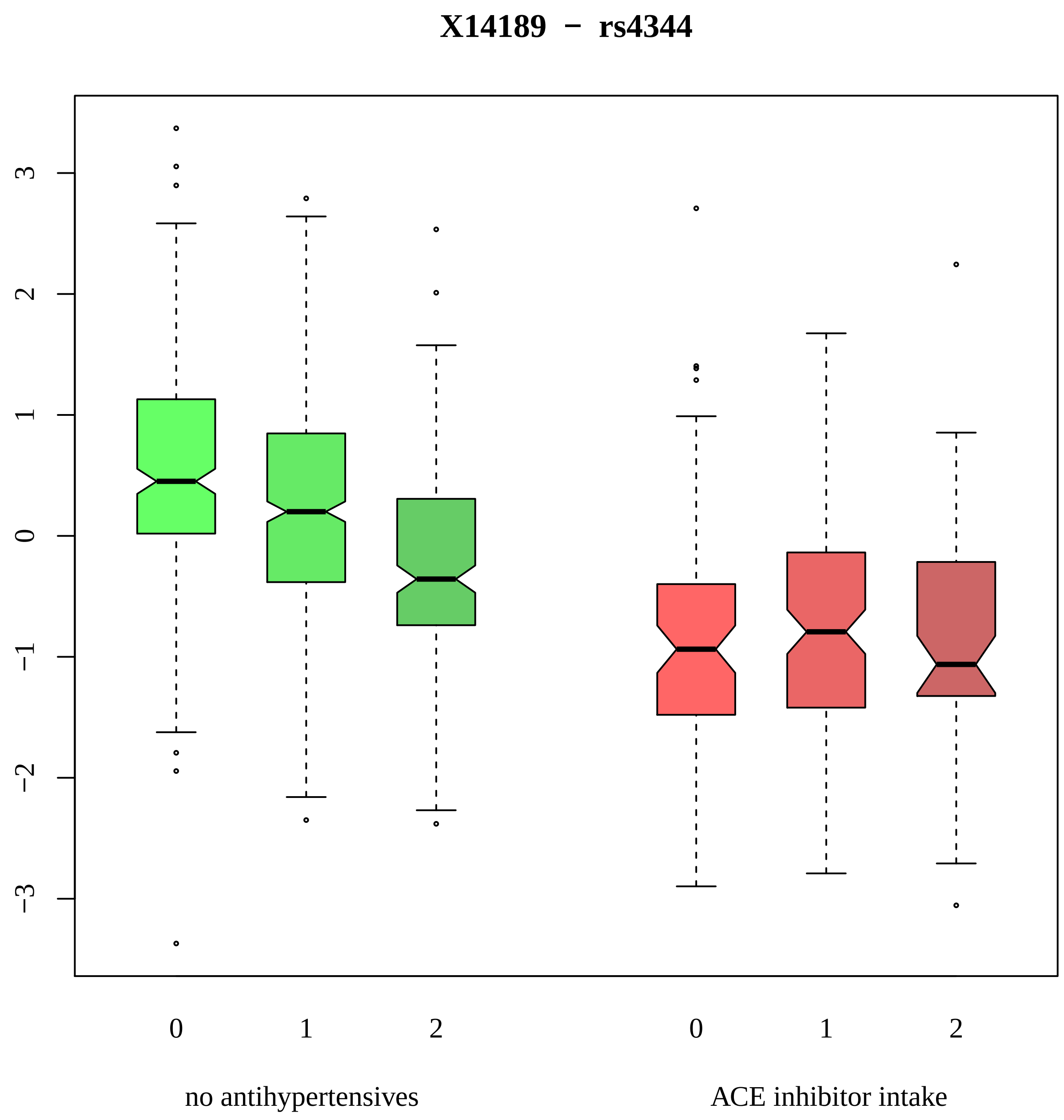

X14189 – rs4344
3
2
1
0
−1
−2
−3
0
1
2
0
1
2
no antihypertensives
ACE inhibitor intake

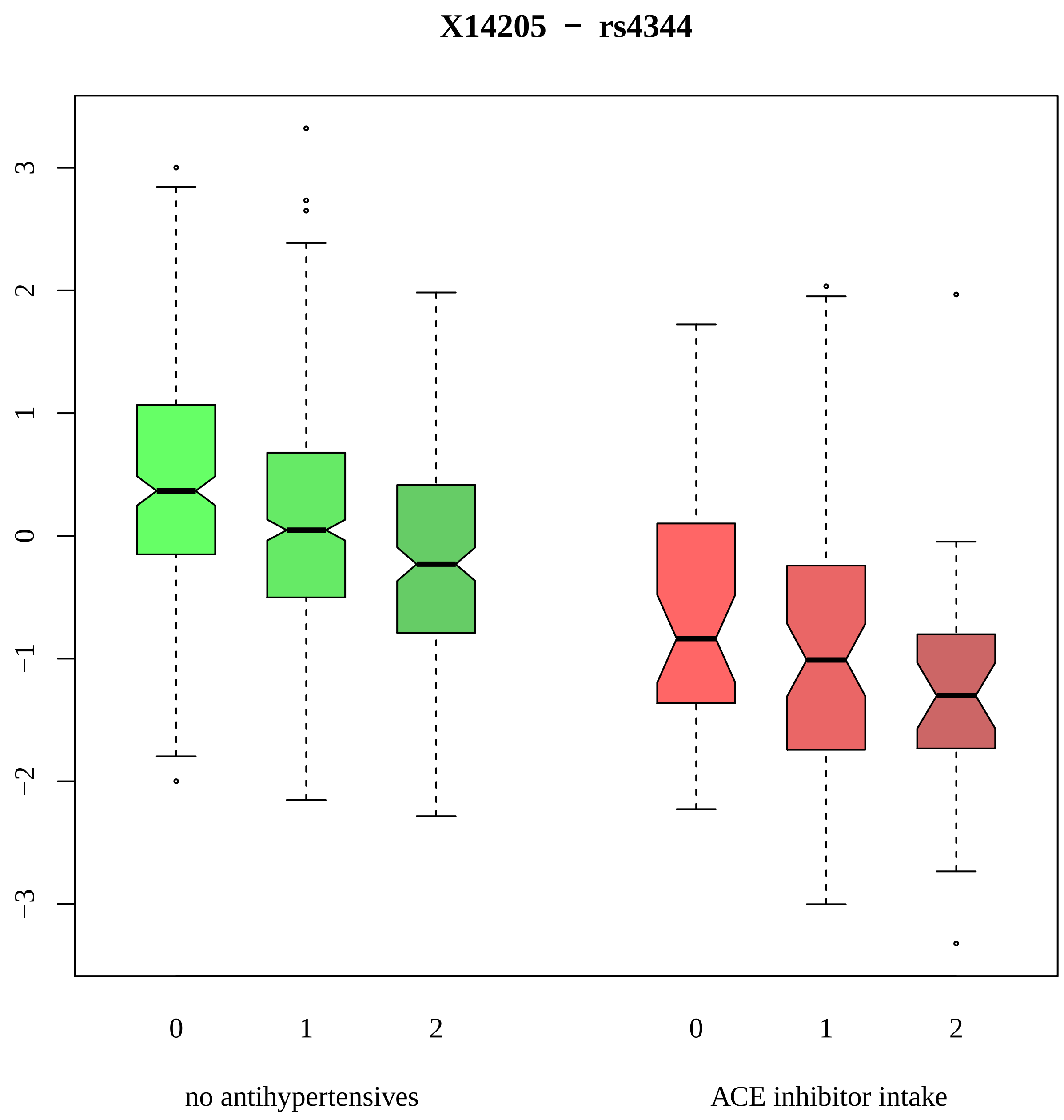

X14205 – rs4344
3
2
1
0
−1
−2
−3
0
1
2
0
1
2
no antihypertensives
ACE inhibitor intake

X14208 − rs4344

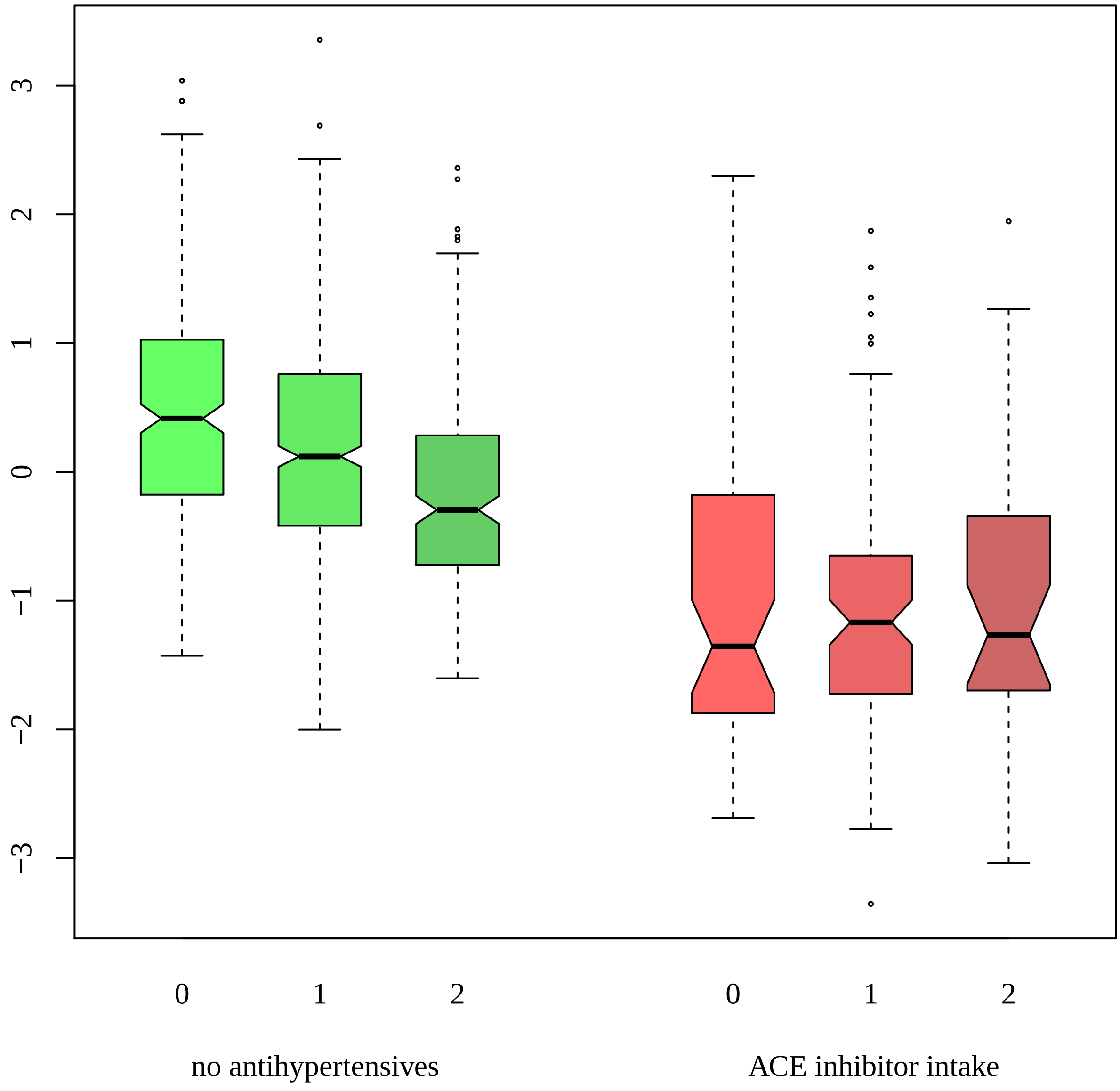

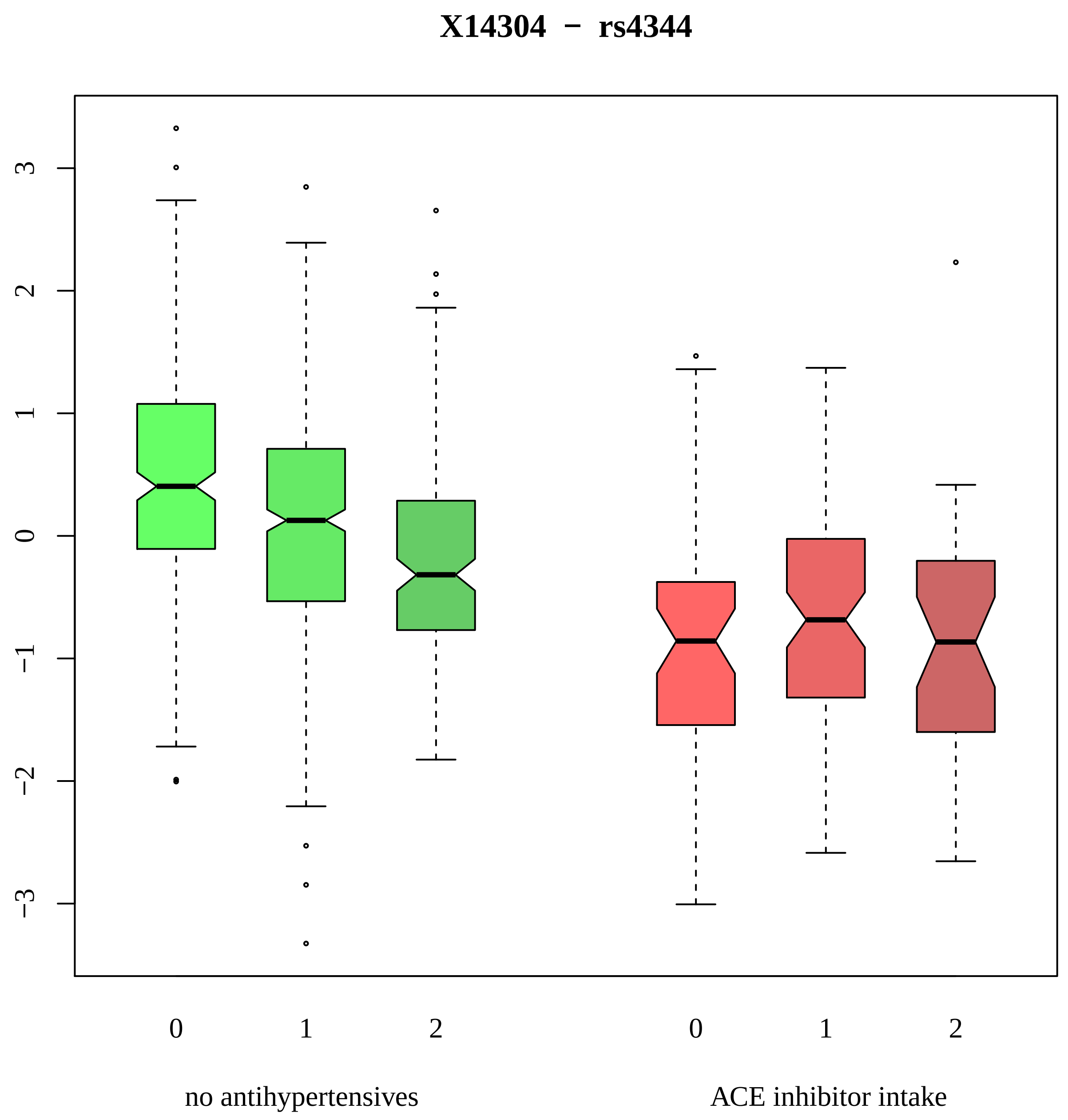
X14304 – rs4344
3
2
1
0
−1
−2
−3
0
1
2
0
1
2
no antihypertensives
ACE inhibitor intake

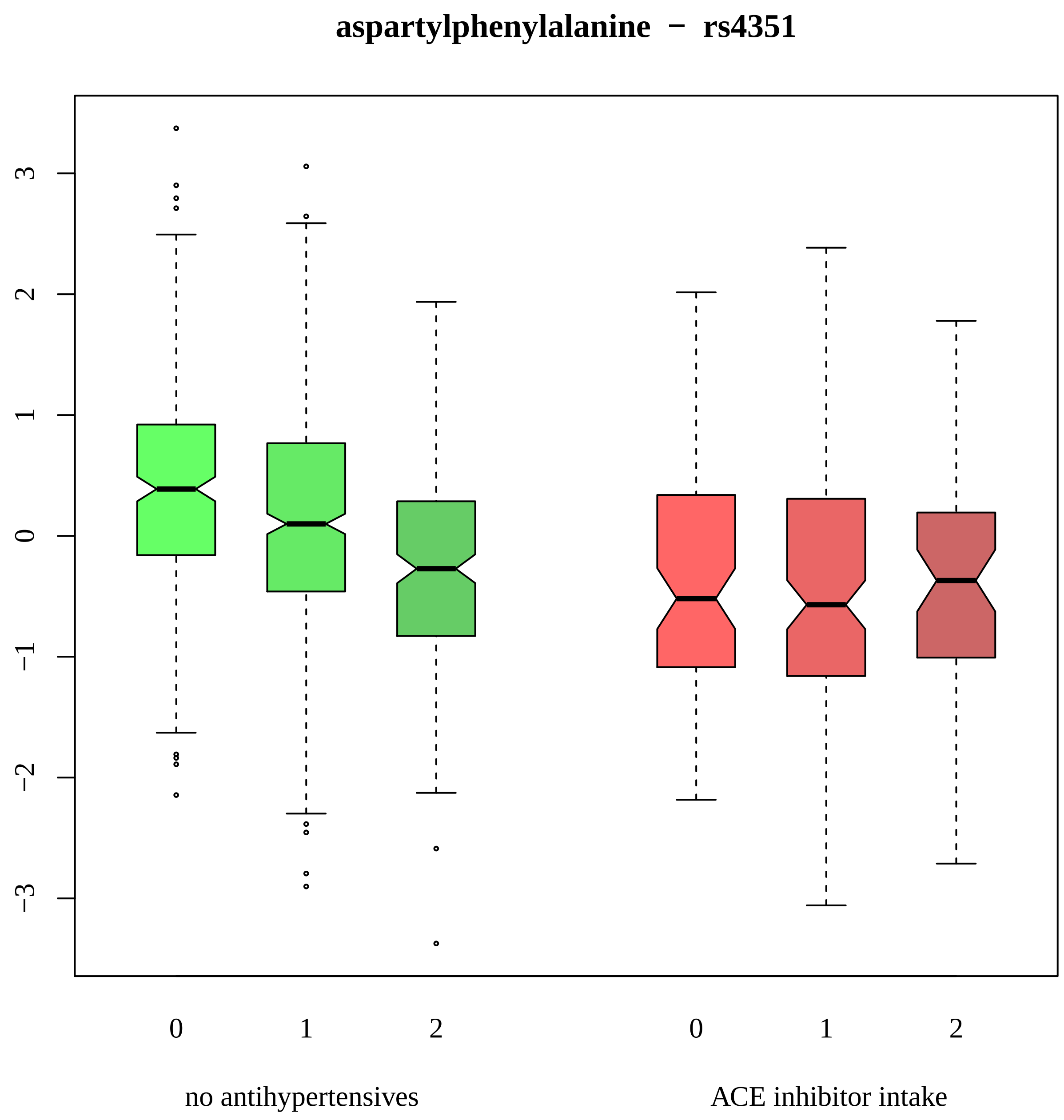

aspartylphenylalanine – rs4351
3
2
1
0
−1
−2
−3
0
1
2
0
1
2
no antihypertensives
ACE inhibitor intake

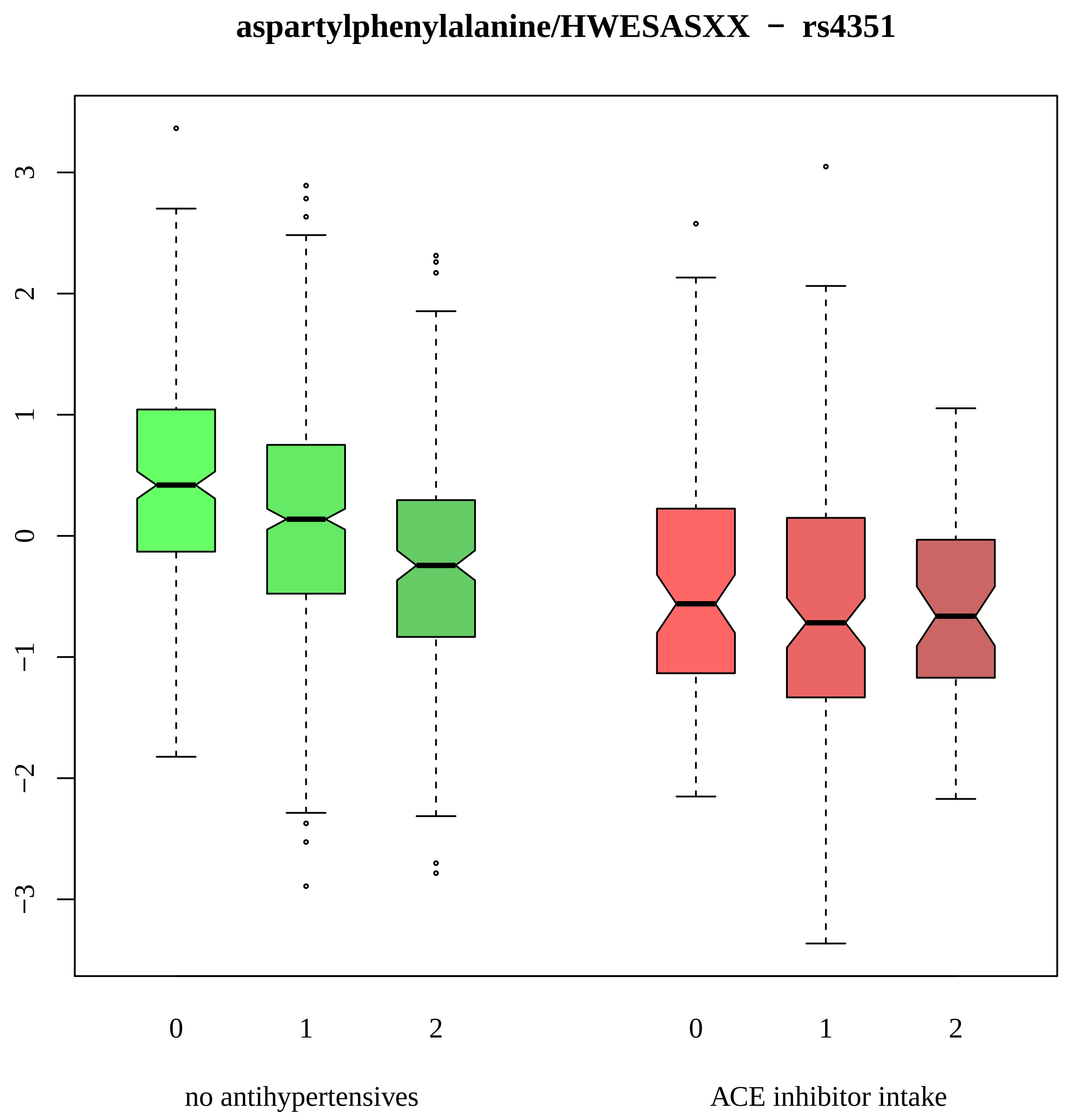
aspartylphenylalanine/HWESASXX – rs4351
3
2
1
0
−1
−2
−3
0
1
2
0
1
2
no antihypertensives
ACE inhibitor intake

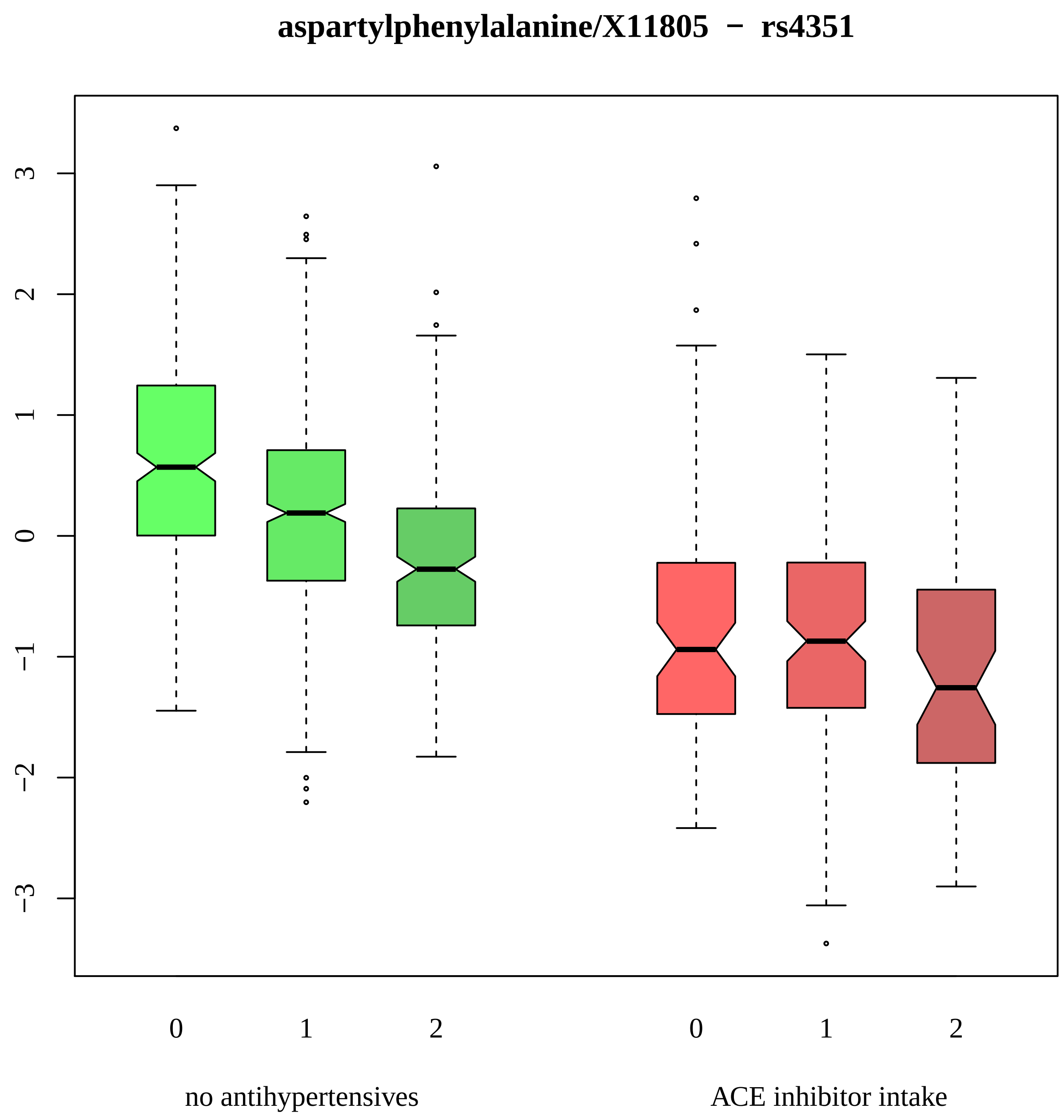

aspartylphenylalanine/X11805 – rs4351
3
2
1
0
−1
−2
−3
0
1
2
0
1
2
no antihypertensives
ACE inhibitor intake

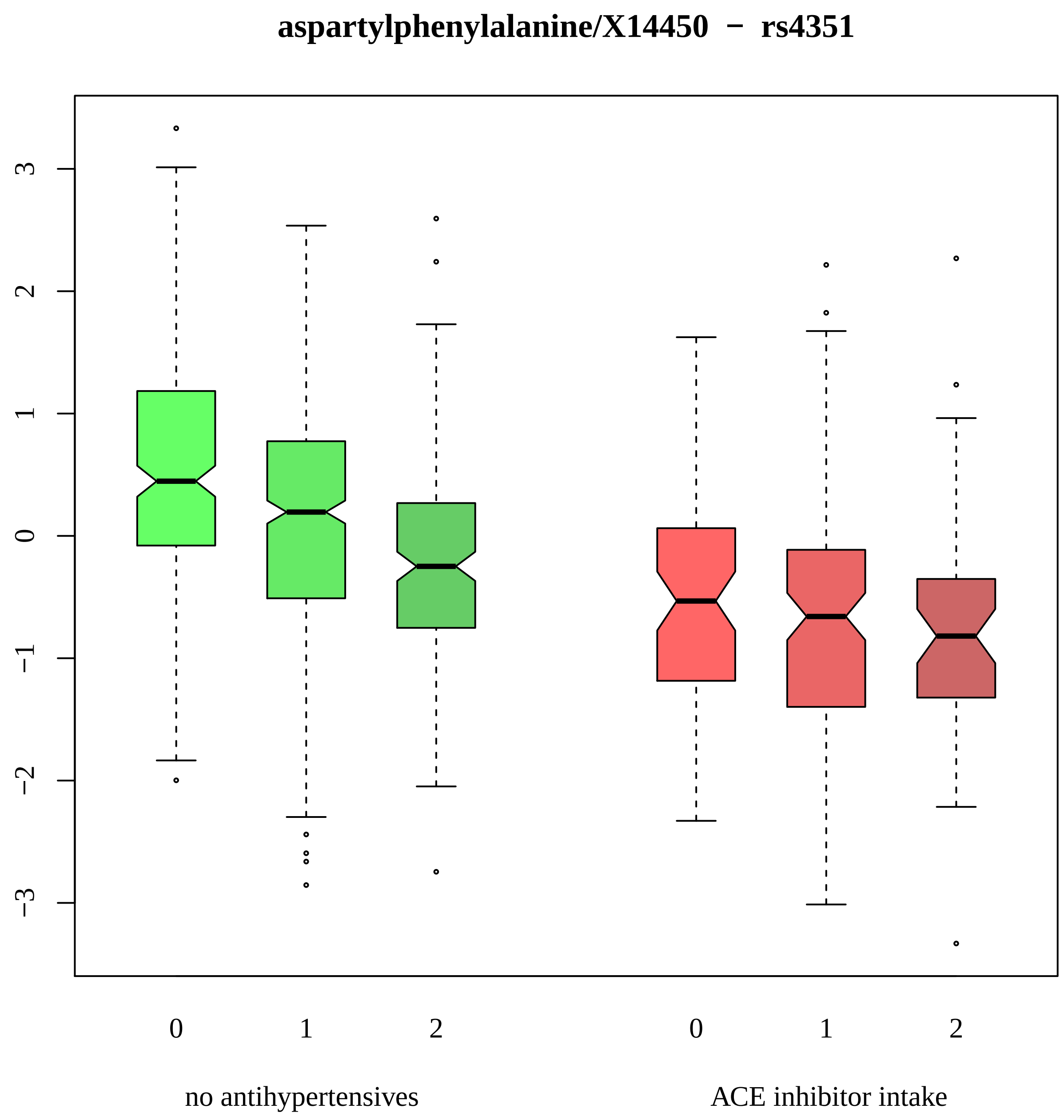
aspartylphenylalanine/X14450 – rs4351
3
2
1
0
−1
−2
−3
0
1
2
0
1
2
no antihypertensives
ACE inhibitor intake

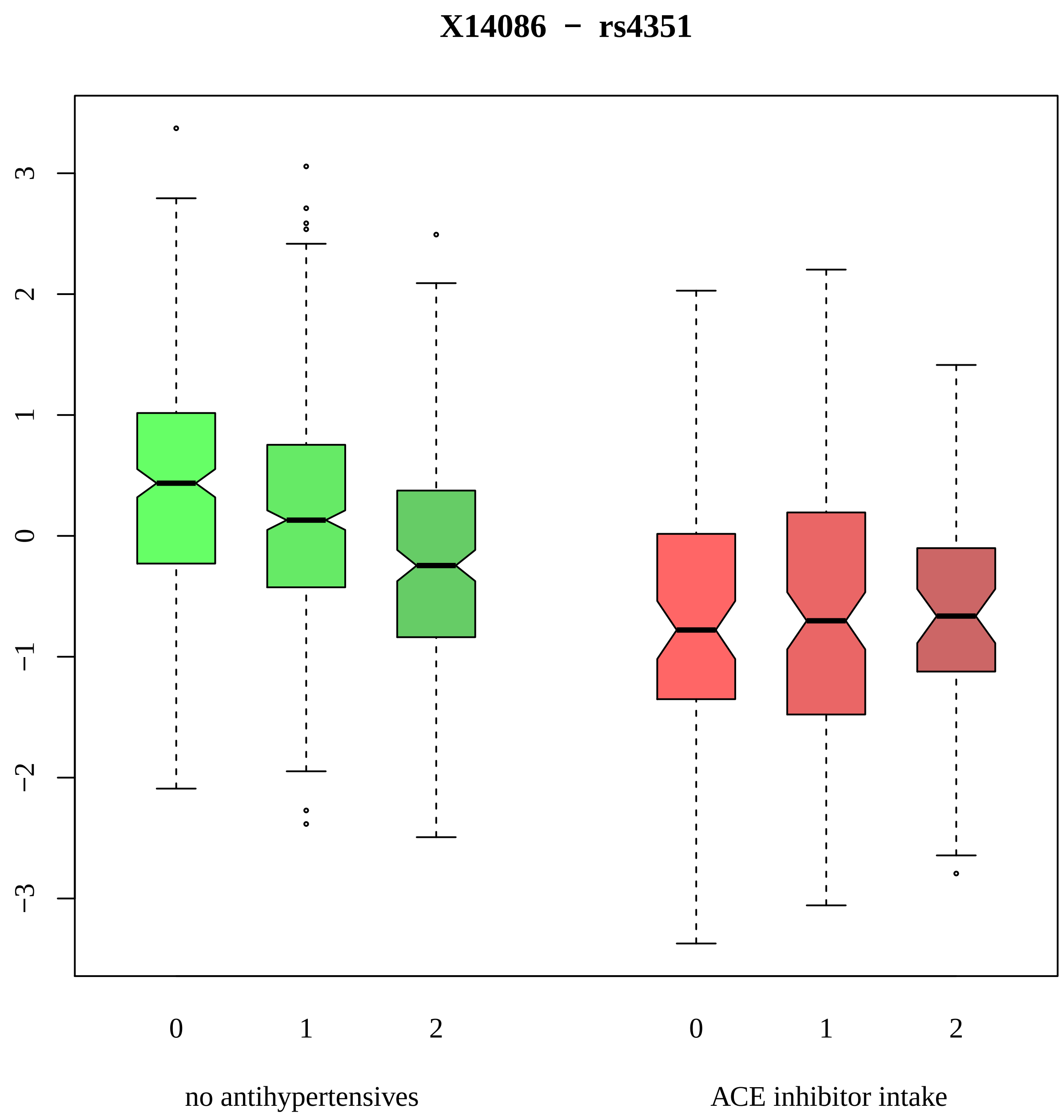
X14086 – rs4351
3
2
1
0
−1
−2
−3
0
1
2
0
1
2
no antihypertensives
ACE inhibitor intake

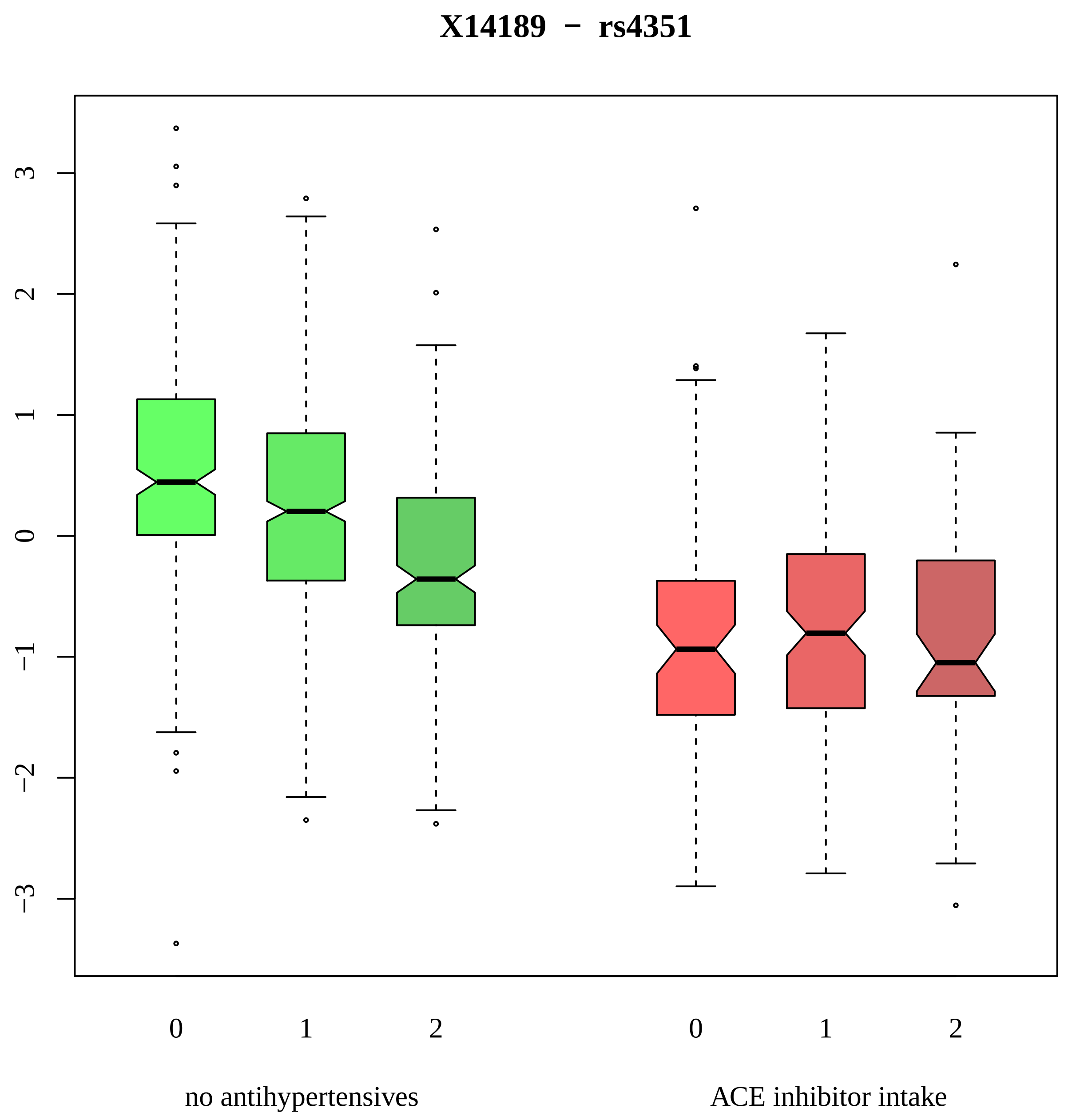

X14189 – rs4351
3
2
1
0
−1
−2
−3
0
1
2
0
1
2
no antihypertensives
ACE inhibitor intake

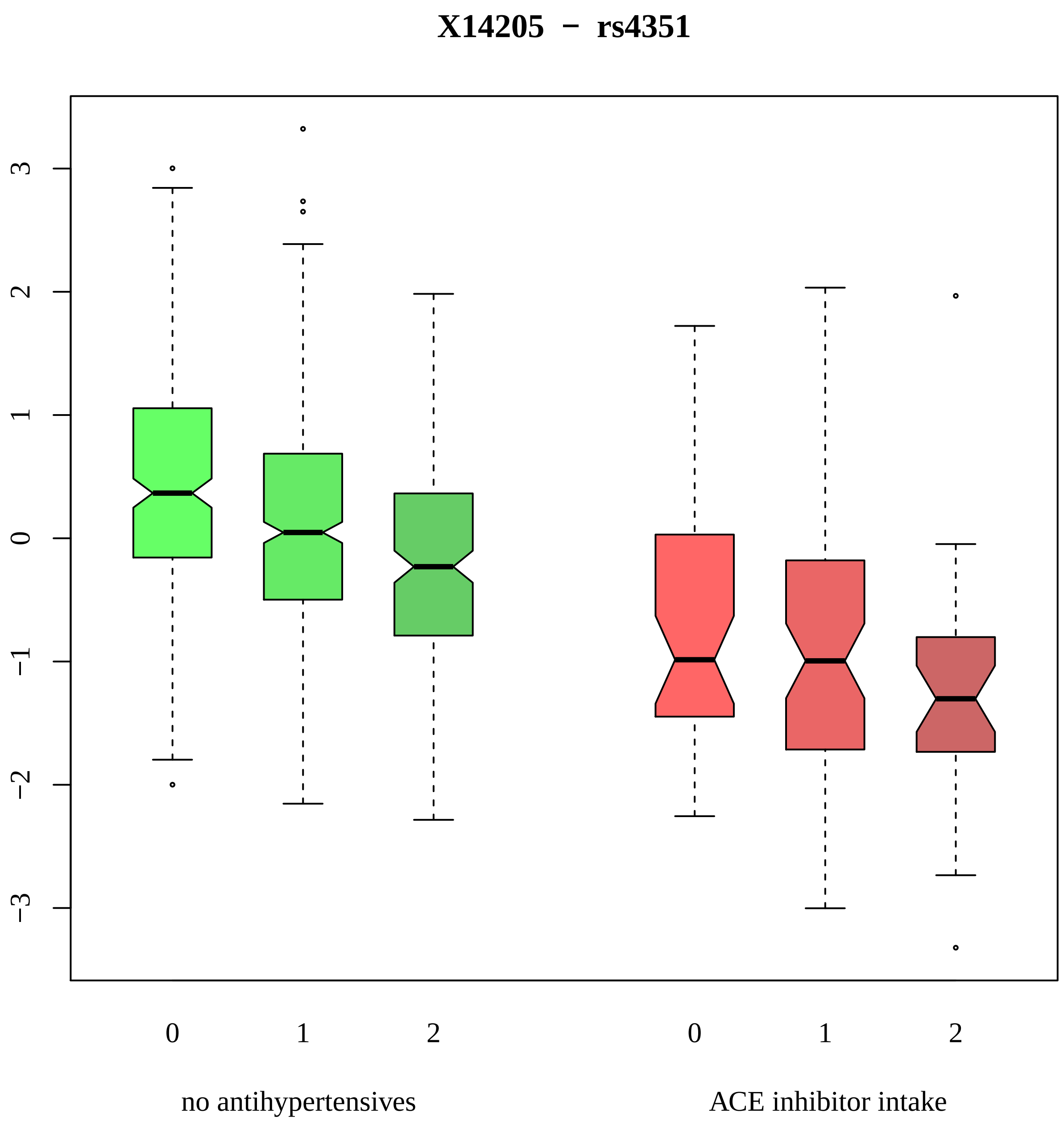

X14205 – rs4351
3
2
1
0
−1
−2
−3
0
1
2
0
1
2
no antihypertensives
ACE inhibitor intake

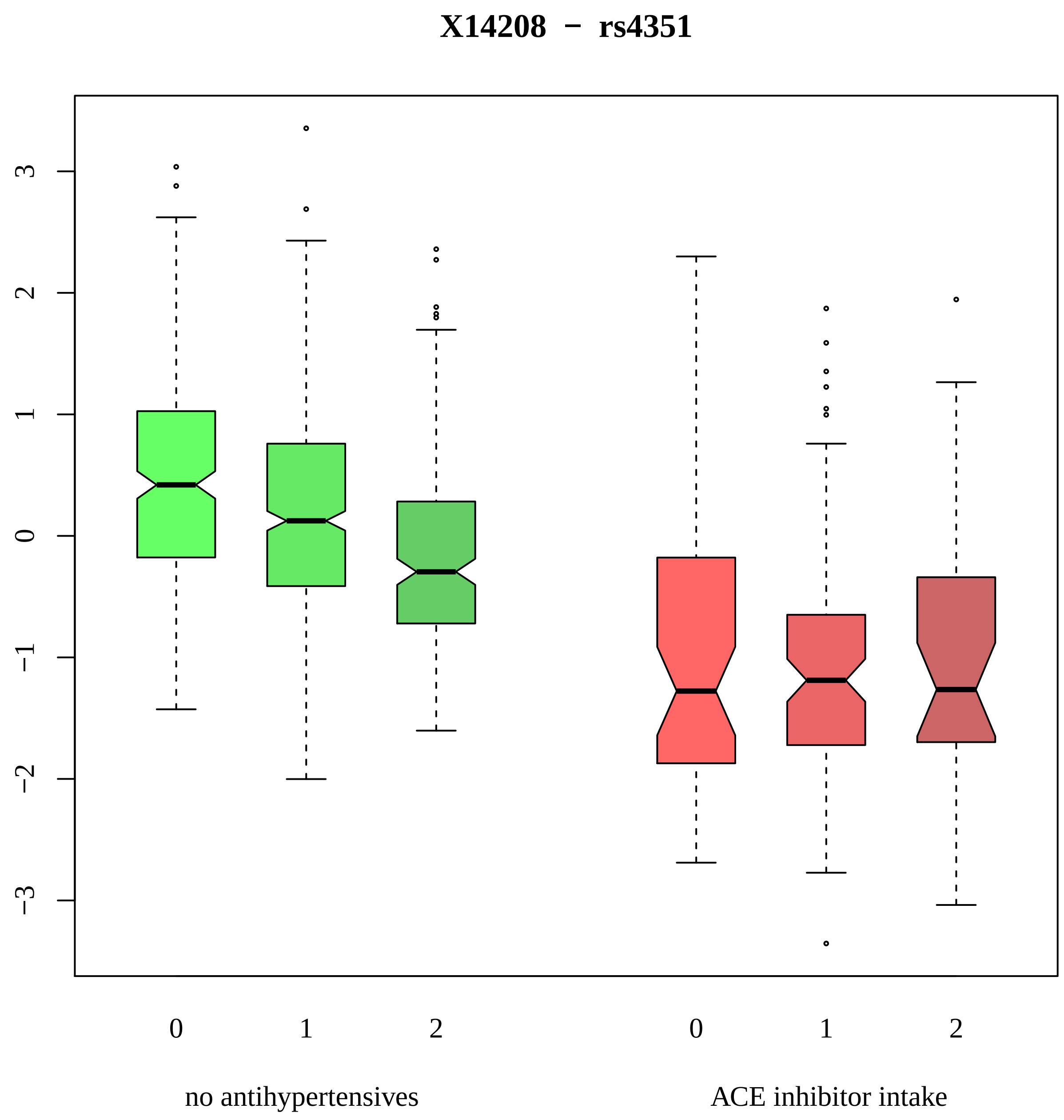

X14208 – rs4351
3
2
1
0
−1
−2
−3
0
1
2
0
1
2
no antihypertensives
ACE inhibitor intake

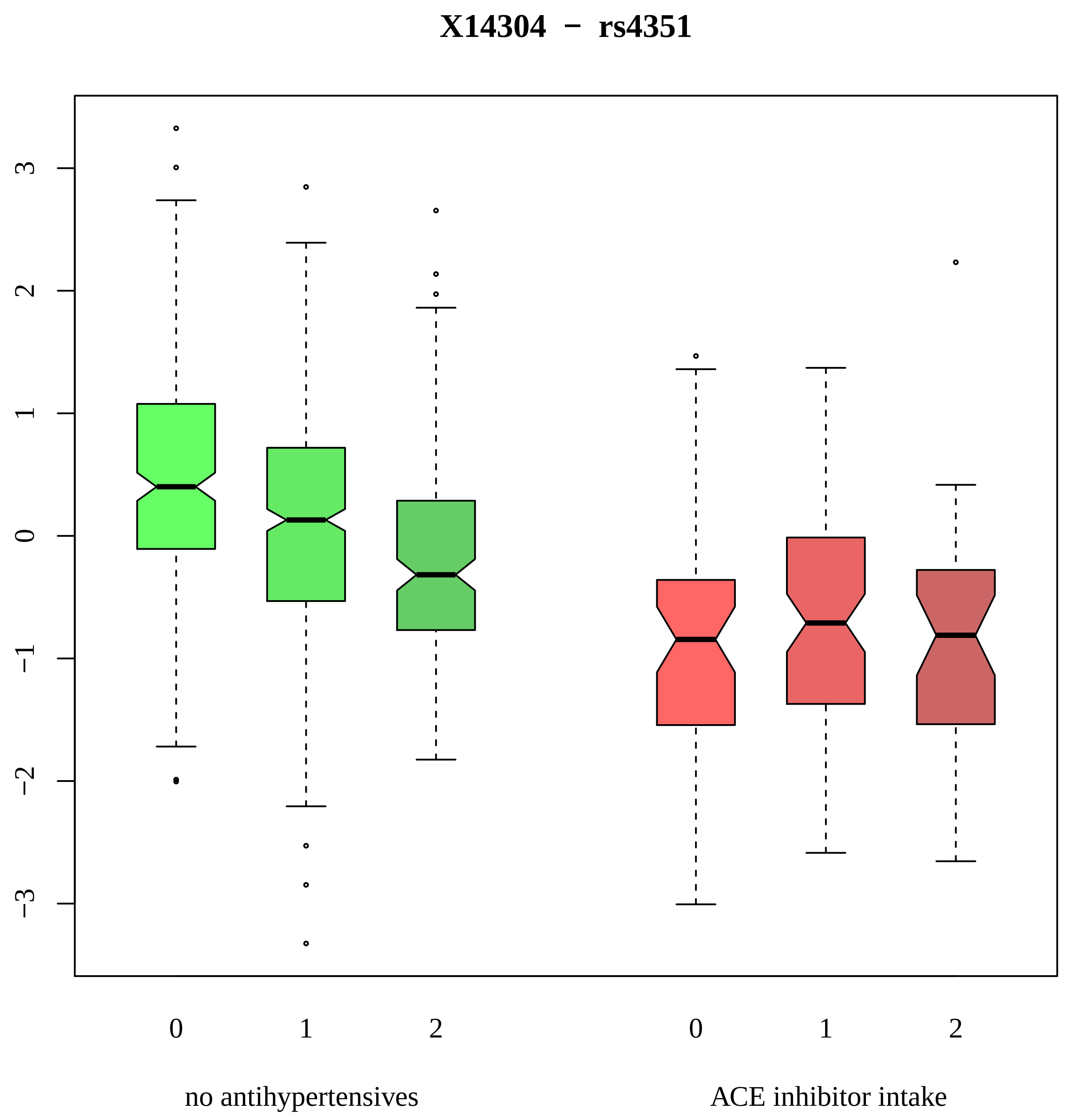
X14304 – rs4351
3
2
1
0
−1
−2
−3
0
1
2
0
1
2
no antihypertensives
ACE inhibitor intake

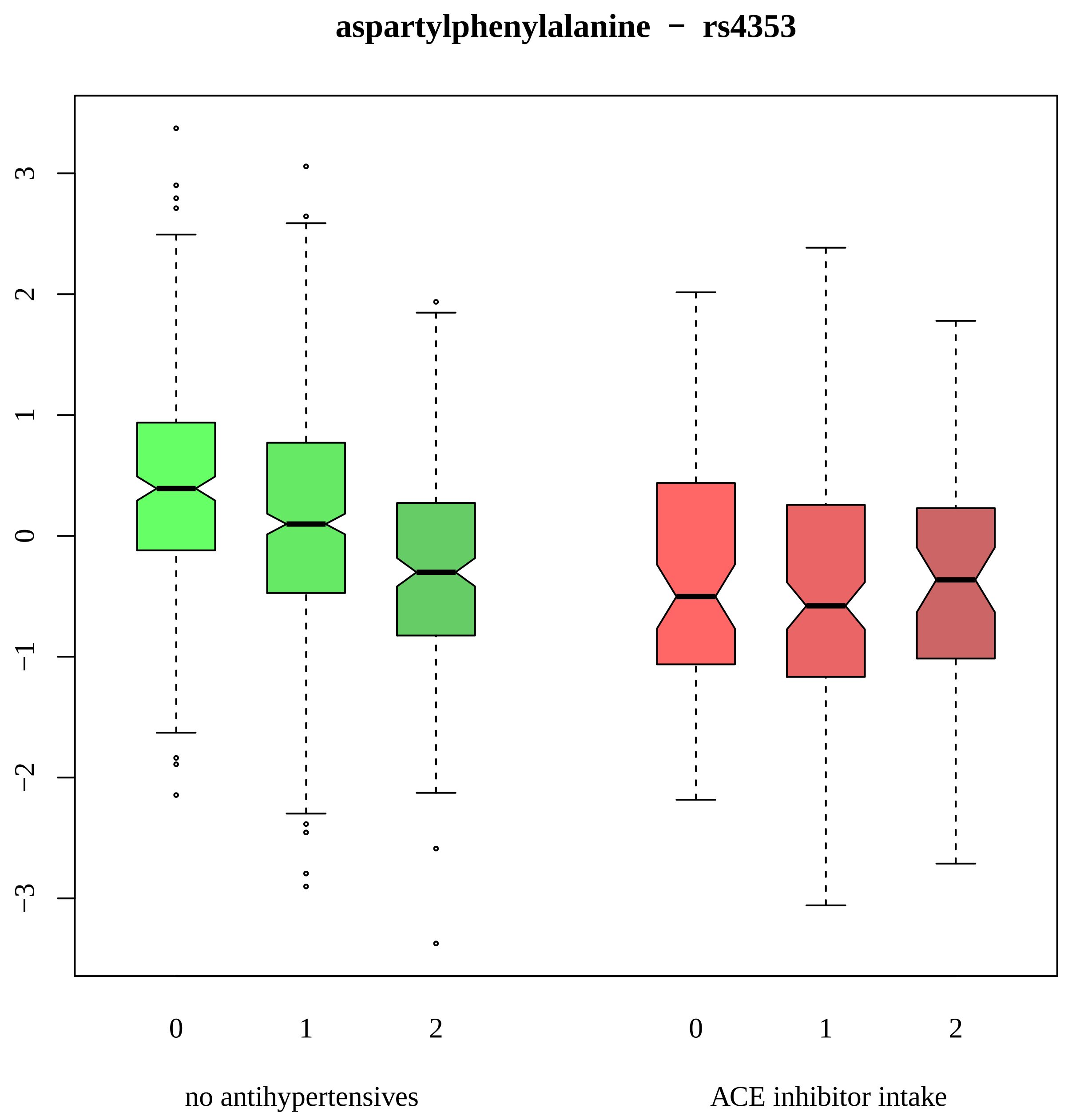
aspartylphenylalanine − rs4353
3
2
1
0
−1
−2
−3
0
1
2
0
1
2
no antihypertensives
ACE inhibitor intake

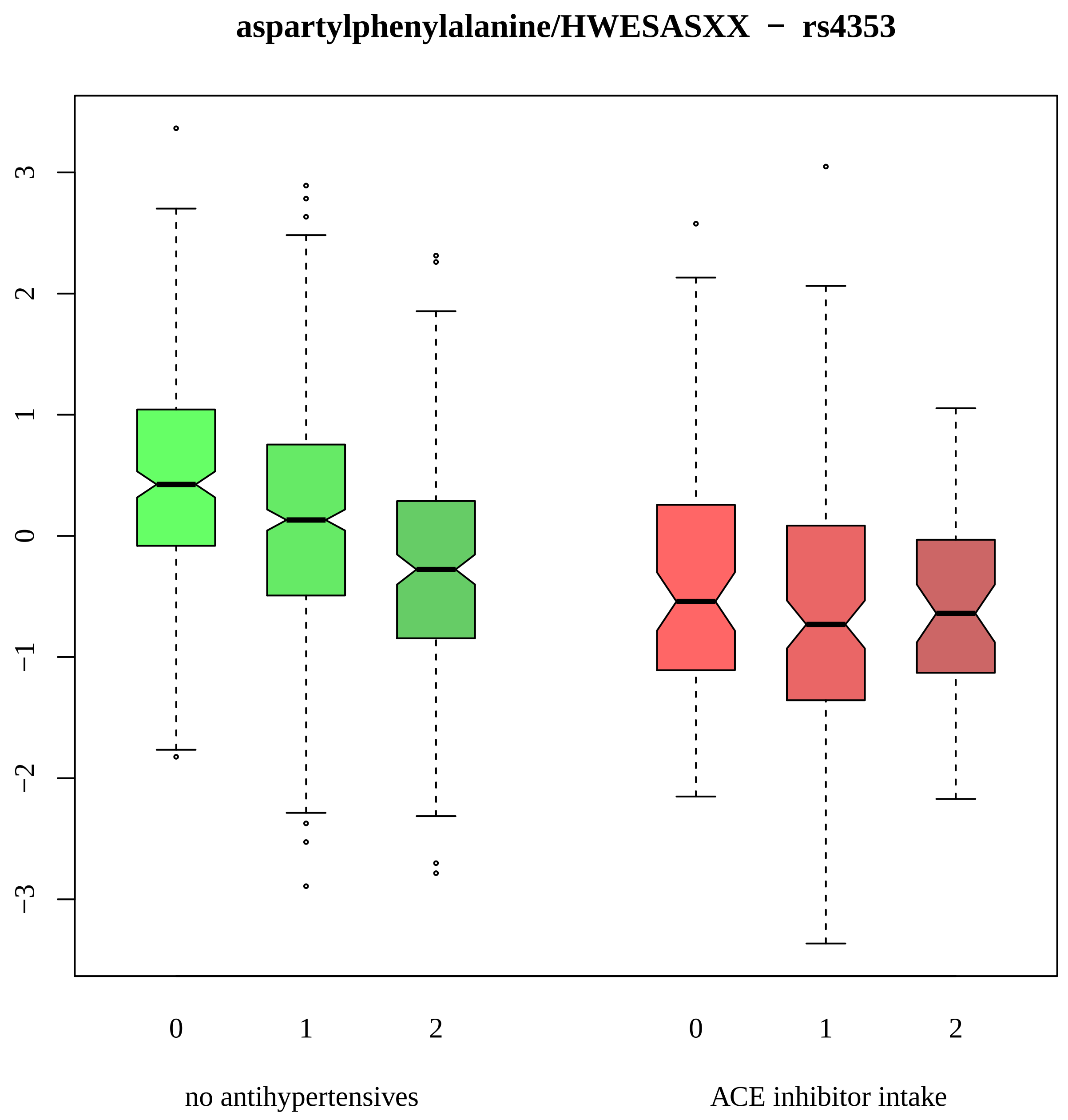
aspartylphenylalanine/HWESASXX – rs4353
3
2
1
0
−1
−2
−3
0
1
2
0
1
2
no antihypertensives
ACE inhibitor intake

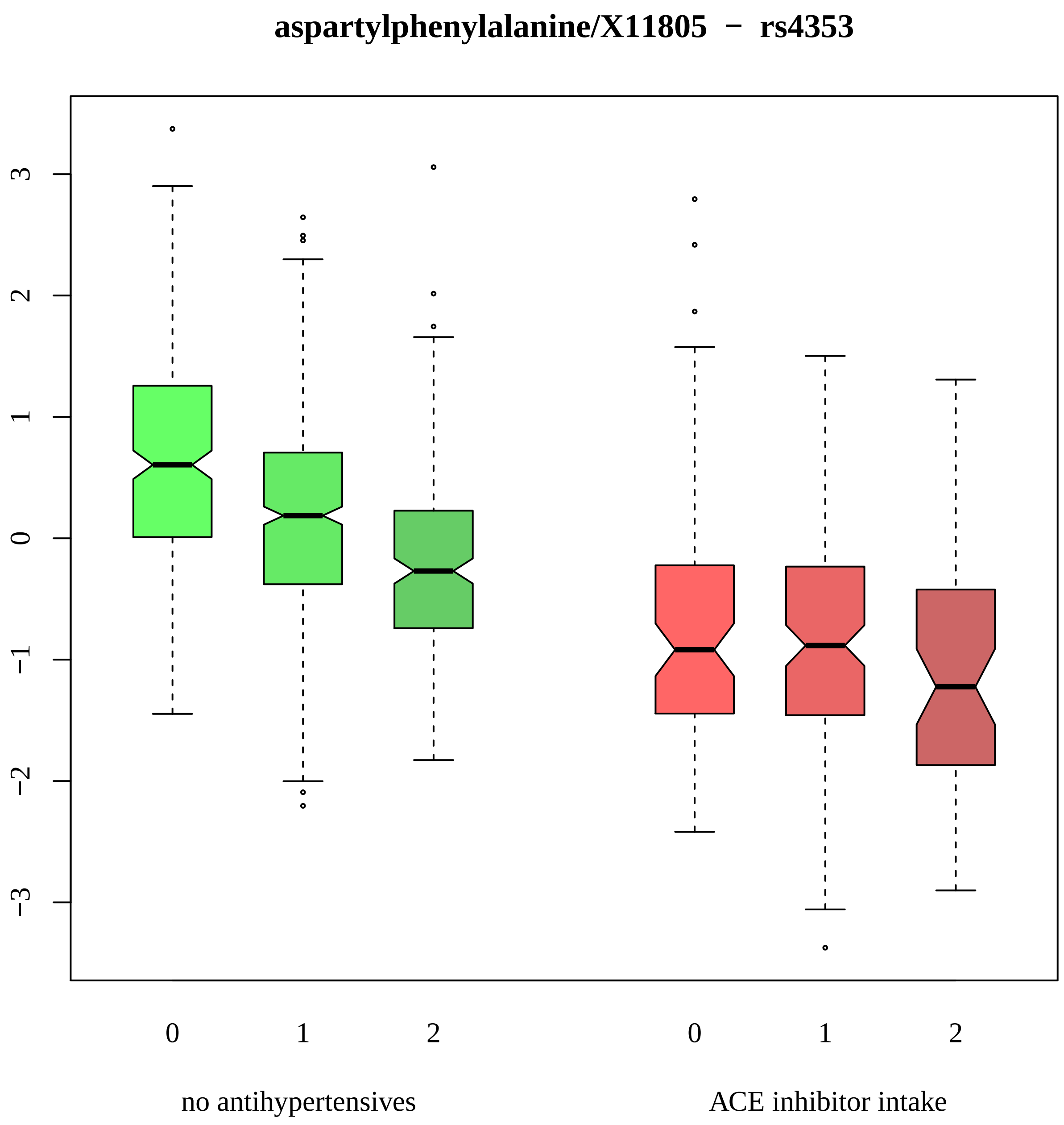
aspartylphenylalanine/X11805 – rs4353
3
2
1
0
−1
−2
−3
0
1
2
0
1
2
no antihypertensives
ACE inhibitor intake

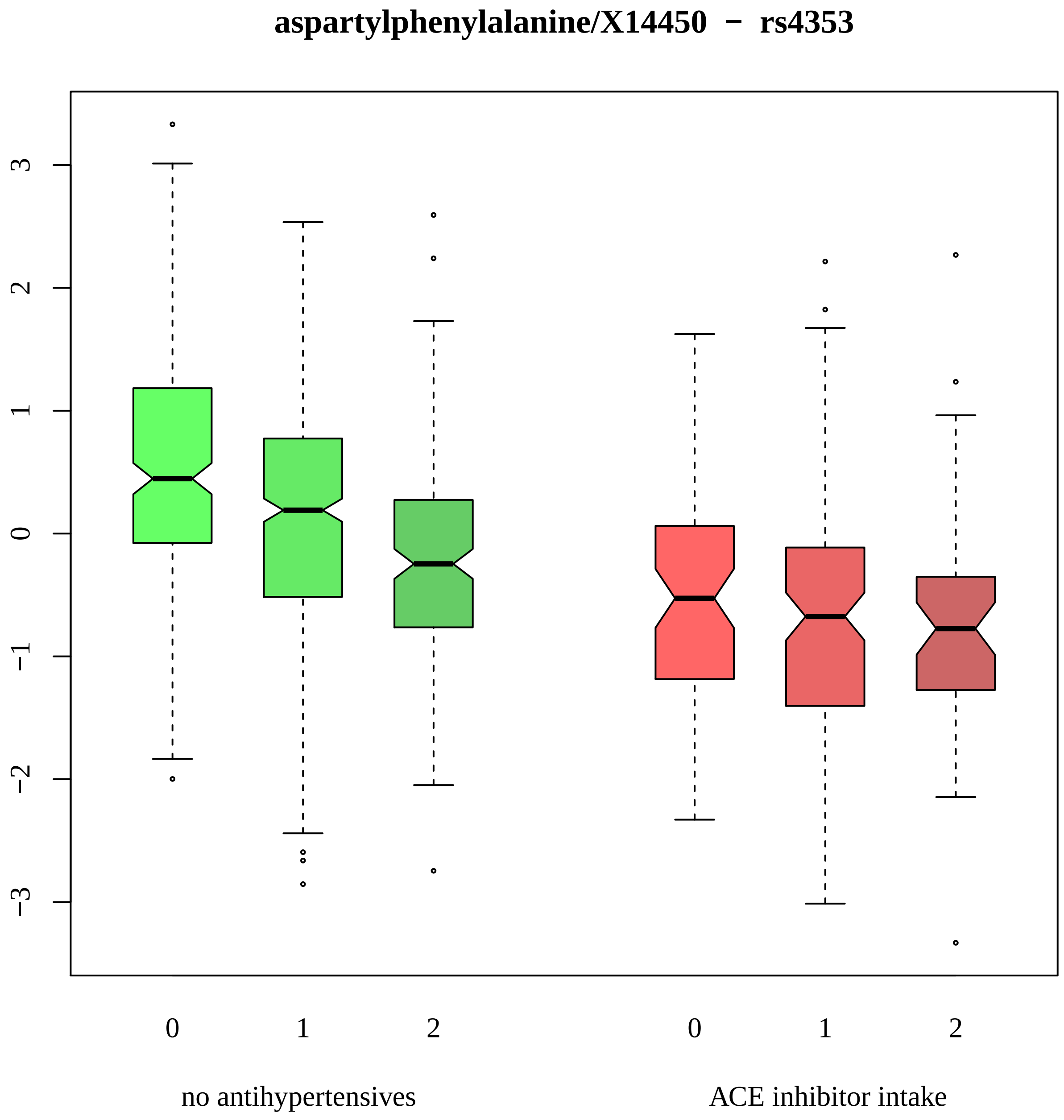
aspartylphenylalanine/X14450 − rs4353
3
2
1
0
−1
−2
−3
0
1
2
0
1
2
no antihypertensives
ACE inhibitor intake

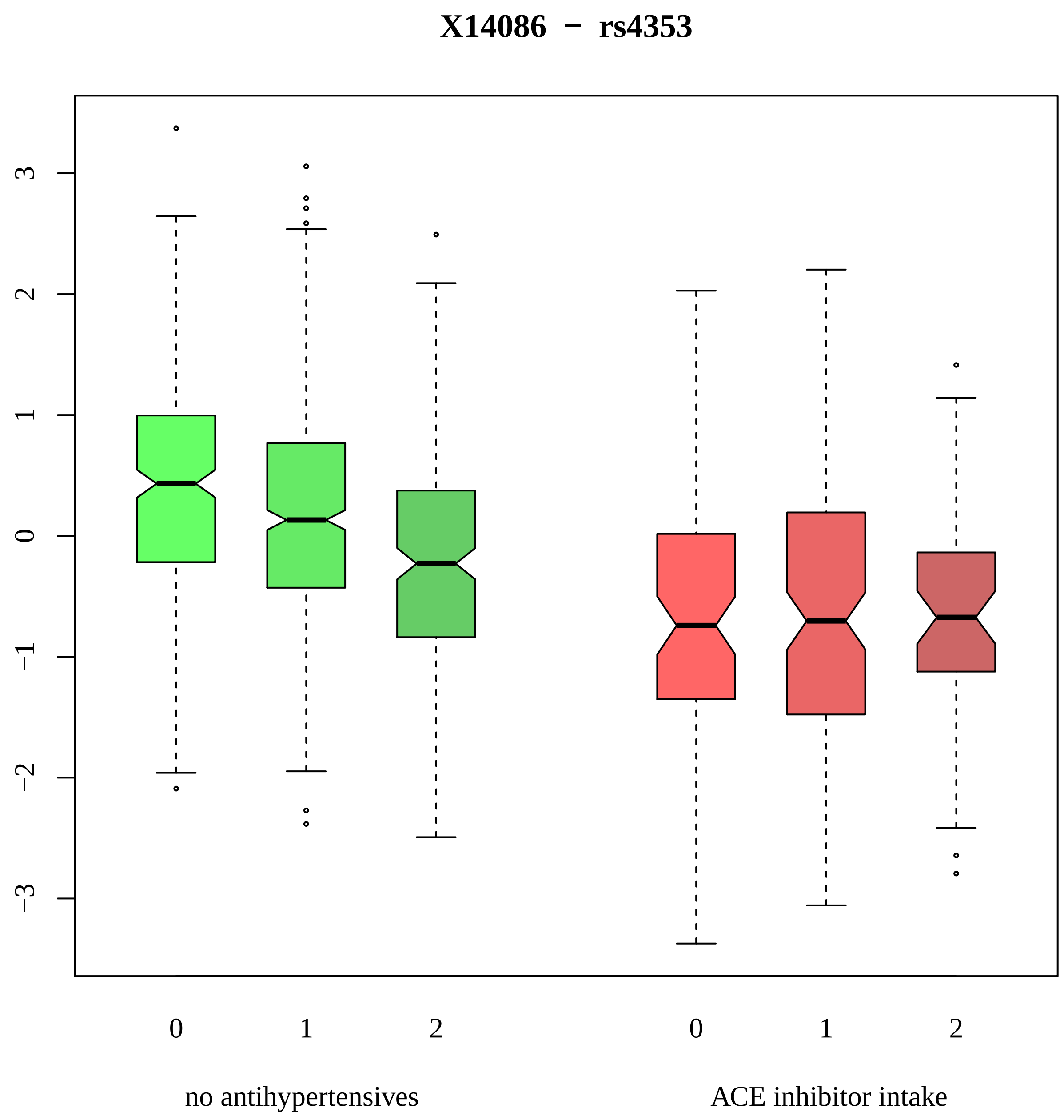

X14086 – rs4353
3
2
1
0
−1
−2
−3
0
1
2
0
1
2
no antihypertensives
ACE inhibitor intake

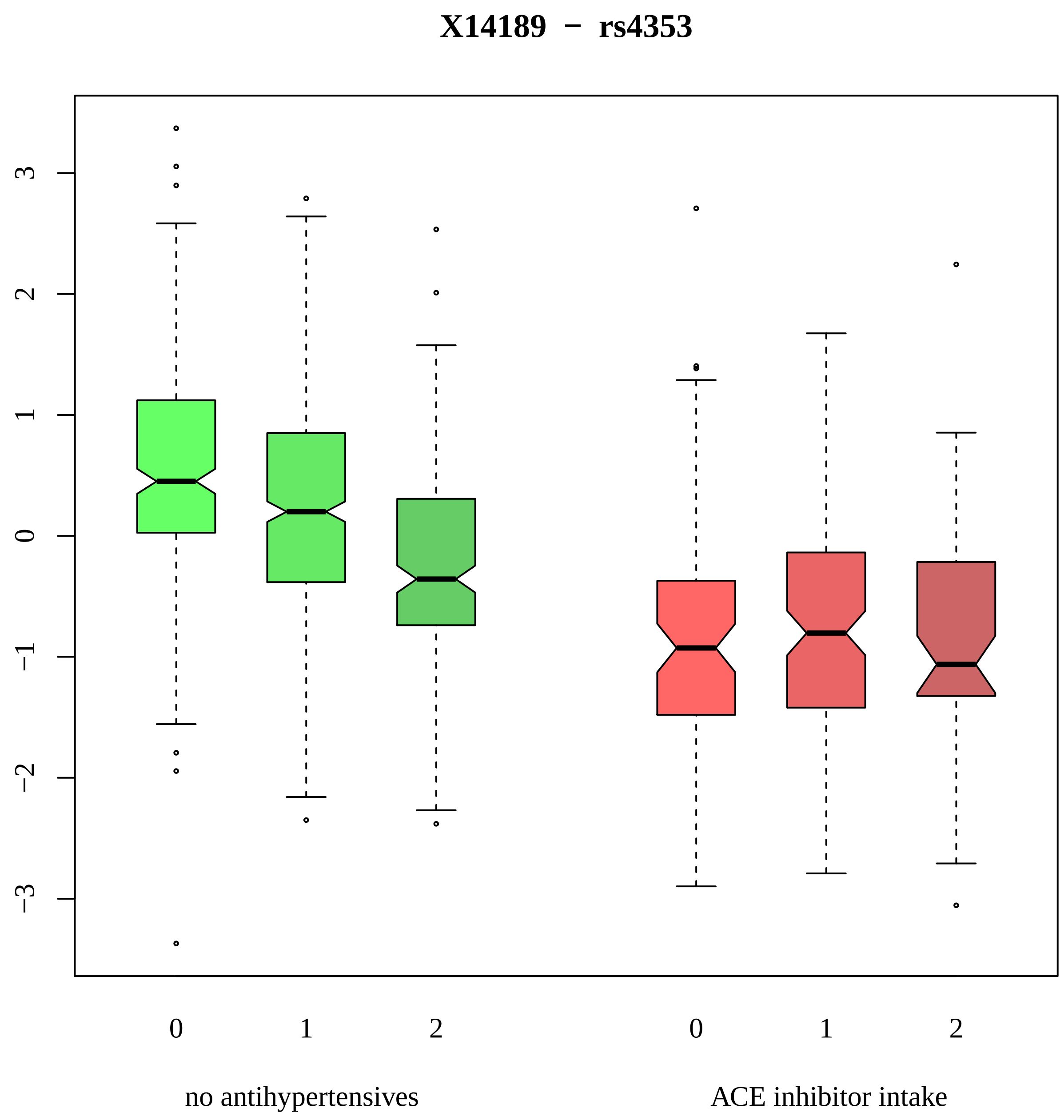
X14189 – rs4353
3
2
1
0
−1
−2
−3
0
1
2
0
1
2
no antihypertensives
ACE inhibitor intake

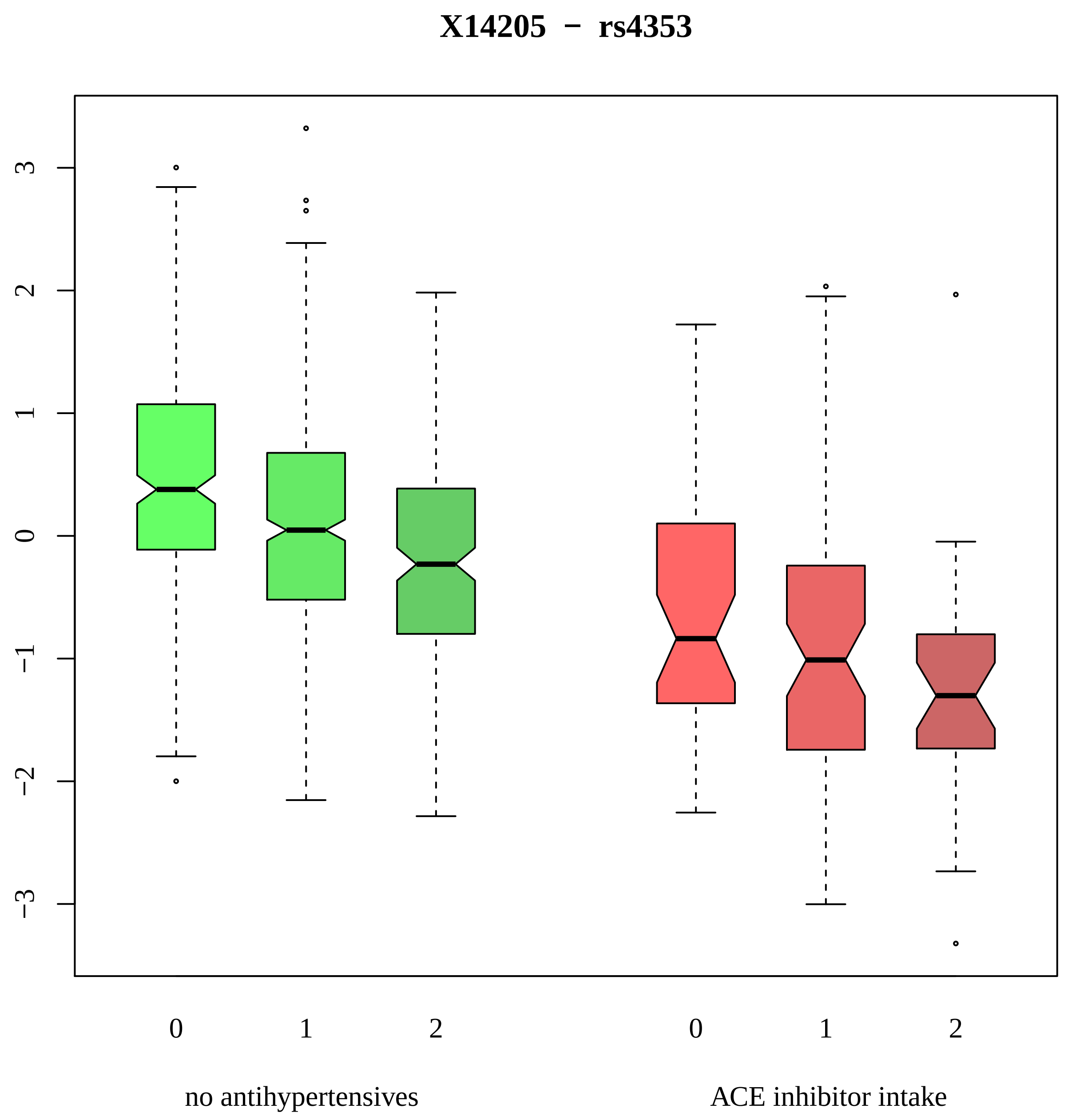
X14205 – rs4353
3
2
1
0
−1
−2
−3
0
1
2
0
1
2
no antihypertensives
ACE inhibitor intake

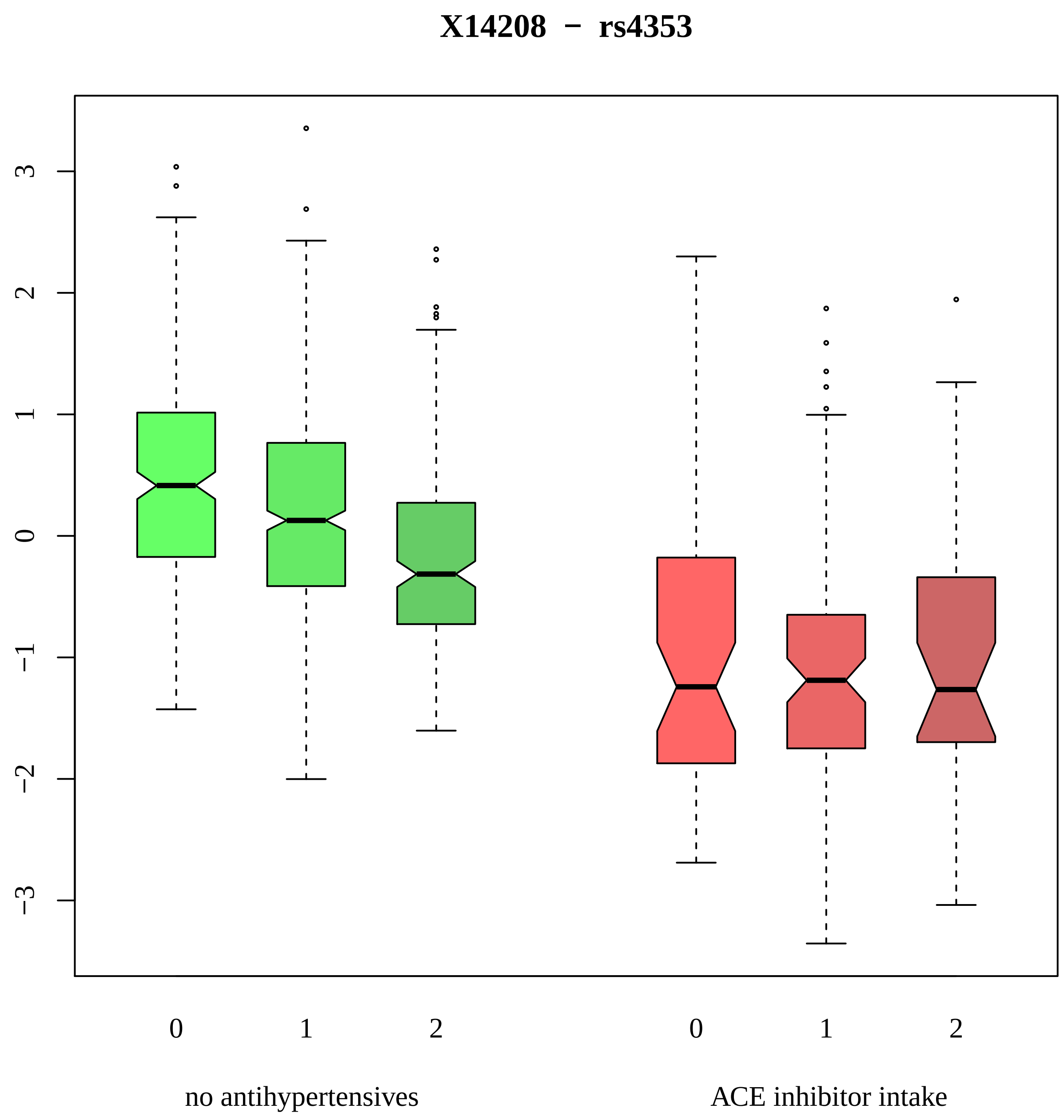
X14208 – rs4353
3
2
1
0
−1
−2
−3
0
1
2
0
1
2
no antihypertensives
ACE inhibitor intake

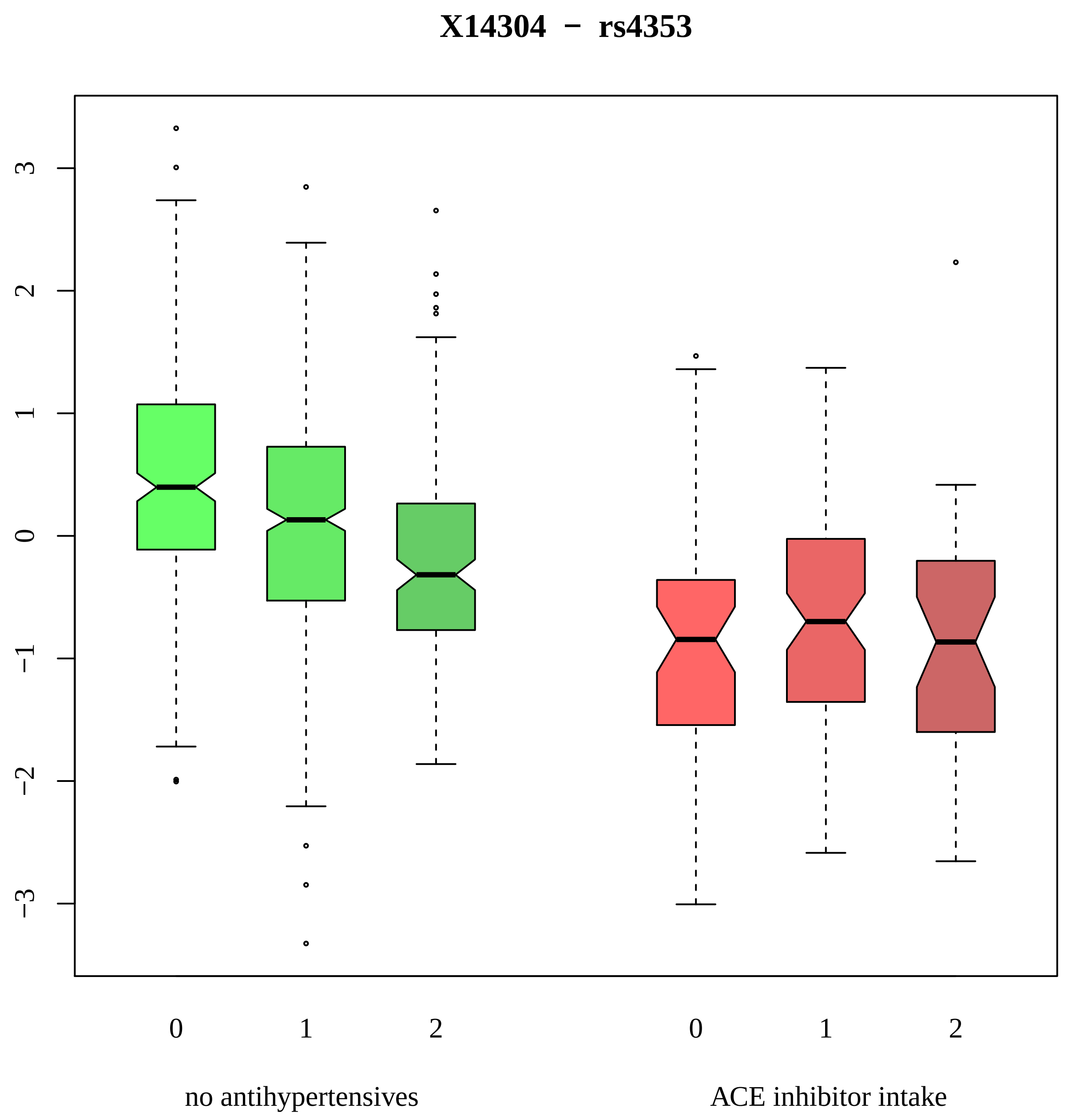

X14304 – rs4353
3
2
1
0
−1
−2
−3
0
1
2
0
1
2
no antihypertensives
ACE inhibitor intake

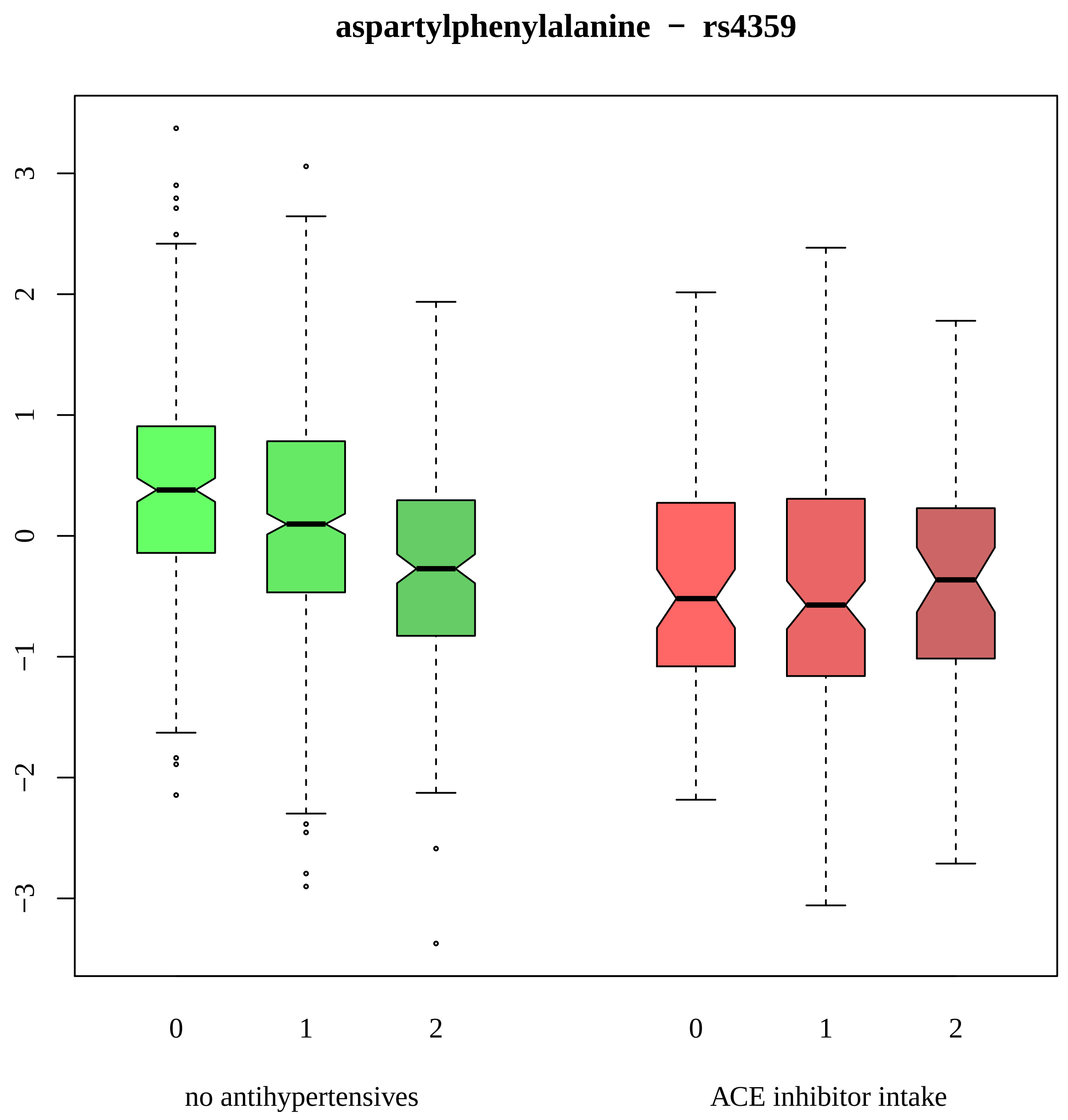

aspartylphenylalanine − rs4359
3
2
1
0
−1
−2
−3
0
1
2
0
1
2
no antihypertensives
ACE inhibitor intake

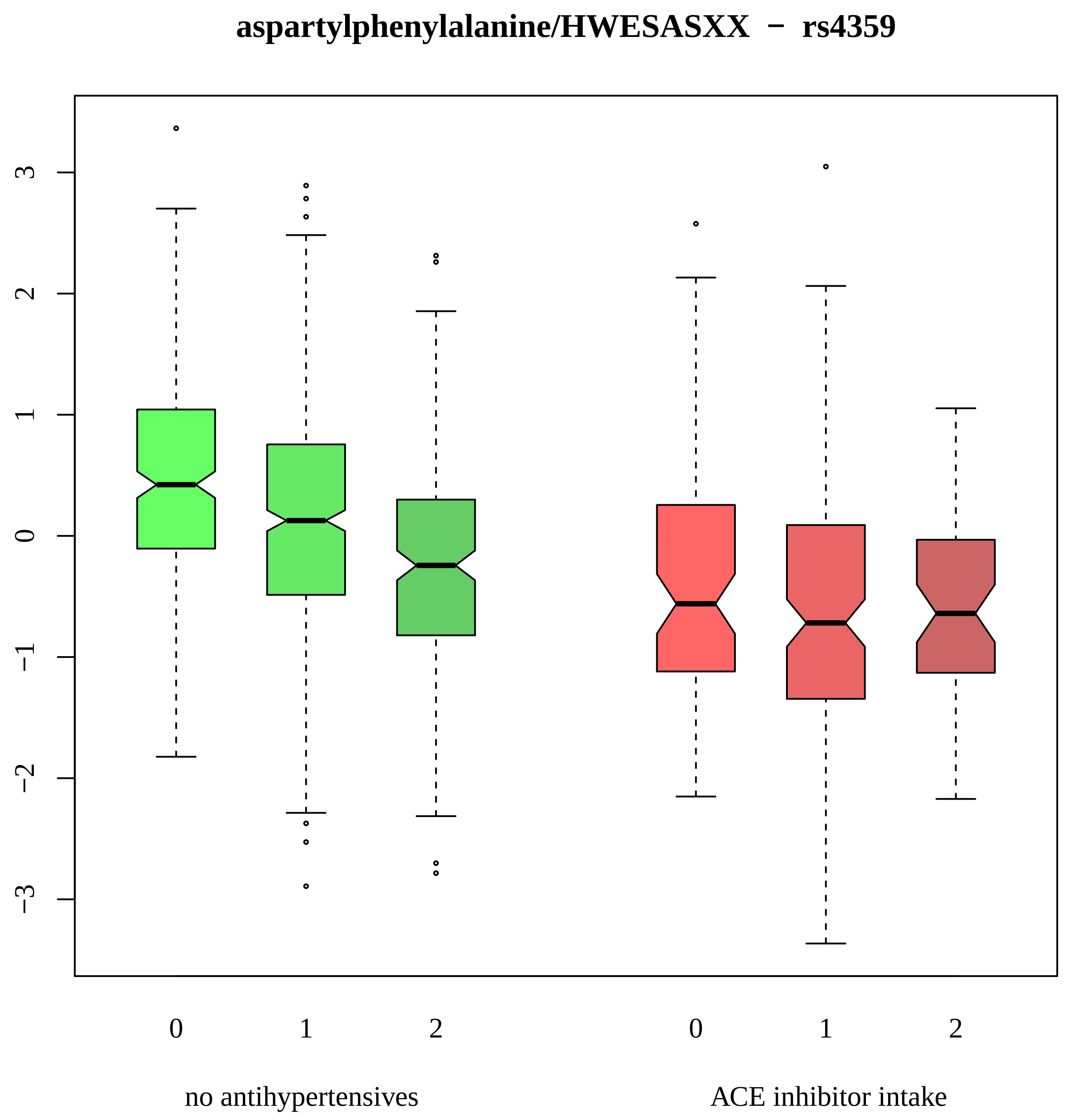
aspartylphenylalanine/HWESASXX – rs4359
3
2
1
0
−1
−2
−3
0
1
2
0
1
2
no antihypertensives
ACE inhibitor intake

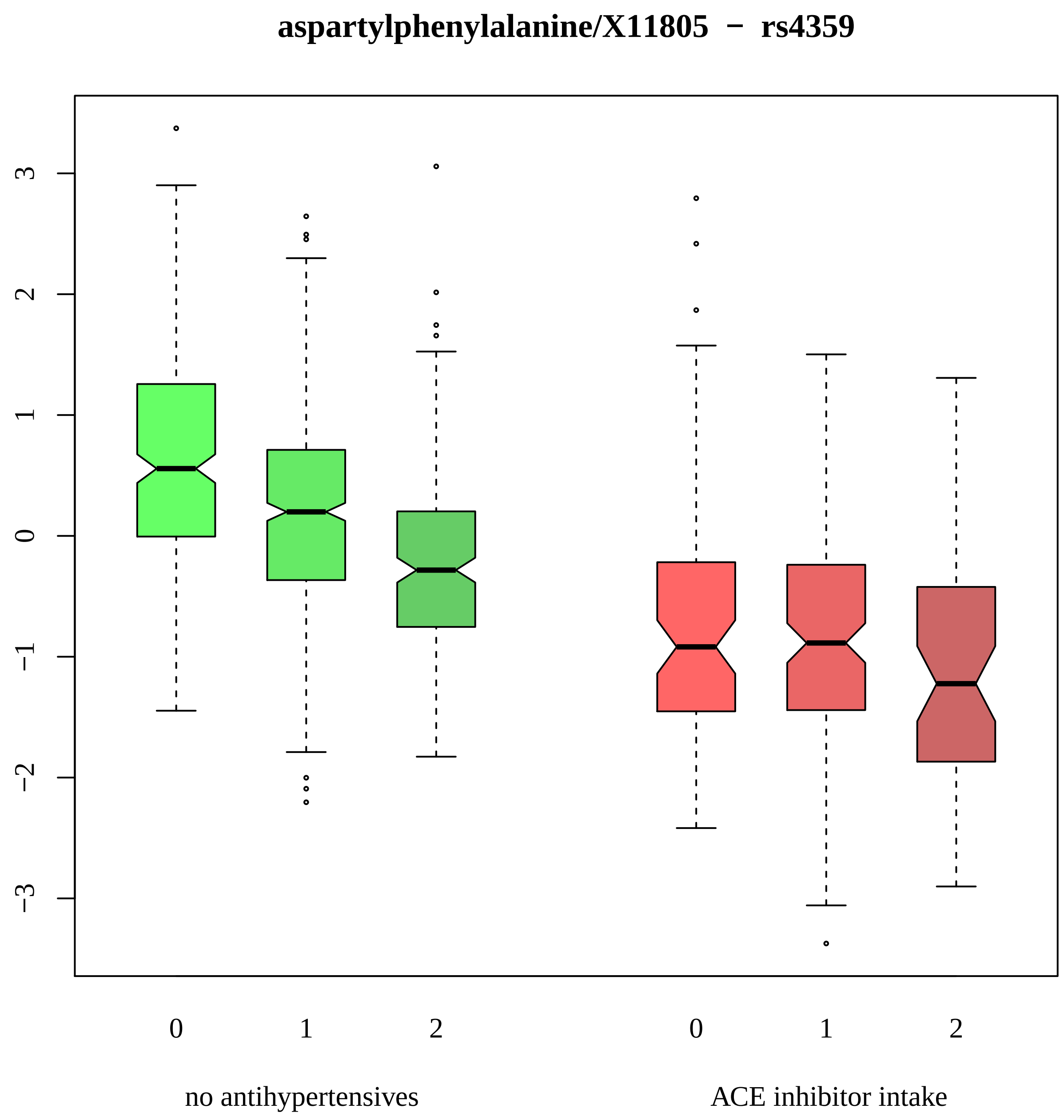
aspartylphenylalanine/X11805 – rs4359
3
2
1
0
−1
−2
−3
0
1
2
0
1
2
no antihypertensives
ACE inhibitor intake

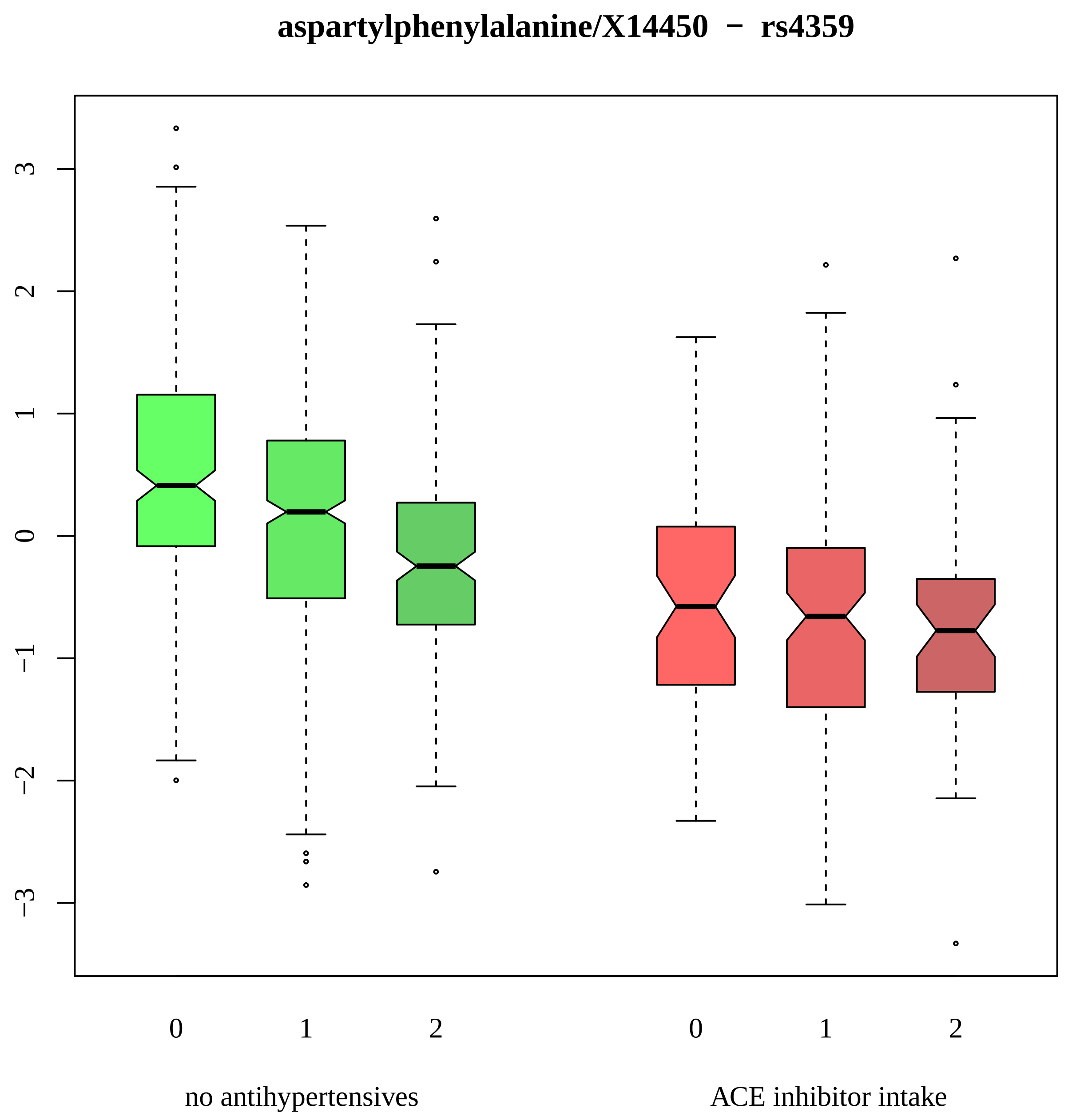

aspartylphenylalanine/X14450 – rs4359
3
2
1
0
−1
−2
−3
0
1
2
0
1
2
no antihypertensives
ACE inhibitor intake

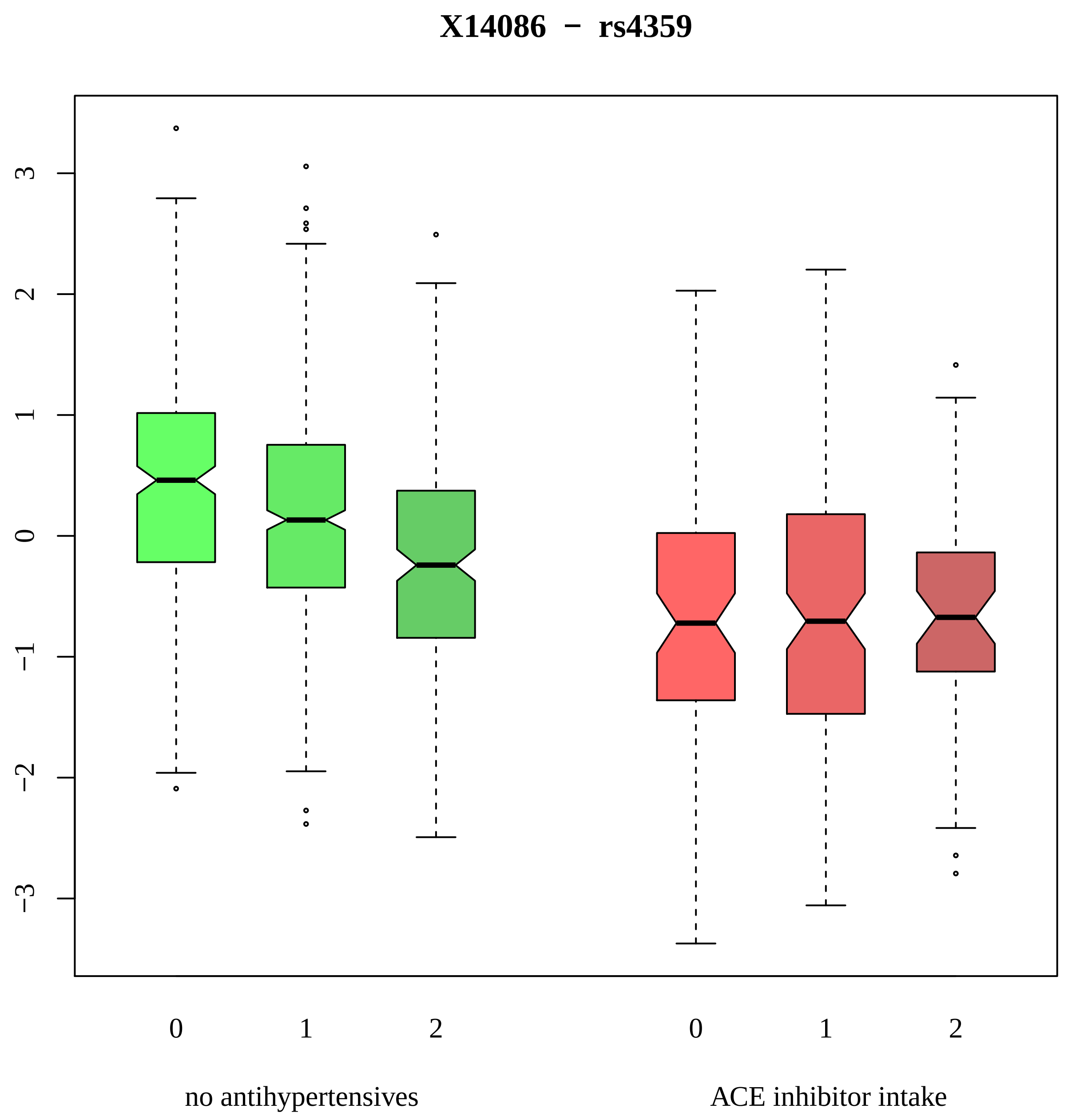

X14086 – rs4359
3
2
1
0
−1
−2
−3
0
1
2
0
1
2
no antihypertensives
ACE inhibitor intake

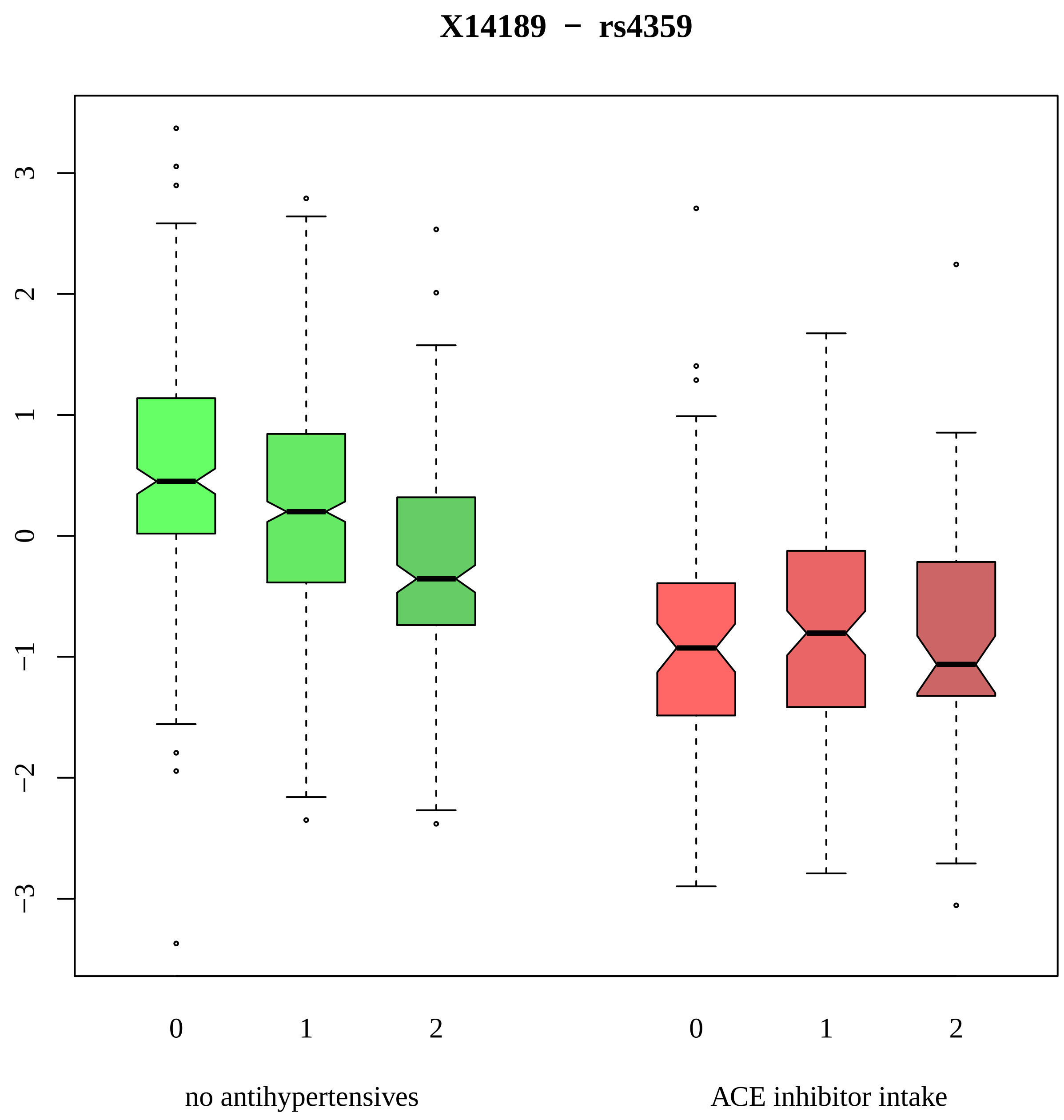
X14189 – rs4359
3
2
1
0
−1
−2
−3
0
1
2
0
1
2
no antihypertensives
ACE inhibitor intake

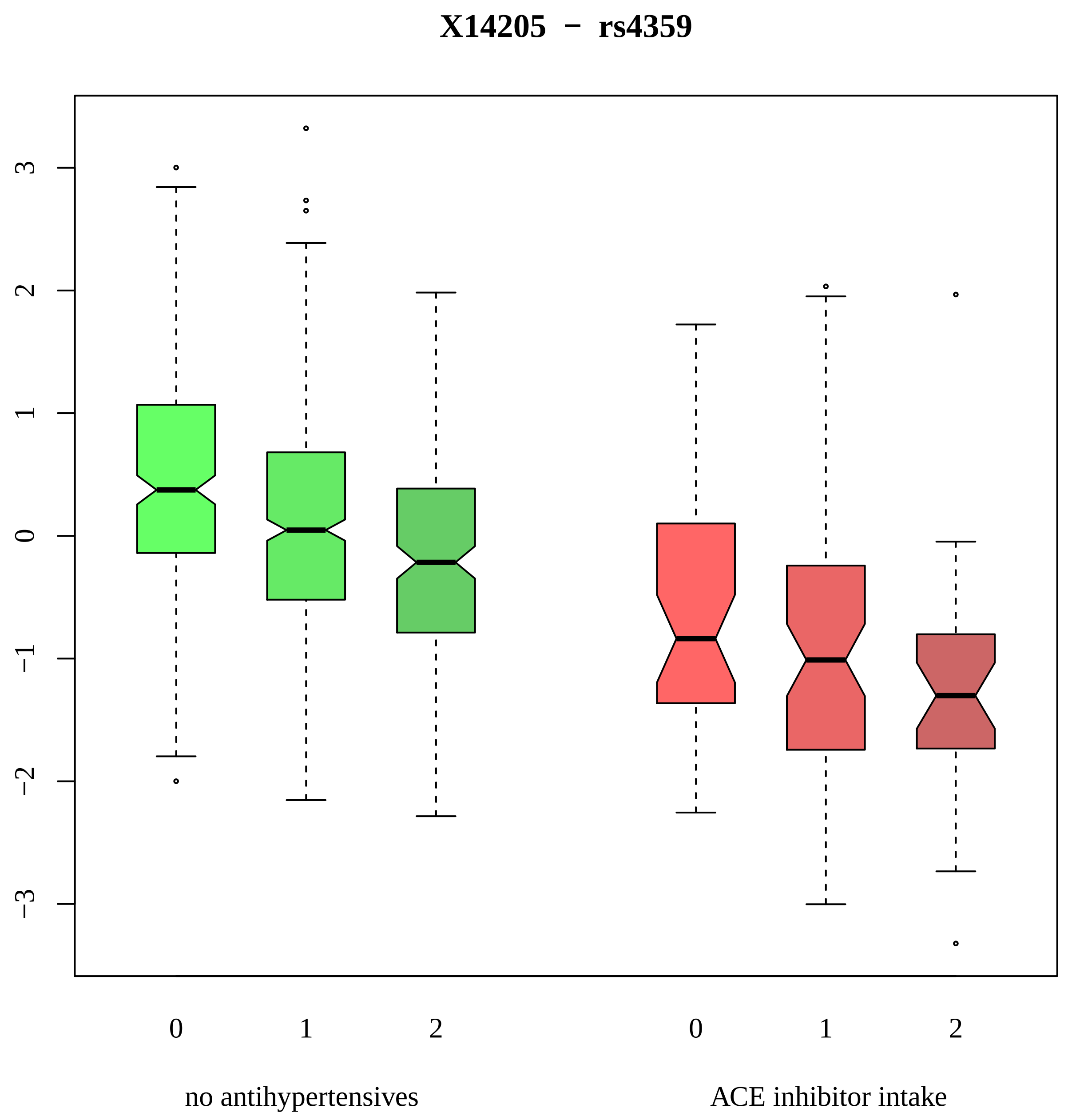
X14205 – rs4359
3
2
1
0
−1
−2
−3
0
1
2
0
1
2
no antihypertensives
ACE inhibitor intake

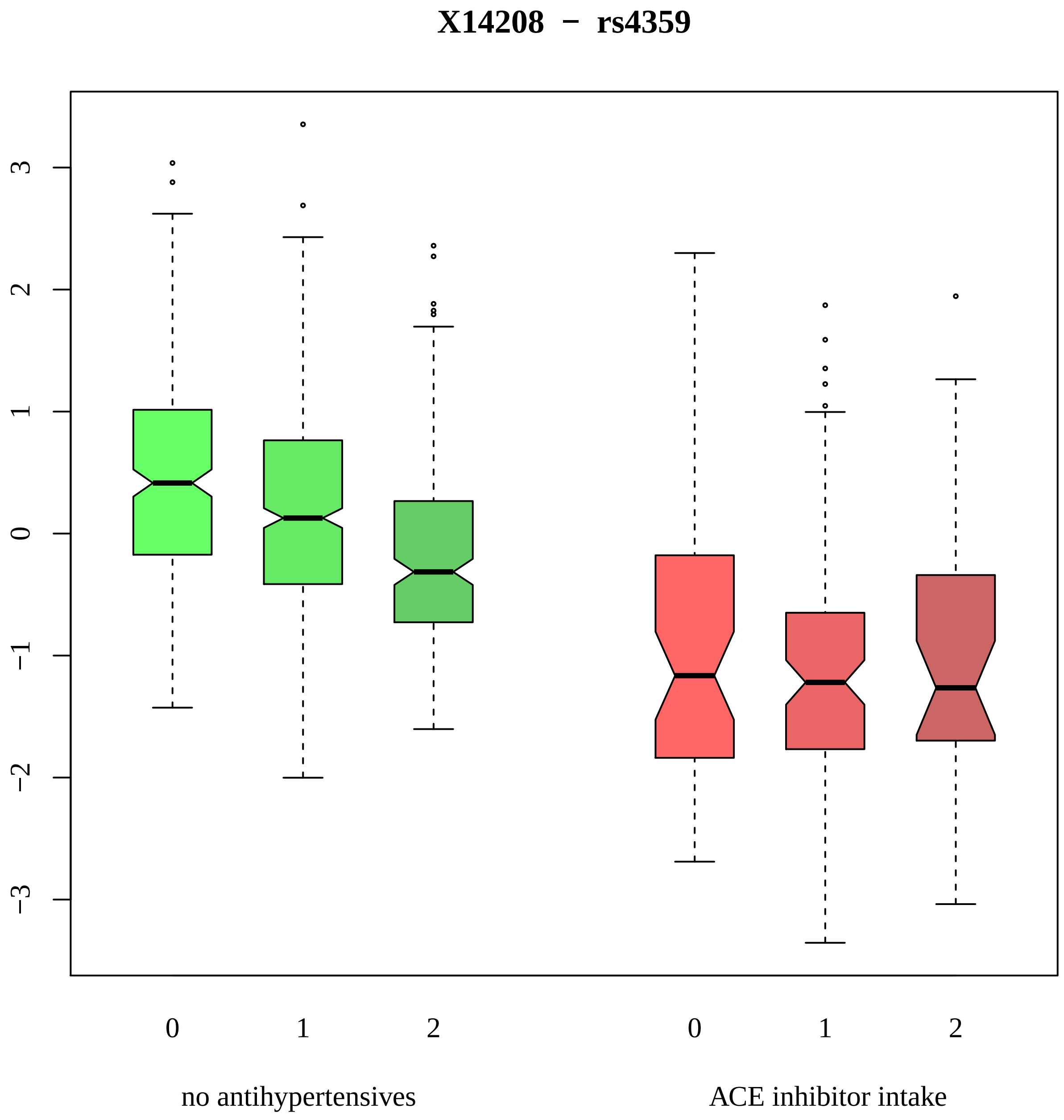
X14208 – rs4359
3
2
1
0
−1
−2
−3
0
1
2
0
1
2
no antihypertensives
ACE inhibitor intake

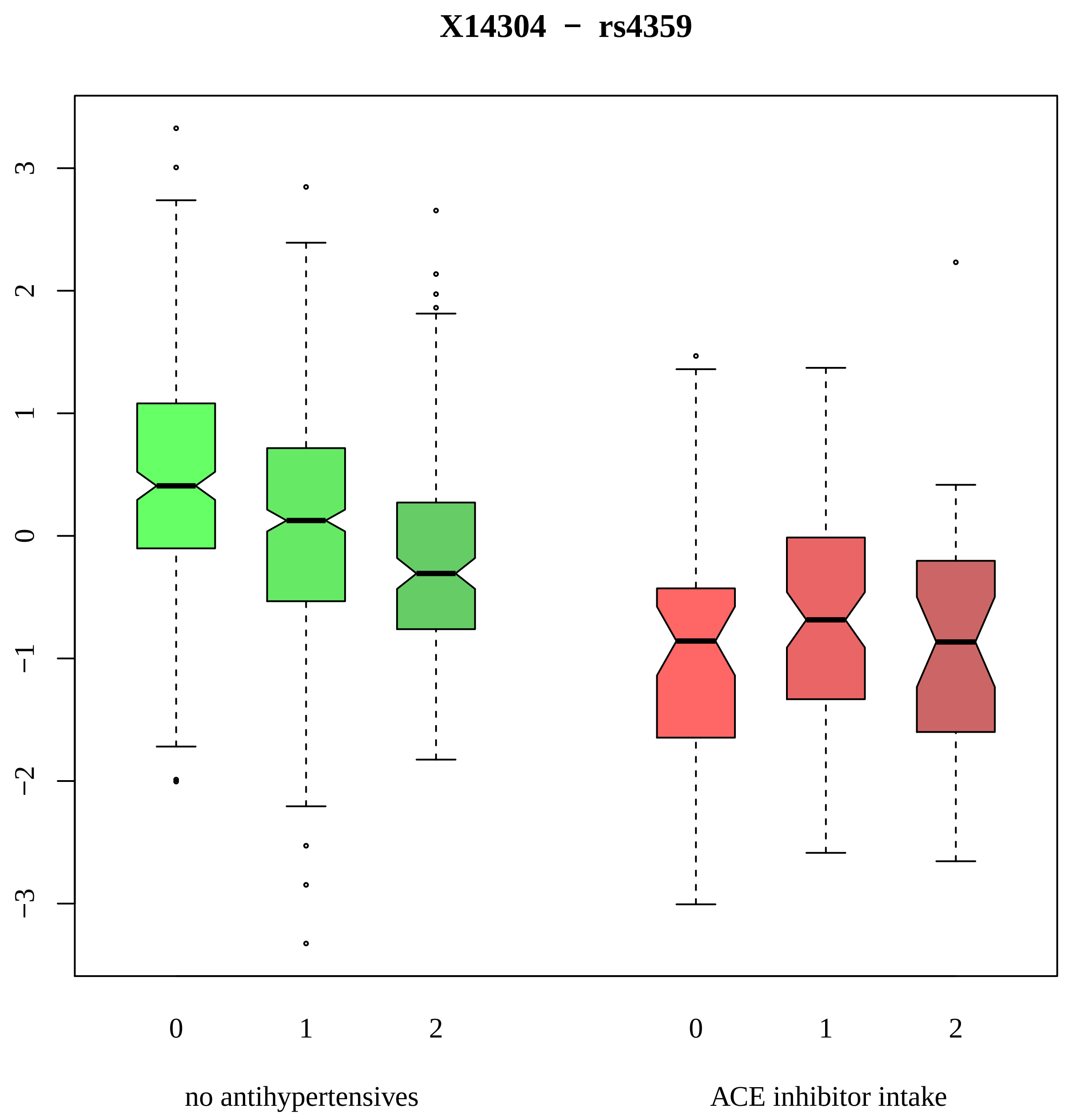
X14304 – rs4359
3
2
1
0
−1
−2
−3
0
1
2
0
1
2
no antihypertensives
ACE inhibitor intake

aspartylphenylalanine − rs4362

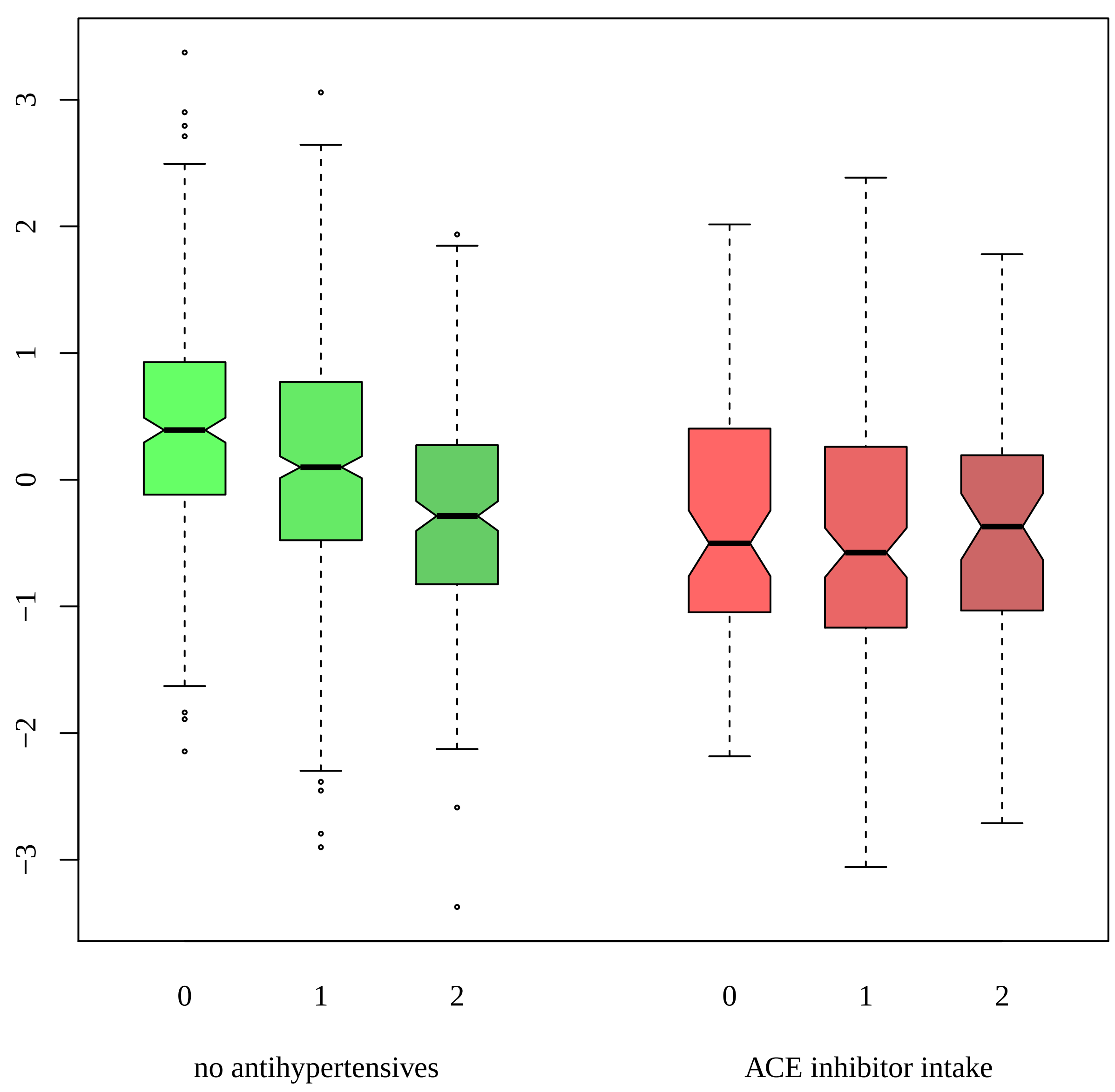

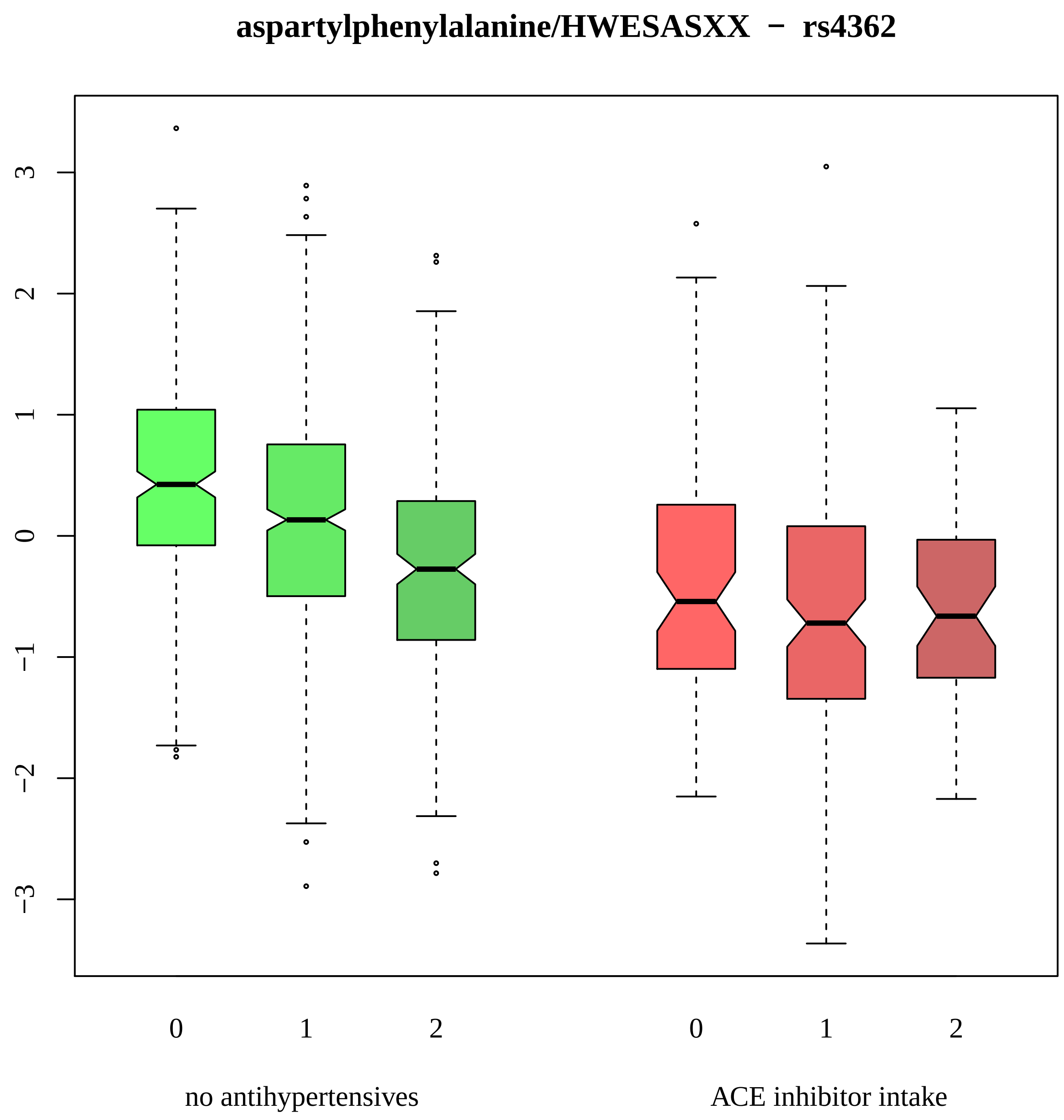
aspartylphenylalanine/HWESASXX – rs4362
3
2
1
0
−1
−2
−3
0
1
2
0
1
2
no antihypertensives
ACE inhibitor intake

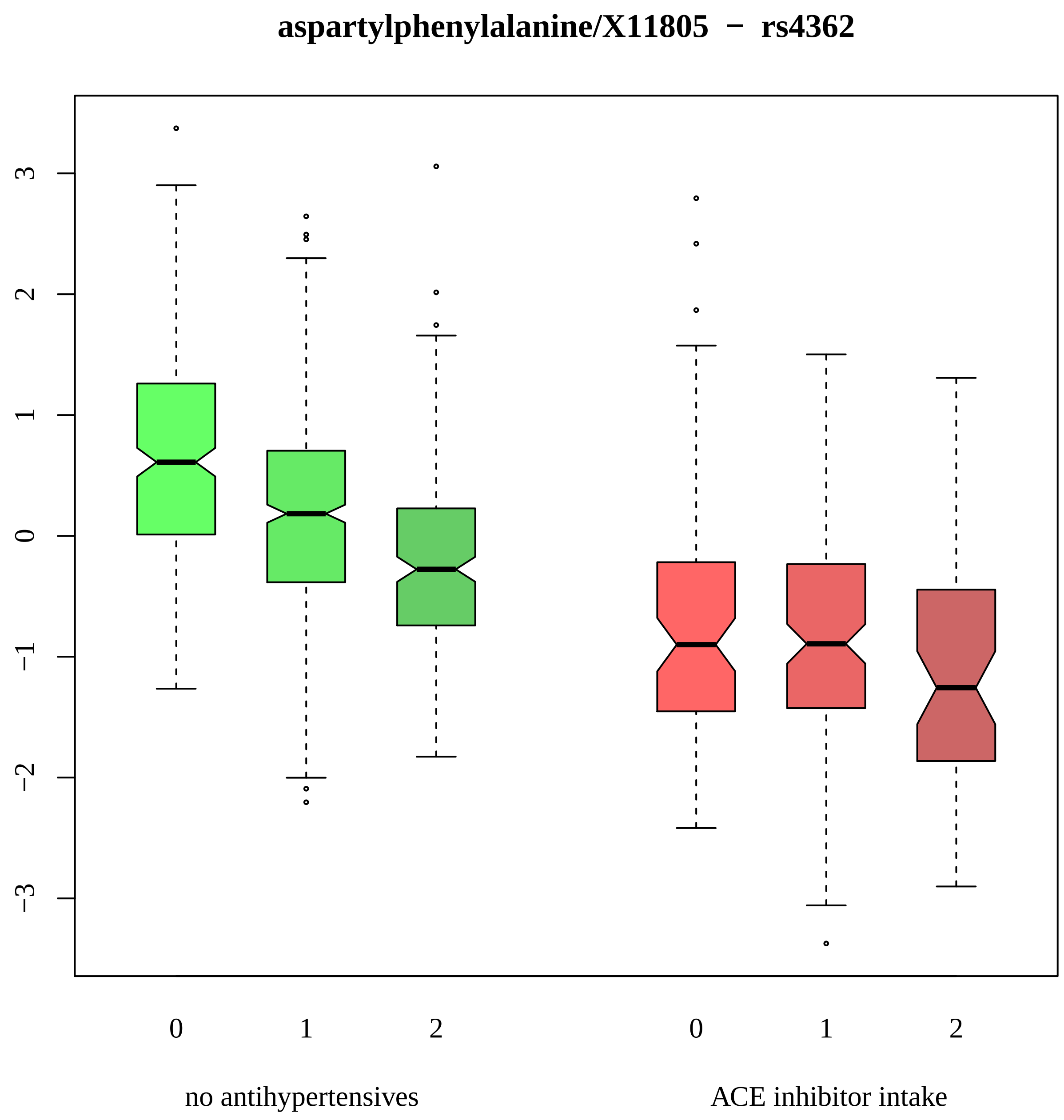

aspartylphenylalanine/X11805 – rs4362
3
2
1
0
−1
−2
−3
0
1
2
0
1
2
no antihypertensives
ACE inhibitor intake

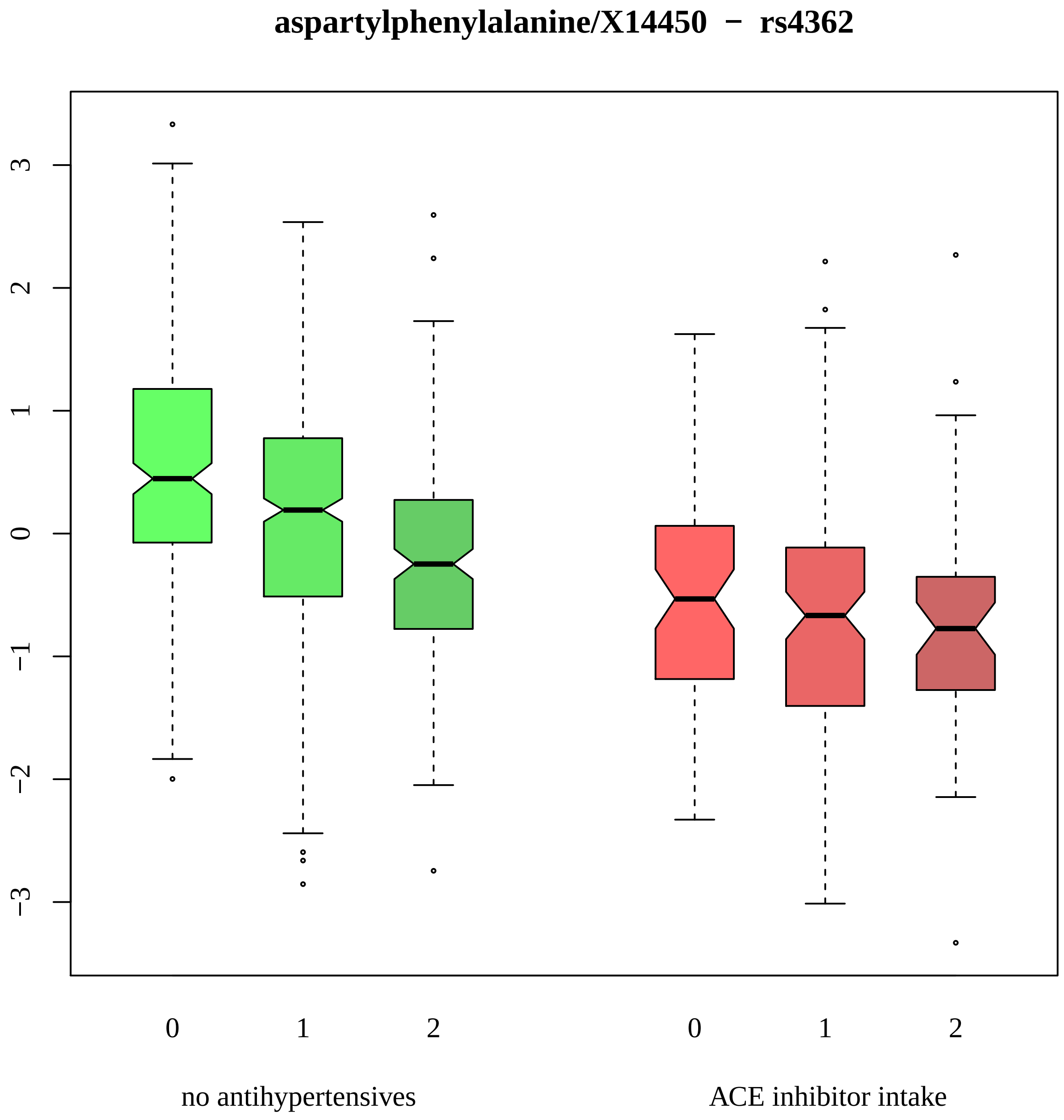

aspartylphenylalanine/X14450 − rs4362
3
2
1
0
−1
−2
−3
0
1
2
0
1
2
no antihypertensives
ACE inhibitor intake

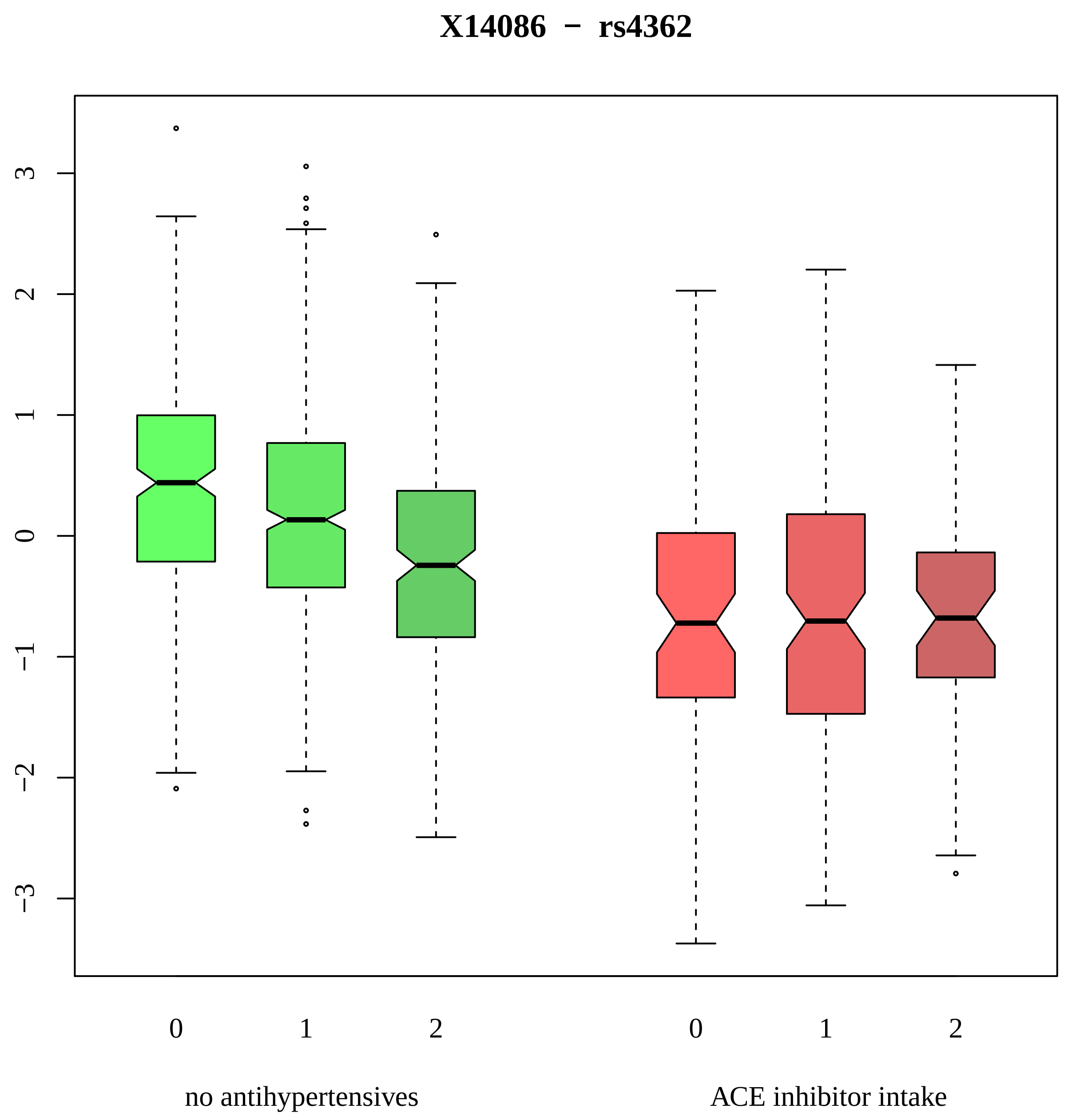
X14086 – rs4362
3
2
1
0
−1
−2
−3
0
1
2
0
1
2
no antihypertensives
ACE inhibitor intake

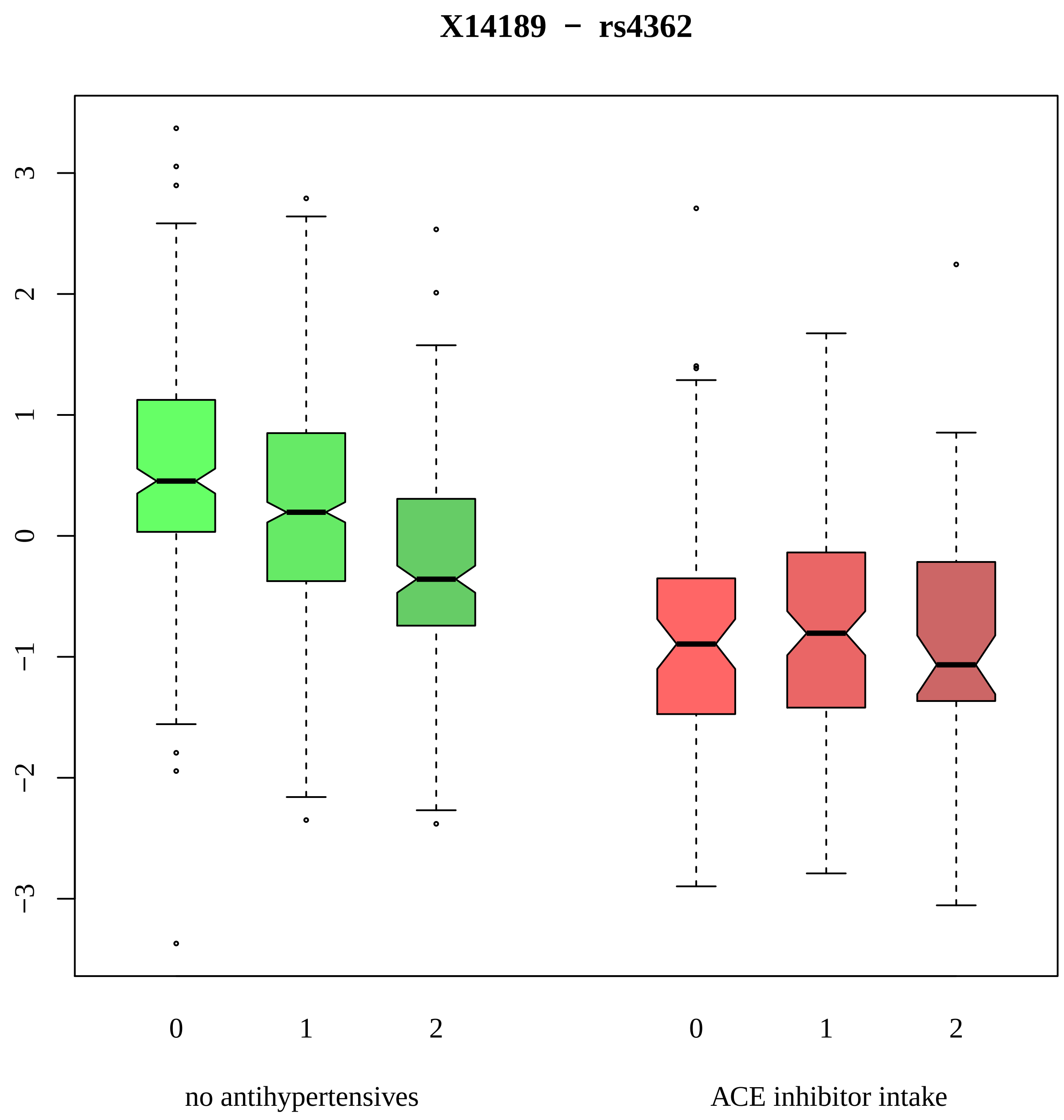
X14189 – rs4362
3
2
1
0
−1
−2
−3
0
1
2
0
1
2
no antihypertensives
ACE inhibitor intake

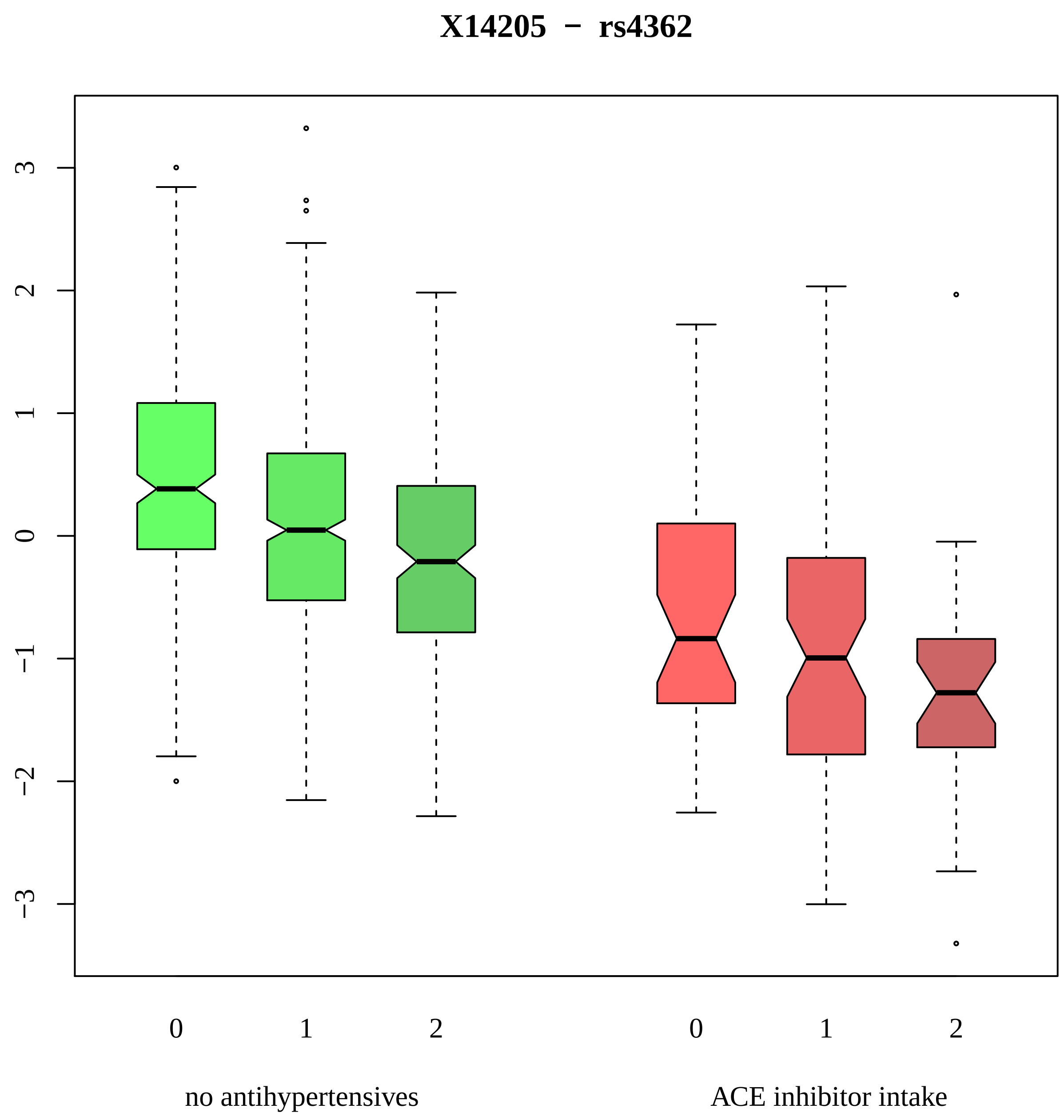
X14205 – rs4362
3
2
1
0
−1
−2
−3
0
1
2
0
1
2
no antihypertensives
ACE inhibitor intake

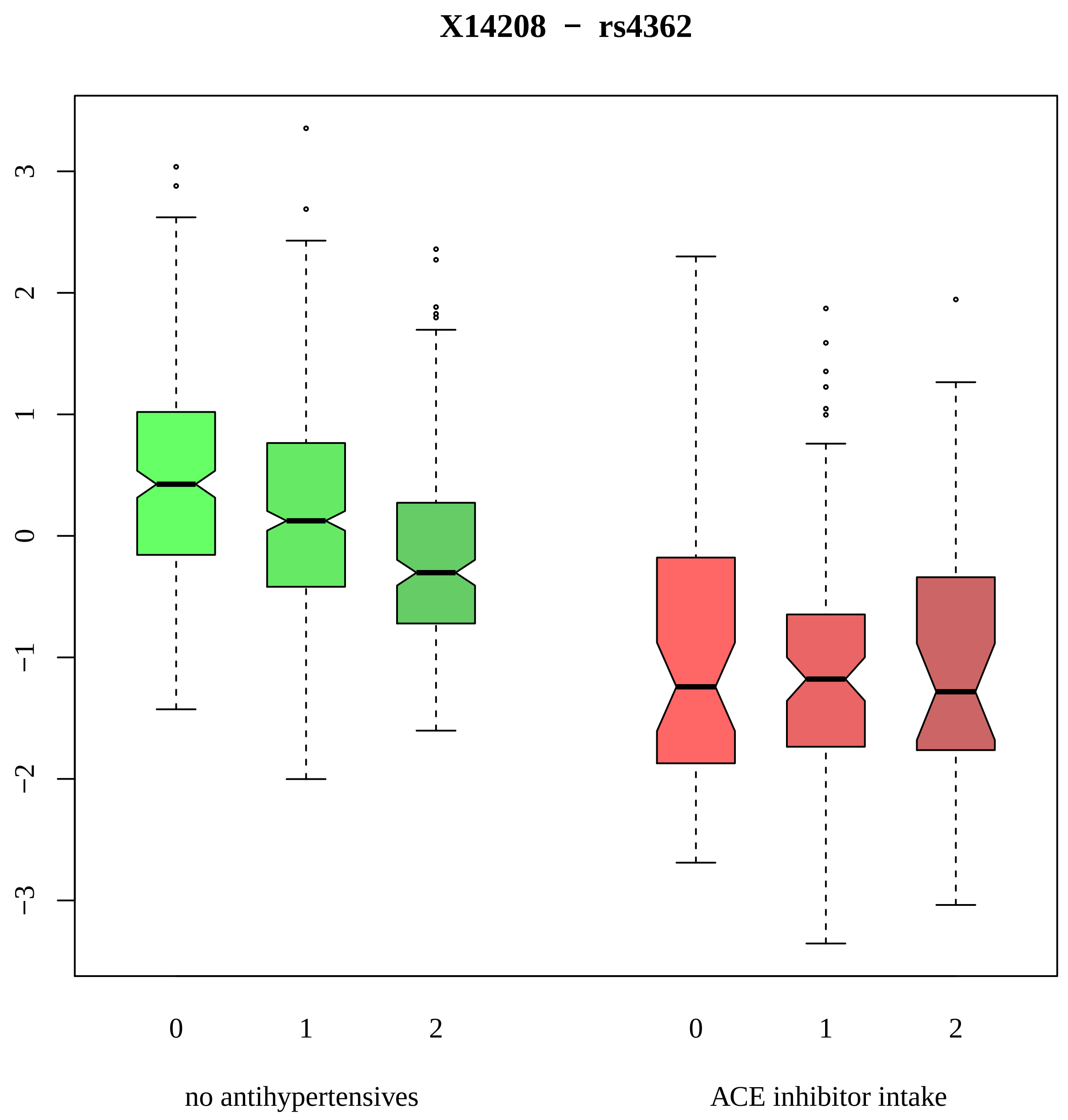

X14208 – rs4362
3
2
1
0
−1
−2
−3
0
1
2
0
1
2
no antihypertensives
ACE inhibitor intake

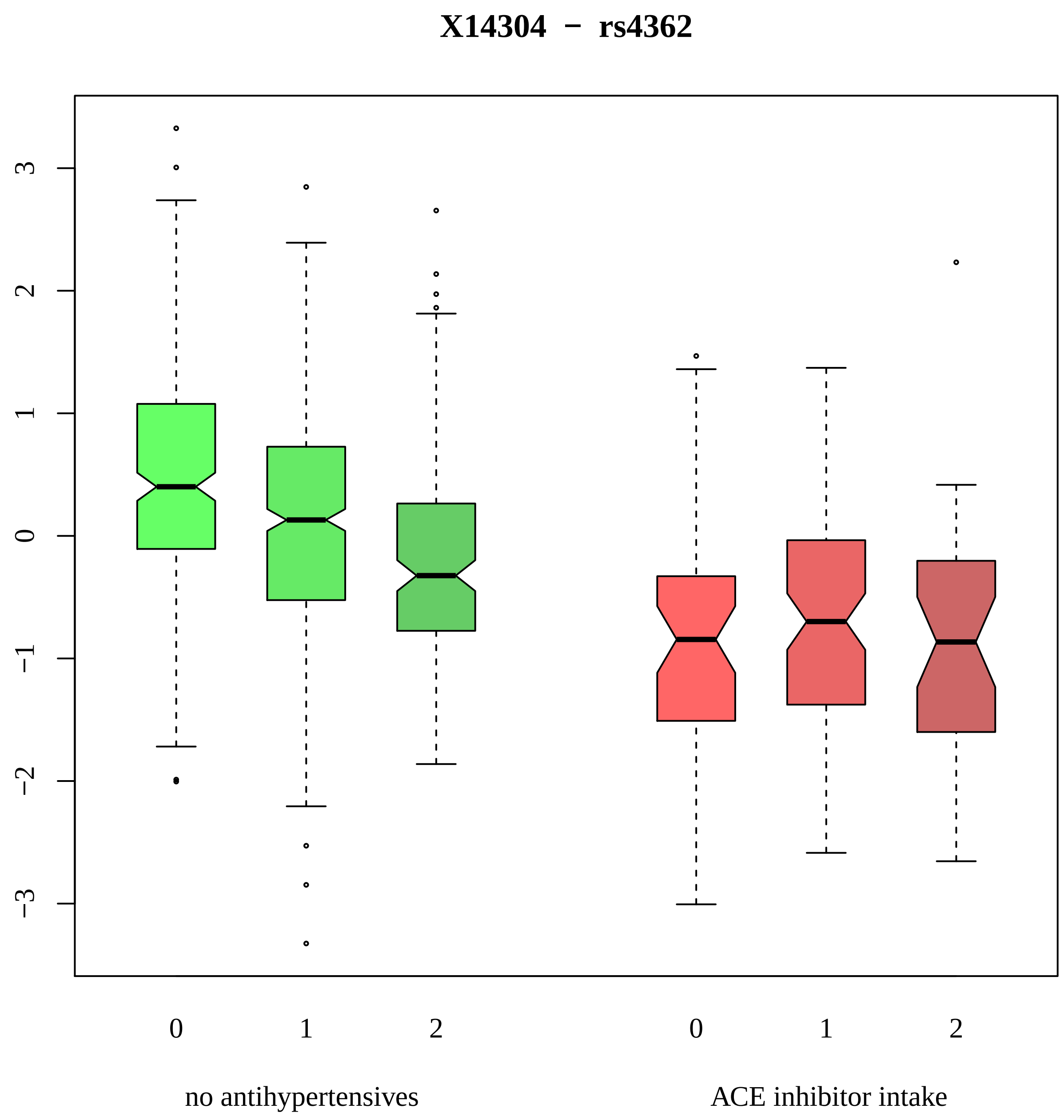

X14304 – rs4362
3
2
1
0
−1
−2
−3
0
1
2
0
1
2
no antihypertensives
ACE inhibitor intake

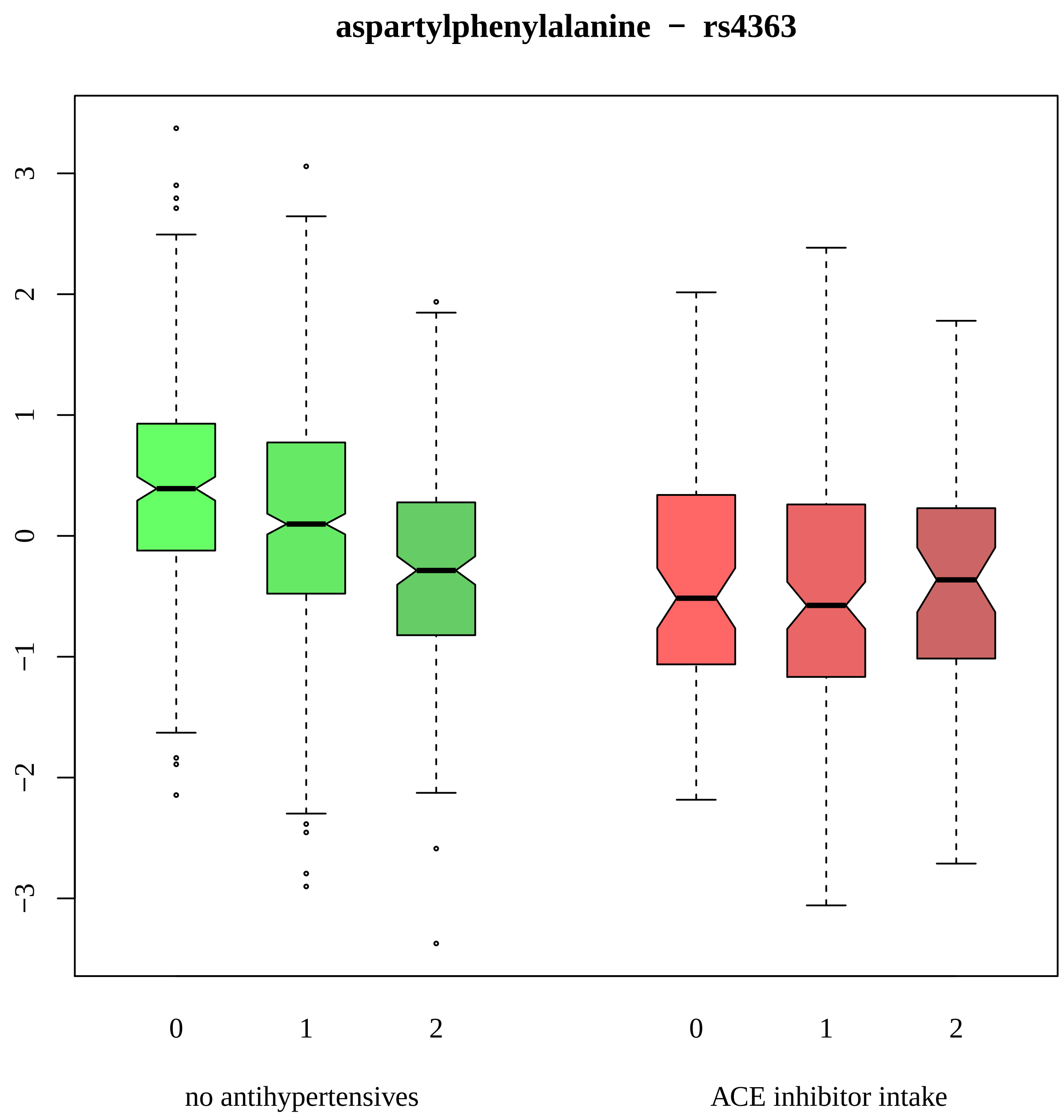

aspartylphenylalanine − rs4363
3
2
1
0
−1
−2
−3
0
1
2
0
1
2
no antihypertensives
ACE inhibitor intake

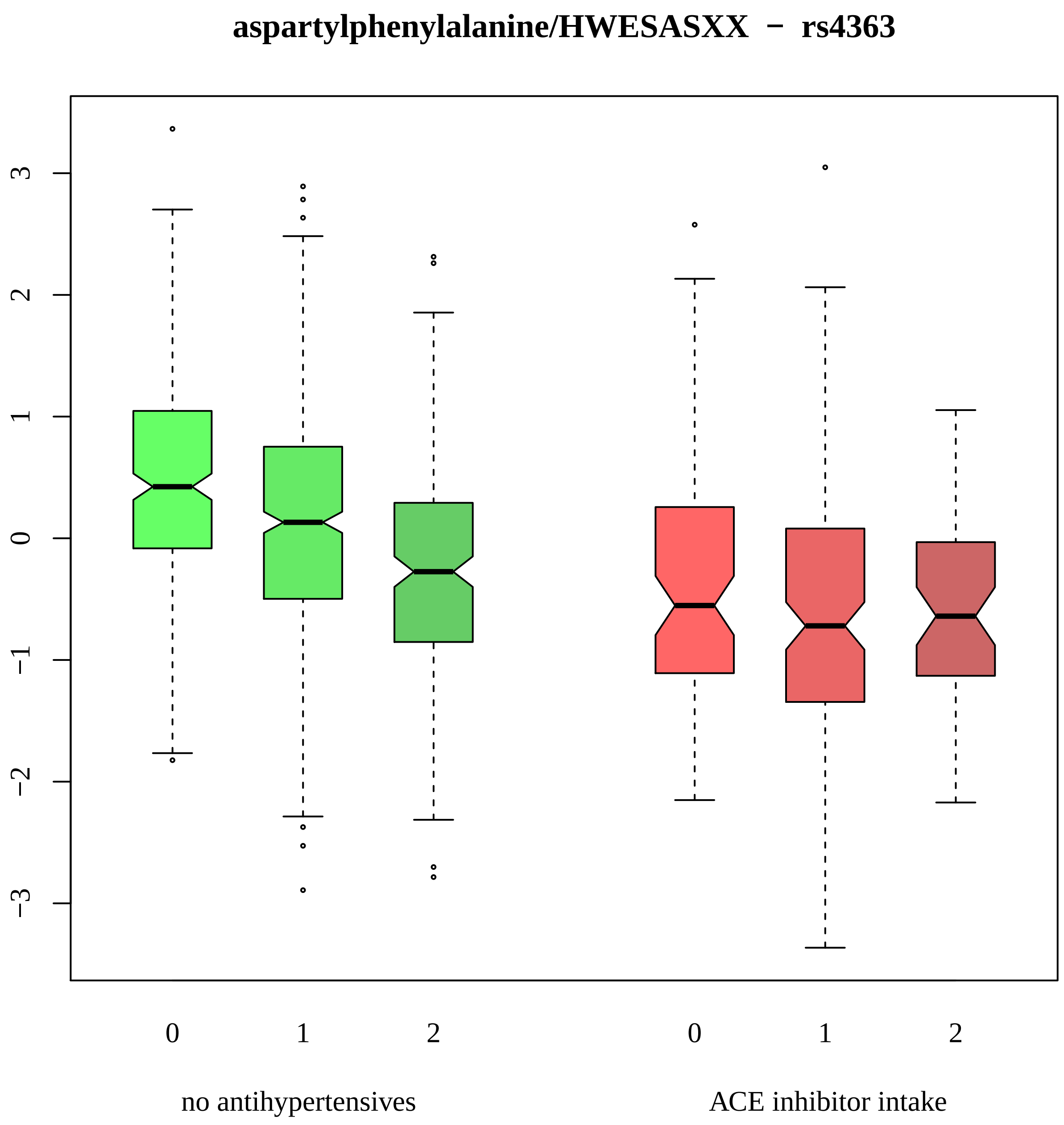

aspartylphenylalanine/HWESASXX − rs4363
3
2
1
0
−1
−2
−3
0
1
2
0
1
2
no antihypertensives
ACE inhibitor intake

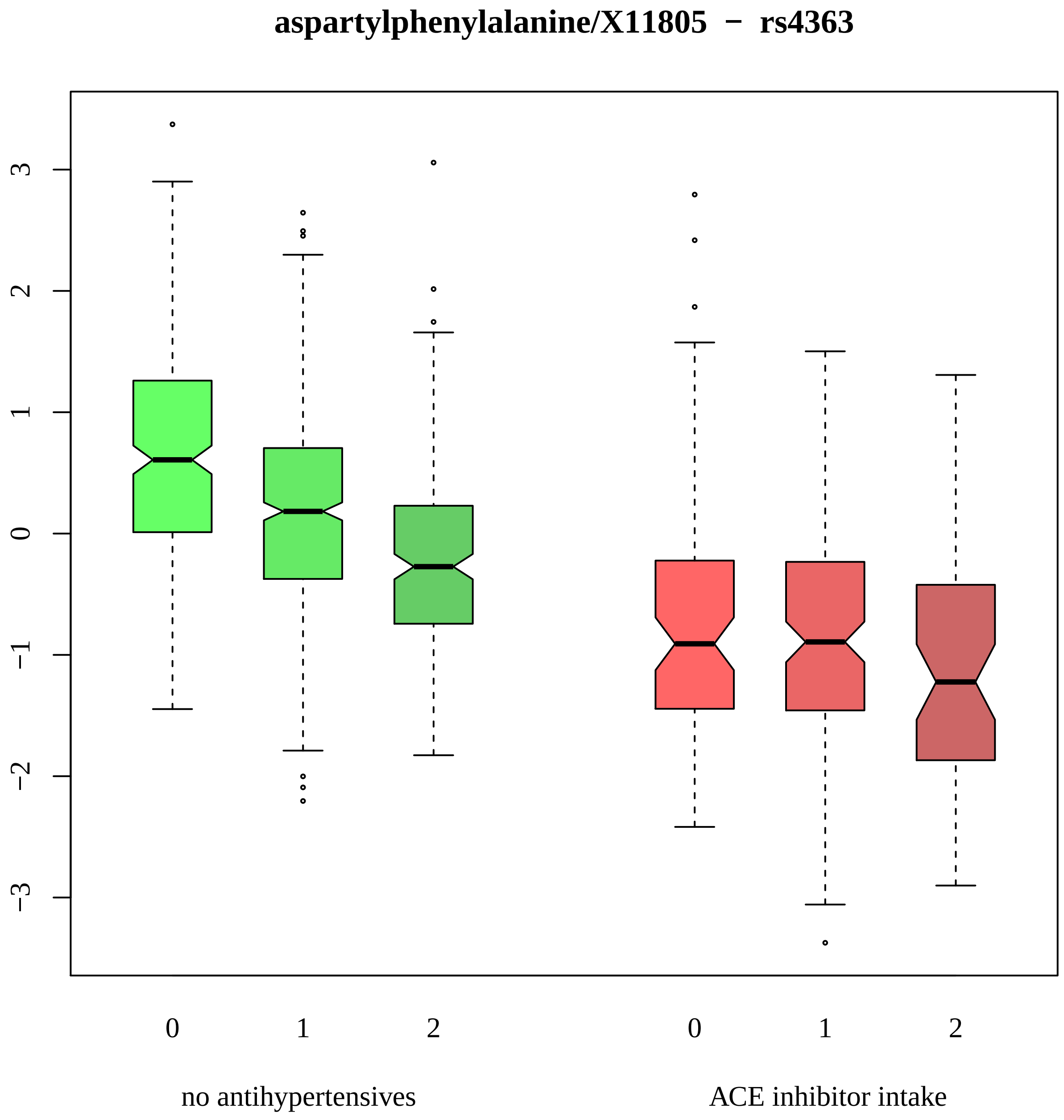

aspartylphenylalanine/X11805 − rs4363
3
2
1
0
−1
−2
−3
0
1
2
0
1
2
no antihypertensives
ACE inhibitor intake

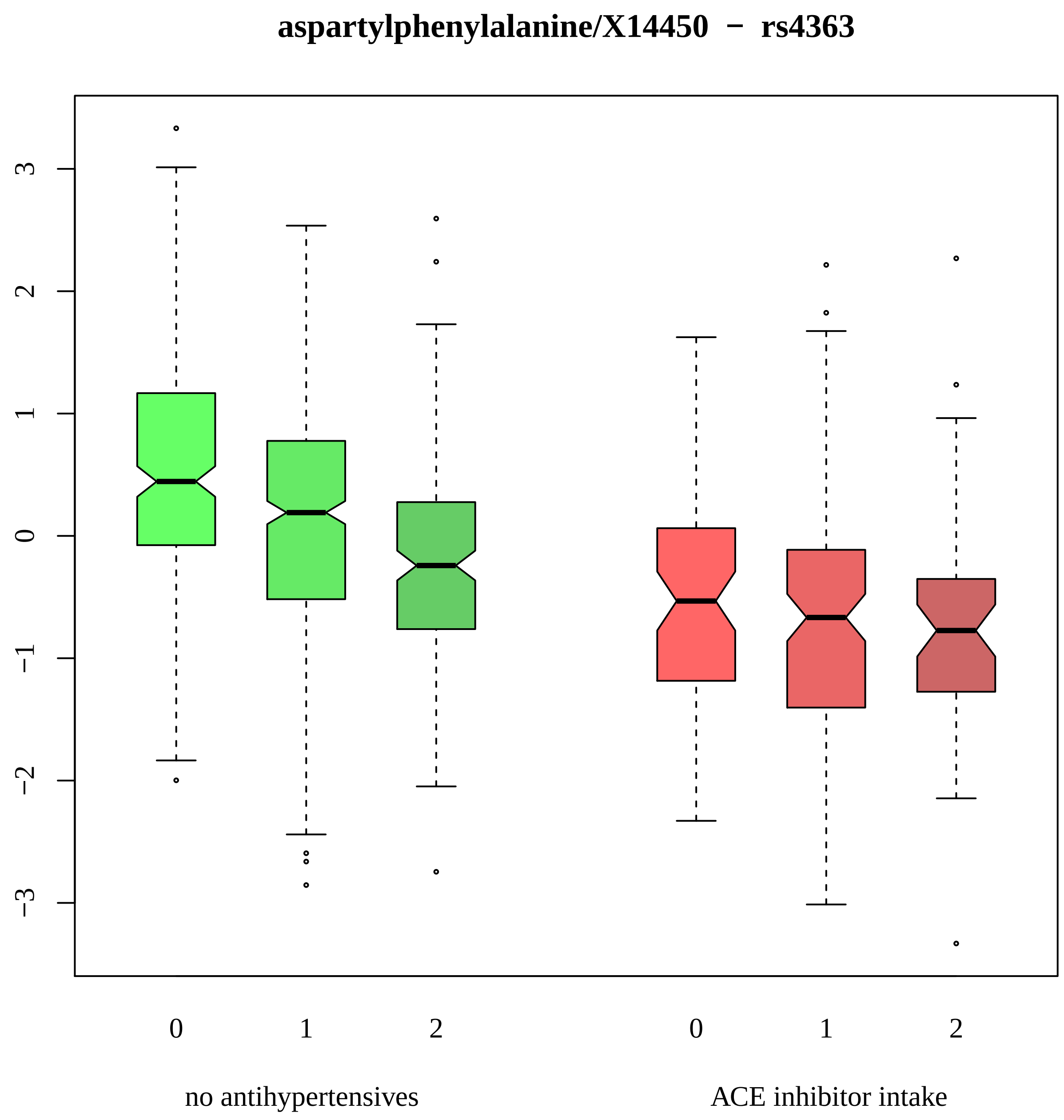
aspartylphenylalanine/X14450 – rs4363
3
2
1
0
−1
−2
−3
0
1
2
0
1
2
no antihypertensives
ACE inhibitor intake

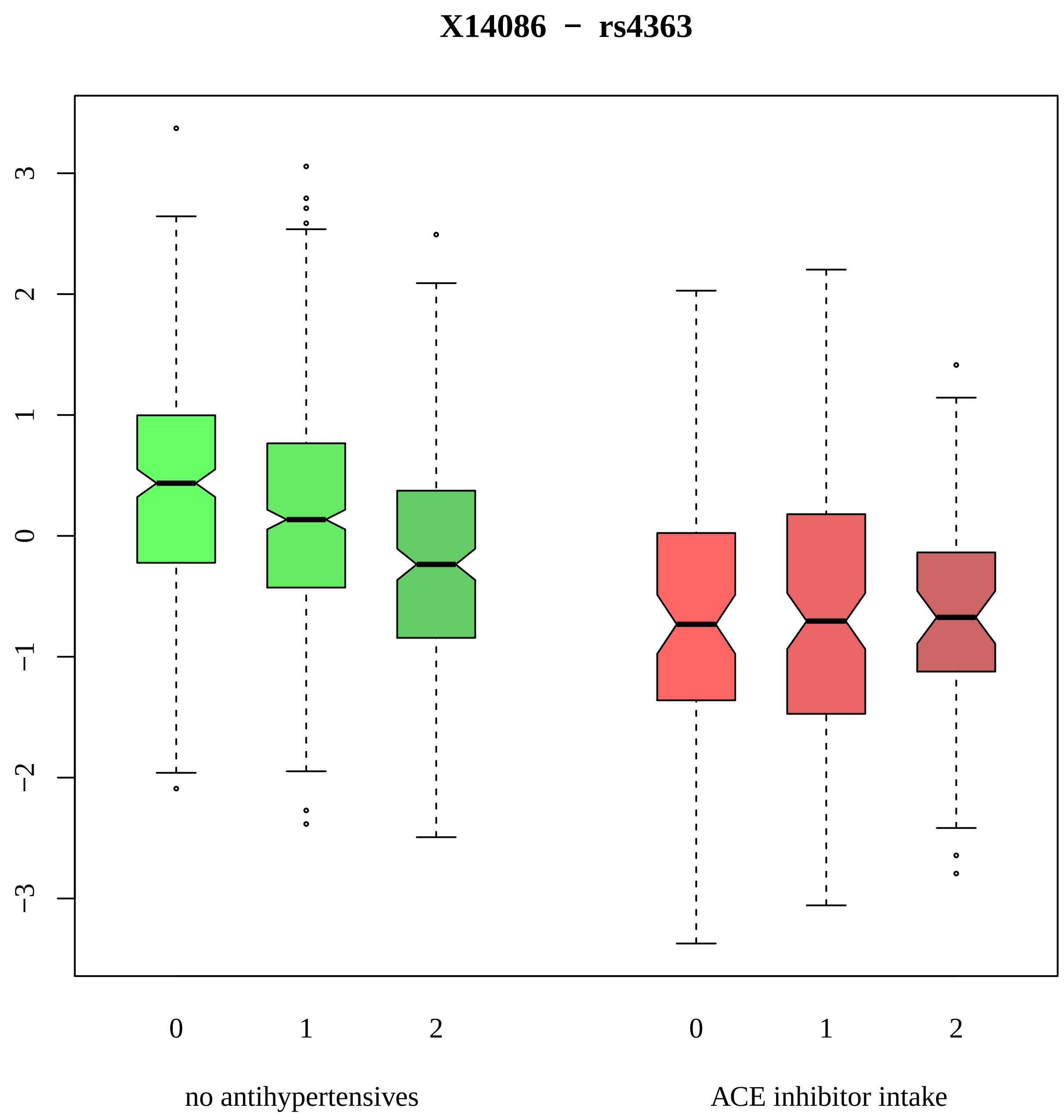
X14086 – rs4363
3
2
1
0
−1
−2
−3
0
1
2
0
1
2
no antihypertensives
ACE inhibitor intake

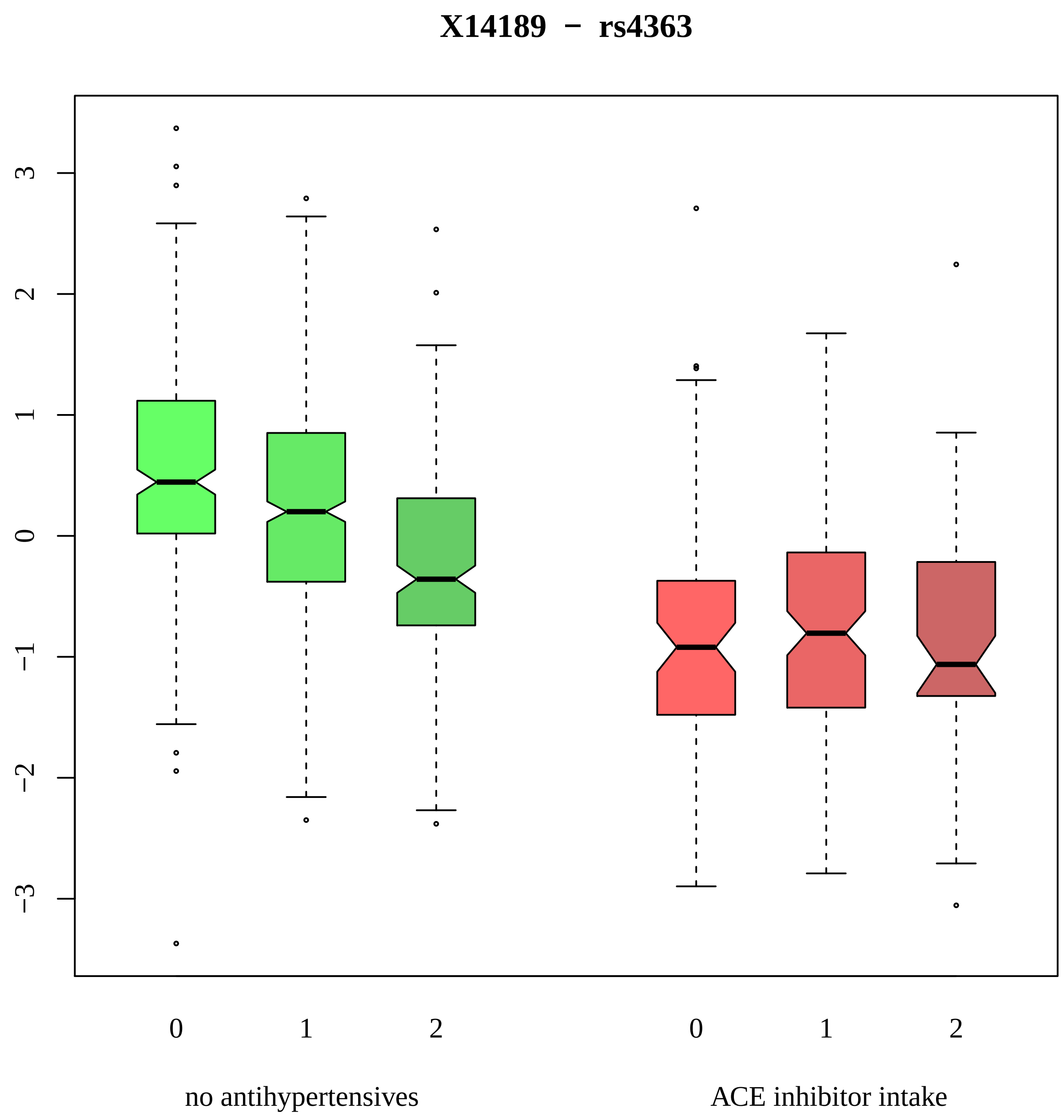

X14189 – rs4363
3
2
1
0
−1
−2
−3
0
1
2
0
1
2
no antihypertensives
ACE inhibitor intake

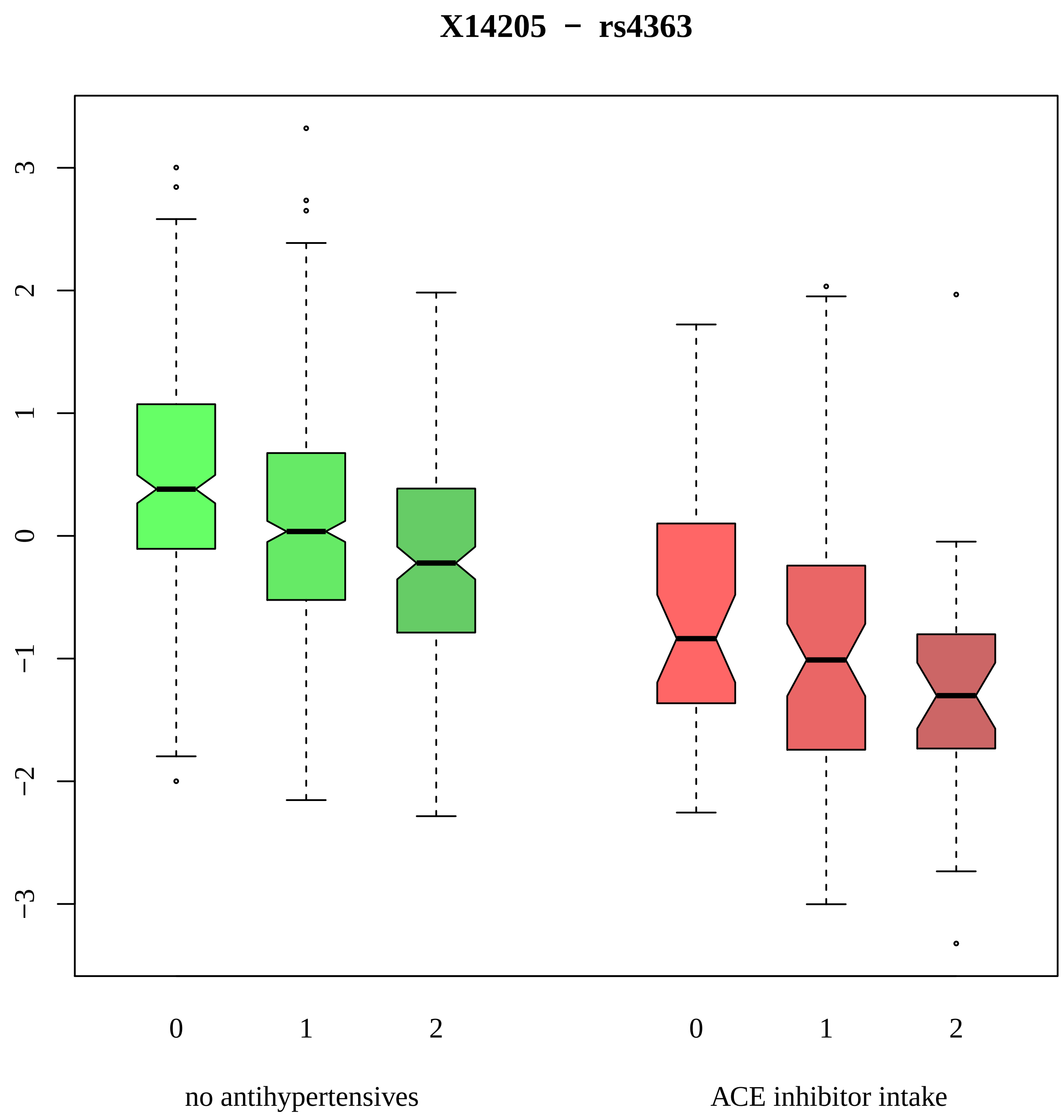

X14205 – rs4363
3
2
1
0
−1
−2
−3
0
1
2
0
1
2
no antihypertensives
ACE inhibitor intake

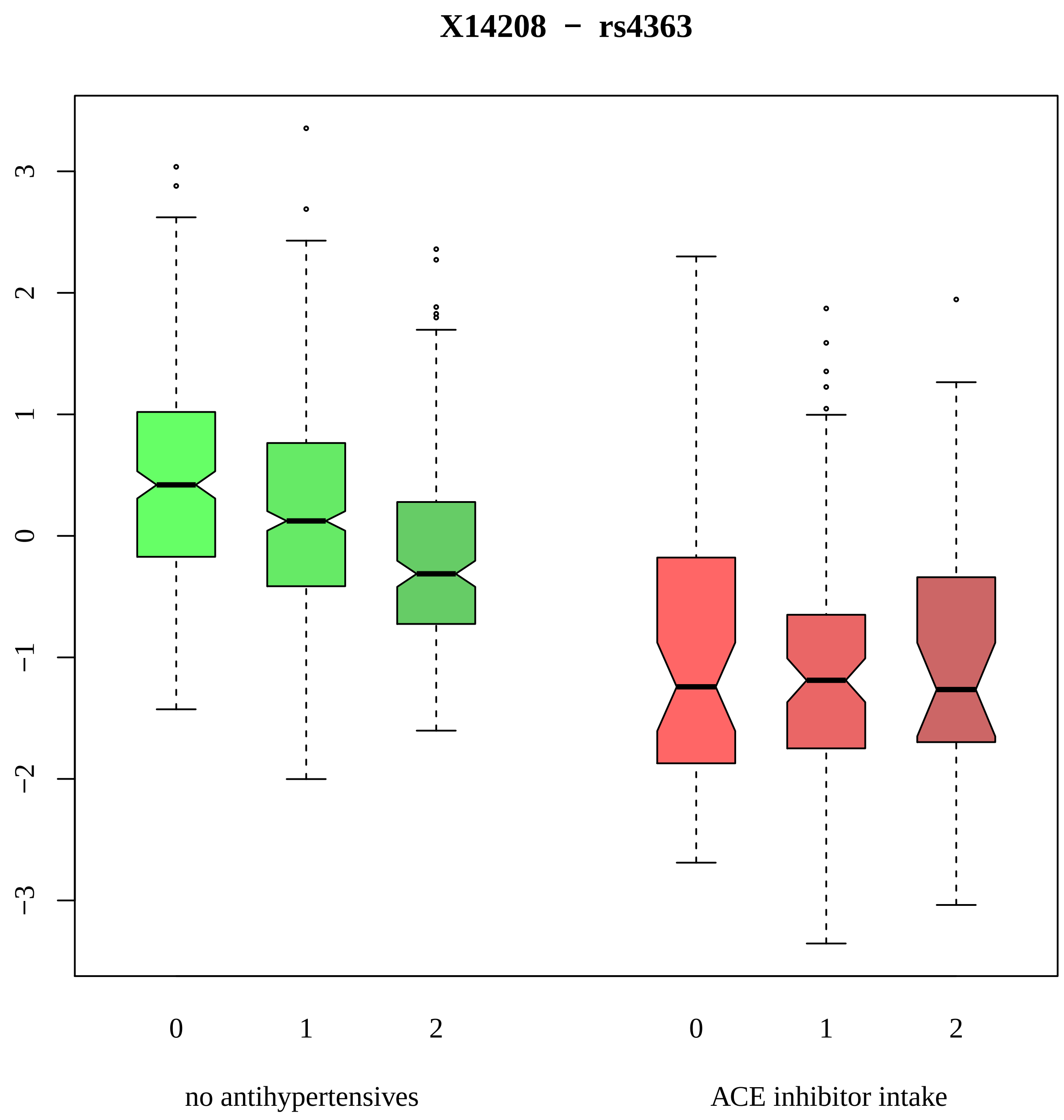
X14208 – rs4363
3
2
1
0
−1
−2
−3
0
1
2
0
1
2
no antihypertensives
ACE inhibitor intake

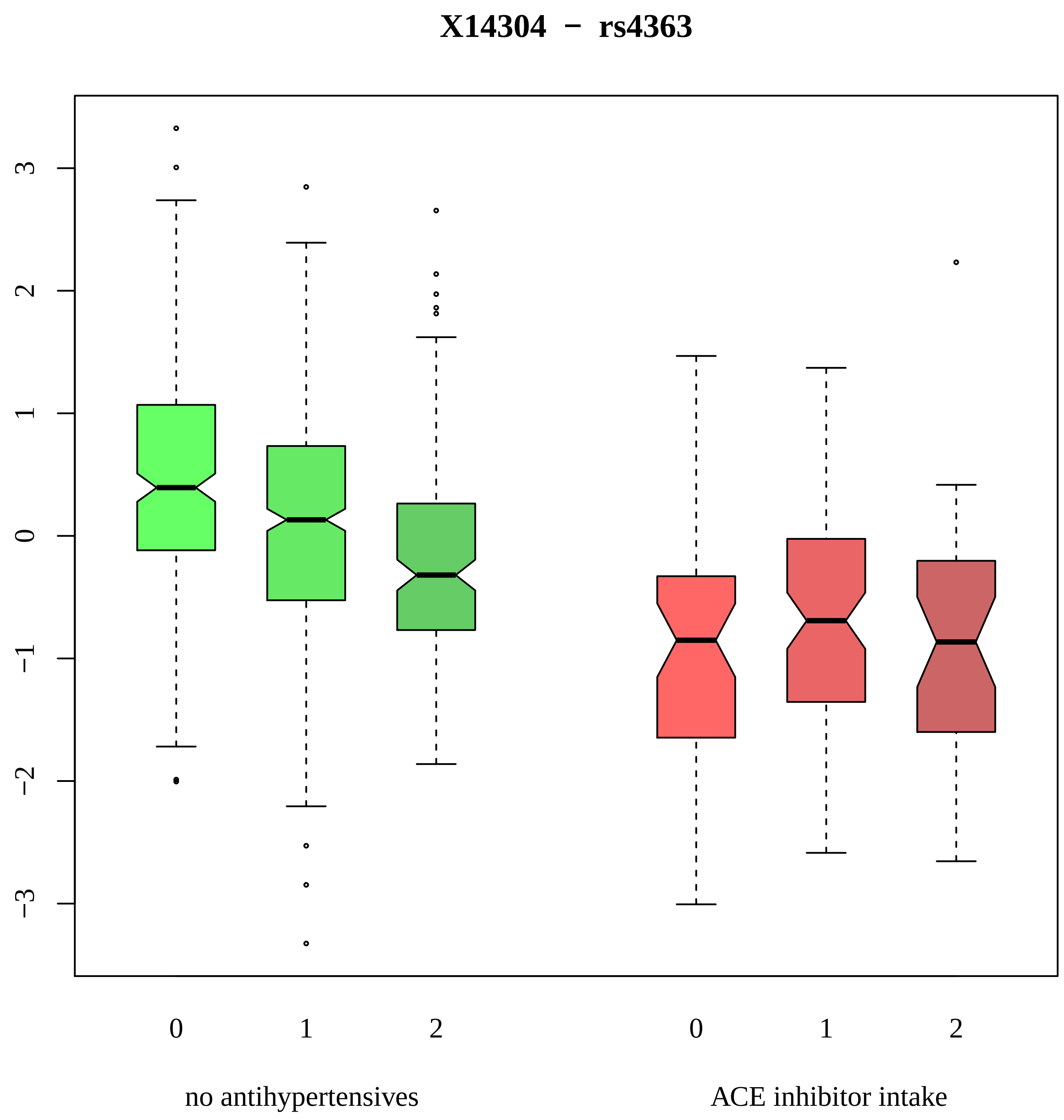

X14304 – rs4363
3
2
1
0
−1
−2
−3
0
1
2
0
1
2
no antihypertensives
ACE inhibitor intake

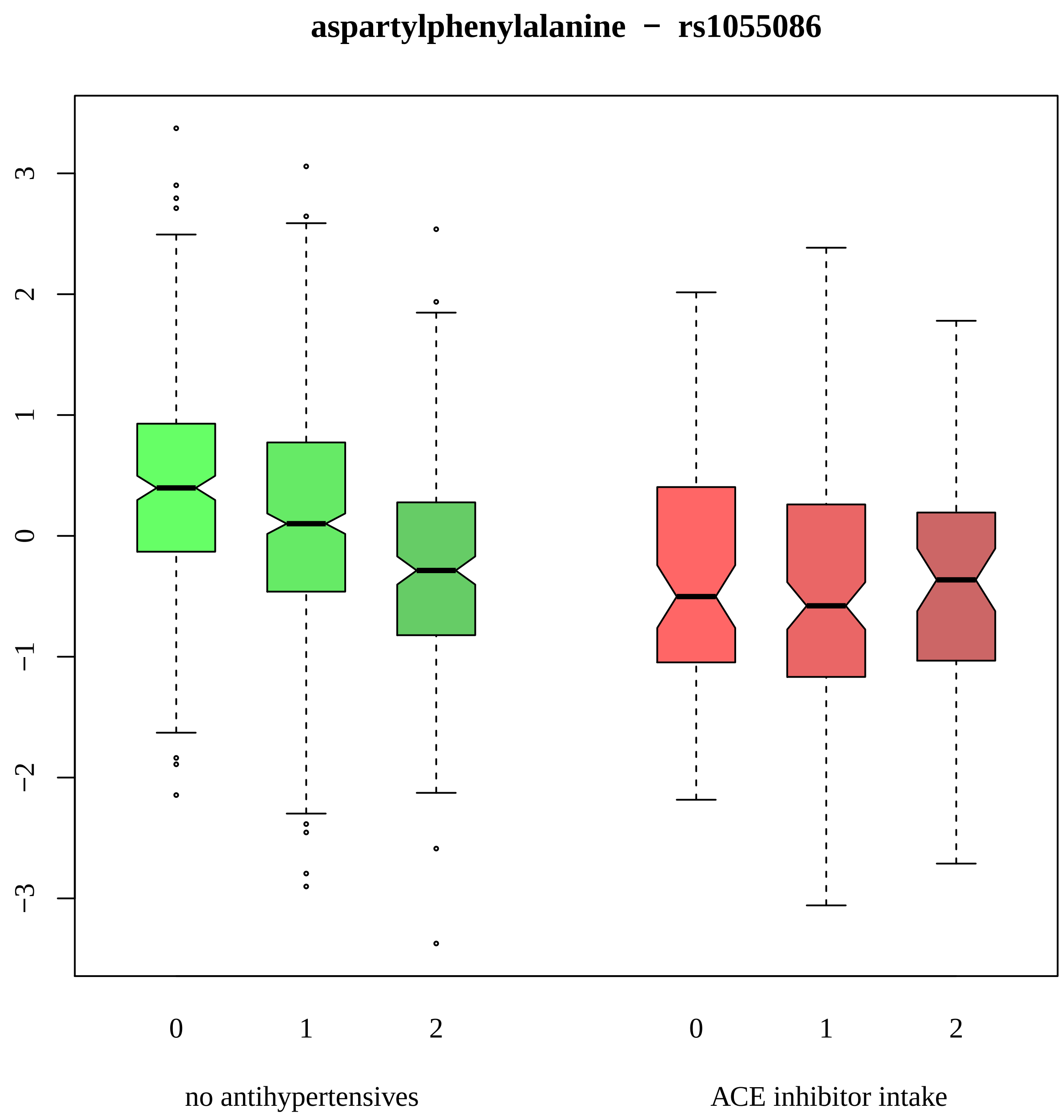

aspartylphenylalanine − rs1055086
3
2
1
0
−1
−2
−3
0
1
2
0
1
2
no antihypertensives
ACE inhibitor intake

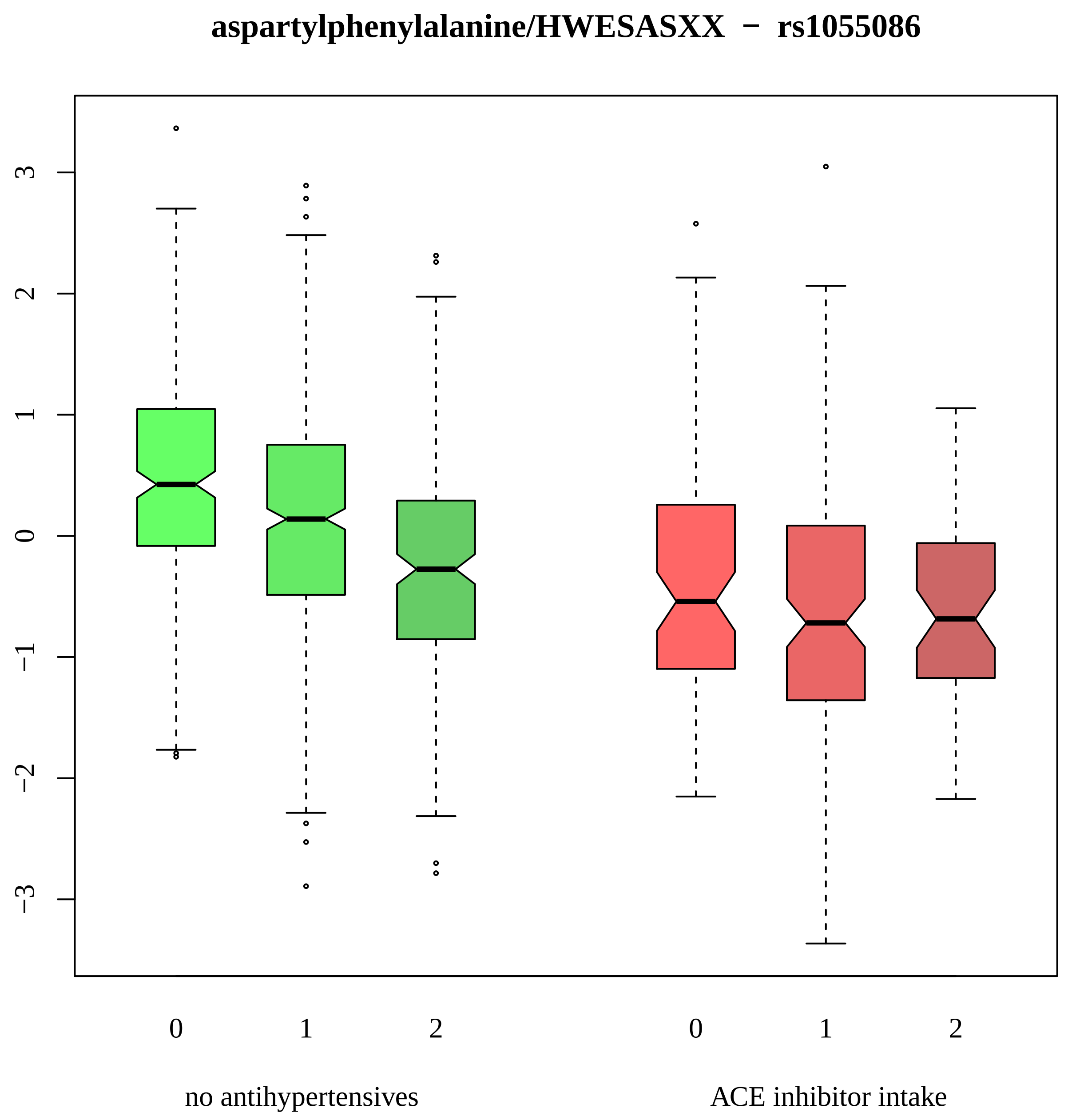

aspartylphenylalanine/HWESASXX – rs1055086
3
2
1
0
−1
−2
−3
0
1
2
0
1
2
no antihypertensives
ACE inhibitor intake

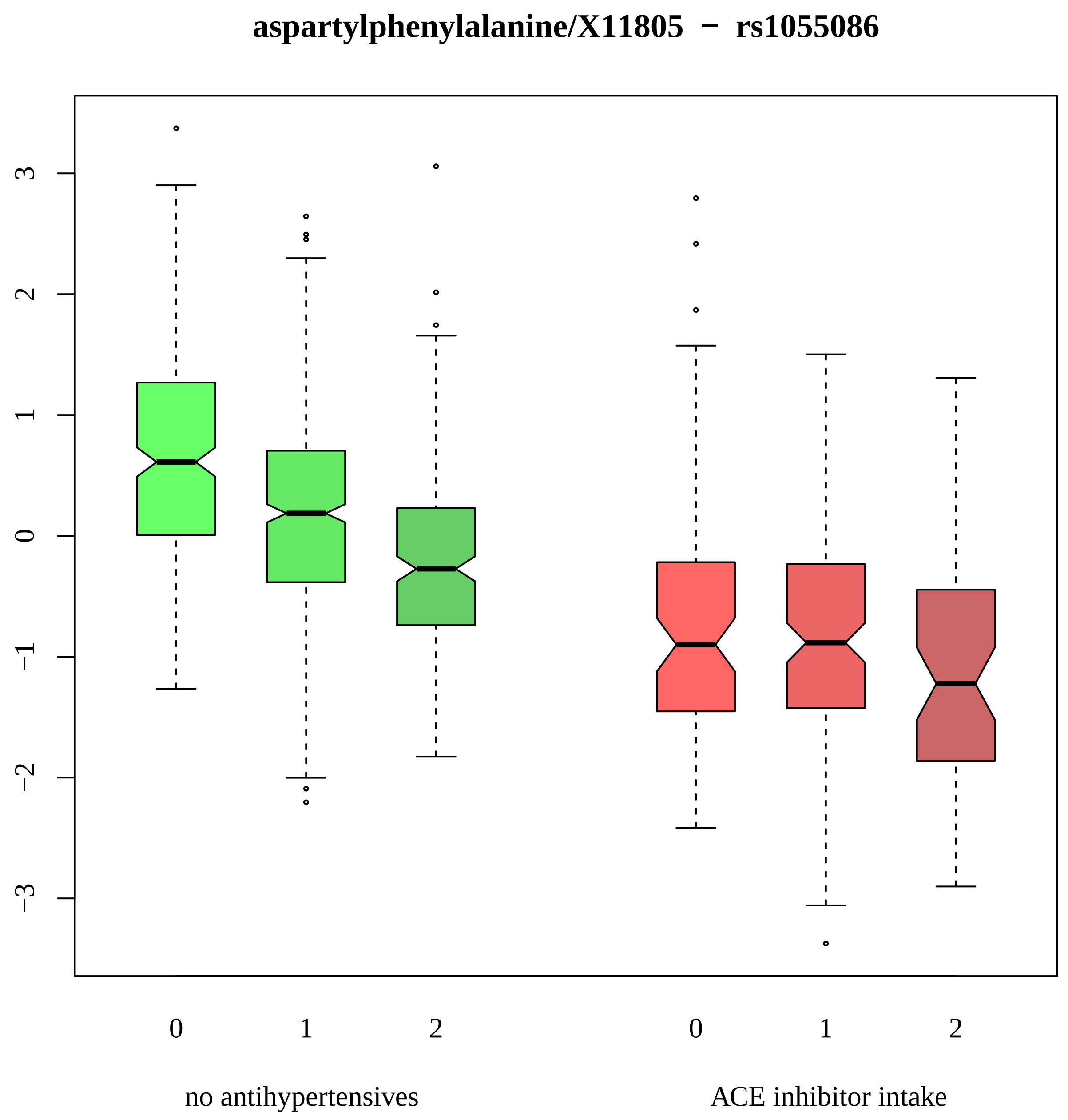
aspartylphenylalanine/X11805 − rs1055086
3
2
1
0
−1
−2
−3
0
1
2
0
1
2
no antihypertensives
ACE inhibitor intake

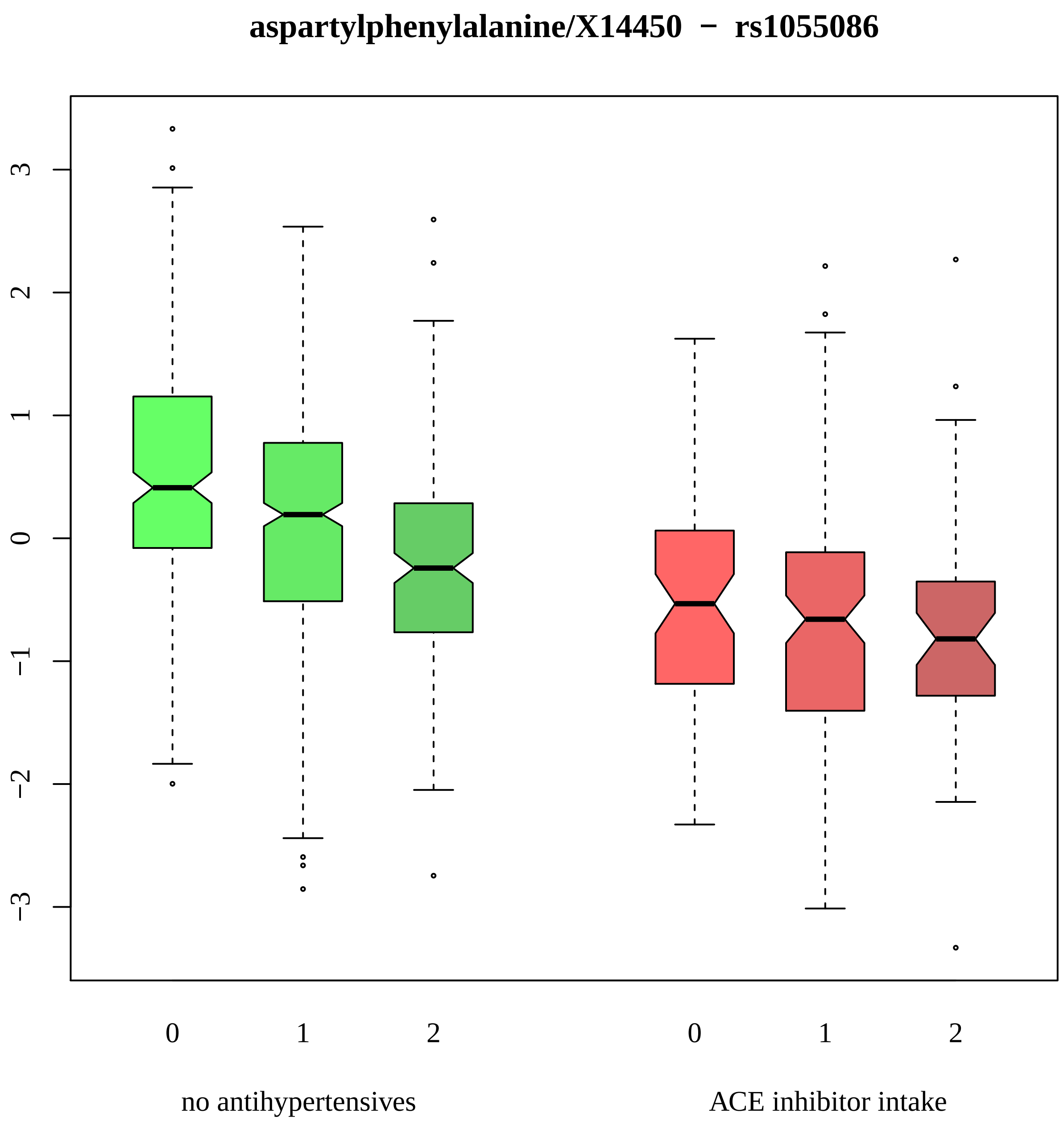

aspartylphenylalanine/X14450 − rs1055086
3
2
1
0
−1
−2
−3
0
1
2
0
1
2
no antihypertensives
ACE inhibitor intake

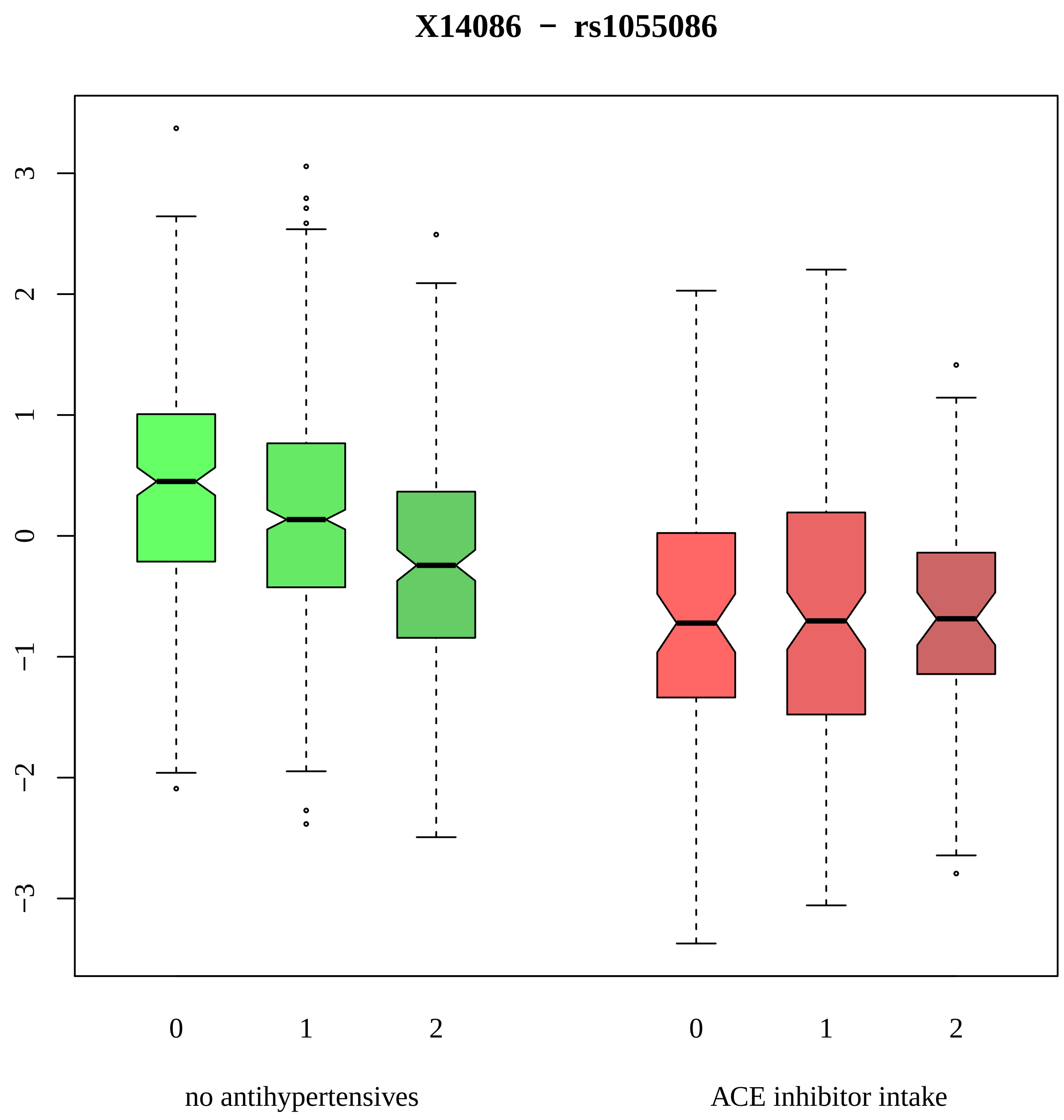

X14086 – rs1055086
3
2
1
0
−1
−2
−3
0
1
2
0
1
2
no antihypertensives
ACE inhibitor intake

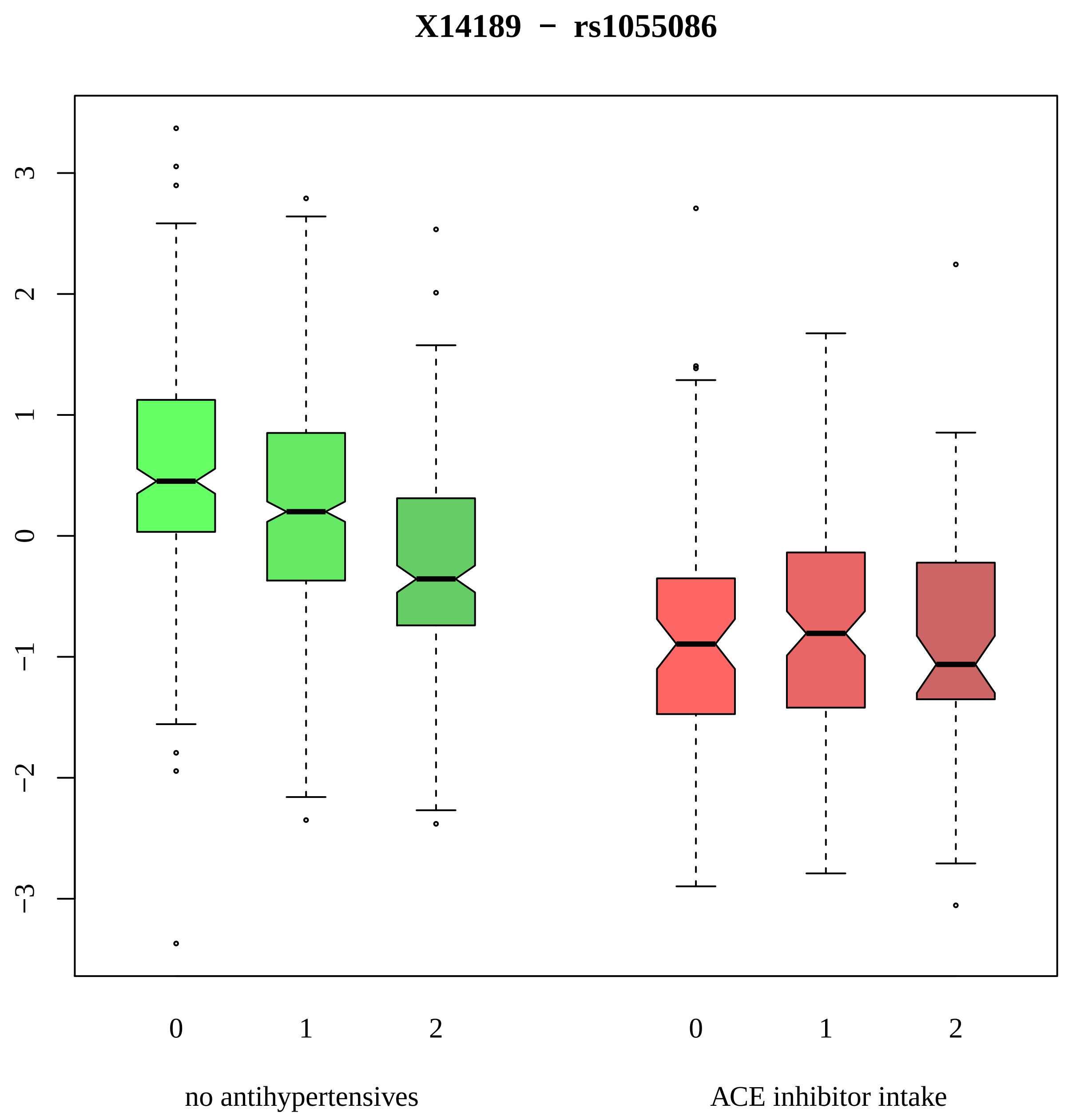
X14189 – rs1055086
3
2
1
0
−1
−2
−3
0
1
2
0
1
2
no antihypertensives
ACE inhibitor intake

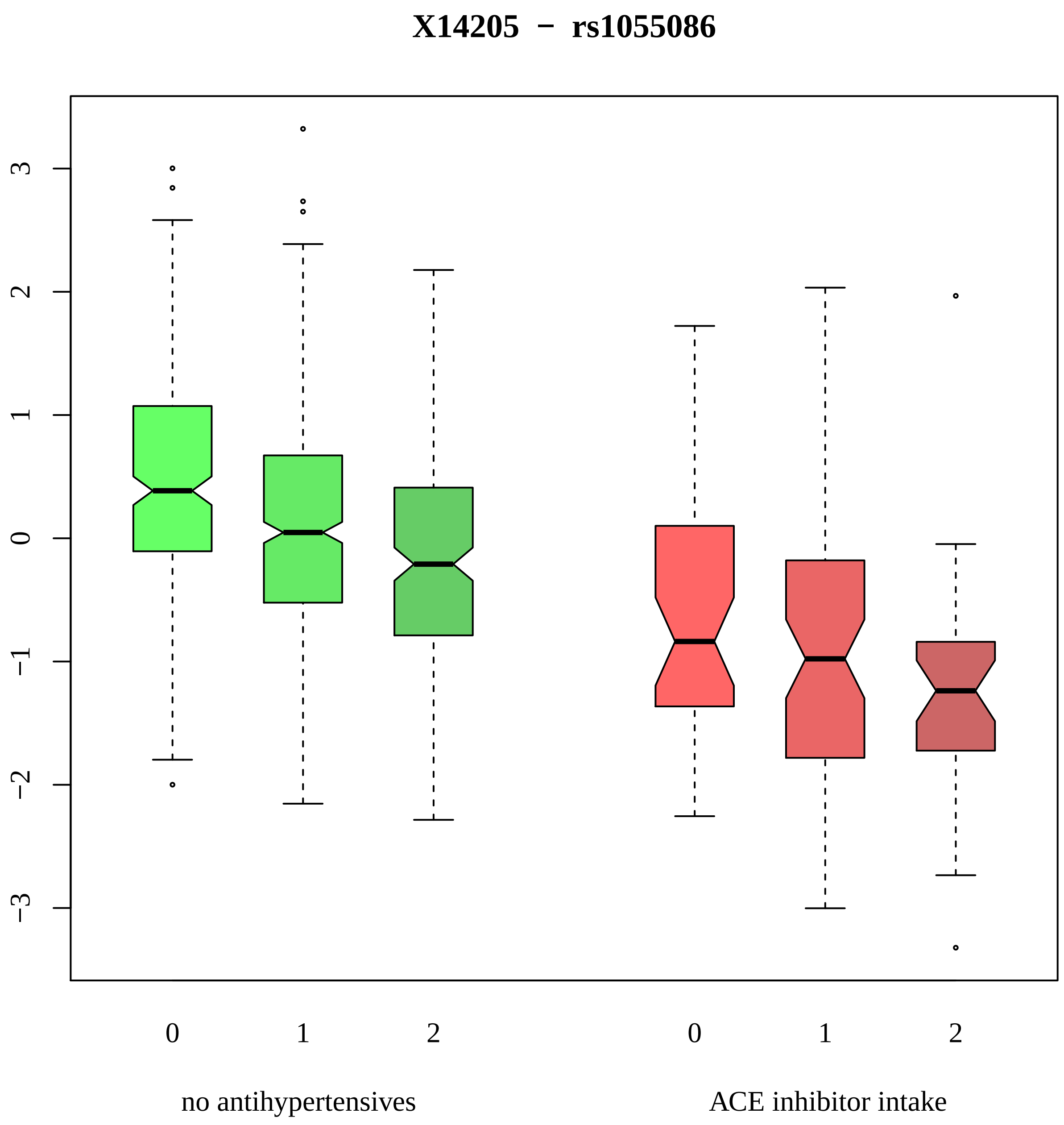
X14205 – rs1055086
3
2
1
0
−1
−2
−3
0
1
2
0
1
2
no antihypertensives
ACE inhibitor intake

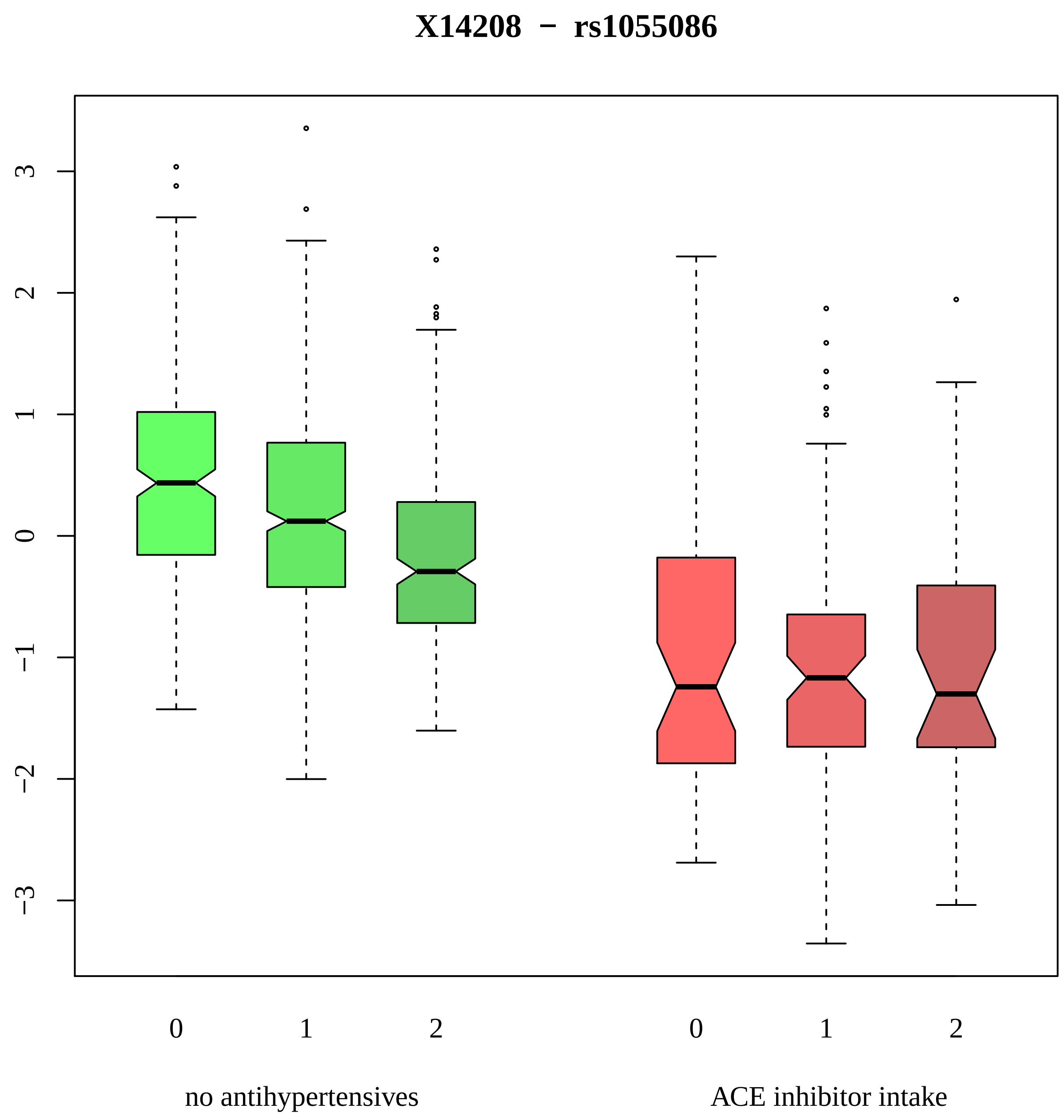

X14208 − rs1055086
3
2
1
0
−1
−2
−3
0
1
2
0
1
2
no antihypertensives
ACE inhibitor intake

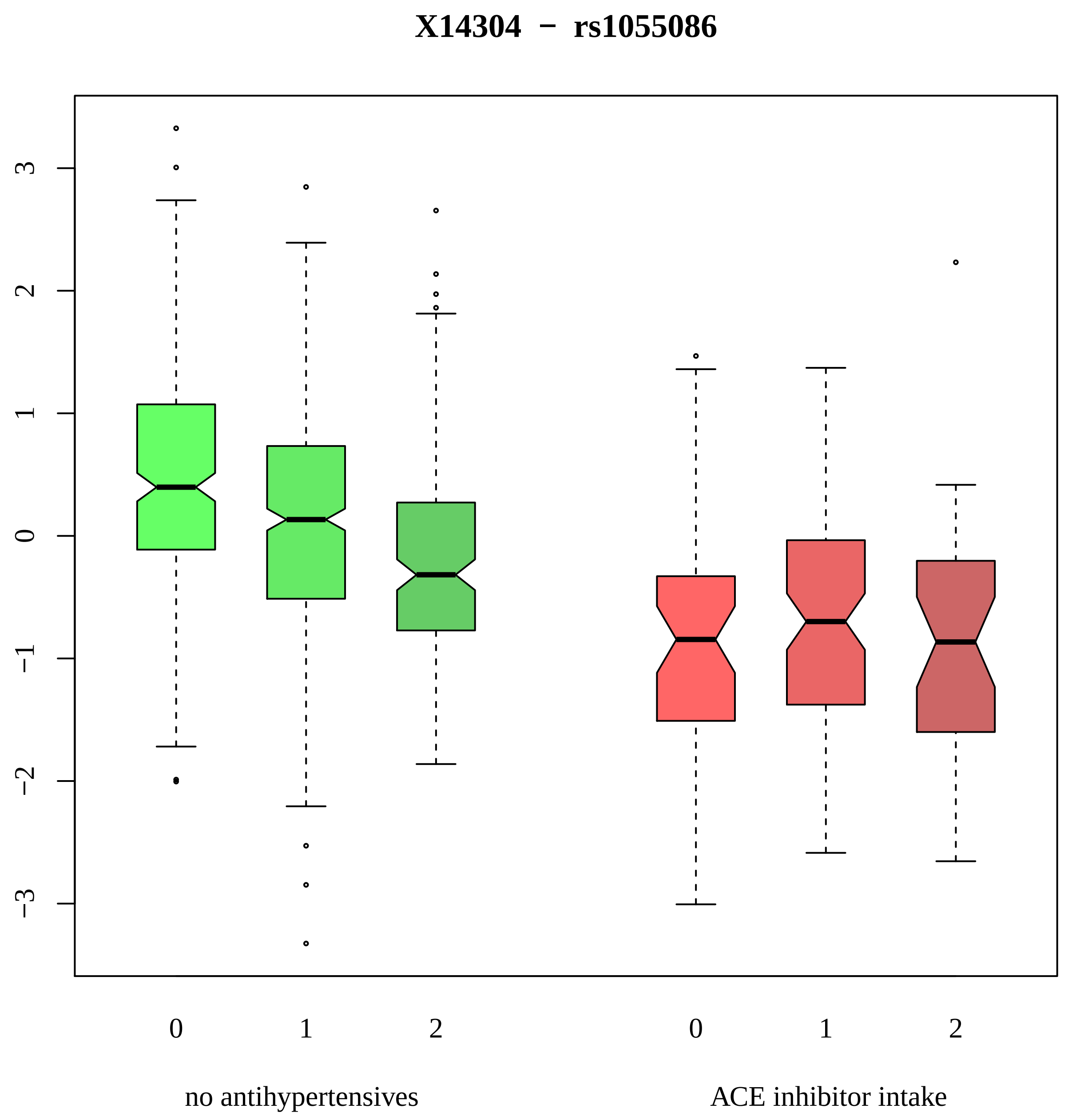
X14304 – rs1055086
3
2
1
0
−1
−2
−3
0
1
2
0
1
2
no antihypertensives
ACE inhibitor intake
