# Supplementary material for: The Pharmacogenetic Footprint of ACE Inhibition: A Population-Based Metabolomics Study
Source: PLoS One. 2016 Apr 27;11(4):e0153163. doi: 10.1371/journal.pone.0153163 (PMC4847917; doi:10.1371/journal.pone.0153163)

The pharmacogenetic footprint of ACE inhibition: a population based metabolomics study

Authors:

Elisabeth Altmaier, Cristina Menni, Margit Heier, Christa Meisinger, Barbara Thorand, Jan Quell, Michael Kobl, Werner Römisch-Margl, Ana M Valdes, Massimo Mangino, Melanie Waldenberger, Konstantin Strauch, Thomas Illig, Jerzy Adamski, Tim D. Spector, Christian Gieger, Karsten Suhre, Gabi Kastenmüller

Journal: PLOS One

Corresponding author:

Gabi Kastenmüller
Helmholtz Zentrum München, German Research Center for Environmental Health,

Ingolstädter Landstr. 1,
D-85764 Neuherberg, Germany

e- mail: g.kastenmueller@helmholtz-muenchen.de

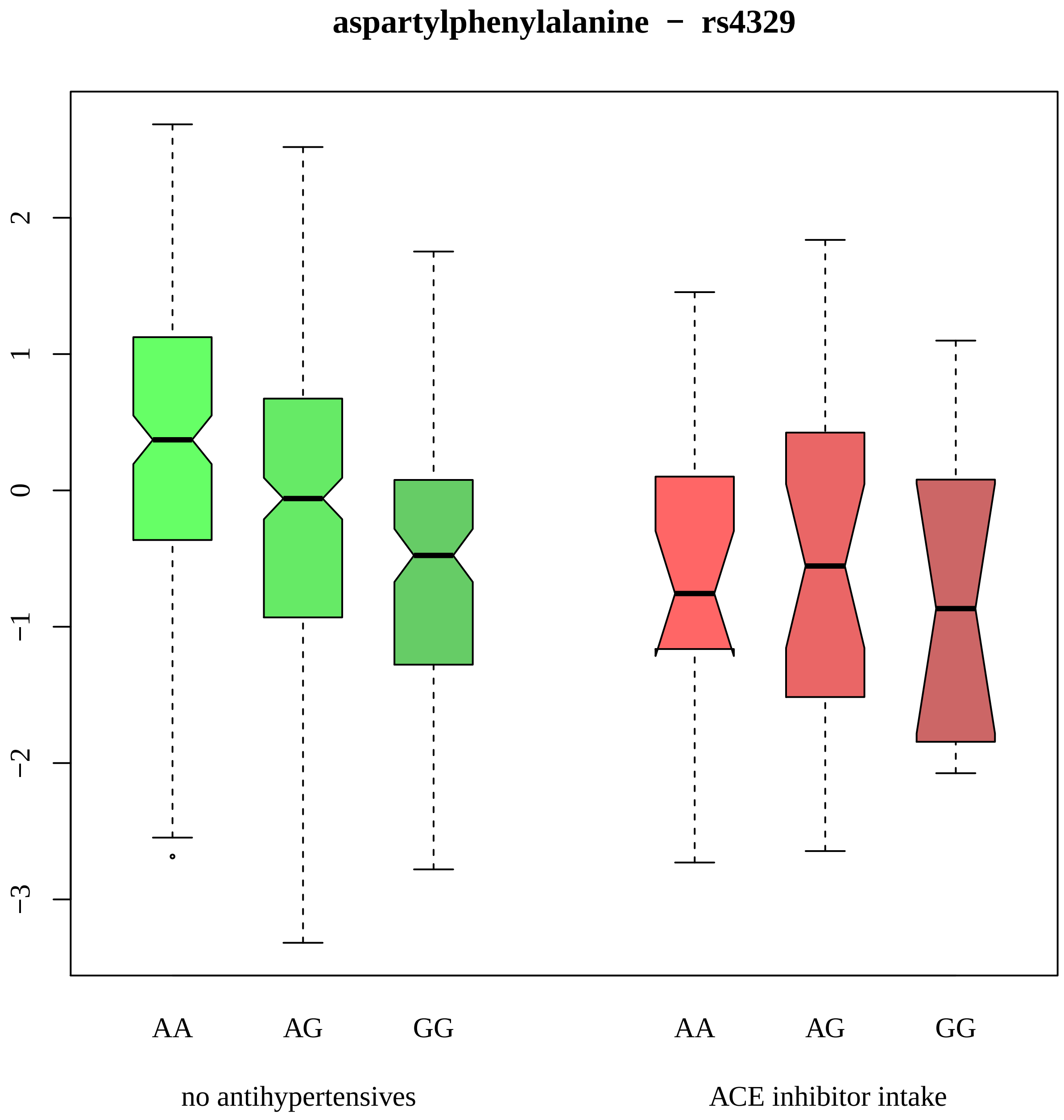

aspartylphenylalanine − rs4329
2
1
0
−1
−2
−3
AA
AG
GG
AA
AG
GG
no antihypertensives
ACE inhibitor intake

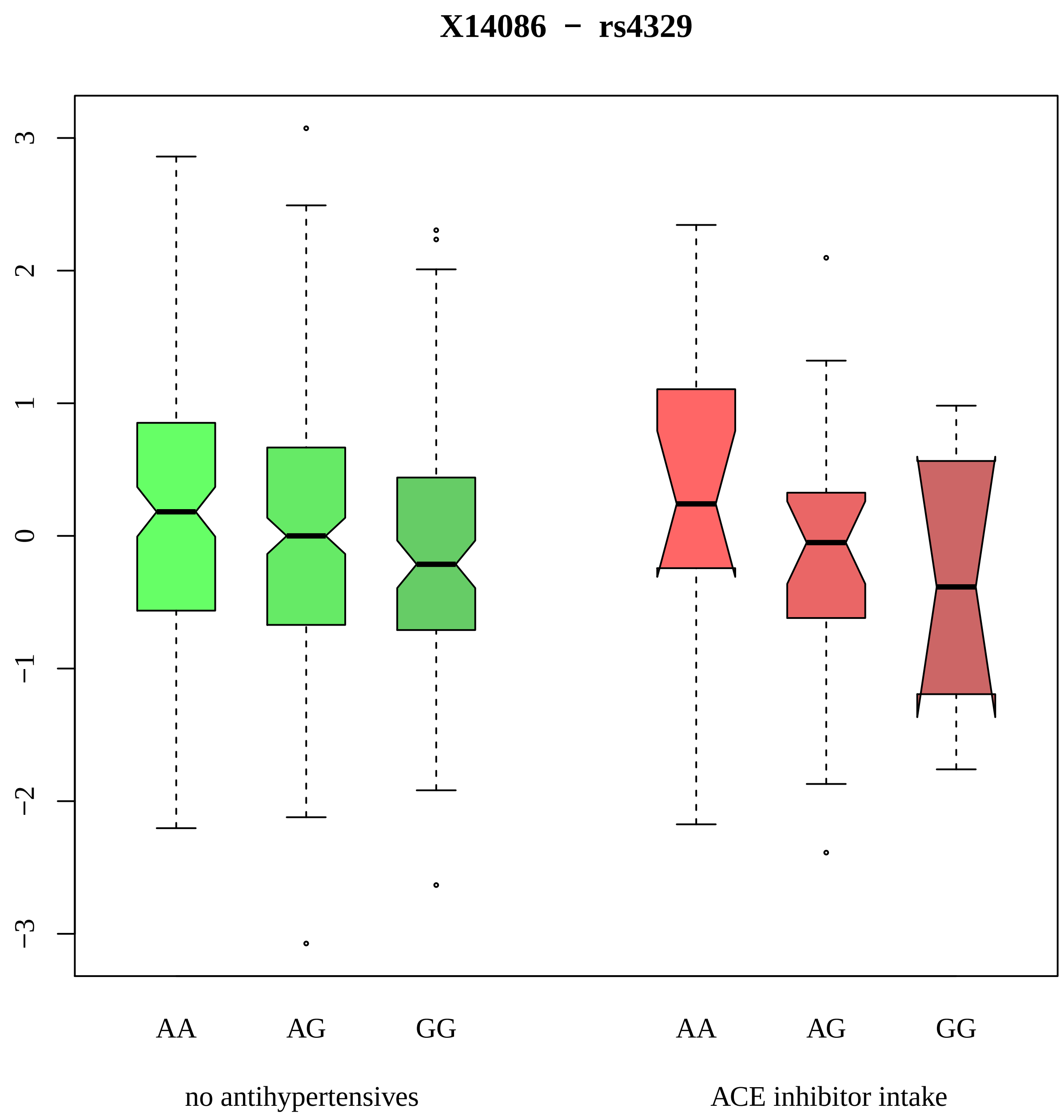
X14086 – rs4329
3
2
1
0
−1
−2
−3
AA
AG
GG
AA
AG
GG
no antihypertensives
ACE inhibitor intake

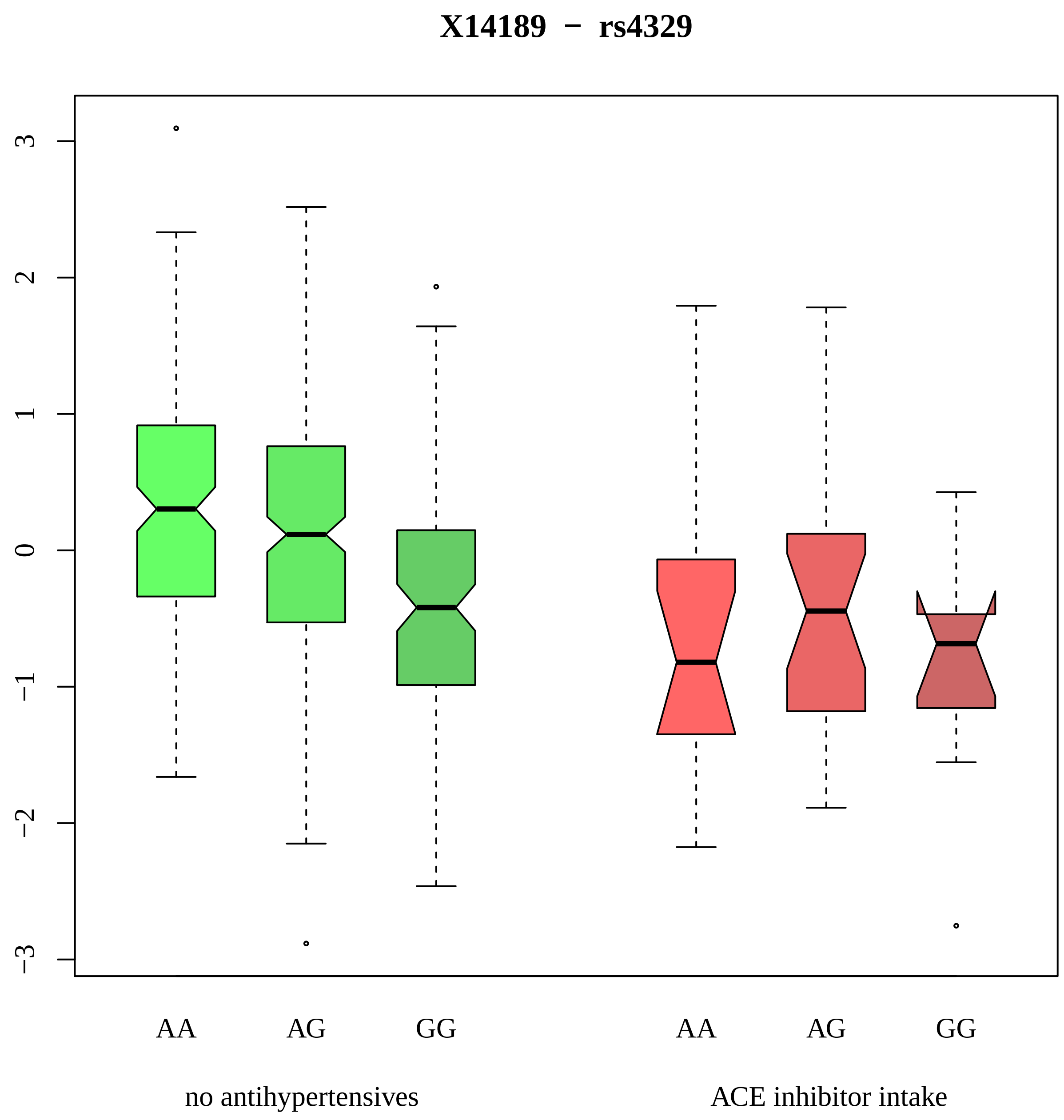
X14189 – rs4329
3
2
1
0
−1
−2
−3
AA
AG
GG
AA
AG
GG
no antihypertensives
ACE inhibitor intake

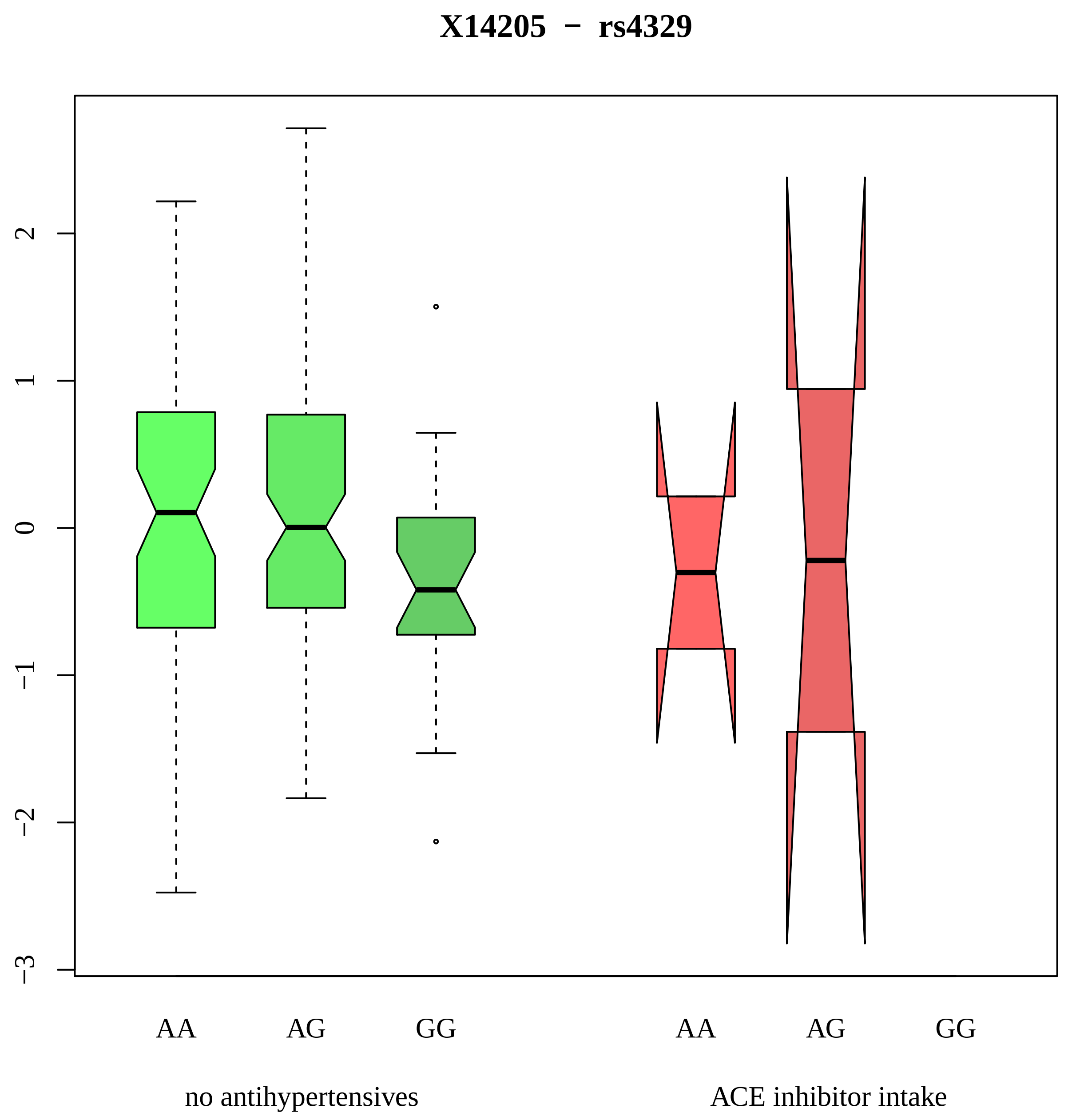

X14205 – rs4329
2
1
0
−1
−2
−3
AA
AG
GG
AA
AG
GG
no antihypertensives
ACE inhibitor intake

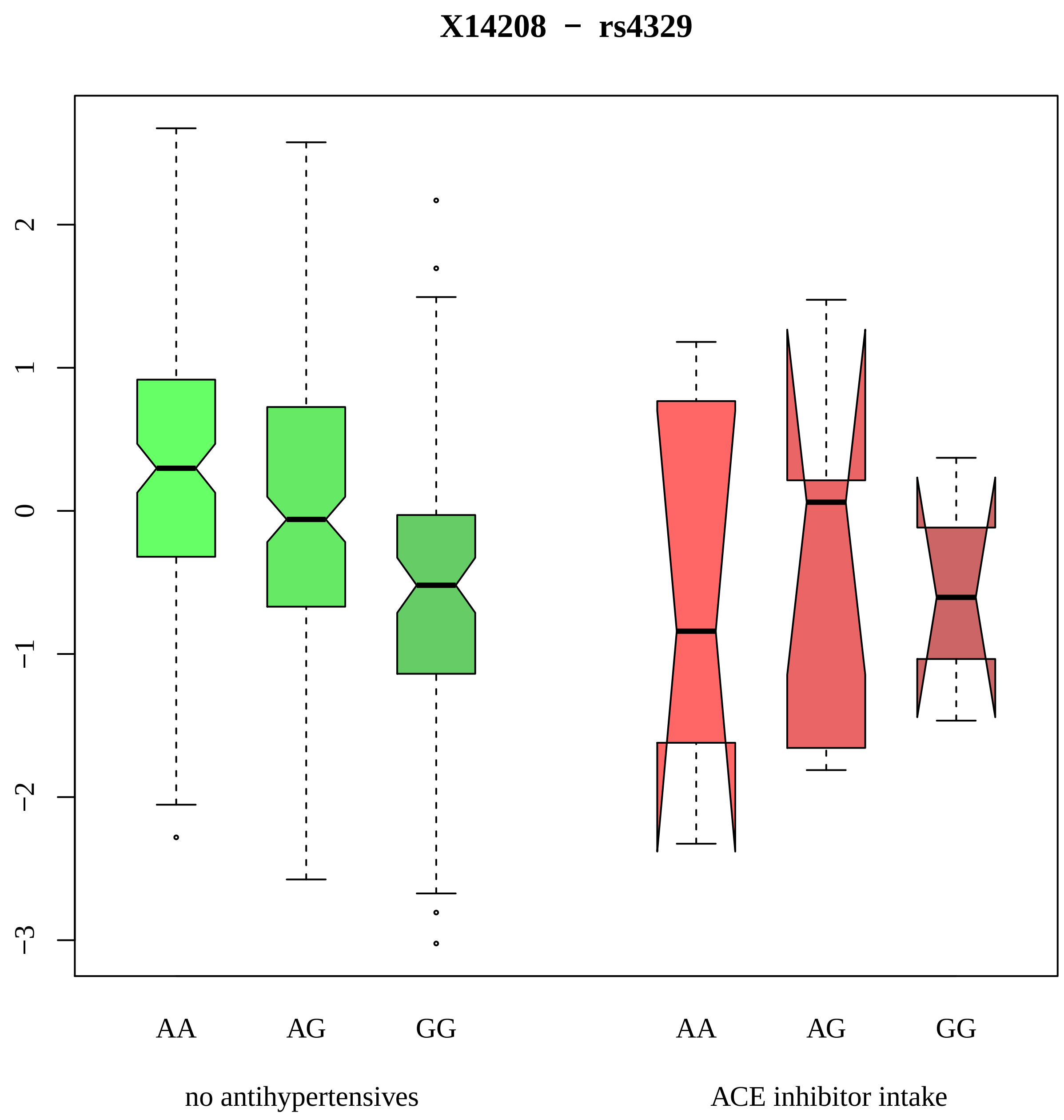
X14208 – rs4329
2
1
0
−1
−2
−3
AA
AG
GG
AA
AG
GG
no antihypertensives
ACE inhibitor intake

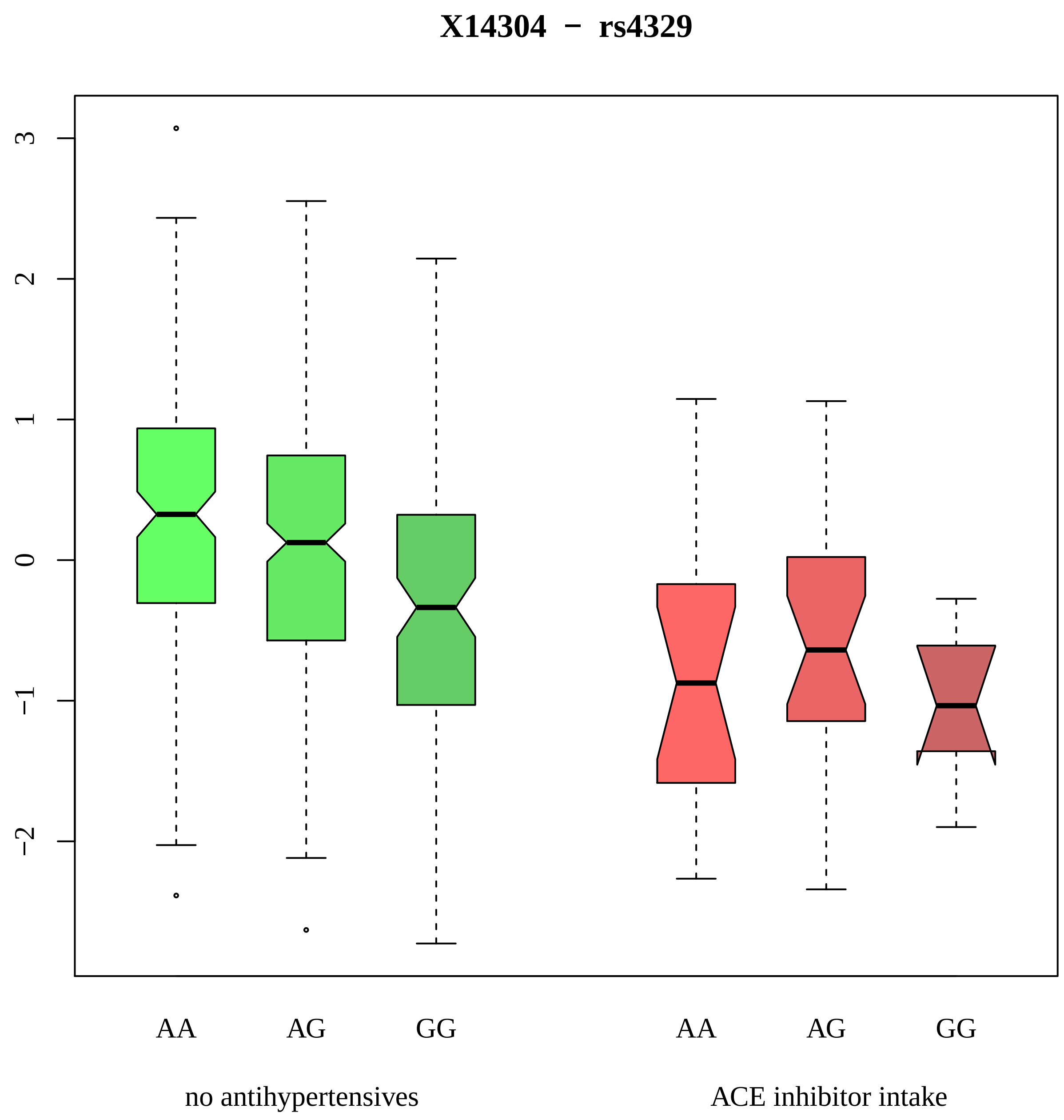
X14304 – rs4329
3
2
1
0
−1
−2
AA
AG
GG
AA
AG
GG
no antihypertensives
ACE inhibitor intake

aspartylphenylalanine/HWESASXX − rs4329

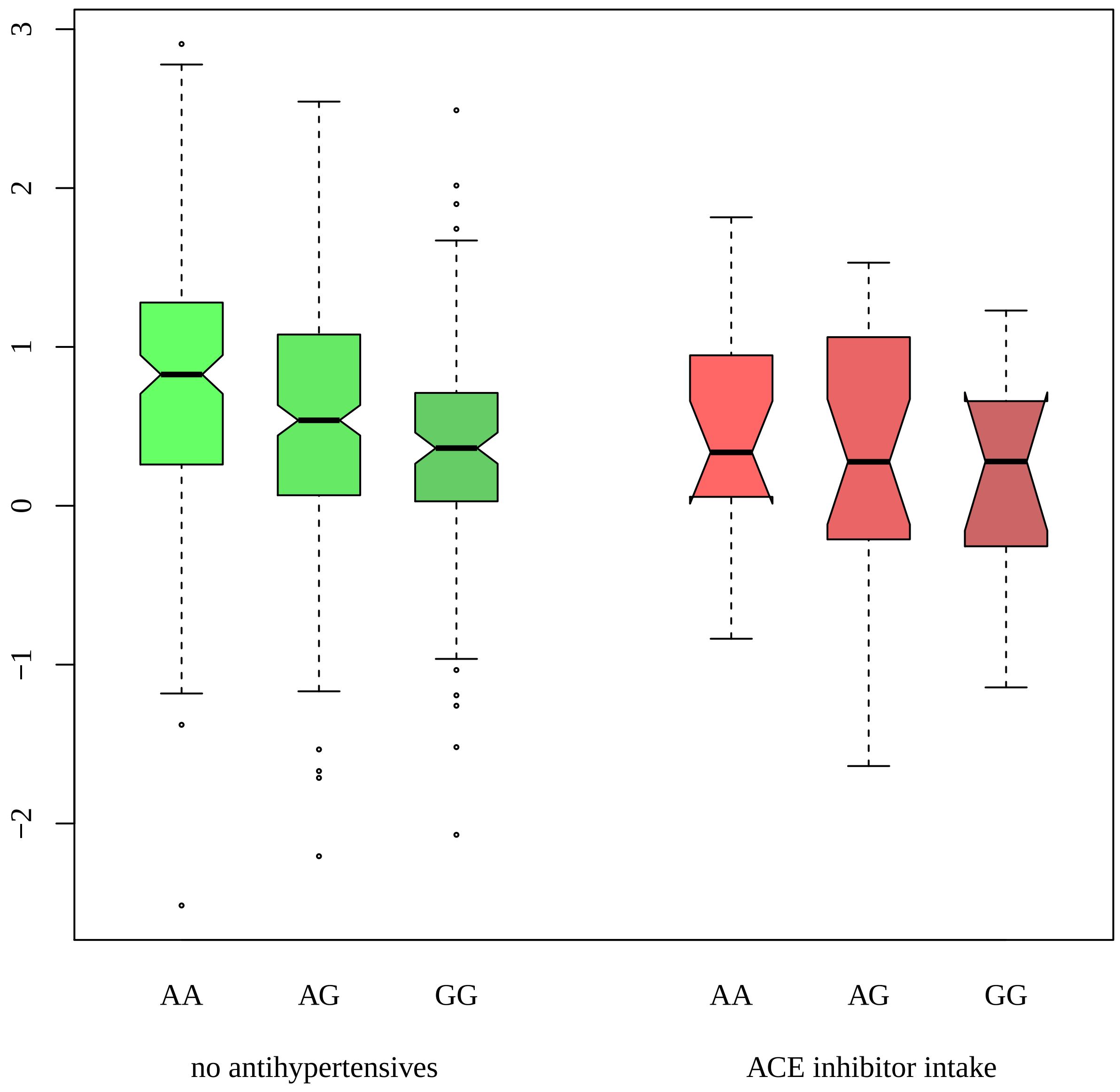

Supplement: S2 Text — (PDF) [file pone.0153163.s006.pdf]
